# Enantioselective Stereodivergent Nucleophile‐Dependent Isothiourea‐Catalysed Domino Reactions

**DOI:** 10.1002/chem.201603318

**Published:** 2016-10-11

**Authors:** Anastassia Matviitsuk, James E. Taylor, David B. Cordes, Alexandra M. Z. Slawin, Andrew D. Smith

**Affiliations:** ^1^ EaStCHEMSchool of ChemistryUniversity of St. Andrews, North HaughSt. AndrewsFifeKY16 9STUK

**Keywords:** domino reactions, enantioselective synthesis, Lewis base, organocatalysis, α,β-unsaturated acyl ammonium

## Abstract

α,β‐Unsaturated acyl ammoniums generated from the reaction of α,β‐unsaturated 2,4,6‐trichlorophenol (TCP) esters bearing a pendent enone with an isothiourea organocatalyst are versatile intermediates in a range of enantioselective nucleophile‐dependent domino processes to form complex products of diverse topology with excellent stereoselectivity. Use of either 1,3‐dicarbonyls, acyl benzothiazoles, or acyl benzimidazoles as nucleophiles allows three distinct, diastereodivergent domino reaction pathways to be accessed to form various fused polycyclic cores containing multiple contiguous stereocentres.

## Introduction

Domino reaction processes are one of the most useful strategies for the rapid generation of molecular complexity in organic synthesis.^1^ Enantioselective organocatalysis is particularly suited to the development of complex tandem or domino processes due to the wide range of distinct activation modes accessible, the ease with which these can be combined, and the high levels of chemo‐ and stereoselectivity often obtained.^2^

**Figure 1:**
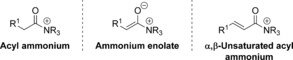
Intermediates accessible from the carboxylic acid oxidation level using tertiary amine Lewis base catalysts.

Tertiary amine Lewis base‐catalysed functionalisation of substrates at the carboxylic acid oxidation level can provide direct access to different catalytic intermediates that have a wide range of applications (Figure 1). To this end, enantiomerically pure catalysts based upon either the DMAP or PPY scaffolds,^3^ cinchona alkaloids,^4^ or isothioureas^5^ are the most widely used. Of the intermediates directly accessible at the carboxylic acid oxidation level using these catalysts, acyl ammonium and ammonium enolates have been the most extensively studied to date and can be utilised in a number of stereoselective processes.^6^ However, the use of α,β‐unsaturated acyl ammonium intermediates generated from stable α,β‐unsaturated carboxylic acid derivatives has received comparatively little attention. Seminal work in this area from Fu and co‐workers used α,β‐unsaturated acyl ammonium intermediates generated from a planar‐chiral DMAP catalyst and α,β‐unsaturated acyl fluorides in [3+2] annulations with silylated indenes to form highly substituted diquinanes with good stereoselectivity (up to 92:8 d.r. and 89:11 e.r.).^7^

**Scheme 1:**
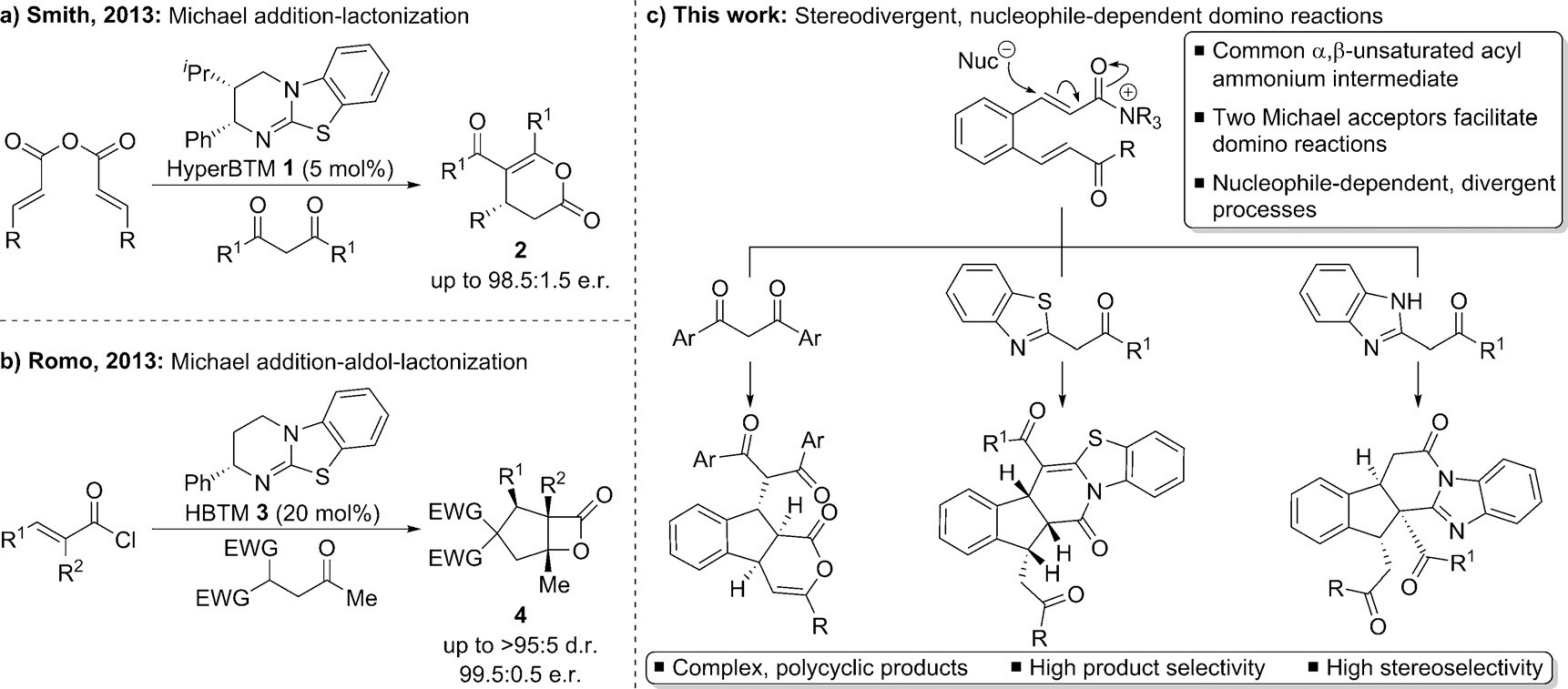
α,β‐Unsaturated acyl ammoniums in domino organocatalytic processes.

Building on this work, we reported the use of an isothiourea catalyst and homoanhydrides as α,β‐unsaturated acyl ammonium precursors in Michael addition‐lactonization reactions with a range of 1,3‐dicarbonyls to form functionalised dihydropyranones **2**, dihydropyridones, or esters (upon ring‐opening) with high enantioselectivity (Scheme 1 a).8a Recent experimental and computational analysis has revealed the importance of non‐bonding 1,5‐S⋅⋅⋅O interactions in governing the chemo‐ and enantioselectivity of annulations of benzothiazoles.8b Romo and co‐workers subsequently used acid chlorides as α,β‐unsaturated acyl ammonium precursors in enantioselective isothiourea‐catalysed domino Michael addition‐aldol‐lactonization reactions using malonate derivatives as nucleophiles to form functionalised cyclopentanes **4** with high stereoselectivity (Scheme 1 b).9a Romo has also used α‐ and β‐aminomalonates as di‐nucleophiles in Michael addition‐lactamization processes with α,β‐unsaturated acyl ammoniums to form substituted γ‐lactams and piperidones.9b The α,β‐unsaturated acyl ammonium intermediate can also serve as an activated dienophile in highly enantioselective organocatalytic Diels–Alder reactions to form fused γ‐ and δ‐lactones.9c Matsubara and co‐workers have recently prepared enantiomerically enriched 1,5‐benzothiazepines through reaction of 2‐aminothiophenols with α,β‐unsaturated acyl ammoniums generated from mixed anhydrides and an isothiourea organocatalyst.^10^

To date, these are the only reported examples investigating the use of α,β‐unsaturated acyl ammonium intermediates.^11^ Further studies into the reactivity and synthetic applicability of these species using readily available tertiary amine based catalysts are required to determine their versatility. To demonstrate the potential of these intermediates, in this manuscript we envisioned that introducing a second Michael acceptor into an α,β‐unsaturated acyl ammonium precursor would allow for the development of more complex domino reaction processes (Scheme 1 c). Addition of suitable nucleophiles into such an α,β‐unsaturated acyl ammonium would initiate a domino process that can utilize the latent ammonium enolate and acyl ammonium reactivity present within the system. Moreover, using pro‐nucleophiles that contain multiple potential sites of reactivity may further increase the molecular complexity accessible in these processes. In this case, the challenge is to generate highly chemo‐, regio‐ and stereoselective processes that favour one specific domino reaction pathway over all others. This is particularly difficult given the multiple electrophilic and nucleophilic sites within the reactants, and such domino processes have not been previously investigated using tertiary amine based catalysis.

Herein the successful realisation of these ideas is reported using an isothiourea‐derived α,β‐unsaturated acyl ammonium generated from bench‐stable activated ester precursors. To the best of our knowledge, these processes are also the first demonstration of using an activated ester as an α,β‐unsaturated acyl ammonium precursor. The exact domino reaction pathway followed is dependent on the intrinsic reactivity within each class of pro‐nucleophile used, which has allowed three distinct and stereodivergent processes to be developed. The fused polycyclic products obtained contain multiple contiguous stereocentres and have complex molecular topologies. Importantly, in each case the products are formed with high specificity and stereoselectivity.

## Results and Discussion

### Reactions using 1,3‐dicarbonyls as nucleophiles

Investigations into the isothiourea‐catalysed domino process began with the treatment of 1,3‐diphenylpropane‐1,3‐dione **6** with a cinnamic acid derivative bearing an *ortho*‐α,β‐unsaturated ketone substituent. However, no cyclisation products were observed under a range of conditions including the use of various carboxylic acid “activating” agents (such as pivaloyl chloride), isothiourea catalysts and bases.^12^ As in situ formation of a reactive mixed anhydride from the carboxylic acid was unsuccessful, attention turned to the use of activated esters as α,β‐unsaturated acyl ammonium precursors. While the use of a 4‐nitrophenol (PNP) ester gave only traces (<5 %) of the expected cyclisation product,^13^ treating bench‐stable 2,4,6‐trichlorophenol (TCP) ester **5** with diketone **6** in the presence of the isothiourea HyperBTM **1** (20 mol %) using polymer‐supported BEMP as a base gave isomeric fused indanes **7 a** and **7 b** as a 75:25 mixture in 46 % yield and excellent 97.5:2.5 e.r. for **7 a** (Table 1, entry 1).^14^, ^15^, ^16^, ^17^ The minor isomer **7 b** was also formed with high enantioselectivity (>99:1 e.r.).^18^ Control experiments on isolated samples of each isomer showed that the products do not interconvert under the reaction conditions. Further optimisation of this domino process showed that isothioureas such as tetramisole hydrochloride (TM⋅HCl) **8** and benzotetramisole (BTM) **9** were not competent catalysts, returning only starting materials (Table 1, entries 2 and 3). Changing the solvent and reaction stoichiometry had an impact on both yield and selectivity (Table 1, entries 4–6), with the optimal conditions using two equivalents of both diketone **6** and PS‐BEMP in THF at room temperature giving fused 1,2,3‐substituted indane **7 a** as a single diastereoisomer in 60 % yield and >99:1 e.r. (Table 1, entry 6). Under these conditions, no base‐promoted background reaction was observed in the absence of the catalyst (Table 1, entry 7). Reducing the catalyst loading (5 or 10 mol %) still gave **7 a** with excellent selectivity, but led to a reduction in isolated yield (48 and 53 %, respectively). Finally, the practicality of the process was demonstrated by performing the reaction on a 5 mmol scale, providing 1.2 g of indane **7 a** as a single stereoisomer (Table 1, entry 1).

**Table 1:** Optimisation of Domino Michael‐Michael‐lactonization reaction.^[a]^

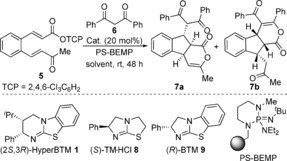						
Entry	Cat.	**5**/**6**/PS‐BEMP	Solvent	Yield [%]^[b]^	**7 a**/**7 b** ^[c]^	e.r. (**7 a**)^[d]^
1	**1**	1:1:1	CH_2_Cl_2_	46	75:25	97.5:2.5
2	**8**	1:1:1	CH_2_Cl_2_	trace	–	–
3	**9**	1:1:1	CH_2_Cl_2_	trace	–	–
4	**1**	1:1:1	MeCN	62	86:14	96.5:2.5
5	**1**	1:2:2	MeCN	70	91:9	95.5:4.5
6	**1**	1:2:2	THF	60	>95:5	>99:1
7	–	1:2:2	THF	–	–	–
8^[e]^	**1**	1:2:2	THF	57	>95:5	>99:1

[a] Reactions performed on 0.1 mmol scale. [b] Combined yield. [c] The **a**/**b** ratio was determined by ^1^H NMR spectroscopic analysis of the crude reaction product. [d] Determined by HPLC analysis. [e] Reaction performed on a 5 mmol scale.

The scope and limitations of the domino process was first explored through variation of the nucleophile (Table 2). Symmetrical aryl substituted diketones bearing electron‐rich, halogen and heterocyclic substituents worked well under the previously optimised conditions, giving fused indanes **10 a**–**12 a** in good yields and excellent diastereo‐ and enantioselectivity.

**Table 2:** Variation of the dicarbonyl nucleophile.^[a–e]^

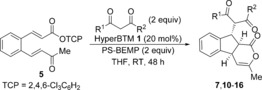
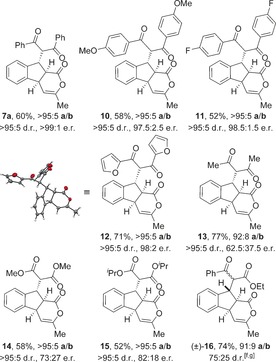

[a] Reactions performed on 0.1 mmol scale. [b] Combined yield. [c] The **a**/**b** ratio was determined by ^1^H NMR spectroscopic analysis of the crude reaction product. [d] The d.r. was determined by ^1^H NMR spectroscopic analysis. [e] The e.r. was determined by HPLC analysis. [f] Reaction performed by using (±)‐HyperBTM **1**. [g] The d.r. at additional stereocentre.

The absolute and relative configuration of **12 a** was confirmed by X‐ray crystallographic analysis, with all other products in this series assigned by analogy.^19^ The use of non‐aryl substituted diketones such as acetylacetone led to a slight drop in product selectivity and a significant reduction in enantioselectivity for **13 a** (62.5:37.5 e.r.), although the diastereoselectivity remained high. A control experiment in the absence of HyperBTM **1** did not lead to product formation, demonstrating that a racemic base‐promoted background reaction is not responsible for the observed drop in enantioselectivity. Malonates are also competent nucleophiles in this process, selectively forming fused products **14 a** and **15 a** with high diastereoselectivity, but with slightly reduced enantioselectivity. The treatment of **5** with non‐symmetrical ethyl benzoylacetate gave indane **16** in 74 % yield, although the additional stereogenic centre was only modestly controlled leading to a 75:25 mixture of diastereoisomers.

Next, a wide range of substituted α,β‐unsaturated TCP esters was subjected to the previously optimised reaction conditions using aryl 1,3‐diketones as nucleophiles (Table 3). The various TCP esters were readily synthesised in four high‐yielding steps from the corresponding substituted 2‐bromobenzaldehyde. Substitution around the benzenoid ring is tolerated, with polycyclic products **17 a**–**19 a** formed with excellent stereoselectivity. Various alkyl and aryl enone substituents were also successfully incorporated. Aryl rings bearing electron‐donating, electron‐withdrawing and halogen substituents all worked well, giving indanes **22 a**–**25 a** with high product selectivity in good yields (up to 69 %) and excellent stereoselectivity in all cases (>95:5 d.r., up to >99:1 e.r.). Combinations of substituted α,β‐unsaturated TCP esters with different aryl 1,3‐diketones were also performed, forming functionalised products **26 a**–**28 a** in good yields with the same high selectivity.

**Table 3:** Variation of the Michael acceptor with 1,3‐diketones.^[a–e]^

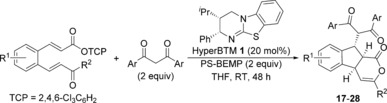
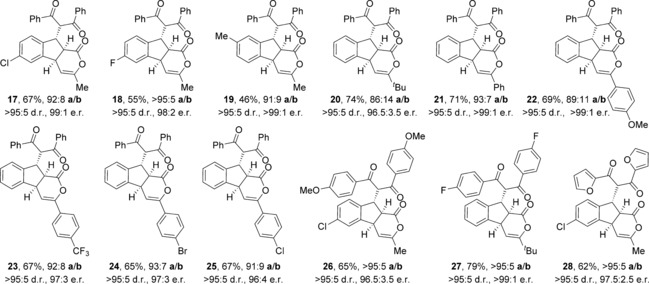

[a] Reactions performed on 0.1 mmol scale. [b] Combined yield. [c] The **a**/**b** ratio was determined by ^1^H NMR spectroscopic analysis of the crude reaction product. [d] The d.r. was determined by ^1^H NMR spectroscopic analysis. [e] The e.r. was determined by HPLC analysis.

**Scheme 2:**
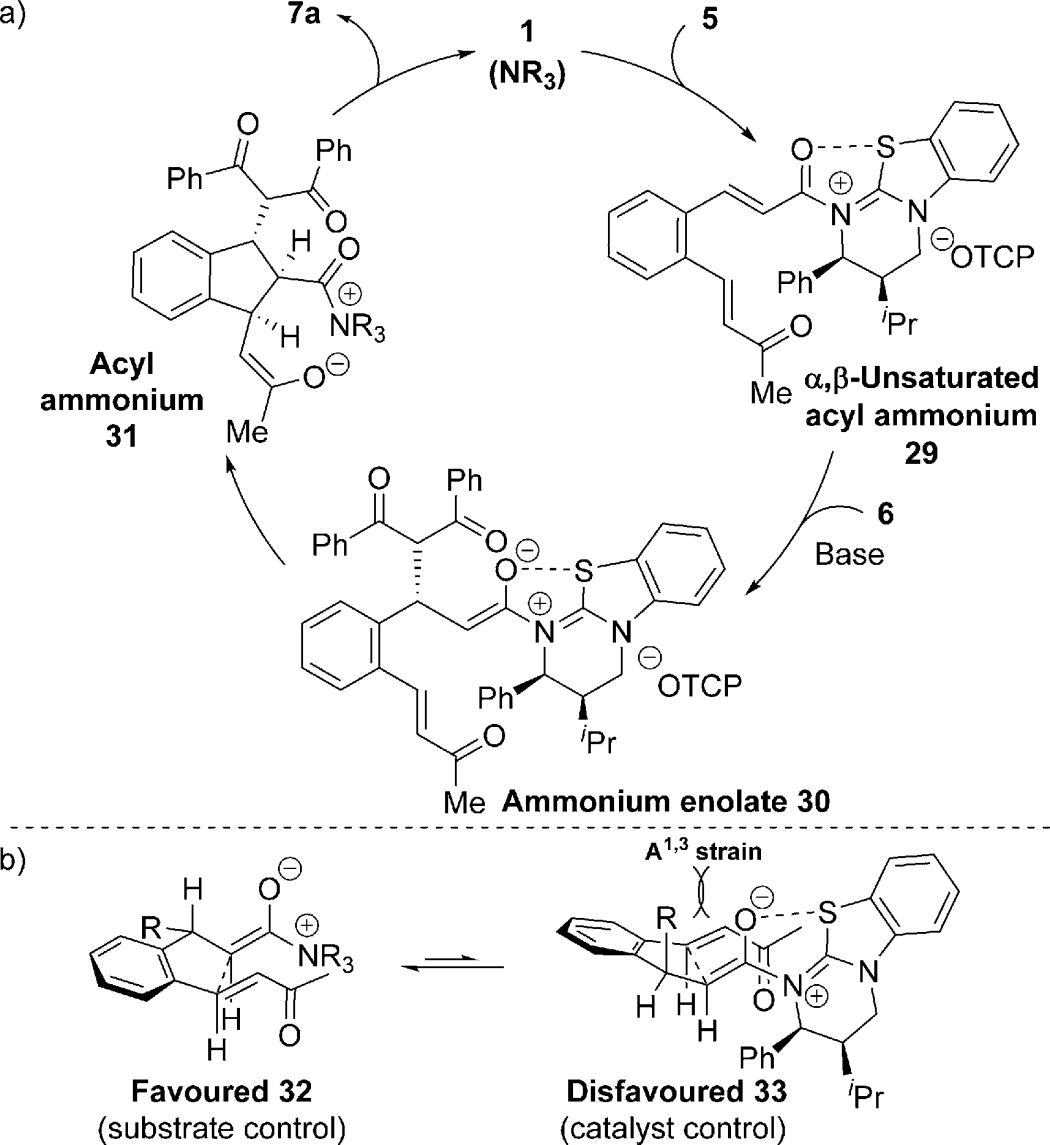
a) Proposed domino Michael‐Michael‐lactonization using 1,3‐dicarbonyls. b) Stereochemical rationale.

The reaction mechanism using 1,3‐dicarbonyls as nucleophiles is proposed to proceed through a domino Michael‐Michael‐lactonization process (Scheme 2 a). Nucleophilic addition of HyperBTM **1** into TCP ester **5** generates an α,β‐unsaturated acyl ammonium intermediate **29**. Michael addition of the enolate of 1,3‐dicarbonyl **6** onto **29** generates ammonium enolate **30**, which undergoes intramolecular Michael addition onto the pendent enone. Lactonization of the resulting enolate onto the acyl ammonium releases polycyclic product **7 a** and regenerates the catalyst. The observed stereochemical outcome is proposed to arise from an initial Michael addition onto the *Re*‐face of α,β‐unsaturated acyl ammonium **29**, which is conformationally locked due to a stabilising non‐bonding O–S interaction (n_O_ to σ*_C–S_), with the *Si*‐face effectively blocked by the stereodirecting groups on the catalyst.^20^ Evidence for such an O–S interaction has previously been obtained both in the solid‐state, through X‐ray analysis of an α,β‐unsaturated acyl isothiourea salt,^8^ and computationally through DFT calculations of possible transition states for a Diels–Alder reaction using an α,β‐unsaturated acyl ammonium.9c Subsequent intramolecular Michael addition of ammonium enolate **30** proceeds under substrate control, with the 1,3‐dicarbonyl, ammonium enolate and enone all adopting pseudo‐equatorial positions in the five‐membered pre‐transition state assembly **32** (Scheme 2 b). Cyclisation under catalyst control is presumably disfavoured due to the presence of A^1,3^ strain between the 1,3‐dicarbonyl substituent and the ammonium enolate.^21^

### Reactions using acyl benzothiazoles as nucleophiles

**Scheme 3:**
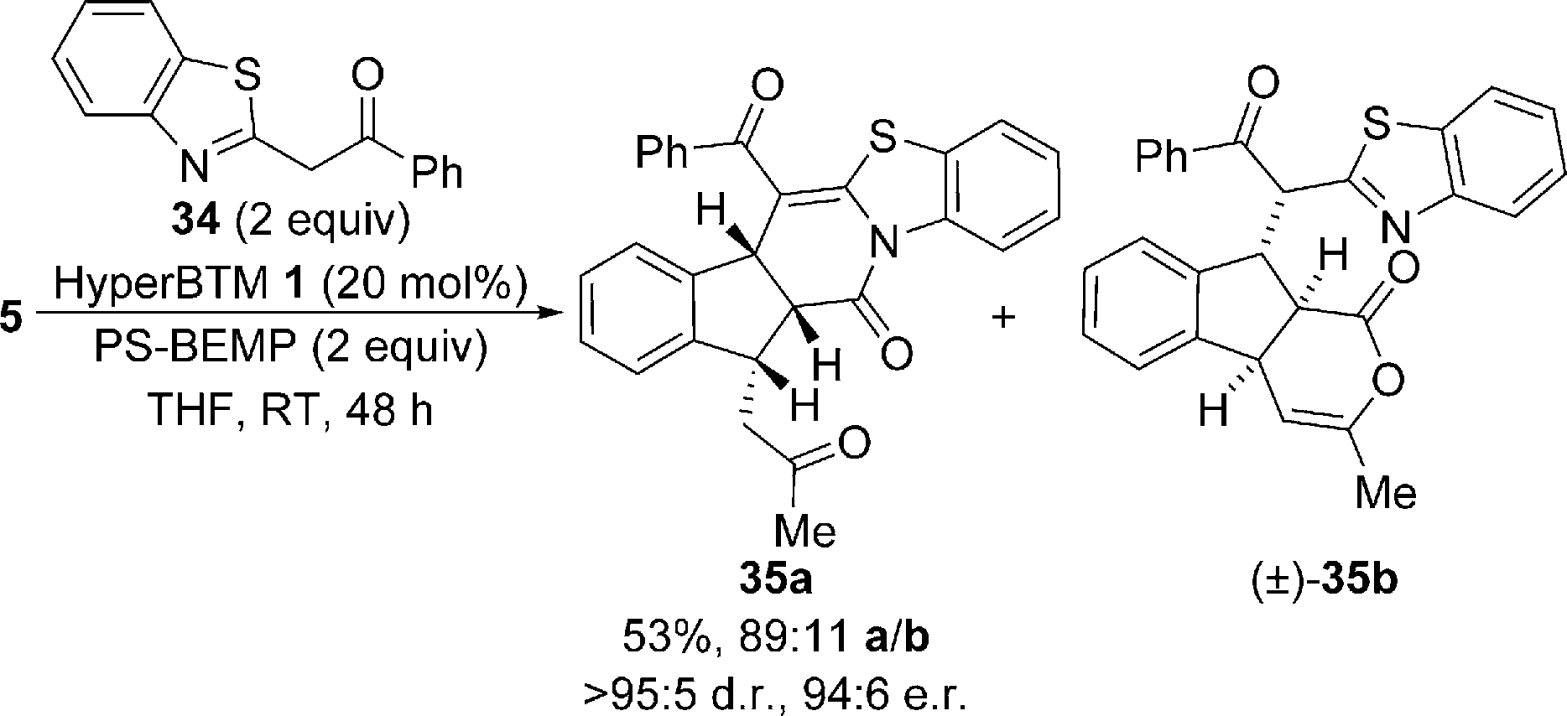
Reaction with 2‐phenacyl benzothiazole **34**.

Having explored the use of various 1,3‐dicarbonyls, the use of acyl benzothiazoles as an alternative pro‐nucleophile class in the domino process was investigated. Reacting α,β‐unsaturated TCP ester **5** with 2‐phenacyl benzothiazole **34** under the previously optimised conditions using HyperBTM **1** (20 mol %) as the catalyst gave a separable 89:11 mixture of functionalised polycyclic products **35 a** and **35 b** in 53 % yield (Scheme 3). In this case, major product **35 a** results from preferential cyclisation through the benzothiazole nitrogen, which is consistent with previous observations using this class of nucleophile.^8^ Interestingly, while the diastereo‐ and enantioselectivity of this process remain high (>95:5 d.r., 94:6 e.r.), the relative configuration around the fused indane **35 a** is different to that observed within the major product from the reaction using 1,3‐dicarbonyls. The relative configuration of minor product **35 b** could not be determined, although it is formed as a racemate suggesting that it may arise from a base‐mediated background process. A control experiment in the absence of HyperBTM **1** confirmed the presence of a base‐promoted reaction in this case.^22^

Intrigued by the change in constitution and configuration within the major product, the scope of the domino process using various acyl benzothiazoles as nucleophiles was explored (Table 4). Substitution within the benzenoid ring of the α,β‐unsaturated ester was possible, although the presence of a methyl substituent led to lower enantioselectivity (82:18 e.r. for **36 a**). The presence of an aryl enone substituent worked particularly well, forming indane **38 a** in 83 % yield with high selectivity (93:7 **a**/**b**) and excellent stereoselectivity (>95:5 d.r., 97:3 e.r.). Within the acyl benzothiazole, halogen substitution around the benzenoid ring gave products **39 a** and **40 a** in good yields and high stereoselectivity. A lower yield was obtained for **41 a** bearing an electron‐donating methoxy substituent (20 %), although the stereocontrol remained high. The relative and absolute configuration of **41 a** was confirmed by X‐ray crystallographic analysis,^23^ with all other products assigned by analogy. Various 2‐arylacyl benzothiazole substituents were also tolerated, forming polycyclic products **42 a**–**44 a** with high product selectivity and with good diastereo‐ and enantiocontrol.

**Table 4:** Substrate scope using acyl benzimidazoles as nucleophiles.^[a–e]^

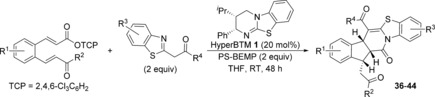
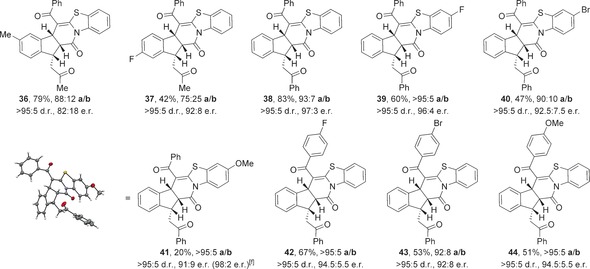

[a] Reactions performed on 0.1 mmol scale. [b] Combined yield. [c] The **a**/**b** ratio was determined by ^1^H NMR spectroscopic analysis of the crude reaction product. [d] The d.r. was determined by ^1^H NMR spectroscopic analysis. [e] The e.r. was determined by HPLC analysis. [f] The e.r. was obtained upon single recrystallisation.

**Scheme 4:**
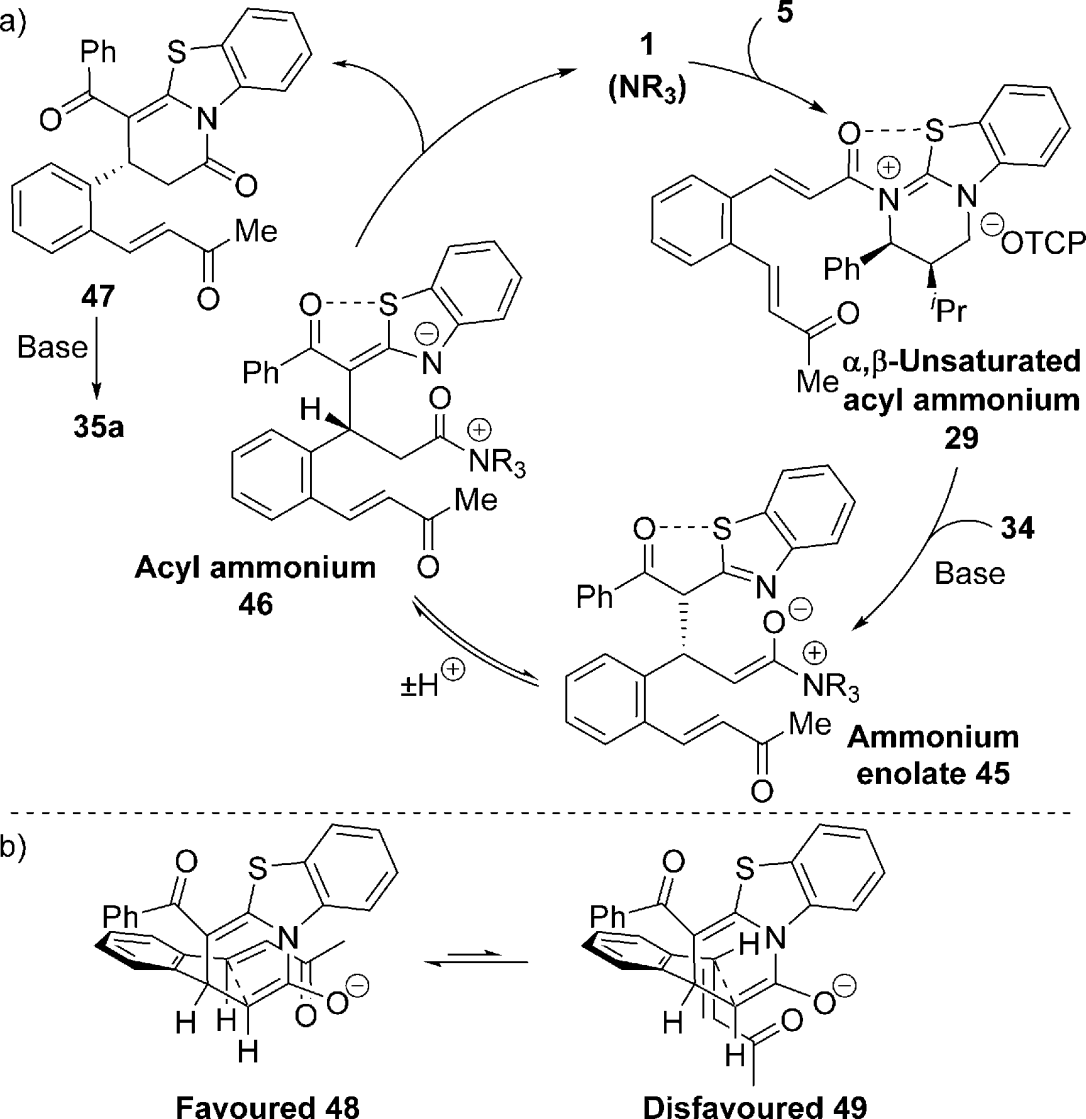
a) Proposed domino Michael‐lactamization‐Michael using acyl benzothiazoles. b) Stereochemical rationale.

The stereodivergence observed in the major products obtained from the reactions using 2‐acyl benzothiazoles compared with 1,3‐dicarbonyls can be rationalised through the operation of an alternative domino Michael‐lactamization‐Michael pathway (Scheme 4 a). After initial Michael addition onto α,β‐unsaturated acyl ammonium **29** the resulting ammonium enolate **45** undergoes preferential proton transfer to give acyl ammonium **46**. Lactamization of the benzothiazole nitrogen onto the acyl ammonium generates dihydropyridone **47** and releases the catalyst. Subsequent base‐mediated cyclisation of **47** generates the observed polycyclic indane **35 a**. In this case, the intramolecular Michael addition occurs through the conformationally restricted enolate of dihydropyridone **47**, with the enone adopting a pseudo‐equatorial position in the forming indane ring (Scheme 4 b).

**Scheme 5:**
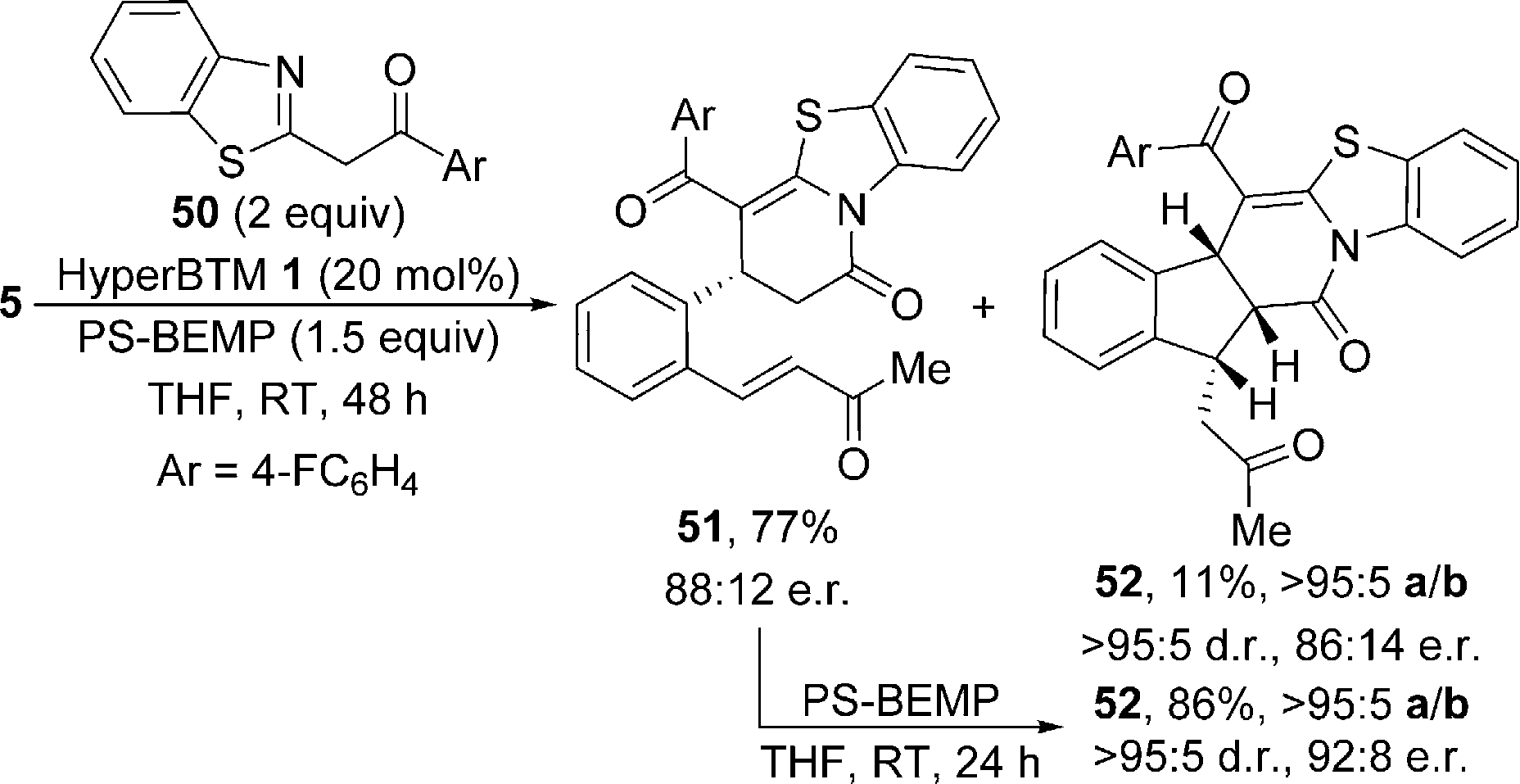
Formation of pre‐cyclised dihydropyridone **51**.

Consistent with this alternative mechanism, treatment of TCP ester **5** with acyl benzothiazole **50** using only 1.5 equiv PS‐BEMP gave dihydropyridone **51** as the major product (77 % yield, 88:12 e.r.), with only 11 % of cyclised product **52** isolated with comparable levels of stereocontrol (Scheme 5). Treating isolated dihydropyridone **51** with PS‐BEMP in the absence of catalyst promoted further cyclisation into **52**, which was obtained in 86 % yield as a single diastereoisomer in 92:8 e.r. This demonstrates that **51** is a viable precursor to indane **52** and the stereochemical outcome of the stepwise cyclisation is consistent with that observed in the domino processes.

### Reactions using acyl benzimidazoles as nucleophiles

**Scheme 6:**
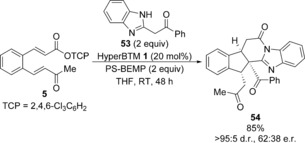
Reaction with 2‐phenacyl benzimidazole **53**.

As acyl benzothiazoles had given a distinct domino reaction pathway, the use of alternative acyl benzazoles was investigated. While treatment of 2‐phenyacyl benzoxazole with α,β‐unsaturated TCP ester **5** under the previously optimised conditions led to a complex isomeric mixture, reaction with 2‐phenyacyl benzimidazole **53** gave a single major product isolated in 83 % yield (Scheme 6). Further characterisation revealed its structure to be fused polycycle **54** containing three contiguous stereocentres, including one quaternary stereocentre. Although **54** was formed as a single diastereoisomer, the enantioselectivity was low (62:6 e.r.).

Intrigued by this observation and the possibility of accessing another distinct domino pathway, the reaction with 2‐phenacyl benzimidazole **53** was optimised (Table 5). A control experiment in the absence of catalyst also led to product **54** in 79 % yield and >95:5 d.r. (Table 5, entry 1), with X‐ray crystallographic analysis confirming the structural assignment and relative configuration.^24^ The presence of a significant racemic base‐promoted background reaction accounts for the low enantioselectivity observed in the presence of HyperBTM **1**. Consequently, the racemic base‐promoted domino reaction of **5** with **53** was first studied. Weaker organic bases such as ^*i*^Pr_2_NEt led to no product formation, but addition of DMAP (20 mol %) gave 31 % of **54** (Table 5, entries 2 and 3). The use of the amidine base DBU led to a complex mixture (Table 5, entry 4), therefore PS‐BEMP was chosen for further study. The domino process was more efficient in CH_2_Cl_2_ compared with either THF or MeCN and the reaction stoichiometry could be reduced to 1.5 equivalents of both **53** and PS‐BEMP, giving **54** in 90 % yield as a single diastereoisomer (Table 5, entries 5–7). The reaction could be performed on a 3.5 mmol scale, leading to the isolation of 1.3 g of fused polycycle **54** in 84 % yield (Table 5, entry 5).

**Table 5:** Reaction optimisation.^[a]^

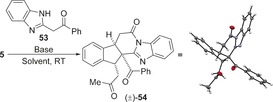						
Entry	Base	Solvent	**5**/**53**/base	*t* [h]	Yield [%]^[b]^	d.r.^[c]^
1	PS‐BEMP	THF	1:2:2	16	79	>95:5
2	^*i*^Pr_2_NEt	THF	1:2:2	16	trace	–
3^[d]^	^*i*^Pr_2_NEt	THF	1:2:2	16	31	>95:5
4	DBU	THF	1:2:2	16	–	–
5	PS‐BEMP	MeCN	1:2:2	16	56	>95:5
6	PS‐BEMP	CH_2_Cl_2_	1:2:2	24	90	>95:5
7	PS‐BEMP	CH_2_Cl_2_	1:1.5:1.5	40	97 (90)^[e]^	>95:5
8^[f]^	PS‐BEMP	CH_2_Cl_2_	1:1.5:1.5	40	(84)^[e]^	>95:5

[a] Reactions performed on 0.1 mmol scale. [b] NMR yield using 1,4‐dinitrobenzene as an internal standard. [c] Determined by ^1^H NMR spectroscopic analysis. [d] Reaction using 20 mol % DMAP. [e] Isolated yield in parentheses. [f] Reaction performed on a 3.5 mmol scale.

The scope and limitations of this process were explored through variation of both the acyl benzimidazole and the α,β‐unsaturated TCP ester (Table 6). Various 2‐arylacyl benzimidazoles containing either electron‐donating, electron‐withdrawing or halogen substituents were tolerated, forming fused indanes **55**–**60** in generally good yield and excellent diastereoselectivity. Introduction of a 2‐furyl substituent gave product **61** in 68 % yield, although the diastereoselectivity was reduced (70:30 d.r.). Substitution around the benzimidazole ring was also well tolerated, giving selective access to polycycles **62**–**64**. The introduction of substituents within the benzenoid ring of the α,β‐unsaturated TCP ester gave the corresponding products **65** and **66** in excellent yield as single diastereoisomers. In contrast with the reactions using acyl benzothiazoles, only an electron‐rich aryl enone substituent could be incorporated, forming product **68** in 80 % yield. The presence of either electron‐withdrawing or halogen substituted aromatic rings on the enone led to mixtures of products and low yields. Notably, the pendent enone could be replaced with an α,β‐unsaturated methyl ester, giving the corresponding indane **69** in 79 % yield with excellent selectivity.

**Table 6:** Substrate scope using acyl benzimidazoles as nucleophiles.^[a, b]^

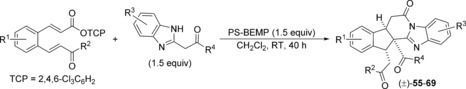
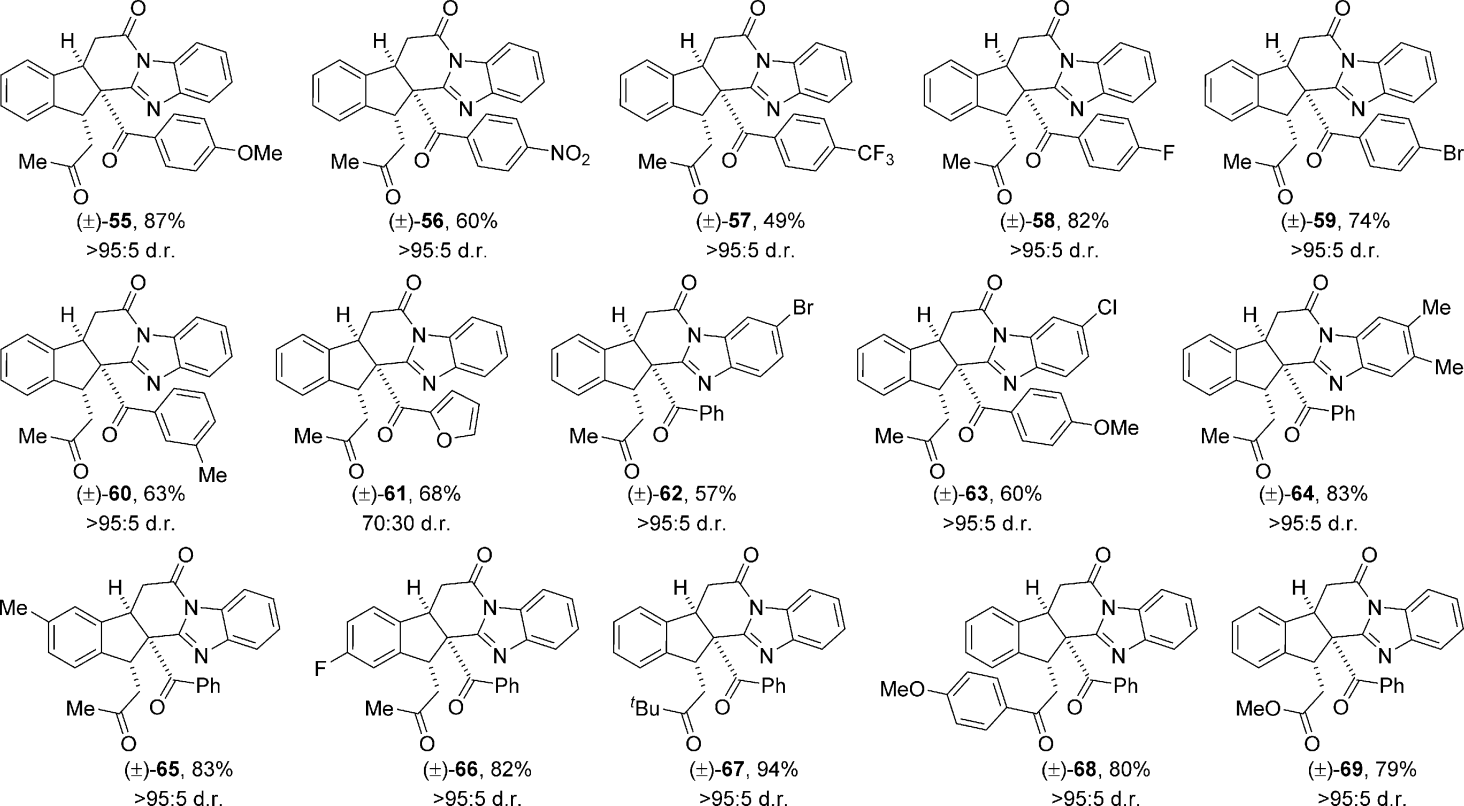

[a] Reactions performed on 0.1 mmol scale. [b] The d.r. was determined by ^1^H NMR spectroscopic analysis.

Having demonstrated a wide scope for the diastereoselective domino process using benzimidazoles as nucleophiles, the possibility of an isothiourea‐catalysed enantioselective variant was revisited.^25^ The reaction of TCP ester **5** and benzimidazole **53** under the previously optimised conditions with the addition of HyperBTM **1** (20 mol %) gave product **54** as a single diastereoisomer, but with low enantioselectivity (Table 7, entry 1). Lowering the temperature to 0 °C led to an improvement, with **54** formed in 70.5:29.5 e.r. (Table 7, entry 2). Changing the base used also had an impact on enantioselectivity. While DBU gave a complex mixture, the use of 2,6‐lutidine gave product **54** in an enhanced 83:17 e.r. (Table 7, entries 3 and 4). Finally, using ^*i*^Pr_2_NEt allowed **54** to be isolated in 60 % yield as a single diastereoisomer in 88:12 e.r. (Table 7, entry 7).

**Table 7:** Optimisation of enantioselective reaction.^[a]^

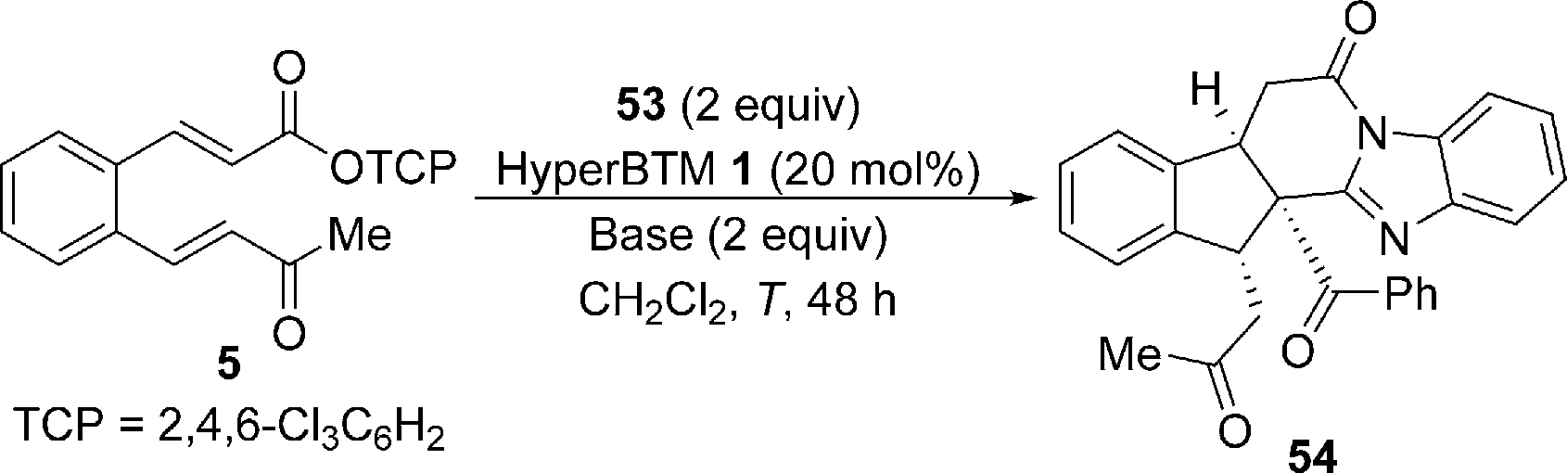					
Entry	Base	*T* [°C]	Yield [%]^[b]^	d.r.^[c]^	e.r.^[d]^
1	PS‐BEMP	RT	97	>95:5	57:43
2	PS‐BEMP	0	65	>95:5	70.5:29.5
3	DBU	0–10	–	–	–
4	2,6‐lutidine	0–10	70	>95:5	83:17
5	^*i*^Pr_2_NEt	0–10	68 (60)^[e]^	>95:5	88:12

[a] Reactions performed on 0.1 mmol scale. [b] NMR yield using 1,4‐dinitrobenzene as an internal standard. [c] Determined by ^1^H NMR spectroscopic analysis. [d] Determined by HPLC analysis. [e] Isolated yield in parentheses.

The newly optimised conditions for the HyperBTM **1**‐catalysed reaction using benzimidazoles were applied to the enantioselective synthesis of a subset of the fused polycycles made previously (Table 8). Structural variation within both the benzimidazole and α,β‐unsaturated TCP ester was tolerated, forming the products in generally good yields with excellent diastereoselectivity and comparable levels of enantioselectivity in each case. The absolute and relative configuration of fused indane **55** was confirmed through X‐ray crystallographic analysis of a recrystallised sample (98.5:1.5 e.r.),^26^ with all other products assigned by analogy.

**Table 8:** Scope of the enantioselective process.^[a–c]^

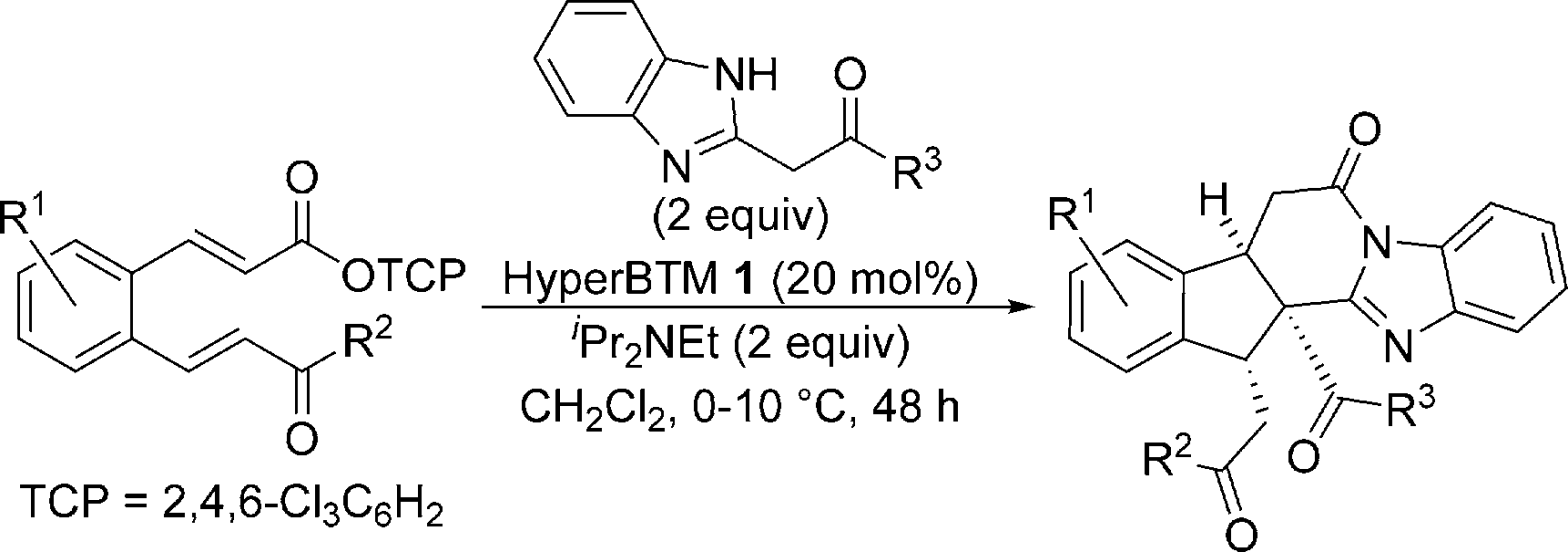
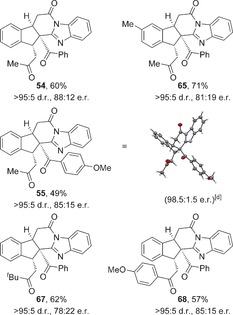

[a] Reactions performed on 0.1 mmol scale. [b] The d.r. was determined by ^1^H NMR spectroscopic analysis. [c] The e.r. was determined by HPLC analysis. [d] The e.r. was obtained upon single recrystallisation.

**Scheme 7:**
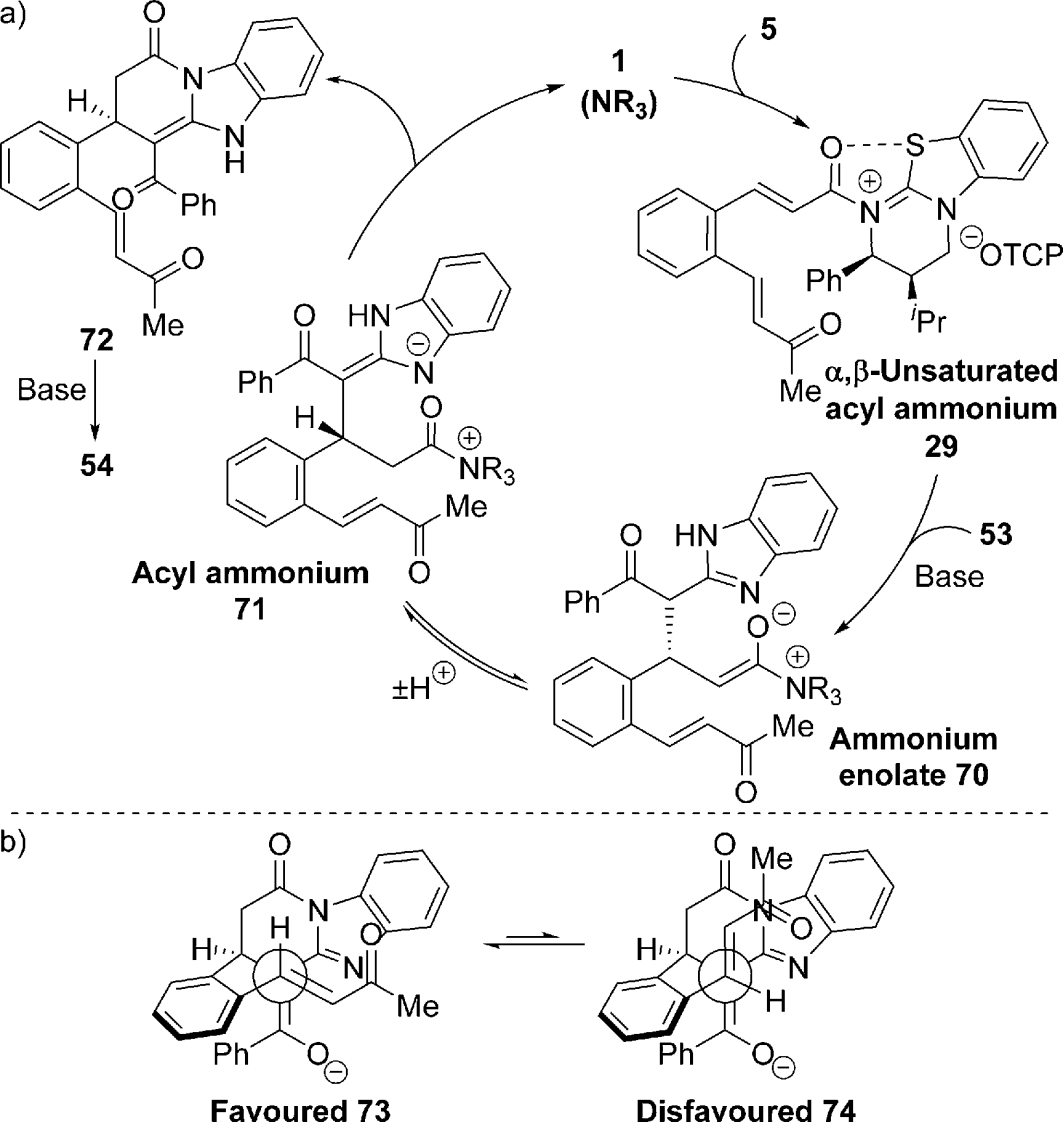
a) Proposed domino Michael‐lactamization‐Michael using acyl benzimidazoles. b) Stereochemical rationale.

Mechanistically, the reactivity and stereoselectivity observed for the reactions using benzimidazoles can be rationalised by the Michael‐lactamization‐Michael domino pathway depicted in Scheme 7 a. Michael addition occurs on the *Re*‐face of α,β‐unsaturated acyl ammonium **29**, with subsequent proton transfer and lactamization of the resulting acyl ammonium **71** generating fused dihydropyridone **72** and releasing the catalyst.

Deprotonation of the benzimidazole followed by intramolecular Michael addition of the enolate formed onto the pendent α,β‐unsaturated ketone gives the observed product **54** containing three contiguous stereocentres, including one all‐carbon quaternary stereocentre. The diastereoselectivity is rationalised by the enone adopting a sterically favoured pseudo‐equatorial position in the forming indane ring during the intramolecular Michael addition (Scheme 7 b). An alternative pathway in which the intramolecular Michael addition occurs from acyl ammonium **71** prior to lactamization cannot be ruled out, however the Michael‐lactamization‐Michael pathway is currently favoured by drawing analogy with the reactions using acyl benzothiazoles.

## Conclusions

α,β‐Unsaturated acyl ammonium intermediates generated from an isothiourea catalyst and bench‐stable α,β‐unsaturated TCP esters bearing pendent Michael acceptors undergo various enantioselective nucleophile‐dependent domino reactions. Three divergent processes are observed by using either 1,3‐dicarbonyls, acyl benzothiazoles, or acyl benzimidazoles as pro‐nucleophiles, forming a range of complex fused polycycles containing multiple contiguous stereocentres with high selectivity and stereocontrol. The different domino processes make use of multiple catalytic intermediates, including α,β‐unsaturated acyl ammonium, ammonium enolate and acyl ammoniums and rely on the intrinsic differences in reactivity within each class of pro‐nucleophile to form the products with high selectivity. Current work in this laboratory is aimed at further developing Lewis base‐catalysed enantioselective transformations.

## Experimental Section

### General

For the synthesis of starting materials, full characterisation data, NMR spectra, and HPLC traces, see the Supporting Information. The research data underpinning this publication can be accessed at DOI: http://dx.doi.org/10.17630/9488831e‐1495‐4e96‐bede‐7e3b4f252018.

### General procedure for the Michael‐Michael‐lactonization reaction with 1,3‐dicarbonyls

HyperBTM **1** (20 mol %), PS‐BEMP (2 equiv), and the appropriate 1,3‐dicarbonyl (2 equiv) were added to a solution of the appropriate α,β‐unsaturated TCP ester in anhydrous THF (0.4 m) at room temperature. The reaction mixture was stirred for 48 h before being filtered to remove the base and concentrated in vacuo. The crude product was purified by column chromatography (petrol/EtOAc) on silica gel to give products of approximately 95 % purity. Analytically pure samples could be obtained through a second chromatographic purification using CH_2_Cl_2_ as eluent.

### General procedure for the Michael‐lactamization‐Michael reaction with acyl benzothiazoles

HyperBTM **1** (20 mol %), PS‐BEMP (2 equiv), and the appropriate acyl benzothiazole (2 equiv) were added to a solution of the appropriate α,β‐unsaturated TCP ester in anhydrous THF (0.4 m) at room temperature. The reaction mixture was stirred for 48 h before being filtered to remove the base and concentrated in vacuo. The crude product was purified by column chromatography (petrol/EtOAc) on silica gel to give products of approximately 95 % purity. Analytically pure samples could be obtained through a second chromatographic purification using CH_2_Cl_2_ as eluent.

### General procedure for the diastereoselective Michael‐lactamization‐Michael reaction with acyl benzimidazoles

PS‐BEMP (1.5 equiv), and the appropriate acyl benzothiazole (2 equiv) were added to a solution of the appropriate α,β‐unsaturated TCP ester in anhydrous CH_2_Cl_2_ (0.4 m) at room temperature. The reaction mixture was stirred for 40 h before being filtered to remove the base and concentrated in vacuo. The crude product was purified by column chromatography (petrol/EtOAc) on silica gel to give products of approximately 95 % purity. Analytically pure samples could be obtained through a second chromatographic purification using CH_2_Cl_2_ as eluent.

### General procedure for the enantioselective Michael‐lactamization‐Michael reaction with acyl benzimidazoles

The appropriate acyl benzimidazole (2 equiv), HyperBTM **1** (20 mol %), and ^*i*^Pr_2_NEt (2.0 equiv) were added to a solution of the appropriate α,β‐unsaturated TCP ester in anhydrous CH_2_Cl_2_ (0.4 m) at 0 °C. The reaction was allowed to warm to 10 °C and stirred for 48 h. The reaction was quenched with 0.1 m HCl and extracted with CH_2_Cl_2_ (×3). The combined organic layers were washed with brine, dried over anhydrous MgSO_4_, filtered and concentrated in vacuo. The crude product was purified by column chromatography (petrol/EtOAc) on silica gel to give products of approximately 95 % purity. Analytically pure samples could be obtained through a second chromatographic purification using CH_2_Cl_2_ as eluent.

## Supporting information

As a service to our authors and readers, this journal provides supporting information supplied by the authors. Such materials are peer reviewed and may be re‐organized for online delivery, but are not copy‐edited or typeset. Technical support issues arising from supporting information (other than missing files) should be addressed to the authors.

Supplementary
