# Supplementary material for: Enantioselective Stereodivergent Nucleophile‐Dependent Isothiourea‐Catalysed Domino Reactions
Source: Chemistry. 2016 Oct 11;22(49):17748–57. doi: 10.1002/chem.201603318 (PMC5132085; doi:10.1002/chem.201603318)
Supplement: Supplementary file 1 — Supplementary [file CHEM-22-17748-s001.pdf]

CHEMISTRY
A European Journal

Supporting Information

Enantioselective Stereodivergent Nucleophile-Dependent Isothiourea-Catalysed Domino Reactions

Anastassia Matviitsuk, James E. Taylor, David B. Cordes, Alexandra M. Z. Slawin, and Andrew D. Smith*[a]

chem_201603318_sm_miscellaneous_information.pdf

Enantioselective Stereodivergent Nucleophile-Dependent Isothiourea-Catalyzed Domino Reactions

Anastassia Matviitsuk,[a] James E. Taylor,[a] David B. Cordes,[a] Alexandra M. Z. Slawin,[a] and Andrew D. Smith[a]*

[a] EaStCHEM, School of Chemistry, University of St Andrews, North Haugh, St Andrews, Fife, KY16 9ST, UK

E-mail: ads10@st-andrews.ac.uk

1 General Information ······ S2

2 General Procedures ······ S3

3 Preparation of Starting Materials ······ S9

3.1 Data for tert-butyl acrylates ······ S9

3.2 Data for phosphoranes ······ S11

3.3 Data for enone tert-butyl esters ······ S12

3.4 Data for enone acids ······ S18

3.5 Data for trichlorophenyl (TCP) esters ······ S24

3.6 Data for 1,3-diketones ······ S31

3.7 Data for acyl benzothiazoles ······ S32

3.8 Data for acyl benzimidazoles ······ S37

4 Michael-Michael-Lactonization with 1,3-Dicarbonyls ······ S44

4.1 Reaction optimization ······ S44

4.2 Data for indanes ***7, 10-28*** ······ S46

5 Michael-Lactamization-Michael with Acyl Benzothiazoles ······ S64

5.1 Screening of alternative acyl benzazoles ······ S64

5.2 Data for indanes ***35-44*** ······ S64

5.3 Formation of pre-cyclised dihydropyridone ***51*** ······ S74

6 Michael-Lactamization-Michael with Acyl Benzimidazoles ······ S76

*6.1 Data for racemic indanes (±)-**54-69*** ······ S76

6.2 Data for enantioenriched indanes ***55, 55, 65, 67, 68*** ······ S92

7 Determination of Stereochemistry ······ S95

7.1 NOE analysis of indane ***7b*** ······ S95

7.2 X-Ray crystal structure of indane ***12a*** ······ S96

7.3 X-Ray crystal structure of indane ***41a*** ······ S96

*7.4 X-Ray crystal structure of indane (±)-**54*** ······ S97

7.5 X-Ray crystal structure of indane ***55*** ······ S97

8 References ······ S98

Appendix I: ^{1}H and ^{13}C{^{1}H} NMR Spectra ······ S99

Appendic II: HPLC Traces ······ S195

1 General Information

Reactions involving moisture sensitive reagents were carried out under a nitrogen atmosphere using standard vacuum line techniques in addition to freshly distilled solvents. All glassware used was flame dried and cooled under vacuum.

Solvents (THF, CH_2Cl_2, toluene, hexane and Et_2O) were obtained anhydrous and purified by an alumina column (Mbraun SPS-800). Anhydrous methanol was obtained by distillation over calcium hydride. All other solvents and commercial reagents were used as supplied without further purification unless stated otherwise.

Room temperature (rt) refers to 20-25 °C. Temperatures of 0 °C and -78 °C were obtained using ice/water and CO_2(s)/acetone baths respectively. Reflux conditions were obtained using a DrySyn metallic heating block equipped with a contact thermometer. *In vacuo* refers to the use of a Büchi Rotavapor® R-2000 rotary evaporator with a Vacuubrand® CVC_2 vacuum controller or a Heidolph Laborota 4001 rotary evaporator with a vacuum controller.

Analytical thin layer chromatography was performed on pre-coated aluminium plates (Kieselgel 60 F_{254} silica). TLC visualisation was carried out with ultraviolet light (254 nm), followed by staining with a 1% aqueous $KMnO_4$ solution. Flash column chromatography was performed on Kieselgel 60 silica in the solvent system stated.

Melting points were recorded on an Electrothermal 9100 melting point apparatus and are uncorrected.

Optical rotations were measured on a PerkinElmer Precisely/Model-341 polarimeter operating at the sodium D line with a 100 mm path cell.

Infrared spectra ($\square_{max}$) were recorded on a Shimadzu IRAffinity-1 Fourier transform infrared spectrophotometer using either thin films or solids with a Pike MIRacle™ ATR accessory. Analysis was performed with Shimadzu IRsolution v1.50 software and only the characteristic peaks are quoted.

HPLC analyses were obtained on a Shimadzu HPLC consisting of a Shimadzu DGU-20A5 degasser, Shimadzu LC-20AT liquid chromatography, Shimadzu SIL-20AT auto sampler, Shimadzu CBM-20A communications bus module, Shimadzu SPD-M20A diode array detector, Shimadzu CTO-20A column oven and a Shimadzu FRC-10A fraction collector. Analysis was performed using Shimadzu LabSolutions v5.42 software and separation was achieved using the column described. All chiral HPLC traces were compared to the authentic racemic trace prepared in analogous fashion.

^{1}H, ^{13}C and ^{19}F nuclear magnetic resonance (NMR) spectra were acquired on either a Bruker Avance 300 (300 MHz, ^{1}H; 75 MHz, ^{13}C; 282 MHz, ^{19}F), Bruker Avance II 400 (400 MHz, ^{1}H; 100 MHz, ^{13}C; 376 MHz, ^{19}F), Bruker Avance 500 (500 MHz, ^{1}H; 125 MHz, ^{13}C; 470 MHz, ^{19}F) or a Bruker Avance III 500 (500 MHz, ^{1}H; 125 MHz, ^{13}C; 470 MHz, ^{19}F) spectrometer at ambient temperature in the deuterated solvent stated. All chemical shifts are quoted in parts per million (ppm) relative to the residual solvent as the internal standard. All coupling constants, *J*, are quoted in Hz and determined by analysis using MestReNova v9.0.0 software. Multiplicities are indicated by: s (singlet), d (doublet), t (triplet), q (quartet), ABq (AB quartet), sept (septet), oct (octet), m (multiplet), dd (doublet of doublets), ddd (doublet of doublet of doublets), dt (doublet of triplets) and td (triplet of doublets). The abbreviation Ar is used to denote aromatic, Ph to denote phenyl, Bn to denote benzyl, br to denote broad br to denote broad and *app* to denote apparent. NMR peak assignments were confirmed using 2D 1H correlated spectroscopy (COSY), 2D 1H nuclear Overhauser effect spectroscopy (NOESY), 2D 1H−13C heteronuclear multiple-bond correlation spectroscopy (HMBC), and 2D 1H−13C heteronuclear single quantum coherence (HSQC) where necessary.

Mass spectrometry (*m/z*) data were acquired by electrospray ionisation (ESI), electron impact (EI) or nanospray ionisation (NSI) at the EPSRC UK National Mass Spectrometry Facility at Swansea University. Low resolution NSI MS was carried out on a Micromass Quattro II spectrometer and high resolution NSI MS on a Thermofisher LTQ Orbitrap XL spectrometer.

2 General Procedures

General Procedure 1: *Heck Reaction*

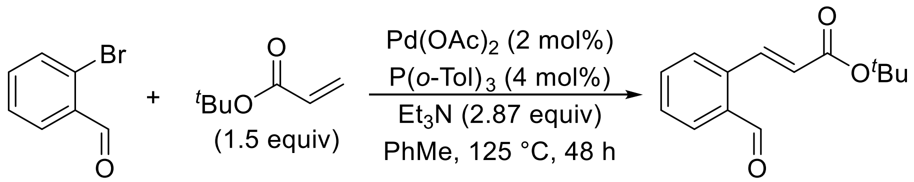

To a solution of the corresponding *o*-bromobenzaldehyde (1.0 equiv) in anhydrous toluene (0.1 M) were successively added $Pd(OAc)_2$ (2 mol%), tri(*o*-tolyl)phosphine (4 mol%), (*E*)-*tert*butylacrylate (1.5 equiv) and triethylamine (2.87 equiv) at room temperature. The reaction mixture was heated at reflux for 2 days, cooled to rt, diluted with ether and filtered through a thin pad of Celite. The filtrate was diluted with water and extracted with ether. The organic layers were combined, dried over $MgSO_4$, and concentrated under

vacuum. The dark thick oil obtained was purified by flash silica chromatography employing mixtures of *n*-hexane and ethylacetate as eluents.

General Procedure 2: *Preparation of Phosphoranes*

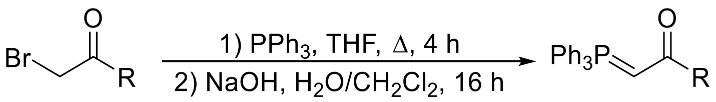

A solution of the corresponding 2-bromoethanone (1.0 equiv) and triphenylphosphine (1.0 equiv) were heated at reflux in anhydrous THF for 4 h. After completion, the reaction mixture was allowed to cool to rt and the phosphonium salt was filtered and washed with Et_2O (×3). The phosphonium salt was then dissolved in H_2O:CH_2Cl_2 (1.5:1) and 2 M aq. NaOH was added. The mixture was stirred overnight at rt and then extracted with CH_2Cl_2 (×3). The combined organic phases were washed with brine (×2), dried ($MgSO_4$) and concentrated *in vacuo* to afford the corresponding phosphorane.

General Procedure 3: *Wittig Reaction*

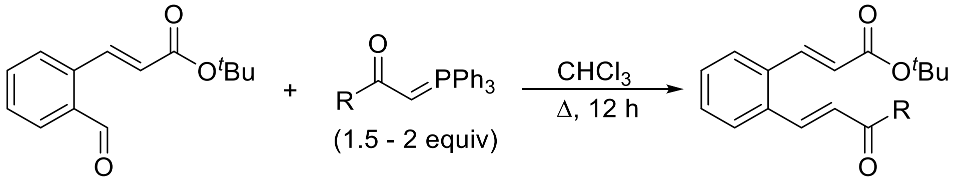

To a solution of (*E*)-*tert*butyl 3-(2-formylphenyl)acrylate (1.0 equiv) in $CHCl_3$, was added the corresponding phosphorane (1.5–2.0 equiv). The reaction mixture was heated at reflux overnight under Ar and then concentrated *in vacuo*. The residue was purified by flash silica column chromatography using a mixture of hexane and ethylacetate (EtOAc) as eluents.

General Procedure 4: *Ester Hydrolysis*

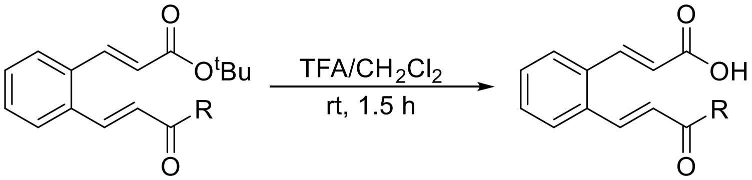

The appropriate ester was dissolved in a mixture of TFA : CH_2Cl_2 (1:2). The reaction was stirred at rt and monitored by TLC until completion. The crude mixture was concentrated *in vacuo* without further purification to give the corresponding carboxylic acid.

General Procedure 5: *Trichlorophenol (TCP) Esterification*

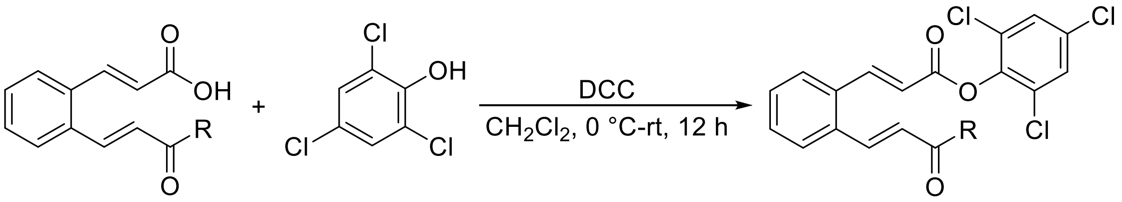

To a solution of the corresponding carboxylic acid (1 equiv) and DCC (1.1 equiv) in CH_2Cl_2 at 0 °C was added 2,4,6-trichlorophenol (TCP-OH) (1.02 equiv) dissolved in CH_2Cl_2 dropwise. The reaction mixture was stirred for 12 h at rt. The crude mixture was filtered to remove the byproduct dicyclohexylurea and concentrated *in vacuo*. The residue was purified by flash silica column chromatography using a mixture of hexane and ethylacetate (EtOAc) as eluents.

General Procedure 6: *Preparation of Diketones*

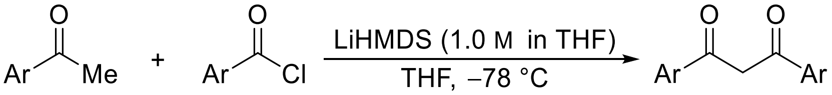

To a solution of arylketone (1.0 equiv) in THF at −78 °C was added LiHMDS (1.0 M in THF, 1.5 equiv) over 15 mins and the resulting mixture stirred at −78 °C for 1 h. Acid chloride (1.2 equiv) was added dropwise as a solution in THF over 5 mins and the solution warmed to room temperature over 1 h and stirred for a further 17 h. The reaction was quenched with 10% citric acid (20 mL) and extracted with EtOAc (2 × 100 mL). The combined organics were washed with H_2O (20 mL), dried over anhydrous $MgSO_4$, filtered and concentrated *in vacuo*. The residue was purified by flash silica column chromatography using a mixture of petrol and diethylether (Et_2O) as eluents.

General Procedure 7: *Preparation of Acyl Benzothiazoles*

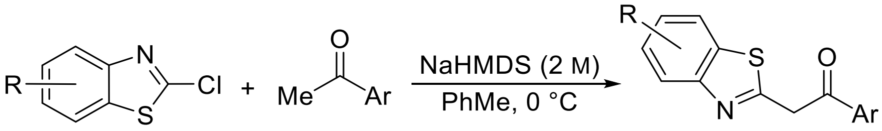

To a degassed solution of chlorobenzoxazole (1 equiv) and carbonyl nucleophile (3 equiv) in anhydrous toluene (0.25–0.35 M) was added NaHMDS (3.0 equiv, 2 M solution in toluene or THF) dropwise at 0 °C, and the solution stirred for 5 h at 0 °C followed by room temperature for 16 h. Excess NaHMDS was quenched by dropwise addition of saturated aqueous NH_4Cl (50 mL) at 0 °C. The organic layer was separated, then the aqueous layer extracted with EtOAc. The combined organic layers were dried over $MgSO_4$, filtered, and concentrated *in vacuo.* The residue was purified as specified to afford the product.

General Procedure 8: *Preparation of Acyl Benzimidazoles*

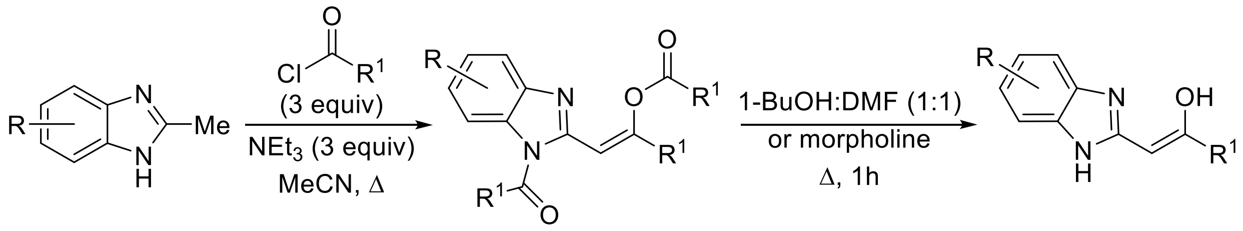

A mixture of 2-methyl-benzimidazole (1 equiv), triethylamine (3.0–3.3 equiv), acetonitrile (1 M), and aroyl chloride (3.0–3.3 equiv) was heated at reflux for 18 h. The cooled reaction mixture was filtered and the precipitate was washed on the filter with 1:1 2-propanol–water, water, and then 2-propanol. After drying, the triacylation product was dissolved in a 1:1 1-butanol–dimethyl formamide mixture and heated at reflux for 1 h. The precipitate was washed with cold 2-propanol and then hexane to give an analytically pure product. If the triacylation product could not be isolated as a solid, the reaction mixture was carefully diluted by adding water (2 mL) and morpholine (6 equiv). Heating was then continued for an additional 15 min. The cooled mass was filtered and washed with cold 2-propanol and then hexane to give an analytically pure product.

General Procedure 9: *Michael-Michael-Lactonization Reaction with 1,3-Dicarbonyls*

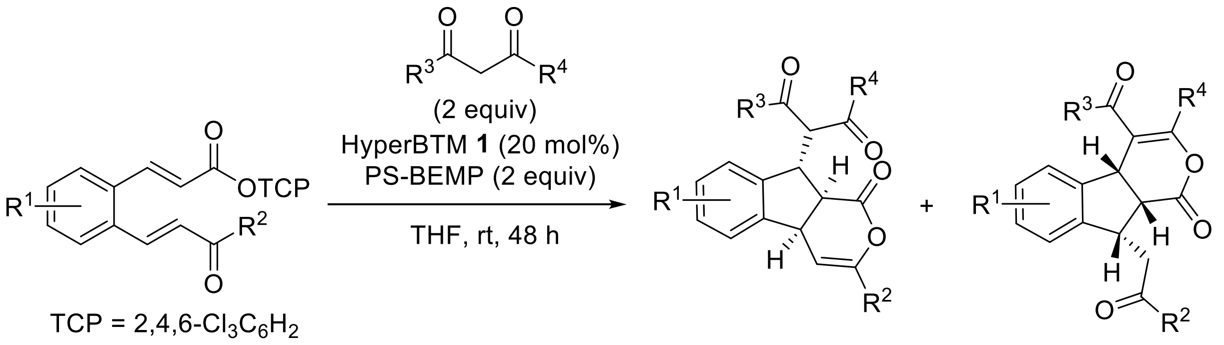

To a solution of the corresponding tricholorophenol-activated ester (1 equiv) in anhydrous THF (0.4 M), was added isothiourea HyperBTM **1** (20 mol%) and polymer-bound 2-*tert*-butylimino-2-diethylamino-1,3,dimethylperhydro,1,3,2,diazaphosphorine (PS-BEMP) (2 equiv) followed by addition of the appropriate 1,3-diketone (2.0 equiv). The reaction mixture was stirred for 48 h at room temperature. The crude mixture was filtered to remove the base and concentrated *in vacuo*. The residue was purified by column chromatography using mixtures of petrolether and ethylacetate as eluents giving the products of approximatly 95% purity. Analytically pure compounds can be obtained after a second purification by column chromatography using dichloromethane as eluent.

General Procedure 10: *Michael-Lactamization-Michael Reaction with Acyl Benzothiazoles*

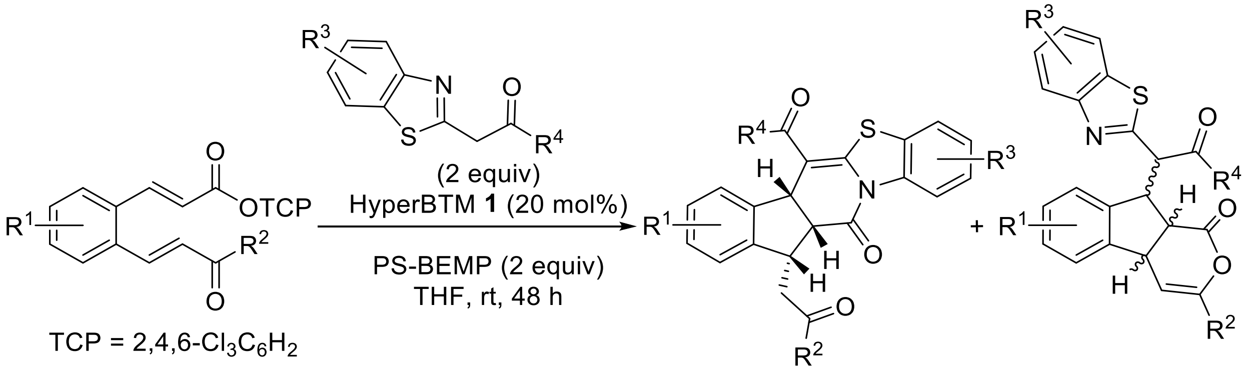

To a solution of the corresponding tricholorophenol-activated ester (1 equiv) in anhydrous THF (0.4 M), was added isothiourea HyperBTM **1** (20 mol%) and polymer-bound 2-*tert*-butylimino-2-diethylamino-1,3,dimethylperhydro,1,3,2,diazaphosphorine (PS-BEMP) (2 equiv) followed by addition of a the appropriate acyl benzothiazole (2.0 equiv). The reaction mixture was stirred for 48 h at room temperature. The crude mixture was filtered to remove the base and concentrated *in vacuo*. The residue was purified by column chromatography using mixtures of petrolether and ethylacetate as eluents giving the products of approximatly 95% purity. Analytically pure compounds can be obtained after a second purification by column chromatography using dichloromethane as eluent.

General Procedure 11: *Racemic Michael-Lactamization-Michael Reaction with Acyl Benzimidazoles*

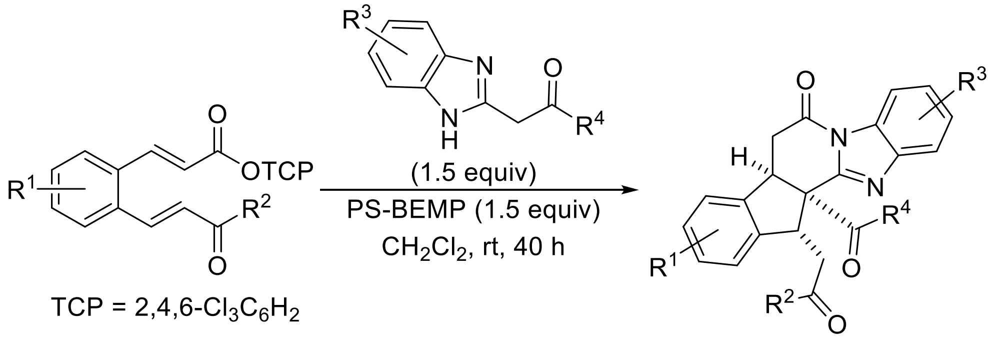

To a solution of the corresponding tricholorophenol-activated ester (1 equiv) in anhydrous CH_2Cl_2 (0.4 M), was added polymer-bound 2-*tert*-butylimino-2-diethylamino-1,3,dimethylperhydro-1,3,2,diazaphosphorine (PS-BEMP) (1.5 equiv) followed by addition of a benzimidazole (1.5 equiv). The reaction mixture was stirred for 40 h at room temperature. The crude mixture was filtered to remove the base and concentrated *in vacuo*. The residue was purified by column chromatography using mixtures of petrolether and ethylacetate as eluents giving the products of approximatly 95% purity. Analytically pure

compounds can be obtained after a second purification by column chromatography using dichloromethane as eluent.

General Procedure 12: *Asymmetric Michael-Lactamization-Michael Reaction with Acyl Benzimidazoles*

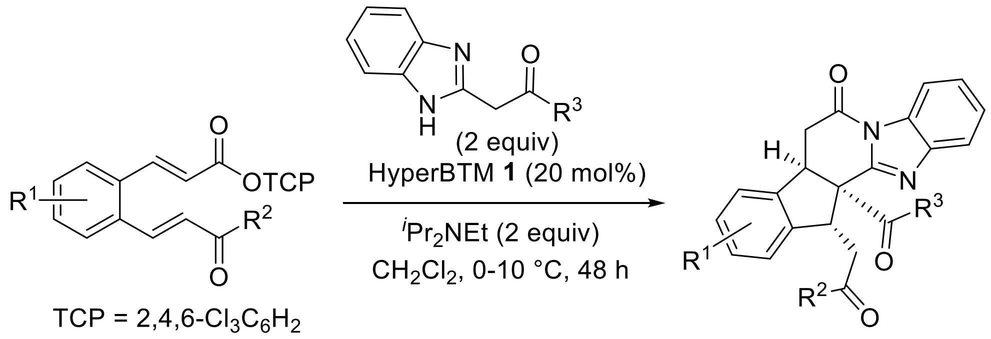

To a solution of the corresponding tricholorophenol-activated ester (1 equiv) in anhydrous CH_2Cl_2 (0.4 M), was added benzimidazole (2.0 equiv), isothiourea HyperBTM **1** (20 mol%) followed by addition of $^{i}Pr_2NEt$ (2.0 equiv) at 0 °C. The reaction mixture was warmed to 10 °C and stirred at this temperature for 48 h. The reaction mixture was quenched with 0.1 M HCl and extracted with CH_2Cl_2. Combined organic layers were washed with brine, dried over anhydrous $MgSO_4$, filtered and concentrated *in vacuo*. The residue was purified by column chromatography using mixtures of petrolether and ethylacetate as eluents giving the products of approximatly 95% purity. Analytically pure compounds can be obtained after a second purification by column chromatography using dichloromethane as eluent.

3 Preparation of Starting Materials

3.1 Data for *tert*-butyl acrylates

tert-Butyl (E)-3-(2-formylphenyl)acrylate (S1)

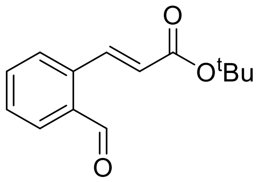

Following General Procedure 1, 2-bromobenzaldehyde (2.9 ml, 25 mmol), Pd(OAc)$_2$ (110 mg, 0.5 mmol), tri-*o*-tolylphosphine (305 mg, 1.0 mmol), (*E*)-*tert*butylacrylate (5.5 ml, 37.5 mmol) and triethylamine (10 ml, 71.7 mmol) in 50 mL PhMe and subsequent chromatography (90:10 hexane : EtOAc, R$_f$ 0.2) afforded the title compound (5.3 g, 92%) as a brown oil. Data in agreement with the literature.[1] ^{1}H NMR (500 MHz, CDCl$_3$) δ$_H$: 1.53 (9H, s, C(C*H*$_3$)$_3$), 6.30 (1H, d, *J* 15.8, CH=C*H*–COO^{t}Bu), 7.52 (1H, td, *J* 7.4, 1.7, C*H*-Ar), 7.59 (2H, qd, *J* 7.8, 3.7, C*H*-Ar), 7.86 (1H, dd, *J* 7.6, 1.0, C*H*-Ar), 8.40 (1H, d, *J* 15.9, C*H*=CH–COO^{t}Bu), 10.30 (1H, s, CO*H*).

tert-Butyl (E)-3-(4-chloro-2-formylphenyl)acrylate (S2)

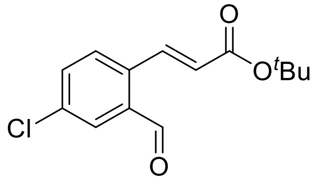

Following General Procedure 1, 5-chloro-2-bromobenzaldehyde (1.1 ml, 5 mmol), Pd(OAc)$_2$ (22 mg, 0.1 mmol), tri-*o*-tolylphosphine (61 mg, 0.2 mmol), (*E*)-*tert*butylacrylate (1.1 ml, 7.5 mmol) and triethylamine (2.0 ml, 14.3 mmol) in 10 mL PhMe and subsequent chromatography (90:10 hexane:EtOAc, R$_f$ 0.2) afforded the title compound (908 mg, 68%) as a yellow solid. mp 55–57 °C; ν$_{max}$ (film) 3003 (alkenyl C-H), 2978 (alkyl C-H), 1699 (C=O); 1636 (C=O); ^{1}H NMR (500 MHz, CDCl$_3$) δ$_H$: 1.54 (9H, s, C(C*H*$_3$)$_3$), 6.30 (1H, d, *J* 15.8, CH=C*H*–COO^{t}Bu), 7.53–7.61 (2H, m, C*H*-Ar), 7.85 (1H, dd, *J* 7.6, 1.0, C*H*-Ar), 8.31 (1H, d, *J* 15.8, C*H*=CH–COO^{t}Bu), 10.30 (1H, s, CO*H*); ^{13}C{^{1}H} NMR (126 MHz, CDCl$_3$) δ$_C$: 28.3 (C(*C*H$_3$)$_3$), 81.4 (*C*(CH$_3$)$_3$), 126.0 (CH=*C*H–COO*t*Bu), 129.5 (*C*H-Ar), 131.1 (*C*H-Ar), 134.0 (*C*-Ar), 134.9 (*C*-Cl), 135.5 (*C*-Ar), 136.2 (*C*-Ar), 138.2 (*C*H=CH–COO^{t}Bu), 165.4 (*C*OO^{t}Bu), 190.2 (*C*OH); *m/z* (NSI$^+$) 289 ([M+Na]$^+$, 100%); HRMS (NSI$^+$) $C_{14}H_{15}ClO_3Na$ [M+Na]$^+$, found 289.0604, requires 289.0602 (+0.7 ppm).

tert-Butyl (_E_)-3-(2-formyl-5-methylphenyl)acrylate (S3)

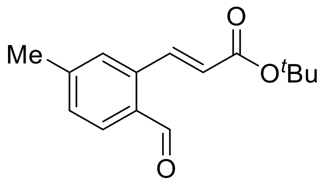

Following General Procedure 1, 4-methyl-2-bromobenzaldehyde (995 mg, 5 mmol), $Pd(OAc)_2$ (22 mg, 0.1 mmol), tri-*o*-tolylphosphine (61 mg, 0.2 mmol), (*E*)-*tert*butylacrylate (1.5 eq) and triethylamine (2.0 ml, 14.3 mmol) in 10 mL PhMe and subsequent chromatography (90:10 hexane : EtOAc, R_f 0.2) afforded the title compound (950 mg, 77%) as a yellow oil. ν_{max} (film) 3001 (alkenyl C-H), 2978 (alkyl C-H), 1697 (C=O), 1634 (C=O); ^{1}H NMR (500 MHz, $CDCl_3$) δ_H: 1.54 (9H, s, C(C*H*$_3$)$_3$), 2.43 (3H, s, Ar-C*H*$_3$), 6.30 (1H, d, *J* 15.8, CH=C*H*–COO^{t}Bu), 7.33 (1H, d, *J* 7.8, C*H*-Ar), 7.42 (1H, s, C*H*-Ar), 7.77 (1H, d, *J* 7.8, C*H*-Ar), 8.40 (1H, d, *J* 15.8, C*H*=CH–COO^{t}Bu), 10.26 (1H, s, CO*H*); ^{13}C{^{1}H} NMR (126 MHz, $CDCl_3$) δ_C: 21.9 (Ar-*C*H$_3$) 28.3 (C(*C*H$_3$)$_3$), 81.0 (*C*(CH$_3$)$_3$), 125.1 (CH=*C*H–COO^{t}Bu), 128.7 (*C*H-Ar), 130.6 (*C*H-Ar), 131.7 (*C*-Ar), 137.1 (*C*-Ar), 140.0 (*C*H=CH–COO^{t}Bu), 145.0 (*C*-Me), 165.7 (*C*OO^{t}Bu), 191.5 (*C*OH); *m/z* (NSI$^+$) 515 ([2M+Na]$^+$, 100%); HRMS (NSI$^+$) $C_{15}H_{18}O_3Na$ [M+Na]$^+$, found 269.1147, requires 269.1148 (–0.4 ppm).

tert-Butyl (_E_)-3-(4-fluoro-2-formylphenyl)acrylate (S4)

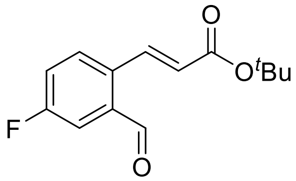

Following General Procedure 1, 5-fluoro-2-bromobenzaldehyde (1.0 g, 5 mmol), $Pd(OAc)_2$ (22 mg, 0.1 mmol), tri-*o*-tolylphosphine (61 mg, 0.2 mmol), (*E*)-*tert*butylacrylate (1.5 eq) and triethylamine (2.0 ml, 14.3 mmol) in 10 mL PhMe and subsequent chromatography (90:10 hexane : EtOAc, R_f 0.2) afforded the title compound (878 mg, 70%) as a yellow oil. ν_{max} (film) 2980 (alkyl C-H), 1705 (C=O), 1605 (C=O); ^{1}H NMR (500 MHz, $CDCl_3$) δ_H: 1.54 (9H, s, C(C*H*$_3$)$_3$), 6.28 (1H, d, *J* 15.8, CH=C*H*–COO^{t}Bu), 7.31 (1H, td, *J* 8.2, 2.8, C*H*-Ar), 7.58 (1H, dd, *J* 8.6, 2.8, C*H*-Ar), 7.63 (1H, dd, *J* 8.6, 5.1, C*H*-Ar), 8.30 (1H, d, *J* 15.8, C*H*=CH–COO^{t}Bu), 10.32 (1H, s, CO*H*); ^{19}F NMR (282 MHz, $CDCl_3$) δ_F: –109.7; ^{13}C{^{1}H} NMR (126 MHz, $CDCl_3$) δ_C: 28.3 (C(*C*H$_3$)$_3$), 81.3 (*C*(CH$_3$)$_3$), 117.1 (d, $^2J_{CF}$ = 22.3, C_{Ar}-F), 117.2 (*C*H-Ar), 121.3 (CH=*C*H–COO^{t}Bu), 121.4 (d, $^2J_{CF}$ = 22.0, C_{Ar}-F), 125.7 (*C*H-Ar), 130.3 (*C*H-Ar), 133.5 (2×*C*-Ar), 138.1 (*C*H=CH–COO^{t}Bu), 163.4 (d, $^1J_{CF}$ = 252.8, C_{Ar}-F), 164.4 (*C*-F), 165.4 (*C*OO^{t}Bu), 190.0 (*C*OH); *m/z*

(NSI$^+$) 273 ([M+Na]$^+$, 85%, 523 ([2M+Na]$^+$, 60%); HRMS (NSI$^+$) $C_{14}H_{15}F_1O_3Na$ [M+Na]$^+$, found 273.0903, requires 273.0897 (+2.0 ppm).

3.2 Data for phosphoranes

1-Phenyl-2-(triphenylphospharanylidene)ethanone (S5)

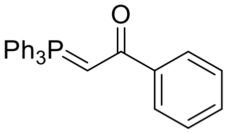

Following General Procedure 2, 2-bromo-1-phenylethanone (11.1 g, 55.5 mmol), triphenyphosphine (14.6 g, 55.5 mmol), anhydrous THF (250 mL) gave the title compound as a white solid (18.7 g, 88%). mp 177–179 °C; {Lit.[2] mp 178–180 °C}; ^{1}H NMR (500 MHz, $CDCl_3$) δ_H: 4.43 (1H, d, *J* 24.6, C*H*), 7.34–7.37 (3H, m, Ar*H*), 7.45–7.58 (9H, m, Ar*H*), 7.70–7.75 (6H, m, Ar*H*), 7.96–7.99 (2H, m, Ar*H*). Data in agreement with the literature.[2]

1-(4-Trifluoromethyl)phenyl)-2-(triphenylphospharanylidene)ethanone (S6)

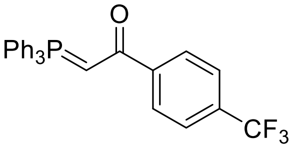

Following General Procedure 2, 2-bromo-1-(4-trifluoromethyl)phenyl)ethanone (7.5 g, 28.1 mmol), triphenyphosphine (7.4 g, 28.1 mmol), anhydrous THF (100 mL) gave the title compound as a yellow solid (12 g, 95%). mp 156–158 °C; {Lit.[1] mp 158–160 °C}; ^{1}H NMR (500 MHz, $CDCl_3$) δ_H: 4.46 (1H, d, *J* 24.5, C*H*), 7.47–7.51 (6H, m, Ar*H*), 7.57–7.61 (5H, m, Ar*H*), 7.69–7.73 (6H, m, Ar*H*), 8.04–8.06 (2H, m, Ar_{CF3}, *H*–2,6). Data in agreement with the literature.[3]

1-(4-Methoxyphenyl)-2-(triphenylphospharanylidene)ethanone (S7)

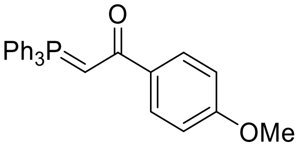

Following General Procedure 2: 2-bromo-1-(4-methoxyphenyl)ethanone (15.0 g, 65.5 mmol), triphenyphosphine (17.2 g, 65.5 mmol), anhydrous THF (200 mL) gave the title compound as a white solid (23 g, 94%). mp 149–151 °C; {Lit.[1] mp 150–153 °C}; ^{1}H NMR (500 MHz, $CDCl_3$) δ_H: 4.36 (1H,

br.s, C*H*), 6.86–6.89 (2H, m, Ar*H*), 7.44–7.48 (6H, m, Ar*H*), 7.53–7.56 (3H, m, Ar*H*), 7.70–7.74 (6H, m, Ar*H*), 7.93–7.95 (2H, m, Ar*H*). Data in agreement with the literature.[4]

3.3 Data for enone *tert*-butyl esters

***tert*-Butyl (*E*)-3-(2-((*E*)-3-oxobut-1-en-1-yl)phenyl)acrylate (S8)**

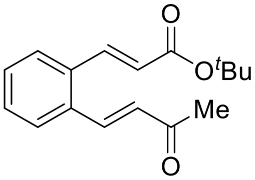

Following General Procedure 3, to a solution of (*E*)-*tert*-butyl 3-(2-formylphenyl)acrylate (1.36 g, 5.88 mmol) in $CHCl_3$, was added a phosphorane, 1-(triphenylphosphanylidene)-2-propanone, (3.80 g, 11.8 mmol, 2.0 equiv). The reaction mixture was refluxed overnight under Ar and the concentrated *in vacuo*. The residue was purified by column chromatography (90:10 Hexane : EtOAc, R_f 0.25) to give the title compound (1.52 g, 95%) as a brown oil. ν_{max} (film) 3087 (alkenyl C-H), 2976 (alkyl C-H), 1705 (C=O); 1672 (C=O); ^{1}H NMR (500 MHz, $CDCl_3$) δ_H: 1.54 (9H, s, C(C*H*$_3$)$_3$), 2.40 (3H, s, COC*H*$_3$), 6.29 (1H, d, *J* 15.8, CH=C*H*– CO$_2$^{t}Bu), 6.60 (1H, d, *J* 15.5, CH=C*H*-COMe), 7.37–7.40 (2H, m, C*H*-Ar), 7.55–7.59 (2H, m, C*H*-Ar), 7.87 (1H, d, *J* 15.5, C*H*=CH–COMe), 7.94 (1H, d, *J* 15.8, C*H*=CH–CO$_2$^{t}Bu); ^{13}C{^{1}H} NMR (126 MHz, $CDCl_3$) δ_C: 27.7 (CO*C*H$_3$), 28.3 (C(*C*H$_3$)$_3$), 81.0 (*C*(CH$_3$)$_3$), 124.0 (CH=*C*H–CO$_2$*t*Bu), 127.7 (*C*H-Ar), 127.8 (*C*H-Ar), 130.0 (*C*H-Ar), 130.3 (*C*H-Ar), 130.5 (CH=*C*H-CO-Me), 134.3 (*C*-Ar), 134.8 (*C*-Ar), 140.2 (*C*H=CH-CO-Me), 140.2 (*C*H=CH–COO^{t}Bu), 165.9 (*C*O$_2$^{t}Bu), 198.2 (*C*OC-Me); *m/z* (NSI$^+$) 567 ([2M+Na]$^+$, 100%), 290 ([M+NH$_4$]$^+$, 45%); HRMS (NSI$^+$) $C_{17}H_{24}O_3N_1$ [M+NH$_4$]$^+$, found 290.1755, requires 290.1751 (+1.5 ppm).

***tert*-butyl (*E*)-3-(2-((*E*)-4,4-dimethyl-3-oxopent-1-en-1-yl)phenyl)acrylate (S9)**

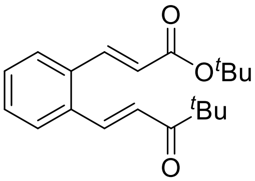

Following General Procedure 3, to a solution of (*E*)-*tert*-butyl 3-(2-formylphenyl)acrylate (0.43 g, 1.86 mmol) in $CHCl_3$, was added a phosphorane, 1-(triphenylphospharanylidene)-3,3-dimethyl-2-butanone, (1.00 g, 2.79 mmol, 1.5 equiv). The reaction mixture was refluxed overnight under Ar and the concentrated *in vacuo*. The residue was purified by column chromatography (90:10 Hexane : EtOAc, R_f 0.3) to give the title compound (0.43 g, 74%) as a yellow solid. mp 87–88 °C; ν_{max} (film) 3003 (alkenyl

C-H), 2976 (alkyl C-H), 1703 (C=O); 1680 (C=O); ^{1}H NMR (500 MHz, $CDCl_3$) δ_H: 1.23 (9H, s, COC(C*H*$_3$)$_3$), 1.54 (9H, s, CO_2C(C*H*$_3$)$_3$), 6.27 (1H, d, *J* 15.8, CH=C*H*–$CO_2{}^t$Bu), 6.97 (1H, d, *J* 15.5, CH=C*H*-CO^{t}Bu), 7.37–7.40 (2H, m, C*H*-Ar), 7.52–7.59 (2H, m, C*H*-Ar), 7.94 (1H, d, *J* 15.8, C*H*=CH–$CO_2{}^t$Bu), 7.98 (1H, d, *J* 15.5, C*H*=CH–CO^{t}Bu); ^{13}C{^{1}H} NMR (126 MHz, $CDCl_3$) δ_C: 26.4 (COC(*C*H$_3$)$_3$), 28.3 (CO_2C(*C*H$_3$)$_3$), 43.4 (CO*C*(CH$_3$)$_3$), 80.9 (CO_2*C*(CH$_3$)$_3$), 123.7 (CH=*C*H–$CO_2{}^t$Bu), 125.1 (CH=*C*H-CO^{t}Bu), 127.9 (*C*H-Ar), 128.3 (*C*H-Ar), 129.8 (*C*H-Ar), 129.9 (*C*H-Ar), 135.1 (2×*C*-Ar), 140.0 (*C*H=CH-CO^{t}Bu), 141.0 (*C*H=CH–$CO_2{}^t$Bu), 165.8 (*C*O$_2{}^t$Bu), 203.9 (*C*O^{t}Bu); *m/z* (NSI$^+$) 332 ([M+NH$_4$]$^+$, 100%); HRMS (NSI$^+$) $C_{20}H_{30}O_3N_1$ [M+NH$_4$]$^+$, found 332.2225, requires 332.2220 (+1.4 ppm).

(*E*)-*tert*-Butyl 3-(2-((*E*)-3-oxo-3-phenylprop-1-en-1-yl)phenyl)acrylate (S10)

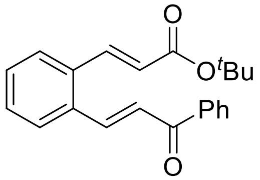

Following General Procedure 3, to a solution of (*E*)-*tert*butyl 3-(2-formylphenyl)acrylate (6.43 g, 27.7 mmol) in $CHCl_3$, was added a phosphorane, 1-phenyl-2-(triphenylphosphoranylidene)-ethanone, (15.8 g, 41.5 mmol, 1.5 equiv). The reaction mixture was refluxed overnight under Ar and the concentrated *in vacuo*. The residue was purified by column chromatography (90:10 Hexane : EtOAc, R_f 0.25) to give the title compound (8.20 g, 88%) as a brown oil. ν_{max} (film) 3084 (alkenyl C-H), 2973 (alkyl C-H), 1703 (C=O); 1662 (C=O); ^{1}H NMR (500 MHz, $CDCl_3$) δ_H: 1.54 (9H, s, C(C*H*$_3$)$_3$), 6.30 (1H, d, *J* 15.8, CH=C*H*–COO^{t}Bu), 7.41 (1H, d, *J* 15.5, CH=C*H*-COC-Ph), 7.41–7.42 (2H, m, C*H*-Ar), 7.50–7.53 (2H, m, C*H*-Ar), 7.57–7.62 (1H, m, C*H*-Ph), 7.66–7.69 (1H, m, C*H*-Ph), 7.72–7.69 (1H, m, C*H*-Ph), 8.00 (1H, d, *J* 15.8, C*H*=CH–CO-Ph) 8.02–8.05 (2H, m, C*H*-Ph), 8.13 (1H, d, *J* 15.6, C*H*=CH–COO^{t}Bu); ^{13}C{^{1}H} NMR (126 MHz, $CDCl_3$) δ_C: 28.3 (C(*C*H$_3$)$_3$), 81.0 (*C*(CH$_3$)$_3$), 124.0 (CH=*C*H–COO^{t}Bu), 126.0 (CH=*C*H-CO-Ph), 128.0 (*C*H-Ar), 128.2 (*C*H-Ar), 128.7 (2×*C*H-Ph), 128.8 (2×*C*H-Ph), 129.9 (*C*H-Ar), 130.3 (*C*H-Ar), 133.1 (*C*H-Ph), 134.9 (*C*-Ar), 135.2 (*C*-Ar), 138.1 (*C*-Ph), 140.7 (*C*H=CH-CO-Ph), 141.8 (*C*H=CH–COO^{t}Bu), 165.8 (*C*OO^{t}Bu), 190.2 (*C*OC-Ph); *m/z* (NSI$^+$) 386 ([2M+NH$_4$]$^+$, 100%), 352 ([M+NH$_4$]$^+$, 60%); HRMS (NSI$^+$) $C_{22}H_{26}O_3N_1$ [M+NH$_4$]$^+$, found 352.1911, requires 352.1907 (+1.1 ppm).

tert-Butyl (E)-3-(2-((E)-3-(4-bromophenyl)-3-oxoprop-1-en-1-yl)phenyl)acrylate (S11)

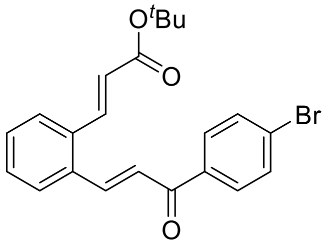

Following General Procedure 3, to a solution of (*E*)-*tert*-butyl 3-(2-formylphenyl)acrylate (0.47 g, 2.0 mmol) in $CHCl_3$, was added a phosphorane, 1-(4-bromo)-phenyl-2-(triphenylphosphora-nylidene)ethanone, (1.8 g, 4.0 mmol, 2.0 equiv). The reaction mixture was refluxed overnight under Ar and the concentrated *in vacuo*. The residue was purified by column chromatography (90:10 Hexane:EtOAc, R_f 0.25) to give the title compound (0.67 g, 80%) as a pale peach solid. mp 116–117 °C; ν_{max} (film) 3061 (alkenyl C-H), 2982 (alkyl C-H), 1697 (C=O); 1607 (C=O); ^{1}H NMR (500 MHz, $CDCl_3$) δ_H: 1.54 (9H, s, C(C*H*$_3$)$_3$), 6.30 (1H, d, *J* 15.8, CH=C*H*–CO$_2{}^t$Bu), 7.35 (1H, d, *J* 15.5, CH=C*H*-COC-Ph$_{Br}$), 7.40–7.45 (2H, m, C*H*-Ar), 7.58–7.60 (1H, m, C*H*-Ar), 7.64–7.67 (2H, m, C*H*-Ph$_{Br}$), 7.66–7.69 (1H, m, C*H*-Ar), 7.89–7.91 (1H, m, C*H*-Ph$_{Br}$), 7.99 (1H, d, *J* 15.8, C*H*=CH–CO$_2{}^t$Bu), 8.13 (1H, d, *J* 15.5, C*H*=CH–CO-Ph$_{Br}$); ^{13}C{^{1}H} NMR (126 MHz, $CDCl_3$) δ_C: 28.3 (C(*C*H$_3$)$_3$), 81.1 (*C*(CH$_3$)$_3$), 124.1 (CH=*C*H–CO$_2{}^t$Bu), 125.4 (CH=*C*H-CO-Ph$_{Br}$), 128.0 (*C*H-Ar), 128.3 (*C*H-Ar), 129.9 (*C*H-Ar), 130.3 (2×*C*H-Ph$_{Br}$), 130.5 (*C*H-Ar), 132.2 (2×*C*H-Ph$_{Br}$), 134.7 (*C*-Ar), 135.3 (*C*-Ar), 136.8 (*C*-Ph$_{Br}$), 140.6 (*C*H=CH-CO-Ph$_{Br}$), 142.4 (*C*H=CH–CO$_2{}^t$Bu), 165.8 (*C*O$_2{}^t$Bu), 189.2 (*C*OC-Ph$_{Br}$); *m/z* (NSI$^+$) 849 ([2M+Na]$^+$, 100%); HRMS (NSI$^+$) $C_{22}H_{21}O_3BrNa$ [M+NH$_4$]$^+$, found 435.0562, requires 435.0566 (–1.0 ppm).

tert-Butyl (E)-3-(2-((E)-3-(4-chlorophenyl)-3-oxoprop-1-en-1-yl)phenyl)acrylate (S12)

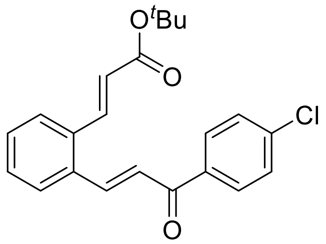

Following General Procedure 3, to a solution of (*E*)-*tert*-butyl 3-(2-formylphenyl)acrylate (0.45 g, 1.9 mmol) in $CHCl_3$, was added a phosphorane, 1-(4-chloro)-phenyl-2-(triphenylphosphornylidene)-ethanone, (1.6 g, 3.8 mmol, 2.0 equiv). The reaction mixture was refluxed overnight under Ar and the concentrated *in vacuo*. The residue was purified by column chromatography (90:10 Hexane : EtOAc, R_f 0.25) to give the title compound (0.68 g, 95%) as a pale yellow solid. mp 118–119 °C; ν_{max} (film) 3113

(alkenyl C-H), 2981 (alkyl C-H), 1697 (C=O); 1665 (C=O); ^{1}H NMR (500 MHz, $CDCl_3$) δ_H: 1.54 (9H, s, C(C*H*$_3$)$_3$), 6.30 (1H, d, *J* 15.8, CH=C*H*–CO$_2$^{t}Bu), 7.36 (1H, d, *J* 15.5, CH=C*H*-COC-Ph$_{Cl}$), 7.40–7.45 (2H, m, C*H*-Ar), 7.46–7.51 (2H, m, C*H*-Ph$_{Cl}$), 7.58–7.60 (1H, m, C*H*-Ar), 7.66–7.68 (1H, m, C*H*-Ar), 7.97–7.99 (1H, m, C*H*-Ph$_{Cl}$), 7.99 (1H, d, *J* 15.8, C*H*=CH–CO-Ph$_{Cl}$), 8.13 (1H, d, *J* 15.5, C*H*=CH–CO$_2$^{t}Bu); ^{13}C{^{1}H} NMR (126 MHz, $CDCl_3$) δ_C: 28.3 (C(*C*H$_3$)$_3$), 81.1 (*C*(CH$_3$)$_3$), 124.1 (CH=*C*H–CO$_2$^{t}Bu), 125.5 (CH=*C*H-CO-Ph$_{Cl}$), 128.0 (*C*H-Ar), 128.3 (*C*H-Ar), 129.2 (2×*C*H-Ph$_{Cl}$), 129.9 (*C*H-Ar), 130.2 (2×*C*H-Ph$_{Cl}$), 130.5 (*C*H-Ar), 134.7 (*C*-Ar), 135.3 (*C*-Ar), 136.4 (*C*-Ph$_{Cl}$), 140.7 (*C*H=CH-CO-Ph$_{Cl}$), 142.3 (*C*H=CH–CO$_2$^{t}Bu), 165.8 (*C*O$_2$^{t}Bu), 188.8 (*C*OC-Ph$_{Cl}$); *m/z* (NSI$^+$) 759 ([2M+Na]$^+$, 100%), 775 ([2M+K]$^+$, 75%), 391 ([M+Na]$^+$, 70%); HRMS (NSI$^+$) $C_{22}H_{21}O_3ClNa$ [M+Na]$^+$, found 391.1074, requires 391.1071 (+0.7 ppm).

***tert*-Butyl (*E*)-3-(2-((*E*)-3-(4-trifluoromethyl)phenyl)-3-oxoprop-1-en-1-yl)phenyl)acrylate (S13)**

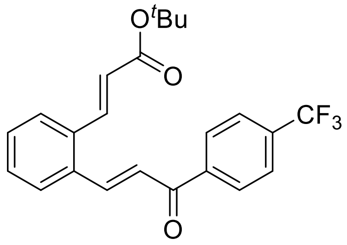

Following General Procedure 3, to a solution of (*E*)-*tert*-butyl 3-(2-formylphenyl)acrylate (0.47 g, 2.1 mmol) in $CHCl_3$, was added a phosphorane, 1-(4-trifluoro)-phenyl-2-(triphenylphosphoranylidene)-ethanone, (0.89 g, 4.0 mmol, 2.0 equiv). The reaction mixture was refluxed overnight under Ar and the concentrated *in vacuo*. The residue was purified by column chromatography (90:10 Hexane : EtOAc, R_f 0.25) to give the title compound (0.76 g, 90%) as a yellow solid. mp 95–97 °C; ν_{max} (film) 3103 (alkenyl C-H), 2982 (alkyl C-H), 1695 (C=O); 1668 (C=O); ^{1}H NMR (500 MHz, $CDCl_3$) δ_H: 1.54 (9H, s, C(C*H*$_3$)$_3$), 6.30 (1H, d, *J* 15.8, CH=C*H*–CO$_2$^{t}Bu), 7.37 (1H, d, *J* 15.6, CH=C*H*-COC-Ph$_{CF3}$), 7.40–7.47 (2H, m, C*H*-Ar), 7.58–7.60 (1H, m, C*H*-Ar), 7.66–7.68 (1H, m, C*H*-Ar), 7.77–7.78 (2H, m, C*H*-Ph$_{CF3}$), 7.99 (1H, d, *J* 15.8, C*H*=CH–CO$_2$^{t}Bu), 8.11–8.13 (2H, m, C*H*-Ph$_{CF3}$) 8.15 (1H, d, *J* 15.6, C*H*=CH–CO-Ph$_{CF3}$); ^{19}F NMR (282 MHz, $CDCl_3$) δ_F: –63.0 (C*F*$_3$); ^{13}C{^{1}H} NMR (126 MHz, $CDCl_3$) δ_C: 28.3 (C(*C*H$_3$)$_3$), 81.1 (*C*(CH$_3$)$_3$), 124.3 (CH=*C*H–CO$_2$^{t}Bu), 125.4 (CH=*C*H-CO-Ph$_{CF3}$), 125.9 (2×*C*H-Ph$_{CF3}$), 125.9 (q, $^3J_{CF}$ = 3.4, C$_{Ar}$-CF$_3$), 127.0 (q, $^1J_{CF}$ = 272.6, C$_{Ar}$-CF$_3$), 128.9 (*C*-Ph$_{CF3}$), 128.1 (*C*H-Ar), 128.3 (*C*H-Ar), 129.0 (2×*C*H-Ph$_{CF3}$), 130.0 (*C*H-Ar), 130.7 (*C*H-Ar), 134.3 (q, $^2J_{CF}$ = 31.2, C$_{Ar}$-CF$_3$), 134.4 (*C*-Ar), 135.4 (*C*-Ar), 140.5 (*C*H=CH-CO-Ph$_{CF3}$), 140.9 (*C*-Ph$_{CF3}$), 143.1 (*C*H=CH–CO$_2$^{t}Bu), 165.8 (*C*O$_2$^{t}Bu), 189.5 (*C*OC-Ph$_{CF3}$); *m/z* (NSI$^+$) 425 ([M+Na]$^+$, 100%), 827 ([2M+Na]$^+$, 45%); HRMS (NSI$^+$) $C_{23}H_{21}O_3F_3Na$ [M+Na]$^+$, found 425.1332, requires 425.1335 (–0.7 ppm).

tert-Butyl (E)-3-(2-((E)-3-(4-methoxy)phenyl)-3-oxoprop-1-en-1-yl)phenyl)acrylate (S14)

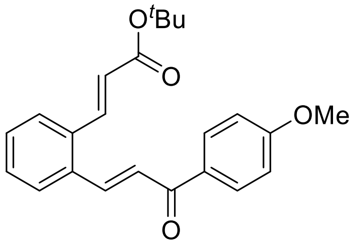

Following General Procedure 3, to a solution of (*E*)-*tert*-butyl 3-(2-formylphenyl)acrylate (0.47 g, 2.0 mmol) in $CHCl_3$, was added a phosphorane, 1-(4-methoxy)-phenyl-2-(triphenylphosphoranylidene)-ethanone, (0.75 g, 4.0 mmol, 2.0 equiv). The reaction mixture was refluxed overnight under Ar and the concentrated *in vacuo*. The residue was purified by column chromatography (90:10 Hexane : EtOAc, R_f 0.20) to give the title compound (0.68 g, 93%) as a yellow solid. mp 84–85 °C; ν_{max} (film) 3057 (alkenyl C-H), 2980 (alkyl C-H), 1690 (C=O); 1661 (C=O); ^{1}H NMR (500 MHz, $CDCl_3$) δ_H: 1.54 (9H, s, C(C*H*$_3$)$_3$), 3.89 (1H, s, OC*H*$_3$), 6.29 (1H, d, *J* 15.8, CH=C*H*–CO$_2$^{t}Bu), 7.39–7.43 (2H, m, C*H*-Ar), 7.41 (1H, d, *J* 15.5, CH=C*H*-COC-Ph$_{OMe}$), 7.56–7.59 (1H, m, C*H*-Ar), 7.66–7.68 (1H, m, C*H*-Ar), 8.00 (1H, d, *J* 15.8, C*H*=CH–CO$_2$^{t}Bu), 8.04–8.05 (2H, m, C*H*-Ph$_{OMe}$), 8.11 (1H, d, *J* 15.5, C*H*=CH–CO-Ph$_{OMe}$), ^{13}C{^{1}H} NMR (126 MHz, $CDCl_3$) δ_C: 28.3 (C(*C*H$_3$)$_3$), 55.7 (O*C*H$_3$), 81.0 (*C*(CH$_3$)$_3$), 114.0 (2×*C*H-Ph$_{OMe}$), 123.9 (CH=*C*H–CO$_2$^{t}Bu), 126.0 (CH=*C*H-CO-Ph$_{OMe}$), 127.9 (*C*H-Ar), 128.2 (*C*H-Ar), 129.8 (*C*H-Ar), 130.1 (*C*H-Ar), 131.0 (*C*-Ph$_{OMe}$), 131.1 (2×*C*H-Ph$_{OMe}$), 135.1 (2×*C*-Ar), 140.9 (*C*H=CH-CO-Ph$_{OMe}$), 141.0 (*C*H=CH–CO$_2$^{t}Bu), 163.7 (Ph*C*-OMe), 165.9 (*C*O$_2$^{t}Bu), 188.4 (*C*OC-Ph$_{OMe}$); *m/z* (NSI$^+$) 751 ([2M+Na]$^+$, 100%), 387 ([2M+K]$^+$, 50%), 387 ([M+Na]$^+$, 40%); HRMS (NSI$^+$) $C_{23}H_{25}O$ [M+H]$^+$, found 365.1750, requires 365.1747 (+0.7 ppm).

tert-Butyl (E)-3-(4-chloro-2-((E)-3-oxobut-1-en-1-yl)phenyl)acrylate (S15)

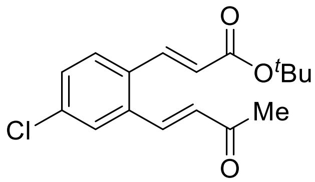

Following General Procedure 3, to a solution of (*E*)-*tert*-butyl 3-(4-chloro-2-formylphenyl)acrylate (828 mg, 3.30 mmol) in $CHCl_3$, was added a phosphorane, 1-(triphenylphosphanylidene)-2-propanone (2.1 g, 6.60 mmol, 2.0 equiv). The reaction mixture was refluxed overnight under Ar and the concentrated *in vacuo*. The residue was purified by column chromatography (90:10 Hexane : EtOAc, R_f 0.25) to give the title compound (841 mg, 83%) as a yellow solid. mp 127–129 °C; ν_{max} (film) 2978 (alkenyl C-H), 2931 (alkyl C-H), 1705 (C=O); 1674 (C=O); ^{1}H NMR (500 MHz, $CDCl_3$) δ_H: 1.54 (9H, s, C(C*H*$_3$)$_3$), 2.41 (3H,

s, COCH_3), 6.28 (1H, d, *J* 15.8, CH=C*H*– $CO_2{}^t$Bu), 6.61 (1H, d, *J* 16.0, CH=C*H*-COMe), 7.36 (1H, dd, *J* 8.4, 2.1, C*H*-Ar), 7.50–7.55 (2H, m, C*H*-Ar), 7.79 (1H, d, *J* 16.0, C*H*=CH–COMe), 7.94 (1H, d, *J* 15.8, C*H*=CH–$CO_2{}^t$Bu); $^{13}C\{^1H\}$ NMR (126 MHz, $CDCl_3$) δ_C: 28.1 (CO*C*H_3), 28.3 (C(*C*H_3)$_3$), 81.3 (*C*(CH_3)$_3$), 124.4 (CH=*C*H–$CO_2{}^t$Bu), 127.6 (*C*H-Ar), 129.1 (*C*H-Ar), 130.3 (*C*H-Ar), 131.2 (CH=*C*H-CO-Me), 133.2 (*C*-Cl), 135.9 (*C*-Ar), 136.0 (*C*-Ar), 138.6 (*C*H=CH-CO-Me), 138.9 (*C*H=CH–COO^{t}Bu), 165.6 (*C*$O_2{}^t$Bu), 197.7 (*C*OC-Me); *m/z* (NSI^+) 635 ($[2M+Na]^+$, 100%), 329 ($[M+Na]^+$, 85%); HRMS (NSI^+) $C_{17}H_{19}ClO_3Na$ $[M+Na]^+$, found 329.0912, requires 329.0915 (–0.9 ppm).

tert-Butyl (_E_)-3-(5-methyl-2-((_E_)-3-oxobut-1-en-1-yl)phenyl)acrylate (S16)

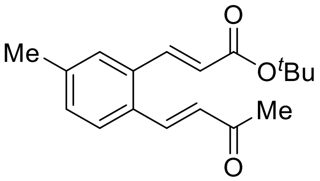

Following General Procedure 3, to a solution of *tert*-butyl (*E*)-3-(2-formyl-5-methylphenyl)acrylate (921 mg, 3.74 mmol) in $CHCl_3$, was added a phosphorane, 1-(triphenylphosphanylidene)-2-propanone (2.4 g, 7.48 mmol, 2.0 equiv). The reaction mixture was refluxed overnight under Ar and the concentrated *in vacuo*. The residue was purified by column chromatography (90:10 Hexane : EtOAc, R_f 0.25) to give the title compound (978 mg, 91%) as a yellow solid. mp 83–85 °C; ν_{max} (film) 2978 (alkenyl C-H), 2930 (alkyl C-H), 1703 (C=O); 1672 (C=O); ^1H NMR (500 MHz, $CDCl_3$) δ_H: 1.54 (9H, s, C(CH_3)$_3$), 2.37 (3H, s, Ar-CH_3), 2.39 (3H, s, COCH_3), 6.28 (1H, d, *J* 15.7, CH=C*H*– $CO_2{}^t$Bu), 6.58 (1H, d, *J* 16.1, CH=C*H*-COMe), 7.20 (1H, d, *J* 7.9, C*H*-Ar), 7.38 (1H, s, C*H*-Ar), 7.49 (1H, d, *J* 7.9, C*H*-Ar), 7.85 (1H, d, *J* 16.1, C*H*=CH–COMe), 7.93 (1H, d, *J* 15.8, C*H*=CH–$CO_2{}^t$Bu); $^{13}C\{^1H\}$ NMR (126 MHz, $CDCl_3$) δ_C: 21.5 (Ar-*C*H_3), 27.6 (CO*C*H_3), 28.3 (C(*C*H_3)$_3$), 81.0 (*C*(CH_3)$_3$), 123.8 (CH=*C*H–$CO_2{}^t$Bu), 127.6 (*C*H-Ar), 128.3 (*C*H-Ar), 129.6 (*C*H-Ar), 131.0 (CH=*C*H-CO-Me), 131.5 (*C*-Ar), 134.7 (*C*-Ar), 140.2 (*C*H=CH-CO-Me), 140.3 (*C*H=CH–COO*t*Bu), 140.7 (*C*-Me), 165.9 (*C*$O_2{}^t$Bu), 198.3 (*C*OCH_3); *m/z* (NSI^+) 595 ($[2M+Na]^+$, 100%), 309 ($[M+Na]^+$, 45%); HRMS (NSI^+) $C_{18}H_{22}O_3Na$ $[M+Na]^+$, found 309.1461, requires 309.1461 (–0.1 ppm).

tert-Butyl (E)-3-(4-fluoro-2-((E)-3-oxobut-1-en-1-yl)phenyl)acrylate (S17)

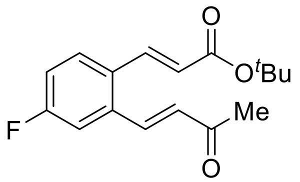

Following General Procedure 3, to a solution of (*E*)-*tert*-butyl 3-(4-fluoro-2-formylphenyl)acrylate (495 mg, 1.98 mmol) in $CHCl_3$, was added a phosphorane, 1-(triphenylphosphanylidene)-2-propanone (1.19 g, 3.75 mmol, 2.0 equiv). The reaction mixture was refluxed overnight under Ar and the concentrated *in vacuo*. The residue was purified by column chromatography (90:10 Hexane : EtOAc, R_f 0.25) to give the title compound (431 mg, 75%) as a yellow solid. mp 89–91 °C; ν_{max} (film) 2980 (alkenyl C-H), 2955 (alkyl C-H), 1705 (C=O); 1674 (C=O); ^{1}H NMR (500 MHz, $CDCl_3$) δ_H: 1.54 (9H, s, C(C*H*$_3$)$_3$), 2.41 (3H, s, COC*H*$_3$), 6.24 (1H, d, *J* 15.8, CH=C*H*–CO$_2$^{t}Bu), 6.59 (1H, d, *J* 16.1, CH=C*H*-COMe), 7.10 (1H, td, *J* 8.3, 2.6, C*H*-Ar), 7.36 (1H, dd, *J* 9.5, 2.6, C*H*-Ar), 7.57 (1H, dd, *J* 8.7, 5.6, C*H*-Ar), 7.81 (1H, d, *J* 16.1, C*H*=CH–COMe), 7.87 (1H, d, *J* 15.8, C*H*=CH–CO$_2$^{t}Bu); ^{19}F NMR (282 MHz, $CDCl_3$) δ_F: –110.3; ^{13}C{^{1}H} NMR (126 MHz, $CDCl_3$) δ_C: 27.9 (CO*C*H$_3$), 28.3 (C(*C*H$_3$)$_3$), 81.2 (*C*(CH$_3$)$_3$), 114.1 (d, $^2J_{CF}$ = 22.5, C_{Ar}-F), 114.2 (*C*H-Ar), 117.7 (d, $^2J_{CF}$ = 22.0, C_{Ar}-F), 117.8 (*C*H-Ar), 123.8 (CH=*C*H–CO$_2$^{t}Bu), 129.9 (d, $^3J_{CF}$ = 8.5, C_{Ar}-F), 129.9 (*C*H-Ar), 131.0 (*C*-Ar), 131.2 (CH=*C*H-CO-Me), 136.4 (*C*-Ar), 138.8 (*C*H=CH-CO-Me), 139.0 (*C*H=CH–COO^{t}Bu), 164.6 (*C*-F), 165.8 (*C*O$_2$*t*Bu), 197.8 (*C*OCH$_3$); *m/z* (NSI$^+$) 603 ([2M+Na]$^+$, 100%), 313 ([M+Na]$^+$, 45%); HRMS (NSI$^+$) $C_{17}H_{19}F_1O_3Na$ [M+Na]$^+$, found 313.1210, requires 313.1210 (–0.1 ppm).

3.4 Data for enone acids

(E)-3-(2-((E)-3-Oxobut-1-en-1-yl)phenyl)acrylic acid (S18)

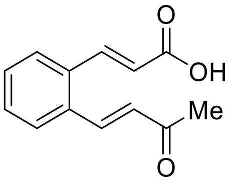

Following General Procedure 4, *tert*-Butyl (*E*)-3-(2-((*E*)-3-oxobut-1-en-1-yl)phenyl)acrylate (1.52 g, 5.59 mmol) gave the corresponding carboxylic acid (1.16 g, 96%) as light green solid. mp 144–146 °C; ν_{max} (film) 3125–2485 br (carb. acid, O-H), 1672 (C=O), 1593 (C=O); ^{1}H NMR (500 MHz, $CDCl_3$) δ_H: 2.43 (3H, s, COC*H*$_3$), 6.39 (1H, d, *J* 15.8, CH=C*H*–COOH), 6.64 (1H, d, *J* 16.1, CH=C*H*-COMe), 7.42–7.46 (2H, m, C*H*-Ar), 7.53–7.71 (2H, m, C*H*-Ar), 7.88 (1H, d, *J* 16.1, C*H*=CH–CO-Me), 8.15 (1H, d, *J* 15.8, C*H*=CH–COOH); ^{13}C{^{1}H} NMR (126 MHz, $CDCl_3$) δ_C: 28.0 (CO*C*H$_3$), 121.0 (CH=*C*H–COOH), 127.9

(*C*H-Ar), 128.0 (*C*H-Ar), 130.5 (CH=*C*H–COMe), 130.7 (*C*H-Ar), 130.8 (*C*H-Ar), 134.1 (*C*-Ar), 134.7 (*C*-Ar), 139.9 (*C*H=CH-CO-Me), 143.7 (*C*H=CH–COOH), 171.5 (*C*OOH), 198.3 (*C*O-Me); *m/z* (NSI^+) 215 ($[M–H]^-$, 100%), 431 ($[2M–H]^-$, 40%); HRMS (NSI^+) $C_{18}H_{15}O_3[M+H]^+$, found 279.1016, requires 279.1016 (+0.1 ppm).

(*E*)-3-(2-((*E*)-4,4-Dimethyl-3-oxopent-1-en-1-yl)phenyl)acrylic acid (S19)

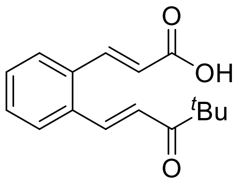

Following General Procedure 4, *tert*-butyl (*E*)-3-(2-((*E*)-4,4-dimethyl-3-oxopent-1-en-1-yl)phenyl)-acrylate (0.43 g, 1.36 mmol) gave the corresponding carboxylic acid (0.36 g, quant.) as a light green solid. mp 147–148 °C; ν_{max} (film) 2976–2517 br (carb. acid, O-H), 1678 (C=O), 1624 (C=O); ^{1}H NMR (500 MHz, $CDCl_3$) δ_H: 1.24 (9H, s, COC(C*H*$_3$)$_3$), 6.39 (1H, d, *J* 15.8, CH=C*H*–COOH), 6.98 (1H, d, *J* 15.5, CH=C*H*-CO*t*Bu), 7.38–7.48 (2H, m, C*H*-Ar), 7.55–7.70 (2H, m, C*H*-Ar), 8.00 (1H, d, *J* 15.5, C*H*=CH–CO^{t}Bu), 8.17 (1H, d, *J* 15.8, C*H*=CH–COOH); ^{13}C{^{1}H} NMR (126 MHz, $CDCl_3$) δ_C: 26.2 (COC(*C*H$_3$)$_3$), 31.0 (CO*C*(CH$_3$)$_3$), 120.2 (CH=*C*H–COOH), 125.4 (CH=*C*H–CO^{t}Bu), 127.8 (*C*H-Ar), 128.4 (*C*H-Ar), 129.9 (*C*H-Ar), 130.4 (*C*H-Ar), 134.1 (*C*-Ar), 135.4 (*C*-Ar), 139.6 (*C*H=CH-CO^{t}Bu), 144.1 (*C*H=CH–COOH), 170.0 (*C*OOH), 192.3 (*C*O^{t}Bu); *m/z* (NSI^+) 539 ($[2M+Na]^+$, 100%), 281 ($[M+Na]^+$, 40%); HRMS (NSI^+) $C_{16}H_{19}O_3[M+H]^+$, found 259.1329, requires 259.1331 (+0.9 ppm).

(*E*)-3-(2-((*E*)-3-Oxo-3-phenylprop-1-en-1-yl)phenyl)acrylic acid (S20)

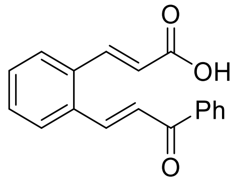

Following General Procedure 4, (*E*)-*tert*-butyl 3-(2-((*E*)-3-oxo-3-phenylprop-1-en-1-yl)phenyl)acrylate (7.09 g, 21.2mmol) gave the corresponding carboxylic acid (5.58 g, 95%) as a yellow solid. mp 155–158 °C; ν_{max} (film) 3113–2523 br (carb. acid, O-H), 1678 (C=O), 1589 (C=O); ^{1}H NMR (500 MHz,$CDCl_3$) δ_H: 6.42 (1H, d, *J* 15.8, CH=C*H*–COOH), 7.42 (1H, d, *J* 15.5, CH=C*H*-CO-Ph), 7.45–7.48 (2H, m, C*H*-Ar), 7.52 (2H, t, *J* 7.6, C*H*-Ar), 7.60 (1H, dd, *J* 10.5, 4.2, C*H*-Ph), 7.64–7.66 (1H, m, C*H*-Ph), 7.69–7.72 (1H, m, C*H*-Ph), 8.04–8.06 (2H, m, C*H*-Ph), 8.15 (1H, d, *J* 15.6, C*H*=CH–COOH), 8.24 (1H, d, *J* 15.8, C*H*=CH–CO-Ph); ^{13}C{^{1}H} NMR (126 MHz, $CDCl_3$)δ_C: 120.9 (CH=*C*H–COOH), 126.5 (CH=*C*H-CO-

Ph), 128.1 (*C*H-Ar), 128.4 (*C*H-Ar), 128.8 (2×*C*H-Ph), 128.9 (2×*C*H-Ph), 130.4 (*C*H-Ar), 130.6 (*C*H-Ar), 133.3 (*C*H-Ph), 134.4 (*C*-Ar), 135.3 (*C*-Ar), 137.9 (*C*-Ph), 141.5 (*C*H=CH-COC-Ph), 144.1 (*C*H=CH–COOH), 171.5 (*C*OOH), 190.3 (*C*OC-Ph); *m/z* (NSI^+) 279 ($[M+H]^+$, 50%); HRMS (NSI^+) $C_{18}H_{15}O_3[M+H]^+$, found 279.1016, requires 279.1016 (+0.1 ppm).

(*E*)-3-(2-((*E*)-3-(4-Bromophenyl)-3-oxoprop-1-en-1-yl)phenyl)acrylic acid (S21)

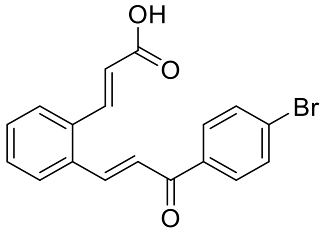

Following General Procedure 4, (*E*)-*tert*-butyl 3-(2-((*E*)-3-oxo-3-(4-bromophenyl)prop-1-en-1-yl)phenyl)acrylate (0.66 g, 1.59 mmol) gave the corresponding carboxylic acid (0.53 g, 93%) as a yellow solid. mp 158–160 °C; ν_{max} (film) 3065–2579 br (carb. acid, O-H), 1682 (C=O), 1584 (C=O); ^{1}H NMR (500 MHz,$CDCl_3$) δ_H: 6.42 (1H, d, *J* 15.8, CH=C*H*–COOH), 7.37 (1H, d, *J* 15.5, CH=C*H*-CO-Ph_{Br}), 7.45–7.49 (2H, m, C*H*-Ar), 7.65–7.67 (3H, m, 2×C*H*-Ph_{Br} + C*H*Ar), 7.69–7.70 (1H, m, C*H*-Ar), 7.91–7.92 (2H, m, C*H*-Ph_{Br}), 8.15 (1H, d, *J* 15.6, C*H*=CH–CO-Ph_{Br}), 8.22 (1H, d, *J* 15.8, C*H*=CH–COOH); ^{13}C{^{1}H} NMR (126 MHz, $CDCl_3$) δ_C: 120.8 (CH=*C*H–COOH), 125.8 (CH=*C*H-CO-Ph_{Br}), 128.1 (*C*H-Ar), 128.4 (*C*-Ph_{Br}), 128.5 (*C*H-Ar), 130.3 (2×*C*H-Ph_{Br}), 130.5 (*C*H-Ar), 130.6 (*C*H-Ar), 132.2 (2×*C*H-Ph_{Br}), 134.4 (*C*-Ar), 135.1 (*C*-Ar), 136.6 (*C*-Ph_{Br}), 142.0 (*C*H=CH-COC-Ph_{Br}), 144.1 (*C*H=CH–COOH), 170.8 (*C*OOH), 189.0 (*C*OC-Ph_{Br}); *m/z* (NSI^+) 737 ($[2M+Na]^+$, 100%), 715 ($[2M+H]^+$, 15%); HRMS (NSI^+) $C_{18}H_{13}O_3BrNa$ $[M+Na]^+$, found 378.9941, requires 378.9940 (+0.2 ppm).

(*E*)-3-(2-((*E*)-3-(4-Chlorophenyl)-3-oxoprop-1-en-1-yl)phenyl)acrylic acid (S22)

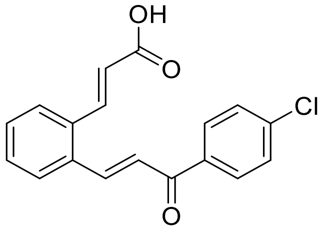

Following General Procedure 4, (*E*)-*tert*-butyl 3-(2-((*E*)-3-oxo-3-(4-chlorophenyl)prop-1-en-1-yl)phenyl)acrylate (0.65 g, 1.78 mmol) gave the corresponding carboxylic acid (0.55 g, quant.) as a green solid. mp 178–181 °C; ν_{max} (film) 3067–2570 br (carb. acid, O-H), 1684 (C=O), 1654 (C=O); ^{1}H NMR (500 MHz,$CDCl_3$) δ_H: 6.42 (1H, d, *J* 15.8, CH=C*H*–COOH), 7.38 (1H, d, *J* 15.5, CH=C*H*-CO-Ph_{Cl}), 7.43–7.52 (4H, m, 2×C*H*-Ar + 2×C*H*-Ph_{Cl}), 7.64–7.66 (1H, m, C*H*Ar), 7.69–7.70 (1H, m, C*H*-Ar), 7.98–

8.00 (2H, m, C*H*-Ph$_{Cl}$), 8.15 (1H, d, *J* 15.6, C*H*=CH–CO-Ph$_{Cl}$), 8.22 (1H, d, *J* 15.8, C*H*=CH–COOH); ^{13}C{^{1}H} NMR (126 MHz, $CDCl_3$) δ_C: 120.7 (CH=*C*H–COOH), 125.9 (CH=*C*H-CO-Ph$_{Cl}$), 128.1 (*C*H-Ar), 128.5 (*C*H-Ar), 129.2 (2×*C*H-Ph$_{OMe}$), 130.2 (2×*C*H-Ph$_{Cl}$), 130.5 (*C*H-Ar), 130.6 (*C*H-Ar), 134.5 (*C*-Ar), 135.1 (*C*-Ar), 136.3 (*C*-Ph$_{Cl}$), 139.7 (*C*-Ph$_{Cl}$), 142.0 (*C*H=CH-COC-Ph$_{Cl}$), 144.0 (*C*H=CH–COOH), 170.5 (*C*OOH), 188.8 (*C*OC-Ph$_{Cl}$); *m/z* (NSI$^+$) 647 ([2M+Na]$^+$, 100%); HRMS (NSI$^+$) $C_{18}H_{13}O_3ClNa[M+Na]^+$, found 335.0450, requires 335.0445 (+1.4 ppm).

(*E*)-3-(2-((*E*)-3-(4-Trifluoromethyl)phenyl)-3-oxoprop-1-en-1-yl)phenyl)acrylic acid (S23)

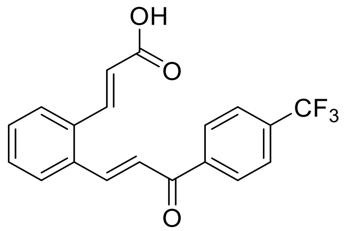

Following General Procedure 4, (*E*)-*tert*-butyl 3-(2-((*E*)-3-oxo-3-(4-trifluoromethyl)phenyl)prop-1-en-1-yl)phenyl)acrylate (0.76 g, 1.88 mmol) gave the corresponding carboxylic acid (0.66 g, quant) as a yellow solid. mp 118–120 °C; ν_{max} (film) 3065–2365 br (carb. acid, O-H), 1682 (C=O), 1659 (C=O); ^{1}H NMR (500 MHz,$CDCl_3$) δ_H: 6.42 (1H, d, *J* 15.8, CH=C*H*–COOH), 7.39 (1H, d, *J* 15.5, CH=C*H*-CO-Ph$_{CF3}$), 7.46–7.52 (2H, m, C*H*-Ar), 7.65–7.67 (1H, m, C*H*Ar), 7.70–7.72 (1H, m, C*H*-Ar), 7.78–7.79 (2H, m, C*H*-Ph$_{CF3}$), 8.13–8.15 (2H, m, C*H*-Ph$_{CF3}$), 8.18 (1H, d, *J* 15.5, C*H*=CH–CO-Ph$_{CF3}$), 8.22 (1H, d, *J* 15.8, C*H*=CH–COOH); ^{19}F NMR (282 MHz, $CDCl_3$) δ_F: –63.1 (C*F*$_3$); ^{13}C{^{1}H} NMR (126 MHz, $CDCl_3$) δ_C: 120.9 (CH=*C*H–COOH), 125.8 (CH=*C*H-CO-Ph$_{CF3}$), 125.9 (q, $^{3}J_{CF}$ = 3.6, C_{Ar}-CF_3), 126.0 (2×*C*H-Ph$_{CF3}$), 127.1 (q, $^{1}J_{CF}$ = 279.5, C_{Ar}-CF_3), 128.2 (*C*H-Ar), 128.5 (*C*H-Ar), 129.0 (2×*C*H-Ph$_{CF3}$), 130.2 (2×*C*H-Ph$_{Cl}$), 130.6 (*C*H-Ar), 130.7 (*C*H-Ar), 134.5 (2×*C*-Ar), 134.7 (q, $^{2}J_{CF}$ = 45.0, C_{Ar}-CF_3), 134.9 (2×*C*-Ph$_{CF3}$), 142.7 (*C*H=CH-COC-Ph$_{CF3}$), 144.0 (*C*H=CH–COOH), 170.5 (*C*OOH), 189.2 (*C*OC-Ph$_{CF3}$); *m/z* (NSI$^+$) 691 ([2M–H]$^-$, 100%); HRMS (NSI$^+$) $C_{19}H_{12}O_3F_3$ [M–H]$^-$, found 345.0740, requires 345.0733 (+2.0 ppm).

(*E*)-3-(2-((*E*)-3-(4-Methoxyphenyl)-3-oxoprop-1-en-1-yl)phenyl)acrylic acid (S24)

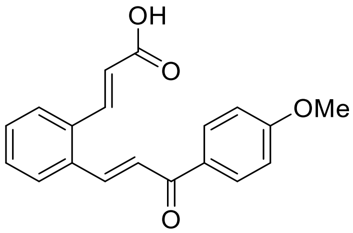

Following General Procedure 4, (*E*)-*tert*-butyl 3-(2-((*E*)-3-oxo-3-(4-methoxyphenyl)prop-1-en-1-yl)phenyl)acrylate (0.68 g, 1.85 mmol) gave the corresponding carboxylic acid (0.57 g, quant) as a yellow solid. mp 194–195 °C; ν_{max} (film) 3063–2515 br (carb. acid, O-H), 1682 (C=O), 1607 (C=O); ^{1}H NMR (500 MHz,$CDCl_3$) δ_H: 3.89 (3H, s, OC*H*$_3$), 6.41 (1H, d, *J* 15.8, CH=C*H*–COOH), 6.99 (2H, m, C*H*-Ph$_{OMe}$), 7.42 (1H, d, *J* 15.5, CH=C*H*-CO-Ph$_{OMe}$), 7.44–7.48 (2H, m, C*H*-Ar), 7.61–7.66 (1H, m, C*H*Ar), 7.68–7.72 (1H, m, C*H*-Ar), 8.05–8.07 (2H, m, C*H*-Ph$_{OMe}$), 8.12 (1H, d, *J* 15.5, C*H*=CH–CO-Ph$_{OMe}$), 8.24 (1H, d, *J* 15.8, C*H*=CH–COOH); ^{13}C{^{1}H} NMR (126 MHz, $CDCl_3$) δ_C: 55.7 (O*C*H$_3$), 114.1 (2×*C*H-Ph$_{OMe}$), 120.5 (CH=*C*H–COOH), 126.5 (CH=*C*H-CO-Ph$_{OMe}$), 128.0 (*C*H-Ar), 128.4 (*C*H-Ar), 130.2 (*C*H-Ar), 130.6 (*C*H-Ar), 130.9 (*C*-Ph$_{OMe}$), 131.2 (2×*C*H-Ph$_{OMe}$), 134.3 (*C*-Ar), 135.6 (*C*-Ar), 140.7 (*C*H=CH-COC-Ph$_{OMe}$), 144.2 (*C*H=CH–COOH), 163.8 (*C*-Ph$_{OMe}$), 170.5 (*C*OOH), 188.5 (*C*OC-Ph$_{OMe}$); *m/z* (NSI$^+$) 639 ([2M+Na]$^+$, 100%), 331 ([M+Na]$^+$, 50%); HRMS (NSI$^+$) $C_{19}H_{17}O_4$ [M+H]$^+$, found 309.1125, requires 309.1121 (+1.2 ppm).

(*E*)-3-(4-Chloro-2-((*E*)-3-Oxobut-1-en-1-yl)phenyl)acrylic (S25)

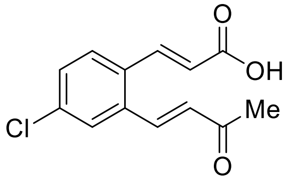

Following General Procedure 4, *tert*-Butyl (*E*)-3-(4-chloro-2-((*E*)-3-oxobut-1-en-1-yl)phenyl)acrylate (827 mg, 2.70 mmol) gave the corresponding carboxylic acid (676 mg, quant.) as a yellow solid. mp 131–133 °C; ν_{max} (film) 3068–2569 br (carb. acid, O-H), 1697 (C=O), 1622 (C=O); ^{1}H NMR (500 MHz, $CDCl_3$) δ_H: 2.42 (3H, s, COC*H*$_3$), 6.38 (1H, d, *J* 15.8, CH=C*H*–COOH), 6.64 (1H, d, *J* 16.0, CH=C*H*-COMe), 7.41 (1H, dd, *J* 8.4, 2.1, C*H*-Ar), 7.56–7.58 (2H, m, C*H*-Ar), 7.80 (1H, d, *J* 16.0, C*H*=CH–CO-Me), 8.06 (1H, d, *J* 15.8, C*H*=CH–COOH); ^{13}C{^{1}H} NMR (126 MHz, $CDCl_3$) δ_C: 28.3 (CO*C*H$_3$), 121.0 (CH=*C*H–COOH), 127.7 (*C*H-Ar), 129.1 (*C*H-Ar), 130.3 (CH=*C*H–COMe), 131.3 (*C*H-Ar), 132.3 (*C*-Cl), 136.1 (*C*-Ar), 136.7 (*C*-Ar), 138.2 (*C*H=CH-CO-Me), 142.4 (*C*H=CH–COOH), 170.2 (*C*OOH), 200.4 (*C*O-Me); *m/z* (NSI$^+$) 273 ([M+Na]$^+$, 100%), 523 ([2M+Na]$^+$, 60%); HRMS (NSI$^+$) $C_{13}H_{11}O_3ClNa$[M+Na]$^+$, found 273.0291, requires 273.0289 (+0.8 ppm).

(*E*)-3-(5-Methyl-2-((*E*)-3-oxobut-1-en-1-yl)phenyl)acrylic acid (S26)

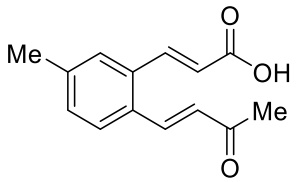

Following General Procedure 4, *tert*-butyl (*E*)-3-(5-methyl-2-((*E*)-3-oxobut-1-en-1-yl)phenyl)acrylate (941 mg, 3.28 mmol) gave the corresponding carboxylic acid (755 mg, quant.) as a yellow solid. mp 173–175 °C; ν_{max} (film) 3061–2506 br (carb. acid, O-H), 1689 (C=O), 1622 (C=O); ^{1}H NMR (500 MHz, $CDCl_3$) δ_H: 2.41 (3H, s, Ar-C-C*H*$_3$), 2.42 (3H, s, COC*H*$_3$), 6.39 (1H, d, *J* 15.8, CH=C*H*–COOH), 6.62 (1H, d, *J* 16.0, CH=C*H*-COMe), 7.16–7.18 (1H, m, C*H*-Ar), 7.43 (1H, s, C*H*-Ar), 7.52 (1H, d, *J* 8.0, C*H*-Ar), 7.86 (1H, d, *J* 16.0, C*H*=CH–CO-Me), 8.15 (1H, d, *J* 15.8, C*H*=CH–COOH); ^{13}C{^{1}H} NMR (126 MHz, $CDCl_3$) δ_C: 21.5 (Ar-C-*C*H$_3$), 27.9 (CO*C*H$_3$), 120.6 (CH=*C*H–COOH), 127.9 (*C*H-Ar), 128.6 (*C*H-Ar), 129.9 (CH=*C*H–COMe), 131.7 (*C*H-Ar), 131.9 (*C*-Ar), 134.1 (*C*-Ar), 139.9 (*C*H=CH-CO-Me), 140.9 (*C*-Me), 143.9 (*C*H=CH–COOH), 171.1 (*C*OOH), 198.4 (*C*O-Me); *m/z* (NSI$^+$) 483 ([2M+Na]$^+$, 100%), 253 ([M+Na]$^+$, 75%); HRMS (NSI$^+$) $C_{14}H_{14}O_3Na[M+Na]^+$, found 253.0833, requires 253.0835 (–0.9 ppm).

(*E*)-3-(4-Fluoro-2-((*E*)-3-Oxobut-1-en-1-yl)phenyl)acrylic (S27)

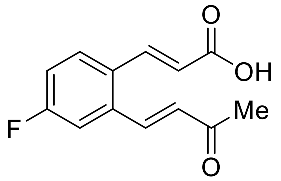

Following General Procedure 4, *tert*-Butyl (*E*)-3-(4-fluoro-2-((*E*)-3-oxobut-1-en-1-yl)phenyl)acrylate (424 mg, 1.46 mmol) gave the corresponding carboxylic acid (342 mg, quant.) as a yellow solid. mp 165–166 °C; ν_{max} (film) 3072–2525 br (carb. acid, O-H), 1682 (C=O), 1601 (C=O); ^{1}H NMR (500 MHz, $CDCl_3$) δ_H: 2.43 (3H, s, COC*H*$_3$), 6.34 (1H, d, *J* 15.8, CH=C*H*–COOH), 6.62 (1H, d, *J* 16.0, CH=C*H*-COMe), 7.15 (1H, td, *J* 8.2, 2.6, C*H*-Ar), 7.29 (1H, dd, *J* 9.4, 2.6, C*H*-Ar), 7.63 (1H, dd, *J* 8.6, 5.6, C*H*-Ar), 7.82 (1H, d, *J* 16.0, C*H*=CH–CO-Me), 8.08 (1H, d, *J* 15.8, C*H*=CH–COOH); ^{19}F NMR (282 MHz, $CDCl_3$) δ_F: –109.0; ^{13}C{^{1}H} NMR (126 MHz, $CDCl_3$) δ_C: 28.3 (CO*C*H$_3$), 114.4 (d, $^{2}J_{CF}$ = 22.6, C_{Ar}-F), 114.5 (*C*H-Ar), 117.8 (d, $^{2}J_{CF}$ = 22.1, C_{Ar}-F), 117.9 (*C*H-Ar), 120.6 (CH=*C*H–COOH), 130.2 (d, $^{3}J_{CF}$ = 8.7, C_{Ar}-F), 130.2 (*C*H-Ar), 131.4 (CH=*C*H–COMe), 136.9 (*C*-Ar), 137.0 (*C*-Ar), 138.4 (*C*H=CH-CO-Me), 142.5 (*C*H=CH–COOH), 164.9 (*C*-F), 170.7 (*C*OOH), 197.8 (*C*O-Me); *m/z* (NSI$^+$) 467 ([2M–H]$^–$,

100%), 233 ([M–H]$^-$, 80%); HRMS (NSI$^+$) $C_{13}H_{10}O_3F_1$[M–H]$^-$, found 233.0617, requires 233.0619 (–1.1 ppm).

3.5 Data for trichlorophenyl (TCP) esters

2,4,6-Trichlorophenyl (*E*)-3-(2-((*E*)-3-oxobut-1-en-1-yl)phenyl)acrylate (5)

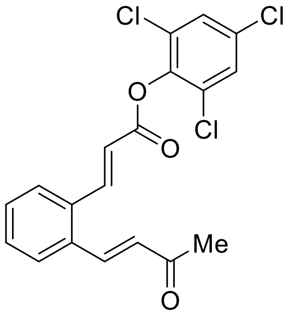

Following General Procedure 5, (*E*)-3-(2-((*E*)-3-Oxo-3-phenylprop-1-en-1-yl)phenyl)acrylic acid (1.36 g, 6.28 mmol), DCC (1.43 g, 6.91 mmol) and 2,4,6-trichlorophenol (TCP-OH) (1.28 g, 6.40 mmol) afforded after SiO_2-chromatography (90:10 Hexane : EtOAc, R_f 0.25) the title compound (2.04 g, 82%) as a brown solid. mp 85–86 °C; ν_{max} (film) 3077 (alkenyl C-H), 2858 (alkylC-H), 1741 (C=O) 1672 (C=O); ^{1}H NMR (500 MHz, $CDCl_3$) δ_H: 2.41 (3H, s, COCH_3), 6.62 (1H, d, *J* 15.8, CH=C*H*–COTCP), 6.64 (1H, d, *J* 16.1, CH=C*H*-COMe), 7.41 (2H, s, C*H*-Ar_{TCP}), 7.44–7.50 (2H, m, C*H*-Ar), 7.60–7.64 (1H, m, C*H*-Ar), 7.68–7.74 (1H, m, C*H*-Ar), 7.90 (1H, d, *J* 16.1, C*H*=CH–CO-Me), 8.31 (1H, d, *J* 15.8, C*H*=CH–COTCP); ^{13}C{^{1}H} NMR (126 MHz, $CDCl_3$) δ_C: 28.0 (CO*C*H_3), 118.9 (CH=*C*H–COTCP), 128.0 (*C*H-Ar), 128.1 (*C*H-Ar), 128.8 (2×*C*H-TCP), 129.8 (2×*C*-TCP), 130.5 (CH=*C*H–COMe), 131.1 (2×*C*H-Ar), 132.2 (*C*-Ar), 133.7 (*C*-Ar), 135.0(*C*-TCP), 139.7(*C*H=CH-CO-Me), 143.1 (CO*C*-TCP), 145.0 (*C*H=CH–COTCP), 162.6 (*C*OTCP), 198.0 (*C*O-Me); *m/z* (NSI$^+$) 413 ([M+NH_4]$^+$, 100%); HRMS (NSI$^+$) $C_{19}H_{14}O_3Cl_3$ [M+H]$^+$, found 395.0005, requires 395.0003 (+0.5 ppm).

2,4,6-Trichlorophenyl (*E*)-3-(2-((*E*)-4,4-dimethyl-3-oxopent-1-en-1-yl)phenyl)acrylate (S28)

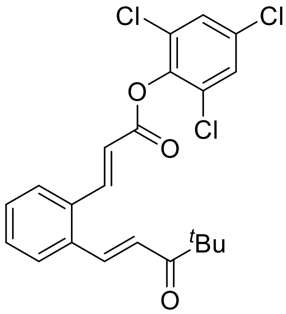

Following General Procedure 5, (*E*)-3-(2-((*E*)-4,4-dimethyl-3-oxopent-1-en-1-yl)phenyl)acrylic acid (0.36 g, 1.39 mmol), DCC (0.32 g, 1.53 mmol) and 2,4,6-trichlorophenol (TCP-OH) (0.28 g, 1.42 mmol) afforded after SiO_2-chromatography (90:10 Hexane : EtOAc, R_f 0.25) the title compound (509 mg, 84%) as a yellow solid. mp 113–115 °C; ν_{max} (film) 3120 (alkenyl C-H), 2967 (alkyl C-H), 1751 (C=O) 1676 (C=O); ^{1}H NMR (500 MHz, $CDCl_3$) δ_H: 1.23 (9H, s, C(C*H*$_3$)$_3$), 6.62 (1H, d, *J* 15.8, CH=C*H*–CO_2TCP), 6.99 (1H, d, *J* 15.5, CH=C*H*-CO*t*Bu), 7.41 (2H, s, C*H*-Ar$_{TCP}$), 7.42–7.53 (2H, m, C*H*-Ar), 7.58–7.65 (1H, m, C*H*-Ar), 7.70–7.72 (1H, m, C*H*-Ar), 8.01 (1H, d, *J* 15.5, C*H*=CH–CO^{t}Bu), 8.33 (1H, d, *J* 15.8, C*H*=CH–CO_2TCP); ^{13}C{^{1}H} NMR (126 MHz, $CDCl_3$) δ_C: 27.3 (C(*C*H$_3$)$_3$), 118.3 (CH=*C*H–CO_2TCP), 128.0 (*C*H-Ar), 128.7 (*C*H-Ar), 128.8 (2×*C*H-TCP), 129.9 (2×*C*-TCP), 130.1 (CH=*C*H–CO^{t}Bu), 130.9 (2×*C*H-Ar), 132.2 (*C*-Ar), 133.8 (*C*-Ar), 135.9 (*C*-TCP), 139.5 (*C*H=CH-CO^{t}Bu), 143.1 (CO$_2$*C*-TCP), 145.6 (*C*H=CH–CO_2TCP), 162.6 (*C*O$_2$TCP), 203.8 (*C*O^{t}Bu); *m/z* (NSI$^+$) 651 ([2M+Na]$^+$, 100%), 332 ([M+NH_4]$^+$, 65%); HRMS (NSI$^+$) $C_{22}H_{19}O_3Cl_3Na$ [M+Na]$^+$, found 459.0281, requires 459.0292 (–2.4 ppm).

(*E*)-2,4,6-Trichlorophenyl 3-(2-((*E*)-3-oxo-3-phenylprop-1-en-1-yl)phenyl)acrylate (S29)

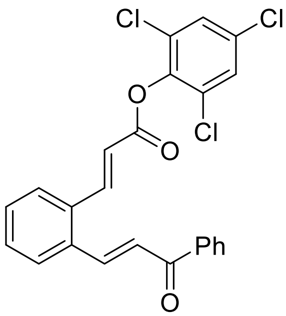

Following General Procedure 5, (*E*)-3-(2-((*E*)-3-Oxobut-1-en-1-yl)phenyl)acrylic acid (1.00 g, 3.60 mmol), DCC (816 mg, 3.96 mmol) and 2,4,6-trichlorophenol (TCP-OH) (732 mg, 3.67 mmol) afforded after SiO_2-chromatography (90:10 Hexane : EtOAc, Rf 0.25) the title compound (1.27 g, 77%) as a yellow solid. mp 64–67 °C; ν_{max} (film) 3068 (alkenyl C-H), 2843 (alkyl C-H), 1735 (C=O), 1672 (C=O);

^{1}H NMR (500 MHz, $CDCl_3$) δ_H: 6.64 (1H, d, *J* 15.8, CH=C*H*–COTCP), 7.41 (2H, s, C*H*-Ar_{TCP}), 7.43 (1H, d, *J* 15.5, CH=C*H*-CO-Ph), 7.47–7.53 (4H, m, C*H*-Ar), 7.58 (1H, m, C*H*-Ph), 7.73 (2H, m, C*H*-Ph), 8.03 (2H, m, C*H*-Ph), 8.17 (1H, d, *J* 15.6, C*H*=CH–COTCP), 8.39 (1H, d, *J* 15.8, C*H*=CH–CO-Ph),^{13}C{^{1}H} NMR (126 MHz, $CDCl_3$) δ_C: 118.7 (CH=*C*H–COTCP), 126.8 (CH=*C*H-CO-Ph), 128.1 (*C*H-Ar), 128.7 (*C*H-Ar), 128.8 (2×*C*H-TCP + 2×*C*H-Ph), 128.9 (2×*C*H-Ph), 129.9 (2×*C*-TCP), 130.4 (*C*H-Ar), 131.0 (*C*H-Ar), 132.2 (*C*-Ar), 133.3 (*C*H-Ph), 134.0 (*C*-Ar), 137.9 (*C*-Ph), 141.3 (*C*H=CH-CO-Ph), 143.1 (CO*C*-TCP), 145.4 (*C*H=CH–COTCP), 162.6 (*C*OTCP), 190.0 (*C*O-Ph); *m/z* (NSI^+) 458 ($[M+H]^+$, 15%); HRMS (NSI^+) $C_{24}H_{16}O_3Cl_3$ $[M+H]^+$, found 457.0155, requires 457.0160 (–1.0 ppm).

2,4,6-Trichlorophenyl (*E*)-3-(2-((*E*)-3-(4-bromophenyl)-3-oxoprop-1-en-1-yl)phenyl)acrylate (S30)

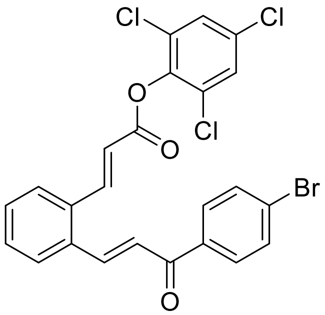

Following General Procedure 5, the corresponding acid (0.53 g, 1.48 mmol), DCC (0.34 g, 1.63 mmol) and 2,4,6-trichlorophenol (0.29 g, 1.50 mmol) afforded after SiO_2-chromatography (90:10 Hexane : EtOAc, R_f 0.25) the title compound (655 mg, 83%) as a pink solid. mp 65–66 °C; ν_{max} (film) 3076 (alkenyl C-H), 1739 (C=O) 1663 (C=O); ^{1}H NMR (500 MHz, $CDCl_3$) δ_H: 6.64 (1H, d, *J* 15.8, CH=C*H*–CO_2TCP), 7.37 (1H, d, *J* 15.5, CH=C*H*-$COPh_{Br}$), 7.42 (2H, s, C*H*-Ar_{TCP}), 7.47–7.54 (2H, m, C*H*-Ar), 7.63–7.64 (2H, m, C*H*-Ph_{Br}), 7.70–7.74 (2H, m, C*H*-Ar), 7.89–7.90 (2H, m, C*H*-Ph_{Br}), 8.17 (1H, d, *J* 15.5, C*H*=CH–$COPh_{Br}$), 8.38 (1H, d, *J* 15.8, C*H*=CH–CO_2TCP); ^{13}C{^{1}H} NMR (126 MHz, $CDCl_3$) δ_C: 118.8 (CH=*C*H–CO_2TCP), 126.2 (*C*H-Ar), 128.2 (*C*H-Ar), 128.4 (*C*-Ph_{Br}), 128.7 (2×*C*H-Ph_{Br}), 128.8 (2×*C*H-TCP), 129.9 (2×*C*-TCP), 130.3 (2×*C*H-Ph_{Br}), 130.6 (CH=*C*H-$COPh_{Br}$), 131.0 (2×*C*H-Ar), 132.2 (*C*-Ar), 134.1 (*C*-Ar), 135.4 (*C*-TCP), 136.6 (*C*H=CH-$COPh_{Br}$), 143.1 (CO_2*C*-TCP), 145.3 (*C*H=CH–CO_2TCP), 162.6 (*C*O_2TCP), 188.9 (*C*OPh_{Br}); *m/z* (NSI^+) 556 ($[M+Na]^+$, 65%); HRMS (NSI^+) $C_{24}H_{14}O_3BrCl_3Na$ $[M+Na]^+$, found 556.9074, requires 556.9084 (–1.8 ppm).

2,4,6-Trichlorophenyl (*E*)-3-(2-((*E*)-3-(4-chlorophenyl)-3-oxoprop-1-en-1-yl)phenyl)acrylate (S31)

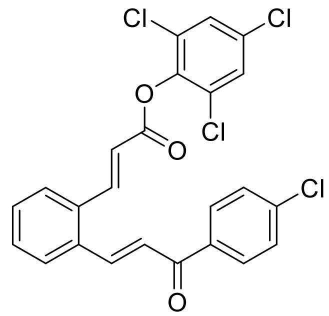

Following General Procedure 5, the corresponding acid (0.55 g, 1.77 mmol), DCC (0.40 g, 1.95 mmol) and 2,4,6-trichlorophenol (0.35 g, 1.81 mmol) afforded after SiO_2-chromatography (90:10 Hexane : EtOAc, R_f 0.25) the title compound (719 mg, 83%) as a pink solid. mp 106–108 °C; ν_{max} (film) 3082 (alkenyl C-H), 3020 (alkyl C-H), 1740 (C=O) 1667 (C=O); ^{1}H NMR (500 MHz, $CDCl_3$) δ_H: 6.64 (1H, d, *J* 15.8, CH=C*H*–CO_2TCP), 7.38 (1H, d, *J* 15.5, CH=C*H*-$COPh_{Cl}$), 7.41 (2H, s, C*H*-Ar_{TCP}), 7.46–7.48 (2H, m, C*H*-Ph_{Cl}), 7.49–7.54 (2H, m, C*H*-Ar), 7.68–7.75 (2H, m, C*H*-Ar), 7.96–7.98 (2H, m, C*H*-Ph_{Cl}), 8.17 (1H, d, *J* 15.5, C*H*=CH–$COPh_{Cl}$), 8.38 (1H, d, *J* 15.8, C*H*=CH–CO_2TCP); ^{13}C{^{1}H} NMR (126 MHz, $CDCl_3$) δ_C: 118.8 (CH=*C*H–CO_2TCP), 126.2 (*C*H-Ar), 128.2 (*C*H-Ar), 128.8 (2×*C*H-Ph_{Cl}), 129.2 (2×*C*H-TCP), 129.9 (2×*C*-TCP), 130.2 (2×*C*H-Ph_{Cl} + C*H*-Ar), 130.6 (CH=*C*H-$COPh_{Cl}$), 131.0 (2×*C*H-Ar), 132.2 (*C*-Ar), 134.0 (*C*-Ar), 135.4 (*C*-TCP), 136.2 (*C*-Ph_{Cl}), 139.7 (*C*-Ph_{Cl}), 141.8 (*C*H=CH-$COPh_{Cl}$), 143.1 (CO_2*C*-TCP), 145.3 (*C*H=CH–CO_2TCP), 162.6 (*C*O_2TCP), 188.7 (*C*OPh_{Cl}); *m/z* (NSI^+) 510 ($[M+NH_4]^+$, 90%); HRMS (NSI^+) $C_{24}H_{15}O_3Cl_4$ $[M+H]^+$, found 490.9763, requires 490.9770 (–1.4 ppm).

2,4,6-Trichlorophenyl (*E*)-3-(2-((*E*)-3-oxo-3-(4-(trifluoromethyl)phenyl)prop-1-en-1-yl)phenyl)-acrylate (S32)

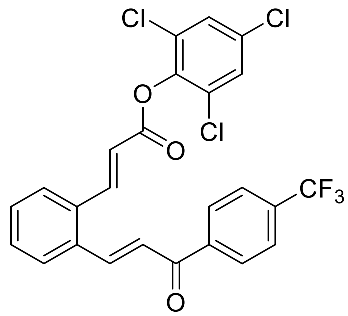

Following General Procedure 5, the corresponding acid (0.64 g, 1.86 mmol), DCC (0.42 g, 2.05 mmol) and 2,4,6-trichlorophenol (0.38 g, 1.90 mmol) afforded after SiO_2-chromatography (90:10 Hexane : EtOAc, R_f 0.3) the title compound (698 mg, 72%) as a yellow foam. mp 54–55 °C; ν_{max} (film) 3078 (alkenyl C-H), 2815 (alkylC-H), 1742 (C=O) 1667 (C=O); ^{1}H NMR (500 MHz, $CDCl_3$) δ_H: 6.65 (1H, d, *J*

15.8, CH=C*H*–CO_2TCP), 7.40 (1H, d, *J* 15.5, CH=C*H*-COPh$_{CF3}$), 7.41 (2H, s, C*H*-Ar$_{TCP}$), 7.50–7.54 (2H, m, C*H*-Ar), 7.70–7.74 (2H, m, C*H*-Ar), 7.75–7.77 (2H, m, C*H*-Ph$_{CF3}$), 8.11–8.13 (2H, m, C*H*-Ph$_{CF3}$), 8.20 (1H, d, *J* 15.5, C*H*=CH–COPh$_{CF3}$), 8.38 (1H, d, *J* 15.8, C*H*=CH–CO_2TCP); ^{19}F NMR (282 MHz, $CDCl_3$) δ_F: −63.1 (C*F*$_3$); ^{13}C{^{1}H} NMR (126 MHz, $CDCl_3$) δ_C: 119.0 (CH=*C*H–CO_2TCP), 125.9 (CH=*C*H-COPh$_{CF3}$), 126.0 (*C*H-Ph$_{CF3}$), 126.1 (*C*H-Ph$_{CF3}$), 126.1 (q, $^3J_{CF}$ = 4.5, C$_{Ar}$-CF_3), 127.1 (q, $^1J_{CF}$ = 263.7, C$_{Ar}$-CF_3), 128.2 (*C*H-Ar), 128.7 (*C*H-Ar), 128.8 (2×*C*H-Ph$_{CF3}$), 129.0 (2×*C*H-TCP), 129.8 (2×*C*-TCP), 130.8 (*C*H-Ar), 130.9 (q, $^2J_{CF}$ = 36.8, C$_{Ar}$-CF_3), 131.1 (*C*H-Ar), 132.3 (*C*-Ar), 134.2 (*C*-Ar + *C*F$_3$), 135.2 (*C*-Ph$_{CF3}$ + *C*-TCP), 142.5 (*C*H=CH-COPh$_{CF3}$), 143.1 (CO_2*C*-TCP + *C*-Ph$_{CF3}$), 145.2 (*C*H=CH–CO_2TCP), 162.5 (*C*O$_2$TCP), 189.1 (*C*OPh$_{CF3}$); *m/z* (NSI$^+$) 1073 ([2M+Na]$^+$, 100%), 1598 ([3M+Na]$^+$, 45%); HRMS (NSI$^+$) $C_{25}H_{18}O_3N_1Cl_3F_3$ [M+NH$_4$]$^+$, found 542.0289, requires 542.0299 (–1.8 ppm).

2,4,6-Trichlorophenyl (*E*)-3-(2-((*E*)-3-(4-methoxyphenyl)-3-oxoprop-1-en-1-yl)phenyl)acrylate (S33)

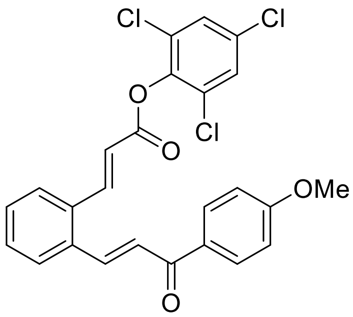

Following General Procedure 5, the corresponding acid (0.55 g, 1.80 mmol), DCC (0.41 g, 1.98 mmol) and 2,4,6-trichlorophenol (0.36 g, 1.84 mmol) afforded after SiO_2-chromatography (80:20 Hexane : EtOAc, R$_f$ 0.25) the title compound (777 mg, 88%) as a yellow foam. mp 56–57 °C; ν_{max} (film) 3075 (alkenyl C-H), 2843 (alkyl C-H), 1742 (C=O) 1597 (C=O); ^{1}H NMR (500 MHz, $CDCl_3$) δ_H: 3.88 (OC*H*$_3$), 6.65 (1H, d, *J* 15.8, CH=C*H*–CO_2TCP), 6.97 (2H, m, C*H*-Ph$_{OMe}$), 7.41 (2H, s, C*H*-Ar$_{TCP}$), 7.43 (1H, d, *J* 15.5, CH=C*H*-COPh$_{OMe}$), 7.45–7.52 (2H, m, C*H*-Ar), 7.70–7.74 (2H, m, C*H*-Ar), 8.03–8.05 (2H, m, C*H*-Ph$_{OMe}$), 8.14 (1H, d, *J* 15.5, C*H*=CH–COPh$_{OMe}$), 8.40 (1H, d, *J* 15.8, C*H*=CH–CO_2TCP); ^{13}C{^{1}H} NMR (126 MHz, $CDCl_3$) δ_C: 55.6 (O*C*H$_3$), 114.1 (2×*C*H-Ph$_{OMe}$), 118.5 (CH=*C*H–CO_2TCP), 126.9 (*C*H-Ph$_{OMe}$), 128.1 (*C*H-Ph$_{OMe}$), 128.1 (*C*H-Ar), 128.7 (*C*H-Ar), 128.8 (2×*C*H-TCP), 129.9 (*C*-TCP), 130.2 (*C*H=CH–COPh$_{OMe}$), 130.9 (*C*H-Ar), 131.0 (*C*H-Ar), 131.1 (2×*C*H- Ph$_{OMe}$), 132.2 (2×*C*-TCP), 133.9 (*C*-Ar), 135.9 (*C*-TCP), 140.5 (*C*H=CH-COPh$_{OMe}$), 143.1 (CO_2*C*-TCP + *C*-Ph$_{OMe}$), 145.6 (*C*H=CH–CO_2TCP), 162.6 (*C*O$_2$TCP), 188.2 (*C*OPh$_{OMe}$); *m/z* (NSI$^+$) 510 ([M+Na]$^+$, 100%), 997 ([2M+Na]$^+$, 55%); HRMS (NSI$^+$) $C_{25}H_{17}O_4Cl_3Na$ [M+Na]$^+$, found 509.0072, requires 509.0085 (–2.5ppm).

2,4,6-Trichlorophenyl (*E*)-3-(4-chloro-2-((*E*)-3-oxobut-1-en-1-yl)phenyl)acrylate (S34)

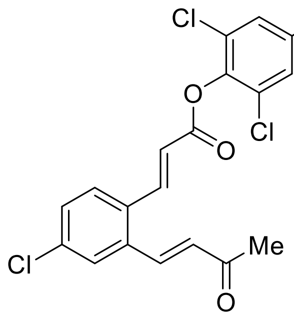

Following General Procedure 5, (*E*)-3-(4-chloro-2-((*E*)-3-oxobut-1-en-1-yl)phenyl)acrylic acid (730 mg, 2.80 mmol), DCC (635 mg, 3.08 mmol) and 2,4,6-trichlorophenol (TCP-OH) (564 mg, 2.86 mmol, 1.02 equiv) afforded after SiO_2-chromatography (90:10 Hexane : EtOAc, R_f 0.25) the title compound (1.04 g, 86%) as a yellow solid. mp 129–131 °C; ν_{max} (film) 3080 (alkenyl C-H), 2932 (alkyl C-H), 1746 (C=O) 1674 (C=O); ^{1}H NMR (500 MHz, $CDCl_3$) δ_H: 2.41 (3H, s, COC*H*$_3$), 6.61 (1H, d, *J* 15.8, CH=C*H*–COTCP), 6.65 (1H, d, *J* 16.0, CH=C*H*-COMe), 7.42 (2H, s, C*H*-Ar$_{TCP}$), 7.44 (2H, dd, *J* 8.4, 2.1, C*H*-Ar), 7.60 (1H, d, *J* 2.1, C*H*-Ar), 7.65 (1H, d, *J* 8.4, C*H*-Ar), 7.82 (1H, d, *J* 16.0, C*H*=CH–CO-Me), 8.22 (1H, d, *J* 15.8, C*H*=CH–COTCP); ^{13}C{^{1}H} NMR (126 MHz, $CDCl_3$) δ_C: 28.4 (CO*C*H$_3$), 119.2 (CH=*C*H–COTCP), 128.0 (*C*H-Ar), 128.9 (2×*C*H-TCP), 129.3 (*C*H-Ar), 129.8 (2×*C*-TCP), 130.5 (CH=*C*H–COMe), 131.8 (2×*C*H-Ar), 132.1 (*C*-Ar), 132.3 (*C*-Ar), 135.0 (*C*-TCP), 138.1 (*C*H=CH-CO-Me), 143.0 (CO*C*-TCP), 143.7 (*C*H=CH–COTCP), 162.4 (*C*OTCP), 197.5 (*C*O-Me); *m/z* (NSI$^+$) 453 ([M+Na]$^+$, 55%), 883 ([2M+Na]$^+$, 35%); HRMS (NSI$^+$) $C_{19}H_{16}O_3N_1Cl_4$ [M+NH$_4$]$^+$, found 445.9875, requires 445.9879 (–0.9 ppm).

2,4,6-Trichlorophenyl (*E*)-3-(5-methyl-2-((*E*)-3-oxobut-1-en-1-yl)phenyl)acrylate (S35)

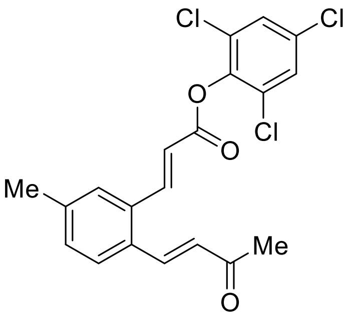

Following General Procedure 5, (*E*)-3-(5-methyl-2-((*E*)-3-oxobut-1-en-1-yl)phenyl)acrylic acid (858 mg, 3.73 mmol), DCC (924 mg, 3.08 mmol) and 2,4,6-trichlorophenol (TCP-OH) (751 mg, 3.80 mmol) afforded after SiO_2-chromatography (90:10 Hexane : EtOAc, R_f 0.25) the title compound (1.04 g, 68%) as a yellow solid. mp 129–131 °C; ν_{max} (film) 3069 (alkenyl C-H), 2928 (alkyl C-H), 1732 (C=O) 1667 (C=O); ^{1}H NMR (500 MHz, $CDCl_3$) δ_H: 2.40 (3H, s, COC*H*$_3$), 2.37 (3H, s, Ar-C*H*$_3$), 6.61 (1H, d, *J* 15.8, CH=C*H*–COTCP), 6.62 (1H, d, *J* 16.0, CH=C*H*-COMe), 7.29 (1H, d, *J* 9.5, C*H*-Ar), 7.42 (2H, s, C*H*-Ar$_{TCP}$), 7.51–7.54 (2H, m, C*H*-Ar), 7.87 (1H, d, *J* 16.0, C*H*=CH–CO-Me), 8.30 (1H, d, *J* 15.8, C*H*=CH–COTCP); ^{13}C{^{1}H} NMR (126 MHz, $CDCl_3$) δ_C: 21.4 (Ar-*C*H$_3$), 27.8 (CO*C*H$_3$), 118.5 (CH=*C*H–COTCP), 127.9 (*C*H-Ar), 128.5 (*C*H-Ar), 128.7 (2×*C*H-TCP), 129.7 (2×*C*-TCP), 130.1 (CH=*C*H–COMe), 132.0 (*C*H-Ar), 132.0 (*C*-Ar), 132.1 (*C*-Ar), 133.6 (*C*-TCP), 139.5 (*C*H=CH-CO-Me), 140.8 (Ar-*C*CH$_3$), 143.0 (CO*C*-TCP), 145.1 (*C*H=CH–COTCP), 162.5 (*C*OTCP), 198.0 (*C*O-Me); *m/z* (NSI$^+$) 841 ([2M+Na]$^+$, 100%), 432 ([M+Na]$^+$, 70%); HRMS (NSI$^+$) $C_{20}H_{19}O_3N_1Cl_3$ [M+NH$_4$]$^+$, found 426.0420, requires 426.0425 (–1.2 ppm).

2,4,6-Trichlorophenyl (*E*)-3-(4-fluoro-2-((*E*)-3-oxobut-1-en-1-yl)phenyl)acrylate (S36)

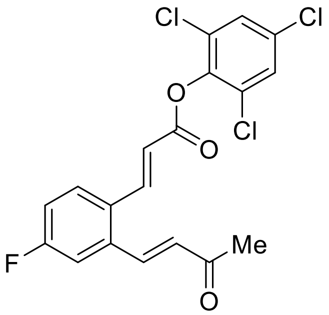

Following General Procedure 5, (*E*)-3-(4-fluoro-2-((*E*)-3-oxobut-1-en-1-yl)phenyl)acrylic acid (341 mg, 1.46 mmol), DCC (331 mg, 1.61 mmol) and 2,4,6-trichlorophenol (TCP-OH) (294 mg, 1.49 mmol) afforded after SiO_2-chromatography (90:10 Hexane : EtOAc, R_f 0.25) the title compound (543 mg, 90%) as a white solid. mp 139–141 °C; ν_{max} (film) 3080 (alkenyl C-H), 2832 (alkyl C-H), 1744 (C=O) 1672

(C=O); ^{1}H NMR (500 MHz, CDCl$_3$) δ_H: 2.41 (3H, s, COC*H*$_3$), 6.57 (1H, d, *J* 15.8, CH=C*H*–COTCP), 6.63 (1H, d, *J* 16.0, CH=C*H*-COMe), 7.18 (1H, td, *J* 8.2, 2.6, C*H*-Ar), 7.31 (1H, dd, *J* 9.4, 2.6, C*H*-Ar), 7.42 (2H, s, C*H*-Ar$_{TCP}$), 7.72 (1H, dd, *J* 8.7, 5.6, C*H*-Ar), 7.84 (1H, d, *J* 16.0, C*H*=CH–CO-Me), 8.24 (1H, d, *J* 15.8, C*H*=CH–COTCP); ^{19}F NMR (282 MHz, CDCl$_3$) δ_F: −108.2; ^{13}C{^{1}H} NMR (126 MHz, CDCl$_3$) δ_C: 28.3 (CO*C*H$_3$), 114.5 (*C*H-Ar), 114.6 (d, $^{2}J_{CF}$ = 22.6, C$_{Ar}$-F), 117.9 (*C*H-Ar), 117.9 (d, $^{2}J_{CF}$ = 22.1, C$_{Ar}$-F), 118.6 (CH=*C*H–COTCP), 128.8 (2×*C*H-TCP), 129.8 (2×*C*-TCP), 130.3 (*C*H-Ar), 130.3 (d, $^{3}J_{CF}$ = 8.8, C$_{Ar}$-F), 131.8 (CH=*C*H–COMe), 132.3 (*C*-Ar), 137.3 (*C*-Ar), 137.4 (*C*-TCP), 138.2 (*C*H=CH-CO-Me), 143.0 (CO*C*-TCP), 143.8 (*C*H=CH–COTCP), 162.5 (Ar*C*-F), 164.1 (d, $^{1}J_{CF}$ = 252.9, C$_{Ar}$-F), 165.1 (*C*OTCP), 197.5 (*C*O-Me); *m/z* (NSI$^+$) 849 ([2M+Na]$^+$, 100%), 436 ([M+Na]$^+$, 60%); HRMS (NSI$^+$) $C_{19}H_{16}O_3Cl_3F_1Na_1$ [M+Na]$^+$, found 434.9721, requires 434.9728 (−1.7 ppm).

3.6 Data for 1,3-diketones

1,3-*bis*(Furan-2-yl)propane-1,3-dione (S37)

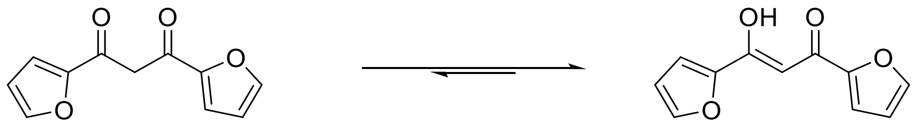

Following General Procedure 6, 2-acetylfuran (550 mg, 5.0 mmol), furoyl chloride (590 µL, 6.0 mmol) and LiHMDS (1.0 M in THF, 7.50 mL, 7.50 mmol) afforded after SiO$_2$-chromatography (80:20 Petrol : Et$_2$O) to afford the corresponding diketone as a pale-yellow solid (395 mg, 39%). mp 69-71 °C {Lit.[5] 70.5–72.0 °C}; ^{1}H NMR (500 MHz, CDCl$_3$) δ_H: 6.58 (2H, dd, *J* 3.5, 1.7, Ar(4)*H*), 6.65 (1H, s, =C*H*), 7.20 (2H, dd, *J* 3.5, 0.6, Ar(3)*H*), 7.61 (2H, dd, *J* 1.7, 0.6, Ar(5)*H*). Data in agreement with the literature.[6]

1,3-*bis*(4-Fluorophenyl)propane-1,3-dione (S38)

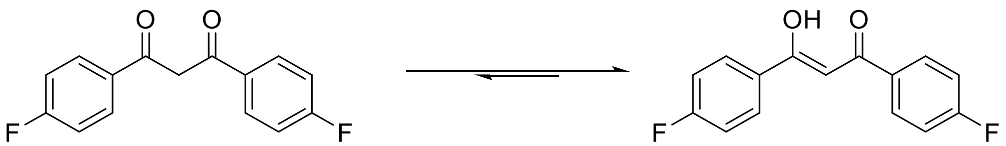

Following General Procedure 6, 4-fluoroacetophenone (690 mg, 5.0 mmol), 4-fluorobenzoyl chloride (620 µL, 6.0 mmol) and LiHMDS (1.0 M in THF, 7.50 mL, 7.50 mmol) and purified by chromatography (90:10 Petrol : Et$_2$O) to afford the corresponding diketone as a pink needle-like solid (780 mg, 60%), mp 108–110 °C {Lit.[7] 109 °C}; ^{1}H NMR (500 MHz, CDCl$_3$) δ_H: 6.75 (1H, s, =C*H*), 7.16–7.19 (4H, m, Ar*H*), 7.99–8.02 (4H, m, Ar*H*). Data in agreement with the literature.[8]

3.7 Data for acyl benzothiazoles

2-Phenacylbenzothiazole (34)

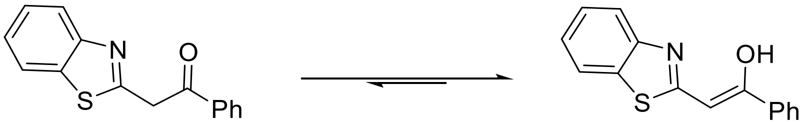

Following General Procedure 7, 2-chlorobenzothiazole (2.62 mL, 20.0 mmol), acetophenone (6.99 mL, 60.0 mmol) in anhydrous, degassed toluene (60 mL) and NaHMDS (30 mL, 2 M in THF, 12.0 mmol) afforded the title compound after trituration with cold hexane as a yellow solid (3.38 g, 67%). mp 115–117 °C {Lit.[9] 113–114 °C}; ^{1}H NMR (500 MHz, $CDCl_3$) δ_H: 4.85 (2H, s, *keto*-C*H*$_2$COAr), 6.38 (1H, s, *enol*-C*H*COHAr), 7.31 (1H, t, *J* 7.6, *enol*benzothiazoleC(6)*H*), 7.39 (1H, t, *J* 7.5, *enol*-benzothiazoleC(6)*H*), 7.42–7.52 (6H, m, Ar*H*), 7.51 (1H, t, *J* 7.7, *enol*-benzothiazoleC(5)*H*), 7.62 (1H, t, *J* 7.4, *keto*-benzothiazoleC(5)*H*), 7.79 (1H, d, *J* 7.9, *enol*benzothiazoleC(4)*H*), 7.82 (1H, d, *J* 8.2, *enol*-benzothiazoleC(7)*H*)), 7.88 (3H, m, *keto*-benzothiazole C(4)*H, enol*-phenacyl C(2')*H*), 8.02 (1H, d, *J* 8.0, *keto*-benzothiazole C(7)*H*), 8.10 (2H, d, *J* 7.5 *keto*phenacyl C(2')*H*). Data in agreement with the literature.[9]

2-(Benzo[d]thiazol-2-yl)-1-(4-fluorophenyl)ethan-1-one (50)

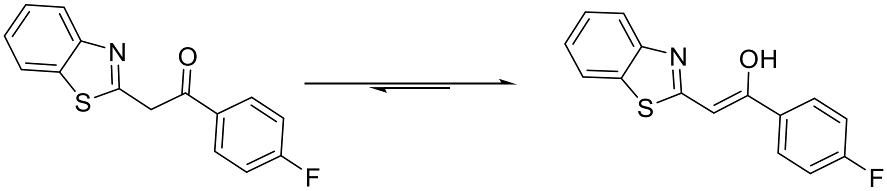

Following General Procedure 7, 2-chlorobenzothiazole (0.65 mL, 5.0 mmol), 1-(4-fluorophenyl)ethan-1-one (1.80 mL, 15.0 mmol) in anhydrous, degassed toluene (15 mL) and NaHMDS (7.50 mL, 2 M in THF, 12.0 mmol) afforded the title compound after trituration with cold hexane as a yellow solid (3.38 g, 67%). mp 122–123 °C; ν_{max} (film) 3049 (alkenyl C-H), 1614 (C=O), 1581 (C=C, aromatic), 1475 (C=C, heteroaromatic), 1138 (C-N); ^{1}H NMR (500 MHz, $CDCl_3$) δ_H: 4.80 (2H, s, *keto*-C*H*$_2$COAr), 6.31 (1H, s, *enol*-C*H*COHAr), 7.13 (2H, t, *J* 8.7, ArH_{enol}), 7.17 (2H, t, *J* 8.7, ArH_{keto}), 7.29–7.31 (1H, m, ArH_{enol}), 7.38–7.41 (1H, m, ArH_{keto}), 7.44–7.46 (1H, m, ArH_{enol}), 7.44–7.46 (1H, m, ArH_{enol}), 7.46–7.49 (1H, m, ArH_{keto}), 7.79 (2H, t, *J* 8.8, *enol*-benzothiazole C*H*), 7.86–7.89 (1H, m, *enol*-benzothiazole C*H*), 7.87–7.89 (2H, m, *keto*-benzothiazole C*H*), 8.01 (1H, d, *J* 8.0, *keto*-benzothiazole C*H*), 8.12–8.15 (2H, m, *keto/enol*-benzothiazole C*H*); ^{19}F NMR (282 MHz, $CDCl_3$) δ_F: −110.0 (*enol*), −103.5 (*keto*); ^{13}C{^{1}H} NMR (126 MHz, $CDCl_3$) δ_C: 44.1 (*keto*-*C*H$_2$-C=O), 90.6 (*enol*-*C*H=COH), 115.7 (d, $^{2}J_{CF}$ = 21.8, *enol*-

C$_{Ar}$-F), 116.2 (d, $^2J_{CF}$ = 21.0, *keto*-C$_{Ar}$-F), 115.7 (*enol*-Ar*C*H), 115.8 (*enol*-Ar*C*H), 116.1 (*keto*-Ar*C*H), 116.3 (*keto*-Ar*C*H), 119.9 (*enol*-benzothiazole*C*H), 121.6 (*enol*-benzothiazole*C*H), 121.7 (*keto*-benzothiazole*C*H), 123.0 (benzothiazole*C*), 123.1 (*keto*-benzothiazole*C*H), 124.3 (*enol*-benzothiazole*C*H), 125.3 (*keto*-benzothiazole*C*H), 126.2 (*keto*-benzothiazole*C*H), 126.7 (*enol*-benzothiazole*C*H), 127.0 (benzothiazole*C*), 128.2 (d, $^3J_{CF}$ = 8.4, C$_{Ar}$-F), 128.2 (2×*enol*-Ar*C*H), 131.3 (Ar*C*), 131.6 (*keto*-Ar*C*H), 131.7 (*keto*-Ar*C*H), 150.1 (benzothiazole*C*), 165.2 (*enol*-*C*=N), 165.4 (*enol*-Ar*C*-F), 166.4 (d, $^1J_{CF}$ = 248.7, C$_{Ar}$-F), 167.3 (*keto*-*C*=N), 167.3 (*keto*-Ar*C*-F), 168.1 (*enol*-CH=*C*OH), 192.7 (*keto*-C=O); *m/z* (NSI$^+$) 272 ([M+H]$^+$, 100%); HRMS (NSI$^+$) $C_{15}H_{11}F_1N_1O_1S_1$ [M+H]$^+$, found 272.0542, requires 272.0540 (+0.8 ppm).

2-(Benzo[d]thiazol-2-yl)-1-(4-bromophenyl)ethan-1-one (S39)

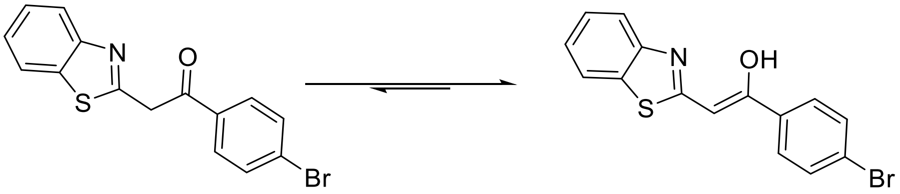

Following General Procedure 7, 2-chlorobenzothiazole (0.65 mL, 5.0 mmol), 1-(4-bromophenyl)ethan-1-one (1.80 mL, 15.0 mmol) in anhydrous, degassed toluene (15 mL) and NaHMDS (7.50 mL, 2 M in THF, 12.0 mmol) afforded the title compound after trituration with cold hexane as a yellow solid (3.38 g, 67%). mp 122–123 °C; ν_{max} (film) 3049 (alkenyl C-H), 1614 (C=O), 1581 (C=C, aromatic), 1475 (C=C, heteroaromatic), 1138 (C-N); ^{1}H NMR (500 MHz, CDCl$_3$) δ_H: 4.80 (2H, s, *keto*-C*H*$_2$COAr), 6.31 (1H, s, *enol*-C*H*COHAr), 7.13 (2H, t, *J* 8.7, Ar*H*$_{enol}$), 7.17 (2H, t, *J* 8.7, Ar*H*$_{keto}$), 7.29–7.31 (1H, m, Ar*H*$_{enol}$), 7.38–7.41 (1H, m, Ar*H*$_{keto}$), 7.44–7.46 (1H, m, Ar*H*$_{enol}$), 7.44–7.46 (1H, m, Ar*H*$_{enol}$), 7.46–7.49 (1H, m, Ar*H*$_{keto}$), 7.79 (2H, t, *J* 8.8, *enol*-benzothiazole C*H*), 7.86–7.89 (1H, m, *enol*-benzothiazole C*H*), 7.87–7.89 (2H, m, *keto*-benzothiazole C*H*), 8.01 (1H, d, *J* 8.0, *keto*-benzothiazole C*H*), 8.12–8.15 (2H, m, *keto/enol*-benzothiazole C*H*); ^{13}C{^{1}H} NMR (126 MHz, CDCl$_3$) δ_C: 44.1 (*keto*-*C*H$_2$-C=O), 90.6 (*enol*-*C*H=COH), 115.7 (*enol*-Ar*C*H), 115.8 (*enol*-Ar*C*H), 116.1 (*keto*-Ar*C*H), 116.3 (*keto*-Ar*C*H), 119.9 (*enol*-benzothiazole*C*H), 121.6 (*enol*-benzothiazole*C*H), 121.7 (*keto*-benzothiazole*C*H), 123.0 (benzothiazole*C*), 123.1 (*keto*-benzothiazole*C*H), 124.3 (*enol*-benzothiazole*C*H), 125.3 (*keto*-benzothiazole*C*H), 126.2 (*keto*-benzothiazole*C*H), 126.7 (*enol*-benzothiazole*C*H), 127.0 (benzothiazole*C*), 128.2 (2×*enol*-Ar*C*H), 131.3 (Ar*C*), 131.6 (*keto*-Ar*C*H), 131.7 (*keto*-Ar*C*H), 150.1 (benzothiazole*C*), 165.2 (*enol*-*C*=N), 165.4 (*enol*-Ar*C*-F), 167.3 (*keto*-*C*=N), 168.1 (*keto*-Ar*C*-F), 180.4 (*enol*-CH=*C*OH), 192.7 (*keto*-C=O); *m/z* (NSI$^+$) 333 ([M+H]$^+$, 100%); HRMS (NSI$^+$) $C_{15}H_{11}O_1N_1Br_1S_1$ [M+H]$^+$, found 331.9744, requires 331.9739 (+1.4 ppm).

2-(Benzo[d]thiazol-2-yl)-1-(4-methoxyphenyl)ethan-1-one (S40)

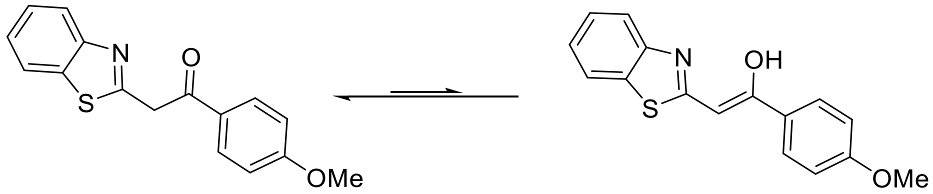

Following General Procedure 7, 2-chlorobenzothiazole (0.65 mL, 5.0 mmol), 1-(4-methoxyphenyl)ethan-1-one (2.25 g, 15.0 mmol) in anhydrous, degassed toluene (15 mL) and NaHMDS (7.50 mL, 2 M in THF, 12.0 mmol) afforded the title compound after trituration with cold hexane as a yellow solid (0.44 g, 31%). mp 104–106 °C; ν_{max} (film) 3057 (alkenyl C-H), 1603 (C=O), 1574 (C=C, aromatic), 1472 (C=C, heteroaromatic), 1177 (C-N); ^{1}H NMR (500 MHz, $CDCl_3$) δ_H: 3.87 (3H, s, *enol*-C*H*$_3$), 3.88 (3H, s, *keto*-C*H*$_3$), 4.78 (2H, s, *keto*-C*H*$_2$COAr), 6.29 (1H, s, *enol*-C*H*COHAr), 6.93–6.95 (2H, m, Ar*H*$_{enol}$), 6.96–6.98 (2H, m, Ar*H*$_{keto}$), 7.36–7.39 (2H, m, *enol*-benzothiazole C*H*), 7.43–7.45 (1H, m, *enol*-benzothiazole C*H*), 7.45–7.48 (2H, m, *keto*-benzothiazole C*H*), 7.76–7.78 (2H, m, Ar*H*$_{enol}$), 7.83–7.87 (1H, m, *keto*-benzothiazole C*H*), 7.87–7.88 (1H, m, *keto*-benzothiazole C*H*), 7.93–7.95 (1H, m, *enol*-benzothiazole C*H*), 8.01 (1H, d, *J* 8.0, *keto*-benzothiazole C*H*), 8.07–8.09 (2H, m, Ar*H*$_{keto}$); ^{13}C{^{1}H} NMR (126 MHz, $CDCl_3$) δ_C: 43.9 (*keto*-*C*H$_2$-C=O), 55.6 (*enol*-*C*H$_3$), 55.7 (*keto*-*C*H$_3$), 89.7 (*enol*-*C*H=COH), 114.0 (2×*enol*-Ar*C*H), 114.2 (2×*keto*-Ar*C*H),) 119.7 (*enol*-benzothiazole*C*H), 121.5 (*enol*-benzothiazole*C*H), 121.7 (*keto*-benzothiazole*C*H), 123.0 (*keto*-benzothiazole*C*H), 123.2 (benzothiazole*C*), 124.1 (*enol*-benzothiazole*C*H), 125.2 (*keto*-benzothiazole*C*H), 126.1 (*keto*-benzothiazole*C*H), 126.6 (*enol*-benzothiazole*C*H), 126.7 (benzothiazole*C*), 127.8 (2×*enol*-Ar*C*H), 131.2 (Ar*C*), 131.3 (2×*keto*-Ar*C*H), 151.2 (*enol*-benzothiazole*C*), 152.7 (*keto*-benzothiazole*C*), 161.5 (*enol*-*C*=N), 164.0 (*keto*-*C*=N), 164.1 (*keto*-Ar*C*-OMe), 168.1 (*enol*-Ar*C*-OMe), 168.2 (*enol*-CH=*C*OH), 192.6 (*keto*-C=O); *m/z* (NSI$^+$) 284 ([M+H]$^+$, 100%); HRMS (NSI$^+$) $C_{16}H_{14}O_2N_1S_1$ [M+H]$^+$, found 284.0739, requires 284.0740 (–0.3 ppm).

2-(6-Fluorobenzo[d]thiazol-2-yl)-1-phenylethan-1-one (S41)

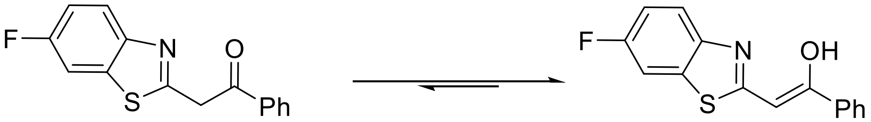

Following General Procedure 7, 6-fluoro-2-chlorobenzothiazole (938 mg, 5.0 mmol), acetophenone (1.70 mL, 15.0 mmol) in anhydrous, degassed toluene (15 mL) and NaHMDS (7.50 mL, 2 M in THF, 12.0 mmol) afforded the title compound after trituration with cold hexane as a yellow solid (1.02 g, 75%). mp 128–129 °C; ν_{max} (film) 3065 (alkenyl C-H), 1614 (C=O), 1564 (C=C, aromatic), 1452 (C=C, heteroaromatic), 1134 (C-N); ^{1}H NMR (500 MHz, $CDCl_3$) δ_H: 4.82 (2H, s, *keto*-C*H*$_2$COAr), 6.34 (1H, s,

enol-C*H*COHAr), 7.17–7.23 (2H, m, *enol*/*keto*-benzothiazole C*H*), 7.44–7.46 (3H, m, ArH_{enol}), 7.49–7.53 (3H, m, ArH_{keto}), 7.55–7.57 (1H, m, *keto*-benzothiazole C*H*), 7.61–7.64 (1H, m, *enol*-benzothiazole C*H*), 7.77–7.80 (1H, m, ArH_{enol}), 7.86–7.88 (2H, m, *enol/keto*-benzothiazole C*H*), 7.93–7.96 (1H, m, ArH_{enol}), 8.07–8.09 (2H, m, ArH_{keto}); ^{19}F NMR (282 MHz, $CDCl_3$) δ_F: –117.1 (*keto*), –116.2 (*enol*); ^{13}C{^{1}H} NMR (126 MHz, $CDCl_3$) δ_C: 43.8 (*keto*-CH_2-C=O), 91.2 (*enol*-*C*H=COH), 107.9 (d, $^2J_{CF}$ = 27.1, *keto*-C_{Ar}-F), 108.0 (d, $^2J_{CF}$ = 27.1, *enol*-C_{Ar}-F), 108.0 (*keto*-benzothiazole*C*H) , 108.1 (*enol*-benzothiazole*C*H), 114.8 (*keto*-benzothiazole*C*H), 114.8 (d, $^2J_{CF}$ = 20.8, *keto*-C_{Ar}-F), 115.0 (d, $^2J_{CF}$ = 20.9, *enol*-C_{Ar}-F), 115.1 (*enol*-benzothiazole*C*H), 121.3 (*keto*-benzothiazole*C*H), 124.0 (*enol*-benzothiazole*C*H), 126.0 (2×*enol*-Ar*C*H), 128.7 (2×*enol*-Ar*C*H), 128.8 (d, $^3J_{CF}$ = 9.5, C_{Ar}-F), 128.8 (2×*keto*-Ar*C*H), 129.1 (2×*keto*-Ar*C*H), 130.5 (*enol*-Ar*C*H), 134.1 (*keto*-Ar*C*H), 134.5 (benzothiazole*C*), 148.0 (benzothiazole*C*), 160.9 (*enol*-*C*=N), 162.1 (d, $^1J_{CF}$ = 303.9, C_{Ar}-F), 163.2 (*keto*-*C*=N), 180.4 (*enol*-CH=*C*OH), 192.7 (*keto*-C=O); *m/z* (NSI$^+$) 272 ([M+H]$^+$, 100%); HRMS (NSI$^+$) $C_{15}H_{11}F_1N_1O_1S_1$ [M+H]$^+$, found 272.0543, requires 272.0540 (+1.1ppm).

2-(6-Bromobenzo[d]thiazol-2-yl)-1-phenylethan-1-one (S42)

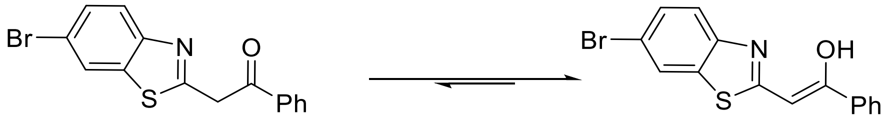

Following General Procedure 7, 6-bromo-2-chlorobenzothiazole (1.24 g, 5.0 mmol), acetophenone (1.70 mL, 15.0 mmol) in anhydrous, degassed toluene (15 mL) and NaHMDS (7.50 mL, 2 M in THF, 12.0 mmol) afforded the title compound after trituration with cold hexane as a yellowish solid (562 mg, 34%). mp 165–166 °C; ν_{max} (film) 3078 (alkenyl C-H), 1609 (C=O), 1595 (C=N), 1473 (C=C, heteroaromatic), 1134 (C-N); ^{1}H NMR (500 MHz, $CDCl_3$) δ_H: 4.82 (2H, s, *keto*-CH_2COAr), 6.36 (1H, s, *enol*-C*H*COHAr), 7.43–7.47 (3H, m, ArH_{enol}), 7.50–7.54 (1H, m, *enol*-benzothiazole C*H*), 7.54–7.58 (2H, m, ArH_{keto}), 7.62–7.65 (1H, m, *keto*-benzothiazole C*H*), 7.70 (1H, d, *J* 8.6, ArH_{keto}), 7.85–7.88 (2H, m, ArH_{enol}), 7.93 (1H, d, *J* 1.8, *enol*-benzothiazole C*H*), 8.03 (1H, d, *J* 1.8, *keto*-benzothiazole C*H*), 8.07–8.09 (2H, m, ArH_{keto}); ^{13}C{^{1}H} NMR (126 MHz, $CDCl_3$) δ_C: 43.8 (*keto*-*C*H_2-C=O), 91.1 (*enol*-*C*H=COH), 117.5 (*C*-Br), 121.5 (*enol*-benzothiazole*C*H) , 124.1 (*enol*-benzothiazole*C*H), 124.1 (*enol*-benzothiazole*C*H) , 124.3 (*enol*-benzothiazole*C*H), 126.1 (2×*enol*-Ar*C*H), 126.4 (*C*-Ar), 128.8 (2×*enol*-Ar*C*H), 129.1 (2×*keto*-Ar*C*H), 130.0 (2×*keto*-Ar*C*H), 130.7 (*enol*-Ar*C*H), 134.2 (*keto*-Ar*C*H), 138.8 (benzothiazole*C*), 148.0 (benzothiazole*C*), 160.9 (*enol*-*C*=N), 165.0 (*keto*-*C*=N), 180.4 (*enol*-CH=*C*OH), 192.7 (*keto*-C=O); *m/z* (NSI$^+$) 333 ([M+H]$^+$, 100%); HRMS (NSI$^+$) $C_{15}H_{11}Br_1N_1O_1S_1$ [M+H]$^+$, found 331.9743, requires 331.9739 (+1.1ppm).

2-(6-Methoxybenzo[d]thiazol-2-yl)-1-phenylethan-1-one (S43)

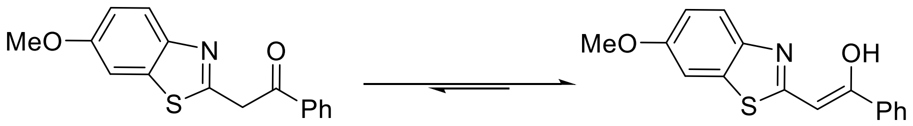

Following General Procedure 7, 6-methoxy-2-chlorobenzothiazole (998 mg, 5.0 mmol), acetophenone (1.70 mL, 15.0 mmol) in anhydrous, degassed toluene (15 mL) and NaHMDS (7.50 mL, 2 M in THF, 12.0 mmol) afforded the title compound after trituration with cold hexane as a yellow solid (878 mg, 62%). mp 156–157 °C; ν_{max} (film) 1599 (C=O), 1456 (C=C, heteroaromatic), 1134 (C-N), 824 (C=C, aromatic); ^{1}H NMR (500 MHz, $CDCl_3$) δ_H: 3.87 (3H, s, *keto*-OC*H*$_3$), 3.88 (3H, s, *enol*-OC*H*$_3$), 4.79 (2H, s, *keto*-C*H*$_2$COAr), 6.32 (1H, s, *enol*-C*H*COHAr), 7.05–7.08 (2H, m, *enol*/*keto*-benzothiazole C*H*), 7.30 (2H, dd, *J* 18.3, 2.5, *enol*-benzothiazole C*H*), 7.43–7.45 (3H, m, ArH_{enol}), 7.49–7.52 (2H, m, *keto*-benzothiazole C*H*), 7.60–7.63 (1H, m, ArH_{keto}), 7.74 (1H, d, *J* 8.9, ArH_{keto}), 7.85–7.87 (2H, m, ArH_{enol}), 7.99 (1H, d, *J* 9.1, ArH_{keto}), 8.07–8.09 (2H, m, ArH_{keto}); ^{13}C{^{1}H} NMR (126 MHz, $CDCl_3$) δ_C: 43.9 (*keto*-*C*H$_2$-C=O), 56.0 (*keto*, *enol*-O*C*H$_3$), 91.3 (*enol*-*C*H=COH), 104.1 (*keto*-benzothiazole*C*H), 104.6 (*enol*-benzothiazole*C*H), 115.5 (*enol*-benzothiazole*C*H), 115.6 (*keto*-benzothiazole*C*H), 121.1 (*keto*-benzothiazole*C*H), 123.5 (*enol*-benzothiazole*C*H), 125.9 (2×*enol*-Ar*C*H), 126.4 (*C*-Ar), 128.7 (2×*enol*-Ar*C*H), 128.9 (2×*keto*-Ar*C*H), 129.0 (2×*keto*-Ar*C*H), 130.2 (*enol*-Ar*C*), 134.0 (*enol*-Ar*C*H), 134.8 (*keto*-Ar*C*H), 136.0 (*keto*-benzothiazole*C*), 137.4 (*keto*-benzothiazole*C*), 145.7 (*enol*-benzothiazole*C*), 147.4 (*keto*-benzothiazole*C*), 157.2 (*enol*-*C*-OMe), 157.8 (*keto*-*C*-OMe), 157.8 (*keto*-*C*-OMe), 160.9 (*enol*-*C*=N), 163.5 (*keto*-*C*=N), 166.3 (*enol*-*C*OH), 194.5 (*keto*-C=O); *m/z* (NSI^+) 284 ($[M+H]^+$, 100%); HRMS (NSI^+) $C_{16}H_{14}O_2N_1S_1$ $[M+H]^+$, found 284.0740, requires 284.0740 (+0.1ppm)

3.8 Data for acyl benzimidazoles

2-Phenacylbenzimidazole (53)

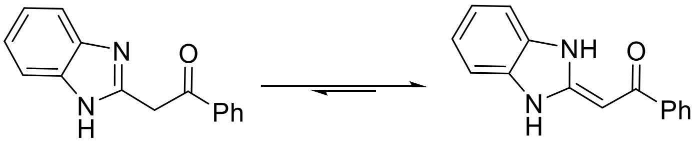

Following the General Procedure 8, 2-methylbenzimidazole (0.66 g, 5.0 mmol), benzoyl chloride (1.92 mL, 16.5 mmol)) and NEt_3 (2.29 mL, 16.5 mmol) in HPLC grade acetonitrile (5 mL) gave the *ester* as a yellow solid. Heating the ester at reflux in 1-BuOH:DMF (5 mL) for 1 h afforded the title compound after filtration as a yellow solid as a 4:1 mixture of enamino:keto tautomers (531 mg, 45%). mp 176–178 °C {Lit.[10] 178–179 °C}; ^{1}H NMR (500 MHz, DMSO-d$_6$) δ_H: 4.69 (2H, s, *keto*-CH_2CO), 6.11 (1H, s, *enamino*-C*H*CO), 7.12–7.21 (4H, m, benzimidazoleC(5)*H* and benzimidazoleC(6)*H*), 7.38–7.60 (9H, m, Ar*H*), 7.62–7.72 (1H, m, *keto*-phenylC(4)*H*), 7.83–7.92 (2H, m, 2×*enamino*-phenylC(2)*H*), 8.04–8.13 (2H, m, 2×*keto*-phenylC(2)*H*), 12.27 (1H, s, *enamino*-N*H*), 12.31 (1H, s, *keto*-N*H*). Data in agreement with the literature.[10]

2-(4-Methoxy)phenacylbenzimidazole (S44)

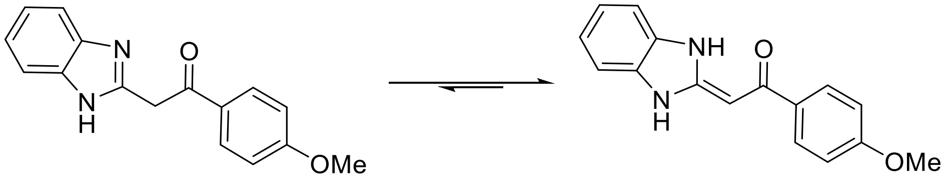

Following the General Procedure 8, 2-methylbenzimidazole (0.66 g, 5.0 mmol), 4-methoxybenzoyl chloride (2.56 g, 15.0 mmol) and NEt_3 (2.10 mL, 15.0 mmol) in HPLC grade acetonitrile (5 mL) and subsequent hydrolysis in morpholine (2.9 mL) afforded the title compound after filtration as a yellow solid as a 1.1:1 mixture of enamino:keto tautomers (866 mg, 65%). mp 205–207 °C {Lit.[10] 208–209 °C}; ^{1}H NMR (500 MHz, DMSO-d$_6$) δ_H: 3.81 (3H, *enamino*-OCH_3), 3.85 (3H, *keto*-OCH_3), 4.59 (2H, s, *keto*-CH_2CO), 6.00 (1H, s, *enamino*-C*H*CO), 7.01 (2H, d, *J* 8.9, *enamino*-H-Ar$_{OMe}$), 7.08 (2H, d, *J* 8.9, *enamino*-H-Ar$_{OMe}$), 7.14–7.16 (4H, m, benzimidazoleC(5)*H* and benzimidazoleC(6)*H*), 7.36 (1H, dd, *J* 5.9, 3.2, *enamino*-benzimidazoleC(4)*H*), 7.45–7.46 (1H, m, *keto*-benzimidazoleC(4)*H*), 7.53 (2H, dd, *J* 5.8, 3.2, *enamino/keto*-benzimidazoleC(7)*H*), 7.80 (2H, d, *J* 8.9, *keto*-H-Ar$_{OMe}$), 8.07 (2H, d, *J* 8.9, *keto*-H-Ar$_{OMe}$), 12.17 (1H, s, *keto*-N*H*), 12.32 (1H, s, *enamino*-N*H*). Data in agreement with the literature.[10]

2-(4-Nitro)phenacylbenzimidazole (S45)

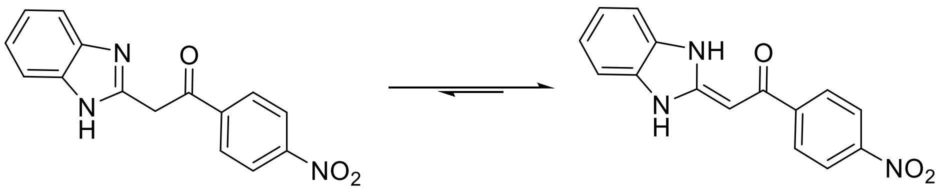

Following the General Procedure 8, 2-methylbenzimidazole (0.66 g, 5.0 mmol), 4-nitrobenzoyl chloride (2.78 g, 15.0 mmol) and NEt_3 (2.10 mL, 15.0 mmol) in HPLC grade acetonitrile (5 mL) and subsequent hydrolysis in 1-BuOH:DMF (8 mL) afforded the title compound after filtration as a red solid as a 1.1:1 mixture of enamino:keto tautomers (947 mg, 67%). mp 270–272 °C {Lit.[10] 266–268 °C}; ^{1}H NMR (500 MHz, DMSO-d_6) δ_H: 3.81 (3H, *enamino*-OCH_3), 3.85 (3H, *keto*-OCH_3), 4.59 (2H, s, *keto*-CH_2CO), 6.00 (1H, s, *enamino*-C*H*CO), 7.01 (2H, d, *J* 8.9, *enamino*-H-Ar_{OMe}), 7.08 (2H, d, *J* 8.9, *enamino*-H-Ar_{OMe}), 7.14–7.16 (4H, m, benzimidazoleC(5)*H* and benzimidazoleC(6)*H*), 7.36 (1H, dd, *J* 5.9, 3.2, *enamino*-benzimidazoleC(4)*H*), 7.45–7.46 (1H, m, *keto*-benzimidazoleC(4)*H*), 7.53 (2H, dd, *J* 5.8, 3.2, *enamino/keto*-benzimidazoleC(7)*H*), 7.80 (2H, d, *J* 8.9, *keto*-H-Ar_{OMe}), 8.07 (2H, d, *J* 8.9, *keto*-H-Ar_{OMe}), 12.17 (1H, s, *keto*-N*H*), 12.32 (1H, s, *enamino*-N*H*). Data in agreement with the literature.[10]

2-(4-Trifluoromethyl)phenacylbenzimidazole (S46)

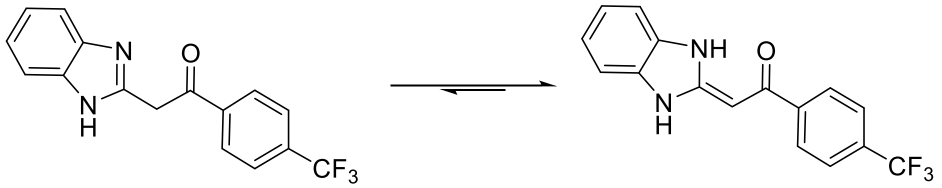

Following the General Procedure 8, 2-methylbenzimidazole (0.66 g, 5.0 mmol), 4-trifluoromethylbenzoyl chloride (2.23 mL, 15.0 mmol) and NEt_3 (2.10 mL, 15.0 mmol) in HPLC grade acetonitrile (5 mL) and subsequent hydrolysis in 1-BuOH:DMF (8 mL) afforded the title compound after filtration as a yellow solid as a 25:1 mixture of enamino:keto tautomers (1.708 g, 69%). mp 240–241 °C; ν_{max} (film) 3009 (N-H), 2758 (C-H), 1616 (C=O), 1327 (C_{Ar}-C_{Ar}); ^{1}H NMR (500 MHz, DMSO-d_6) δ_H: 4.75 (2H, s, *keto*-CH_2CO), 6.09 (1H, s, *enamino*-C*H*CO), 7.17–7.20 (2H, m, *enamino*-benzimidazoleC(5)*H* and benzimidazoleC(6)*H*), 7.37–7.40 (1H, m, benzimidazoleC(4)*H*), 7.57–7.59 (1H, m, *enamino*-benzimidazoleC(7)*H*), 7.81 (2H, d, *J* 8.4, *enamino*-H-Ar_{CF3}), 7.95 (2H, d, *J* 8.4, *keto*-H-Ar_{CF3}), 8.05 (2H, d, *J* 8.4, *enamino*-H-Ar_{CF3}), 8.27 (2H, d, *J* 8.4, *keto*-H-Ar_{CF3}), 12.33 (1H, s, *enamino*-N*H*); ^{19}F NMR (282 MHz, $CDCl_3$) δ_F: –61.0 (CF_3); ^{13}C NMR (126 MHz, $CDCl_3$) δ_C: 79.2 (*enamino*-C*H*), 110.5 (C*H*-azaAr), 113.9 (C*H*-azaAr), 122.2 (*C*H-azaAr), 122.6 (*C*H-azaAr), 123.2 (*C*F_3), 125.3 (2×CH-Ar_{CF3}), 125.3 (q, $^3J_{CF}$ = 3.8, C_{Ar}-CF_3), 126.5 (2×CH-Ar_{CF3}), 129.4 (*C*-azaAr), 129.5 (q, $^2J_{CF}$ = 32.4, C_{Ar}-CF_3), 129.6 (*C*-azaAr),

142.2 (C-Ar$_{CF3}$), 153.2 (N=*C*(C)-N), 171.0 (*C*=OAr$_F$); *m/z* (NSI$^+$) 305 ([M+H]$^+$, 100%); HRMS (NSI$^+$) $C_{16}H_{12}N_2O_1F_3$ [M+H]$^+$, found 305.0898, requires 305.0896 (+0.6 ppm).

2-(4-Fluoro)phenacylbenzimidazole (S47)

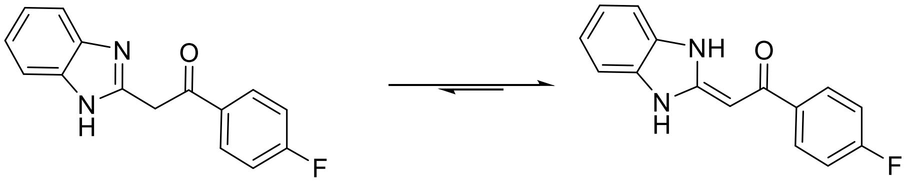

Following the General Procedure 8, 2-methylbenzimidazole (0.66 g, 5.0 mmol), 4-fluorobenzoyl chloride (1.77 mL, 15.0 mmol) and NEt_3 (2.10 mL, 15.0 mmol) in HPLC grade acetonitrile (5 mL) and subsequent hydrolysis in 1-BuOH:DMF (8 mL) afforded the title compound after filtration as a yellow solid as a 4:1 mixture of enamino:keto tautomers (1.708 g, 69%). mp 204–205 °C; ν_{max} (film) 3057 (N-H), 2758 (C-H), 1616 (C=O), 1491 (C$_{Ar}$-C$_{Ar}$); ^{1}H NMR (500 MHz, DMSO-d$_6$) δ_H: 4.67 (2H, s, *keto*-C*H*$_2$CO), 5.99 (1H, s, *enamino*-C*H*CO), 7.14–7.17 (2H, m, *enamino/keto*-benzimidazoleC(5)*H* and benzimidazoleC(6)*H*), 7.25–7.29 (2H, *enamino*-H-Ar$_F$), 7.36–7.38 (1H, m, benzimidazoleC(4)*H*), 7.40–7.42 (2H, *keto*-H-Ar$_F$), 7.54–7.55 (1H, m, *enamino*-benzimidazoleC(7)*H*), 7.89–7.92 (2H, *enamino*-H-Ar$_F$), 8.16–8.19 (2H, m, *keto*-H-Ar$_F$), 12.21 (1H, s, *enamino*-N*H*), 12.33 (1H, s, *keto*-N*H*); ^{19}F NMR (282 MHz, CDCl$_3$) δ_F: –111.8 (*enamino*), –105.3 (*keto*); ^{13}C NMR (126 MHz, CDCl$_3$) δ_C: 39.2 (*C*H$_2$), 78.5 (*enamino*-C*H*), 110.4 (C*H*-azaAr), 113.9 (C*H*-azaAr), 115.1 (C*H*-Ar$_F$), 115.3 (C*H*-Ar$_F$), 116.0 (d, $^2J_{CF}$ = 21.5, C$_{Ph}$-F), 122.0 (*C*H-azaAr), 122.3 (*C*H-azaAr), 128.0 (C*H*-Ar$_F$), 128.1 (C*H*-Ar$_F$), 131.6 (d, $^3J_{CF}$ = 9.8, C$_{Ph}$-F), 134.5 (*C*-azaAr), 134.6 (d, $^4J_{CF}$ = 2.8, C$_{Ph}$-F), 134.6 (*C*-azaAr), 153.5 (N=*C*(C)-N), 162.0 (NH-*C*-NH), 163.0 (d, $^1J_{CF}$ = 246.3, C$_{Ph}$-F), 164.0 (*C*-F), 170.8 (*C*=OAr$_F$); *m/z* (NSI$^+$) 255 ([M+H]$^+$, 100%); HRMS (NSI$^+$) $C_{15}H_{12}F_1N_2O_1$ [M+H]$^+$, found 255.0930, requires 255.0928 (+0.7 ppm).

2-(4-Bromo)phenacylbenzimidazole (S48)

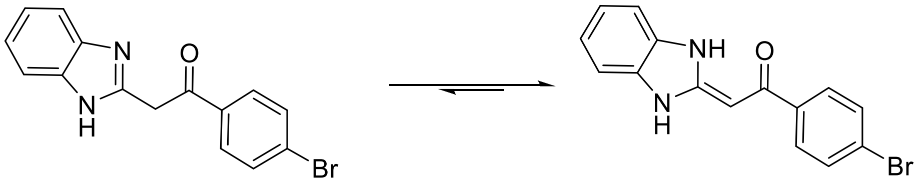

Following the General Procedure 8, 2-methylbenzimidazole (0.66 g, 5.0 mmol), 4-bromobenzoyl chloride (3.29 g, 15.0 mmol) and NEt_3 (2.10 mL, 15.0 mmol) in HPLC grade acetonitrile (5 mL) and subsequent hydrolysis in 1-BuOH:DMF (8 mL) afforded the title compound after filtration as a yellow solid as a 8:1 mixture of enamino:keto tautomers (864 mg, 55%). mp 238–240 °C {Lit.[10] 244–246 °C}; ^{1}H NMR (500

MHz, DMSO-d_6) δ_H: 4.66 (2H, s, *keto*-CH_2CO), 6.03 (1H, s, *enamino*-C*H*CO), 7.15–7.18 (2H, m, benzimidazoleC(5)*H* and benzimidazoleC(6)*H*), 7.35–7.39 (1H, m, *enamino*-benzimidazoleC(4)*H*), 7.54–7.58 (1H, m, *keto*-benzimidazoleC(4)*H*), 7.53 (2H, dd, *J* 5.8, 3.2, *enamino/keto*-benzimidazoleC(7)*H*), 7.63–7.66 (2H, m, *enamino*-H-Ar_{Br}), 7.75–7.77 (2H, m, *J* 8.9, *keto*-H-Ar_{Br}), 7.79–7.81 (2H, m, *enamino*-H-Ar_{Br}), 8.00–8.02 (2H, m, *keto*-H-Ar_{Br}), 12.25 (1H, s, *enamino*-N*H*), 12.34 (1H, s, *keto*-N*H*). Data in agreement with the literature.[10]

2-(3-Methyl)phenacylbenzimidazole (S49)

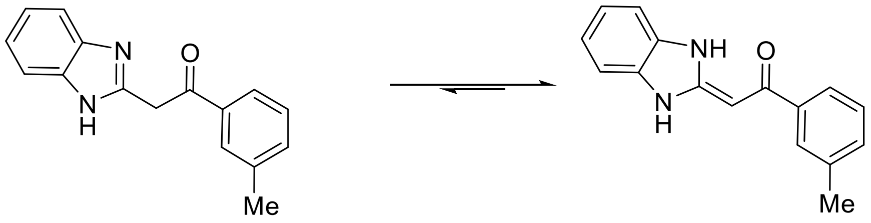

Following the General Procedure 8, 2-methylbenzimidazole (0.66 g, 5.0 mmol), 3-methylbenzoyl chloride (1.98 mL, 15.0 mmol) and NEt_3 (2.10 mL, 15.0 mmol) in HPLC grade acetonitrile (5 mL) and subsequent hydrolysis with morpholine (1.7 mL) afforded the title compound after filtration as a yellow solid as a 3.5:1 mixture of enamino:keto tautomers (0.744 g, 59%). mp 142–143 °C; ν_{max} (film) 3053 (N-H), 2778 (C-H), 1684 (C=O), 1506 (C_{Ar}-C_{Ar}); ^{1}H NMR (500 MHz, DMSO-d_6) δ_H: 2.38 (3H, s, *enamino*-CH_3), 2.39 (3H, s, *keto*-CH_3), 4.66 (2H, s, *keto*-CH_2CO), 6.09 (1H, s, *enamino*-C*H*CO), 7.15–7.17 (2H, m, *enamino/keto*-benzimidazoleC(5)*H* and benzimidazoleC(6)*H*), 7.27 (2H, d, *J* 8.1, *enamino*-H-Ar_{Me}), 7.37 (2H, d, *J* 8.1, *keto*-H-Ar_{Me}), 7.46–7.52 (1H, m, *enamino/keto*-benzimidazoleC(4) (7)*H*), 7.98 (2H, d, *J* 8.1, *keto*-H-Ar_{Me}), 12.32 (1H, s, *enamino*-N*H*); ^{13}C NMR (126 MHz, $CDCl_3$) δ_C: 21.0 (*enamino*-*C*H_3), 21.2 (*keto*-*C*H_3), 46.1 (*C*H_2), 80.0 (*enamino*-*C*H), 122.0 (*C*H-azaAr), 125.5 (*C*H-Ar_{Me}), 128.6 (*C*H-azaAr), 129.0 (*C*H-Ar_{Me}), 129.4 (*C*H-azaAr), 133.5 (C-Ar_{Me}), 134.3 (*C*-azaAr), 139.4 (*C*-azaAr), 144.2 (*C*-Me), 149.0 (N=*C*-N), 153.7 (NH-*C*-NH), 169.1 (*enamino*-*C*=OAr_{Me}), 194.7 (*keto*-*C*=OAr_{Me}); *m/z* (NSI^+) 251 ($[M+H]^+$, 100%); HRMS (NSI^+) $C_{16}H_{15}N_2O_1$ $[M+H]^+$, found 251.1181, requires 251.1179 (+0.8 ppm).

2-(Furan-2-yl)phenacylbenzimidazole (S50)

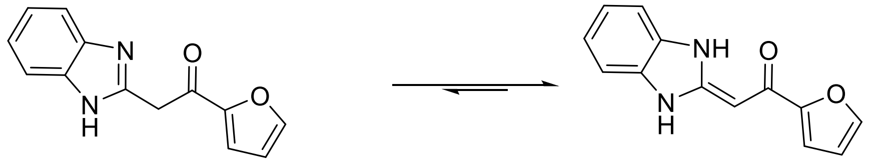

Following the General Procedure 8, 2-methylbenzimidazole (0.66 g, 5.0 mmol), 2-furoyl chloride (1.60 mL, 16.5 mmol) and NEt_3 (2.30 mL, 16.5 mmol) in HPLC grade acetonitrile (5 mL) and subsequent hydrolysis with morpholine (1.7 mL) afforded the title compound after filtration as a yellow solid as a 3:1 mixture of enamino:keto tautomers (0.306 g, 27%). mp 182–183 °C; ν_{max} (film) 3053 (N-H), 2585 (C-H), 1614 (C=O), 1574 (C_{Ar}-C_{Ar}); ^{1}H NMR (500 MHz, DMSO-d_6) δ_H: 4.46 (2H, s, *keto*-CH_2CO), 5.78 (1H, s, *enamino*-C*H*CO), 6.55 (1H, dd, *J* 3.4, 1.7, *enamino*-H–Fur), 6.77 (1H, dd, *J* 3.6, 1.8, *keto*-H–Fur), 6.87 (1H, dd, *J* 3.4, 0.7, *enamino*-H–Fur), 7.12–7.16 (2H, m, *enamino/keto*-benzimidazoleC(5)*H* and benzimidazoleC(6)*H*), 7.46–7.54 (2H, m, *enamino/keto*-benzimidazoleC(4)(7)*H*), 7.65 (1H, d, *J* 3.3, *keto*-H-Fur), 7.74–7.75 (1H, m, *enamino*-H–Fur), 8.06 (1H, d, *J* 2.1, *keto*-H-Fur), 12.13 (1H, s, *keto*-N*H*); 12.38 (1H, s, *enamino*-N*H*); ^{13}C NMR (126 MHz, $CDCl_3$) δ_C: 39.1 (*C*H_2), 76.1 (*enamino*-*C*H), 109.9 (*enamino*-*C*H-Fur), 111.8 (*enamino*-*C*H-Fur), 113.1 (*C*H-azaAr), 120.0 (*C*H-azaAr), 122.0 (*C*H-azaAr), 122.3 (*C*H-azaAr), 131.0 (*C*-azaAr), 134.0 (*C*-azaAr), 148.3 (N=*C*-N), 153.1 (*enamino*-*C*H-Fur), 166.1 (NH-*C*-NH), 183.0 (*C*=OFur); *m/z* (NSI$^+$) 227 ([M+H]$^+$, 100%; 249 [M+Na]$^+$, 30%); HRMS (NSI$^+$) $C_{13}H_{11}N_2O_2$ [M+H]$^+$, found 227.0815, requires 227.0815 (–0.0 ppm).

2-Phenacyl-(5-bromo)benzimidazole (S51)

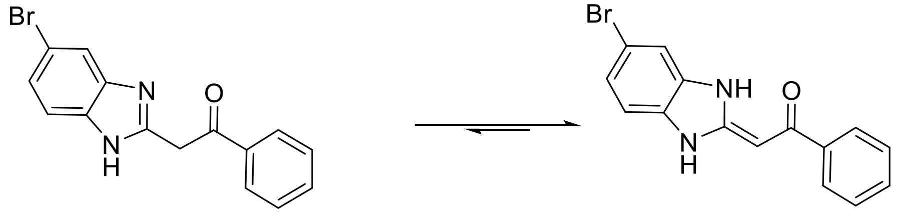

Following the General Procedure 8, 2-methyl-(5-bromo)benzimidazole (0.53 g, 3.0 mmol), benzoyl chloride (1.05 mL, 9.0 mmol) and NEt_3 (1.26 mL, 9.0 mmol) in HPLC grade acetonitrile (4 mL) and subsequent hydrolysis with morpholine (1.7 mL) afforded the title compound after filtration as a yellow solid as a 2.9:1 mixture of enamino:keto tautomers (165 mg, 21%). mp 171–172 °C; ν_{max} (film) 3088 (N-H), 2675 (C-H), 1593 (C=O), 1396 (C_{Ar}-C_{Ar}); ^{1}H NMR (500 MHz, DMSO-d_6) δ_H: 4.70 (2H, s, *keto*-CH_2CO), 6.15 (1H, s, *enamino*-C*H*CO), 7.31 (1H, dd, *J* 8.4, 2.0, *enamino*-benzimidazoleC(7)*H*), 7.40 (1H, dd, *J* 6.7, 3.3, *keto*-benzimidazoleC(6)*H*), 7.45–7.49 (4H, m, 3×H-Ph and *enamino*-benzimidazoleC(4)*H*), 7.57 (1H, t, *J* 7.7, *enamino*-benzimidazoleC(7)*H*), 7.69 (1H, t, *J* 7.4, *keto*-

benzimidazoleC(4)*H*), 7.74–7.76 (1H, m, *keto*-benzimidazoleC(7)*H*), 7.85–7.86 (2H, m, *enamino*-H-Ph), 8.07–8.09 (2H, m, *keto*-H-Ph), 12.49 (1H, s, *keto*-N*H*), 12.55 (1H, s, *enamino*-N*H*); ^{13}C NMR (126 MHz, $CDCl_3$) δ_C: 39.6 (*C*H_2), 81.5 (*enamino*-*C*H), 114.0 (*enamino*-*C*H-azaAr), 124.8 (*enamino*-*C*H-azaAr), 125.5 (*keto*-*C*-Cl), 125.6 (2×*enamino*-*C*H-Ph), 128.4 (*enamino*-*C*-Cl), 128.5 (2×*keto*-*C*H-Ph), 128.6 (3×*enamino*-*C*H-Ph), 128.9 (2×*keto*-*C*H-Ph), 130.0 (*keto*-*C*H-Ph), 131.0 (*keto*-*C*-azaAr), 133.0 (*keto*-*C*-azaAr), 133.8 (*keto*-*C*H-azaAr), 135.9 (*keto*-*C*-Ph), 136.3 (*enamino*-*C*-Ph), 154.5 (N=*C*-N), 168.4 (*enamino*-*C*=OAr$_{Me}$), 195.0 (*keto*-*C*=OAr$_{Me}$); *m/z* (NSI$^+$) 315 ([M+H]$^+$, 100%); HRMS (NSI$^+$) $C_{15}H_{12}Br_1N_2O_1$ [M+H]$^+$, found 315.0132, requires 315.0128 (+1.4 ppm).

2-(4-Methoxy)phenacyl-(5-chloro)benzimidazole (S52)

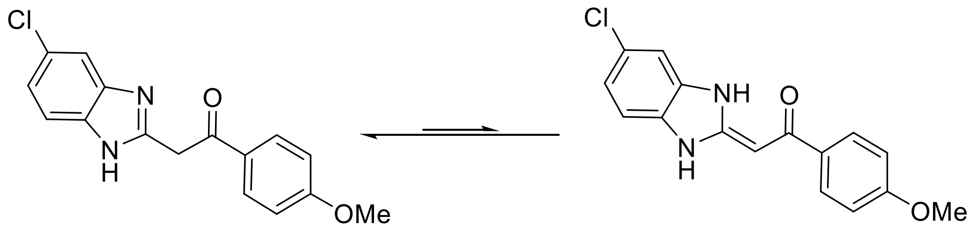

Following the General Procedure 8, 2-methyl-(5-chloro)benzimidazole (0.50 g, 3.0 mmol), 4-methoxybenzoyl chloride (1.54 g, 9.0 mmol) and NEt_3 (1.26 mL, 9.0 mmol) in HPLC grade acetonitrile (4 mL) and subsequent hydrolysis with morpholine (1.7 mL) afforded the title compound after filtration as a yellow solid as a 1:1.4 mixture of enamino:keto tautomers (520 mg, 58%). mp 159–160 °C; ν_{max} (film) 3302 (N-H), 2691 (C-H), 1591 (C=O), 1495 (C_{Ar}-C_{Ar}); ^{1}H NMR (500 MHz, DMSO-d$_6$) δ_H: 3.81 (3H, s, *enamino*-OC*H*$_3$), 3.85 (3H, s, *keto*-OC*H*$_3$), 4.62 (2H, s, *keto*-C*H*$_2$CO), 6.06 (1H, s, *enamino*-C*H*CO), 7.02 (2H, d, *J* 8.9, *enamino*-C*H*-Ar$_{OMe}$), 7.08 (2H, d, *J* 8.9, *keto*-C*H*-Ar$_{OMe}$), 7.15–7.18 (2H, m, *enamino/keto*-benzimidazoleC(7)*H*), 7.38–7.49 (2H, m, *enamino*-benzimidazoleC(4)(6)*H*), 7.53–7.61 (2H, m, *keto*-benzimidazoleC(4)(6)*H*), 7.81 (2H, d, *J* 8.9, *enamino*-C*H*-Ar$_{OMe}$), 8.06 (2H, d, *J* 8.9, *keto*-C*H*-Ar$_{OMe}$), 12.37 (1H, s, *keto*-N*H*), 12.49 (1H, s, *enamino*-N*H*), 12.54 (1H, s, *enamino*-N*H*); ^{13}C NMR (126 MHz, $CDCl_3$) δ_C: 39.3 (*C*H_2), 55.3 (*enamino*-O*C*H_3), 55.6 (*keto*-O*C*H_3), 80.4 (*enamino*-*C*H), 110.3 (*keto*-*C*H-azaAr), 111.6 (*enamino*-*C*H-azaAr), 113.9 (*enamino*-*C*H-Ar$_{OMe}$), 114.1 (*enamino*-*C*H-Ar$_{OMe}$), 114.6 (*enamino*-*C*H-azaAr), 116.1 (*keto*-*C*H-azaAr), 121.9 (*keto*-*C*H-azaAr), 126.1 (*C*-Ar$_{OMe}$), 128.6 (*enamino*-*C*-Cl), 128.9 (*keto*-*C*-Cl), 132.3 (*keto*-*C*H-azaAr), 132.8 (*keto*-*C*-azaAr), 133.0 (*keto*-*C*-azaAr), 137.0 (*enamino*-*C*-azaAr), 139.1 (*enamino*-*C*-azaAr), 155.1 (N=*C*-N), 160.8 (*enamino*-*C*-OMe), 163.5 (*keto*-*C*-OMe) 167.8 (NH-*C*-NH), 167.9 (*enamino*-*C*=OAr$_{OMe}$), 193.3 (*keto*-*C*=OAr$_{OMe}$); *m/z* (NSI$^+$) 301 ([M+H]$^+$, 100%); HRMS (NSI$^+$) $C_{16}H_{14}Cl_1N_2O_2$ [M+H]$^+$, found 301.0741, requires 301.0738 (+0.9 ppm).

2-Phenacyl-(5,6-dimethyl)benzimidazole (S53)

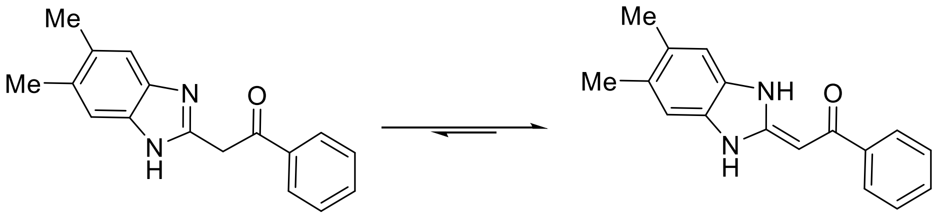

Following the General Procedure 8, 2-methyl-(5,6-dimethyl)benzimidazole (0.48 g, 3.0 mmol), benzoyl chloride (1.05 mL, 9.0 mmol) and NEt_3 (1.26 mL, 9.0 mmol) in HPLC grade acetonitrile (4 mL) and subsequent hydrolysis with morpholine (1.7 mL) afforded the title compound after filtration as a yellow solid as a 5:1 mixture of enamino:keto tautomers (622 mg, 78%). mp 190–191 °C; ν_{max} (film) 3034 (N-H), 2590 (C-H), 1575 (C=O), 1396 (C_{Ar}-C_{Ar}); ^{1}H NMR (500 MHz, DMSO-d_6) δ_H: 2.29 (6H, 2×CH_3), 4.67 (2H, s, *keto*-C*H*$_2$CO), 6.02 (1H, s, *enamino*-C*H*CO), 7.25 (2H, s, *enamino*-benzimidazoleC(4)(7)*H*), 7.30 (2H, s, *keto*-benzimidazoleC(4)(7)*H*), 7.40–7.47 (3H, m, *enamino*-*H*-Ph), 7.55–7.58 (3H, m, *keto*-*H*-Ph), 7.81–7.84 (2H, m, *enamino*-H-Ph), 8.07–8.08 (2H, m, *keto*-H-Ph), 12.18 (1H, s, *enamino*-N*H*); ^{13}C NMR (126 MHz, $CDCl_3$) δ_C: 19.9 (*enamino*-CH_3), 20.0 (*keto*-CH_3), 80.0 (*enamino*-*C*H), 125.5 (2×*C*H-Ph), 128.4 (2×*C*H-Ph), 128.5 (*C*H-azaAr), 128.9 (*C*H-azaAr), 129.6 (*C*H-Ph), 130.5 (2×*C*-CH_3), 137.5 (*C*-Ph), 152.9 (N=*C*-N), 169.4 (NH-*C*-NH), 169.4 (*C*=OPh); *m/z* (NSI^+) 271 ($[M+H]^+$, 100%); HRMS (NSI^+) $C_{17}H_{17}N_2O_1$ $[M+H]^+$, found 265.1336, requires 265.1335 (+0.2 ppm).

4 Michael-Michael-Lactonization with 1,3-Dicarbonyls

4.1 Reaction optimization

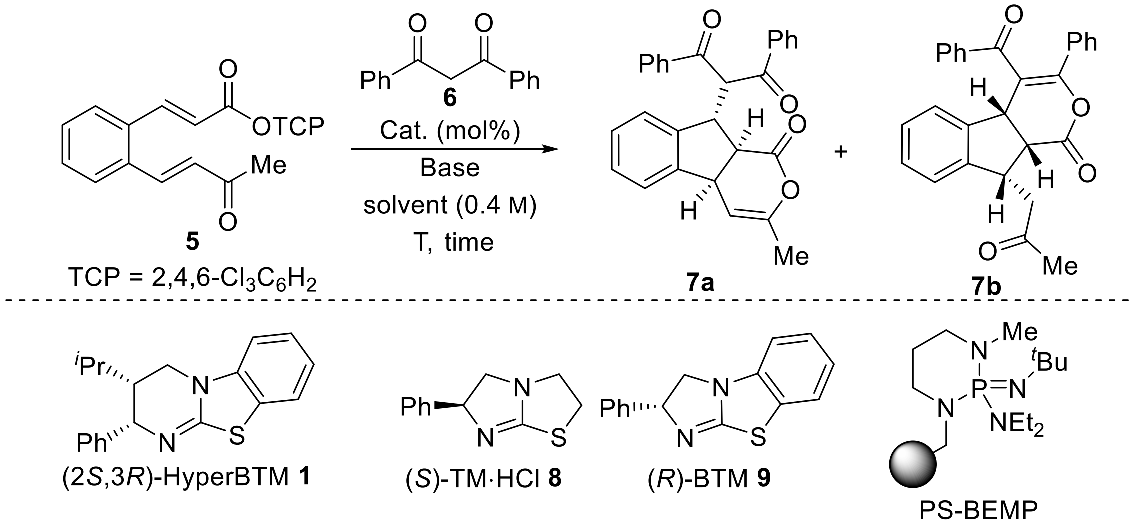

Entry[a]	Catalyst (mol%)	Base	**5**:**6**:base (equiv)	Solvent (Time, h)	T (°C)	Yield (%)[b]	**7a**:**7b**[c]	er of **7a**[d]
**Base Screening**								
1	**1** (20)	$^{i}Pr_2NEt$	1:1:1	CH_2Cl_2 (48)	rt	trace	-	-
2	**1** (20)	K_2CO_3	1:1:1	CH_2Cl_2 (48)	rt	-	-	-
3	**1** (20)	DBU	1:1:1	CH_2Cl_2 (48)	rt	decomposition of **5**	-	-
4	**1** (20)	PS-BEMP	1:1:1	CH_2Cl_2 (48)	rt	46	75:25	
**Catalyst Screening**								
5	**8** (20)	PS-BEMP	1:1:1	CH_2Cl_2 (48)	rt	trace	-	-
6	**9** (20)	PS-BEMP	1:1:1	CH_2Cl_2 (48)	rt	trace	-	-
**Solvent Screening**								
7	**1** (20)	PS-BEMP	1:1:1	THF (48)	rt	21	75:25	>99:1
8	**1** (20)	PS-BEMP	1:1:1	1,4-Dioxane (48)	rt	20	71:29	>99:1

9	**1** (20)	PS-BEMP	1:1:1	$C_2H_4Cl_2$ (48)	rt	35	80:20	97.5:2.5
10	**1** (20)	PS-BEMP	1:1:1	PhMe (48)	rt	15	83:17	98.5:1.5
11	**1** (20)	PS-BEMP	1:1:1	$CHCl_3$ (48)	rt	47	78:22	99:1
12	**1** (20)	PS-BEMP	1:1:1	MeCN (48)	rt	62	86:14	96.5:3.5
**Reaction Stoichiometry/Solvent**								
13	**1** (20)	PS-BEMP	1:3:3	MeCN (16)	rt	71	89:11	95.5:4.5
14	**1** (20)	PS-BEMP	1:2:2	MeCN (48)	rt	70	91:9	95.5:4.5
15	**1** (20)	PS-BEMP	2:1:1	MeCN (48)	rt	trace	-	-
16	**1** (20)	PS-BEMP	1:2:2	$CHCl_3$ (48)	rt	55	71:29	98.5:1.5
17	**1** (20)	PS-BEMP	1:2:2	THF (48)	rt	60	>95:5	>99:1
**Temperature/Solvent Effect**								
18	**1** (20)	PS-BEMP	1:2:2	MeCN (>48)	0	26	50:50	98.5:1.5
19	**1** (20)	PS-BEMP	1:2:2	MeCN (16)	50	64	93:7	93.5:6.5
20	**1** (20)	PS-BEMP	1:2:2	THF (16)	50	49	91:9	96.5:3.5
21	**1** (20)	PS-BEMP	1:2:2	$CHCl_3$ (16)	50	50	75:25	97:3
22	**1** (20)	PS-BEMP	1:2:2	THF (24/16)[e]	rt/50	58	>95:5	>99:1
**Catalyst loading**								
23	**1** (10)	PS-BEMP	1:2:2	THF (48)	rt	53	>95:5	98.5:1.5
24	**1** (5)	PS-BEMP	1:2:2	THF (>48)	rt	48	95:5	97:3

[a] Reactions performed on 0.1 mmol scale. [b] Combined yield of **7a** and **7b**. [c] Determined by ^1H NMR spectroscopic analysis of the crude reaction product. [d] Determined by HPLC analysis for the major product. [e] 24 h at rt until the full conversion of **5**, then heating at 50 °C for 16 h.

4.2 Data for indanes 7, 10-28

2-((4a*S*,9*S*,9a*R*)-3-Methyl-1-oxo-1,4a,9,9a-tetrahydroindeno[2,1-*c*]pyran-9-yl)-1,3-diphenylpropane-1,3-dione (7a)

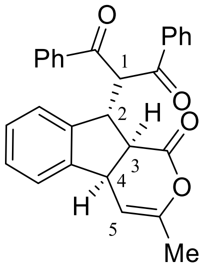

Following General Procedure 9, the corresponding TCP-ester (39.5 mg, 0.1 mmol), 1,3-dipheny-1,3-propanedione (44.8 mg, 0.2 mmol) and PS-BEMP (2.0 mmol/g loading, 90.0 mg, 0.2 mmol) in THF (0.25 mL) and subsequent chromatography (80:20 Petrol : EtOAc, R_f 0.2) afforded the title compound (25.4 mg, 60%) as a white solid. mp 79–81 °C; $[\alpha]_D^{20}$ +16.7 (*c* 1.0, $CHCl_3$); Chiral HPLC analysis, Chiralpak IB (97:3 hexane : IPA, flow rate 1.5 mLmin^{-1}, 211 nm, 40 °C) t_R major: 18.2 min, t_R minor: 29.0 min, >99:1 er; ν_{max} (film) 2922 (C-H), 1751 (C=O), 1694 (C=O), 1667 (C=O); ^{1}H NMR (500 MHz, $CDCl_3$) δ_H: 1.87 (3H, s, COCH_3), 3.45 (1H, dd, *J* 9.0, 4.2, H–3), 4.07–4.12 (1H, m, H–4), 4.81–4.86 (1H, m, H–2), 5.17 (1H,d, *J* 4.9, H–5),5.72 (1H, d, *J* 6.1, H–1), 7.10–7.22 (4H, m, H–Ar), 7.37–7.43 (4H, m, H–Ph), 7.51–7.55 (2H, m, H–Ph), 7.89–7.91 (4H, m, H–Ph); ^{13}C NMR (126 MHz, $CDCl_3$) δ_C: 19.1 (CO-CH_3), 40.4 (CH-2), 45.5 (CH-4), 48.5 (CH-3), 59.4 (CH-1), 99.9 (CH-5), 123.7 (CH–Ar), 125.7 (CH-Ar), 127.5 (CH-Ph1), 128.2, (CH-Ar), 128.8 (2×CH-Ph2), 128.9 (2×CH-Ph2), 129.0 (2×CH-Ph1, Ar), 129.0 (2×CH-Ph1), 133.8 (CH-Ph2), 133.9 (CH-Ph1), 136.0 (CH-C=OO*C*), 136.7 (C-Ph2), 140.9 (C-Ph1), 143.7 (C1-Ar), 148.1 (C6-Ar), 170.0 (CH-*C*=OO), 195.0 (*C*=OPh2), 195.6 (*C*=OPh1); *m/z* (NSI$^+$) 867 ([2M+Na]$^+$, 100%); HRMS (NSI$^+$) $C_{28}H_{23}O_4$ [M+H]$^+$, found 423.1587, requires 423.1591 (–0.9 ppm).

(4a*R*,9*S*,9a*R*)-4-Benzoyl-9-(2-oxopropyl)-3-phenyl-9,9a-dihydroindeno[2,1-*c*]pyran-1(4aH)-one (7b)

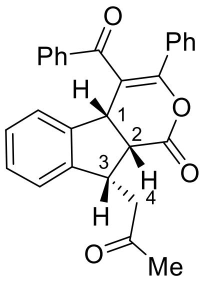

The title compound was observed as a minor product in the inseparable product mixture (r.r. 2:1) under different reaction conditions than described in General Procedure 9 (24 h, CH_2Cl_2 as a reaction solvent); Chiral HPLC analysis, Chiralpak IB (97:3 hexane : IPA, flow rate 1.5 mLmin^{-1}, 211 nm, 40 °C) t_R major: 15.2 min, t_R minor: 20.1 min, >99:1 er; δ_H: 2.35 (3H, s, COC*H*$_3$), 3.23 (1H, dd, *J* 18.0, 5.9, H–4a), 3.60 (1H, dd, *J* 18.0, 9.8, H–4b), 3.95 (1H, t, *J* 7.3, H–2), 4.03–4.07 (1H, m, H–3), 4.74 (1H, d, *J* 7.9, H–1), 7.10–7.22 (4H, m, H–Ar), 7.37–7.43 (4H, m, H–Ph), 7.51–7.55 (2H, m, H–Ph), 7.71–7.73 (4H, m, H–Ph).

1,3-Bis(4-methoxyphenyl)-2-((4a*R*,9*R*,9a*S*)-3-methyl-1-oxo-1,4a,9,9a-tetrahydroindeno[2,1-*c*]pyran-9-yl)propane-1,3-dione (10a)

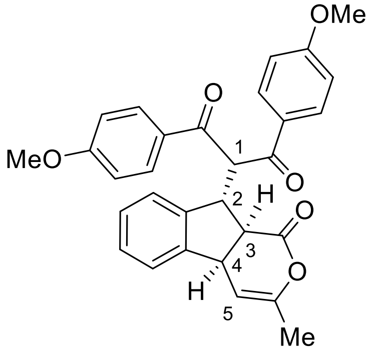

Following General Procedure 9, the corresponding TCP-ester (39.5 mg, 0.1 mmol), 1,3-bis(4-methoxyphenyl)propane-1,3-dione (56.8 mg, 0.2 mmol) and PS-BEMP (2.0 mmol/g loading, 90.0 mg, 0.2 mmol) in THF (0.25 mL) and subsequent chromatography (98:2 CH_2Cl_2 : EtOAc, R_f 0.20) afforded the title compound (28.0 mg, 58%) as a colorless foam. mp 86–87 °C; $[\alpha]_D^{20}$+39.0 (*c* 0.5, $CHCl_3$); Chiral HPLC analysis, Chiralpak AS-H (90:10 hexane : IPA, flow rate 1.5 mLmin^{-1}, 220 nm, 40 °C) t_R minor: 18.5 min, t_R major: 28.6 min, 97.5:2.5 er; ν_{max} (film) 3071 (C-H), 1751 (C=O), 1684 (C=O), 1599 (C=O); ^{1}H NMR (500 MHz, $CDCl_3$) δ_H: 1.89 (3H, s, COC*H*$_3$), 3.42 (1H, dd, *J* 8.8, 3.7, H–3), 3.85 (6H, s, 2×*p*-OC*H*$_3$), 4.06–4.15 (1H, m, H–4), 4.90 (1H, dd, *J* 6.2, 4.0, H–2), 5.21 (1H, d, *J* 5.1, H–5), 5.51 (1H, d, *J* 6.7, H–1), 6.84–.6.92 (4H, m, H–Ar), 7.11–7.26 (4H, m, H–Ph), 7.94 (4H, dd, *J* 8.8, 2.4, H–Ph), 7.89–7.91 (4H, m, H–Ph); ^{13}C NMR (126 MHz, $CDCl_3$) δ_C: 19.1 (CO-*C*H$_3$), 40.3 (CH-2), 45.7 (CH-4), 48.3

(CH-3), 55.6 (2×*p*-OCH$_3$) 60.0 (CH-1), 99.8 (CH-5), 114.2 (4×*C*H-Ph1+Ph2), 123.6 (*C*H-Ar), 125.9 (*C*H-Ar), 127.5 (*C*H-Ar), 128.1 (*C*H-Ar), 129.1 (*C*-Ph1), 129.8 (*C*-Ph2), 131.4 (4×*C*H-Ph1+Ph2), 141.5 (CH-C=OO*C*), 141.6 (*C*-Ar), 148.2 (*C*-Ar), 163.9 (*C*-Ph1), 164.1 (*C*-Ph2), 170.0 (CH-*C*=OO), 193.4 (*C*=OPh2), 194.1 (*C*=OPh1); *m/z* (NSI$^+$) 987 ([2M+Na]$^+$, 100%), 505 ([M+Na]$^+$, 95%); HRMS (NSI$^+$) $C_{30}H_{26}O_6Na$ [M+Na]$^+$, found 505.1620, requires 505.1622 (–0.3 ppm).

1,3-Bis(4-fluorophenyl)-2-((4a*R*,9*R*,9a*S*)-3-methyl-1-oxo-1,4a,9,9a-tetrahydroindeno[2,1-*c*]pyran-9-yl)propane-1,3-dione (11a)

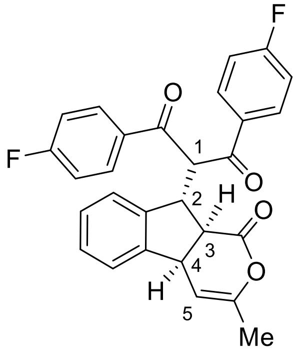

Following General Procedure 9, the corresponding TCP-ester (39.5 mg, 0.1 mmol), 1,3-bis(4-fluorophenyl)propane-1,3-dione (52.0 mg, 0.2 mmol) and PS-BEMP (2.0 mmol/g loading, 90.0 mg, 0.2 mmol) in THF (0.25 mL) and subsequent chromatography (80:20 Petrol : EtOAc, R_f 0.20) afforded the title compound (23.8 mg, 52%) as a light yellow crystalline solid. mp 113–114 °C; $[\alpha]_D^{20}$ +19.2 (*c* 0.5, $CHCl_3$); Chiral HPLC analysis, Chiralpak IB (97:3 hexane : IPA, flow rate 1.5 mLmin^{-1}, 211 nm, 40 °C) t_R major: 16.5 min, t_R minor: 28.2 min, 98.5:1.5 er; ν_{max} (film) 3071 (C-H), 1751 (C=O), 1684 (C=O), 1599 (C=O); ^{1}H NMR (500 MHz, $CDCl_3$) δ_H: 1.87 (3H, s, COC*H*$_3$), 3.39 (1H, dd, *J* 8.9, 4.1, H–3), 4.06–4.11 (1H, m, H–4), 4.84 (1H, dd, *J* 6.0, 4.4, H–2), 5.18 (1H, d, *J* 5.0, H–5), 5.56 (1H, d, *J* 6.3, H–1), 7.05–7.11 (4H, m, H–Ar), 7.11–7.13 (2H, m, H–PhF), 7.17–7.20 (2H, m, H–PhF), 7.91–7.96 (4H, m, H–PhF); ^{19}F NMR (282 MHz, $CDCl_3$) δ_F: –103.5 (PhF1), –103.2 (PhF2); ^{13}C NMR (126 MHz, $CDCl_3$) δ_C: 19.1 (CO-*C*H$_3$), 40.4 (CH-2), 45.5 (CH-4), 48.4 (CH-2), 59.9 (CH-1), 99.8 (CH-5), 116.2 (2×*C*H-PhF1), 116.3 (d, $^2J_{CF}$ = 27.5, C_{Ar}-F), 116.4 (2×*C*H-PhF2), 123.8 (*C*H-Ar), 125.6 (*C*H-Ar), 127.6 (*C*H-Ar), 128.4 (*C*H-Ar), 131.6 (2×*C*H-PhF1), 131.6 (d, $^3J_{CF}$ = 6.5, C_{Ar}-F), 131.7 (2×*C*H-PhF2), 132.3 (*C*-PhF1), 133.8 (*C*-PhF2), 140.7 (CH-C=OO*C*), 143.6 (*C*-Ar), 148.2 (*C*-Ar), 165.2 (*C*-PhF1), 166.2 (d, $^1J_{CF}$ = 252.0, C_{Ar}-F), 167.2 (*C*-PhF2), 169.9 (CH-*C*=OO), 193.3 (*C*=OPhF2), 193.9 (*C*=OPhF2); *m/z* (NSI$^+$) 939 ([2M+Na]$^+$, 100%), 481 ([M+Na]$^+$, 85%); HRMS (NSI$^+$) $C_{28}H_{20}O_4F_2Na$ [M+Na]$^+$, found 481.1213, requires 481.1222 (–1.8 ppm).

1,3-Di(furan-2-yl)-2-((4a*R*,9*R*,9a*S*)-3-methyl-1-oxo-1,4a,9,9a-tetrahydroindeno[2,1-*c*]pyran-9-yl)propane-1,3-dione (12a)

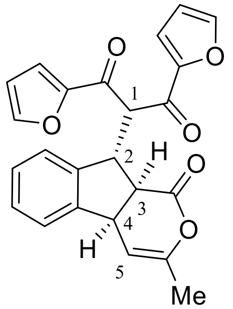

Following General Procedure 9, the corresponding TCP-ester (39.5 mg, 0.1 mmol), 1,3-di(furan-2-yl)propane-1,3-dione (40.8 mg, 0.2 mmol) and PS-BEMP (2.0 mmol/g loading, 90.0 mg, 0.2 mmol) in THF (0.25 mL) and subsequent chromatography (98:2 CH_2Cl_2 : EtOAc, R_f 0.2) afforded the title compound (28.6 mg, 71%) as colorless crystalline solid. mp 186–187 °C; $[\alpha]_D^{20}$ +43.0 (*c* 0.5, $CHCl_3$); Chiral HPLC analysis, Chiralpak AS-H (90:10 hexane : IPA, flow rate 1.5 mLmin^{-1}, 211 nm, 40 °C) t_R minor: 12.1 min, t_R major: 21.8 min, 98:2 er; ν_{max} (film) 3021 (C-H), 1751 (C=O), 1680 (C=O), 1649 (C=O); ^{1}H NMR (500 MHz, $CDCl_3$) δ_H: 1.87 (3H, s, COC*H*$_3$), 3.40 (1H, dd, *J* 8.8, 3.3, H–3), 4.11–4.17 (1H, m, H–4), 4.87 (1H, dd, *J* 7.3, 3.2, H–2), 5.21 (1H, d, *J* 7.2, H–1), 5.23 (1H, d, *J* 4.2, H–5), 6.51 (2H, dd, *J* 8.4, 2.1, H–Fur), 7.07–7.24 (4H, m, H–Ar), 7.29 (1H, d, *J* 3.5, H–Fur), 7.35 (1H, d, *J* 3.5, H–Fur), 7.53 (2H, d, *J* 6.2, H–Fur); ^{13}C NMR (126 MHz, $CDCl_3$) δ_C: 19.1 (CO-*C*H$_3$), 40.3 (CH-2), 45.4 (CH-4), 47.6 (CH-3), 60.6 (CH-1), 99.7 (CH-5), 113.0 (2×*C*H-fur), 119.5 (2×*C*H-fur), 123.6 (*C*H-Ar), 125.9 (*C*H-Ar), 127.5 (*C*H-Ar), 128.3 (*C*H-Ar), 140.9 (CH-C=OO*C*), 143.6 (*C*-Ar), 147.3 (2×*C*-fur), 148.3 (*C*-Ar), 151.8 (*C*H-fur), 152.3 (*C*H-fur), 169.7 (CH-*C*=OO), 182.6 (*C*=O-fur), 183.1 (*C*=O-fur); *m/z* (NSI$^+$) 827 ([2M+Na]$^+$, 100%), 425 ([M+Na]$^+$, 65%); HRMS (NSI$^+$) $C_{24}H_{19}O_6$ [M+H]$^+$, found 403.1172, requires 403.1176 (–1.0 ppm).

3-((4a*R*,9*R*,9a*S*)-3-Methyl-1-oxo-1,4a,9,9a-tetrahydroindeno[2,1-*c*]pyran-9-yl)pentane-2,4-dione (13a)

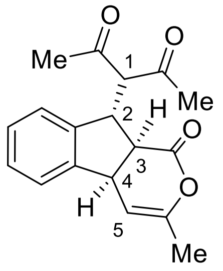

Following General Procedure 9, the corresponding TCP-ester (39.5 mg, 0.1 mmol), pentane-2,4-dione (21 μL mg, 0.2 mmol) and PS-BEMP (2.0 mmol/g loading, 90.0 mg, 0.2 mmol) in THF (0.25 mL) and subsequent chromatography (80:20 Petrol : EtOAc, R_f 0.2) afforded the title compound (22.9 mg, 77%) as a colorless oil; $[\alpha]_D^{20}$ +5.7 (*c* 1.0, $CHCl_3$); Chiral HPLC analysis, Chiralpak AS-H (97:3 hexane : IPA, flowrate 1.0 mLmin^{-1}, 211 nm, 40 °C) t_R major: 11.9 min, t_R minor: 15.9 min, 62.5:37.5 er; ν_{max} (film) 3054 (C-H), 1725 (C=O), 1678 (C=O), 1652 (C=O); ^{1}H NMR (500 MHz, $CDCl_3$) δ_H: 1.89 (3H, s, CH=COCH_3), 2.14 (3H, s, COCH_3), 2.27 (3H, s, COCH_3), 3.04 (1H, dd, *J* 8.8, 2.5, H–3), 3.93 (1H, d, *J* 9.4, H–1), 4.05–4.07 (1H, m, H–4), 4.65 (1H, dd, *J* 9.4, 2.4, H–2), 5.30 (1H, d, *J* 5.1, H–5), 7.14–7.18 (3H, m, H–Ar), 7.23–7.24 (1H, m, H–Ar); ^{13}CNMR (126 MHz, $CDCl_3$) δ_C: 19.1 (CH=CO-*C*H_3), 28.6 (CO*C*H_3), 31.8 (CO*C*H_3), 39.9 (CH–2), 45.3 (CH–4), 47.6 (CH–3), 72.0 (CH–1), 99.4 (CH–5), 123.9 (CH-Ar), 125.3 (CH-Ar), 127.9, (CH-Ar), 128.6 (CH-Ar), 140.6 (CH=*C*O-CH_3), 143.4 (C1-Ar), 148.5 (C6-Ar), 169.7 (CH-*C*=OO), 203.2 (*C*OMe), 203.8 (*C*OMe); *m/z* (NSI$^+$) 321 ([M+Na]$^+$, 30%); HRMS (NSI$^+$) $C_{18}H_{18}O_4Na$ [M+Na]$^+$, found 321.1107, requires 321.1097 (+3.0 ppm).

Dimethyl 2-((4a*S*,9*R*,9a*R*)-3-methyl-1-oxo-1,4a,9,9a-tetrahydroindeno[2,1-*c*]pyran-9-yl)malonate (14a)

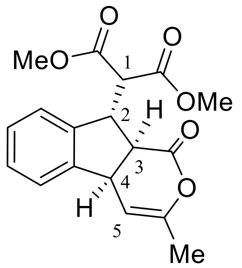

Following General Procedure 9, the corresponding TCP-ester (39.5 mg, 0.1 mmol), dimethylyl malonate (23 μL, 0.2 mmol) and PS-BEMP (2.0 mmol/g loading, 90.0 mg, 0.2 mmol) in THF (0.25 mL) and subsequent chromatography (80 : 20 Petrol : EtOAc, R_f 0.2) afforded the title compound (33 mg, 58%) as a colorless oil. $[\alpha]_D$ +3.0 (*c* 1.0, $CHCl_3$); Chiral HPLC analysis, Chiralpak OJ-H (97:3 hexane : IPA,

flowrate 1.0 mLmin^{-1}, 220 nm, 40 °C) t_R minor: 28.7 min, t_R major: 33.5 min, 73:27 er; ν_{max} (film) 3068 (C-H), 1733 (C=O), 1687 (C=O), 1663 (C=O); ^{1}H NMR (500 MHz, $CDCl_3$) δ_H: 1.88 (3H, s, COCH_3), 3.58 (1H, dd, *J* 9.0, 5.3, H–3), 3.70 (3H, s, COOCH_3), 3.73 (3H, s, COOCH_3), 3.88 (1H, d, *J* 6.3, H–1), 4.08–4.15 (1H, m, H–4), 4.42 (1H, t, *J* 5.8, H–2), 5.11 (1H, dd, *J* 4.4, 1.0, H–5), 7.17–7.22 (2H, m, H–Ar), 7.22–7.26 (2H, m, H–Ar); ^{13}C NMR (126 MHz, $CDCl_3$) δ_C: 19.0 (CO-*C*H_3), 40.6 (CH-*C*H-CHCOO), 45.2 (*C*H-CH=CO), 47.9 (*C*H-C=OO), 52.8 (CO_2*C*H_3), 52.9 (CO_2*C*H_3), 54.2 (MeO_2C*C*HCO_2Me), 100.1 (*C*H=COC=O), 123.8 (CH-Ar), 125.1 (CH-Ar), 127.7, (CH-Ar), 128.6 (CH-Ar), 139.9 (CH-C=OO*C*), 143.7 (C1-Ar), 147.9 (C6-Ar), 168.2 (*C*O_2Me), 168.7 (*C*O_2Me), 169.8 (CH-*C*=OO); HRMS (NSI$^+$) $C_{18}H_{19}O_6$ [M+H]$^+$, found 331.1176, requires 331.1176 (–0.0 ppm).

Diisopropyl 2-((4a*R*,9*S*,9a*S*)-3-methyl-1-oxo-1,4a,9,9a-tetrahydroindeno[2,1-*c*]pyran-9-yl)malonate (15a)

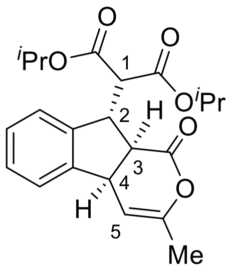

Following General Procedure 9, the corresponding TCP-ester (39.5 mg, 0.1 mmol), diisopropyl malonate (38 μL, 0.2 mmol) and PS-BEMP (2.0 mmol/g loading, 90.0 mg, 0.2 mmol) in THF (0.25 mL) and subsequent chromatography (90:10 Petrol : EtOAc, R_f 0.3) afforded the title compound (20.1 mg, 52%) as a colorless oil; $[\alpha]_D^{20}$ +28.0 (*c* 0.5, $CHCl_3$); Chiral HPLC analysis, Chiralpak OJ-H (98:2 hexane : IPA, flowrate 0.5 mLmin^{-1}, 211 nm, 40 °C) t_R minor: 15.3 min, t_R major: 16.3 min, 82:18 er; ν_{max} (film) 2982 (C-H), 1748 (C=O), 1724 (C=O); ^{1}H NMR (500 MHz, $CDCl_3$) δ_H: 1.09 (3H, d, *J* 6.3, ^{i}Pr-CH_3), 1.15 (3H, d, *J* 6.3, ^{i}Pr-CH_3), 1.18 (3H, d, *J* 6.3, ^{i}PrCH-CH_3), 1.24 (3H, d, *J* 6.3, ^{i}PrCH-CH_3), 1.88 (3H, s, COCH_3), 3.61 (1H, dd, *J* 9.0, 5.4, H–3), 3.80 (1H, d, *J* 5.6, H–1), 4.11 (1H, dd, *J* 8.3, 2.2, H–4), 4.40 (1H, t, *J* 5.5, H–2), 4.97 (1H, sept, *J* 6.2, ^{i}Pr-C*H*), 5.04 (1H, sept, *J* 6.2, ^{i}Pr-C*H*), 5.10 (1H, d, *J* 4.2, H–5), 7.16–7.24 (3H, m, H–Ar), 7.35 (1H, d, *J* 7.6, H–Ar); ^{13}C NMR (126 MHz, $CDCl_3$) δ_C: 19.0 (CO-*C*H_3), 21.6 (2×^{i}PrCH-*C*H_3), 21.7 (2×^{i}PrCH-*C*H_3), 40.6 (CH-2), 45.4 (CH-4), 47.7 (CH-3), 54.8 (CH-1), 69.4 (^{i}Pr-*C*H), 69.5 (^{i}Pr-*C*H), 100.2 (CH-5), 123.6 (*C*H-Ar), 125.7 (*C*H-Ar), 127.5, (*C*H-Ar), 128.4 (*C*H-Ar), 140.1 (CH-C=OO*C*), 143.8 (*C*1-Ar), 147.8 (*C*6-Ar), 167.4 (*C*O_2^{i}Pr), 168.2 (*C*O_2^{i}Pr), 169.9 (CH-*C*=OO); *m/z* (NSI$^+$) 409 ([M+Na]$^+$, 100%); HRMS (NSI$^+$) $C_{22}H_{26}O_6Na$[M+Na]$^+$, found 409.1624, requires 409.1622 (+0.6 ppm).

Ethyl 2-((4a*R*,9*S*,9a*S*)-3-methyl-1-oxo-1,4a,9,9a-tetrahydroindeno[2,1-*c*]pyran-9-yl)-3-oxo-3-phenylpropanoate (±16a)

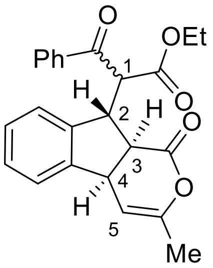

Following General Procedure 9, the corresponding TCP-ester (39.5 mg, 0.1 mmol), ethyl benzoylacetate (43 μL, 0.2 mmol) and PS-BEMP (2.0 mmol/g loading, 90.0 mg, 0.2 mmol) in THF (0.25 mL)and subsequent chromatography (80:20 Petrol : EtOAc, R_f 0.2) afforded the title compound as inseparable diastereomeric mixture (29.4 mg, 74%, dr 75:25) as a colorless oil; ν_{max} (film) 2945 (C-H), 1743 (C=O), 1687 (C=O), 1671 (C=O);

16a (major): ^{1}H NMR (500 MHz, $CDCl_3$) δ_H: 1.00 (3H, t, *J* 7.1, OCH_2CH_3), 1.87 (3H, s, $COCH_3$), 3.60 (1H, dd, *J* 8.9, 5.9, H–3), 4.03 (2H, qd, *J* 7.1, 2.2, OCH_2CH_3), 4.11–4.15 (1H, m, H–4), 4.57 (1H, t, *J* 5.1, H–2), 4.85 (1H, d, *J* 5.1, H–1), 5.07 (1H, d, *J* 3.7, H–5), 7.16–7.24 (4H, m, H–Ar), 7.44–7.48 (2H, m, H–Ph), 7.56–7.59 (1H, m, H–Ph), 7.97 (2H, d, *J* 7.4, H–Ph); ^{13}C NMR (126 MHz, $CDCl_3$) δ_C: 13.9 (OCH_2CH_3), 19.0 (CO-CH_3), 40.6 (CH-2), 45.8 (CH-4), 47.8 (CH-3), 56.6 (CH-1), 61.8 (OCH_2CH_3) 99.7 (CH-5), 123.7 (CH–Ar), 125.8 (CH-Ar), 127.5 (CH-Ar), 128.4 (CH-Ar), 128.8 (2×CH-Ph), 128.9 (2×CH-Ph), 133.8 (CH-Ph), 140.2 (CH-C=OO*C*), 143.5 (C-Ph1), 143.8 (C1-Ar), 147.7 (C6-Ar), 169.6 (*C*O_2Et), 170.1 (CH-*C*=OO), 194.4 (*C*=OPh);

16a' (minor): ^{1}H NMR (500 MHz, $CDCl_3$) δ_H: 1.14 (3H, t, *J* 7.1, OCH_2CH_3), 1.90 (3H, s, $COCH_3$), 3.41 (1H, dd, *J* 8.8, 3.8, H–3), 4.03 (2H, qd, *J* 7.1, 2.2, OCH_2CH_3), 4.11–4.15 (1H, m, H–4), 4.67 (1H, d, *J* 8.3, H–1), 4.77 (1H, dd, *J* 8.3, 3.8, H–2), 5.21 (1H, d, *J* 4.9, H–5), 7.16–7.24 (4H, m, H–Ar), 7.44–7.48 (2H, m, H–Ph), 7.56–7.59 (1H, m, H–Ph), 7.97 (2H, d, *J* 7.4, H–Ph).

^{13}C NMR (126 MHz, $CDCl_3$) δ_C: 14.0 (OCH_2CH_3), 19.1 (CO-CH_3), 40.5 (CH-2), 45.5 (CH-4), 47.5 (CH-3), 57.3 (CH-1), 62.0 (OCH_2CH_3) 100.4 (CH-5), 123.7 (CH–Ar), 125.9 (CH-Ar), 127.6 (CH-Ar), 128.3 (CH-Ar), 128.6 (2×CH-Ph), 128.9 (2×CH-Ph), 133.8 (CH-Ph), 140.2 (CH-C=OO*C*), 141.0 (C-Ph1), 141.9 (C1-Ar), 143.8 (C6-Ar), 168.4 (*C*O_2Et), 168.6 (CH-*C*=OO), 193.6 (*C*=OPh); *m/z* (NSI^+) 413 ($[M+Na]^+$, 100%); HRMS (NSI^+) $C_{24}H_{22}O_5Na$ $[M+Na]^+$, found 413.1354, requires 413.1359 (–1.3 ppm).

2-((4a*R*,9*R*,9a*S*)-6-Chloro-3-methyl-1-oxo-1,4a,9,9a-tetrahydroindeno[2,1-*c*]pyran-9-yl)-1,3-diphenylpropane-1,3-dione (17a)

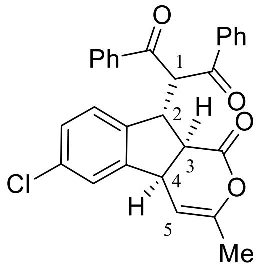

Following General Procedure 9, the corresponding TCP-ester (43.0 mg, 0.1 mmol), 1,3-dipheny-1,3-propanedione (44.8 mg, 0.2 mmol) and PS-BEMP (2.0 mmol/g loading, 90.0 mg, 0.2 mmol) in THF (0.25 mL) and subsequent chromatography (80:20 Petrol : EtOAc, R_f 0.2) afforded the title compound (30.6 mg, 67%) as a colorless oil; $[\alpha]_D^{20}$ +18.7 (*c* 1.0, $CHCl_3$); Chiral HPLC analysis, Chiralpak IB (97:3 hexane : IPA, flow rate 1.5 mLmin^{-1}, 211 nm, 40 °C) t_R major: 18.7 min, t_R minor: 31.3 min, 99:1 er; ν_{max} (film) 2926 (C-H), 1749 (C=O), 1695 (C=O), 1668 (C=O); ^{1}H NMR (500 MHz, $CDCl_3$) δ_H: 1.88 (3H, s, COCH_3), 3.44 (1H, dd, *J* 9.0, 4.6, H–3), 4.07–4.13 (1H, m, H–4), 4.76 (1H, t, *J* 5.2, H–2), 5.12 (1H, d, *J* 4.0, H–5), 5.72 (1H, d, *J* 5.9, H–1), 7.06–7.18 (3H, m, H–Ar), 7.39–7.45 (4H, m, H–Ph), 7.51–7.55 (2H, m, H–Ph), 7.91–7.93 (4H, m, H–Ph); ^{13}C NMR (126 MHz, $CDCl_3$) δ_C: 19.1 (CO-*C*H$_3$), 40.4 (CH-2), 45.6 (CH-4), 48.0 (CH-3), 59.2 (CH-1), 99.3 (CH-5), 124.0 (CH-Ar), 127.0 (CH-Ar), 127.8 (CH-Ar), 128.8 (2×CH-Ph2), 128.9 (2×CH-Ph2), 129.1 (2×CH-Ph1), 129.2 (2×CH-Ph1), 134.0 (CH-Ph2), 133.9 (CH-Ph1), 134.0 (C-Cl), 135.8 (CH-C=OO*C*), 136.6 (C-Ph2), 139.4 (C-Ph1), 145.6 (C1-Ar), 148.5 (C6-Ar), 169.6 (CH-*C*=OO), 194.9 (*C*=OPh2), 195.5 (*C*=OPh1); *m/z* (NSI$^+$) 479 ([M+Na]$^+$, 100%), 935 ([2M+Na]$^+$, 65%); HRMS (NSI$^+$) $C_{28}H_{22}ClO_4$ [M+H]$^+$, found 457.1199, requires 457.1201 (–0.5 ppm).

2-((4a*R*,9*R*,9a*S*)-6-Fluoro-3-methyl-1-oxo-1,4a,9,9a-tetrahydroindeno[2,1-*c*]pyran-9-yl)-1,3-diphenylpropane-1,3-dione (18a)

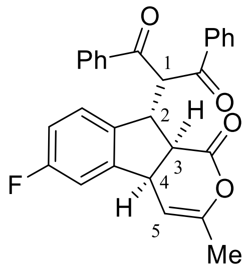

Following General Procedure 9, the corresponding TCP-ester (41.4 mg, 0.1 mmol), 1,3-dipheny-1,3-propanedione (44.8 mg, 0.2 mmol) and PS-BEMP (2.0 mmol/g loading, 90.0 mg, 0.2 mmol) in THF (0.25

mL) and subsequent chromatography (80:20 Petrol : EtOAc, R_f 0.2) afforded the title compound (24.2 mg, 55%) as a yellow oil; $[\alpha]_D^{20}$ +34.1 (*c* 1.0, $CHCl_3$); Chiral HPLC analysis, Chiralpak IB (97:3 hexane : IPA, flow rate 1.5 mLmin^{-1}, 211 nm, 40 °C) t_R major: 18.6 min, t_R minor: 30.7 min, 98:2 er; ν_{max} (film) 2924 (C-H), 1745 (C=O), 1695 (C=O), 1663 (C=O); ^{1}H NMR (500 MHz, $CDCl_3$) δ_H: 1.88 (3H, s, COCH_3), 3.46 (1H, dd, *J* 8.9, 4.3, H–3), 4.07–4.09 (1H, m, H–4), 4.78 (1H, t, *J* 4.9, H–2), 5.12 (1H, d, *J* 4.4, H–5), 5.72 (1H, d, *J* 6.0, H–1), 6.78–6.81 (2H, m, H–Ar), 7.16–7.18 (1H, m, H–Ar), 7.38–7.44 (4H, m, H–Ph), 7.52–7.57 (2H, m, H–Ph), 7.90–7.92 (4H, m, H–Ph); ^{19}F NMR (282 MHz, $CDCl_3$) δ_F: −114.3; ^{13}C NMR (126 MHz, $CDCl_3$) δ_C: 19.1 (CO-CH$_3$), 40.4 (CH-2), 45.9 (CH-4), 47.8 (CH-3), 59.3 (CH-1), 99.2 (CH-5), 110.7 (CH-Ar), 110.8 (d, $^2J_{CF}$ = 22.6, C_{Ar}-F), 114.6 (CH-Ar), 114.6 (d, $^2J_{CF}$ = 22.5, C_{Ar}-F), 127.1 (CH-Ar), 128.8 (2×CH-Ph2), 128.9 (2×CH-Ph2), 128.9 (d, $^3J_{CF}$ = 8.9, C_{Ar}-F), 129.1 (2×CH-Ph1), 129.1 (2×CH-Ph1), 133.9 (CH-Ph2), 134.0 (CH-Ph1), 135.9 (CH-C=OO*C*), 136.3 (C-Ph2), 136.6 (C-Ph1), 145.9 (C1-Ar), 148.6 (C6-Ar), 163.0 (d, $^1J_{CF}$ = 246.9, C_{Ar}-F), 164.0 (C-F), 169.6 (CH-*C*=OO), 195.0 (*C*=OPh2), 195.5 (*C*=OPh1); *m/z* (NSI$^+$) 463 ([M+Na]$^+$, 100%), 903 ([2M+Na]$^+$, 25%); HRMS (NSI$^+$) $C_{24}H_{28}O_6Cl$ [M+H]$^+$, found 463.1306, requires 463.01316 (–2.2 ppm).

2-((4a*R*,9*R*,9a*S*)-3,7-Dimethyl-1-oxo-1,4a,9,9a-tetrahydroindeno[2,1-*c*]pyran-9-yl)-1,3-diphenylpropane-1,3-dione (19a)

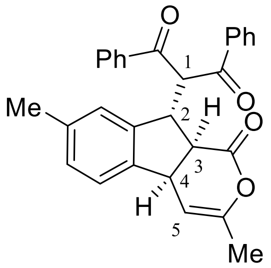

Following General Procedure 9, the corresponding TCP-ester (41.0 mg, 0.1 mmol), 1,3-dipheny-1,3-propanedione (44.8 mg, 0.2 mmol) and PS-BEMP (2.0 mmol/g loading, 90.0 mg, 0.2 mmol) in THF (0.25 mL) and subsequent chromatography (80:20 Petrol : EtOAc, R_f 0.2) afforded the title compound (20.1 mg, 46%) as a yellow oil; $[\alpha]_D^{20}$ +17.2 (*c* 0.5, $CHCl_3$); Chiral HPLC analysis, Chiralpak IB (98:2 hexane : IPA, flow rate 1.0 mLmin^{-1}, 211 nm, 40 °C) t_R major: 22.8 min, t_R minor: 33.6 min, >99:1 er; ν_{max} (film) 2922 (C-H), 1749 (C=O), 1697 (C=O), 1668 (C=O); ^{1}H NMR (500 MHz, $CDCl_3$) δ_H: 1.85 (3H, s, COCH_3), 2.21 (3H, s, ArC-CH_3), 3.43 (1H, dd, *J* 9.0, 4.6, H–3), 4.04–4.09 (1H, m, H–4), 4.78–4.80 (1H, m, H–2), 5.16 (1H, d, *J* 4.6, H–5), 5.69 (1H, d, *J* 6.2, H–1), 6.95–7.02 (3H, m, H–Ar), 7.40 (4H, dt, *J* 15.3, 7.8, H–Ph), 7.53 (2H, dd, *J* 16.3, 7.6, H–Ph), 7.90 (4H, d, *J* 8.0, H–Ph); ^{13}C NMR (126 MHz, $CDCl_3$) δ_C: 19.0 (CO-*C*H$_3$), 21.4 (ArC-*C*H$_3$), 40.0 (CH-2), 45.6 (CH-4), 48.4 (CH-3), 59.4 (CH-1), 100.0 (CH-5), 123.4 (CH-Ar), 126.2 (CH-Ar), 128.8 (2×CH-Ph2), 128.9 (2×CH-Ph2), 129.0 (2×CH-Ph1), 129.0 (2×CH-Ph1), 129.1 (CH-Ar), 133.7 (CH-Ph2), 133.8 (CH-Ph1), 136.0 (C-Cl), 136.7 (CH-C=OO*C*), 137.3 (C-Ph2), 140.8 (C-Ph1), 141.1 (C1-Ar), 147.9 (C6-Ar), 170.1 (CH-*C*=OO), 195.1 (*C*=OPh2), 195.5 (*C*=OPh1); *m/z* (NSI$^+$) 459 ([M+Na]$^+$, 100%), 895 ([2M+Na]$^+$, 50%); HRMS (NSI$^+$) $C_{29}H_{24}O_4Na$ [M+Na]$^+$, found 459.1567, requires 459.1567 (0.0 ppm).

2-((4a*R*,9*R*,9a*S*)-3-(*tert*-Butyl)-1-oxo-1,4a,9,9a-tetrahydroindeno[2,1-*c*]pyran-9-yl)-1,3-diphenylpropane-1,3-dione (20a)

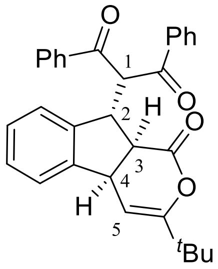

Following General Procedure 9, the corresponding TCP-ester (43.8 mg, 0.1 mmol), 1,3-dipheny-1,3-propanedione (44.8 mg, 0.2 mmol) and PS-BEMP (2.0 mmol/g loading, 90.0 mg, 0.2 mmol) in THF (0.25

mL) and subsequent chromatography (80:20 Petrol : EtOAc, R_f 0.2) afforded the title compound (35.0 mg, 74%) as a yellow oil; $[\alpha]_D^{20}$ +20.4 (*c* 0.5, $CHCl_3$); Chiral HPLC analysis, Chiralpak IB (97:3 hexane : IPA, flow rate 1.5 mLmin^{-1}, 211 nm, 40 °C) t_R major: 10.1 min, t_R minor: 12.1 min, 96.5:3.5 er; ν_{max} (film) 2997 (C-H), 1749 (C=O), 1695 (C=O), 1597 (C=O); ^{1}H NMR (500 MHz, $CDCl_3$) δ_H: 1.10 (9H, s, $(CH_3)_3$), 3.44 (1H, dd, *J* 8.9, 4.5, H–3), 4.11 (1H, dd, *J* 8.9, 5.0, H–4), 4.79 (1H, t, *J* 5.1, H–2), 5.18 (1H, d, *J* 5.0, H–5), 5.74 (1H, d, *J* 5.9, H–1), 7.08–7.19 (4H, m, H–Ar), 7.37–7.43 (4H, m, H–Ph),7.50–7.54 (2H, m, H–Ph), 7.89–7.92 (4H, m, H–Ph); ^{13}C NMR (126 MHz, $CDCl_3$) δ_C: 27.5 ($C(CH_3)_3$), 34.8 ($C(CH_3)_3$), 40.2 (CH-2), 45.6 (CH-4), 48.3 (CH-3), 59.1 (CH-1), 96.0 (CH-5), 123.7 (CH–Ar), 125.5 (CH-Ar), 127.5 (CH-Ar), 128.2, (CH-Ar), 128.8 (2×CH-Ph1), 128.9 (2×CH-Ph2), 129.0 (2×CH-Ph1), 129.0 (2×CH-Ph2), 133.9 (CH-Ph1), 133.7 (CH-Ph2), 136.0 (C-Ph1), 136.7 (C-Ph2), 141.1 (C1-Ar), 143.8 (C6-Ar), 158.8 (C=OO*C*$(CH_3)_3$), 170.2 (CH-*C*=OO), 195.1 (*C*=OPh2), 195.7 (*C*=OPh1); *m/z* (NSI$^+$) 487 ([M+Na]$^+$, 100%), 951 ([2M+Na]$^+$, 70%); HRMS (NSI$^+$) $C_{28}H_{23}O_4$ [M+H]$^+$, found 423.1587, requires 423.1591 (–0.9 ppm).

2-((4a*S*,9*S*,9a*R*)-1-Oxo-3-phenyl-1,4a,9,9a-tetrahydroindeno[2,1-*c*]pyran-9-yl)-1,3-diphenylpropane-1,3-dione (21a)

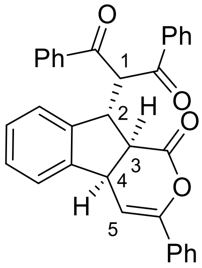

Following General Procedure 9, the corresponding TCP-ester (45.8 mg, 0.1 mmol), 1,3-dipheny-1,3-propanedione (44.8 mg, 0.2 mmol) and PS-BEMP (2.0 mmol/g loading, 90.0 mg, 0.2 mmol) in THF (0.25 mL) and subsequent chromatography (80:20 Petrol : EtOAc, R_f 0.2) afforded the title compound (34.4 mg, 71%) as a yellow solid. mp 85–87 °C; $[\alpha]_D^{20}$ +50.3 (*c* 0.5, $CHCl_3$); Chiral HPLC analysis, Chiralpak IB (97:3 hexane : IPA, flow rate 1.5 mLmin^{-1}, 211 nm, 40 °C) t_R major: 26.9 min, t_R minor: 40.3 min, >99:1 er; ν_{max} (film) 3077 (C-H), 1740 (C=O), 1694 (C=O), 1665 (C=O); ^{1}H NMR (500 MHz, $CDCl_3$) δ_H: 3.60 (1H, dd, *J* 9.0, 4.0, H–3), 4.37 (1H, dd, *J* 8.9, 5.4, H–4), 4.92 (1H, dd, *J* 5.8, 4.1, H–2), 5.78 (1H, d, *J* 6.2, H–5), 5.97 (1H, d, *J* 5.3, H–1), 7.19–7.24 (3H, m, H–Ar), 7.33–7.35 (1H, m, H–Ar), 7.37–7.45 (7H, m, 2×CH-Ph1, 2×CH-Ph2, 3×CH-Ph3), 7.50–7.56 (2H, m, 1×CH-Ph1, 1×CH-Ph2), 7.58–7.62 (2H, m, CH-Ph3), 7.90–7.95 (4H, m, 2×CH-Ph1, 2×CH-Ph2); ^{13}C NMR (126 MHz, $CDCl_3$) δ_C: 40.9 (CH-2), 45.6 (CH-4), 48.6 (CH-3), 59.4 (CH-1), 99.8 (CH-5), 123.8 (CH-Ar), 124.9 (2×CH-Ph3), 125.7 (CH-Ar), 127.2 (CH-Ph1), 128.3, (CH-Ar), 128.6 (2×CH-Ph2), 128.8 (2×CH-Ph2), 128.9 (2×CH-Ph1, Ar), 129.0

(2×CH-Ph3),129.1 (2×CH-Ph1), 129.3 (CH-Ph3), 132.4 (C-Ph3), 133.8 (CH-Ph2), 133.9 (CH-Ph1), 136.0(CH-C=OO*C*), 136.6 (C-Ph2), 141.0 (C-Ph1), 143.3 (C1-Ar), 148.8 (C6-Ar), 169.6 (CH-*C*=OO), 195.0 (*C*=OPh2), 195.5 (*C*=OPh1); *m/z* (NSI^+) 507 ($[M+Na]^+$, 65%), 485 ($[M+H]^+$, 35%); HRMS (NSI^+) $C_{33}H_{28}O_5N$ $[M+O+NH_4]^+$, found 518.1954, requires 518.1962 (–1.5 ppm).

2-((4a*R*,9*R*,9a*S*)-3-(4-Methoxyphenyl)-1-oxo-1,4a,9,9a-tetrahydroindeno[2,1-*c*]pyran-9-yl)-1,3-diphenylpropane-1,3-dione (22a)

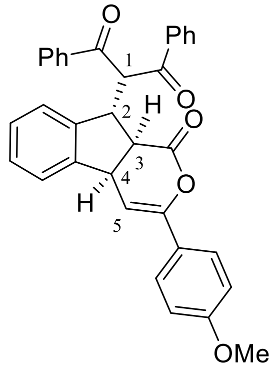

Following General Procedure 9, the corresponding TCP-ester (48.8 mg, 0.1 mmol), 1,3-dipheny-1,3-propanedione (44.8 mg, 0.2 mmol) and PS-BEMP (2.0 mmol/g loading, 90.0 mg, 0.2 mmol) in THF (0.25 mL) and subsequent chromatography (98:2 CH_2Cl_2 : EtOAc, R_f 0.2) afforded the title compound (35.4 mg, 69%) as a yellow oil; $[\alpha]_D^{20}$ +68.8 (*c* 0.5, $CHCl_3$); Chiral HPLC analysis, Chiralpak AS-H (90:10 hexane : IPA, flow rate 1.0 $mLmin^{-1}$, 211 nm, 40 °C) t_R major: 36.5 min, t_R minor: 43.1 min, >99:1 er; ν_{max} (film) 2932 (C-H), 1751 (C=O), 1694 (C=O), 1670 (C=O); ^1H NMR (500 MHz, $CDCl_3$) δ_H: 3.57 (1H, dd, *J* 8.9, 4.0, H–3), 3.81 (3H, s, OC*H*$_3$), 4.33 (1H, dd, *J* 8.9, 5.3, H–4), 4.91 (1H, dd, *J* 5.7, 4.0, H–2), 5.76 (1H, d, *J* 6.2, H–5), 5.82 (1H, d, *J* 5.7, H–1), 6.87 (2H, d, *J* 8.9, H–Ph_{OMe}), 7.10–7.13 (1H, m, H–Ar), 7.18–7.19 (2H, m, H–Ar), 7.23–7.25 (1H, m, H–Ar), 7.37–7.44 (4H, m, H–Ph), 7.51–7.55 (4H, m, 2×H–Ph_{OMe} + 2×H–Ph), 7.91–7.94 (4H, m, H–Ph); ^{13}C NMR (126 MHz, $CDCl_3$) δ_C: 40.8 (CH-2), 45.7 (CH-4), 48.6 (CH-3), 55.5 (O*C*H$_3$), 59.5 (CH-1), 97.9 (CH-5), 113.9 (2×CH-Ph_{OMe}), 123.8 (CH-Ar), 125.0 (C-Ph_{OMe}), 125.7 (CH-Ar), 126.3 (2×CH-Ph_{OMe}), 127.7 (CH-Ar), 128.3 (CH-Ar), 128.8 (2×CH-Ph1), 128.9 (2×CH-Ph2), 129.0 (2×CH-Ph1), 129.1 (2×CH-Ph2), 133.8 (CH-Ph1), 133.9 (CH-Ph2), 135.8 (CH-C=OO*C*), 136.0 (C-Ph2), 136.7 (C-Ph_{OMe}), 141.0 (C-Ph1), 143.5 (C1-Ar), 148.6 (C6-Ar), 160.4 (Ph-*C*-OMe), 169.7 (CH-*C*=OO), 195.0 (*C*=OPh2), 195.6 (*C*=OPh1); *m/z* (NSI^+) 537 ($[M+Na]^+$, 100%), 1051 ($[2M+Na]^+$, 25%); HRMS (NSI^+) $C_{34}H_{26}O_5Na$ $[M+Na]^+$, found 537.1659, requires 537.1672 (–2.5 ppm).

2-((4a*R*,9*R*,9a*S*)-1-Oxo-3-(4-(trifluoromethyl)phenyl)-1,4a,9,9a-tetrahydroindeno[2,1-*c*]pyran-9-yl)-1,3-diphenylpropane-1,3-dione (23a)

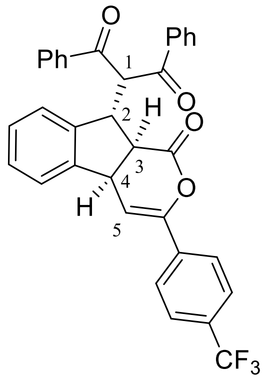

Following General Procedure 9, the corresponding TCP-ester (52.6 mg, 0.1 mmol), 1,3-dipheny-1,3-propanedione (44.8 mg, 0.2 mmol) and PS-BEMP (2.0 mmol/g loading, 90.0 mg, 0.2 mmol) in THF (0.25 mL) and subsequent chromatography (80:20 Petrol : EtOAc, R_f 0.2) afforded the title compound (37.0 mg, 67%) as a yellow oil; $[\alpha]_D^{20}$ +19.8 (*c* 0.5, $CHCl_3$); Chiral HPLC analysis, Chiralpak AS-H (90:10 hexane : IPA, flow rate 1.0 mLmin^{-1}, 211 nm, 40 °C) t_R major: 18.9 min, t_R minor: 23.5 min, 97:3 er; ν_{max} (film) 2924 (C-H), 1751 (C=O), 1695 (C=O), 1668 (C=O); ^{1}H NMR (500 MHz, $CDCl_3$) δ_H: 3.67 (1H, dd, *J* 9.1, 3.9, H–3), 4.42 (1H, dd, *J* 9.0, 5.4, H–4), 4.90 (1H, dd, *J* 5.5, 4.1, H–2), 5.78 (1H, d, *J* 5.9, H–5), 6.08 (1H, d, *J* 5.4, H–1), 7.20–7.21 (2H, m, H–Ar), 7.37–7.46 (4H, m, H–Ph), 7.53–7.57 (2H, m, H–Ar), 7.60–7.62 (2H, m, H–Ph_{CF3}), 7.71–7.73 (2H, m, H–Ph_{CF3}), 7.89–7.94 (4H, m, H–Ph); ^{19}F NMR (282 MHz, $CDCl_3$) δ_F: –63.0 (C*F₃*); ^{13}C NMR (126 MHz, $CDCl_3$) δ_C: 41.0 (CH-2), 45.4 (CH-4), 48.7 (CH-3), 59.3 (CH-1), 102.0 (CH-5), 123.8 (CH-Ar), 125.1 (2×CH-Ph_{CF3}), 125.7 (q, $^3J_{CF}$ = 3.7, C_{Ar}-CF_3), 125.7 (CH-Ar), 126.8 (q, $^1J_{CF}$ = 285.2, C_{Ar}-CF_3), 127.9 (CH-Ar), 128.5 (CH-Ar), 128.8 (2×CH-Ph2 + 2×CH-Ph1), 128.9 (2×CH-Ph2 + 2×CH-Ph_{CF3}), 129.0 (q, $^2J_{CF}$ = 40.9, C_{Ar}-CF_3), 129.1 (2×CH-Ph1), 129.1 (2×CH-Ph_{CF3}), 130.8 (C-Ph_{CF3}), 133.9 (CH-Ph1), 134.0 (CH-Ph2), 135.8 (CH-C=OO*C*), 136.0 (C-Ph2), 136.5 (C-Ph_{CF3}), 141.0 (C-Ph1), 142.9 (C1-Ar), 147.6 (C6-Ar), 169.2 (CH-*C*=OO), 195.0 (*C*=OPh2), 195.5 (*C*=OPh1); *m/z* (NSI^+) 575 ($[M+Na]^+$, 100%); HRMS (NSI^+) $C_{32}H_{23}O_4F_3Na$ $[M+Na]^+$, found 575.1428, requires 575.1441 (–2.2 ppm).

2-((4a*R*,9*R*,9a*S*)-3-(4-Bromophenyl)-1-oxo-1,4a,9,9a-tetrahydroindeno[2,1-*c*]pyran-9-yl)-1,3-diphenylpropane-1,3-dione (24a)

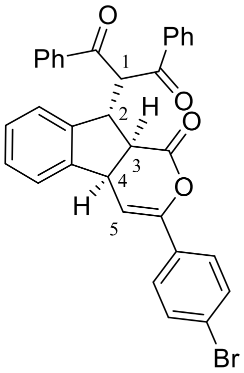

Following General Procedure 8, the corresponding TCP-ester (53.7 mg, 0.1 mmol), 1,3-dipheny-1,3-propanedione (44.8 mg, 0.2 mmol) and PS-BEMP (2.0 mmol/g loading, 90.0 mg, 0.2 mmol) in THF (0.25 mL) and subsequent chromatography (80:20 Petrol : EtOAc, R_f 0.2) afforded the title compound (36.6 mg, 65%) as a yellow oil; $[\alpha]_D^{20}$ +30.6 (*c* 0.5, $CHCl_3$); Chiral HPLC analysis, Chiralpak AS-H (90:10 hexane : IPA, flow rate 1.0 mLmin^{-1}, 211 nm, 40 °C) t_R major: 27.6 min, t_R minor: 33.3 min, 97:3 er; ν_{max} (film) 3077 (C-H), 1740 (C=O), 1694 (C=O), 1665 (C=O); ^{1}H NMR (500 MHz, $CDCl_3$) δ_H: 3.62 (1H, dd, *J* 9.0, 3.9, H–3), 4.36 (1H, dd, *J* 9.0, 5.3, H–4), 4.86–4.91 (1H, m, H–2), 5.76 (1H, d, *J* 6.0, H–5), 5.97 (1H, d, *J* 5.3, H–1), 7.19–7.20 (2H, m, H–Ar), 7.37–7.45 (6H, m, 2×H–Ph + 2×H–Ar + 2×H–Ph_{Br}), 7.47 (4H, s, H–Ph), 7.53–7.58 (2H, m, 2×H–Ph_{Br}), 7.89–7.94 (4H, m, H–Ph); ^{13}C NMR (126 MHz, $CDCl_3$) δ_C: 40.9 (CH-2), 45.4 (CH-4), 48.7 (CH-3), 59.4 (CH-1), 100.3 (CH-5), 123.8 (CH-Ar), 125.7 (CH-Ar), 126.4 (2×CH-Ph_{Br}), 127.9 (CH-Ar), 128.4 (CH-Ar), 128.8 (2×CH-Ph2 + 2×CH-Ph1), 128.9 (2×CH-Ph2), 129.0 (2×CH-Ph1), 129.1 (2×CH-Ph_{Br}), 131.8 (2×CH-Ph_{Br}), 133.9 (C-Ph1), 134.0 (C-Ph2), 135.1 (CH-C=OO*C*), 136.0 (C-Ph2), 136.6 (C-Ph_{Br}), 141.0 (C-Ph1), 143.1 (C1-Ar), 145.2 (C6-Ar), 169.3 (CH-*C*=OO), 195.0 (*C*=OPh2), 195.5 (*C*=OPh1); *m/z* (NSI$^+$) 586 ([M+Na]$^+$, 90%); HRMS (NSI$^+$) $C_{33}H_{23}O_5BrNa$ [M+Na]$^+$, found 601.0606, requires 601.0621 (–2.5 ppm).

2-((4a*R*,9*R*,9a*S*)-3-(4-Chlorophenyl)-1-oxo-1,4a,9,9a-tetrahydroindeno[2,1-*c*]pyran-9-yl)-1,3-diphenylpropane-1,3-dione (25a)

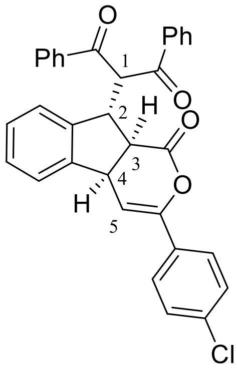

Following General Procedure 9, the corresponding TCP-ester (49.2 mg, 0.1 mmol), 1,3-dipheny-1,3-propanedione (44.8 mg, 0.2 mmol) and PS-BEMP (2.0 mmol/g loading, 90.0 mg, 0.2 mmol) in THF (0.25 mL) and subsequent chromatography (80:20 Petrol : EtOAc, R_f 0.2) afforded the title compound (34.8 mg, 67%) as a yellow oil; $[\alpha]_D^{20}$ +55.0 (*c* 0.5, $CHCl_3$); Chiral HPLC analysis, Chiralpak AS-H (90:10 hexane : IPA, flow rate 1.0 mLmin^{-1}, 211 nm, 40 °C) t_R major: 25.7 min, t_R minor: 30.7 min, 96:4 er; ν_{max} (film) 2924 (C-H), 1759 (C=O), 1694 (C=O), 1595 (C=O); ^{1}H NMR (500 MHz, $CDCl_3$) δ_H: 3.62 (1H, dd, *J* 9.0, 4.0, H–3), 4.37 (1H, dd, *J* 9.0, 5.3, H–4), 4.89–4.91 (1H, m, H–2), 5.77 (1H, d, *J* 6.0, H–5), 5.95 (1H, d, *J* 5.3, H–1), 7.19–7.20 (2H, m, H–Ar), 7.31–7.33 (2H, m, H–Ar), 7.37–7.45 (6H, m, 4×H–Ph + 2×H–Ph_{Cl}), 7.53–7.56 (4H, m, 2×H–Ar + 2×H–Ph_{Cl}), 7.89–7.94 (4H, m, H–Ph); ^{13}C NMR (126 MHz, $CDCl_3$) δ_C: 40.8 (CH-2), 45.4 (CH-4), 48.5 (CH-3), 59.3 (CH-1), 100.1 (CH-5), 123.7 (CH-Ar), 126.1 (2×CH-Ph_{Cl}), 127.2 (CH-Ar), 127.7 (CH-Ar), 128.3 (CH-Ar), 128.7 (2×CH-Ph2 + 2×CH-Ph1), 128.8 (2×CH-Ph2), 128.9 (2×CH-Ph1), 129.0 (2×CH-Ph_{Cl}), 129.0 (2×CH-Ph_{Cl}), 130.8 (C-Ph_{Cl}), 133.8 (C-Ph1 + C-Ph2), 135.1 (CH-C=OO*C*), 135.9 (C-Ph2), 136.5 (C-Ph_{Cl}), 140.9 (C-Ph1), 143.0 (C1-Ar), 147.8 (C6-Ar), 169.2 (CH-*C*=OO), 194.9 (*C*=OPh2), 195.4 (*C*=OPh1); *m/z* (NSI$^+$) 541 ([M+Na]$^+$, 70%); HRMS (NSI$^+$) $C_{33}H_{23}O_4Cl_1Na$ [M+Na]$^+$, found 541.1168, requires 541.1177 (–1.7 ppm).

2-((4a*R*,9*R*,9a*S*)-6-Chloro-3-methyl-1-oxo-1,4a,9,9a-tetrahydroindeno[2,1-*c*]pyran-9-yl)-1,3-bis(4-methoxyphenyl)propane-1,3-dione (26a)

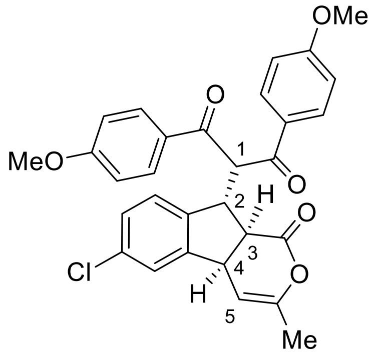

Following General Procedure 9, the corresponding TCP-ester (43.0 mg, 0.1 mmol), 1,3-bis(4-fluorophenyl)propane-1,3-dione (56.8 mg, 0.2 mmol) and PS-BEMP (2.0 mmol/g loading, 90.0 mg, 0.2 mmol) in THF (0.25 mL) and subsequent chromatography (80:20 Petrol : EtOAc, R_f 0.15) afforded the title compound (33.6 mg, 65%) as white needles. mp 145–146 °C; $[\alpha]_D^{20}$ +10.2 (*c* 0.5, $CHCl_3$); Chiral HPLC analysis, Chiralpak AS-H (90:10 hexane : IPA, flow rate 1.5 mLmin^{-1}, 211 nm, 40 °C) t_R minor: 17.5 min, t_R major: 25.3 min, 96.5:3.5 er; ν_{max} (film) 2938 (C-H), 1734 (C=O), 1676 (C=O), 1655 (C=O); ^{1}H NMR (500 MHz, $CDCl_3$) δ_H: 1.87 (3H, s, COCH_3), 3.38 (1H, dd, *J* 8.8, 4.1, H–3), 3.83 (3H, s, *p*-OCH_3), 3.84 (3H, s, *p*-OCH_3), 4.06–4.09 (1H, m, H–4), 4.80 (1H, dd, *J* 6.1, 4.1, H–2), 5.13 (1H, d, *J* 4.7, H–5), 5.48 (1H, d, *J* 6.6, H–1), 6.86–.6.89 (3H, m, H–Ph), 7.06–7.09 (2H, m, H–Ar), 7.11–7.15 (1H, m, H–Ar), 7.93 (4H, dd, *J* 8.8, 5.4, H–Ph); ^{13}C NMR (126 MHz, $CDCl_3$) δ_C: 19.1 (CO-*C*H_3), 40.2 (CH-2), 45.8 (CH-4), 47.9 (CH-3), 55.7 (2×*p*-O*C*H_3), 59.7 (CH-1), 99.2 (CH-5), 114.3 (4×*C*H-Ph1 + Ph2), 123.9 (*C*H-Ar), 127.2 (*C*H-Ar), 127.7 (*C*H-Ar), 128.9 (*C*-Ph1), 129.6 (*C*-Ph2), 131.4 (4×*C*H-Ph1 + Ph2), 133.9 (*C*-Cl), 139.9 (CH-C=OO*C*), 145.6 (*C*-Ar), 148.6 (*C*-Ar), 164.1 (*C*-Ph1), 164.2 (*C*-Ph2), 169.6 (CH-*C*=OO), 193.3 (*C*=OPh2), 193.9 (*C*=OPh1); *m/z* (NSI$^+$) 517 ([M+H]$^+$, 100%); HRMS (NSI$^+$) $C_{30}H_{26}Cl_1O_6Na$ [M+H]$^+$, found 517.1401, requires 517.1412 (–2.2 ppm).

1,3-Bis(4-fluorophenyl)-2-((4a*R*,9*R*,9a*S*)-3-methyl-1-oxo-1,4a,9,9a-tetrahydroindeno[2,1-*c*]pyran-9-yl)propane-1,3-dione (27a)

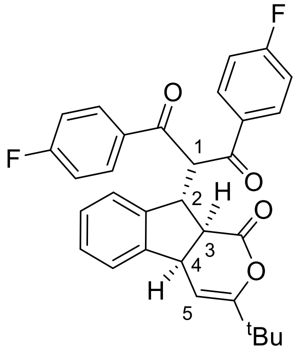

Following General Procedure 9, the corresponding TCP-ester (45.6 mg, 0.1 mmol), 1,3-bis(4-fluorophenyl)propane-1,3-dione (52.0 mg, 0.2 mmol) and PS-BEMP (2.0 mmol/g loading, 90.0 mg, 0.2 mmol) in THF (0.25 mL) and subsequent chromatography (80: 20 Petrol : EtOAc, R_f 0.20) afforded the title compound (39.5 mg, 79%) as colorless needles; $[\alpha]_D^{20}$ +55.0 (*c* 0.5, $CHCl_3$); Chiral HPLC analysis, Chiralpak IB (97:3 hexane : IPA, flow rate 1.5 mLmin^{-1}, 211 nm, 40 °C) t_R major: 7.5 min, t_R minor: 9.5 min, >99:1 er; ν_{max} (film) 2926 (C-H), 1746 (C=O), 1694 (C=O), 1597 (C=O); ^{1}H NMR (500 MHz, $CDCl_3$) δ_H: 1.10 (9H, s, CO(CH_3)$_3$), 3.38 (1H, dd, *J* 8.9, 4.3, H–3), 4.09 (1H, dd, *J* 8.9, 5.0, H–4), 4.77–4.82 (1H, m, H–2), 5.19 (1H, d, *J* 5.1, H–5), 5.57 (1H, d, *J* 6.3, H–1), 7.04–7.12 (4H, m, H–Ar), 7.13–7.11 (2H, m, H–PhF), 7.18–7.21 (2H, m, H–PhF), 7.91–7.96 (4H, m, H–PhF); ^{19}F NMR (282 MHz, $CDCl_3$) δ_F: –103.6 (PhF1), –103.2 (PhF2); ^{13}C NMR (126 MHz, $CDCl_3$) δ_C: 27.5 (CO(CH_3)$_3$), 40.2 (CH-2), 45.6 (CH-4), 48.3 (CH-3), 59.7 (*C*H-1), 99.9 (CH-5), 116.1 (d, $^2J_{CF}$ = 22.0, C_{Ar}-F), 116.2 (2×*C*H-PhF1), 116.2 (d, $^2J_{CF}$ = 21.9, C_{Ar}-F), 116.4 (2×*C*H-PhF2), 123.8 (*C*H-Ar), 125.5 (*C*H-Ar), 127.6 (*C*H-Ar), 128.4 (*C*H-Ar), 131.5 (d, $^3J_{CF}$ = 8.1, C_{Ar}-F), 131.6 (2×*C*H-PhF1), 131.7 (2×*C*H-PhF2), 132.3 (*C*-PhF1), 133.0 (*C*-PhF2), 140.9 (CH-C=OO*C*), 143.8 (*C*-Ar), 148.4 (*C*-Ar), 159.0 (*C*-PhF1), 164.6 (*C*-PhF2), 166.0 (d, $^1J_{CF}$ = 249.8, C_{Ar}-F), 170.1 (CH-*C*=OO), 193.4 (*C*=OPhF2), 194.0 (*C*=OPhF2); *m/z* (NSI$^+$) 523 ([M+Na]$^+$, 100%), 1023 ([2M+Na]$^+$, 75%); HRMS (NSI$^+$) $C_{24}H_{28}O_6Cl$ [M+Na]$^+$, found 523.1676, requires 523.1691 (–2.9 ppm).

2-((4a*R*,9*R*,9a*S*)-6-Chloro-3-methyl-1-oxo-1,4a,9,9a-tetrahydroindeno[2,1-*c*]pyran-9-yl)-1,3-di(furan-2-yl)propane-1,3-dione (28a)

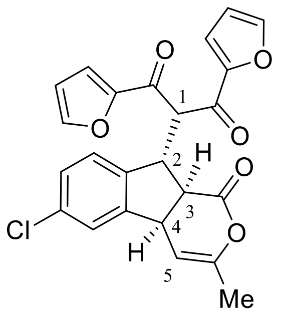

Following General Procedure 9, the corresponding TCP-ester (43.0 mg, 0.1 mmol), 1,3-di(furan-2-yl)propane-1,3-dione (40.8 mg, 0.2 mmol) and PS-BEMP (2.0 mmol/g loading, 90.0 mg, 0.2 mmol) in THF (0.25 mL) and subsequent chromatography (98:2 CH_2Cl_2 : EtOAc, R_f 0.2) afforded the title compound (27.1 mg, 62%) as a yellow oil; $[\alpha]_D^{20}$ +60.0 (*c* 0.5, $CHCl_3$); Chiral HPLC analysis, Chiralpak AS-H (90:10 hexane : IPA, flow rate 1.5 mLmin^{-1}, 211 nm, 40 °C) t_R minor: 11.5 min, t_R major: 18.9 min, 97.5:2.5 er; ν_{max} (film) 3136 (C-H), 1749 (C=O), 1684 (C=O), 1657 (C=O); ^{1}H NMR (500 MHz, $CDCl_3$) δ_H: 1.88 (3H, s, COC*H*$_3$), 3.40 (1H, dd, *J* 8.8, 3.5, H–3), 4.07–4.15 (1H, m, H–4), 4.80 (1H, dd, *J* 7.1, 3.5, H–2), 5.18 (1H, d, *J* 5.1, H–5), 5.21 (1H, d, *J* 7.1, H–1), 6.51 (2H, ddd, *J* 8.1, 3.6, 1.6, H–Fur), 7.05–7.12 (2H, m, H–Ar), 7.19–7.21 (1H, m, Ar-H), 7.30 (1H, d, *J* 3.6, H–Fur), 7.36 (1H, d, *J* 3.6, H–Fur), 7.54 (2H, dd, *J* 9.5, 0.8, H–Fur); ^{13}C NMR (126 MHz, $CDCl_3$) δ_C: 19.1 (CO-*C*H$_3$), 40.2 (CH-2), 45.5 (CH-4), 47.1 (CH-3), 60.2 (CH-1), 99.0 (CH-5), 113.1 (2×*C*H-fur), 119.5 (*C*H-fur), 119.6 (*C*H-fur), 124.0 (*C*H-Ar), 127.2 (*C*H-Ar), 127.7 (*C*H-Ar), 134.1 (*C*-Cl), 139.3 (CH-C=OO*C*), 145.6 (*C*-Ar), 147.5 (2×*C*H-fur), 148.8 (*C*-Ar), 151.7 (*C*-fur), 152.3 (*C*-fur), 169.3 (CH-*C*=OO), 182.4 (*C*=O-fur), 182.9 (*C*=O-fur); *m/z* (NSI$^+$) 459 ([M+Na]$^+$, 100%), 895 ([2M+Na]$^+$, 45%); HRMS (NSI$^+$) $C_{24}H_{28}O_6Cl$ [M+H]$^+$, found 437.0782, requires 437.0786 (–1.0 ppm).

5 Michael-Lactamization-Michael with Acyl Benzothiazoles

5.1 Screening of alternative acyl benzazoles

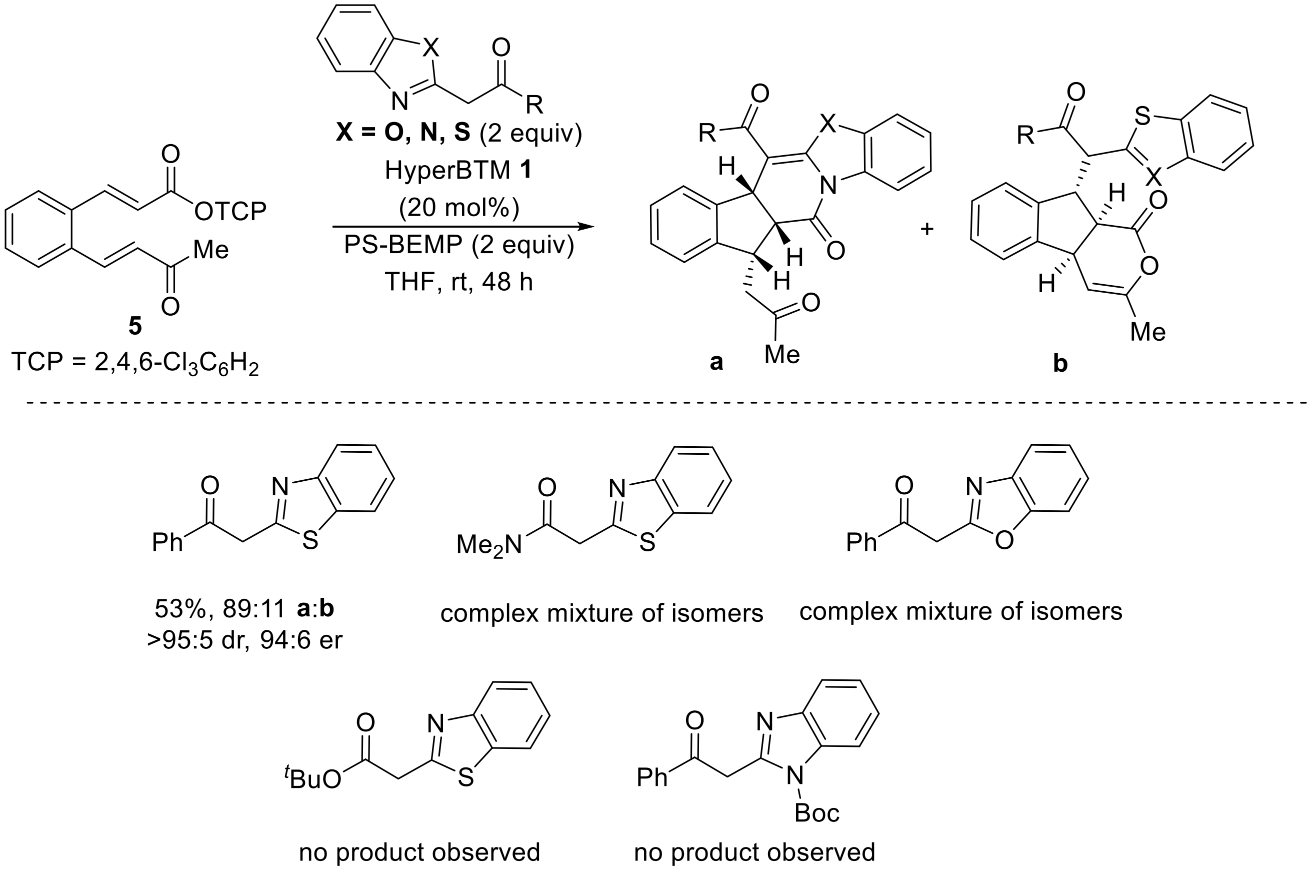

5.2 Data for indanes 35-44

(6a*R*,11*S*,11a*R*)-6-Benzoyl-11-(2-oxopropyl)-11,11a-dihydrobenzo[4,5]thiazolo[3,2-*a*]indeno[1,2-*d*]pyridin-12(6a*H*)-one (35a)

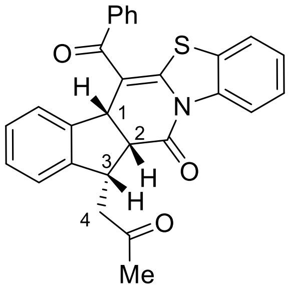

Following General Procedure 10, the corresponding TCP-ester (39.6 mg, 0.1 mmol), 2-phenacylbenzothiazole (50.6 mg, 0.2 mmol) and PS-BEMP (2.0 mmol/g loading, 90.0 mg, 0.2 mmol) in

THF (0.25 mL) and subsequent chromatography (80:20 Petrol : EtOAc, R_f 0.2) afforded the title compound (23.9 mg, 53%) as a yellow solid. mp 186–188 °C; $[\alpha]_D^{20}$ +83.7 (*c* 1.0, $CHCl_3$); Chiral HPLC analysis, Chiralpak IB (95:5 hexane : IPA, flow rate 1.5 mLmin^{-1}, 211 nm, 40 °C) t_R minor: 18.4 min, t_R major: 31.4 min, 94:6 er; ν_{max} (film) 2920 (C-H), 1711 (C=O), 1609 (C=O), 1481 (C_{Ar}-C_{Ar}); ^{1}H NMR (500 MHz, $CDCl_3$) δ_H: 2.42 (3H, s, COC*H*$_3$), 3.21 (1H, dd, *J* 18.1, 5.4, H–4a), 3.61 (1H, dd, *J* 18.1, 5.4, H–4b), 3.96 (1H, t, *J* 6.5, H–2), 4.01–4.03 (1H, m, H–3), 4.67 (1H, d, *J* 6.5, H–1), 7.09–7.22 (5H, m, H–Ph), 7.25–7.28 (1H, m, H-azaAr), 7.43–7.51 (4H, m, H–Ar), 7.69–7.71 (2H, m, H-azaAr), 8.31 (1H, d, *J* 6.8, H-azaAr); ^{13}C NMR (126 MHz, $CDCl_3$) δ_C: 30.5 (CO-*C*H$_3$), 42.0 (*C*H$_2$), 42.7 (*C*H-3), 43.0 (*C*H-2), 50.1 (*C*H-1), 104.6 (O=C-*C*=C), 118.5 (*C*H-azaAr), 121.8 (*C*H-azaAr), 122.1 (*C*H-azaAr), 123.2 (*C*H-Ar), 125.9 (*C*H-Ar), 126.7 (*C*H-azaAr), 127.4 (2×*C*H-Ph), 127.6 (*C*H-Ar), 127.7 (*C*-azaAr), 127.8 (*C*H-Ar), 128.6 (2×*C*H-Ph), 130.5 (*C*H-Ph), 136.2 (*C*-Ph), 139.4 (*C*-azaAr), 143.3 (*C*1-Ar), 143.6 (*C*6-Ar), 155.4 (O=C-C=*C*), 169.8 (CH-*C*=ON), 191.3 (*C*=OPh), 208.2 (*C*=OCH$_3$); *m/z* (NSI$^+$) 452 ([M+H]$^+$, 100%), 490 ([M+K]$^+$, 70%); HRMS (NSI$^+$) $C_{28}H_{22}O_3N_1S_1$ [M+H]$^+$, found 452.1309, requires 452.1315 (–1.3 ppm).

(6a*R*,11*S*,11a*R*)-6-Benzoyl-8-methyl-11-(2-oxopropyl)-11,11a-dihydrobenzo[4,5]thiazolo[3,2-*a*]indeno[1,2-*d*]pyridin-12(6a*H*)-one (36a)

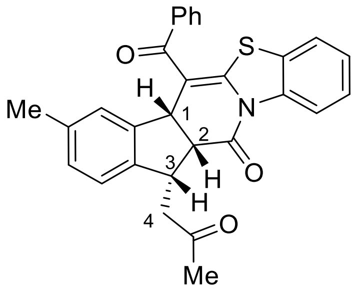

Following General Procedure 10, the corresponding TCP-ester (41.0 mg, 0.1 mmol), 2-phenacylbenzothiazole (50.6 mg, 0.2 mmol) and PS-BEMP (2.0 mmol/g loading, 90.0 mg, 0.2 mmol) in THF (0.25 mL) and subsequent chromatography (80:20 Petrol : EtOAc, R_f 0.2) afforded the title compound (36.8 mg, 79%) as a yellow solid. mp 255–257 °C; $[\alpha]_D^{20}$ +96.7 (*c* 0.5, $CHCl_3$); Chiral HPLC analysis, Chiralpak IB (97:3 hexane : IPA, flow rate 1.5 mLmin^{-1}, 211 nm, 40 °C) t_R major: 20.6 min, t_R minor: 37.6 min, 82:18 er; ν_{max} (film) 3017 (C-H), 1717 (C=O), 1614 (C=O), 1593 (C_{Ar}-C_{Ar}); ^{1}H NMR (500 MHz, $CDCl_3$) δ_H: 2.27 (3H, s, ArC-C*H*$_3$), 2.43 (3H, s, COC*H*$_3$), 3.20 (1H, dd, *J* 18.1, 5.4, H–4a), 3.60 (1H, dd, *J* 18.1, 5.4, H–4b), 3.93 (1H, t, *J* 6.7, H–2), 4.00 (1H, dd, *J* 10.4, 5.4, H–3) 4.67 (1H, d, *J* 6.7, H–1), 6.91 (1H, s, H–Ar), 7.00–7.04 (2H, m, H–Ar), 7.36–7.39 (1H, m, H-azaAr), 7.45–7.54 (5H, m, H-Ph), 7.72–7.74 (2H, m, H-azaAr), 8.34 (1H, d, *J* 6.8, H-azaAr); ^{13}C NMR (126 MHz, $CDCl_3$) δ_C: 21.4

(ArC-*C*H_3), 30.5 (CO-*C*H_3), 42.1 (*C*H_2), 42.4 (*C*H-3), 42.9 (*C*H-2), 50.2 (*C*H-1), 104.7 (O=C-*C*=C), 118.5 (*C*H-aAr), 121.8 (2×*C*H-aAr), 123.4 (*C*H-Ar), 125.9 (*C*H-Ar), 126.7 (*C*H-azaAr), 127.4 (2×*C*H-Ph), 128.5 (*C*H-Ar), 127.7 (*C*-aAr), 128.6 (2×*C*H-Ph), 130.5 (*C*H-Ph), 136.2 (*C*-Ph), 137.5 (Ar*C*-CH_3), 139.4 (*C*-azaAr), 140.6 (*C*1-Ar), 143.4 (*C*6-Ar), 155.4 (O=C-C=*C*), 169.9 (CH-*C*=ON), 191.3 (*C*=OPh), 208.3 (*C*=OCH_3); *m/z* (NSI^+) 504 ($[M+K]^+$, 100%), 466 ($[M+H]^+$, 72%); HRMS (NSI^+) $C_{29}H_{23}O_3$ N_1 Na_1S_1 $[M+Na]^+$, found 488.1278, requires 488.1291 (–2.6 ppm).

(6a*R*,11*S*,11a*R*)-6-Benzoyl-9-fluoro-11-(2-oxopropyl)-11,11a-dihydrobenzo[4,5]thiazolo[3,2-*a*]indeno[1,2-*d*]pyridin-12(6a*H*)-one (37a)

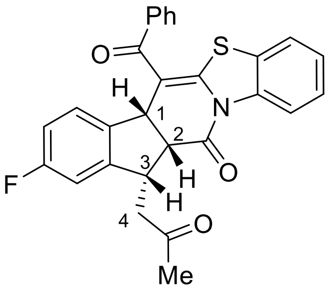

Following General Procedure 10, the corresponding TCP-ester (41.4 mg, 0.1 mmol), 2-phenacylbenzothiazole (50.6 mg, 0.2 mmol) and PS-BEMP (2.0 mmol/g loading, 90.0 mg, 0.2 mmol) in THF (0.25 mL) and subsequent chromatography (80:20 Petrol : EtOAc, R_f 0.2) afforded the title compound (19.7 mg, 42%) as a yellow solid. mp 234–236 °C; $[\alpha]_D^{20}$ +97.8 (*c* 0.5, $CHCl_3$); Chiral HPLC analysis, Chiralpak AS-H (95:5 hexane : IPA, flow rate 1.0 $mLmin^{-1}$, 211 nm, 40 °C) t_R minor: 23.4 min, t_R major: 29.4 min, 92:8 er; ν_{max} (film) 2924 (C-H), 1717 (C=O), 1613 (C=O), 1483 (C_{Ar}-C_{Ar}); ^{1}H NMR (500 MHz, $CDCl_3$) δ_H: 2.41 (3H, s, COC*H*$_3$), 3.14 (1H, dd, *J* 18.2, 5.6, H–4a), 3.62 (1H, dd, *J* 18.1, 10.2, H–4b), 3.94 (1H, t, *J* 6.5, H–2), 3.99–4.02 (1H, m, H–3), 4.62 (1H, d, *J* 6.5, H–1), 6.80–6.84 (2H, m, H–Ar_F), 7.00–7.02 (1H, m, H–Ar_F), 7.28–7.30 (2H, m, H-azaAr), 7.44–7.48 (3H, m, H–Ph), 7.50–7.52 (1H, m, H-azaAr), 7.67–7.68 (2H, m, H–Ph), 8.32–8.34 (1H, m, H-azaAr); ^{19}F NMR (282 MHz, $CDCl_3$) δ_F: –114.3; ^{13}C NMR (126 MHz, $CDCl_3$) δ_C: 30.4 (CO-*C*H_3), 42.0 (*C*H_2), 42.5 (*C*H-3), 42.8 (*C*H-2), 50.5 (*C*H-1), 104.5 (O=C-*C*=C), 110.0 (d, $^2J_{CF}$ = 23.3, C_{Ar}-F), 110.1 (*C*H-Ar_F), 114.4 (*C*H-Ar_F), 114.5 (d, $^2J_{CF}$ = 22.6, C_{Ar}-F), 118.6 (*C*H-azaAr), 122.0 (*C*H-azaAr), 124.4 (*C*H-Ar_F), 124.4 (d, $^3J_{CF}$ = 8.9, C_{Ar}-F), 126.1 (*C*H-azaAr), 126.9 (*C*H-azaAr), 127.4 (2×*C*H-Ph), 128.7 (2×*C*H-Ph), 127.8 (*C*-azaAr), 130.7 (*C*H-Ph), 136.2 (*C*-Ph), 139.4 (*C*-azaAr), 146.0 (*C*1-Ar), 146.1 (*C*6-Ar), 155.6 (O=C-C=*C*), 162.0 (*C*-F), 163.0 (d, $^1J_{CF}$ = 245.9, C_{Ar}-F), 169.7 (CH-*C*=ON), 191.2 (*C*=OPh), 207.9 (*C*=OCH_3); *m/z* (NSI^+) 508 ($[M+K]^+$, 100%); HRMS (NSI^+) $C_{28}H_{20}O_3N_1F_1Na_1S_1$ $[M+Na]^+$, found 492.1030, requires 492.1040 (–2.1 ppm).

(6a*R*,11*S*,11a*R*)-6-Benzoyl-11-(2-oxo-2-phenylethyl)-11,11a-dihydrobenzo[4,5]thiazolo[3,2-*a*]indeno[1,2-*d*]pyridin-12(6a*H*)-one (38a)

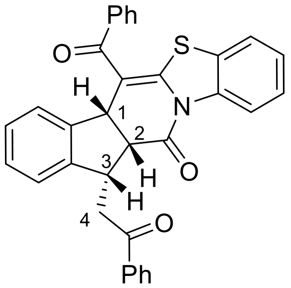

Following General Procedure 10, the corresponding TCP-ester (45.8 mg, 0.1 mmol), 2-phenacylbenzothiazole (50.6 mg, 0.2 mmol) and PS-BEMP (2.0 mmol/g loading, 90.0 mg, 0.2 mmol) in THF (0.25 mL) and subsequent chromatography (90:10 Petrol : EtOAc, R_f 0.3) afforded the title compound (42.6 mg, 83%) as a yellow solid. mp 262–264 °C; $[\alpha]_D^{20}$ +183.5 (*c* 0.2, $CHCl_3$); Chiral HPLC analysis, Chiralpak AS-H (90:10 hexane : IPA, flow rate 0.5 mLmin^{-1}, 211 nm, 40 °C) t_R major: 38.2 min, t_R minor: 43.3 min, 97:4 er; ν_{max} (film) 2926 (C-H), 1715 (C=O), 1684 (C=O), 1487 (C_{Ar}-C_{Ar}); ^{1}H NMR (500 MHz, $CDCl_3$) δ_H: 3.71 (1H, dd, *J* 17.3, 4.5, H–4a), 4.07 (1H, t, *J* 6.2, H–2), 4.20–4.26 (1H, m, H–3), 4.29 (1H, dd, *J* 17.3, 8.4, H–4b), 4.74 (1H, d, *J* 6.2, H–1), 7.12–7.24 (6H, m, 5×H–Ph + H-azaAr), 7.25–7.28 (1H, m, H-azaAr), 7.43–7.51 (4H, m, H–Ar), 7.69–7.71 (2H, m, H-azaAr), 8.31 (1H, d, *J* 6.8, H-azaAr); ^{13}C NMR (126 MHz, $CDCl_3$) δ_C: 30.5 (CO-*C*H_3), 42.0 (*C*H_2), 42.7 (*C*H-3), 43.0 (*C*H-2), 50.1 (*C*H-1), 104.6 (O=C-*C*=C), 118.5 (*C*H-aAr), 121.8 (*C*H-aAr), 122.1 (*C*H-azaAr), 123.2 (*C*H-Ar), 125.9 (*C*H-Ar), 126.7 (*C*H-azaAr), 127.4 (2×*C*H-Ph), 127.6 (*C*H-Ar), 127.7 (*C*-aAr), 127.8 (*C*H-Ar), 128.6 (2×*C*H-Ph), 130.5 (*C*H-Ph), 136.2 (*C*-Ph), 139.4 (*C*-azaAr), 143.3 (*C*1-Ar), 143.6 (*C*6-Ar), 155.4 (O=C-C=*C*), 169.8 (CH-*C*=ON), 191.3 (*C*=OPh), 208.2 (*C*=OCH_3); *m/z* (NSI$^+$) 514 ([M+H]$^+$, 100%); HRMS (NSI$^+$) $C_{33}H_{24}O_3N_1S_1$ [M+H]$^+$, found 514.1458, requires 514.1471 (–2.6 ppm).

(6a*R*,11*S*,11a*R*)-6-Benzoyl-3-fluoro-11-(2-oxo-2-phenylethyl)-11,11a-dihydrobenzo[4,5]thiazolo-[3,2-*a*]indeno[1,2-*d*]pyridin-12(6a*H*)-one (39a)

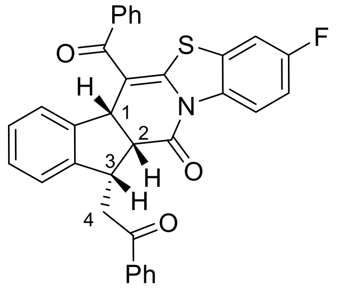

Following General Procedure 10, the corresponding TCP-ester (45.8 mg, 0.1 mmol), 2-phenacyl(6-fluoro)benzothiazole (54.2 mg, 0.2 mmol) and PS-BEMP (2.0 mmol/g loading, 90.0 mg, 0.2 mmol) in THF (0.25 mL) and subsequent chromatography (80:20 Petrol : EtOAc, R_f 0.25) afforded the title compound (31.9 mg, 60%) as a yellow solid. mp 238–240 °C; $[\alpha]_D^{20}$ +137.2 (*c* 1.0, $CHCl_3$); Chiral HPLC analysis, Chiralpak ADH (90:10 hexane : IPA, flow rate 1.0 mLmin^{-1}, 211 nm, 40 °C) t_R major: 45.7 min, t_R minor: 50.2 min, 96:4 er; ν_{max} (film) 2924 (C-H), 1712 (C=O), 1684 (C=O), 1487 (C_{Ar}-C_{Ar}); ^{1}H NMR (500 MHz, $CDCl_3$) δ_H: 3.71 (1H, dd, *J* 16.5, 3.7, H–4a), 4.08 (1H, t, *J* 6.2, H–2), 4.22–4.27 (1H, m, H–3), 4.29 (1H, dd, *J* 16.7, 9.6, H–4b), 4.75 (1H, d, *J* 6.2, H–1), 6.88–6.92 (1H, m, H-azaAr), 7.11–7.13 (1H, m, H-azaAr), 7.17–7.25 (4H, m, H–Ar), 7.45–7.47 (3H, m, H–Ph), 7.53–7.56 (2H, m, H–Ph), 7.62–7.63 (1H, m, H–Ph), 7.72–7.74 (2H, m, H–Ph), 8.15 (2H, d, *J* 7.4, H–Ph), 8.22 (1H, dd, *J* 9.2, 4.6, H-azaAr); ^{19}F NMR (282 MHz, $CDCl_3$) δ_F: –115.2; ^{13}C NMR (126 MHz, $CDCl_3$) δ_C: 37.6 (*C*H_2), 43.1 (*C*H-3), 43.3 (*C*H-2), 50.5 (*C*H-1), 105.1 (O=C-*C*=C), 108.9 (d, $^2J_{CF}$ = 26.5, C_{Ar}-F), 109.0 (*C*H-azaAr$_F$), 113.8 (d, $^2J_{CF}$ = 23.1, C_{Ar}-F), 113.9 (*C*H-azaAr$_F$), 119.8 (*C*H-azaAr$_F$), 122.4 (*C*H-Ar), 123.4 (*C*H-Ar), 127.4 (2×*C*H-Ph), 127.8 (*C*H-Ar), 128.0 (*C*H-Ar), 128.3 (2×*C*H-Ph), 128.7 (2×*C*H-Ph), 128.8 (2×*C*H-Ph), 129.9 (*C*-azaAr), 129.9 (d, $^3J_{CF}$ = 9.1, C_{Ar}-F), 132.6 (*C*-azaAr), 133.3 (*C*H-Ph), 137.2 (*C*-Ph), 139.3 (*C*-Ph), 143.3 (*C*1-Ar), 143.9 (*C*6-Ar), 155.4 (O=C-C=*C*), 159.4 (*C*-F), 160.4 (d, $^1J_{CF}$ = 247.3, C_{Ar}-F), 169.7 (CH-*C*=ON), 191.4 (*C*=OPh), 199.4 (CH_2*C*=OPh); *m/z* (NSI^+) 532 ($[M+H]^+$, 100%), 554 ($[M+Na]^+$, 85%); HRMS (NSI^+) $C_{33}H_{23}O_3N_1F_1S_1$ $[M+H]^+$, found 566.1366, requires 532.1377 (–2.1 ppm).

(6a*R*,11*S*,11a*R*)-6-Benzoyl-3-bromo-11-(2-oxo-2-phenylethyl)-11,11a-dihydrobenzo[4,5]thiazolo-[3,2-*a*]indeno[1,2-*d*]pyridin-12(6a*H*)-one (40a)

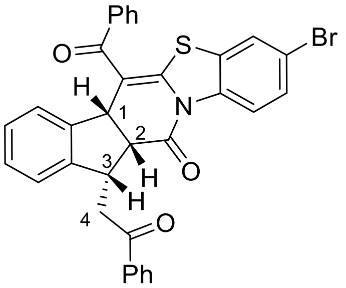

Following General Procedure 10, the corresponding TCP-ester (45.8 mg, 0.1 mmol), 2-phenacyl(6-bromo)benzothiazole (66.4 mg, 0.2 mmol) and PS-BEMP (2.0 mmol/g loading, 90.0 mg, 0.2 mmol) in THF (0.25 mL) and subsequent chromatography (80:20 Petrol : EtOAc, R_f 0.25) afforded the title compound (27.8 mg, 47%) as a yellow solid. mp 262–264 °C; $[\alpha]_D^{20}$ +105.7 (*c* 0.3, $CHCl_3$); Chiral HPLC analysis, Chiralpak ADH (85:15 hexane : IPA, flow rate 1.0 $mLmin^{-1}$, 211 nm, 40 °C) t_R major: 36.9 min, t_R minor: 43.6 min, 92.5:7.5 er; ν_{max} (film) 2872 (C-H), 1715 (C=O), 1686 (C=O), 1501 (C_{Ar}-C_{Ar}); ^1H NMR (500 MHz, $CDCl_3$) δ_H: 3.71 (1H, dd, *J* 16.9, 4.3, H–4a), 4.08 (1H, t, *J* 6.7, H–2), 4.21–4.25 (1H, m, H–3), 4.27 (1H, dd, *J* 16.9, 9.4, H–4b), 4.75 (1H, d, *J* 6.8, H–1), 7.11–7.25 (4H, m, H–Ar), 7.30 (1H, dd, *J* 8.9, 2.1, H-azaAr_{Br}), 7.45–7.47 (3H, m, H–Ph), 7.53–7.57 (3H, m, H–Ph), 7.62–7.65 (1H, m, H-azaAr_{Br}), 7.72–7.74 (2H, m, H–Ph), 8.10–8.12 (1H, H-azaAr_{Br}), 8.14–8.15 (2H, H–Ph; ^{13}C NMR (126 MHz, $CDCl_3$) δ_C: 37.6 (CH_2), 43.1 (*C*H-3), 43.2 (*C*H-2), 50.7 (*C*H-1), 105.1 (O=C-*C*=C), 119.0 (*C*-Br), 119.7 (*C*H-azaAr_{Br}), 122.5 (*C*H-Ar), 123.4 (*C*H-Ar), 124.5 (*C*H-Ar), 127.6 (2×*C*H-Ph), 127.8 (*C*H-Ar), 128.1 (*C*H-azaAr_{Br}), 128.3 (2×*C*H-Ph), 128.7 (2×*C*H-Ph), 128.8 (2×*C*H-Ph), 129.8 (*C*H-azaAr_{Br}), 130.2 (*C*-azaAr), 130.9 (*C*H-Ph), 133.4 (*C*H-Ph), 135.5 (*C*-azaAr_{Br}), 137.2 (*C*-Ph), 139.3 (*C*-Ph), 143.2 (*C*1-Ar), 143.9 (*C*6-Ar), 154.9 (O=C-C=*C*), 169.8 (CH-*C*=ON), 191.5 (*C*=OPh), 199.4 (CH_2*C*=OPh); *m/z* (NSI^+) 1185 ($[2M+H]^+$, 100%); HRMS (NSI^+) $C_{33}H_{23}O_3N_1Br_1S_1$ $[M+H]^+$, found 592.0570, requires 592.0577 (–1.1 ppm).

(6a*R*,11*S*,11a*R*)-6-Benzoyl-3-methoxy-11-(2-oxo-2-phenylethyl)-11,11a-dihydrobenzo[4,5]thiazolo-[3,2-*a*]indeno[1,2-*d*]pyridin-12(6a*H*)-one (41a)

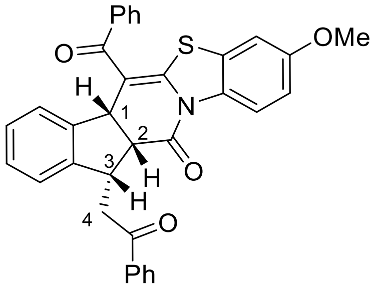

Following General Procedure 10, the corresponding TCP-ester (45.8 mg, 0.1 mmol), 2-phenacyl(6-methoxy)benzothiazole (56.6 mg, 0.2 mmol) and PS-BEMP (2.0 mmol/g loading, 90.0 mg, 0.2 mmol) in THF (0.25 mL) and subsequent chromatography (80:20–70:30 Petrol : EtOAc, R_f 0.2) afforded the title compound (11.3 mg, 20%) as a yellow solid. mp 240–241 °C; $[\alpha]_D^{20}$ +161.2 (*c* 0.3, $CHCl_3$); Chiral HPLC analysis, Chiralpak IB (93:7 hexane : IPA, flow rate 1.0 mLmin^{-1}, 254 nm, 40 °C) t_R minor: 33.1 min, t_R major: 46.6 min, 91:9 (recryst. 98:2) er; ν_{max} (film) 2934 (C-H), 1707 (C=O), 1684 (C=O), 1485 (C_{Ar}-C_{Ar}); ^{1}H NMR (500 MHz, $CDCl_3$) δ_H: 3.70 (1H, dd, *J* 17.5, 4.9, H–4a), 3.79 (3H, s, OC*H*$_3$), 4.05 (1H, t, *J* 6.4, H–2), 4.20–4.28 (1H, m, H–3), 4.30 (1H, dd, *J* 17.5, 9.1, H–4b), 4.73 (1H, d, *J* 6.8, H–1), 6.74 (1H, dd, *J* 9.2, 2.6, H-azaAr$_{OMe}$), 6.98 (1H, d, *J* 2.6, H-azaAr$_{OMe}$), 7.12–7.24 (4H, m, H–Ar), 7.44–7.46 (3H, m, H–Ph), 7.53–7.56 (3H, m, H–Ph), 7.61–7.64 (1H, m, H-azaAr$_{OMe}$), 7.72–7.74 (2H, m, H–Ph), 8.15–8.16 (2H, H–Ph); ^{13}C NMR (126 MHz, $CDCl_3$) δ_C: 37.7 (*C*H$_2$), 43.1 (*C*H-3), 43.3 (*C*H-2), 50.5 (*C*H-1), 55.8 (O*C*H$_3$), 104.6 (O=C-*C*=C), 106.2 (*C*H-azaAr$_{OMe}$), 113.2 (*C*H-azaAr$_{OMe}$), 119.4 (*C*H-azaAr$_{OMe}$), 122.4 (*C*H-Ar), 123.4 (*C*H-Ar), 127.5 (2×*C*H-Ph), 127.7 (*C*H-Ar), 127.9 (*C*H-Ar), 128.4 (2×*C*H-Ph), 128.7 (2×*C*H-Ph), 128.8 (2×*C*H-Ph), 129.3 (*C*-azaAr$_{OMe}$), 130.2 (*C*-azaAr$_{OMe}$), 130.6 (*C*H-Ph), 133.3 (*C*H-Ph), 137.3 (*C*-Ph), 139.6 (*C*-Ph), 143.5 (*C*1-Ar), 144.0 (*C*6-Ar), 155.9 (O=C-C=*C*), 157.7 (*C*-OMe), 169.5 (CH-*C*=ON), 191.2 (*C*=OPh), 199.5 (CH$_2$*C*=OPh); *m/z* (NSI$^+$) 1185 ([M+H]$^+$, 40%); HRMS (NSI$^+$) $C_{34}H_{26}O_4N_1S_1$ [M+H]$^+$, found 544.1566, requires 544.1577 (+2.0 ppm).

(6a*R*,11*S*,11a*R*)-6-(4-Fluorobenzoyl)-11-(2-oxo-2-phenylethyl)-11,11a-dihydrobenzo[4,5]thiazolo-[3,2-*a*]indeno[1,2-*d*]pyridin-12(6a*H*)-one (42a)

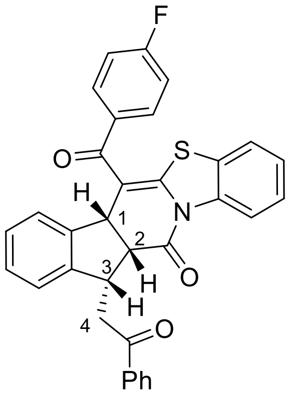

Following General Procedure 10, the corresponding TCP-ester (45.8 mg, 0.1 mmol), 2-(4-fluoro) phenacyl benzothiazole (54.2 mg, 0.2 mmol) and PS-BEMP (2.0 mmol/g loading, 90.0 mg, 0.2 mmol) in THF (0.25 mL) and subsequent chromatography (80:20 Petrol : EtOAc, R_f 0.25) afforded the title compound (35.6 mg, 67%) as a yellow solid. mp 259–261 °C; $[\alpha]_D^{20}$ +94.0 (*c* 0.2, $CHCl_3$); Chiral HPLC analysis, Chiralpak IB (95:5 hexane : IPA, flow rate 1.5 mLmin^{-1}, 211 nm, 40 °C) t_R major: 23.8 min, t_R minor: 37.1 min, 94.5:5.5 er; ν_{max} (film) 2920 (C-H), 1728 (C=O), 1678 (C=O), 1477 (C_{Ar}-C_{Ar}); ^{1}H NMR (500 MHz, $CDCl_3$) δ_H: 3.73 (1H, dd, *J* 17.0, 4.4, H–4a), 4.07 (1H, t, *J* 6.2, H–2), 4.23–4.30 (1H, m, H–3), 4.31 (1H, dd, *J* 17.0, 9.7, H–4b), 4.71 (1H, d, *J* 6.8, H–1), 7.10–7.17 (4H, m, Ar*H*), 7.18–7.24 (4H, m, 2×H–Ph + 2×H–Ph_F), 7.48–7.50 (1H, m, H-azaAr), 7.54–7.57 (2H, m, H-azaAr), 7.62–7.65 (1H, m, H-azaAr), 7.72–7.78 (2H, m, H–Ph_F), 8.15–8.17 (2H, m, H–Ph) 8.25–8.27 (1H, m, H-azaAr); ^{19}F NMR (282 MHz, $CDCl_3$) δ_F: –109.0; ^{13}C NMR (126 MHz, $CDCl_3$) δ_C: 37.6 (*C*H_2), 43.1 (*C*H-3), 43.3 (*C*H-2), 50.8 (*C*H-1), 104.5 (O=C-*C*=C), 115.7 (*C*H-Ph_F), 115.8 (d, $^2J_{CF}$ = 21.7, C_{Ph}-F), 115.9 (*C*H-Ph_F), 118.6 (*C*H-azaAr), 121.9 (*C*H-azaAr), 122.5 (*C*H-azaAr), 123.3 (*C*H-Ar), 126.0 (*C*H-Ar), 126.9 (*C*H-azaAr), 127.7 (*C*-azaAr), 127.8 (*C*H-Ar), 128.1 (*C*H-Ar), 128.4 (2×*C*H-Ph), 128.8 (2×*C*H-Ph), 129.9 (*C*H-Ph_F), 130.0 (d, $^3J_{CF}$ = 8.6, C_{Ph}-F), 130.0 (*C*H-Ph_F), 133.3 (*C*H-Ph), 135.7 (*C*-Ph_F), 136.3 (*C*-Ph), 137.3 (*C*-azaAr), 143.3 (*C*1-Ar), 144.1 (*C*6-Ar), 156.0 (N-*C*=C), 164.1 (d, $^1J_{CF}$ = 251.3, C_{Ph}-F), 165.1 (Ph-*C*-F) 169.8 (CH-*C*=ON), 189.8 (*C*=OPh_F), 199.5 (*C*=OPh); *m/z* (NSI$^+$) 532 ([M+H]$^+$, 100%), 554 ([M+Na]$^+$, 15%) HRMS (NSI$^+$) $C_{33}H_{23}O_3N_1F_1S_1$ [M+H]$^+$, found 532.1369, requires 532.1377 (–1.5 ppm).

(6a*R*,11*S*,11a*R*)-6-(4-Bromobenzoyl)-11-(2-oxo-2-phenylethyl)-11,11a-dihydrobenzo[4,5]thiazolo-[3,2-*a*]indeno[1,2-*d*]pyridin-12(6a*H*)-one (43a)

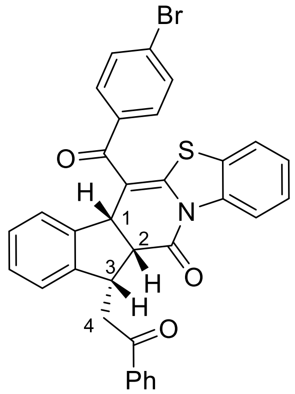

Following General Procedure 10, the corresponding TCP-ester (45.8 mg, 0.1 mmol), 2-(4-bromo) phenacyl benzothiazole (66.4 mg, 0.2 mmol) and PS-BEMP (2.0 mmol/g loading, 90.0 mg, 0.2 mmol) in THF (0.25 mL) and subsequent chromatography (80:20 Petrol : EtOAc, R_f 0.25) afforded the title compound (31.4 mg, 53%) as a yellow solid. mp 241–243 °C; $[\alpha]_D^{20}$ +137.5 (*c* 0.2, $CHCl_3$); Chiral HPLC analysis, Chiralpak IB (95:5 hexane : IPA, flow rate 1.5 $mLmin^{-1}$, 211 nm, 40 °C) t_R major: 26.6 min, t_R minor: 39.8 min, 92:8 er; ν_{max} (film) 2924 (C-H), 1714 (C=O), 1682 (C=O), 1481 (C_{Ar}-C_{Ar}); ^{1}H NMR (500 MHz, $CDCl_3$) δ_H: 3.72 (1H, dd, *J* 17.5, 4.7, H–4a), 4.08 (1H, t, *J* 6.3, H–2), 4.20–4.30 (1H, m, H–3), 4.31 (1H, dd, *J* 17.5, 9.7, H–4b), 4.67 (1H, d, *J* 6.7, H–1), 7.22–7.24 (4H, m, Ar*H*), 7.48–7.50 (1H, m, H-azaAr), 7.54–7.64 (8H, m, 3×H–Ph + 2×H–Ph_{Br} + 3×H-azaAr), 8.15–8.17 (2H, m, H–Ph) 8.25–8.27 (1H, m, H-azaAr); ^{13}C NMR (126 MHz, $CDCl_3$) δ_C: 37.6 (*C*H_2), 43.1 (*C*H-3), 43.2 (*C*H-2), 50.7 (*C*H-1), 104.3 (O=C-*C*=C), 118.6 (*C*H-azaAr), 121.9 (*C*H-azaAr), 122.5 (*C*H-azaAr), 123.2 (*C*H-Ar), 125.2 (*C*-Br), 126.0 (*C*H-Ar), 126.9 (*C*H-azaAr), 127.6 (*C*-azaAr), 127.8 (*C*H-Ar), 128.1 (*C*H-Ar), 128.4 (2×*C*H-Ph), 128.8 (2×*C*H-Ph), 129.2 (2×*C*H-Ph_{Br}), 131.9 (2×*C*H-Ph_{Br}), 133.3 (*C*H-Ph), 136.3 (*C*-Ph), 137.3 (*C*-Ph_{Br}), 138.3 (*C*-azaAr), 143.2 (*C*1-Ar), 144.0 (*C*6-Ar), 156.2 (N-*C*=C), 169.8 (CH-*C*=ON), 189.9 (*C*=OPh_{Br}), 199.4 (*C*=OPh); *m/z* (NSI^+) 1185 ($[2M+H]^+$, 100%); HRMS (NSI^+) $C_{33}H_{23}O_3N_1Br_1S_1$ $[M+H]^+$, found 592.0571, requires 592.0577 (–0.9 ppm).

(6a*R*,11*S*,11a*R*)-6-(4-Methoxybenzoyl)-11-(2-oxo-2-phenylethyl)-11,11a-dihydrobenzo[4,5]-thiazolo[3,2-*a*]indeno[1,2-*d*]pyridin-12(6a*H*)-one (44a)

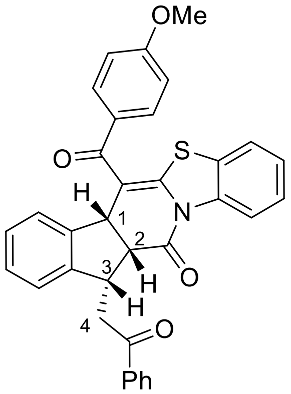

Following General Procedure 10, the corresponding TCP-ester (45.8 mg, 0.1 mmol), 2-(4-methoxy) phenacyl benzothiazole (56.6 mg, 0.2 mmol) and PS-BEMP (2.0 mmol/g loading, 90.0 mg, 0.2 mmol) in THF (0.25 mL) and subsequent chromatography (80:20→70:30 Petrol : EtOAc, R_f 0.20) afforded the title compound (27.7 mg, 51%) as a yellow solid. mp 225–227 °C; $[\alpha]_D^{20}$ +129.0 (*c* 0.2, $CHCl_3$); Chiral HPLC analysis, Chiralpak AS-H (90:10 hexane : IPA, flow rate 0.5 mLmin^{-1}, 220 nm, 40 °C) t_R major: 53.3 min, t_R minor: 60.7 min, 94.5:5.5 er; ν_{max} (film) 2928 (C-H), 1709 (C=O), 1686 (C=O), 1483 (C_{Ar}-C_{Ar}); ^{1}H NMR (500 MHz, $CDCl_3$) δ_H: 3.70–3.75 (1H, m, H–4a), 4.05 (1H, t, *J* 6.4, H–2), 4.25–4.28 (1H, m, H–3), 4.31 (1H, dd, *J* 17.8, 9.4, H–4b), 4.80 (1H, d, *J* 6.8, H–1), 6.95 (1H, d, *J* 8.8, 2×H–Ph_{OMe}), 7.13–7.18 (2H, m, H–Ph), 7.20–7.25 (4H, m, Ar*H*), 7.46–7.48 (1H, m, H-azaAr), 7.53–7.56 (2H, H-azaAr), 7.62–7.63 (1H, m, H–Ph), 7.76 (1H, d, *J* 8.8, 2×H–Ph_{OMe}), 8.11–8.19 (2H, m, H–Ph) 8.20–8.29 (1H, m, H-azaAr); ^{13}C NMR (126 MHz, $CDCl_3$) δ_C: 37.7 (*C*H_2), 43.1 (*C*H-3), 43.5 (*C*H-2), 50.8 (*C*H-1), 55.5 (O-CH_3), 105.0 (O=C-*C*=C), 113.9 (2×*C*H–Ph_{OMe}), 118.5 (*C*H-azaAr), 121.8 (*C*H-azaAr), 122.5 (*C*H-azaAr), 123.5 (*C*H-Ar), 125.9 (*C*H-Ar), 126.7 (*C*H-azaAr), 127.7 (*C*H-Ar), 128.0 (*C*H-Ar), 128.4 (2×*C*H-Ph), 128.8 (2×*C*H-Ph), 129.8 (2×*C*H-Ph_{OMe}), 130.1 (*C*-azaAr), 131.9 (*C*-Ph_{OMe}), 133.3 (*C*H-Ph), 136.4 (*C*-Ph), 137.3 (*C*-azaAr), 143.5 (*C*1-Ar), 144.1 (*C*6-Ar), 155.1 (N-*C*=C), 161.7 (*C*-Ph_{OMe}), 169.9 (CH-*C*=ON), 190.2 (*C*=OPh_{OMe}), 199.5 (*C*=OPh); *m/z* (NSI$^+$) 544 ([M+H]$^+$, 98%), 566 ([M+Na]$^+$, 30%); HRMS (NSI$^+$) $C_{34}H_{25}O_4N_1Na_1S_1$ [M+Na]$^+$, found 566.1385, requires 566.1397 (–2.0 ppm).

5.3 Formation of pre-cyclised dihydropyridone 51

(*R*,*E*)-4-(4-Fluorobenzoyl)-3-(2-(3-oxobut-1-en-1-yl)phenyl)-2,3-dihydro-1*H*-benzo[4,5]thiazolo[3,2-*a*]pyridin-1-one (51)

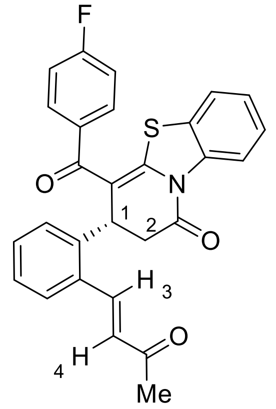

Following General Procedure 10, the corresponding TCP-ester (39.6 mg, 0.1 mmol), 2-(4-fluoro)phenacylbenzothiazole (54.2 mg, 0.2 mmol) and PS-BEMP (70.0 mg, 0.15 mmol) in THF (0.25 mL) and subsequent chromatography (70:30 Petrol : EtOAc, R_f 0.2) afforded the title compound (36.2 mg, 77%) as a yellow solid. mp 98–101 °C; $[\alpha]_D^{20}$ +117.4 (*c* 0.2, $CHCl_3$); Chiral HPLC analysis, Chiralpak AS-H (95:5 hexane : IPA, flow rate 1.5 mLmin^{-1}, 220 nm, 40 °C) t_R major: 23.3 min, t_R minor: 31.5 min, 88:12 er; ν_{max} (film) 2926 (C-H), 1726 (C=O), 1599 (C=O), 1481 (C_{Ar}-C_{Ar}); ^{1}H NMR (500 MHz, $CDCl_3$) δ_H: 2.33 (OC*H*$_3$), 2.85 (1H, dd, *J* 15.8, 2.3, H–2a), 3.28 (1H, dd, *J* 15.8, 7.4, H–2b), 4.61 (1H, dd, *J* 7.4, 2.3, H–2), 6.65 (1H, d, *J* 15.6, H–4), 6.91–6.93 (2H, m, H–Ph_F), 7.13–7.18 (4H, m, Ar*H*), 7.29–7.32 (2H, m, H–Ph_F), 7.36–7.39 (2H, m, H-azaAr), 7.55 (1H, d, *J* 15.6, H–3), 7.62–7.64 (1H, m, H-azaAr), 8.44–8.46 (1H, m, H-azaAr); ^{19}F NMR (282 MHz, $CDCl_3$) δ_F: –109.1; ^{13}C NMR (126 MHz, $CDCl_3$) δ_C: 29.2 (O*C*H$_3$), 35.4 (*C*H-1), 40.7 (*C*H$_2$), 107.1 (O=C-*C*=C), 115.4 (*C*H-Ph_F), 115.5 (*C*H-Ph_F), 115.5 (d, $^2J_{CF}$ = 21.7, C_{Ph}-F), 117.6 (*C*H-azaAr), 122.2 (*C*H-azaAr), 126.2 (*C*H-azaAr), 127.0 (*C*H-Ar), 127.4 (*C*H-azaAr), 127.7 (*C*-azaAr), 128.5 (2×*C*H-Ar), 128.5 (d, $^4J_{CF}$ = 4.0, C_{Ph}-F), 129.1 (*C*H-Ar), 129.1 (*C*H-Ph_F), 129.1 (d, $^3J_{CF}$ = 8.6, C_{Ph}-F), 129.7 (*C*H-Ph_F), 131.3 (*C*-4), 132.8 (*C*-azaAr), 135.5 (*C*1-Ar), 135.5 (*C*6-Ar), 136.0 (C-Ph_F), 139.8 (*C*-azaAr), 157.3 (N-*C*=C), 163.9 (d, $^1J_{CF}$ = 251.2, C_{Ph}-F), 164.9 (Ph-*C*-F), 167.3 (CH-*C*=ON), 190.0 (*C*=OPh_F), 197.4 (*C*=OMe); *m/z* (NSI$^+$) 470 ([M+H]$^+$, 70%); HRMS (NSI$^+$) $C_{28}H_{21}O_3N_1F_1S_1$ [M+H]$^+$, found 470.1212, requires 470.1221 (–1.8 ppm).

(6a*R*,11*S*,11a*R*)-6-(4-Fluorobenzoyl)-11-(2-oxopropyl)-11,11a-dihydrobenzo[4,5]thiazolo[3,2-*a*]indeno[1,2-*d*]pyridin-12(6a*H*)-one (52)

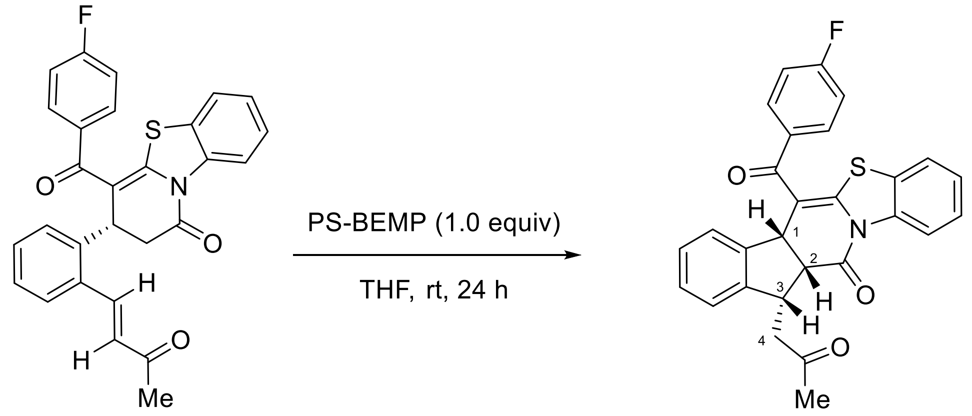

51 **52**

Pre-cyclisation intermediate **51** (36.2 mg, 0.08 mmol, 1.0 equiv) and PS-BEMP (2.0 mmol/g loading, 40 mg, 1.0 equiv) in THF were stirred 24 h at rt. Subsequent chromatography (80:20 Petrol : EtOAc, R_f 0.25) afforded the title compound (31.0 mg, 86%) as a yellow solid. mp 190–192 °C; $[\alpha]_D^{20}$ +128.0 (*c* 0.2, $CHCl_3$); Chiral HPLC analysis, Chiralpak IB (97:3 hexane : IPA, flow rate 1.5 mLmin^{-1}, 211 nm, 40 °C) t_R major: 25.9 min, t_R minor: 43.1 min, 92:8 er; ν_{max} (film) 2923 (C-H), 1735 (C=O), 1677 (C=O), 1452 (C_{Ar}-C_{Ar}); ^{1}H NMR (500 MHz, $CDCl_3$) δ_H: 2.45 (OC*H*$_3$), 3.24 (1H, dd, *J* 18.1, 5.5, H–4a), 3.65 (1H, dd, *J* 18.1, 10.5,, H–4b), 3.97 (1H, t, *J* 6.5,, H–2), 4.00–4.20 (1H, m, H–3), 4.66 (1H, d, *J* 6.8, H–1), 7.09–7.20 (6H, m, 4×Ar*H* + 2×H–Ph_F), 7.24–7.32 (2H, H-azaAr), 7.53–7.54 (1H, m, H-azaAr), 7.74–7.77 (2H, m, H–Ph_F), 8.33–8.35 (1H, m, H-azaAr); ^{19}F NMR (282 MHz, $CDCl_3$) δ_F: –109.0; ^{13}C NMR (126 MHz, $CDCl_3$) δ_C: 30.6 (O*C*H$_3$), 42.1 (*C*H$_2$), 42.9 (*C*H-3), 43.2 (*C*H-2), 50.2 (*C*H-1), 104.5 (O=C-*C*=C), 115.8 (d, $^2J_{CF}$ = 21.8, C_{Ph}-F), 115.7 (*C*H-Ph_F), 115.9 (*C*H-Ph_F), 118.6 (*C*H-azaAr), 122.0 (*C*H-azaAr), 122.3 (*C*H-azaAr), 123.2 (*C*H-Ar), 126.1 (*C*H-Ar), 126.9 (*C*H-azaAr), 127.1 (*C*-azaAr), 127.8 (*C*H-Ar), 128.1 (*C*H-Ar), 129.9 (d, $^3J_{CF}$ = 8.5, C_{Ph}-F), 129.9 (*C*H-Ph_F), 130.0 (*C*H-Ph_F), 136.2 (*C*-Ph_F), 138.8 (*C*-azaAr), 140.6 (*C*1-Ar), 143.7 (*C*6-Ar), 157.8 (N-*C*=C), 169.8 (CH-*C*=ON), 188.1 (*C*=OPh_F), 208.3 (*C*=OMe); *m/z* (NSI$^+$) 492 ([M+H]$^+$, 100 %); HRMS (NSI$^+$) $C_{28}H_{20}O_3N_1F_1S_1Na$ [M+Na]$^+$, found 492.1031, requires 492.1021 (–1.9 ppm).

6 Michael-Lactamization-Michael with Acyl Benzimidazoles

6.1 Data for racemic indanes (±) 54-69

12a-Benzoyl-12-(2-oxopropyl)-7,7a,12,12a-tetrahydro-6*H*-benzo[4,5]imidazo[1,2-*a*]indeno[2,1-*c*]pyridin-6-one (±54)

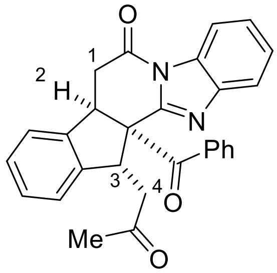

Following General Procedure 11, the corresponding TCP-ester (39.6 mg, 0.1 mmol), 2-phenacylbenzimidazole (35.5 mg, 0.15 mmol) and PS-BEMP (2.0 mmol/g loading, 75.0 mg) in CH_2Cl_2 (0.5 mL) and subsequent chromatography (80:20 Petrol : EtOAc, R_f 0.2) afforded the title compound (39.1 mg, 90 %) as a pink solid. mp 195–196 °C; ν_{max} (film) 2924 (C-H), 1721 (C=O), 1668 (C=O), 1450 (C_{Ar}-C_{Ar}); ^{1}H NMR (500 MHz, $CDCl_3$) δ_H: 1.99 (3H, s, COC*H*$_3$), 2.74–2.76 (2H, m, H–1a,b), 3.48 (1H, dd, *J* 18.3, 2.4, H–4a), 3.76 (1H, dd, *J* 18.3, 5.8, H–4b), 4.61 (1H, dd, *J* 5.8, 2.4, H–3), 5.42 (1H, dd, *J* 8.2, 5.8, H–2), 7.11–7.22 (4H, m, H–Ar), 7.25–7.28 (2H, m, H-azaAr), 7.45–7.48 (2H, m, H-Ph), 7.54 (1H, t, *J* 7.3, H-Ph), 7.65 (1H, dd, *J* 5.9, 3.2, H-azaAr), 8.12 (1H, dd, *J* 5.9, 3.2, H-azaAr), 8.54 (2H, d, *J* 7.3, H-Ph); ^{13}C NMR (126 MHz, $CDCl_3$) δ_C: 31.0 (CO-*C*H$_3$), 33.3 (*C*H$_2$-4), 44.0 (*C*H$_2$-1), 44.3 (*C*H-3), 49.0 (*C*H-2), 65.6 (*C*$_{quat}$), 115.8 (*C*H-aAr), 120.0 (*C*H-aAr), 122.7 (*C*H-Ar), 125.3 (*C*H-Ar), 125.8 (*C*H-azaAr), 126.3 (*C*H-azaAr), 128.2 (*C*H-Ar), 128.6 (*C*H-Ar), 128.6 (2×*C*H-Ph), 130.8 (2×*C*H-Ph), 131.6 (*C*-aAr), 133.9 (*C*H-Ph), 135.1 (*C*-Ph), 139.1 (*C*-azaAr), 142.3 (*C*1-Ar), 143.9 (*C*6-Ar), 152.8 (N=*C*(C)-N), 167.3 (CH-*C*=ON), 196.3 (*C*=OPh), 205.7 (*C*=OCH$_3$); *m/z* (NSI$^+$) 435 ([M+H]$^+$, 100%), 891 ([2M+Na]$^+$, 30%); HRMS (NSI$^+$) $C_{28}H_{23}O_3N_2$ [M+H]$^+$, found 435.1696, requires 435.1703 (–1.7 ppm).

12a-(4-Methoxybenzoyl)-12-(2-oxopropyl)-7,7a,12,12a-tetrahydro-6*H*-benzo[4,5]imidazo[1,2-*a*]indeno[2,1-*c*]pyridin-6-one (±55)

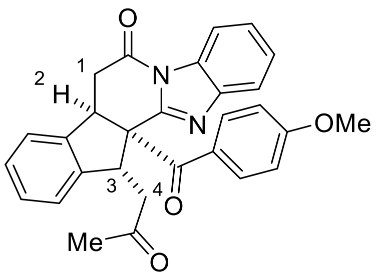

Following General Procedure 11, the corresponding TCP-ester (39.6 mg, 0.1 mmol), 2-(4-methoxy)phenacylbenzimidazole (39.9 mg, 0.15 mmol) and PS-BEMP (2.0 mmol/g loading, 75.0 mg) in THF (0.5 mL) and subsequent chromatography (80:20 – 70:30 Petrol : EtOAc, R_f 0.15) afforded the title compound (40.2 mg, 87 %) as a pale pink solid. mp 139–141 °C; ν_{max} (film) 2930 (C-H), 1721 (C=O), 1655 (C=O), 1452 (C_{Ar}-C_{Ar}); ^{1}H NMR (500 MHz, $CDCl_3$) δ_H: 1.99 (3H, s, COC*H*$_3$), 2.70–2.77 (2H, m, H–1a,b), 3.48 (1H, dd, *J* 18.3, 2.8, H–4a), 3.80 (1H, dd, *J* 18.3, 5.9, H–4b), 4.60 (1H, dd, *J* 5.9, 2.8, H–3), 5.41 (1H, t, *J* 7.1, H–2), 6.94 (2H, d, *J* 9.1, H-Ar_{OMe}), 7.07–7.17 (2H, m, H-azaAr), 7.20–7.27 (4H, m, H-Ar), 7.64–7.66 (1H, m, H-azaAr), 8.11–8.12 (1H, m, H-azaAr), 8.62 (2H, d, *J* 9.0, H-Ar_{OMe}); ^{13}C NMR (126 MHz, $CDCl_3$) δ_C: 31.1 (CO-*C*H$_3$), 33.3 (*C*H$_2$-4), 43.9 (*C*H$_2$-1), 44.2 (*C*H-3), 49.0 (*C*H-2), 55.6 (O*C*H$_3$), 65.6 (C_{quat}), 113.8 (2×*C*H-Ar_{OMe}), 115.7 (*C*H-azaAr), 119.9 (*C*H-azaAr), 122.6 (*C*H-azaAr), 125.2 (*C*H-azaAr), 125.7 (*C*H-Ar), 126.0 (*C*H-Ar), 127.9 (*C*-Ar_{OMe}), 128.1 (*C*H-Ar), 128.5 (*C*H-Ar), 131.6 (*C*-azaAr), 133.5 (2×*C*H-Ar_{OMe}), 139.2 (*C*-azaAr), 142.2 (*C*1-Ar), 144.0 (*C*6-Ar), 153.2 (N=*C*(C)-N), 164.0 (*C*-OMe), 167.5 (CH-*C*=ON), 194.2 (*C*=OAr_{OMe}), 205.8 (*C*=OCH$_3$); *m/z* (NSI$^+$) 465 ([M+H]$^+$, 100%); HRMS (NSI$^+$) $C_{29}H_{25}O_4N_2$ [M+H]$^+$, found 465.1805, requires 465.1809 (–0.8 ppm).

12a-(4-Nitrobenzoyl)-12-(2-oxopropyl)-7,7a,12,12a-tetrahydro-6*H*-benzo[4,5]imidazo[1,2-*a*]indeno[2,1-*c*]pyridin-6-one (±56)

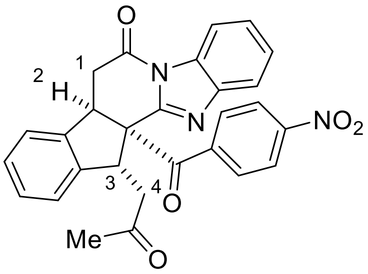

Following General Procedure 11, the corresponding TCP-ester (39.6 mg, 0.1 mmol), 2-(4-methoxy)phenacylbenzimidazole (42.2 mg, 0.15 mmol) and PS-BEMP (2.0 mmol/g loading, 75.0 mg) in THF (0.5 mL) and subsequent chromatography (80:20 – 70:30 Petrol : EtOAc, R_f 0.15) afforded the title compound (28.7 mg, 60 %) as an orange crystalline solid. mp 99–101 °C; ν_{max} (film) 2926 (C-H), 1721 (C=O), 1676 (C=O), 1528 (N-O), 1452 (C_{Ar}-C_{Ar}); ^{1}H NMR (500 MHz, $CDCl_3$) δ_H: 2.05 (3H, s, COC*H*$_3$), 2.73 (1H, dd, *J* 17.3, 3.8, H–1a), 2.82 (1H, dd, *J* 17.3, 10.1, H–1b), 3.55 (1H, dd, *J* 18.3, 2.7, H–4a), 3.68 (1H, dd, *J* 18.3, 5.9, H–4b), 4.67 (1H, dd, *J* 5.9, 2.7, H–3), 5.45 (1H, dd, *J* 10.1, 4.0, H–2), 7.17–7.29 (4H, m, H-Ar), 7.32–7.34 (2H, m, H-azaAr), 7.68–7.69 (1H, m, H-azaAr), 8.14–8.15 (1H, m, H-azaAr), 8.31 (2H, d, *J* 8.8, H-Ar_{NO2}), 8.72 (2H, d, *J* 8.8, H-Ar_{NO2}); ^{13}C NMR (126 MHz, $CDCl_3$) δ_C: 31.1 (CO-*C*H$_3$), 33.1 (*C*H$_2$-4), 44.1 (*C*H$_2$-1), 44.3 (*C*H-3), 49.4 (*C*H-2), 65.6 (C_{quat}), 115.7 (*C*H-azaAr), 120.0 (*C*H-azaAr), 122.8 (*C*H-azaAr), 123.5 (2×*C*H-Ar_{NO2}), 125.6 (*C*H-azaAr), 125.8 (*C*H-Ar), 126.1 (*C*H-Ar), 128.4 (*C*H-Ar), 128.9 (*C*H-Ar), 131.5 (*C*-azaAr), 131.9 (2×*C*H-Ar_{NO2}), 138.6 (*C*-azaAr), 140.0 (*C*-Ar_{NO2}), 142.1 (*C*1-Ar), 143.5 (*C*6-Ar), 150.4 (*C*-NO_2), 152.0 (N=*C*(C)-N), 166.8 (CH-*C*=ON), 195.8 (*C*=OAr_{NO2}), 205.4 (*C*=OCH$_3$); *m/z* (NSI^+) 480 ($[M+H]^+$, 100%); HRMS (NSI^+) $C_{28}H_{22}O_5N_3$ $[M+H]^+$, found 480.1551, requires 480.1554 (–0.6 ppm).

12a-(4-(Trifluoromethyl)benzoyl)-12-(2-oxopropyl)-7,7a,12,12a-tetrahydro-6*H*-benzo[4,5]imidazo[1,2-*a*]indeno[2,1-*c*]pyridin-6-one (±57)

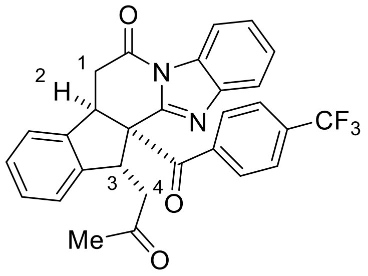

Following General Procedure 11, the corresponding TCP-ester (39.6 mg, 0.1 mmol), 2-(4-(trifluoromethyl)phenacylbenzimidazole (46.0 mg, 0.15 mmol) and PS-BEMP (2.0 mmol/g loading, 75.0 mg) in THF (0.5 mL) and subsequent chromatography (80:20 Petrol : EtOAc, R_f 0.2) afforded the title compound (24.4 mg, 49 %) as a pink solid. mp 71–72 °C; ν_{max} (film) 2928 (C-H), 1722 (C=O), 1674 (C=O), 1452 (C_{Ar}-C_{Ar}); ^{1}H NMR (500 MHz, $CDCl_3$) δ_H: 2.04 (3H, s, COC*H*$_3$), 2.71 (1H, dd, *J* 17.3, 3.7 Hz H–1a), 2.81 (1H, dd, *J* 17.3, 10.5, H–1b), 3.53 (1H, dd, *J* 18.3, 3.0, H–4a), 3.72 (1H, dd, *J* 18.3, 5.7, H–4b), 4.60 (1H, dd, *J* 5.7, 3.0, H–3), 5.45 (1H, dd, *J* 10.5, 3.7, H–2), 7.16–7.29 (4H, m, H-Ar), 7.31–7.33 (2H, m, H-azaAr), 7.67–7.69 (1H, m, H-azaAr), 7.75 (2H, d, *J* 8.4, H-Ar_{CF3}), 8.14–8.16 (1H, m, H-azaAr), 8.68 (2H, d, *J* 8.3, H-Ar_{CF3}); ^{19}F NMR (282 MHz, $CDCl_3$) δ_F: –63.3 (C*F*$_3$); ^{13}C NMR (126 MHz, $CDCl_3$) δ_C: 31.1 (CO-*C*H$_3$), 33.2 (*C*H$_2$-4), 44.0 (*C*H$_2$-1), 44.3 (*C*H-3), 49.2 (*C*H-2), 65.6 (C_{quat}), 115.8 (*C*H-azaAr), 120.0 (*C*H-azaAr), 122.7 (*C*H-azaAr), 125.5 (*C*H-azaAr), 125.5 (*C*H-Ar_{CF3}), 125.6 (*C*H-Ar_{CF3}), 125.6 (q, $^4J_{CF}$ = 3.6, C_{Ar}-CF_3), 125.9 (*C*H-Ar), 126.0 (*C*H-Ar), 127.2 (q, $^1J_{CF}$ = 294.7, C_{Ar}-CF_3), 128.3 (*C*H-Ar), 128.8 (*C*H-Ar), 131.3 (q, $^2J_{CF}$ = 40.3, C_{Ar}-CF_3), 131.2 (2×*C*H-Ar_{CF3}), 131.5 (*C*-azaAr), 137.9 (*C*-Ar_{CF3}), 138.8 (*C*-azaAr), 142.1 (*C*1-Ar), 143.6 (*C*6-Ar), 152.3 (N=*C*(C)-N), 167.0 (CH-*C*=ON), 195.8 (*C*=OAr_{CF3}), 205.5 (*C*=OCH$_3$); *m/z* (NSI$^+$) 503 ([M+H]$^+$, 100%); HRMS (NSI$^+$) $C_{29}H_{22}$ $O_3N_2F_3$ [M+H]$^+$, found 503.1567, requires 503.1577 (–2.0 ppm).

12a-(4-Fluorobenzoyl)-12-(2-oxopropyl)-7,7a,12,12a-tetrahydro-6*H*-benzo[4,5]imidazo[1,2-*a*]indeno[2,1-*c*]pyridin-6-one (±58)

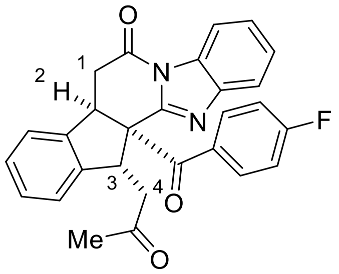

Following General Procedure 11, the corresponding TCP-ester (39.6 mg, 0.1 mmol), 2-(4-fluoro)phenacylbenzimidazole (38.1 mg, 0.15 mmol) and PS-BEMP (2.0 mmol/g loading, 75.0 mg) in THF (0.5 mL) and subsequent chromatography (80:20 Petrol : EtOAc, R_f 0.2) afforded the title compound (36.9 mg, 82 %) as a colorless crystalline solid. mp 211–212 °C; ν_{max} (film) 2926 (C-H), 1722 (C=O), 1666 (C=O), 1452 (C_{Ar}-C_{Ar}); ^{1}H NMR (500 MHz, $CDCl_3$) δ_H: 1.99 (3H, s, COC*H*$_3$), 2.67 (1H, dd, *J* 17.2, 3.6, H–1a), 2.74 (1H, dd, *J* 17.2, 10.6, H–1b), 3.49 (1H, dd, *J* 18.3, 2.8, H–4a), 3.75 (1H, dd, *J* 18.3, 5.8, H–4b), 4.61 (1H, dd, *J* 5.8, 2.8, H–3), 5.40 (1H, dd, *J* 10.6, 3.6, H–2), 7.10–7.18 (4H, m, H–Ar), 7.21–7.24 (2H, m, H-azaAr), 7.27–7.29 (2H, m, H-Ar_F), 7.65 (1H, dd, *J* 6.1, 3.2, H-azaAr), 8.12 (1H, dd, *J* 6.2, 3.2, H-azaAr), 8.66 (2H, dd, *J* 9.0, 5.4, H-Ar_F); ^{19}F NMR (282 MHz, $CDCl_3$) δ_F: –103.2; ^{13}C NMR (126 MHz, $CDCl_3$) δ_C: 31.0 (CO-*C*H$_3$), 33.3 (*C*H$_2$-4), 43.8 (*C*H$_2$-1), 44.1 (*C*H-3), 49.0 (*C*H-2), 65.5 (C_{quat}), 115.7 (d, $^2J_{CF}$ = 21.7, C_{Ph}-F), 115.7 (*C*H-azaAr), 119.8 (*C*H-azaAr), 122.5 (*C*H-Ar), 125.3 (*C*H-Ar_F), 125.7 (*C*H-azaAr), 125.8 (*C*H-azaAr), 128.1 (*C*H-Ar), 128.5 (*C*H-Ar), 131.3 (d, $^4J_{CF}$ = 3.0, C_{Ph}-F), 131.4 (*C*-azaAr), 133.7 (d, $^3J_{CF}$ = 9.3, C_{Ph}-F), 138.8 (*C*-azaAr), 142.0 (*C*1-Ar), 143.7 (*C*6-Ar), 152.6 (N=*C*(C)-N), 166.0 (d, $^1J_{CF}$ = 257.0, C_{Ph}-F), 167.1 (CH-*C*=ON), 194.5 (*C*=OAr_F), 205.4 (*C*=OCH$_3$); *m/z* (NSI$^+$) 453 ([M+H]$^+$); HRMS (NSI$^+$) $C_{28}H_{22}O_3N_2F$ [M+H]$^+$, found 453.1607, requires 453.1609 (–0.4 ppm).

12a-(4-Bromobenzoyl)-12-(2-oxopropyl)-7,7a,12,12a-tetrahydro-6*H*-benzo[4,5]imidazo[1,2-*a*]indeno[2,1-*c*]pyridin-6-one (±59)

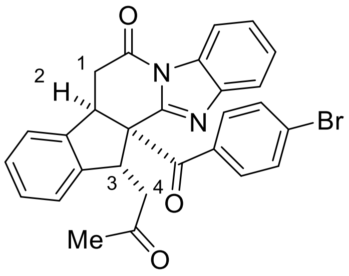

Following General Procedure 11, the corresponding TCP-ester (39.6 mg, 0.1 mmol), 2-(4-bromo)phenacylbenzimidazole (47.3 mg, 0.15 mmol) and PS-BEMP (2.0 mmol/g loading, 75.0 mg) in THF (0.5 mL) and subsequent chromatography (80:20 Petrol : EtOAc, R_f 0.2) afforded the title compound (37.8 mg, 74 %) as a yellow solid. mp 95–96 °C; ν_{max} (film) 2924 (C-H), 1721 (C=O), 1667 (C=O), 1451 (C_{Ar}-C_{Ar}); ^{1}H NMR (500 MHz, $CDCl_3$) δ_H: 2.00 (3H, s, COC*H*$_3$), 2.67 (1H, dd, *J* 17.3, 3.4, H–1a), 2.75 (1H, dd, *J* 17.1, 10.7, H–1b), 3.49 (1H, dd, *J* 18.3, 2.6, H–4a), 3.72 (1H, dd, *J* 18.3, 5.8, H–4b), 4.59–4.60 (1H, m, H–3), 5.39 (1H, dd, *J* 10.6, 3.2, H–2), 7.11–7.26 (4H, m, H-Ar), 7.27–7.29 (2H, m, H-azaAr), 7.61 (2H, d, *J* 8.7, H-Ar_{Br}), 7.62–7.66 (1H, m, H-azaAr), 8.10–8.12 (1H, m, H-azaAr), 8.46 (2H, d, *J* 8.6, H-Ar_{Br}); ^{13}C NMR (126 MHz, $CDCl_3$) δ_C: 31.1 (CO-*C*H_3), 33.2 (*C*H_2-4), 43.9 (*C*H_2-1), 44.2 (*C*H-3), 49.1 (*C*H-2), 65.6 (C_{quat}), 115.8 (*C*H-azaAr), 120.0 (*C*H-azaAr), 122.7 (*C*H-azaAr), 125.4 (*C*H-azaAr), 125.9 (2×*C*H-Ar), 128.3 (*C*H-Ar), 128.7 (*C*H-Ar), 129.5 (*C*-Br), 131.5 (*C*-azaAr), 131.9 (2×*C*H-Ar_{Br}), 132.4 (2×*C*H-Ar_{Br}), 133.7 (*C*-Ar_{Br}), 138.9 (*C*-azaAr), 142.1 (*C*1-Ar), 143.7 (*C*6-Ar), 152.5 (N=*C*(C)-N), 167.2 (CH-*C*=ON), 195.3 (*C*=OAr_{NO2}), 205.5 (*C*=OCH_3); *m/z* (NSI^+) 513 ($[M+H]^+$, 100%); HRMS (NSI^+) $C_{28}H_{22}O_3N_2Br$ $[M+H]^+$, found 513.0807, requires 513.0850 (–0.3 ppm).

12a-(3-Methylbenzoyl)-12-(2-oxopropyl)-7,7a,12,12a-tetrahydro-6*H*-benzo[4,5]imidazo[1,2-*a*]indeno[2,1-*c*]pyridin-6-one (±60)

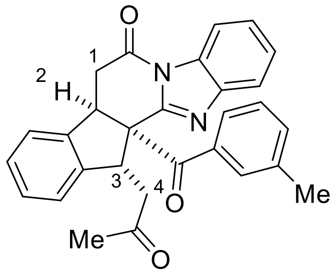

Following General Procedure 11, the corresponding TCP-ester (39.6 mg, 0.1 mmol), 2-(4-methoxy)phenacylbenzimidazole (37.5 mg, 0.15 mmol) and PS-BEMP (2.0 mmol/g loading, 75.0 mg) in CH_2Cl_2 (0.5 mL) and subsequent chromatography (80:20 Petrol : EtOAc, R_f 0.2) afforded the title compound (28.3 mg, 63 %) as a off-white solid. mp 51–52 °C; ν_{max} (film) 2920 (C-H), 1717 (C=O), 1663 (C=O), 1450 (C_{Ar}-C_{Ar}); ^{1}H NMR (500 MHz, $CDCl_3$) δ_H: 2.00 (3H, s, COC*H*$_3$), 2.40 (3H, s, Ar-C*H*$_3$), 2.72–2.79 (2H, m, H–1a,b), 3.48 (1H, dd, *J* 18.3, 3.0, H–4a), 3.74 (1H, dd, *J* 18.3, 5.9, H–4b), 4.61 (1H, dd, *J* 5.9, 3.0, H–3), 5.43 (1H, dd, *J* 8.3, 5.9, H–2), 7.10–7.24 (4H, m, H–Ar), 7.22–7.28 (2H, m, 1×H-azaAr + 1×H-Ar_{Me}), 7.34–7.36 (2H, m, H-Ar_{Me}), 7.64 (1H, dd, *J* 6.4, 2.9, H-azaAr), 8.12 (1H, dd, *J* 6.4, 2.9, H-azaAr), 8.24 (1H, s, H-Ar_{Me}), 8.40–8.42 (1H, m, H-azaAr); ^{13}C NMR (126 MHz, $CDCl_3$) δ_C: 21.5 (Ar-*C*H$_3$), 31.0 (CO-*C*H$_3$), 33.3 (*C*H$_2$-4), 44.0 (*C*H$_2$-1), 44.4 (*C*H-3), 49.0 (*C*H-2), 65.7 (C_{quat}), 115.7 (*C*H-azaAr), 119.9 (*C*H-azaAr), 122.6 (*C*H-Ar), 125.3 (*C*H-Ar), 125.7 (*C*H-azaAr), 125.9 (*C*H-azaAr), 128.0 (*C*H-Ar), 128.1 (*C*H-Ar), 128.4 (*C*H-Ar_{Me}), 128.5 (*C*H-Ar_{Me}), 131.1 (*C*H-Ar_{Me}), 131.5 (*C*-azaAr), 134.6 (*C*H-Ar_{Me}), 135.2 (C-Ar_{Me}), 138.4 (*C*-azaAr), 139.1 (C_{Ar}-CH$_3$), 142.3 (*C*1-Ar), 144.0 (*C*6-Ar), 145.0 (C_{Ar}-Me), 152.9 (N=*C*(C)-N), 167.3 (CH-*C*=ON), 196.4 (*C*=OAr_{Me}), 205.7 (*C*=OCH$_3$); *m/z* (NSI$^+$) 471 ([M+Na]$^+$, 100%); HRMS (NSI$^+$) $C_{29}H_{24}$ O_3N_2Na[M+Na]$^+$, found 471.1683, requires 471.1679 (+0.8 ppm).

12a-(Furan-2-carbonyl)-12-(2-oxopropyl)-7,7a,12,12a-tetrahydro-6*H*-benzo[4,5]imidazo[1,2-*a*]indeno[2,1-*c*]pyridin-6-one (±61)

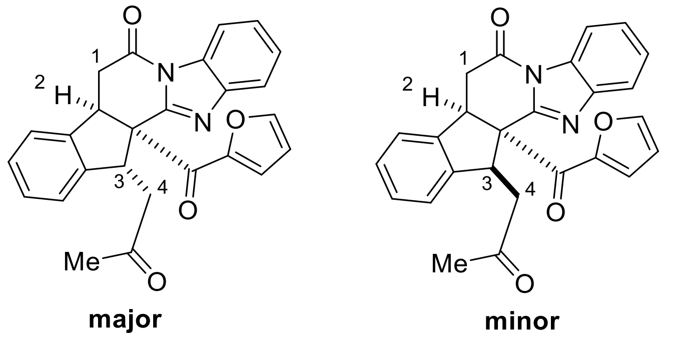

Following General Procedure 11, the corresponding TCP-ester (39.6 mg, 0.1 mmol), 2-furoylbenzimidazole (45.2 mg, 0.15 mmol) and PS-BEMP (2.0 mmol/g loading, 75.0 mg) in CH_2Cl_2 (0.5 mL) and subsequent chromatography (70:30 Petrol : EtOAc, R_f 0.2) afforded the title compound (28.4 mg, 68%) as a diastereomeric mixture (2.5:1 dr). The major diastereomer was separated from the minor one: brown solid was obtained (15.1 mg, 36%).

major: mp 69–70 °C; ν_{max} (film) 2924 (C-H), 1722 (C=O), 1655 (C=O), 1452 (C_{Ar}-C_{Ar}); ^{1}H NMR (500 MHz, $CDCl_3$) δ_H: 2.06 (3H, s, COC*H*$_3$), 2.85–2.86 (2H, m, H–1a,b), 3.44 (1H, dd, *J* 18.1, 3.9, H–4a), 3.66 (1H, dd, *J* 18.1, 5.8, H–4b), 4.55 (1H, dd, *J* 5.8, 3.9, H–3), 5.19 (1H, dd, *J* 7.7, 6.1, H–2), 6.55 (1H, dd, *J* 3.7, 1.7, H–Fur), 7.10–7.24 (4H, m, H–Ar), 7.30–7.32 (2H, m, H-azaAr), 7.58 (1H, dd, *J* 1.7, 0.7, H-Fur), 7.66–7.68 (1H, m, H-azaAr), 8.15–8.17 (2H, m, H-azaAr + H-Fur); ^{13}C NMR (126 MHz, $CDCl_3$) δ_C: 31.0 (CO-*C*H$_3$), 33.7 (*C*H$_2$-4), 43.4 (*C*H-3), 43.8 (*C*H$_2$-1), 48.6 (*C*H-2), 63.5 (C_{quat}), 112.8 (*C*H-Fur), 115.8 (*C*H-azaAr), 120.0 (*C*H-azaAr), 122.9 (*C*H-Ar), 123.2 (*C*H-Fur), 125.4 (*C*H-Ar), 125.7 (*C*H-azaAr), 125.8 (*C*H-azaAr), 128.1 (*C*H-Ar), 128.6 (*C*H-Ar), 131.6 (*C*-azaAr), 139.1 (*C*-azaAr), 142.4 (*C*1-Ar), 144.0 (*C*6-Ar), 147.8 (*C*H-Fur), 150.8 (*C*-Fur), 152.7 (N=*C*(C)-N), 167.2 (CH-*C*=ON), 183.7 (*C*=OFur), 205.9 (*C*=OCH$_3$); *m/z* (NSI$^+$) 425 ([M+Na]$^+$, 100%); HRMS (NSI$^+$) $C_{26}H_{21}O_4N_2$[M+H]$^+$, found 425.1491, requires 425.1496 (–1.1 ppm).

minor: ^{1}H NMR (500 MHz, $CDCl_3$) δ_H: 2.24 (3H, s, COC*H*$_3$), 2.95 (1H, dd, *J* 17.7, 8.8, H–4a), 3.20 (1H, dd, *J* 17.7, 5.7, H–4b), 3.27–3.28 (2H, m, H–1a,b), 4.36 (1H, t, *J* 6.0, H–3), 5.30 (1H, dd, *J* 9.0, 5.6, H–2), 6.42 (1H, dd, *J* 3.7, 1.8, H–Fur), 7.20–7.22 (4H, m, H–Ar), 7.27–7.28 (1H, m, H-Fur), 7.35–7.39 (2H, m, H-azaAr), 7.66–7.68 (1H, m, H-azaAr), 8.25 (1H, dd, *J* 7.3, 2.1, H–Fur).

12a-Benzoyl-3-bromo-12-(2-oxopropyl)-7,7a,12,12a-tetrahydro-6*H*-benzo[4,5]imidazo[1,2-*a*]indeno[2,1-*c*]pyridin-6-one (±62)

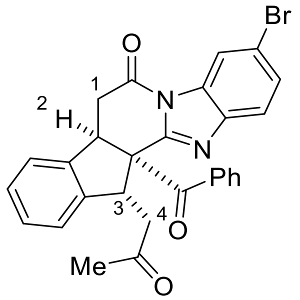

Following General Procedure 11, the corresponding TCP-ester (39.6 mg, 0.1 mmol), 2-phenacyl-(6-bromo)benzimidazole (47.0 mg, 0.15 mmol) and PS-BEMP (2.0 mmol/g loading, 75.0 mg) in CH_2Cl_2 (0.5 mL) and subsequent chromatography (80:20 Petrol : EtOAc, R_f 0.2) afforded the title compound as inseparable mixture of two rotamers (29.1 mg, 57 %) as an off-white solid. mp 88–89 °C; ν_{max} (film) 3073 (C-H), 2926 (C-H), 1721 (C=O), 1664 (C=O), 1449 (C_{Ar}-C_{Ar}); ^{1}H NMR (500 MHz, $CDCl_3$) δ_H: 1.99 (3H, s, COC*H*$_3$), 2.67–2.82 (2H, m, H–1a,b), 3.48 (1H, dt, *J* 18.3, 3.3, H–4a), 3.74 (1H, ddd, *J* 18.3, 5.8, 2.6, H–4b), 4.61 (1H, dt, *J* 5.8, 3.3, H–3), 5.40 (1H, dt, *J* 8.2, 5.4, H–2), 7.09–7.24 (4H, m, H–Ar), 7.36 (1H, td, *J* 8.6, 2.0, H-azaAr), 7.45–7.48 (2H, m, H-Ph), 7.53–7.56 (1H, m, H-Ph), 7.78 (1H, d, *J* 2.0, H-azaAr), 7.96 (1H, d, *J* 8.6, H-azaAr), 8.29 (1H, d, *J* 2.0, H-azaAr), 8.51 (2H, d, *J* 7.4, H-Ph); ^{13}C NMR (126 MHz, $CDCl_3$) δ_C: 30.9 (CO-*C*H$_3$), 33.1 (*C*H$_2$-4), 43.8 (*C*H$_2$-1), 2× 44.2 (*C*H-3), 49.0 (*C*H-2), 2× 65.6 (C_{quat}), 116.7 (*C*H-aAr), 118.3 (*C*-Br), 118.7 (*C*H-aAr), 119.1 (*C'*-Br), 122.5 (*C*H-Ar), 122.9 (*C*H-aAr), 2× 125.8 (*C*H-azaAr), 128.1 (*C*H-Ar), 128.6 (*C*H-Ar), 128.6 (2×*C*H-Ph), 130.6 (2×*C*H-Ph), 2× 133.9 (*C*H-Ph), 2× 134.9 (*C*-aAr), 138.7 (*C*-Ph), 141.1 (*C*1-Ar), 141.7 (*C*-azaAr), 143.4 (*C*6-Ar), 2× 153.3 (N=*C*(C)-N), 167.0 (CH-*C*=ON), 195.9 (*C*=OPh), 205.5 (*C*=OCH$_3$); *m/z* (NSI$^+$) 535 ([M+Na]$^+$, 100%), 1049 ([2M+Na]$^+$, 40%); HRMS (NSI$^+$) $C_{28}H_{21}O_3N_2Br_1Na$ [M+Na]$^+$, found 535.0614, requires 535.0628 (–2.6 ppm).

3-Chloro-12a-(4-methoxybenzoyl)-12-(2-oxopropyl)-7,7a,12,12a-tetrahydro-6*H*-benzo[4,5]imidazo[1,2-*a*]indeno[2,1-*c*]pyridin-6-one (±63)

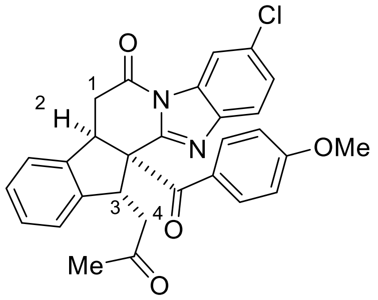

Following General Procedure 11, the corresponding TCP-ester (39.6 mg, 0.1 mmol), 2-(4-methoxy)phenacyl(6-chloro)benzimidazole (45.1 mg, 0.15 mmol) and PS-BEMP (2.0 mmol/g loading, 75.0 mg) in CH_2Cl_2 (0.5 mL) and subsequent chromatography (80:20 – 70:30 Petrol : EtOAc, R_f 0.2) afforded the title compound as inseparable mixture of two rotamers (30.1 mg, 62 %) as an off-white foam. mp 102–103 °C; ν_{max} (film) 2926 (C-H), 1721 (C=O), 1655 (C=O), 1454 (C_{Ar}-C_{Ar}); ^{1}H NMR (500 MHz, $CDCl_3$) δ_H: 1.98 (3H, s, COC*H*₃), 2.68–2.75 (2H, m, H–1a,b), 3.48 (1H, dt, *J* 18.4, 3.0, H–4a), 3.77 (1H, ddd, *J* 18.3, 5.8, 2.5, H–4b), 2× 3.87 (Ar-OC*H*₃), 4.60 (1H, dt, *J* 5.8, 3.0, H–3), 5.38 (1H, q, *J* 6.4, H–2), 6.94 (2H, d, *J* 9.0, H-Ar_{OMe}); 7.10–7.24 (4H, m, H–Ar), 7.54 (1H, d, *J* 8.7, H-azaAr), 7.62 (1H, d, *J* 2.1, H-azaAr), 8.01 (1H, d, *J* 8.7, H-azaAr), 8.13 (1H, d, *J* 2.1, H-azaAr), 8.59 (2H, d, *J* 7.8, H-Ar_{OMe}); ^{13}C NMR (126 MHz, $CDCl_3$) δ_C: 31.1 (CO-*C*H₃), 2× 33.2 (*C*H₂-4), 43.9 (*C*H₂-1), 2× 44.2 (*C*H-3), 2× 49.1 (*C*H-2), 55.7 (Ar-O*C*H₃), 2× 65.6 (C_{quat}), 113.8 (2×*C*H-Ar_{OMe}), 116.0 (*C*H-aAr), 116.5 (*C*H-aAr), 119.9 (*C*H-aAr), 120.6 (*C*H-aAr), 122.6 (*C*H-Ar), 2× 125.8 (*C*H-azaAr), 126.0 (*C*H-Ar), 127.7 (*C'*-Cl), 127.8 (*C'*-Cl), 128.2 (*C*H-Ar), 128.6 (*C*H-Ar), 130.1 (*C*-aAr), 130.9 (*C*-Ar_{OMe}), 131.5 (*C*-aAr), 132.0 (*C*-aAr), 133.5 (2×*C*H-Ar_{OMe}), 139.0 (*C*1-Ar), 140.8 (*C*-azaAr), 143.8 (*C*-azaAr), 143.9 (*C*6-Ar), 153.9 (N=*C*(C)-N), 154.6 (N=*C*(C)-N), 164.1 (*C*-OMe), 167.3 (CH-*C*=ON), 167.4 (CH-*C*=ON), 193.9 (*C*=OAr_{OMe}), 205.7 (*C*=OCH₃); *m/z* (NSI^+) 499 ($[M+H]^+$, 100%); HRMS (NSI^+) $C_{29}H_{24}O_3N_2Cl$ $[M+H]^+$, found 499.1408, requires 499.1419 (–2.2 ppm).

12a-Benzoyl-2,3-dimethyl-12-(2-oxopropyl)-7,7a,12,12a-tetrahydro-6*H*-benzo[4,5]imidazo[1,2-*a*]indeno[2,1-*c*]pyridin-6-one (±64)

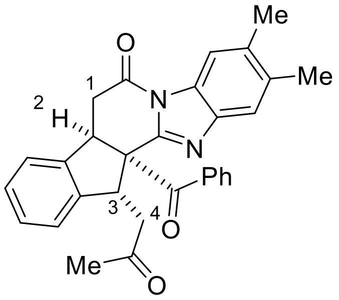

Following General Procedure 11, the corresponding TCP-ester (39.6 mg, 0.1 mmol), 2-phenacyl(5,6-dimethyl)benzimidazole (39.5 mg, 0.15 mmol) and PS-BEMP (2.0 mmol/g loading, 75.0 mg) in CH_2Cl_2 (0.5 mL) and subsequent chromatography (80:20 Petrol : EtOAc, R_f 0.2) afforded the title compound (38.4 mg, 83 %) as a brown solid. mp 52–53 °C; ν_{max} (film) 2924 (C-H), 1719 (C=O), 1665 (C=O), 1464 (C_{Ar}-C_{Ar}); ^{1}H NMR (500 MHz, $CDCl_3$) δ_H: 1.99 (3H, s, COC*H*$_3$), 2×2.27 (3H, s, aAr-Me), 2.74–2.78 (2H, m, H–1a,b), 3.45 (1H, dd, *J* 18.3, 3.0, H–4a), 3.71 (1H, dd, *J* 18.3, 5.8, H–4b), 4.58 (1H, dd, *J* 5.8, 3.0, H–3), 5.38 (1H, dd, *J* 9.0, 5.1, H–2), 7.05–7.25 (4H, m, H–Ar), 7.40 (1H, s, H-azaAr), 7.45–7.48 (2H, m, H-Ph), 7.51–7.54 (1H, m, H-Ph), 7.90 (1H, s, H-azaAr), 8.51 (2H, d, *J* 7.5, H-Ph); ^{13}C NMR (126 MHz, $CDCl_3$) δ_C: 20.3 (aAr-*C*H$_3$), 20.4 (aAr-*C*H$_3$), 31.0 (CO-*C*H$_3$), 33.3 (*C*H$_2$-4), 43.9 (*C*H$_2$-1), 44.3 (*C*H-3), 48.9 (*C*H-2), 65.5 (C_{quat}), 115.9 (*C*H-aAr), 120.1 (*C*H-aAr), 122.6 (*C*H-Ar), 125.9 (*C*H-Ar), 128.1 (*C*H-Ar), 128.5 (*C*H-Ar), 128.5 (2×*C*H-Ph), 129.9 (*C*-aAr), 130.7 (2×*C*H-Ph), 133.7 (*C*H-Ph), 134.2 (aAr-*C*CH$_3$), 135.0 (*C*-Ph), 135.2 (aAr-*C*CH$_3$), 139.2 (*C*-azaAr), 140.7 (*C*1-Ar), 143.9 (*C*6-Ar), 151.9 (N=*C*(C)-N), 167.3 (CH-*C*=ON), 196.4 (*C*=OPh), 205.8 (*C*=OCH$_3$); *m/z* (NSI$^+$) 463 ([M+H]$^+$, 100%); HRMS (NSI$^+$) $C_{30}H_{27}O_3N_2$ [M+H]$^+$, found 463.2007, requires 463.2016 (–2.0 ppm).

12a-Benzoyl-9-methyl-12-(2-oxopropyl)-7,7a,12,12a-tetrahydro-6*H*-benzo[4,5]imidazo[1,2-*a*]indeno[2,1-*c*]pyridin-6-one (±65)

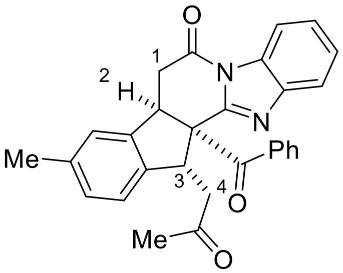

Following General Procedure 11, the corresponding TCP-ester (41.0 mg, 0.1 mmol), 2-phenacylbenzimidazole (35.5 mg, 0.15 mmol) and PS-BEMP (2.0 mmol/g loading, 75.0 mg) in CH_2Cl_2 (0.5 mL) and subsequent chromatography (80:20 Petrol : EtOAc, R_f 0.2) afforded the title compound (37.3 mg, 83 %) as a yellow solid. mp 79–81 °C; ν_{max} (film) 2922 (C-H), 1717 (C=O), 1663 (C=O), 1451 (C_{Ar}-C_{Ar}); ^{1}H NMR (500 MHz, $CDCl_3$) δ_H: 1.99 (3H, s, COC*H*$_3$), 2.25 (3H, s, C*H*$_3$–Ar), 2.69–2.76 (2H, m, H–1a,b), 3.47 (1H, dd, *J* 18.3, 2.9, H–4a), 3.75 (1H, dd, *J* 18.3, 5.8, H–4b), 4.58 (1H, dd, *J* 6.2, 2.8, H–3), 5.39 (1H, dd, *J* 8.4, 5.8, H–2), 6.92 (1H, d, *J* 7.7, H–Ar), 7.01 (1H, s, H–Ar), 7.12 (1H, d, *J* 7.7, H–Ar), 7.28–7.30 (2H, dd, *J* 5.9, 3.0, H-azaAr), 7.47 (2H, t, *J* 7.7, H-Ph), 7.54 (1H, t, *J* 7.2, H-Ph), 7.65 (1H, dd, *J* 6.1, 3.2, H-azaAr), 8.13 (1H, dd, *J* 6.2, 3.1, H-azaAr), 8.56 (2H, d, *J* 7.7, H-Ph); ^{13}C NMR (126 MHz, $CDCl_3$) δ_C: 21.5 (*C*H$_3$–Ar), 31.1 (CO-*C*H$_3$), 33.3 (*C*H$_2$-4), 44.1 (*C*H$_2$-1), 44.2 (*C*H-3), 48.8 (*C*H-2), 65.8 (C_{quat}), 115.7 (*C*H-aAr), 120.0 (*C*H-aAr), 123.3 (*C*H-Ar), 125.3 (*C*H-Ar), 125.6 (*C*H-azaAr), 125.7 (*C*H-azaAr), 128.6 (2×*C*H-Ph), 129.3 (*C*H-Ar), 130.8 (2×*C*H-Ph), 131.6 (*C*-aAr), 133.8 (*C*H-Ph), 135.2 (*C*-Ph), 138.0 (*C*-azaAr), 139.1 (*C*-CH$_3$), 140.9 (*C*1-Ar), 142.3 (*C*6-Ar), 152.9 (N=*C*(C)-N), 167.4 (CH-*C*=ON), 196.3 (*C*=OPh), 205.7 (*C*=OCH$_3$); *m/z* (NSI$^+$) 471 ([M+Na]$^+$, 100%), 919 ([2M+Na]$^+$, 30%); HRMS (NSI$^+$) $C_{29}H_{24}O_3N_2Na$ [M+Na]$^+$, found 471.1692, requires 471.1679 (+2.7 ppm).

12a-Benzoyl-10-fluoro-12-(2-oxopropyl)-7,7a,12,12a-tetrahydro-6*H*-benzo[4,5]imidazo[1,2-*a*]indeno[2,1-*c*]pyridin-6-one (±66)

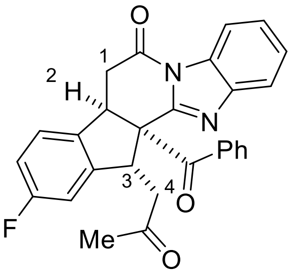

Following General Procedure 11, the corresponding TCP-ester (41.4 mg, 0.1 mmol), 2-phenacylbenzimidazole (35.5 mg, 0.15 mmol) and PS-BEMP (2.0 mmol/g loading, 75.0 mg) in CH_2Cl_2 (0.5 mL) and subsequent chromatography (80:20 Petrol : EtOAc, R_f 0.2) afforded the title compound (37.1 mg, 82 %) as a colorless oil; ν_{max} (film) 2926 (C-H), 1721 (C=O), 1667 (C=O), 1449 (C_{Ar}-C_{Ar}); ^{1}H NMR (500 MHz, $CDCl_3$) δ_H: 2.01 (3H, s, COC*H*$_3$), 2.72–2.77 (2H, m, H–1a,b), 3.43 (1H, dd, *J* 18.3, 3.2, H–4a), 3.75 (1H, dd, *J* 18.3, 5.8, H–4b), 4.55 (1H, dd, *J* 5.8, 3.2, H–3), 5.38 (1H, t, *J* 7.1, H–2), 6.85 (1H, td, *J* 8.7, 2.6, H–Ar), 6.99 (1H, dd, *J* 8.7, 2.6, H–Ar), 7.15 (1H, dd, *J* 8.5, 4.8, H–Ar), 7.29 (2H, dd, *J* 6.1, 3.2 Hz H-azaAr), 7.46 (2H, t, *J* 7.7, H-Ph), 7.54 (1H, t, *J* 7.4, H-Ph), 7.66 (1H, dd, *J* 6.4, 2.9, H-azaAr), 8.13 (1H, dd, *J* 6.4, 2.9, H-azaAr), 8.50 (2H, d, *J* 7.6, H-Ph); ^{19}F NMR (282 MHz, $CDCl_3$) δ_F: –113.1; ^{13}C NMR (126 MHz, $CDCl_3$) δ_C: 30.8 (CO-*C*H$_3$), 33.4 (*C*H$_2$-4), 43.6 (*C*H$_2$-1), 43.7 (*C*H-3), 48.6 (*C*H-2), 65.6 (C_{quat}), 113.5 (*C*H-Ar), 113.6 (d, $^2J_{CF}$ = 22.6, C_{Ar}-F), 114.9 (*C*H-Ar), 115.0 (d, $^2J_{CF}$ = 22.7, C_{Ar}-F), 115.6 (*C*H-azaAr), 119.9 (*C*H-azaAr), 123.7 (*C*H-Ar), 123.8 (d, $^3J_{CF}$ = 9.2, C_{Ar}-F), 125.3 (*C*H-azaAr), 125.8 (*C*H-azaAr), 128.6 (2×*C*H-Ph), 130.6 (2×*C*H-Ph), 131.4 (*C*-azaAr), 133.9 (*C*H-Ph), 134.5 (d, $^4J_{CF}$ = 2.3, C_{Ar}-F), 134.9 (*C*-Ph), 142.1 (*C*-azaAr), 146.0 (*C*1-Ar), 146.1 (*C*6-Ar), 152.4 (N=*C*(C)-N), 161.8 (*C*-F), 162.8 (d, $^1J_{CF}$ = 246.5, C_{Ar}-F), 167.0 (CH-*C*=ON), 195.8 (*C*=OPh), 205.2 (*C*=OCH$_3$); *m/z* (NSI$^+$) 255 ([M+H]$^+$, 100%); HRMS (NSI$^+$) $C_{15}H_{12}F_1N_2O_1$ [M+H]$^+$, found 255.0930, requires 255.0928 (+0.7 ppm).

12a-Benzoyl-12-(3,3-dimethyl-2-oxobutyl)-7,7a,12,12a-tetrahydro-6*H*-benzo[4,5]imidazo[1,2-*a*]indeno[2,1-*c*]pyridin-6-one (±67)

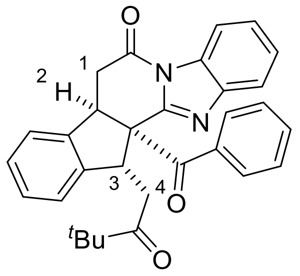

Following General Procedure 11, the corresponding TCP-ester (43.8 mg, 0.1 mmol), 2-phenacylbenzimidazole (39.5 mg, 0.15 mmol) and PS-BEMP (2.0 mmol/g loading, 75.0 mg) in THF (0.5 mL) and subsequent chromatography (90:10 – 80:20 Petrol : EtOAc, R_f 0.2) afforded the title compound (44.8 mg, 94%) as a colorless crystalline solid. mp 88–89 °C; ν_{max} (film) 2926 (C-H), 1728 (C=O), 1661 (C=O), 1530 (C_{Ar}-C_{Ar}), 1452 (C_{Ar}-C_{Ar}); ^{1}H NMR (500 MHz, $CDCl_3$) δ_H: 0.86 (9H, s, $(CH_3)_3$), 2.67 (1H, dd, *J* 17.3, 3.0, H–1a), 2.86 (1H, dd, *J* 17.1, 11.0, H–1b), 3.49 (1H, dd, *J* 18.3, 2.9, H–4a), 3.80 (1H, dd, *J* 18.3, 5.9, H–4b), 4.63 (1H, dd, *J* 6.1, 2.7, H–3), 5.45 (1H, dd, *J* 11.0, 3.1, H–2), 7.08–7.21 (4H, m, H-Ar), 7.26–7.27 (2H, m, H-azaAr), 7.45–7.48 (2H, m, H-Ph), 7.52–7.55 (1H, m, H-Ph), 7.64–7.66 (1H, m, H-azaAr), 8.11–8.13 (1H, m, H-azaAr), 8.56 (2H, d, *J* 7.6, H-Ph); ^{13}C NMR (126 MHz, $CDCl_3$) δ_C: 26.0 (CO-C(CH$_3$)$_3$), 33.4 (CH$_2$-4), 37.3 (CH$_2$-1), 44.4 (*C*H-3), 49.2 (*C*H-2), 65.8 (C_{quat}), 115.7 (*C*H-azaAr), 120.0 (*C*H-azaAr), 122.6 (*C*H-azaAr), 125.3 (*C*H-azaAr), 125.7 (*C*H-Ar), 126.1 (*C*H-Ar), 128.1 (*C*H-Ar), 128.4 (*C*H-Ar), 128.6 (2×*C*H-Ph), 130.8 (2×*C*H-Ph), 131.6 (*C*-azaAr), 133.8 (*C*H-Ph), 135.4 (*C*-Ph), 139.0 (*C*-azaAr), 142.3 (*C*1-Ar), 144.3 (*C*6-Ar), 152.9 (N=*C*(C)-N), 167.4 (CH-*C*=ON), 196.5 (*C*=OPh), 212.7 (*C*=O^{t}Bu); *m/z* (NSI$^+$) 477 ([M+H]$^+$, 100%); HRMS (NSI$^+$) $C_{31}H_{28}N_2O_3$ [M+H]$^+$, found 477.2175, requires 477.2178 (–0.3 ppm).

12a-Benzoyl-12-(2-(4-methoxyphenyl)-2-oxoethyl)-7,7a,12,12a-tetrahydro-6*H*-benzo[4,5]imidazo[1,2-*a*]indeno[2,1-*c*]pyridin-6-one (±68)

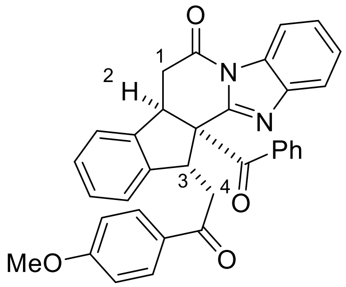

Following General Procedure 11, the corresponding TCP-ester (48.8 mg, 0.1 mmol), 2-phenacylbenzimidazole (35.5 mg, 0.15 mmol) and PS-BEMP (2.0 mmol/g loading, 75.0 mg) in CH_2Cl_2 (0.5 mL) and subsequent chromatography (80:20 – 70:30 Petrol : EtOAc, R_f 0.15) afforded the title compound (42.1 mg, 80 %) as a yellow solid. mp 61–62 °C; ν_{max} (film) 2930 (C-H), 1728 (C=O), 1667 (C=O), 1452 (C_{Ar}-C_{Ar}); ^{1}H NMR (500 MHz, $CDCl_3$) δ_H: 3.17 (1H, dd, *J* 16.5, 3.1, H–1a), 3.32 (1H, dd, *J* 16.5, 11.2, H–1b), 3.51 (1H, dd, *J* 18.3, 3.1, H–4a), 3.79 (1H, dd, *J* 18.4, 5.9, H–4b), 3.80 (3H, s, OCH_3), 4.73 (1H, dd, *J* 6.1, 3.3, H–3), 5.60 (1H, dd, *J* 11.2, 3.0, H–2), 6.84 (2H, d, *J* 8.8, H–Ar_{OMe}), 7.05–7.15 (2H, m, H-azaAr), 7.20–7.29 (4H, m, H–Ar), 7.44–7.47 (2H, m, H-Ph), 7.50–7.53 (1H, m, H-Ph), 7.65 (1H, dd, *J* 5.6, 3.4, H-azaAr), 7.76 (2H, d, *J* 8.8, H–Ar_{OMe}), 8.14 (1H, dd, *J* 6.1, 3.0, H-azaAr), 8.57 (2H, d, *J* 7.6, H-Ph); ^{13}C NMR (126 MHz, $CDCl_3$) δ_C: 33.3 (*C*H_2-4), 38.5 (*C*H_2-1), 44.3 (*C*H-3), 49.5 (*C*H-2), 55.5 (O*C*H_3), 65.8 (C_{quat}), 113.7 (*C*H-Ar_{OMe}), 115.6 (*C*H-aAr), 119.9 (*C*H-aAr), 122.4 (*C*H-Ar), 125.2 (*C*H-Ar), 125.6 (*C*H-azaAr), 126.2 (*C*H-azaAr), 127.9 (*C*H-Ar), 128.3 (*C*H-Ar), 128.5 (2×*C*H-Ph), 129.9 (*C*-Ar_{OMe}), 130.4 (*C*H-Ar_{OMe}), 130.7 (2×*C*H-Ph), 131.5 (*C*-aAr), 133.7 (*C*H-Ph), 135.1 (*C*-Ph), 138.9 (*C*-azaAr), 142.2 (*C*1-Ar), 143.8 (*C*6-Ar), 152.8 (N=*C*(C)-N), 163.6 (*C*-OMe), 167.3 (CH-*C*=ON), 195.3 (*C*=OAr_{OMe}), 196.3 (*C*=OPh); *m/z* (NSI$^+$) 549 ([M+Na]$^+$, 100%); HRMS (NSI$^+$) $C_{34}H_{26}O_4N_2Na$ [M+Na]$^+$, found 549.1770, requires 549.1785 (–2.7 ppm).

Methyl 2-(12a-benzoyl-6-oxo-7,7a,12,12a-tetrahydro-6*H*-benzo[4,5]imidazo[1,2-*a*]indeno[2,1-*c*]pyridin-12-yl)acetate (±69)

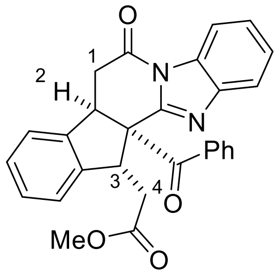

Following General Procedure 11, the corresponding TCP-ester (41.1 mg, 0.1 mmol), 2-phenacylbenzimidazole (35.5 mg, 0.15 mmol) and PS-BEMP (2.0 mmol/g loading, 75.0 mg) in CH_2Cl_2 (0.5 mL) and subsequent chromatography (80:20 Petrol : EtOAc, R_f 0.2) afforded the title compound (35.6 mg, 79 %) as a yellow solid. mp 125–127 °C; ν_{max} (film) 2922 (C-H), 1732 (C=O), 1670 (C=O), 1445 (C_{Ar}-C_{Ar}); ^{1}H NMR (500 MHz, $CDCl_3$) δ_H: 2.45–2.54 (1H, m, H–1a), 2.73 (1H, dd, *J* 15.3, 3.8, H–1b), 3.52–3.55 (1H, m, H–4a), 3.65 (3H, s, CO_2CH_3), 3.83 (1H, dd, *J* 18.4, 5.9, H–4b), 4.67–4.68 (1H, m, H–3), 5.39 (1H, dd, *J* 11.9, 3.8, H–2), 7.16–7.31 (6H, m, 4×H–Ar + 2× H-azaAr), 7.52–7.61 (3H, m, H-Ph), 7.67–7.69 (1H, m, H-azaAr), 8.15–8.17 (1H, m, H-azaAr), 8.67 (2H, d, *J* 7.9, H-Ph); ^{13}C NMR (126 MHz, $CDCl_3$) δ_C: 33.2 (*C*H_2-4), 35.4 (*C*H_2-1), 44.0 (*C*H-3), 50.3 (*C*H-2), 51.9 (CO_2CH_3), 65.9 (C_{quat}), 115.8 (*C*H-aAr), 119.9 (*C*H-aAr), 122.8 (*C*H-Ar), 125.3 (*C*H-Ar), 125.6 (*C*H-azaAr), 125.8 (*C*H-azaAr), 128.5 (*C*H-Ar), 128.6 (*C*H-Ar), 128.6 (2×*C*H-Ph), 130.9 (2×*C*H-Ph), 131.6 (*C*-aAr), 133.9 (*C*H-Ph), 135.1 (*C*-Ph), 139.2 (*C*-azaAr), 142.2 (*C*1-Ar), 143.0 (*C*6-Ar), 152.7 (N=*C*(C)-N), 167.4 (CH-*C*=ON), 171.5 (CO_2CH_3), 195.7 (*C*=OPh); *m/z* (NSI^+) 451 ($[M+H]^+$, 100%); HRMS (NSI^+) $C_{28}H_{22}N_2O_4$ $[M+H]^+$, found 451.1660, requires 451.1658 (+0.4 ppm).

6.2 Data for enantioenriched indanes 54, 55, 65, 67, 68

(7a*R*,12*R*,12a*R*)-12a-Benzoyl-12-(2-oxopropyl)-7,7a,12,12a-tetrahydro-6*H*-benzo[4,5]imidazo[1,2-a]indeno[2,1-*c*]pyridin-6-one (54)

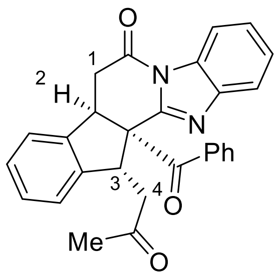

Following General Procedure 12, the corresponding TCP-ester (39.6 mg, 0.1 mmol), 2-phenacylbenzimidazole (47.3 mg, 0.20 mmol) and $^{i}Pr_2NEt$ (34 μL, 0.20 mmol) in CH_2Cl_2 (0.5 mL) and subsequent chromatography (80:20 Petrol : EtOAc, R_f 0.2) afforded the title compound (26.1 mg, 60%) as a pink solid. The analytical data is in agreement with (±)-**54**. $[\alpha]_D^{20}$ –56.0 (*c* 0.3, $CHCl_3$); Chiral HPLC analysis, Chiralpak IA (97:3 hexane : IPA, flow rate 1.5 mLmin^{-1}, 211 nm, 40 °C) t_R major: 14.8 min, t_R minor: 16.6 min, 88:12 er.

(7a*R*,12*R*,12a*R*)-12a-(4-Methoxybenzoyl)-12-(2-oxopropyl)-7,7a,12,12a-tetrahydro-6*H*-benzo[4,5]imidazo[1,2-*a*]indeno[2,1-*c*]pyridin-6-one (55)

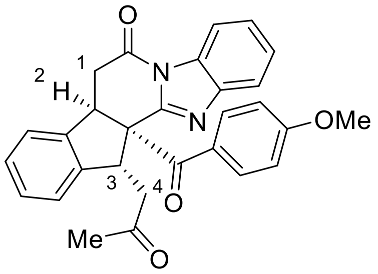

Following General Procedure 12, the corresponding TCP-ester (39.6 mg, 0.1 mmol), 2-(4-methoxy)phenacylbenzimidazole (53.2 mg, 0.20 mmol) and $^{i}Pr_2NEt$ (34 μL, 0.20 mmol) in CH_2Cl_2 (0.5 mL) and subsequent chromatography (80:20 – 70:30 Petrol : EtOAc, R_f 0.2) afforded the title compound (22.6 mg, 49%) as a pale pink solid. The analytical data is in agreement with (±)-**55**. $[\alpha]_D^{20}$ –16.0 (*c* 0.7, $CHCl_3$); Chiral HPLC analysis, Chiralpak IA (98:2 hexane : IPA, flow rate 1.5 mLmin^{-1}, 211 nm, 40 °C) t_R minor: 29.8 min, t_R major: 34.0 min, 85:15 er (after single recryst. 98.5:1.5 er).

(7a*R*,12*R*,12a*R*)-12a-Benzoyl-9-methyl-12-(2-oxopropyl)-7,7a,12,12a-tetrahydro-6*H*-benzo[4,5]imidazo[1,2-*a*]indeno[2,1-*c*]pyridin-6-one (65)

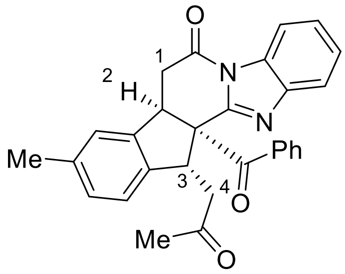

Following General Procedure 12, the corresponding TCP-ester (41.0 mg, 0.1 mmol), 2-phenacylbenzimidazole (47.3 mg, 0.20 mmol) and $^{i}Pr_2NEt$ (34 μL, 0.20 mmol) in CH_2Cl_2 (0.5 mL) and subsequent chromatography (80:20 Petrol : EtOAc, R_f 0.2) afforded the title compound (32.0 mg, 71%) as a yellow solid. The analytical data is in agreement with (±)-**65**. $[\alpha]_D^{20}$ −42.1 (*c* 0.9, $CHCl_3$); Chiral HPLC analysis, Chiralpak IA (97.5:2.5 hexane : IPA, flow rate 0.5 $mLmin^{-1}$, 211 nm, 40 °C) t_R minor: 19.5 min, t_R major: 20.8 min, 81:19 er.

(7a*R*,12*R*,12a*R*)-12a-Benzoyl-12-(3,3-dimethyl-2-oxobutyl)-7,7a,12,12a-tetrahydro-6*H*-benzo[4,5]imidazo[1,2-*a*]indeno[2,1-*c*]pyridin-6-one (67)

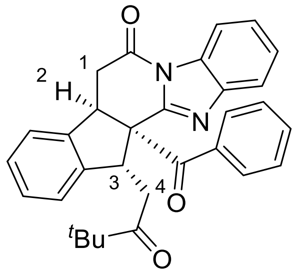

Following General Procedure 12, the corresponding TCP-ester (43.8 mg, 0.1 mmol), 2-phenacylbenzimidazole (47.3 mg, 0.20 mmol) and $^{i}Pr_2NEt$ (34 μL, 0.20 mmol) in CH_2Cl_2 (0.5 mL) and subsequent chromatography (90:10 Petrol : EtOAc, R_f 0.2) afforded the title compound (29.6 mg, 62%) as a colorless solid. The analytical data is in agreement with (±)-**67**. $[\alpha]_D^{20}$ −74.3 (*c* 0.6, $CHCl_3$); Chiral HPLC analysis, Chiralpak IA (99:1 hexane : IPA, flow rate 1.0 $mLmin^{-1}$, 211 nm, 40 °C) t_R major: 16.2 min, t_R minor: 19.8 min, 78:22 er.

(7a*R*,12*R*,12a*R*)-12a-Benzoyl-12-(2-(4-methoxyphenyl)-2-oxoethyl)-7,7a,12,12a-tetrahydro-6*H*-benzo[4,5]imidazo[1,2-*a*]indeno[2,1-*c*]pyridin-6-one (68)

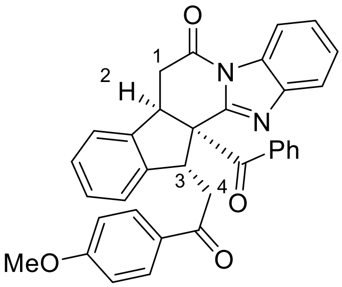

Following General Procedure 12, the corresponding TCP-ester (48.8 mg, 0.1 mmol), 2-phenacylbenzimidazole (47.3 mg, 0.20 mmol) and $^{i}Pr_2NEt$ (34 μL, 0.20 mmol) in CH_2Cl_2 (0.5 mL) and subsequent chromatography (80:20 – 70:30 Petrol : EtOAc, R_f 0.15) afforded the title compound (30.0 mg, 57%) as a colorless solid. The analytical data is in agreement with (±)-**68**. $[\alpha]_D^{20}$ −42.1 (*c* 0.9, $CHCl_3$); Chiral HPLC analysis, Chiralpak IB (97:3 hexane : IPA, flow rate 1.5 mLmin^{-1}, 211 nm, 40 °C) t_R minor: 16.1 min, t_R major: 29.7 min, 85:15 er.

7 Determination of Stereochemistry

7.1 NOE analysis of indane 7b

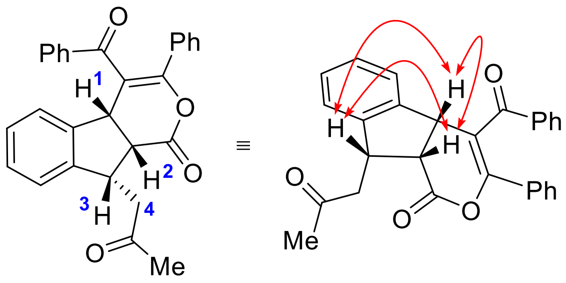

7b **Key NOE interactions**

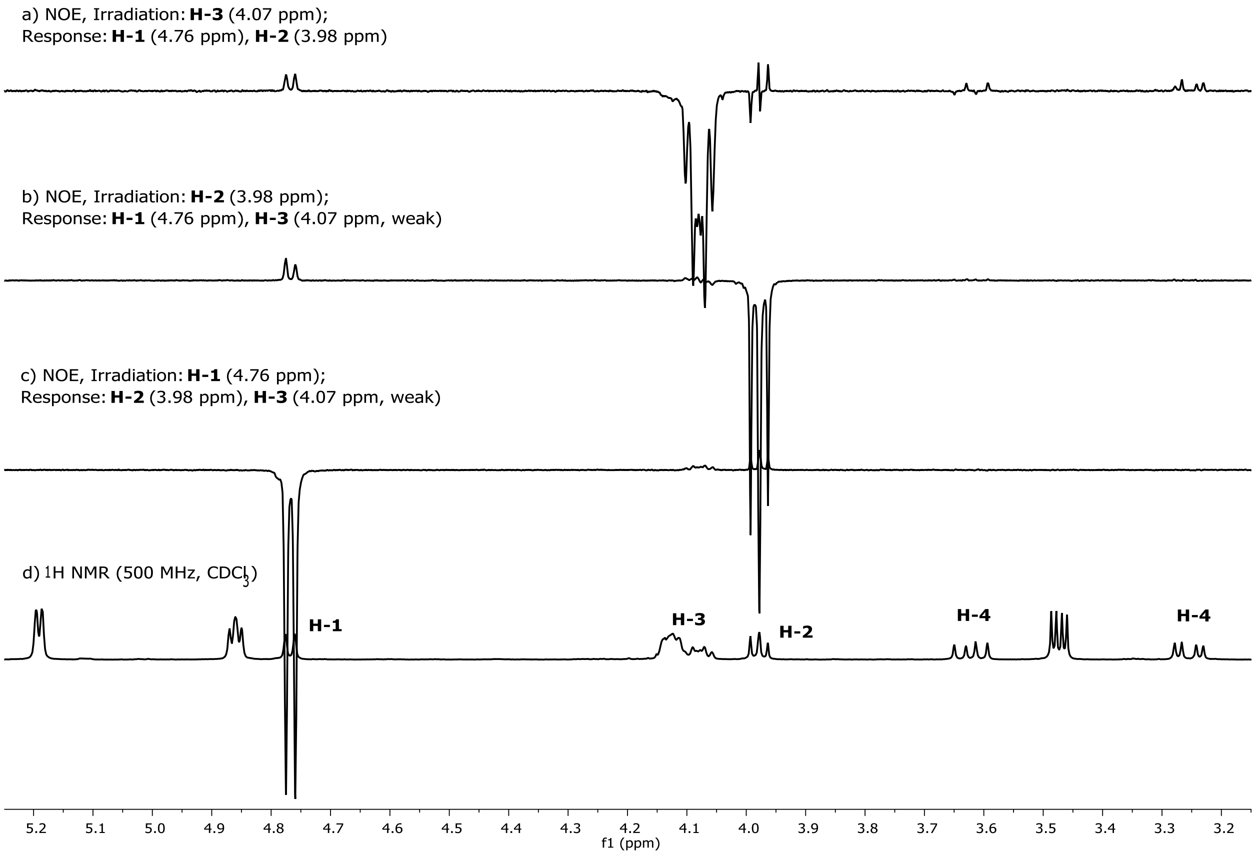

Figure S1: Summary of NOE analysis to confirm the relative configuration of indane **7b**

7.2 X-Ray crystal structure of indane 12a

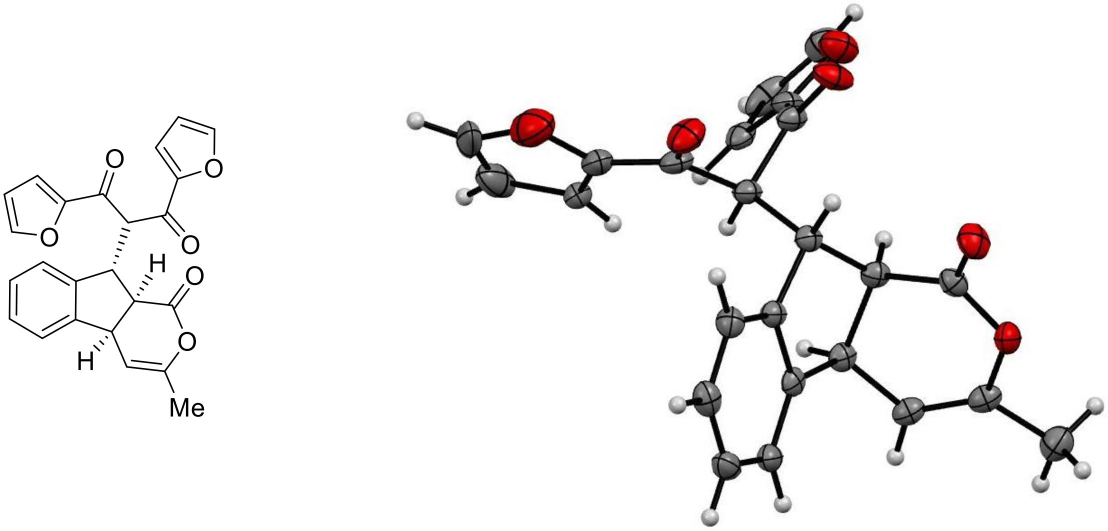

Figure S2: X-Ray crystal structure confirming the relative and absolute stereochemistry of indane **12a**

7.3 X-Ray crystal structure of indane 41a

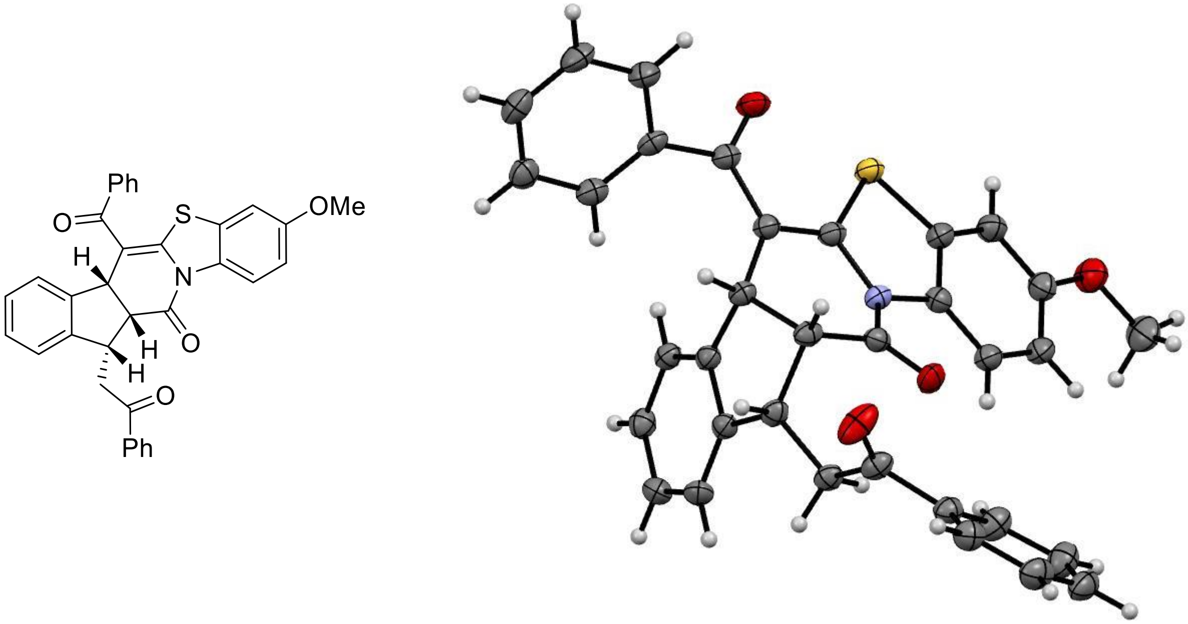

Figure S3: X-Ray crystal structure confirming the relative and absolute stereochemistry of indane **41a**

7.4 X-Ray crystal structure of indane (±)-54

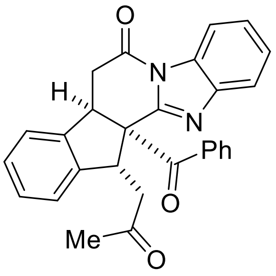

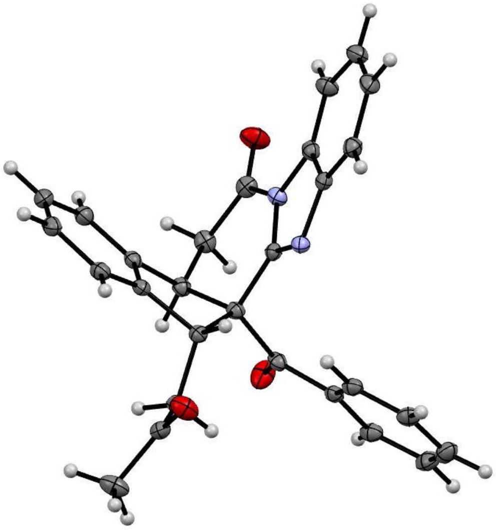

Figure S4: X-Ray crystal structure confirming the relative stereochemistry of indane **(±)-54**

7.5 X-Ray crystal structure of indane 55

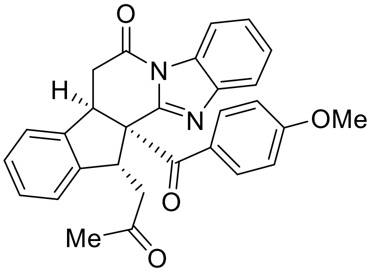

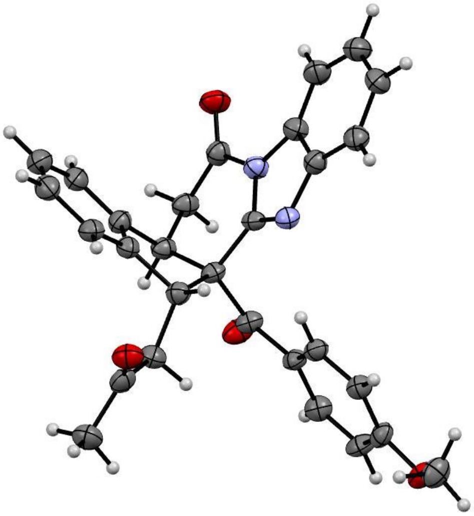

Figure S5: X-Ray crystal structure confirming the relative and absolute stereochemistry of indane **55**

8 References

[1] C. S. Bryan and M. Lautens, *Org. Lett.* 2010, **12**, 2754-2757.

[2] D. B. Denney and S. T. Ross, *J. Org. Chem.* 1962, **27**, 998-1000.

[3] D. Belmessieri, D. B. Cordes, A. M. Z. Slawin and A. D. Smith *Org. Lett.*, 2013, **15**, 3472-3475.

[4] D. B. Denney, L. C. Smith, J. Song, C. J. Rossi and C. D. Hall, *J. Org. Chem.* 1963, **28**, 778-780.

[5] G. S. Hammond and F. S. Schultz, *J. Am. Chem. Soc*. 1952, **74**, 329-332.

[6] K. Nomura, K. Asano, T. Kurahashi and S. Matsubara, *Heterocycles* 2008, **76**, 1381-1399.

[7] M. Lipp, F. Dallacker, S. Munnes, *Liebigs Ann. Chem. Bd*. 1958, **618**, 110-117.

[8] T. Yamada, T. Nagata, K. D. Sugi, K. Yorozu, T. Ikeno, Y. Ohtsuka, D. Miyazaki and T. Mukaiyama *Chem. Eur. J.* 2003, **9**, 4485-4509.

[9] Y. Kubota, S. Tanaka, K. Funabiki and M. Matsui, *Org. Lett.* 2012, **14**, 4682-4685.

[10] I. B. Dzvinchuk, A. M. Nesterenko, A. B. Ryabitskii, M. O. Lozinskii and V. V. Polovinko; *Chem. Heterocycl. Compd.* 2011, **47**, 953-963.

Appendix I: ^{1}H and ^{13}C{^{1}H} NMR Spectra

***tert*-Butyl (*E*)-3-(4-chloro-2-formylphenyl)acrylate (S2)**

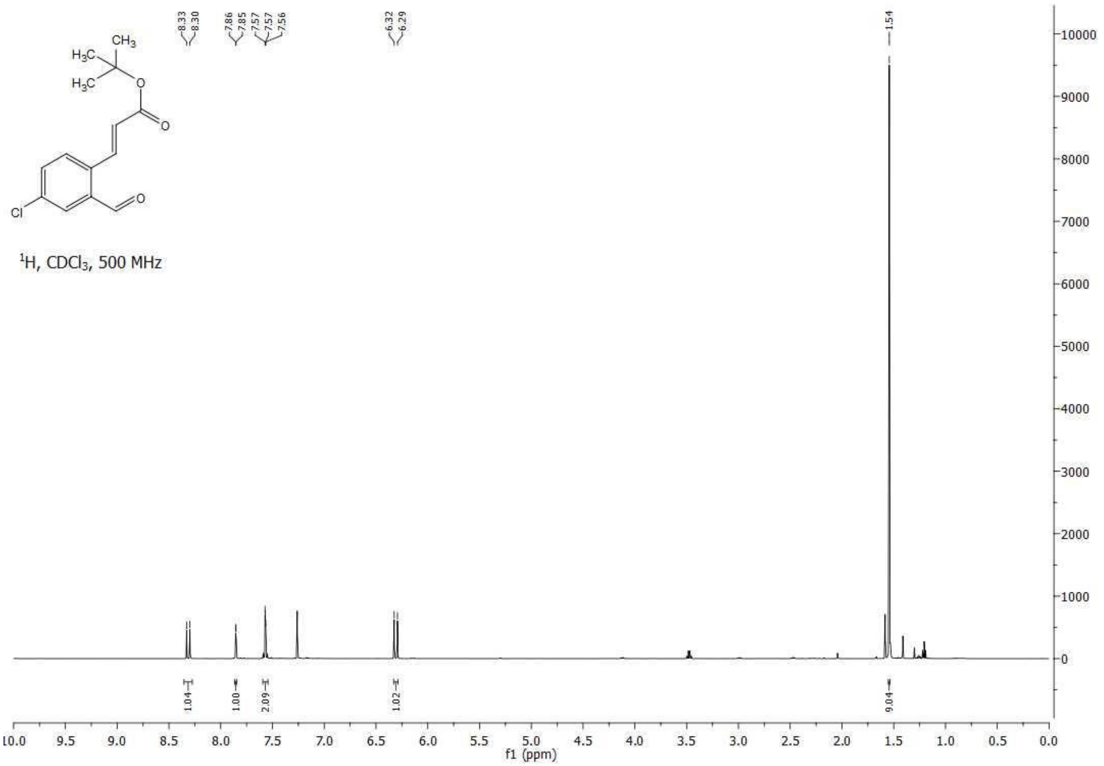

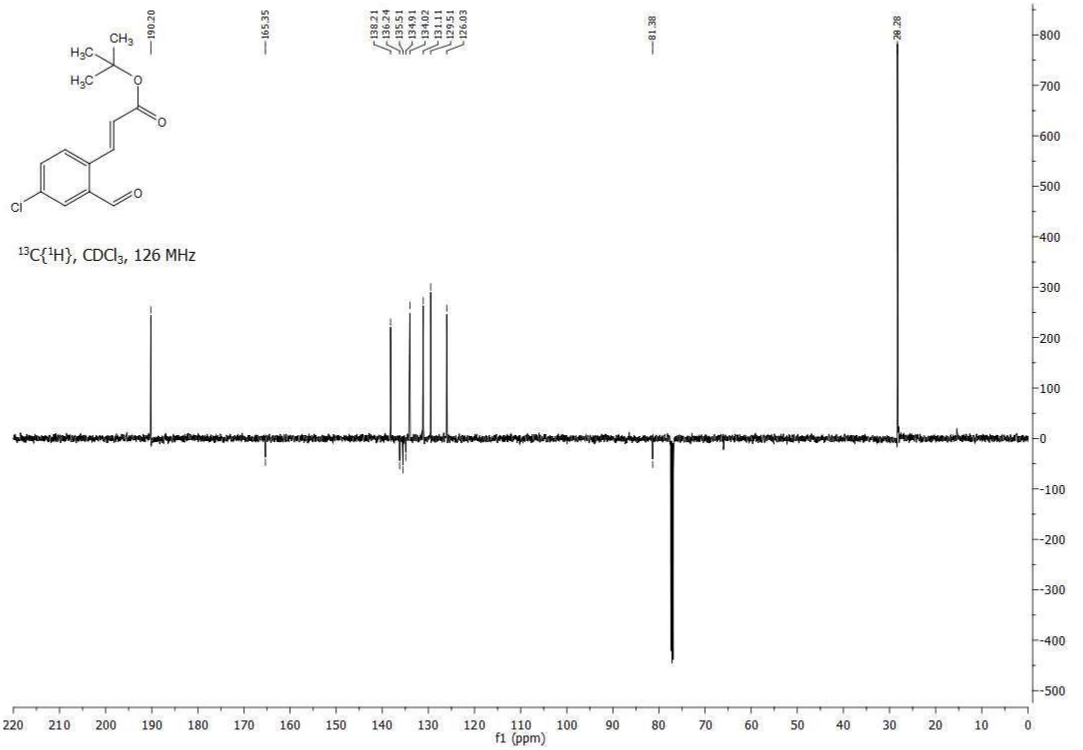

tert-butyl (_E_)-3-(2-formyl-5-methylphenyl)acrylate (S3)

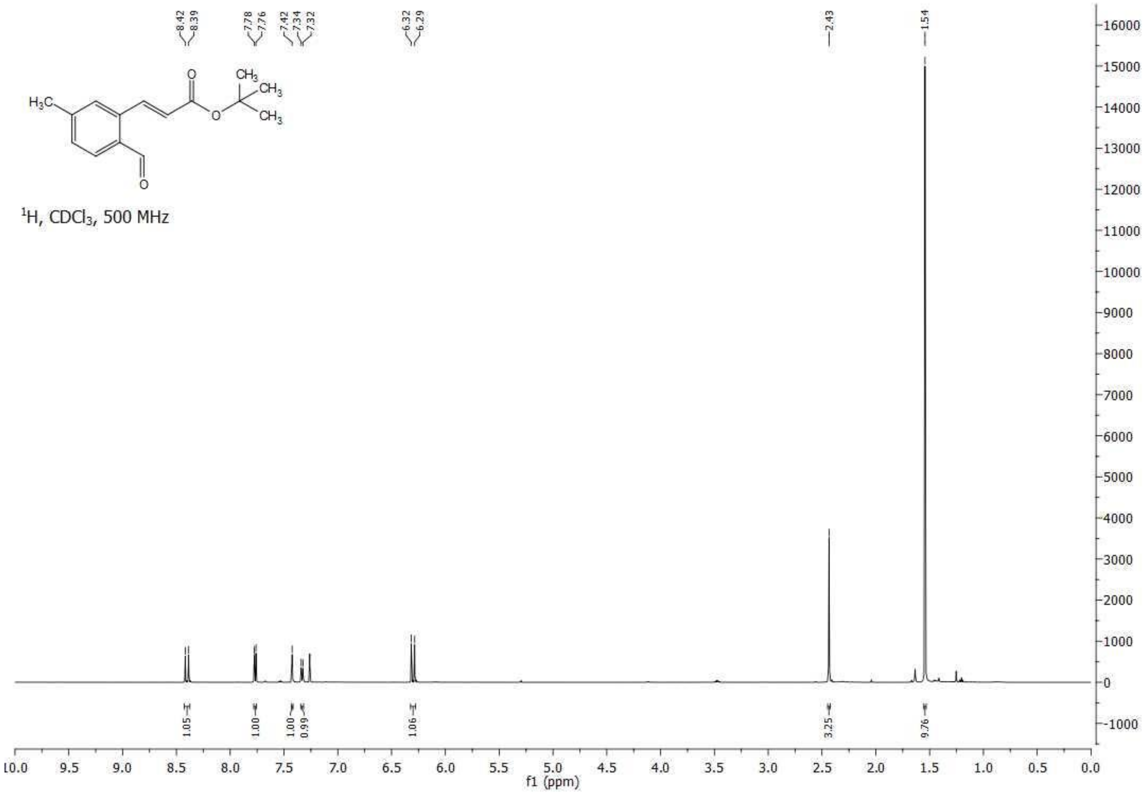

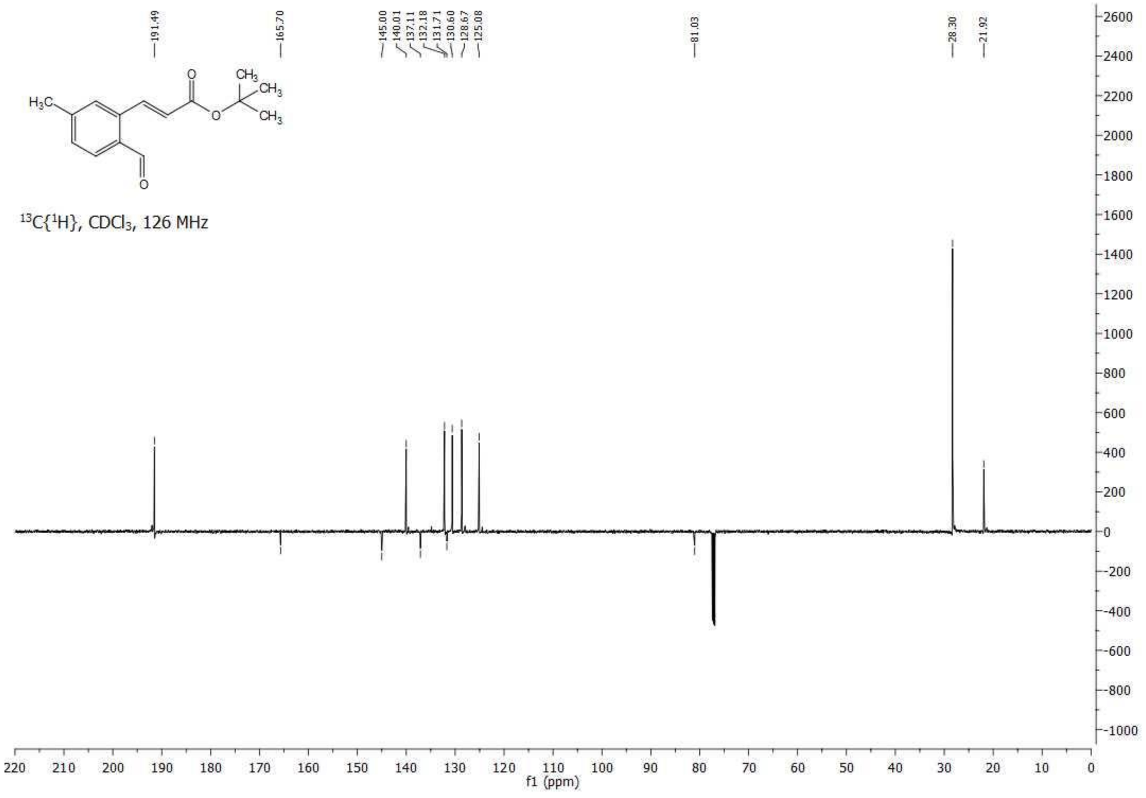

tert-Butyl (_E_)-3-(4-fluoro-2-formylphenyl)acrylate (S4)

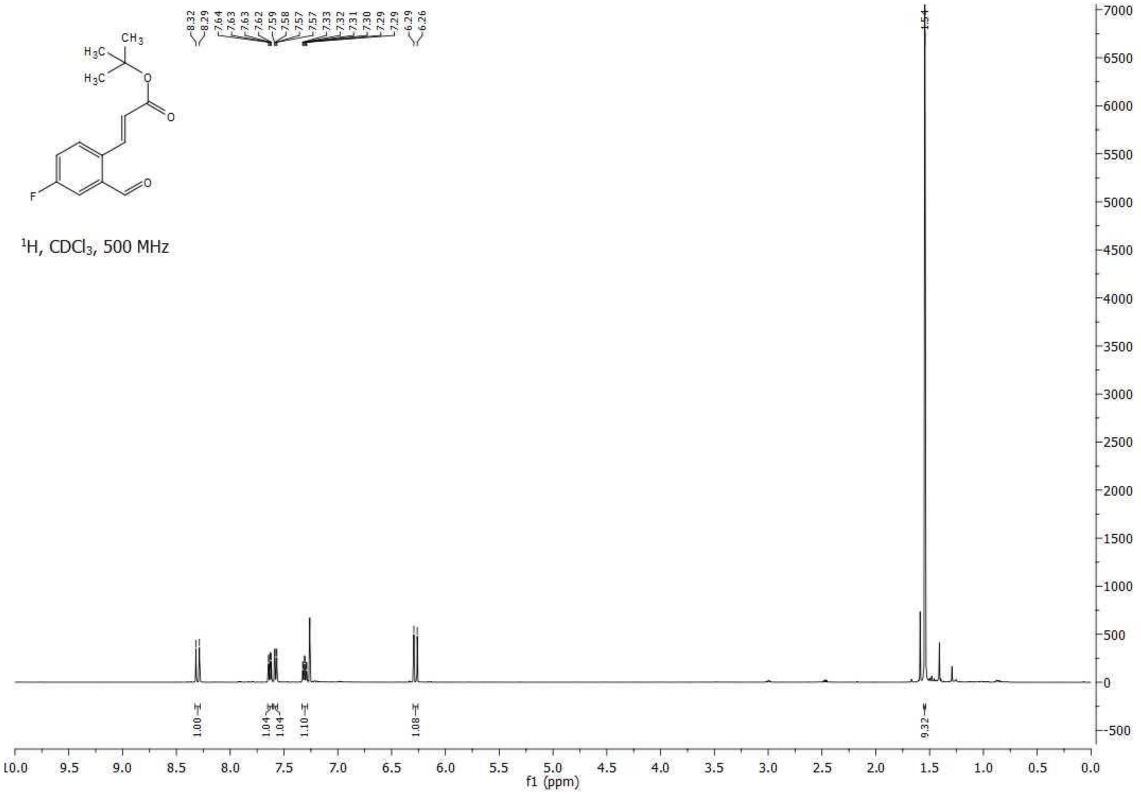

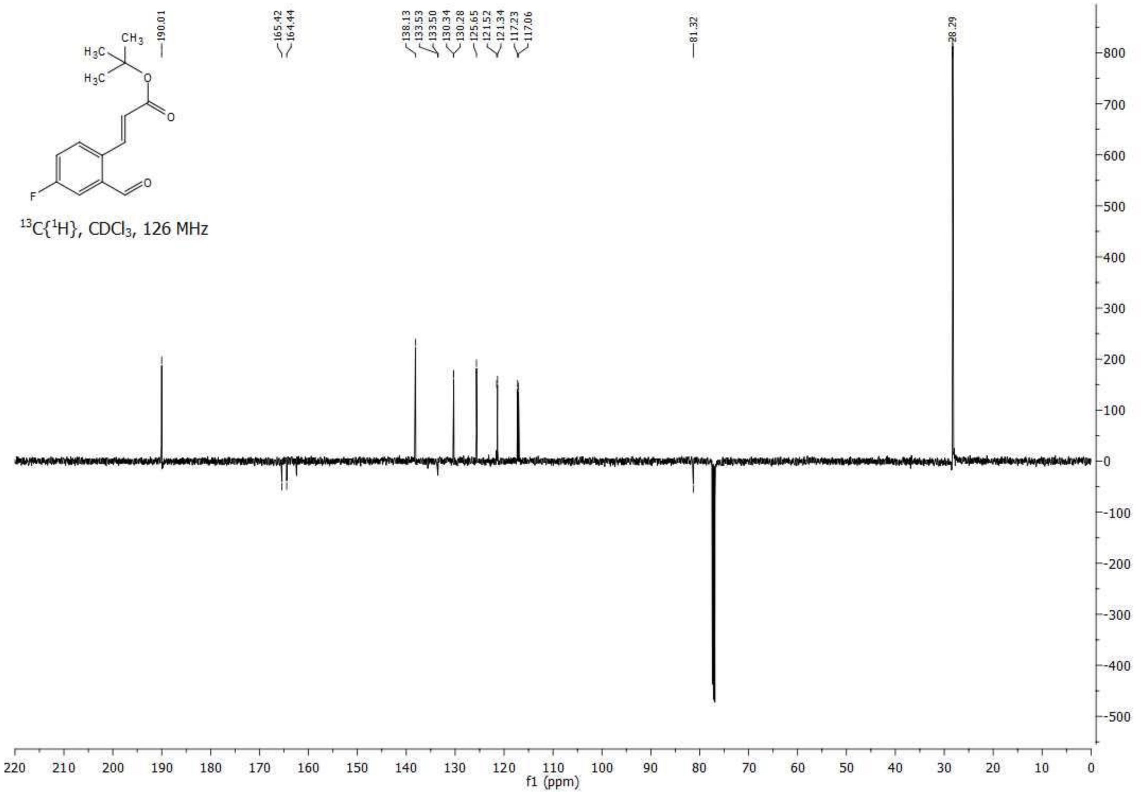

***tert*-Butyl (*E*)-3-(2-((*E*)-3-oxobut-1-en-1-yl)phenyl)acrylate (S8)**

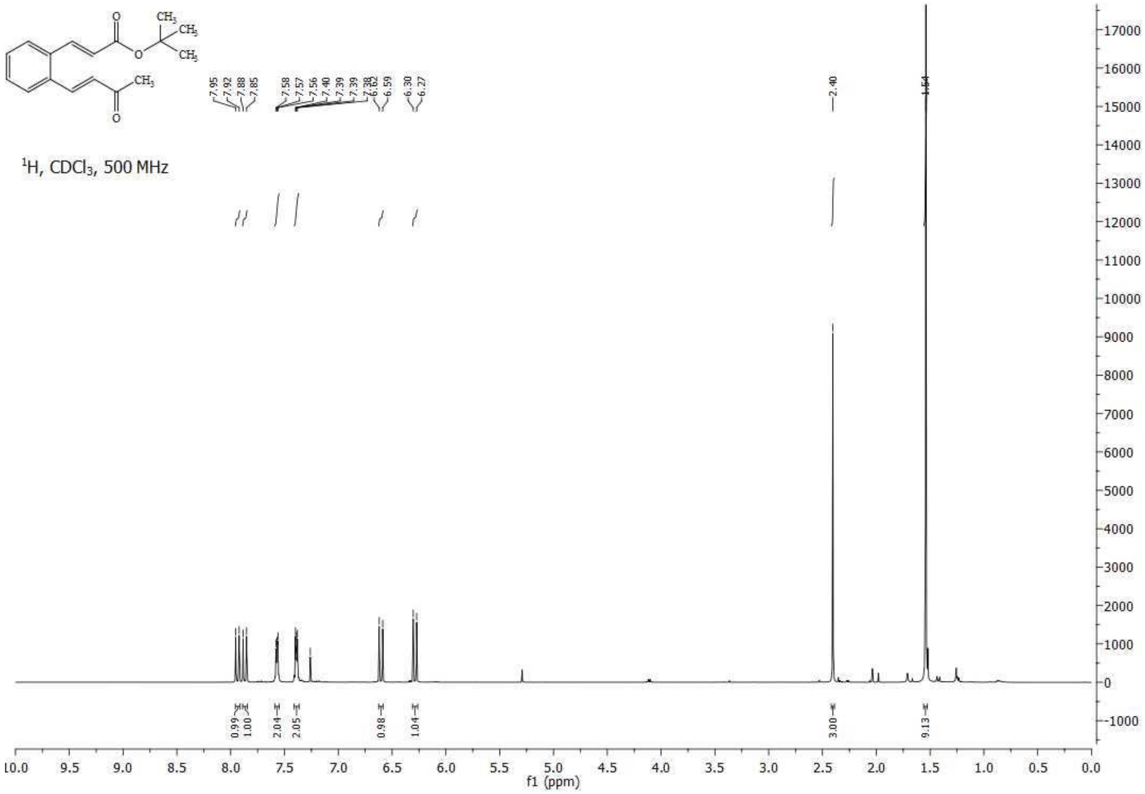

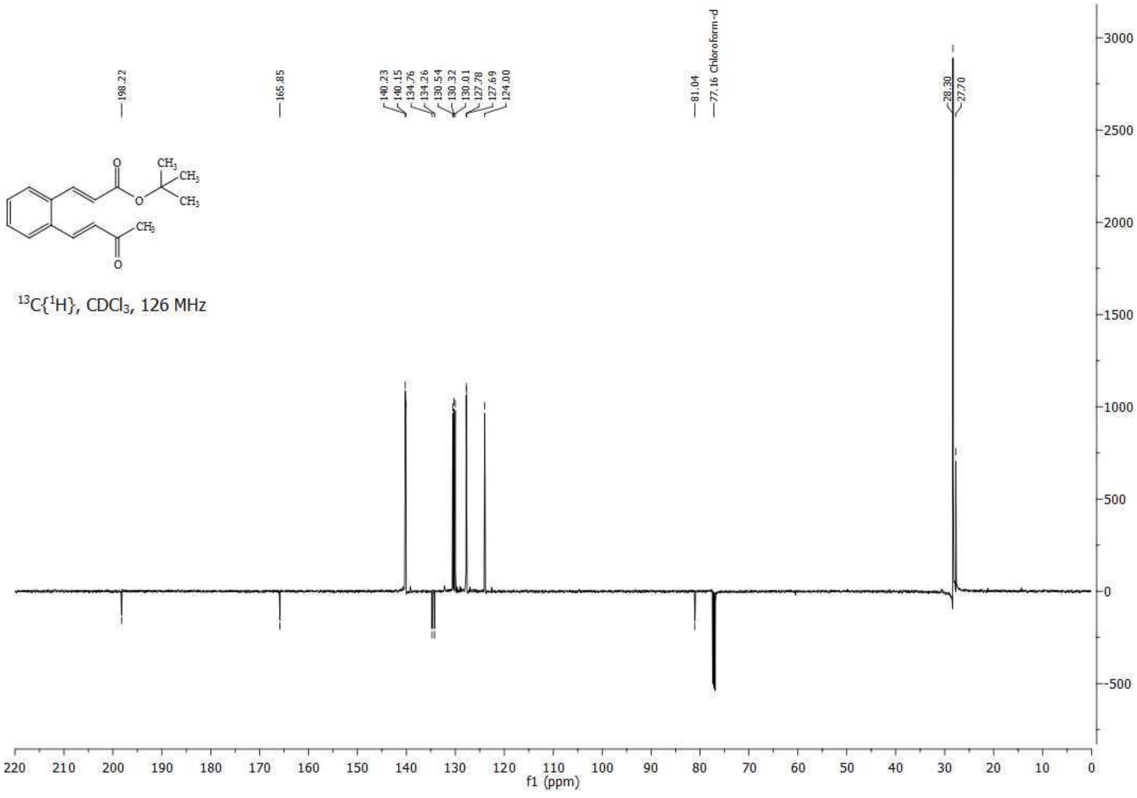

tert-Butyl (E)-3-(2-((E)-4,4-dimethyl-3-oxopent-1-en-1-yl)phenyl)acrylate (S9)

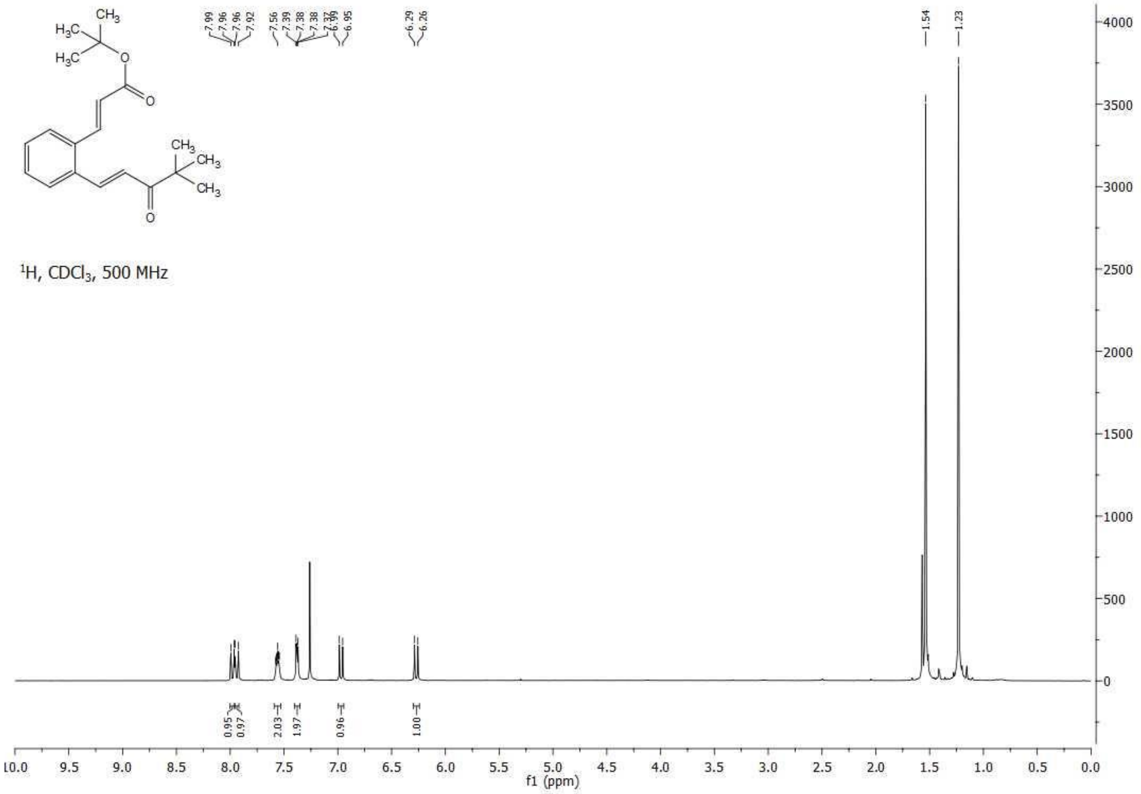

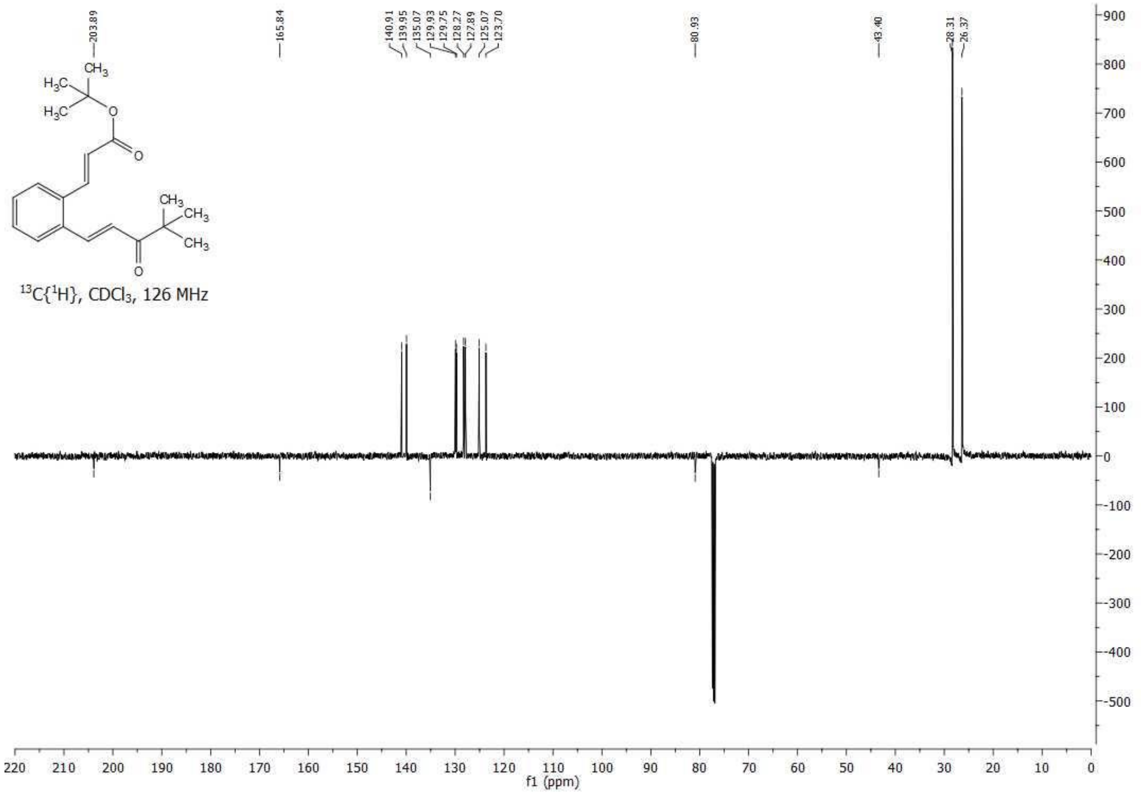

(*E*)-*tert*-Butyl 3-(2-((*E*)-3-oxo-3-phenylprop-1-en-1-yl)phenyl)acrylate (S10)

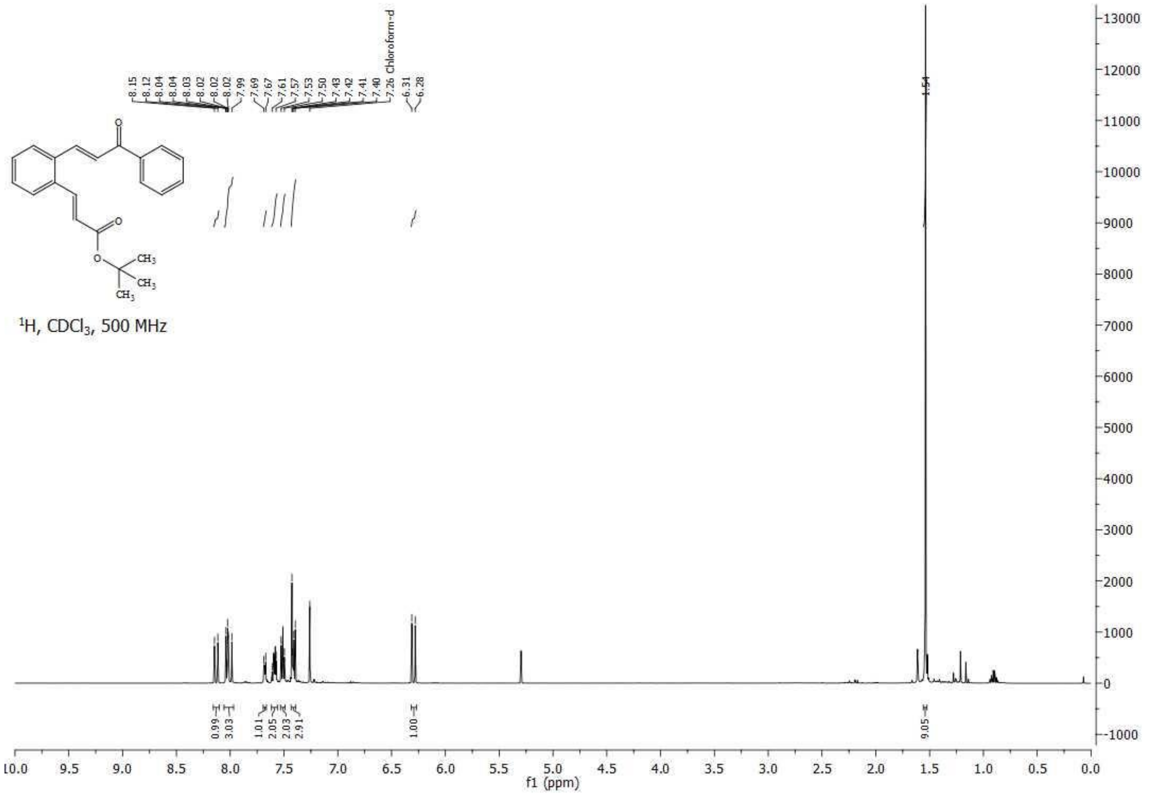

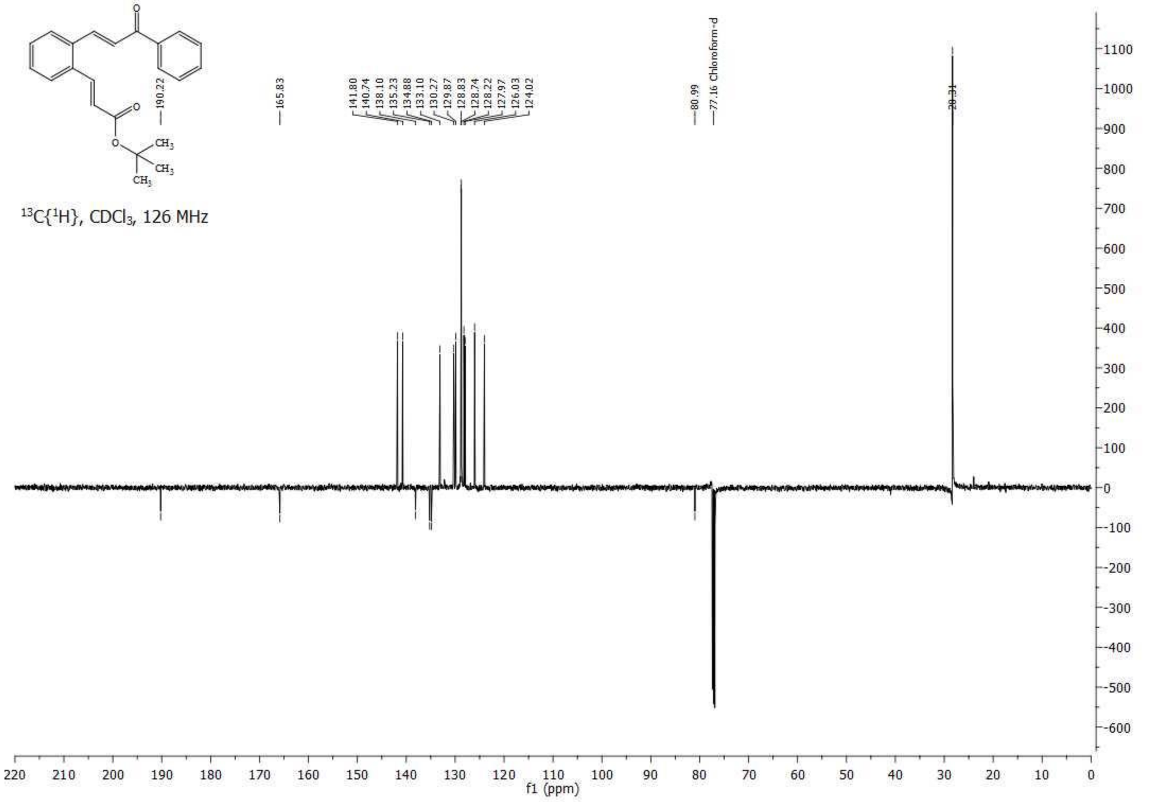

tert-Butyl (_E_)-3-(2-((_E_)-3-(4-bromophenyl)-3-oxoprop-1-en-1-yl)phenyl)acrylate (S11)

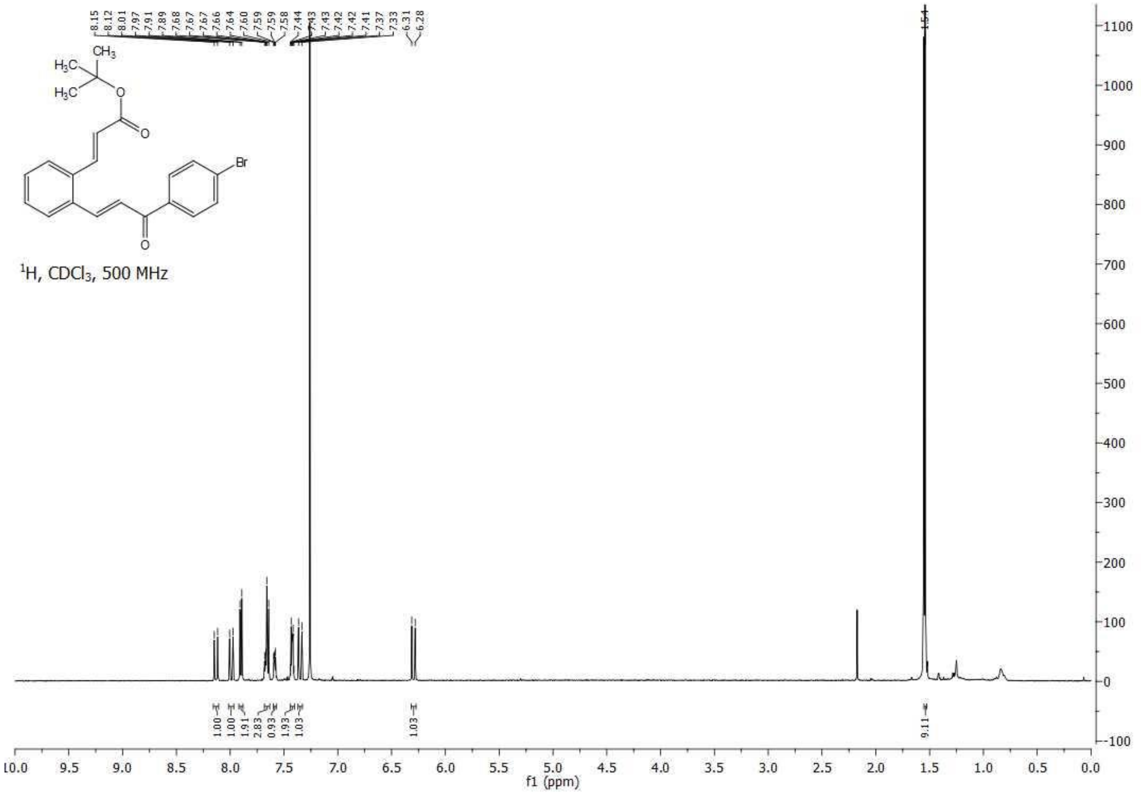

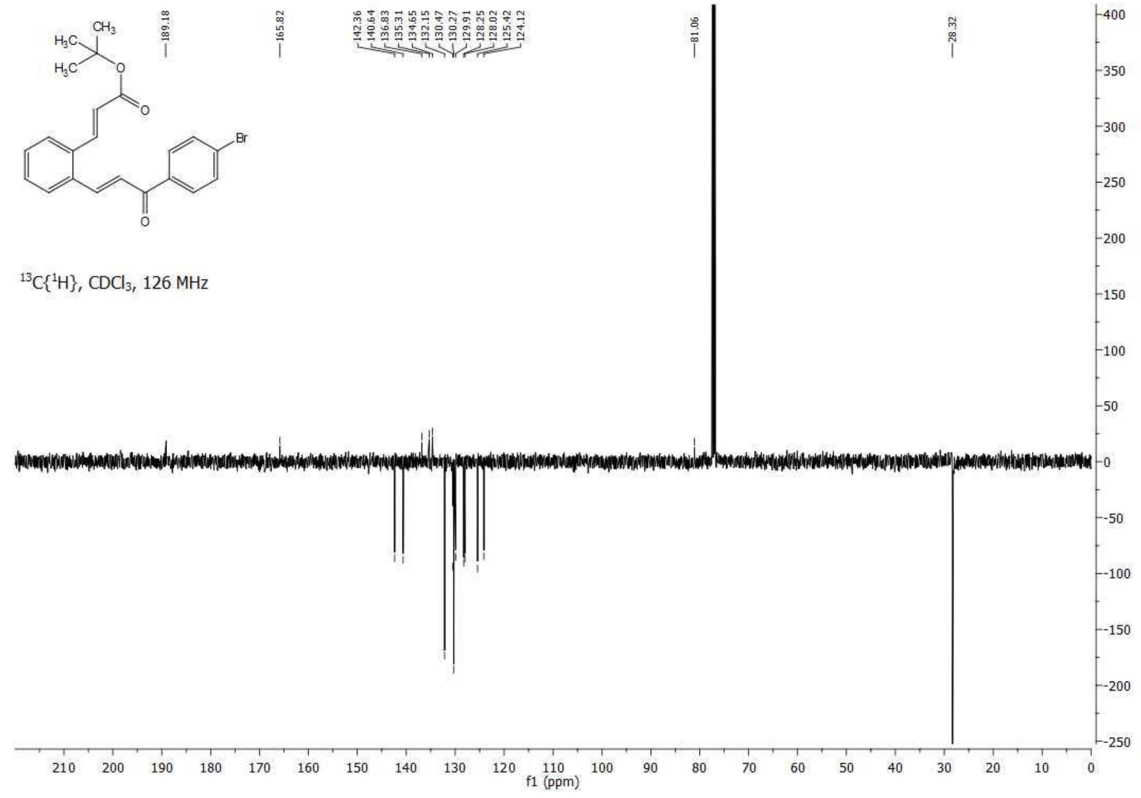

***tert*-Butyl (*E*)-3-(2-((*E*)-3-(4-chlorophenyl)-3-oxoprop-1-en-1-yl)phenyl)acrylate (S12)**

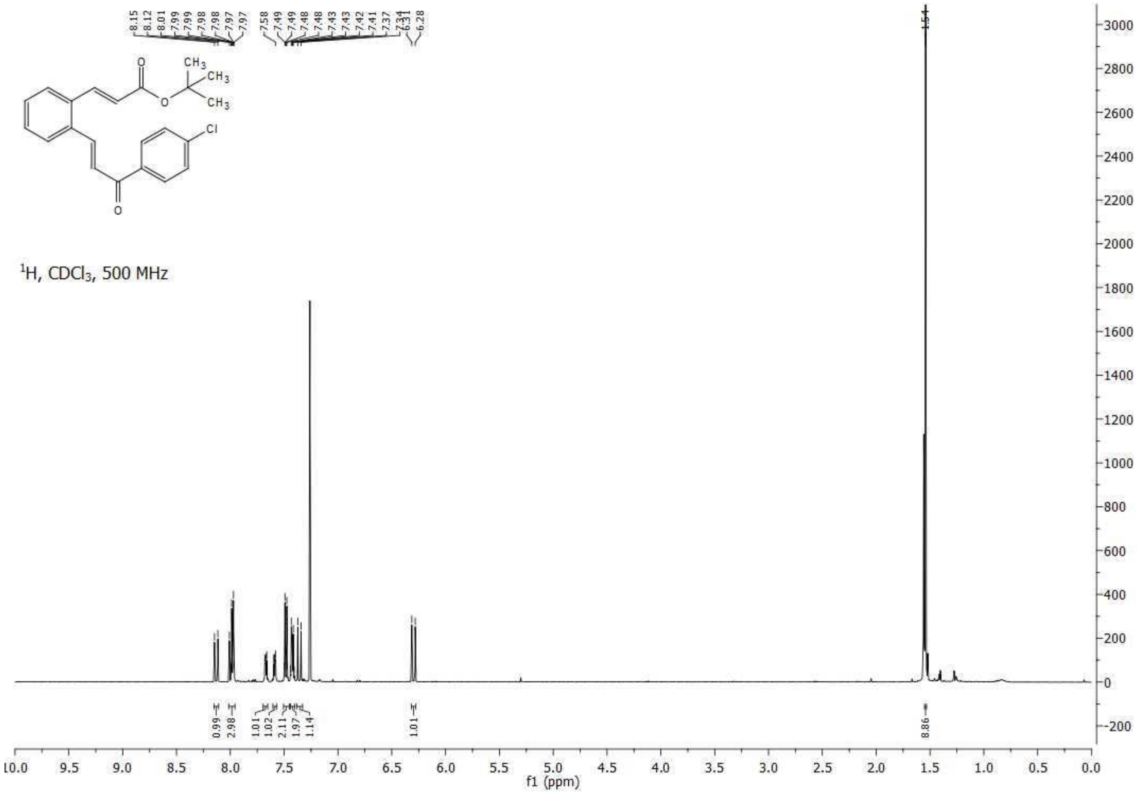

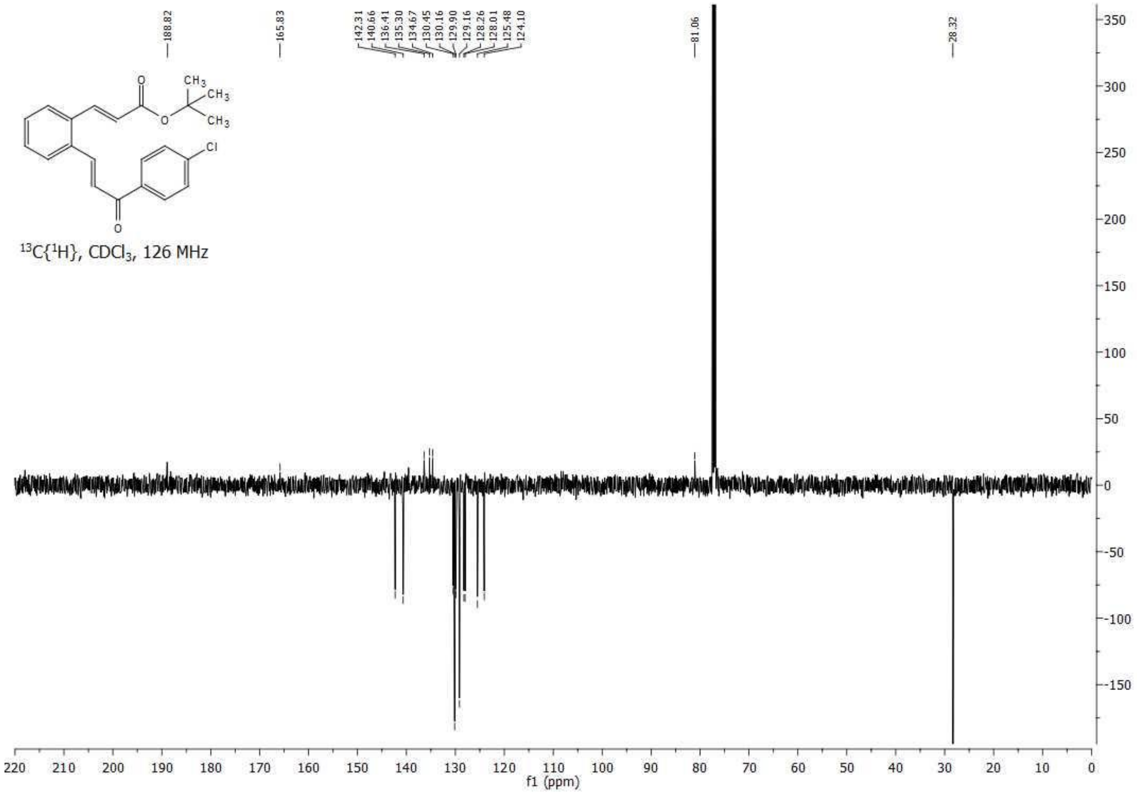

***tert*-Butyl (*E*)-3-(2-((*E*)-3-(4-trifluoromethyl)phenyl)-3-oxoprop-1-en-1-yl)phenyl)acrylate (S13)**

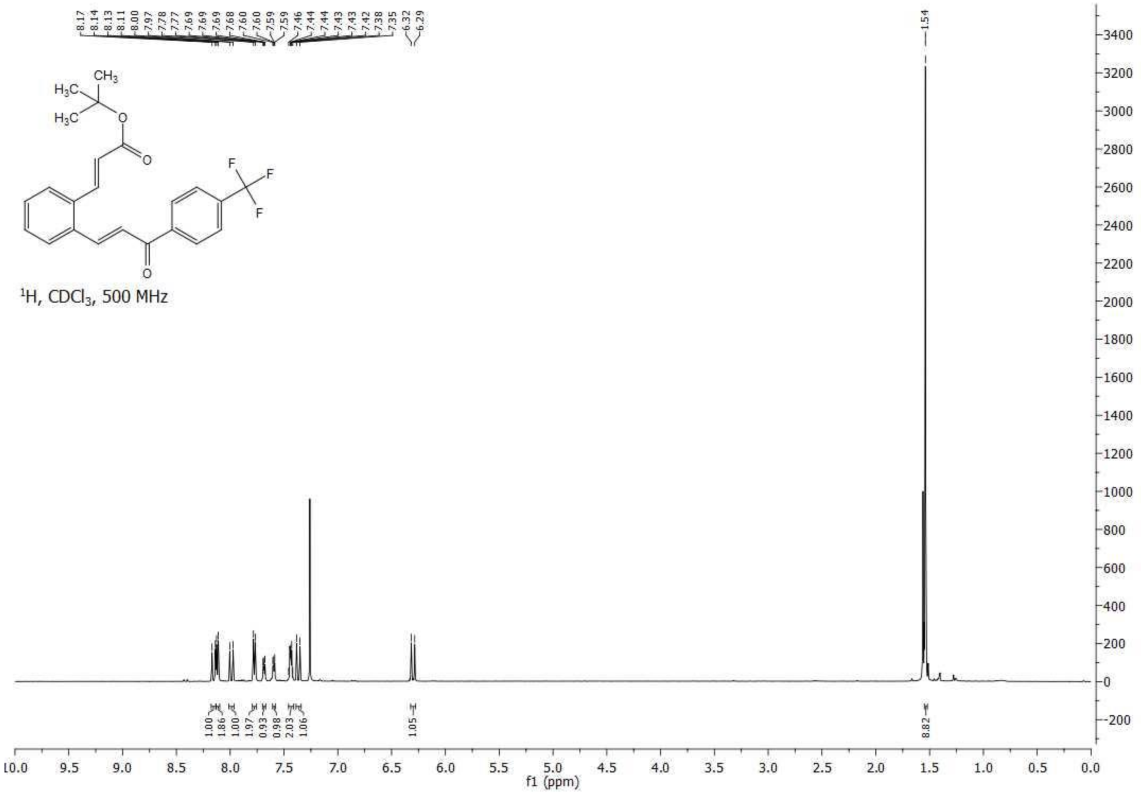

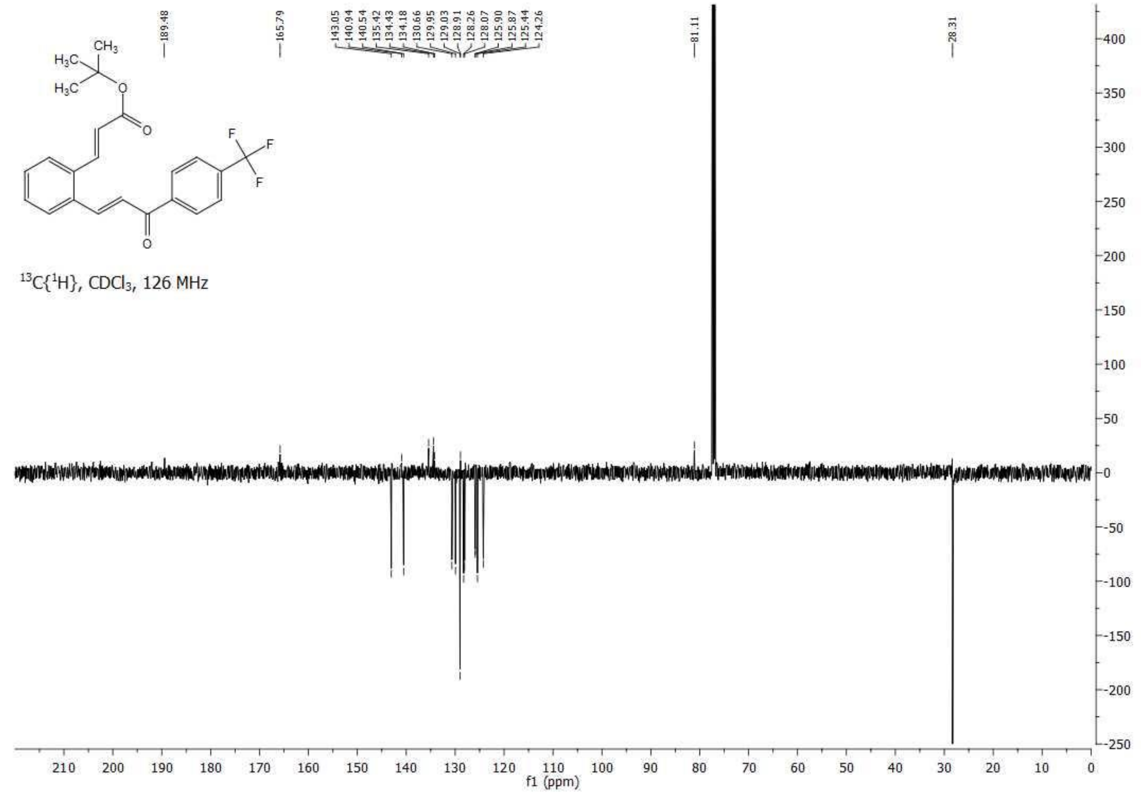

***tert*-Butyl (*E*)-3-(2-((*E*)-3-(4-methoxy)phenyl)-3-oxoprop-1-en-1-yl)phenyl)acrylate (S14)**

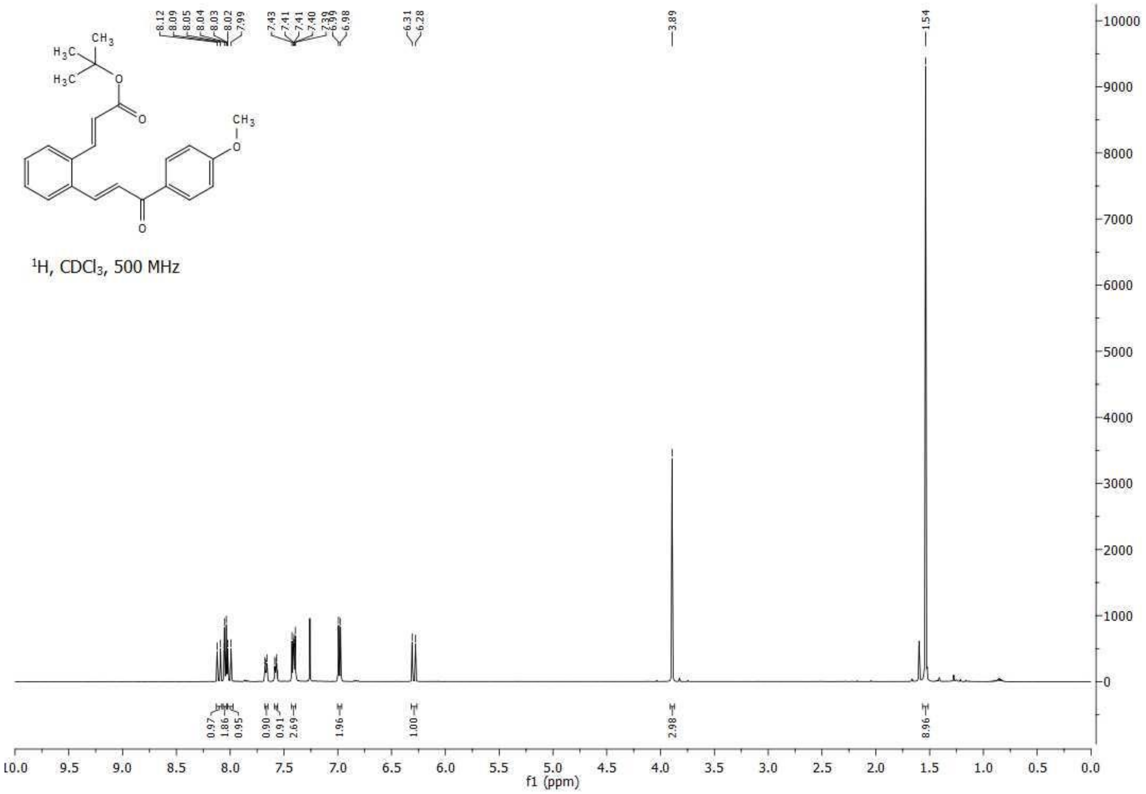

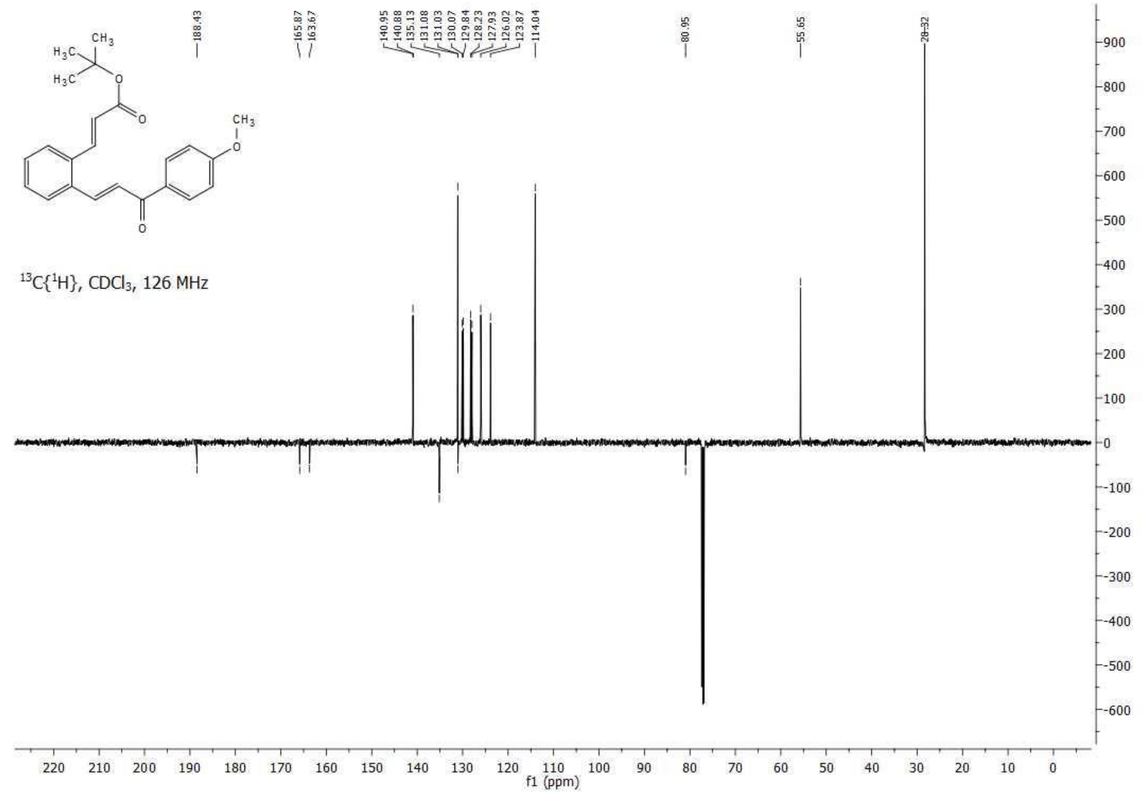

***tert*-Butyl (*E*)-3-(4-chloro-2-((*E*)-3-oxobut-1-en-1-yl)phenyl)acrylate (S15)**

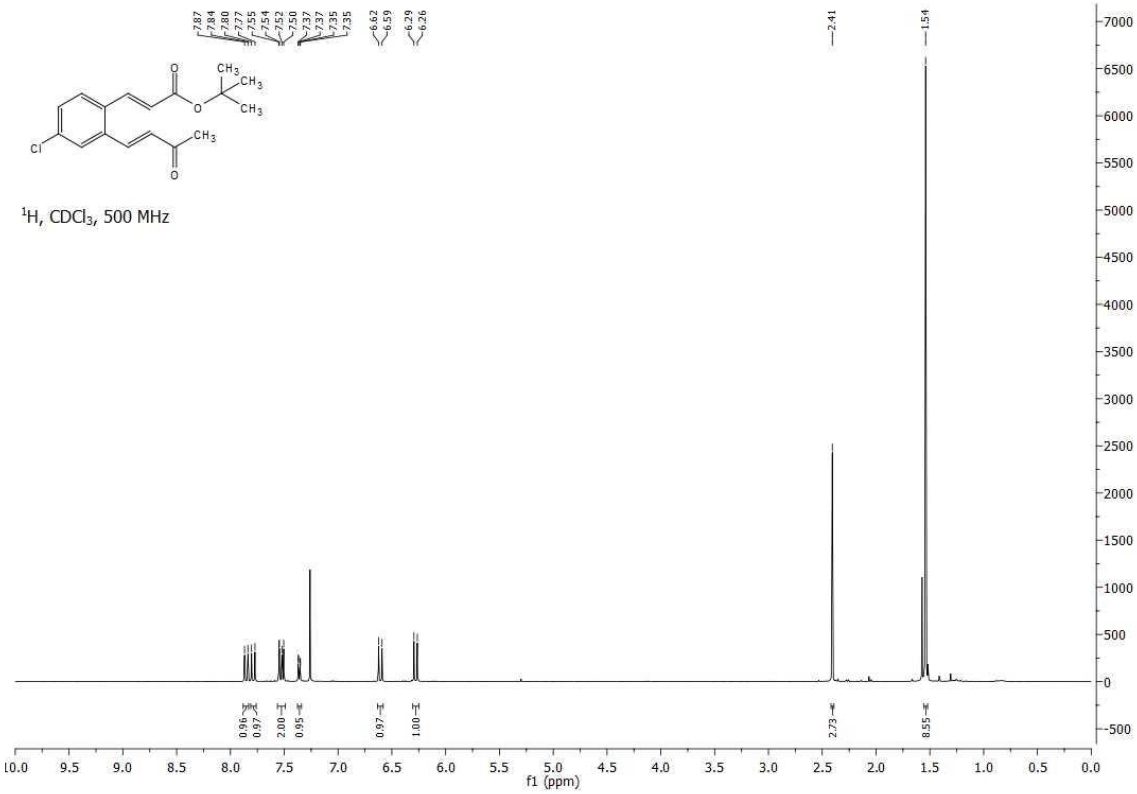

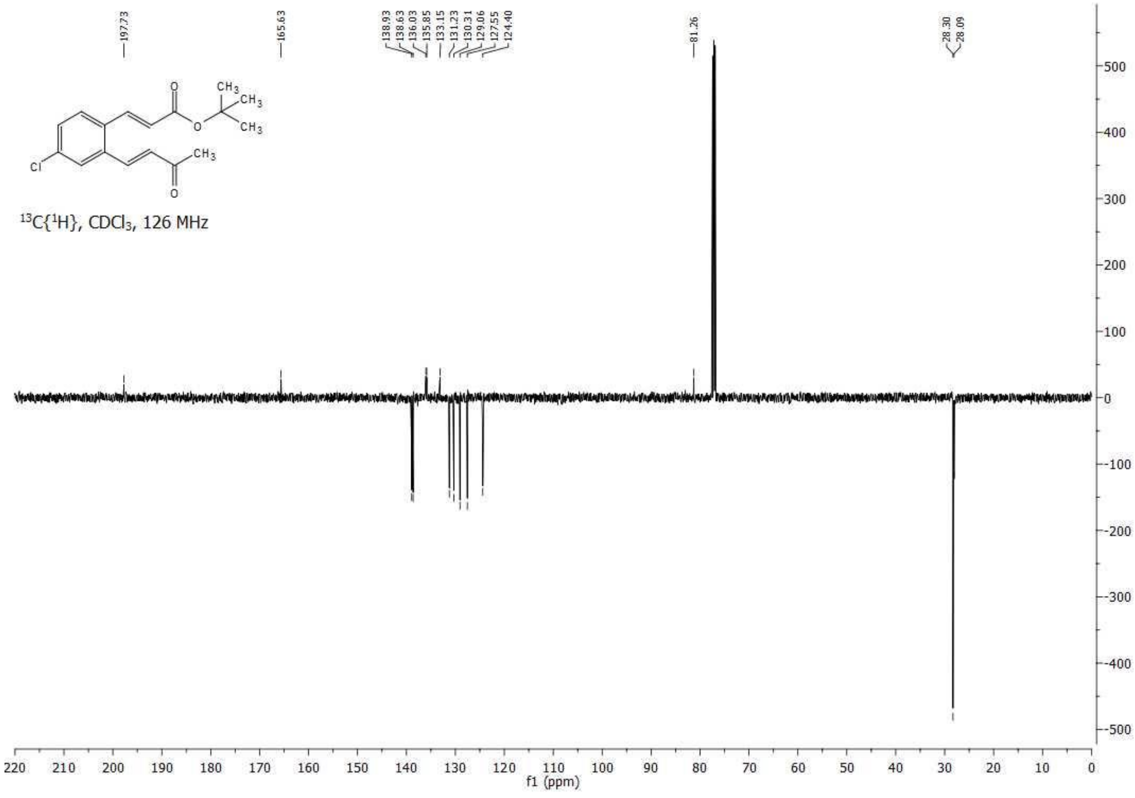

tert-Butyl (E)-3-(5-methyl-2-((E)-3-oxobut-1-en-1-yl)phenyl)acrylate (S16)

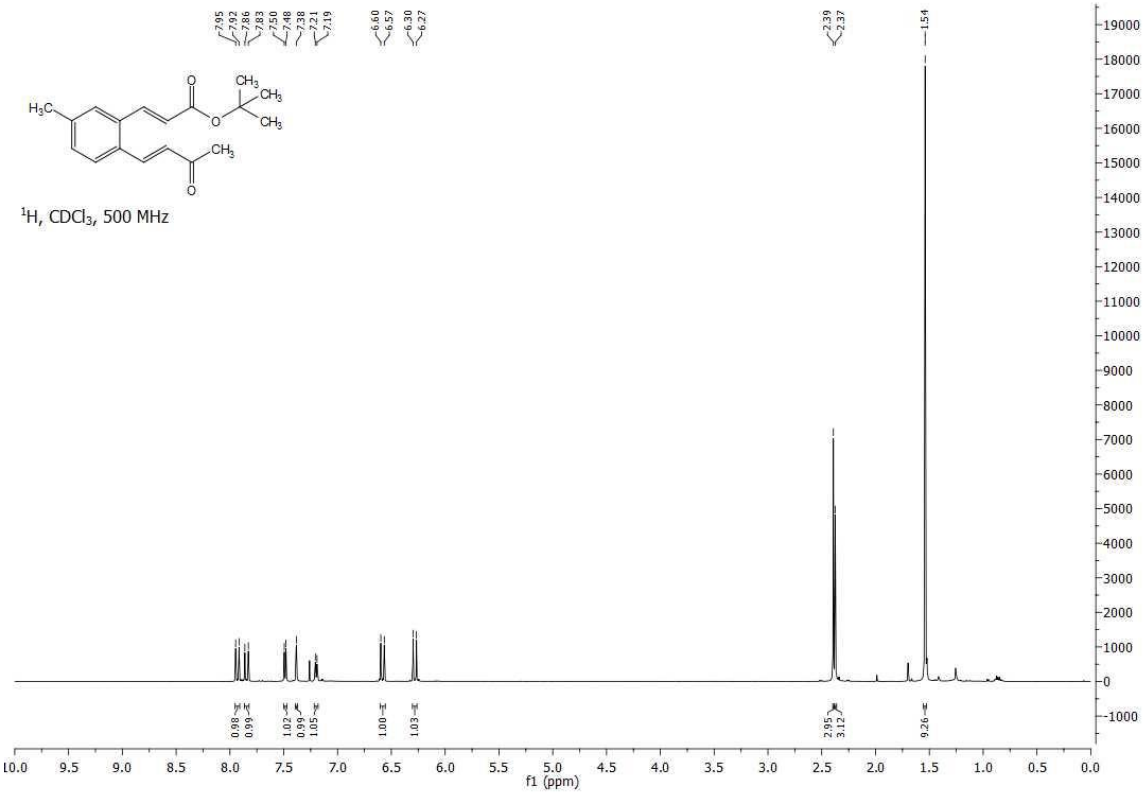

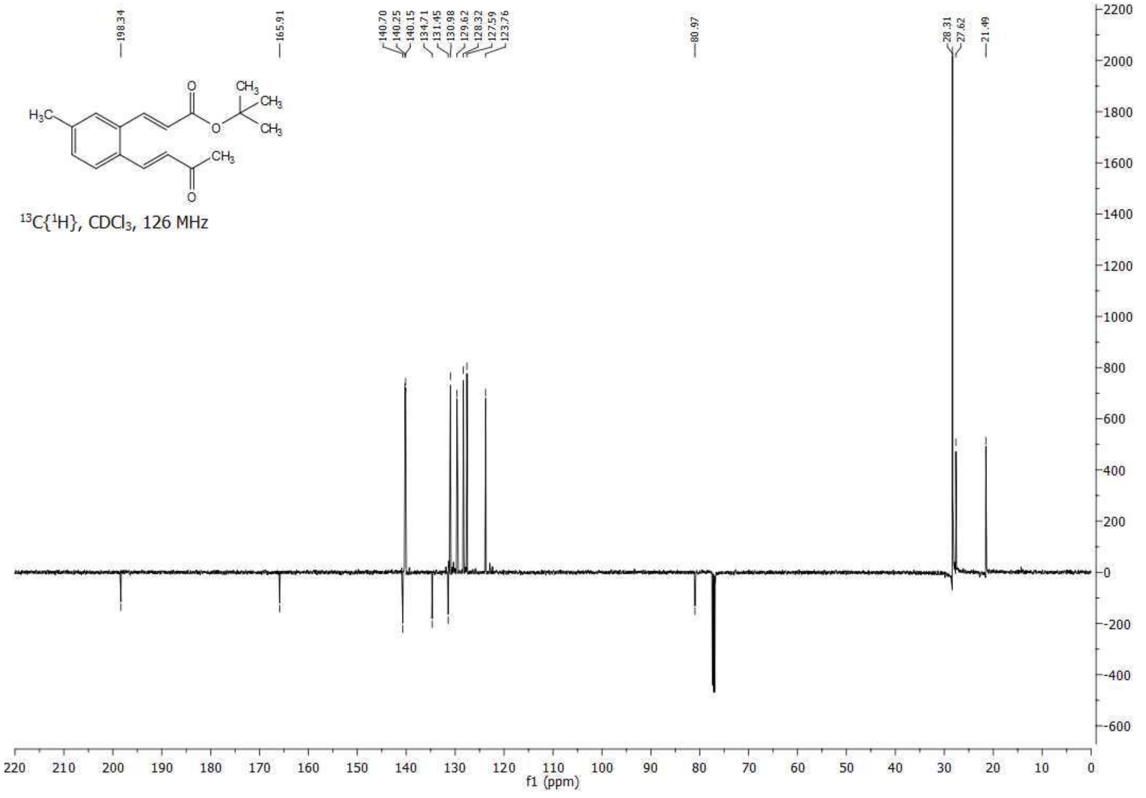

***tert*-Butyl (*E*)-3-(4-fluoro-2-((*E*)-3-oxobut-1-en-1-yl)phenyl)acrylate (S17)**

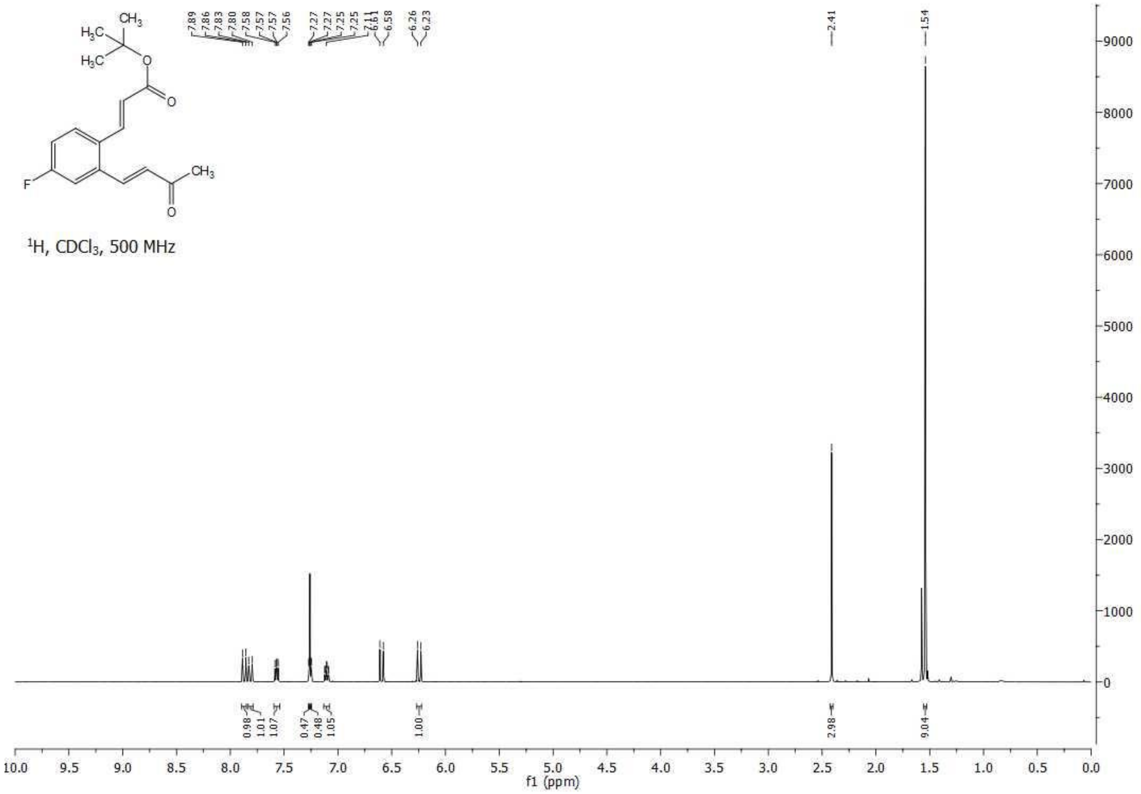

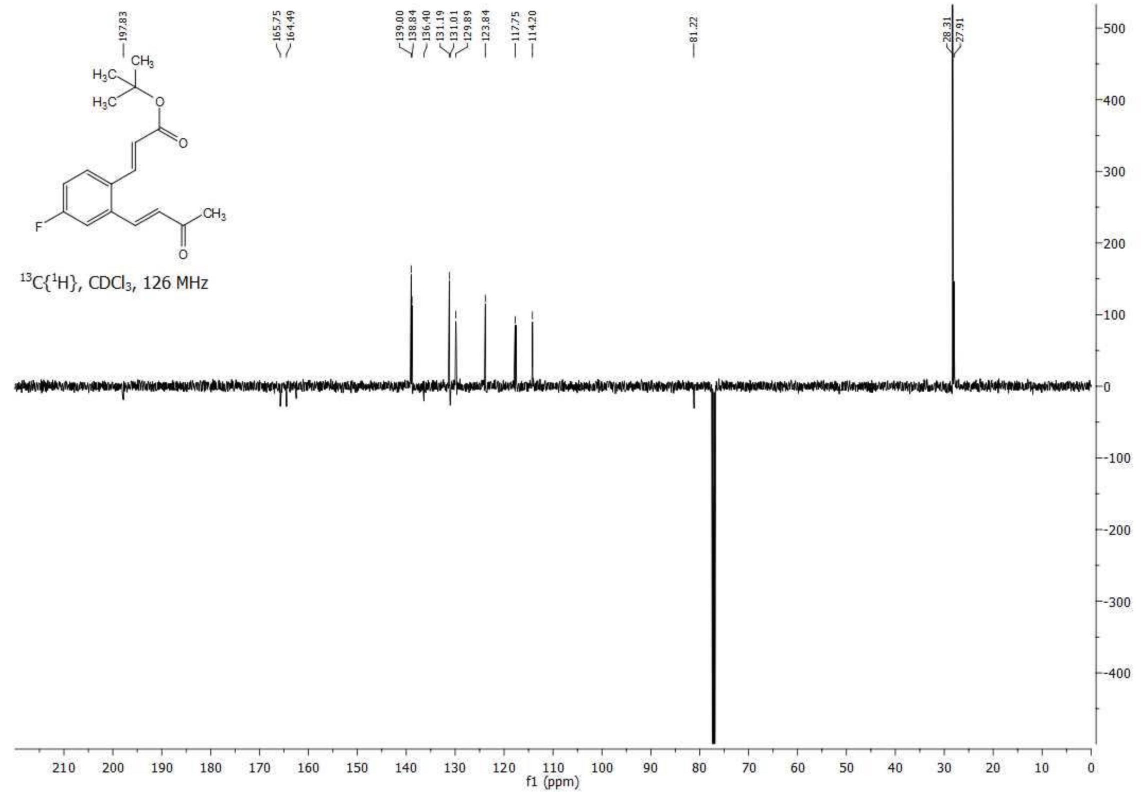

(*E*)-3-(2-((*E*)-3-Oxobut-1-en-1-yl)phenyl)acrylic acid (S18)

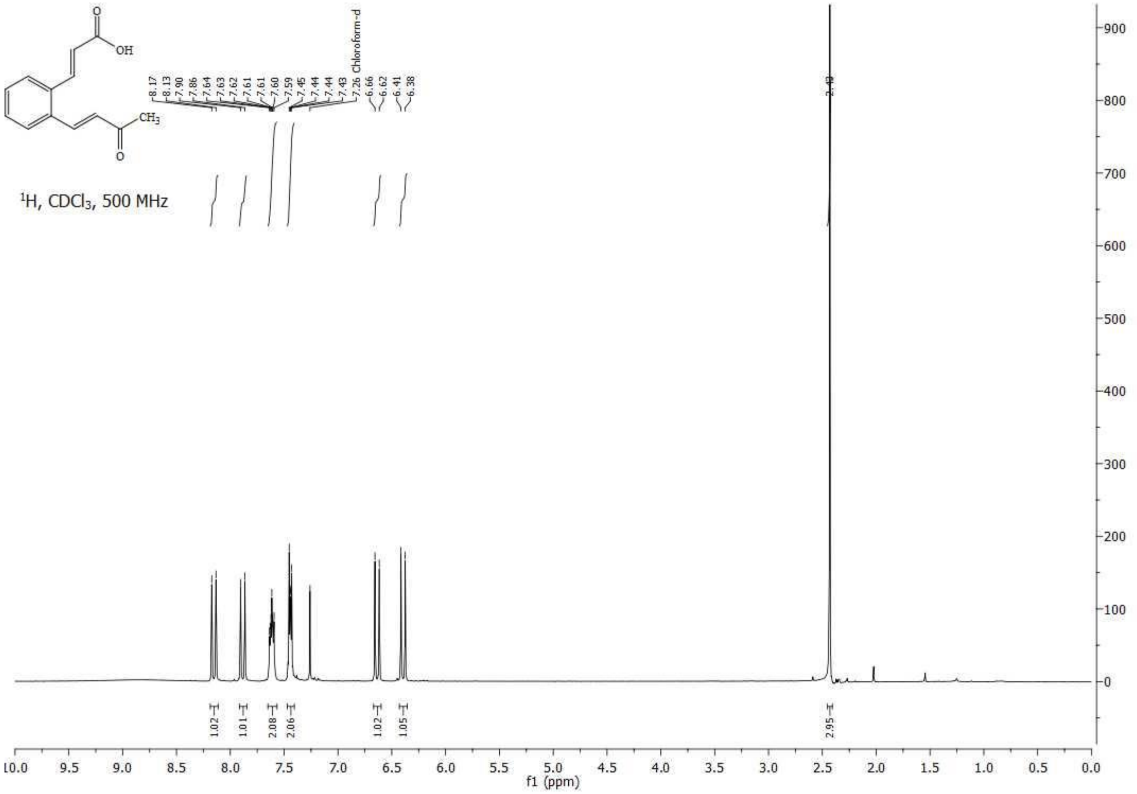

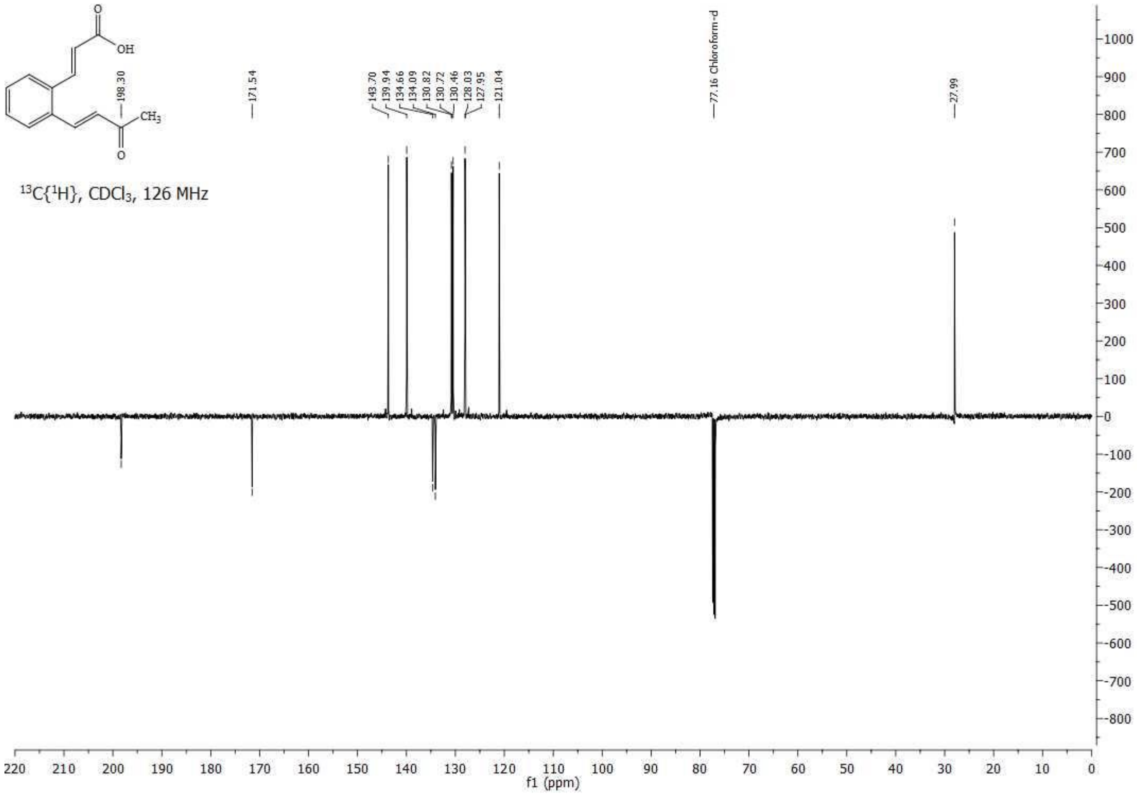

(*E*)-3-(2-((*E*)-4,4-Dimethyl-3-oxopent-1-en-1-yl)phenyl)acrylic acid (S19)

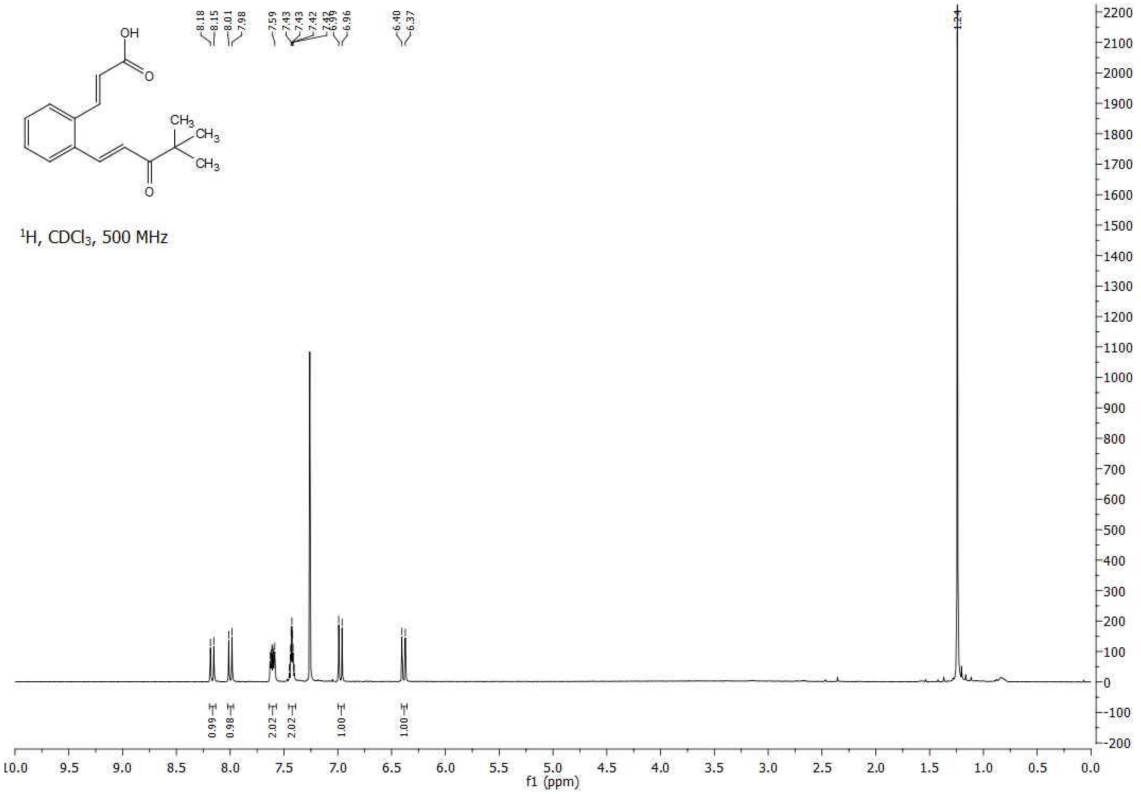

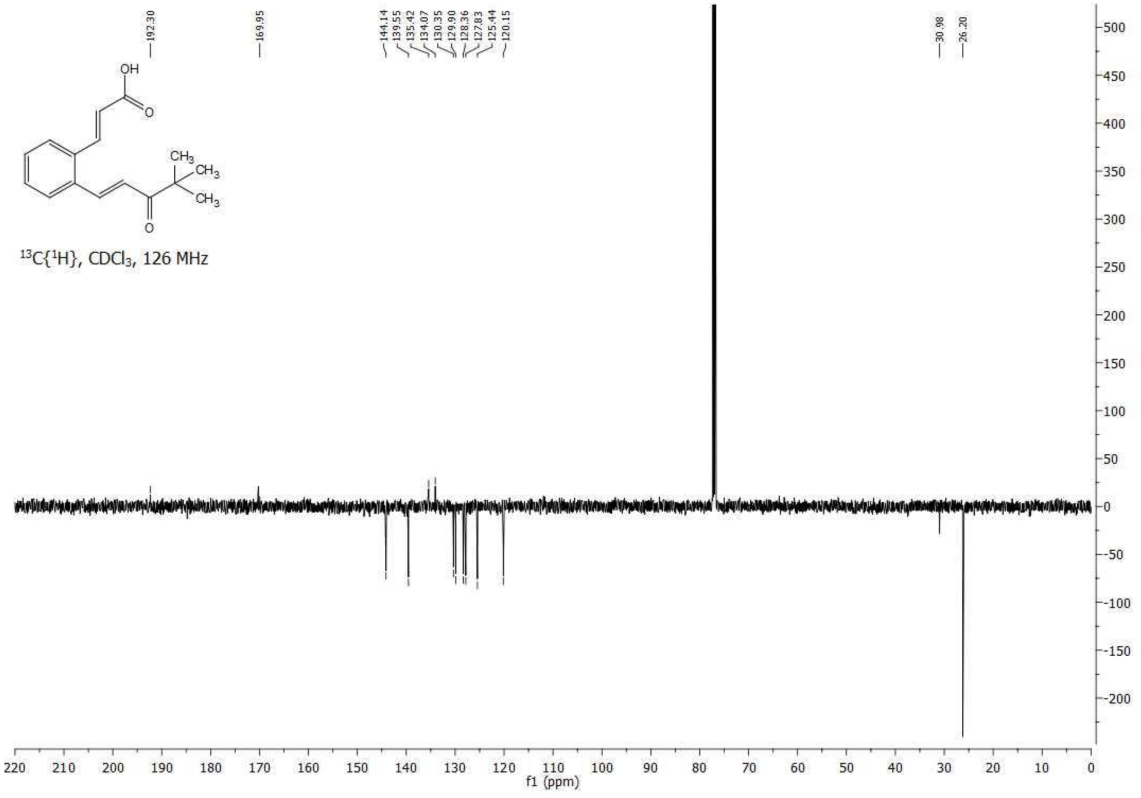

(*E*)-3-(2-((*E*)-3-Oxo-3-phenylprop-1-en-1-yl)phenyl)acrylic acid (S20)

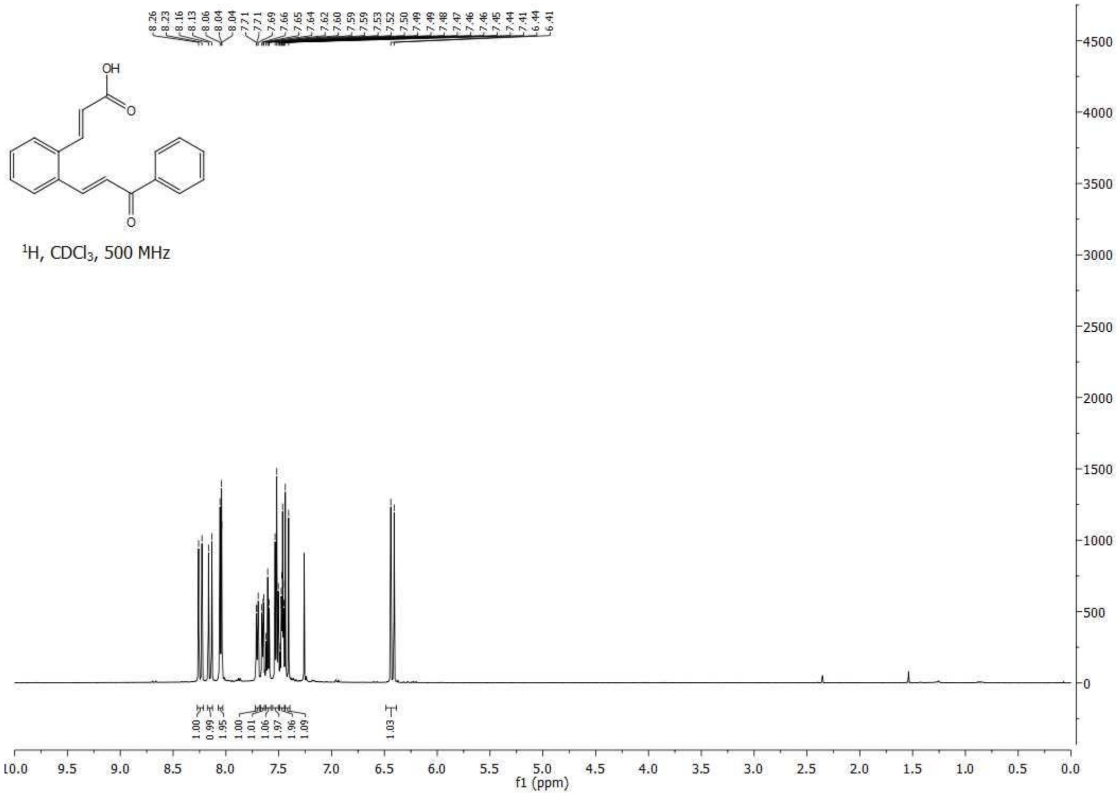

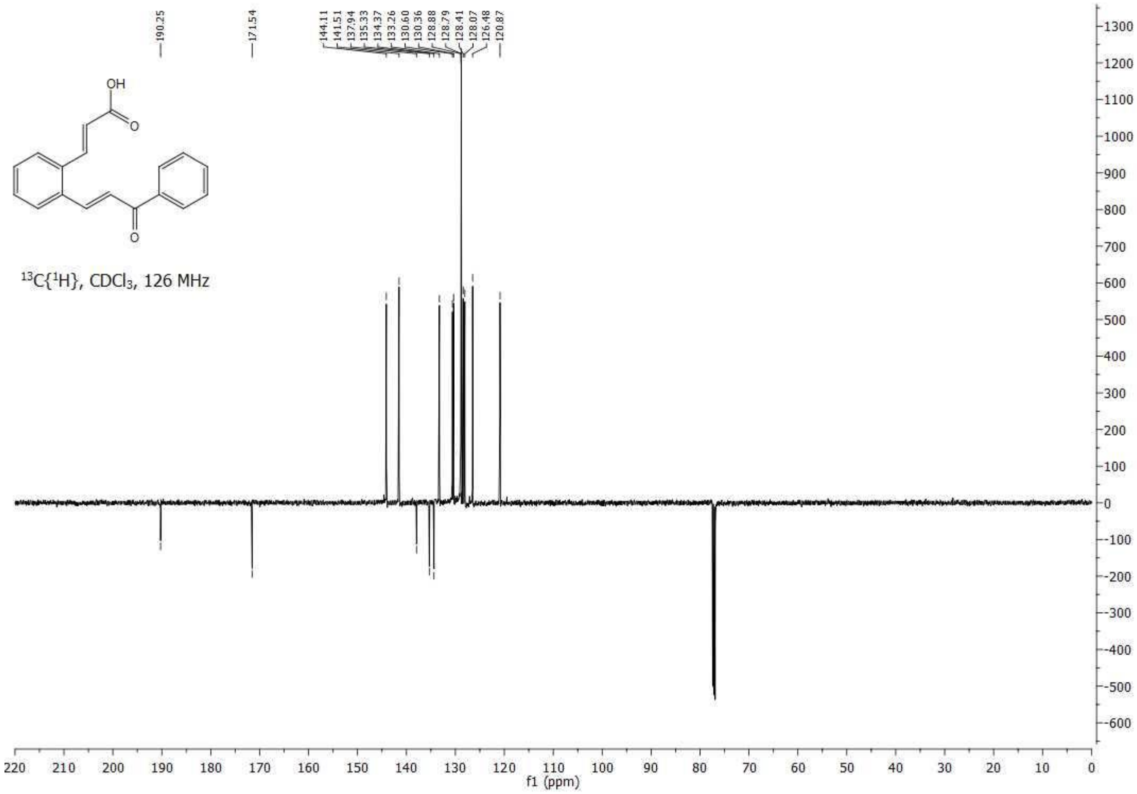

(*E*)-3-(2-((*E*)-3-(4-Bromophenyl)-3-oxoprop-1-en-1-yl)phenyl)acrylic acid (S21)

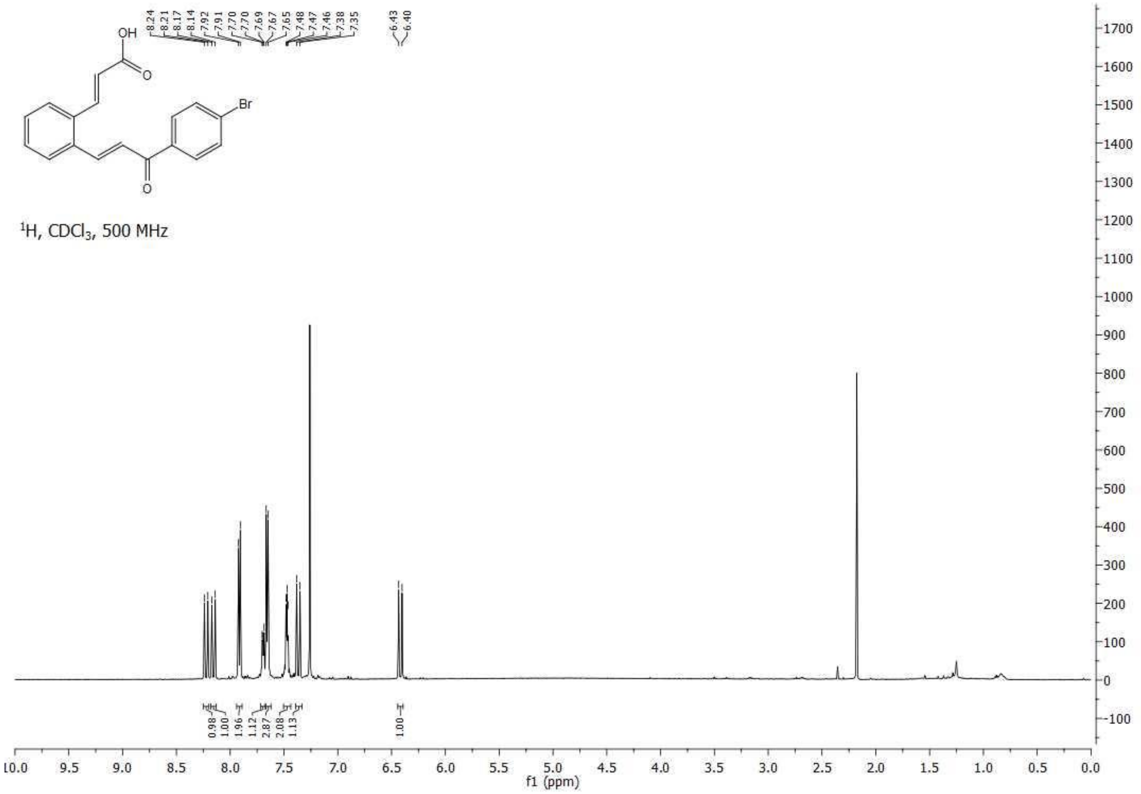

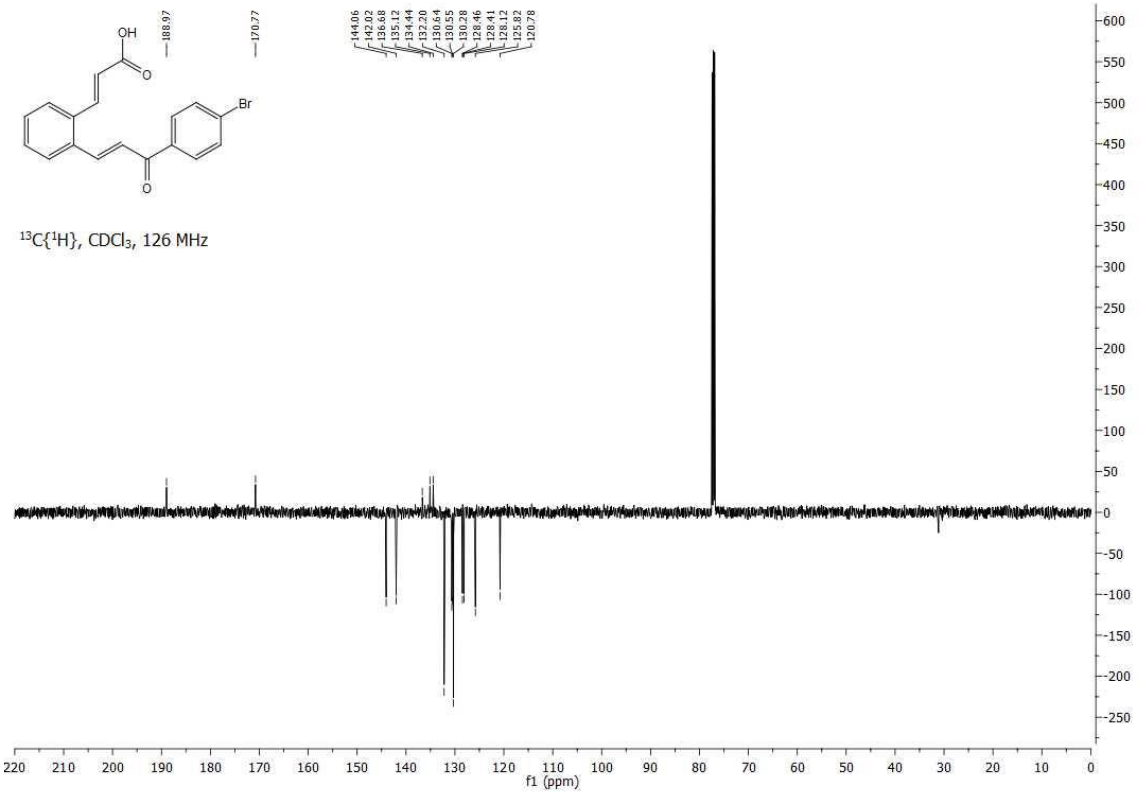

(*E*)-3-(2-((*E*)-3-(4-Chlorophenyl)-3-oxoprop-1-en-1-yl)phenyl)acrylic acid (S22)

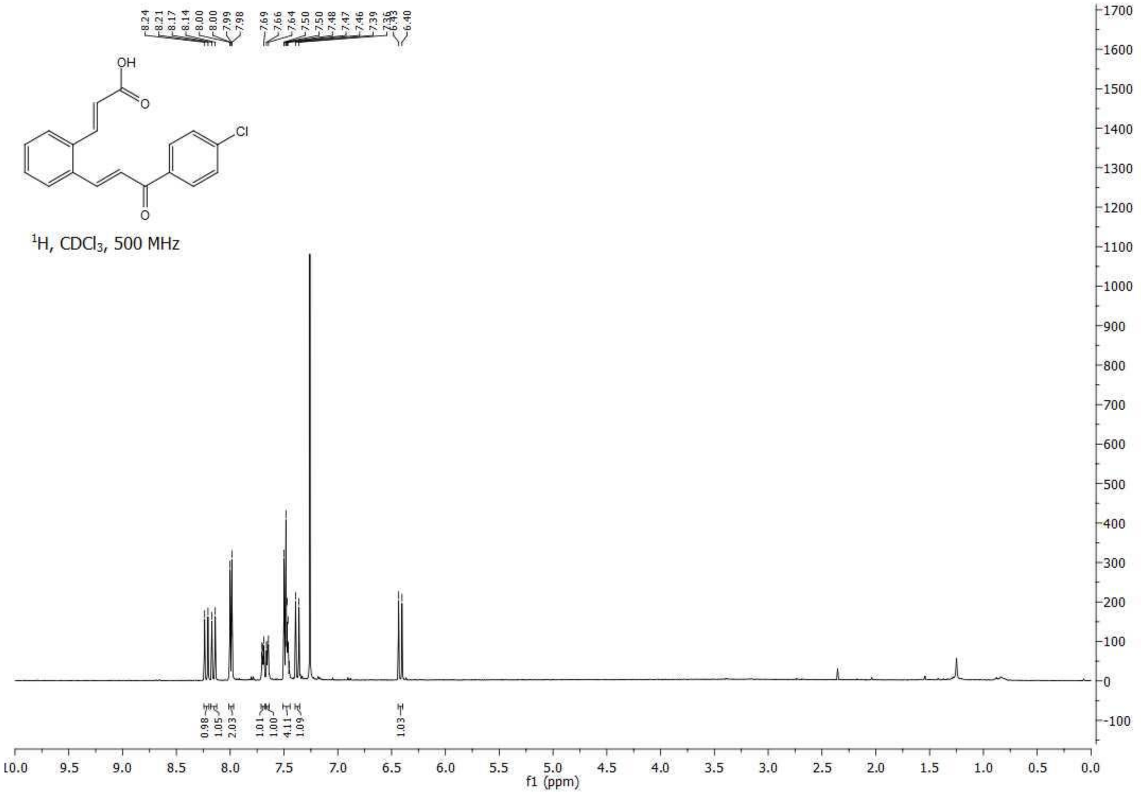

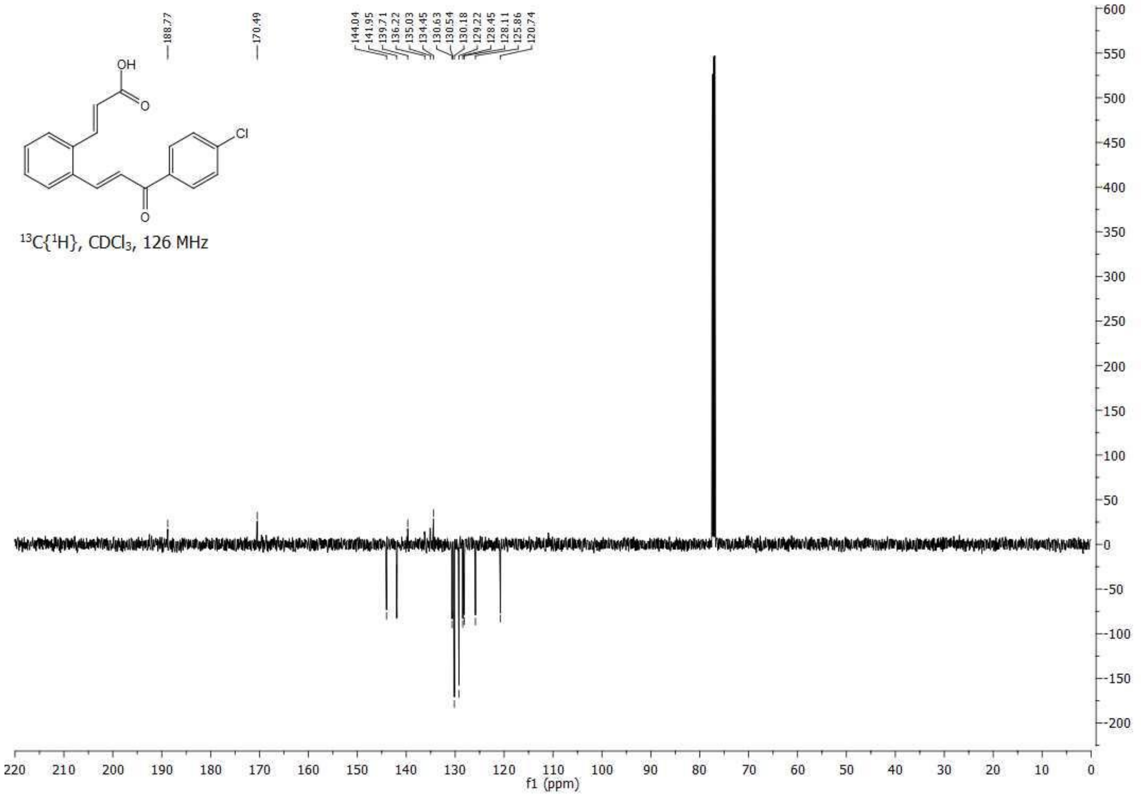

(*E*)-3-(2-((*E*)-3-(4-Trifluoromethyl)phenyl)-3-oxoprop-1-en-1-yl)phenyl)acrylic acid (S23)

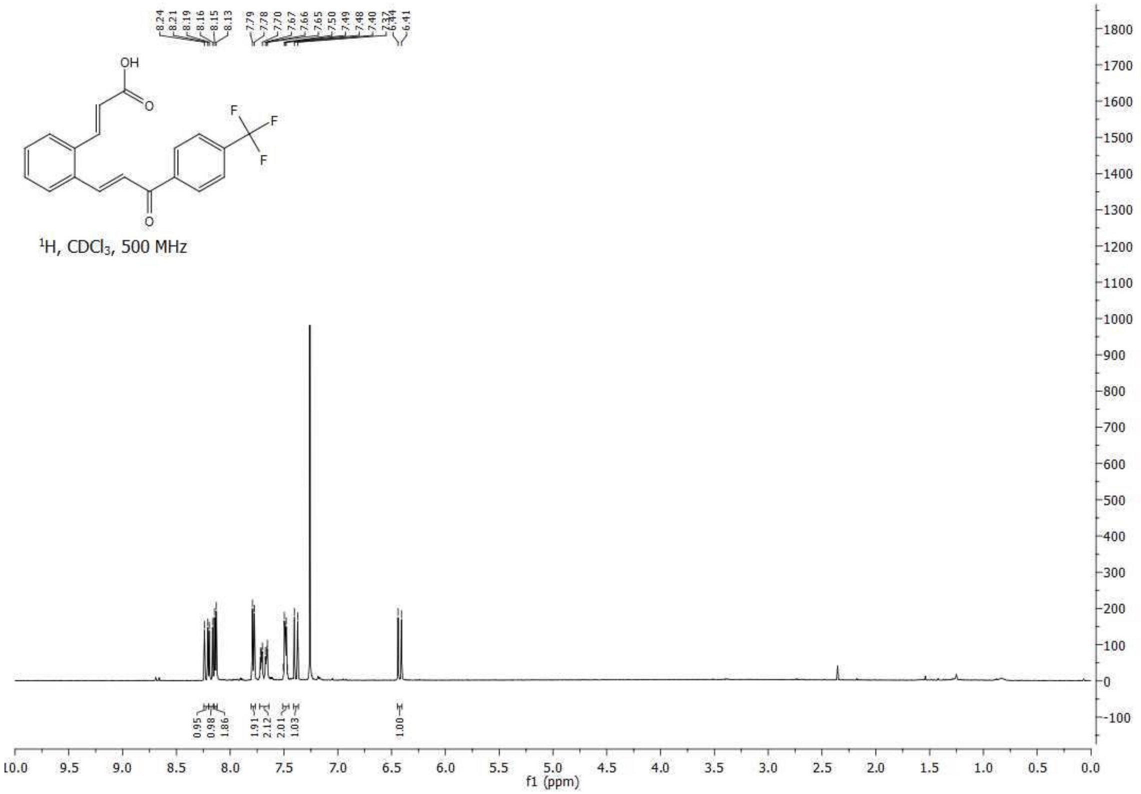

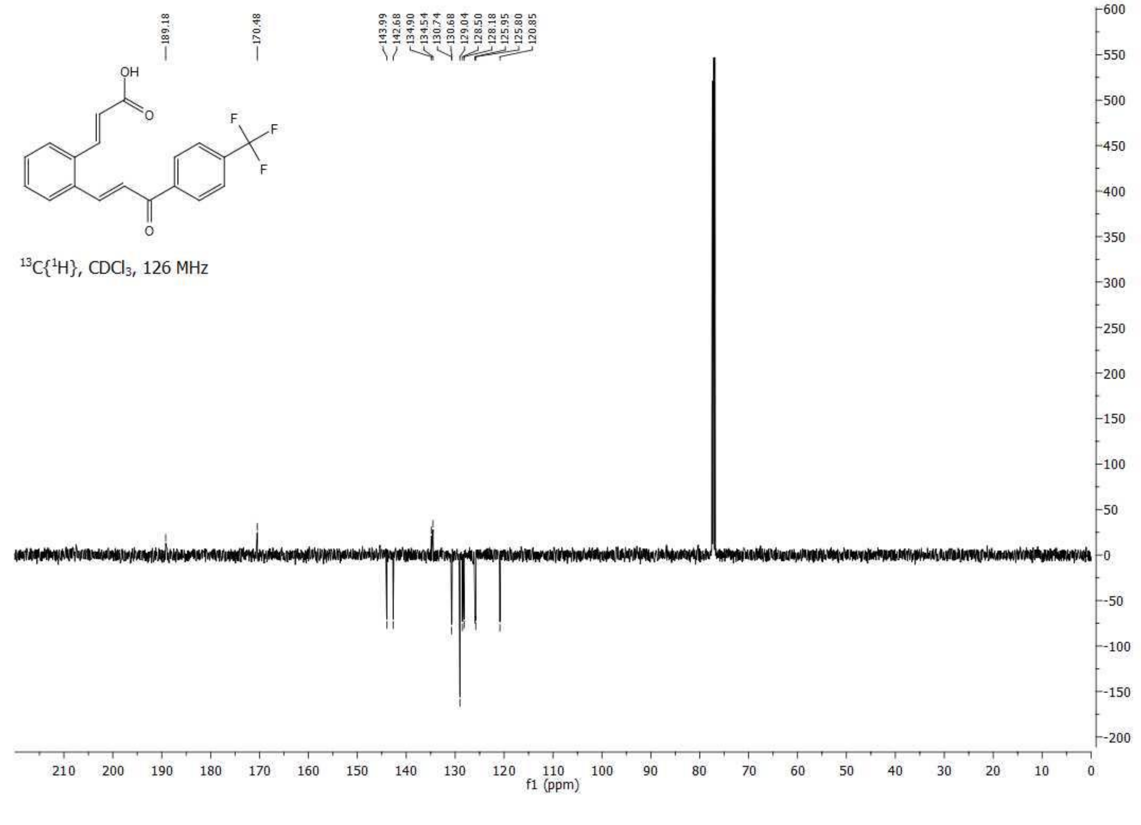

(*E*)-3-(2-((*E*)-3-(4-Methoxyphenyl)-3-oxoprop-1-en-1-yl)phenyl)acrylic acid (S24)

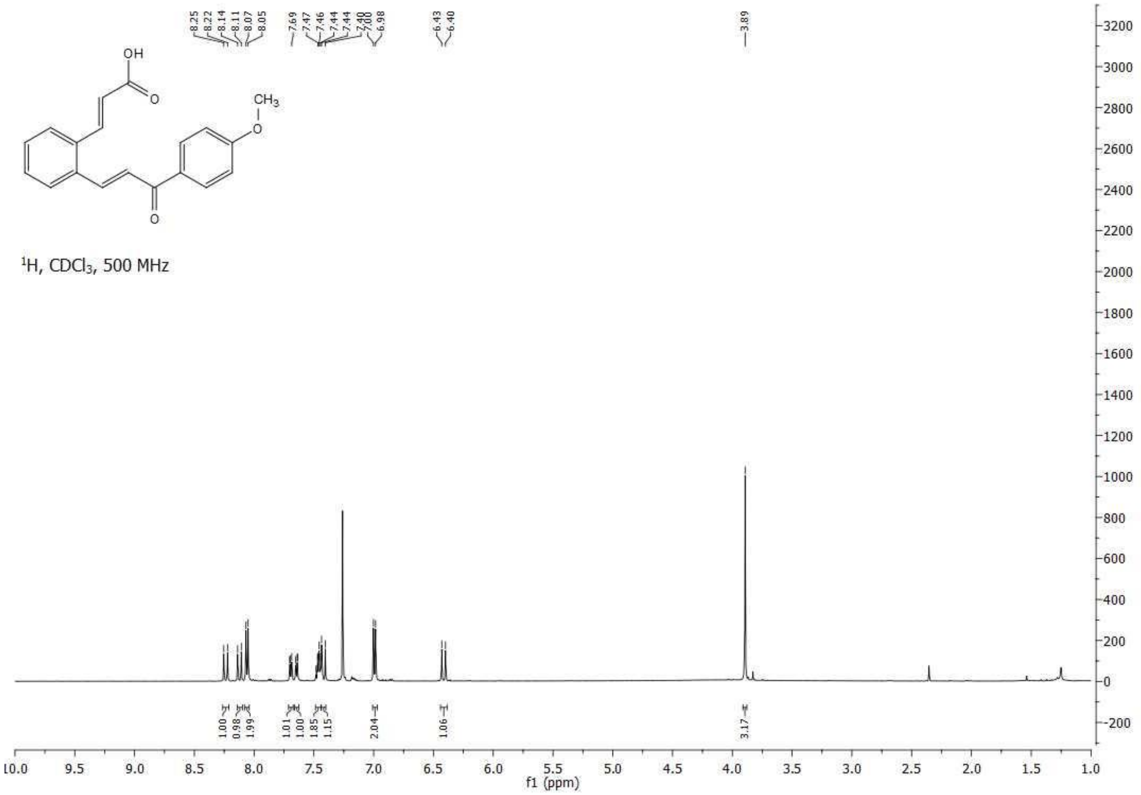

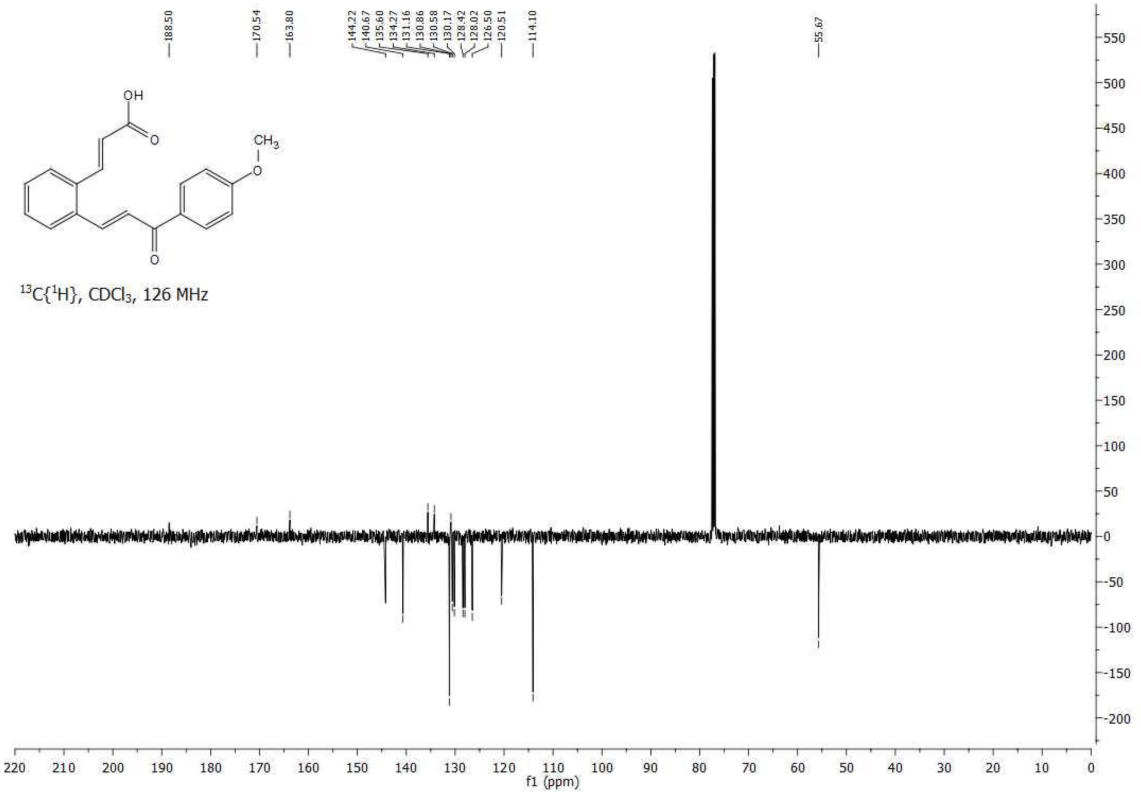

(*E*)-3-(4-Chloro-2-((*E*)-3-oxobut-1-en-1-yl)phenyl)acrylic (S25)

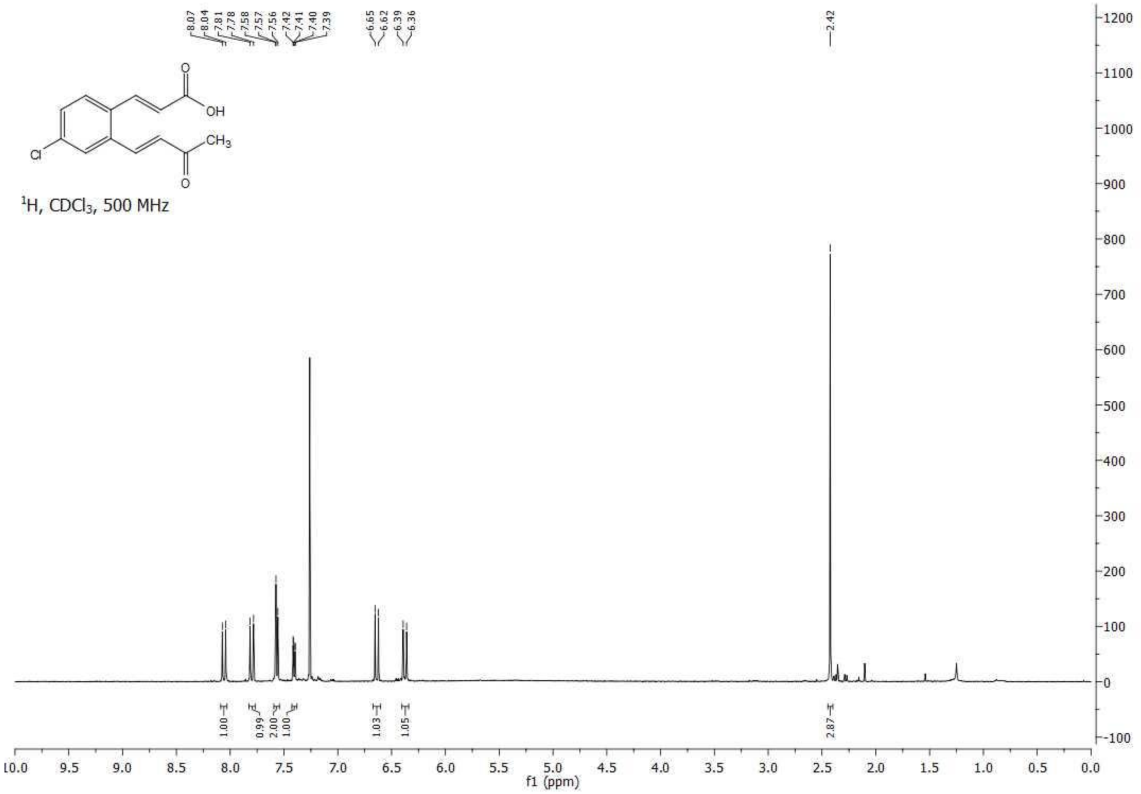

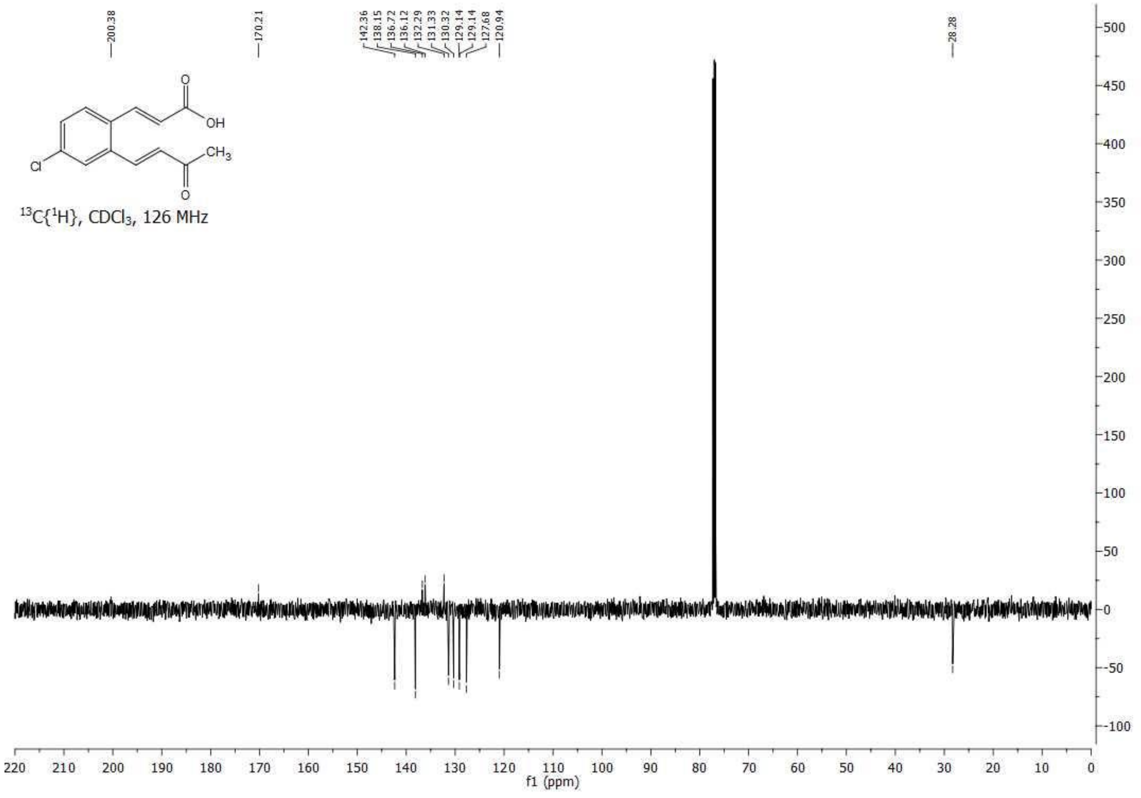

(*E*)-3-(5-Methyl-2-((*E*)-3-oxobut-1-en-1-yl)phenyl)acrylic acid (S26)

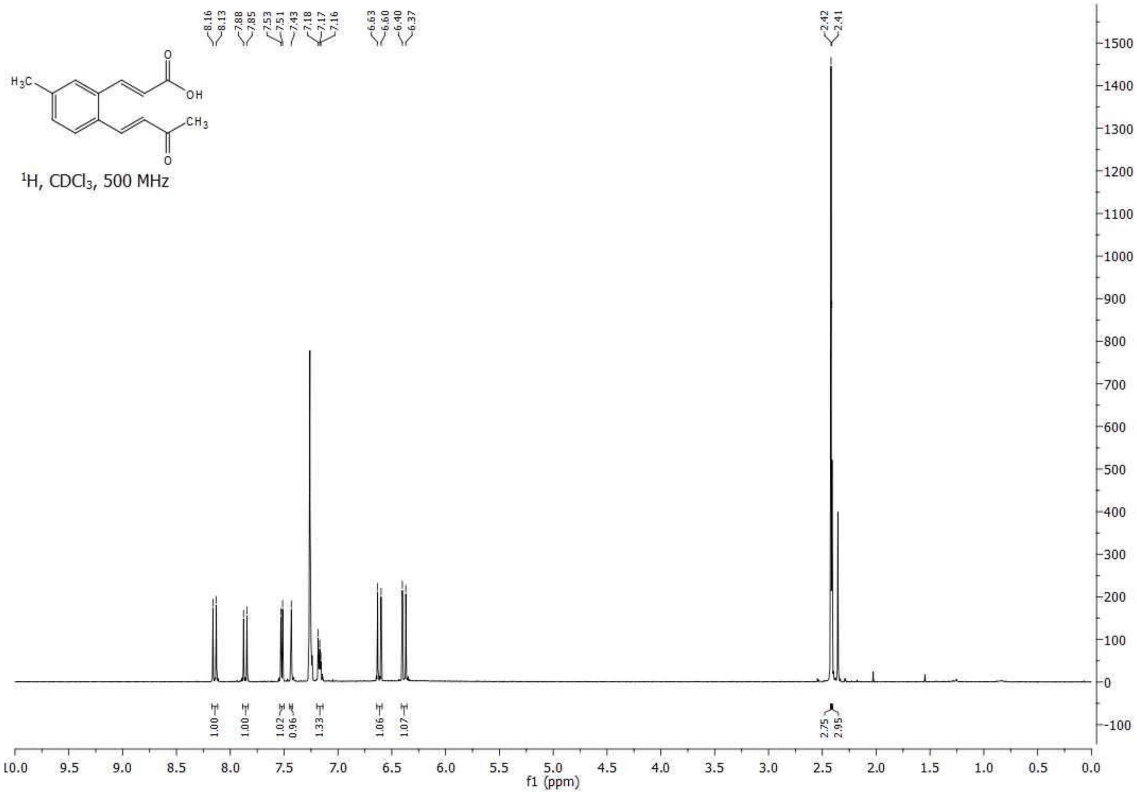

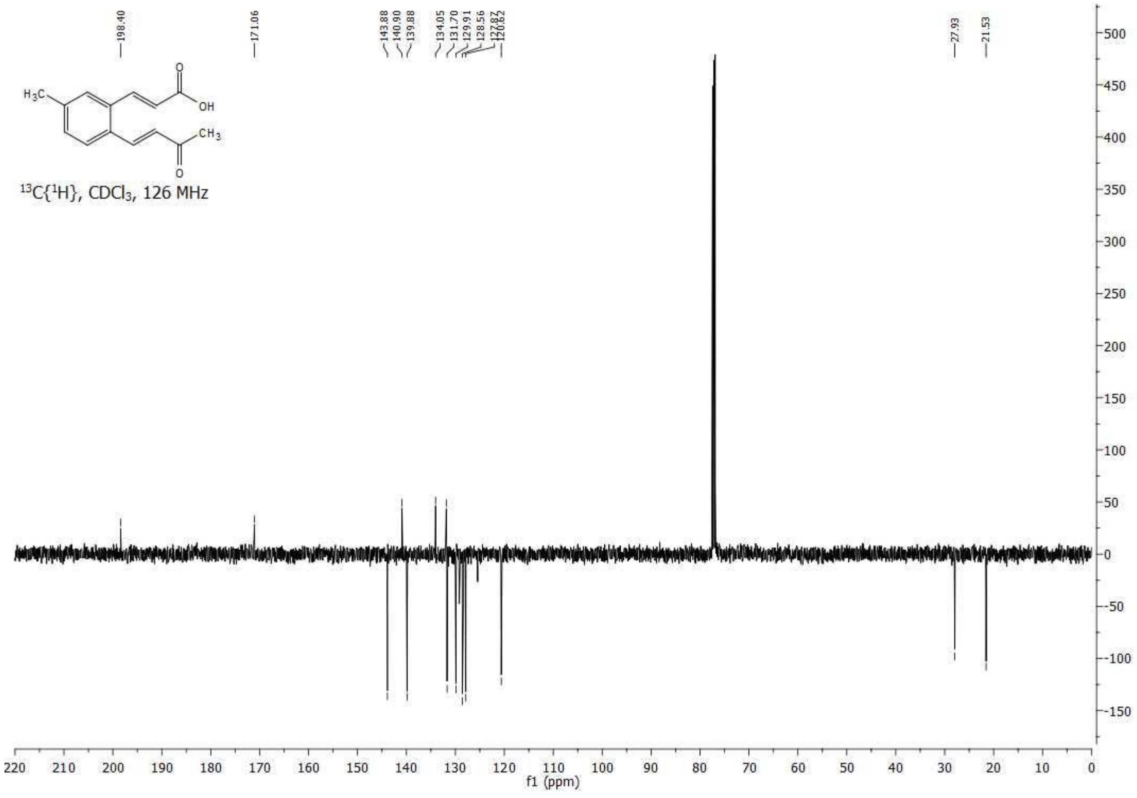

(*E*)-3-(4-Fluoro-2-((*E*)-3-oxobut-1-en-1-yl)phenyl)acrylic (S27)

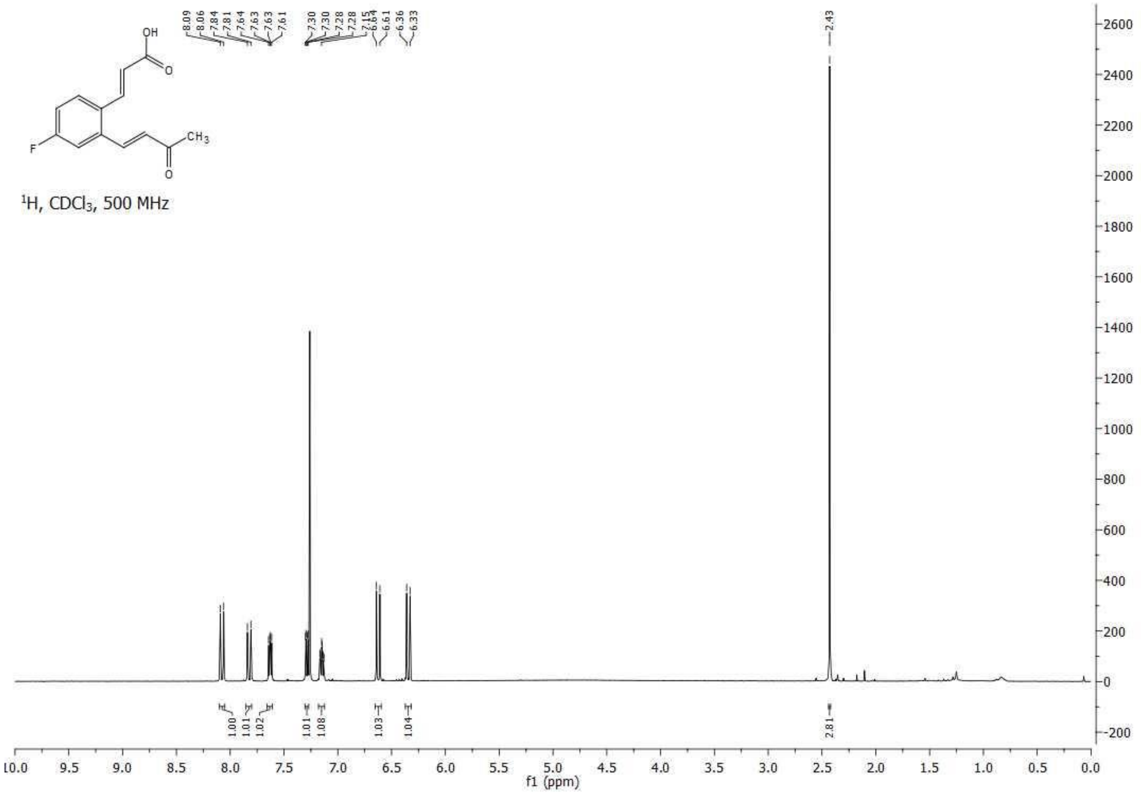

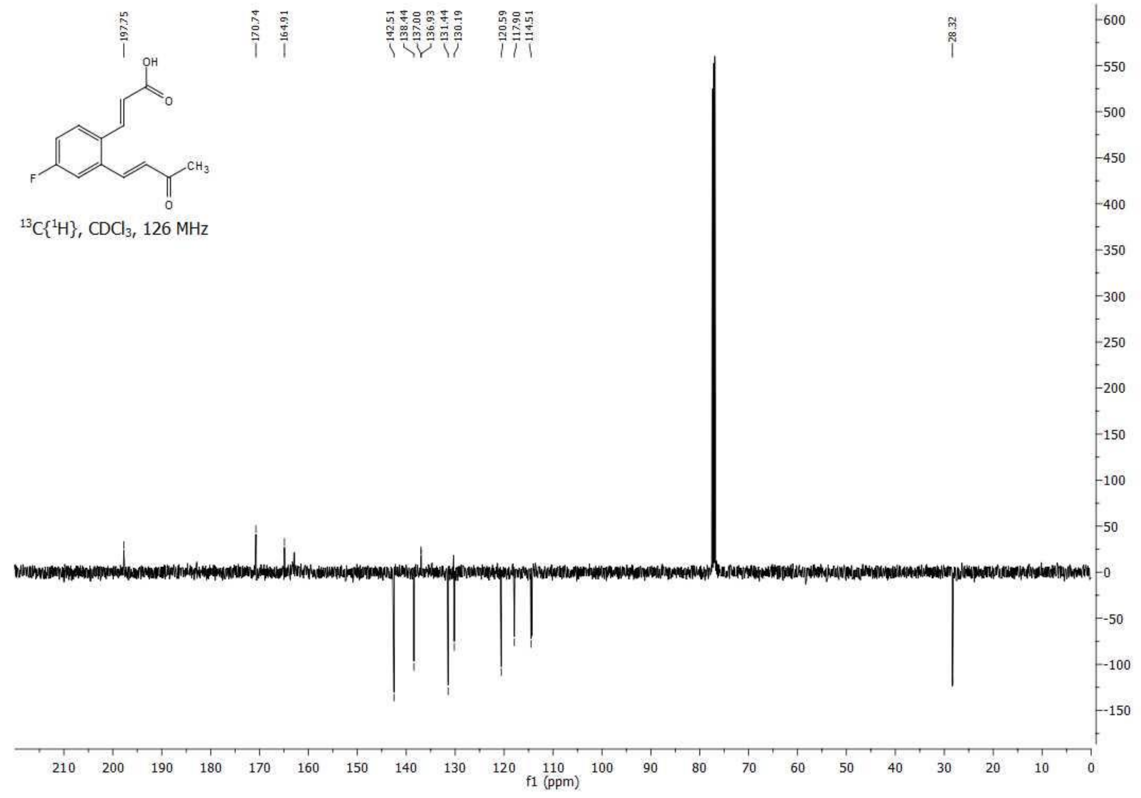

2,4,6-Trichlorophenyl (*E*)-3-(2-((*E*)-3-oxobut-1-en-1-yl)phenyl)acrylate (5)

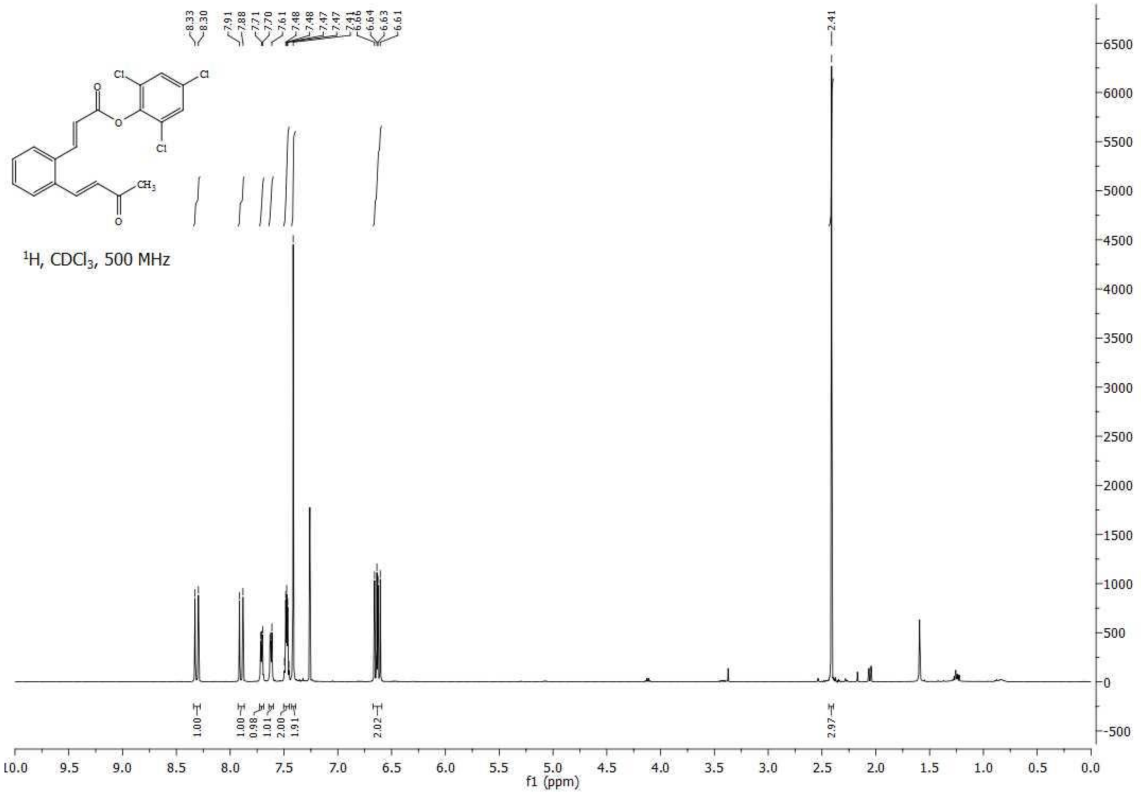

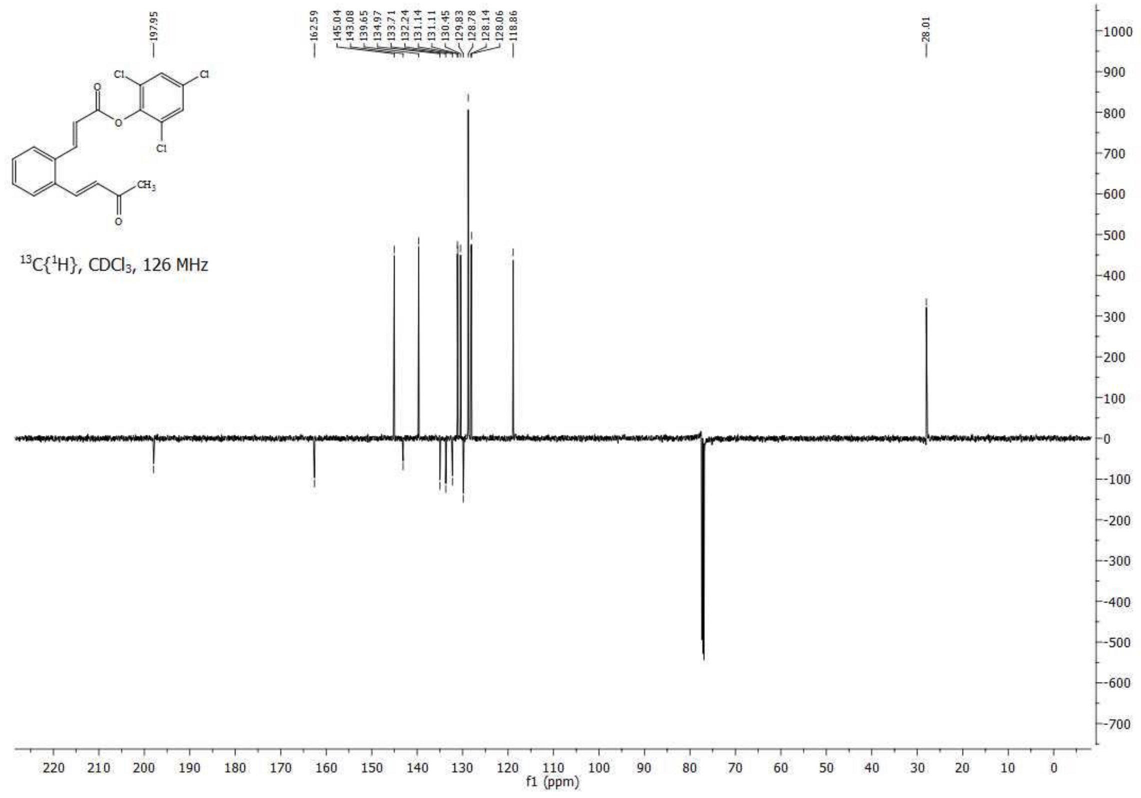

2,4,6-Trichlorophenyl (*E*)-3-(2-((*E*)-4,4-dimethyl-3-oxopent-1-en-1-yl)phenyl)acrylate (S28)

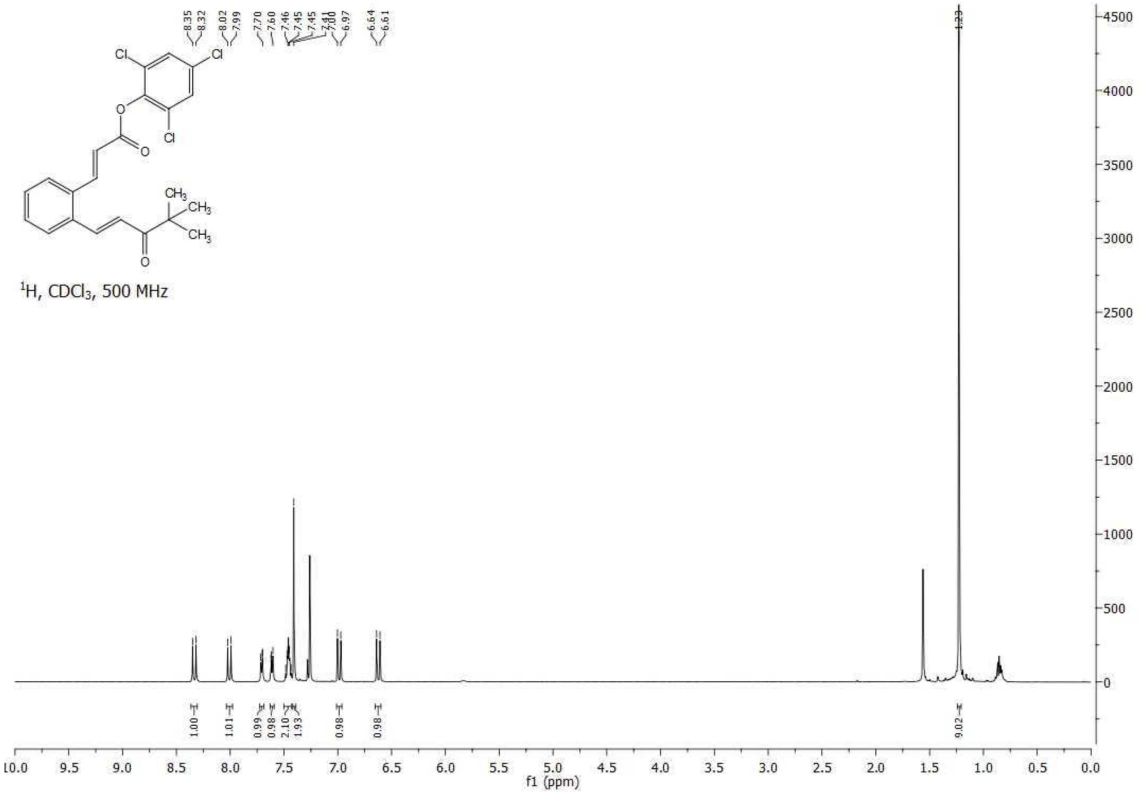

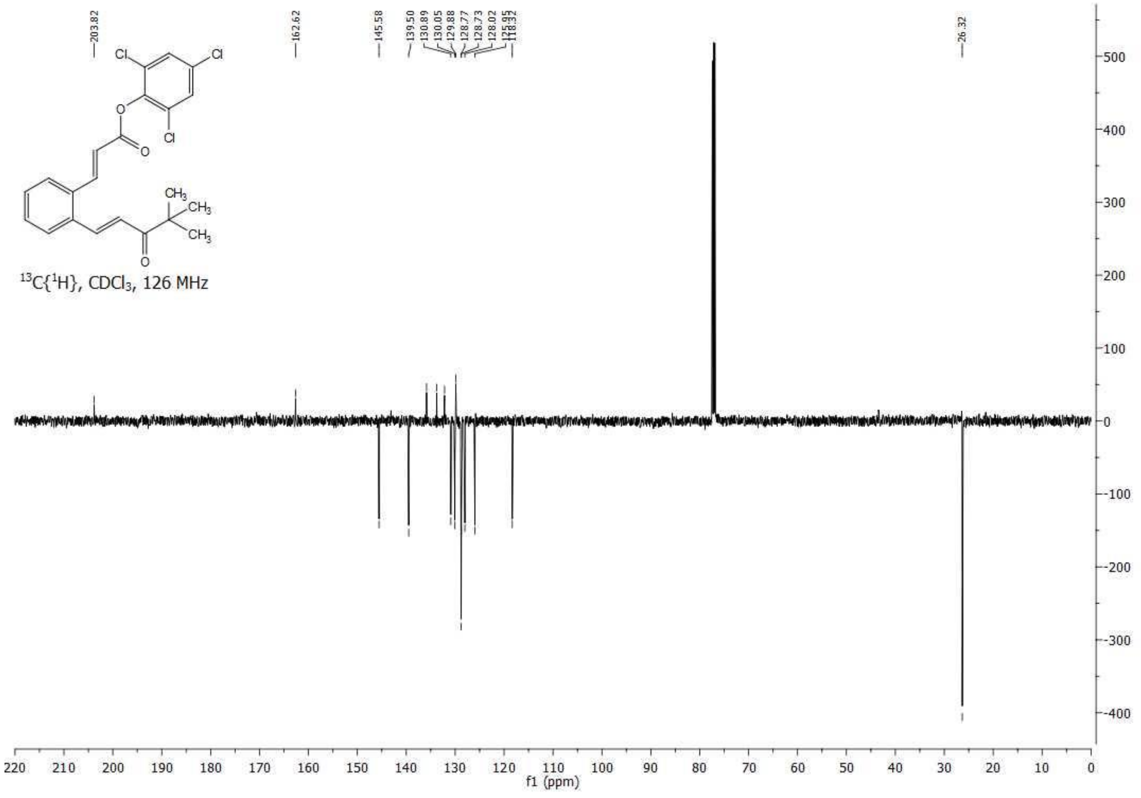

(*E*)-2,4,6-Trichlorophenyl 3-(2-((*E*)-3-oxo-3-phenylprop-1-en-1-yl)phenyl)acrylate (S29)

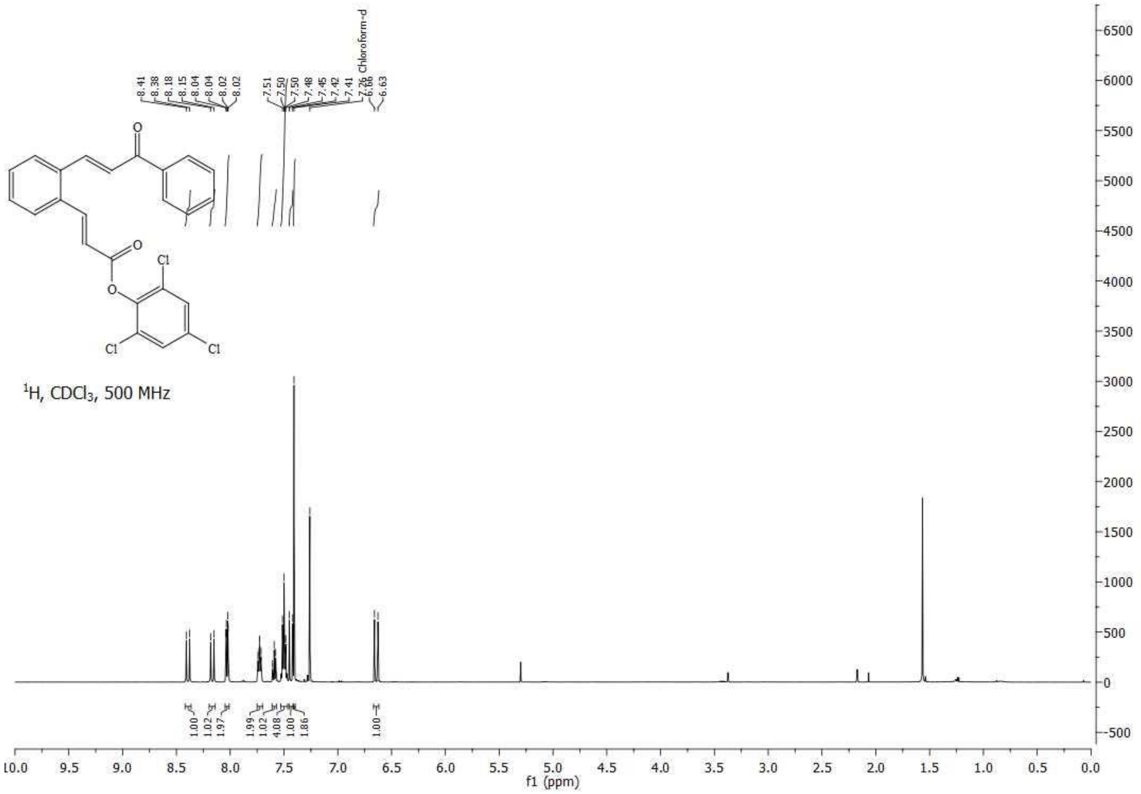

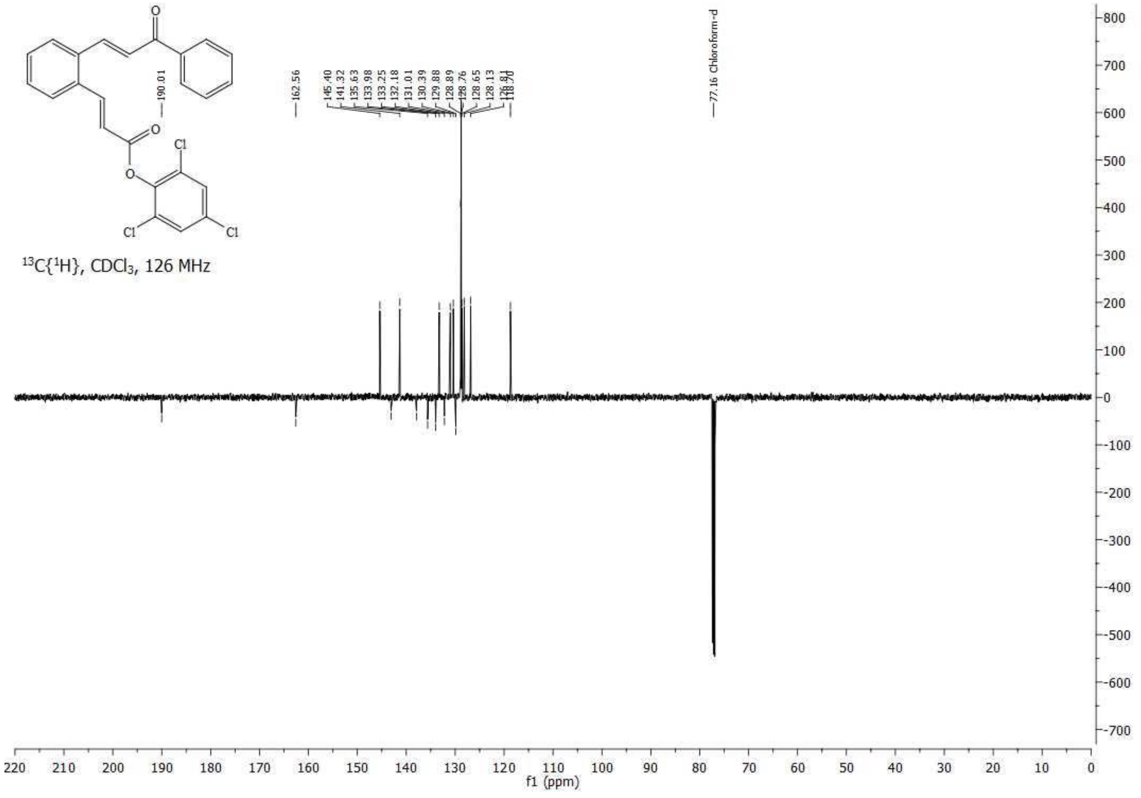

2,4,6-Trichlorophenyl (*E*)-3-(2-((*E*)-3-(4-bromophenyl)-3-oxoprop-1-en-1-yl)phenyl)acrylate (S30)

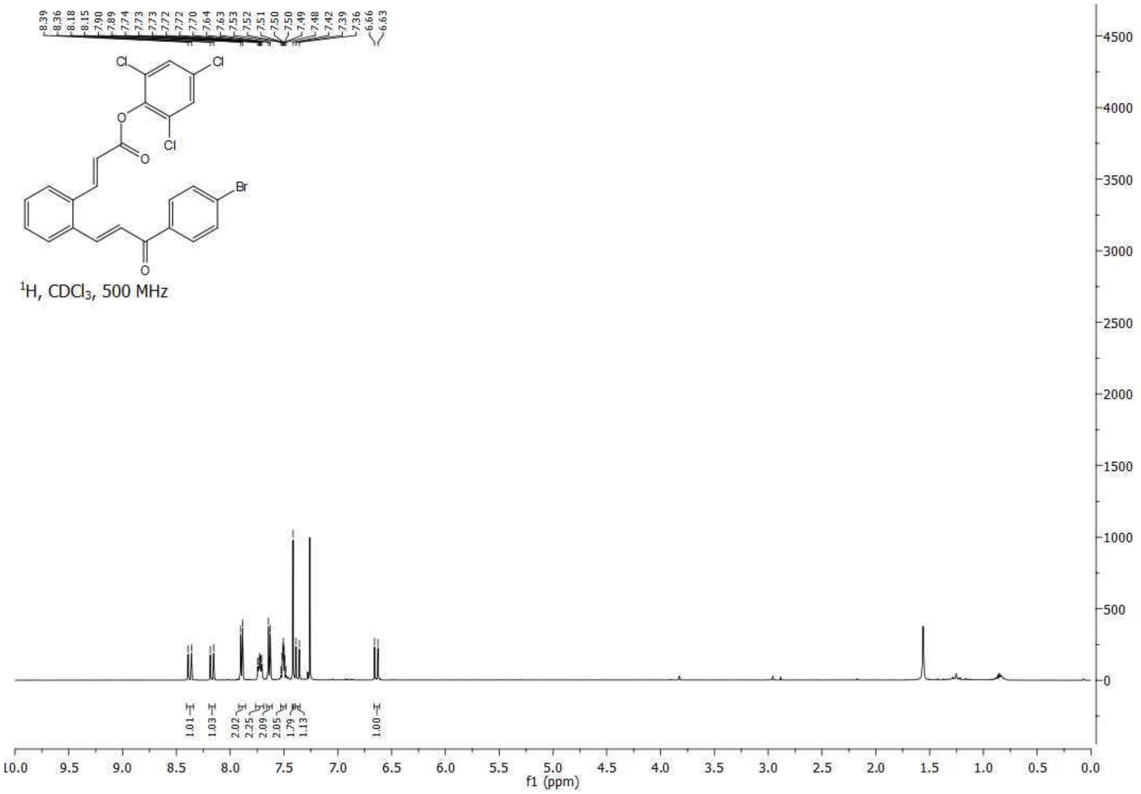

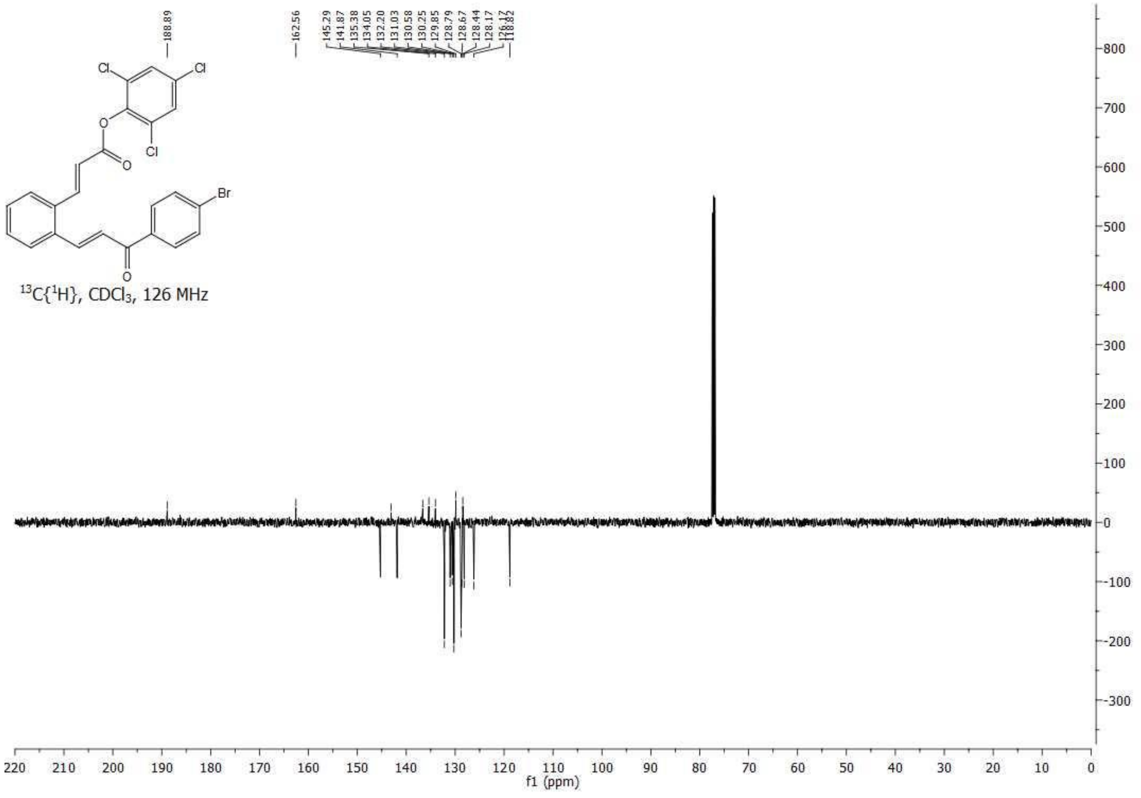

2,4,6-Trichlorophenyl (*E*)-3-(2-((*E*)-3-(4-chlorophenyl)-3-oxoprop-1-en-1-yl)phenyl)acrylate (S31)

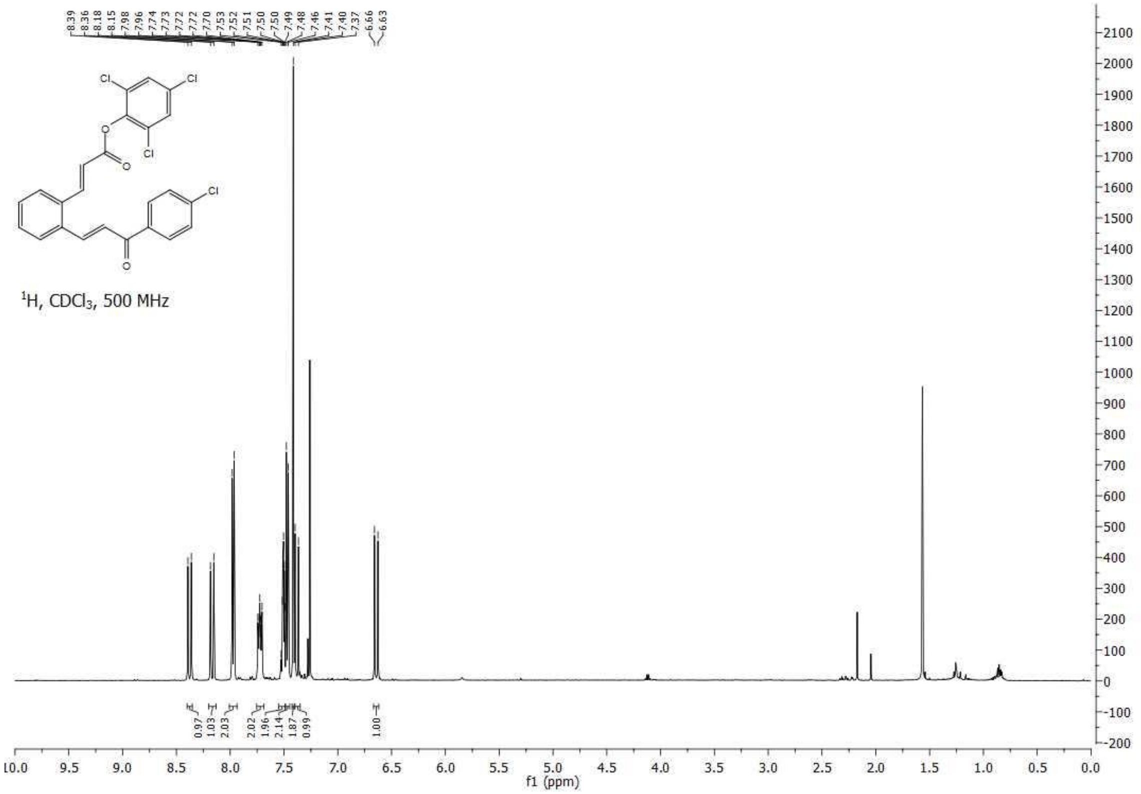

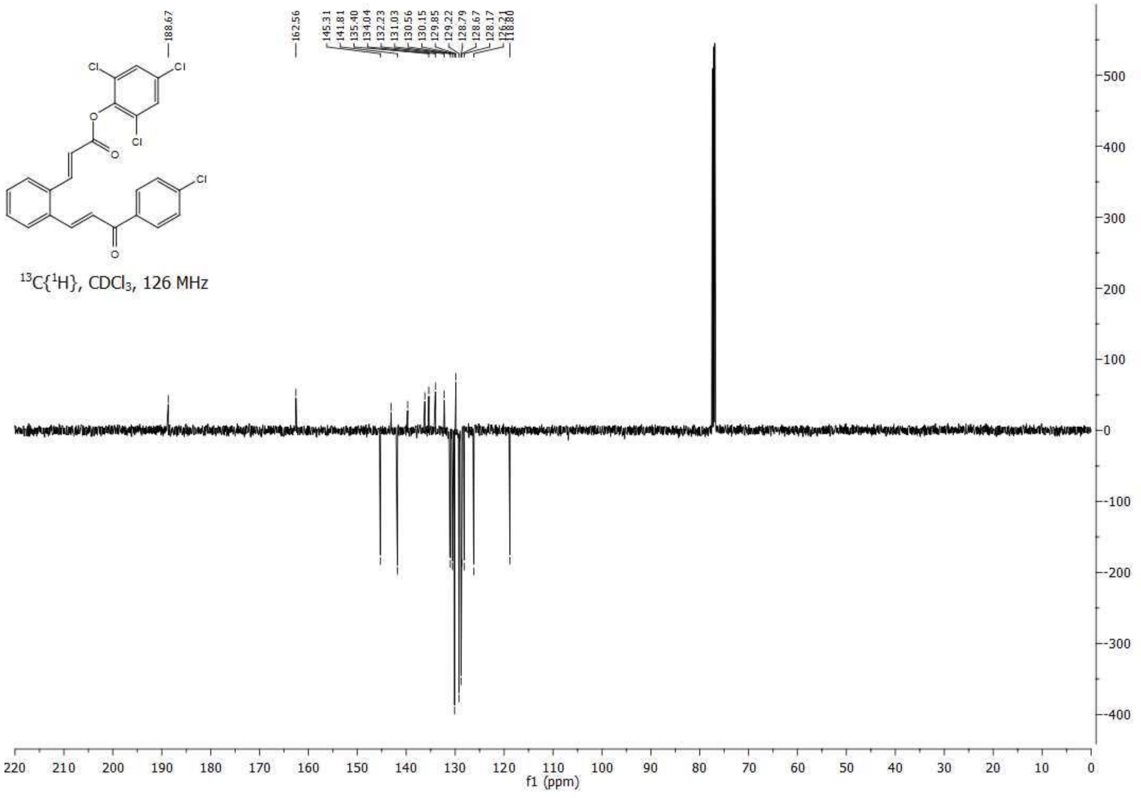

2,4,6-Trichlorophenyl (*E*)-3-(2-((*E*)-3-oxo-3-(4-(trifluoromethyl)phenyl)prop-1-en-1-yl)phenyl)-acrylate (S32)

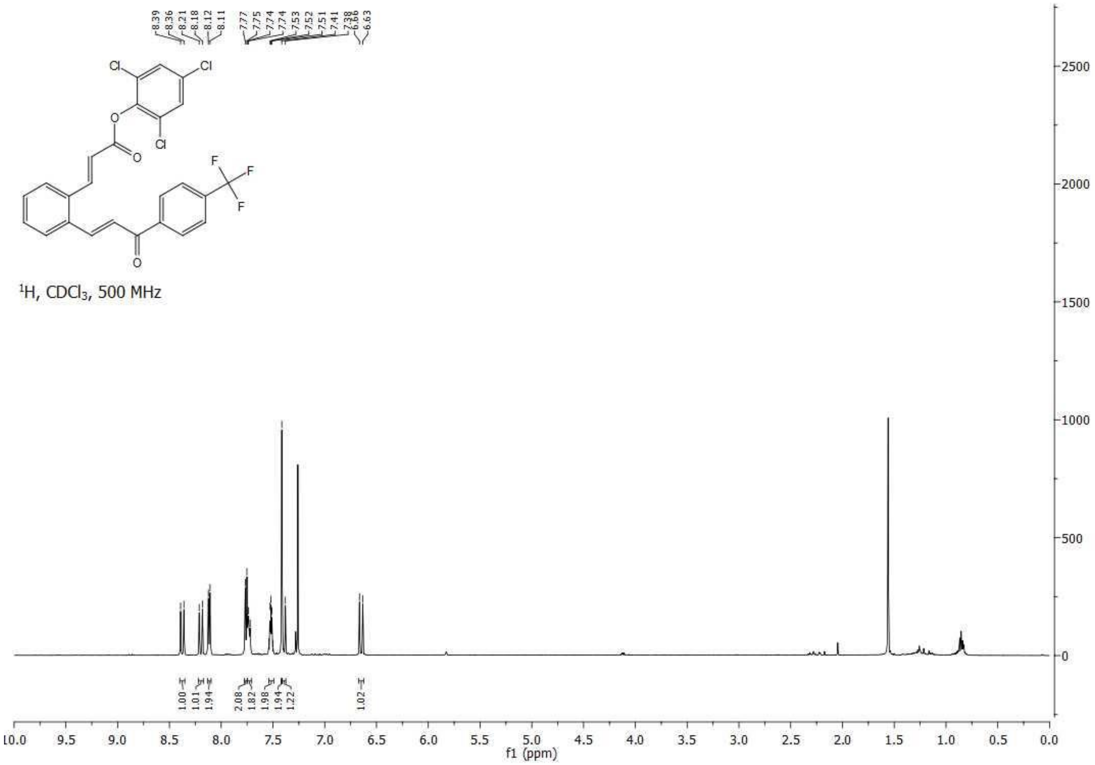

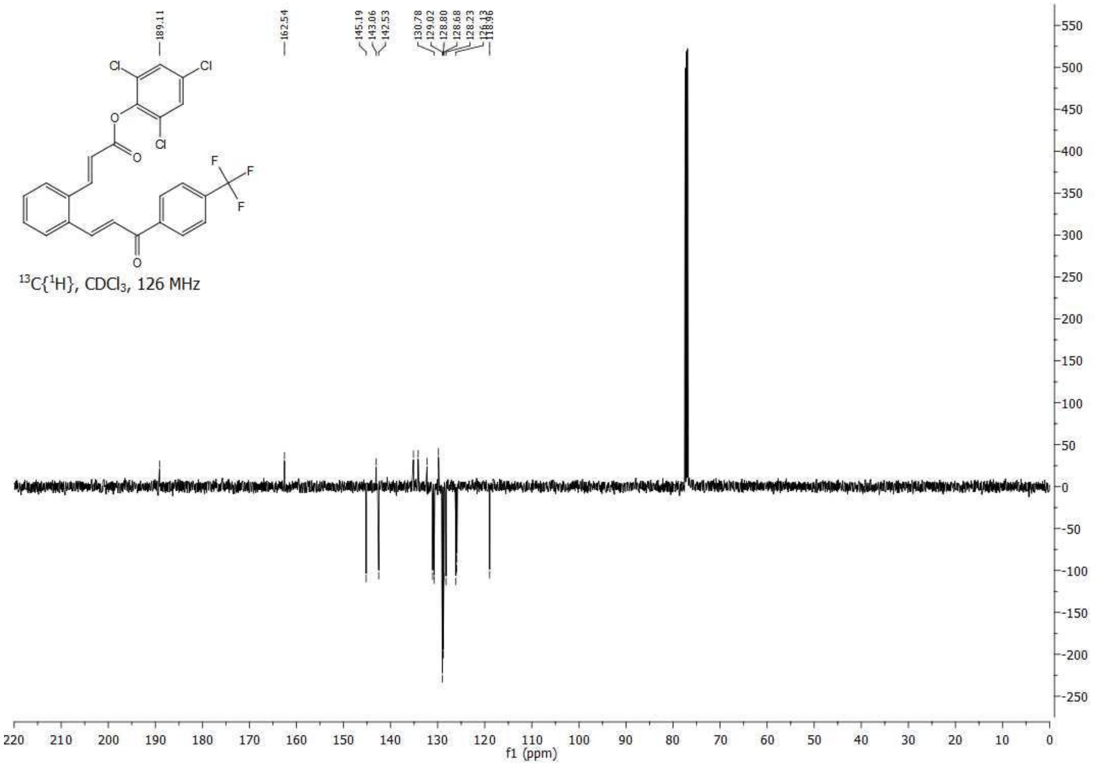

2,4,6-Trichlorophenyl (*E*)-3-(2-((*E*)-3-(4-methoxyphenyl)-3-oxoprop-1-en-1-yl)phenyl)acrylate (S33)

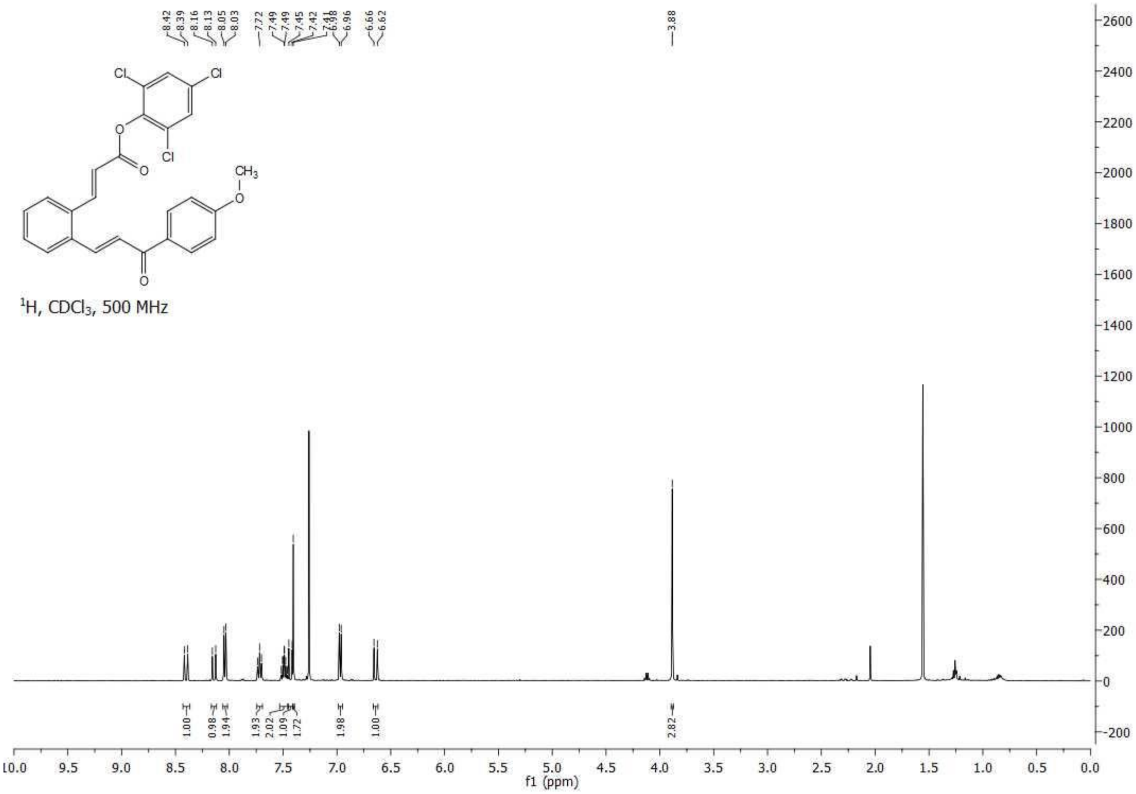

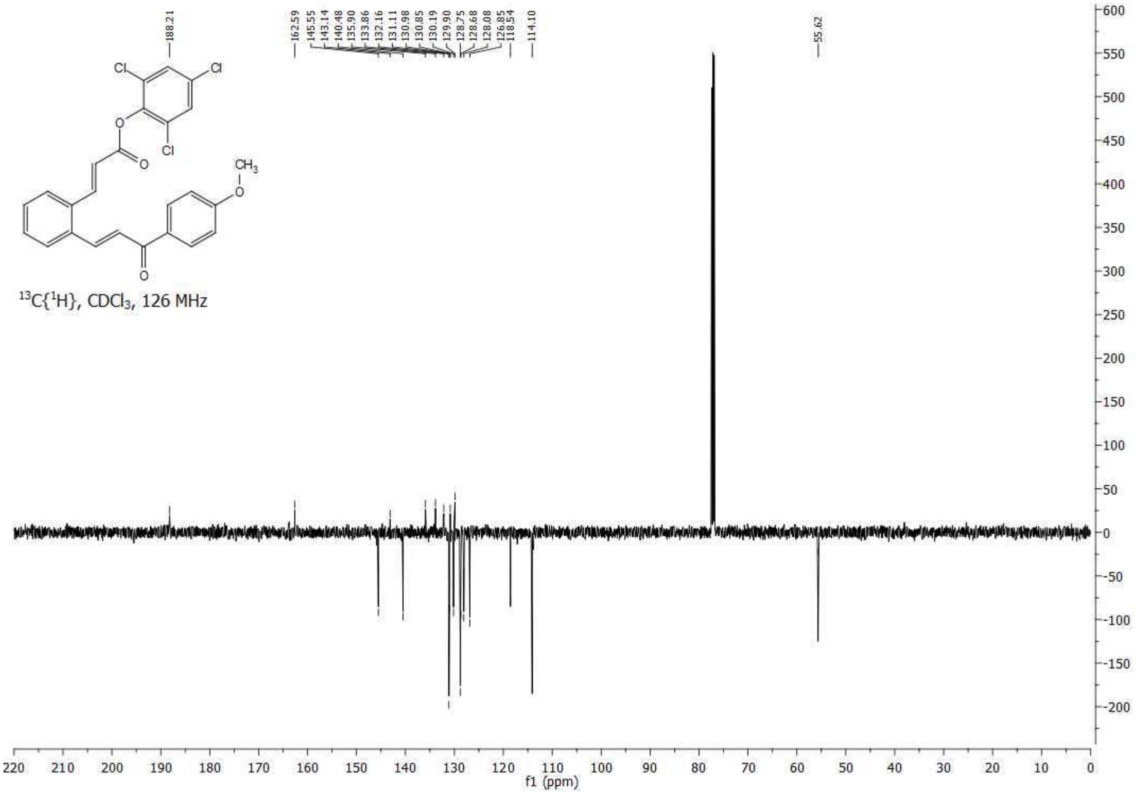

2,4,6-Trichlorophenyl (*E*)-3-(4-chloro-2-((*E*)-3-oxobut-1-en-1-yl)phenyl)acrylate (S34)

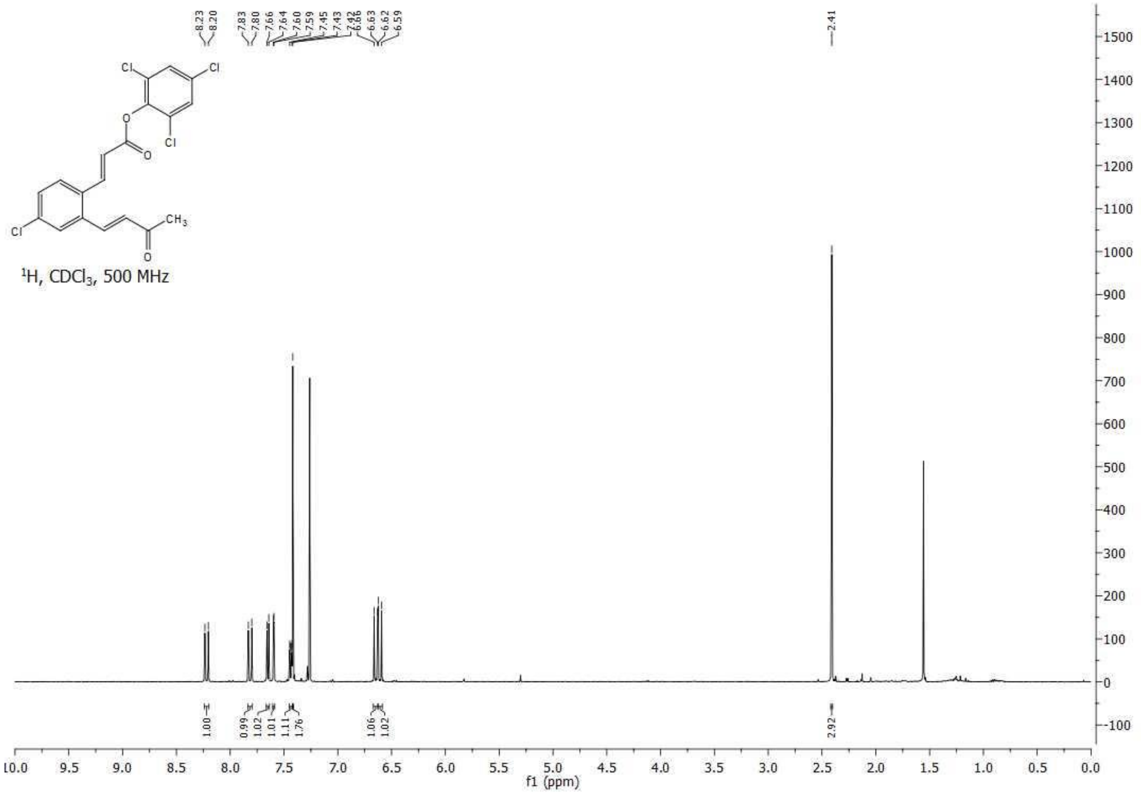

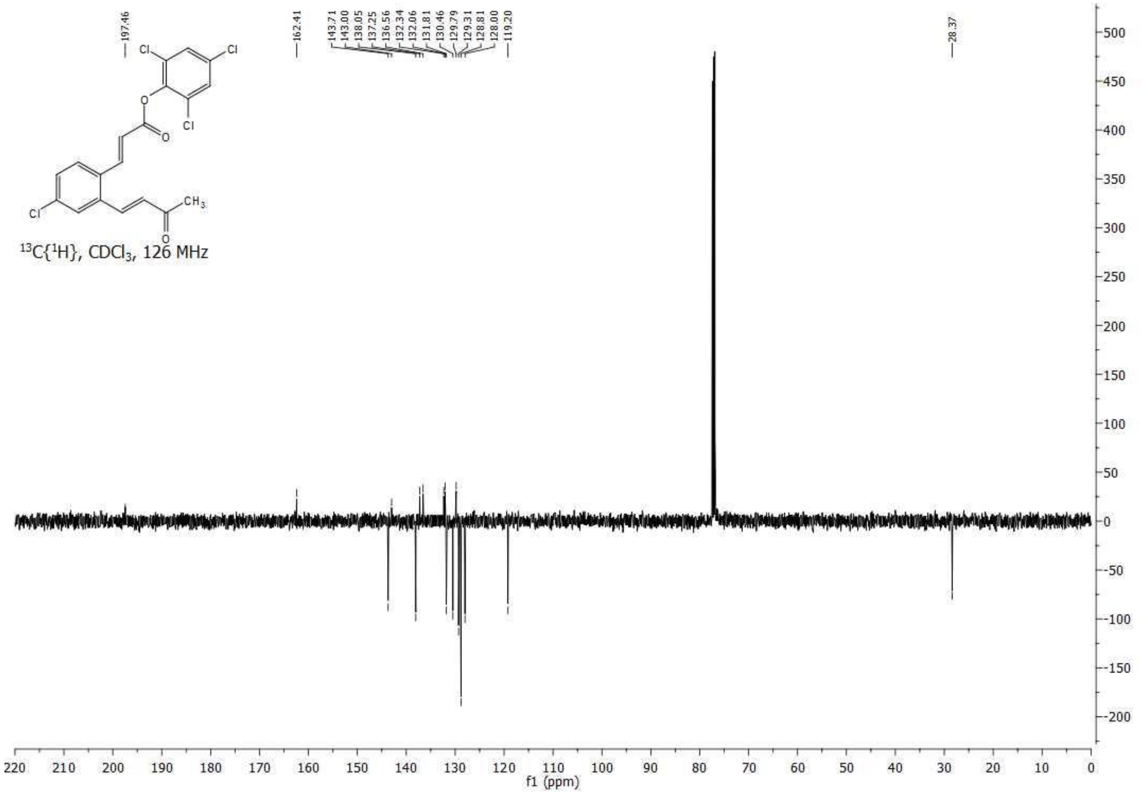

2,4,6-Trichlorophenyl (*E*)-3-(5-methyl-2-((*E*)-3-oxobut-1-en-1-yl)phenyl)acrylate (S35)

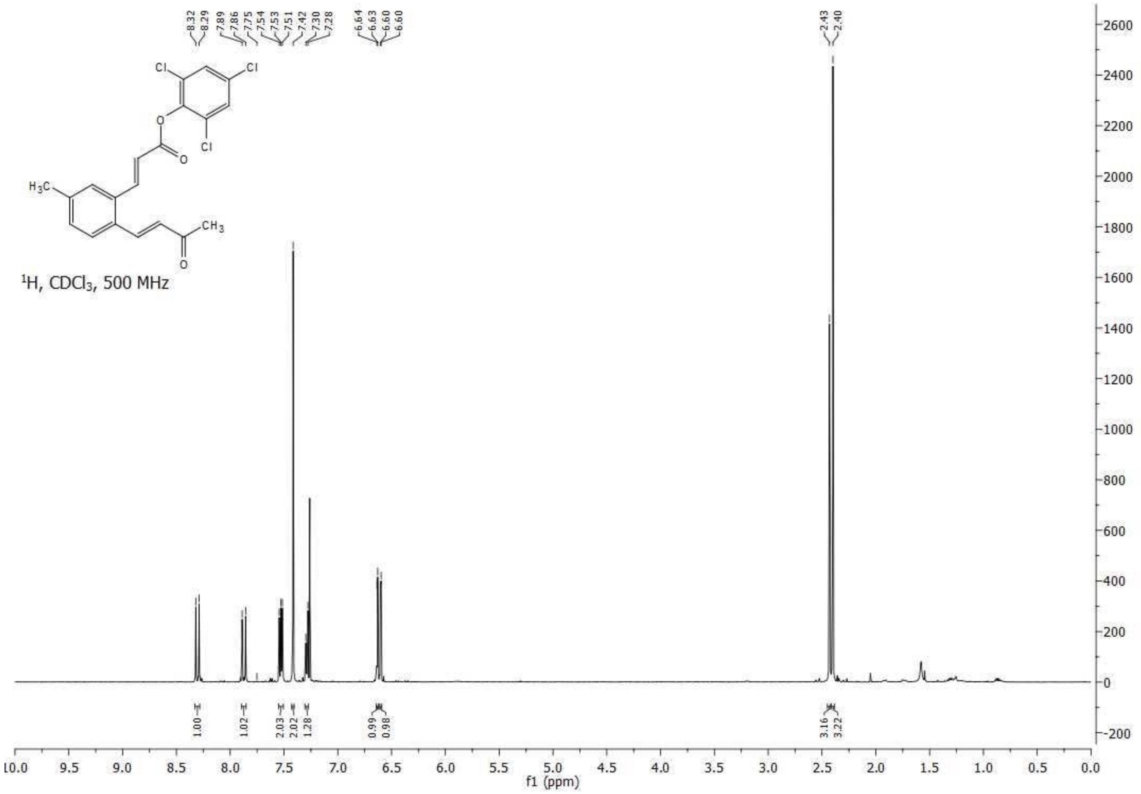

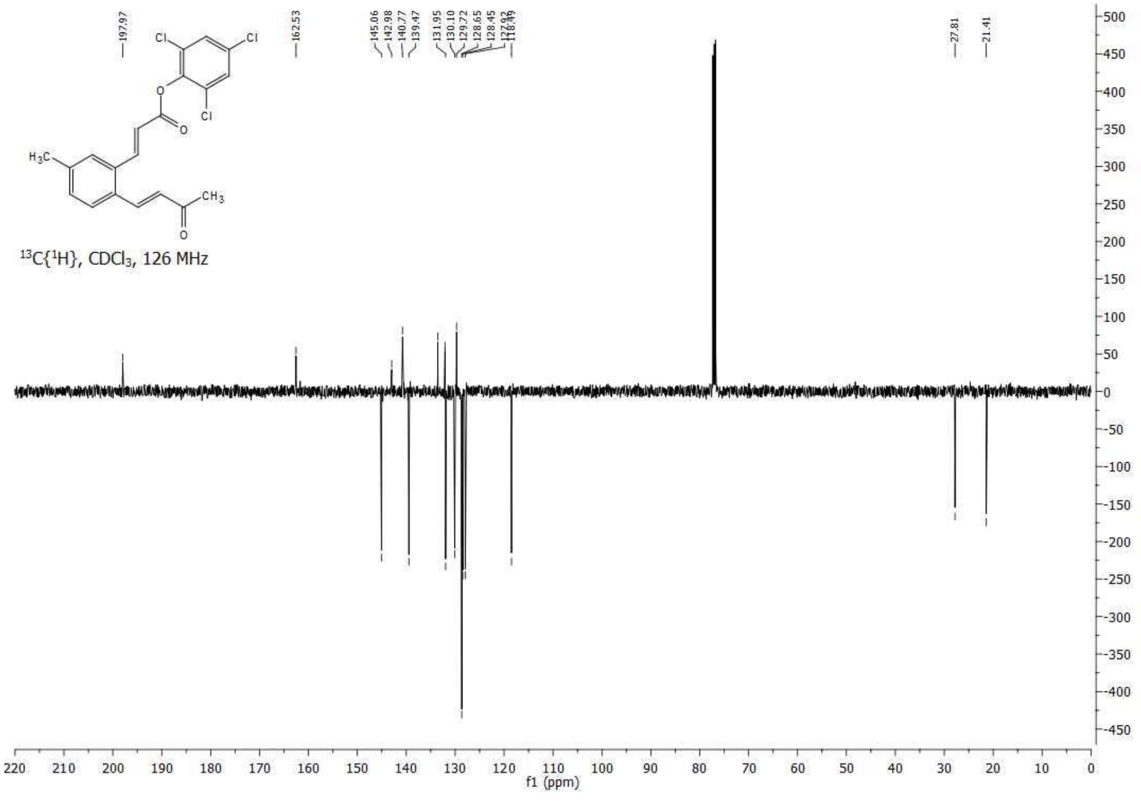

2,4,6-Trichlorophenyl (*E*)-3-(4-fluoro-2-((*E*)-3-oxobut-1-en-1-yl)phenyl)acrylate (S36)

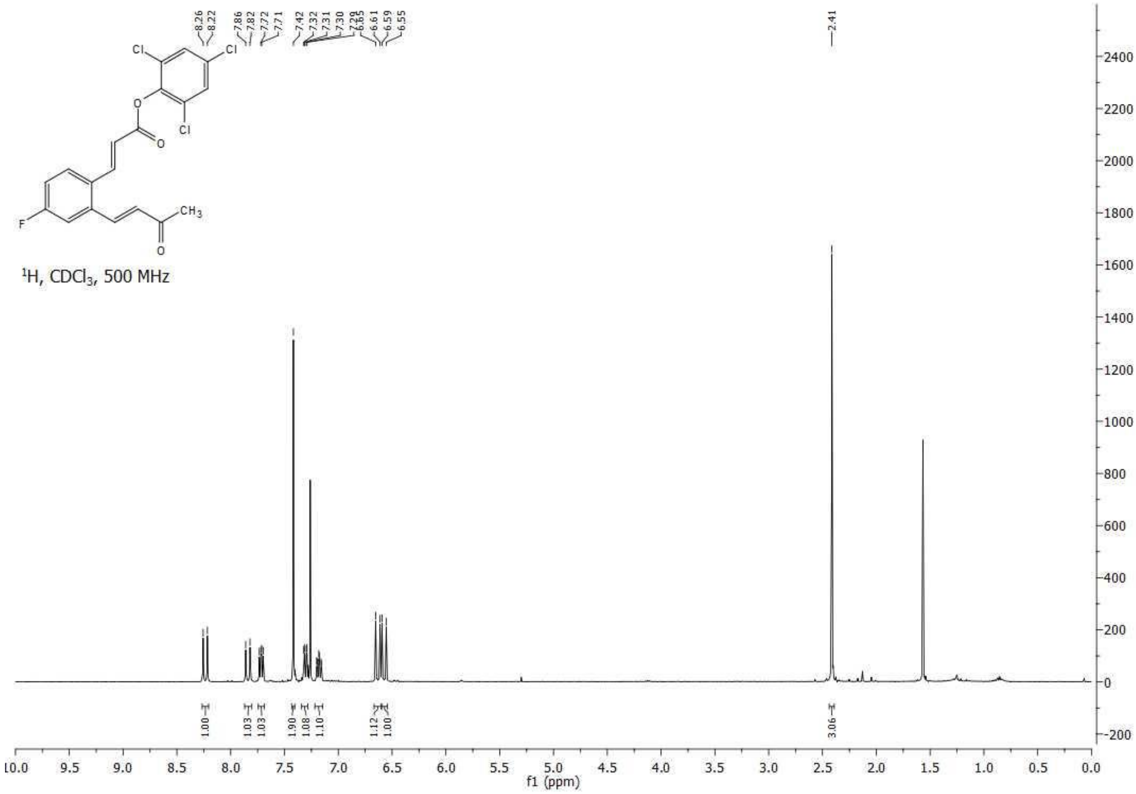

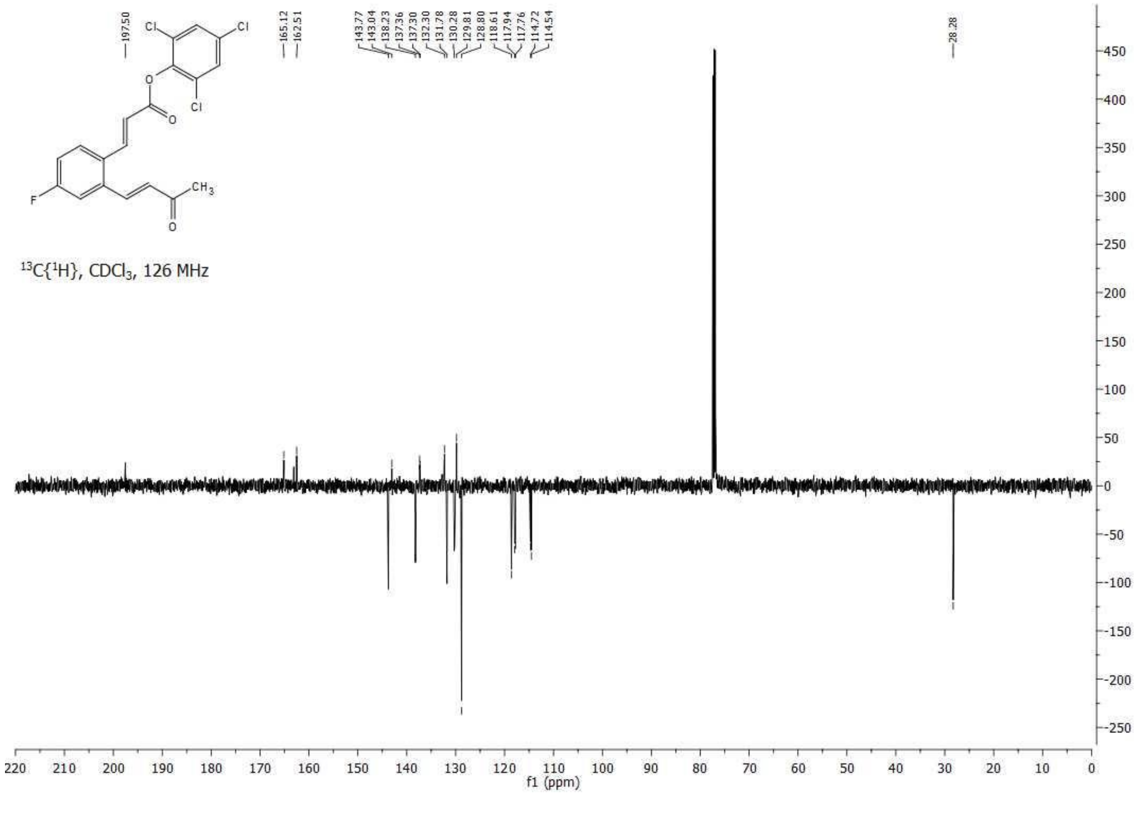

2-(Benzo[d]thiazol-2-yl)-1-(4-fluorophenyl)ethan-1-one (53)

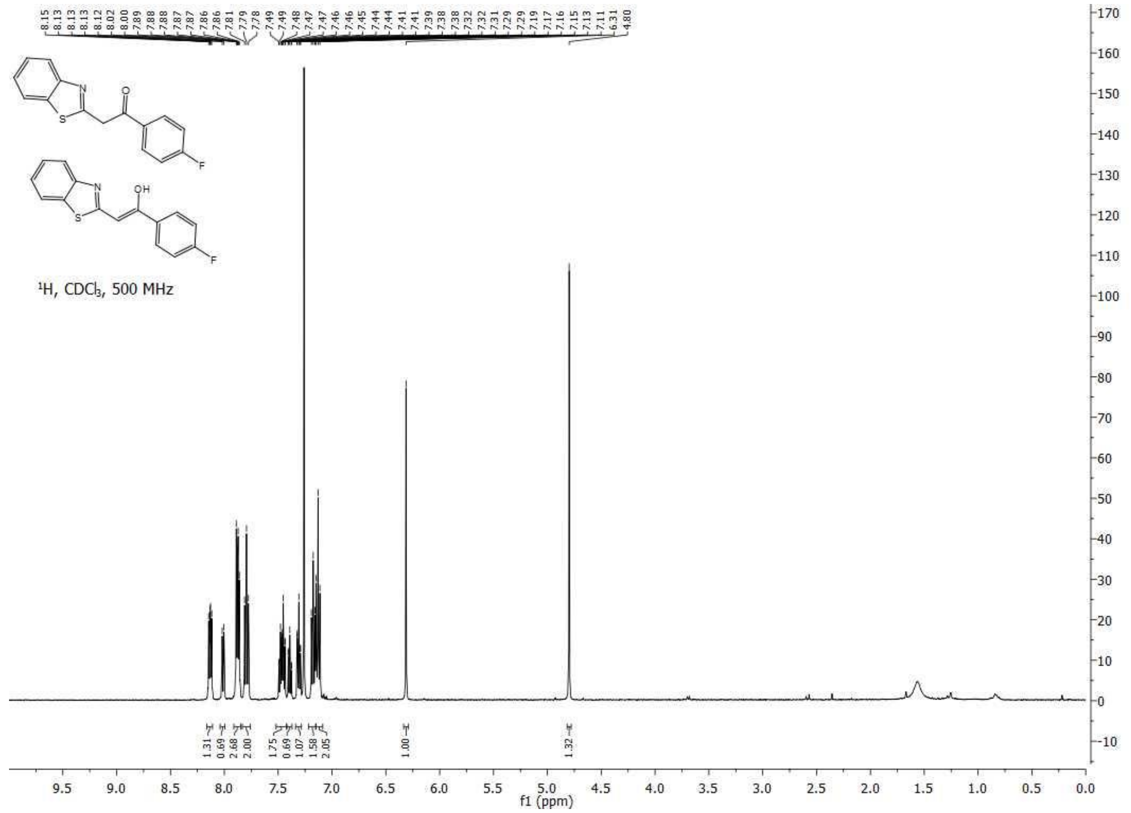

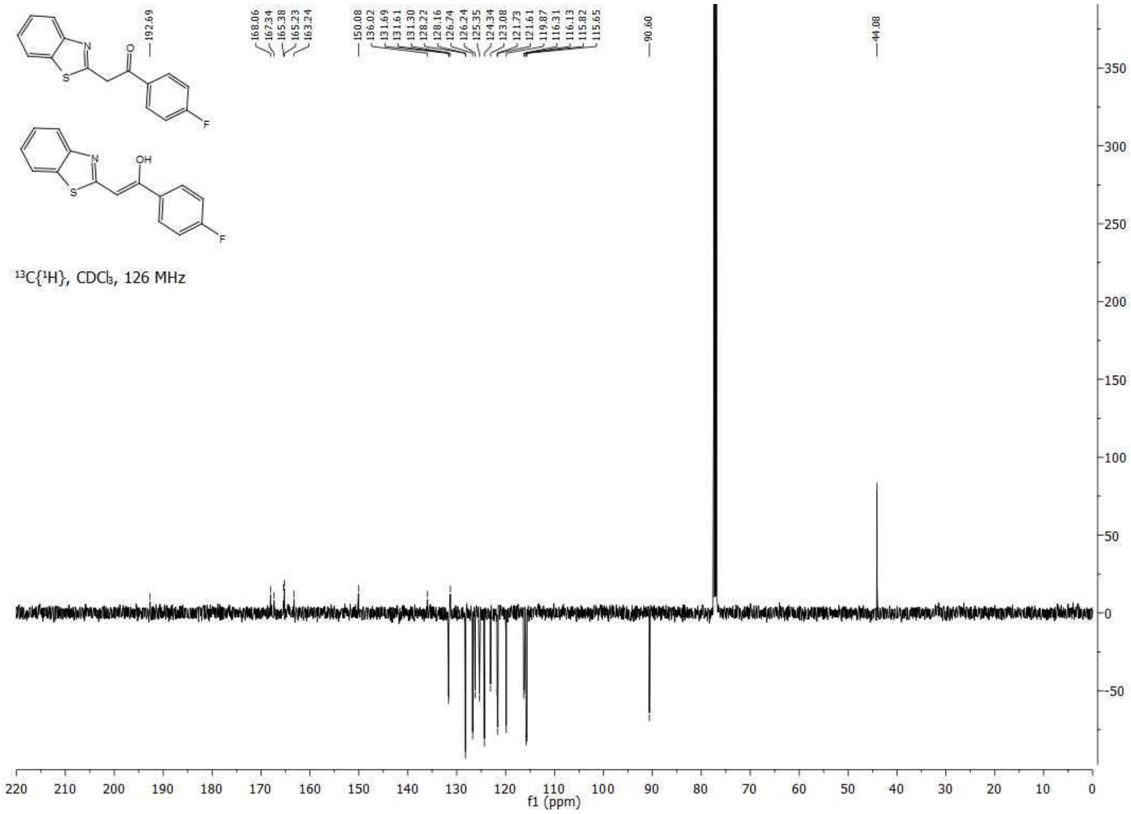

2-(Benzo[d]thiazol-2-yl)-1-(4-bromophenyl)ethan-1-one (S39)

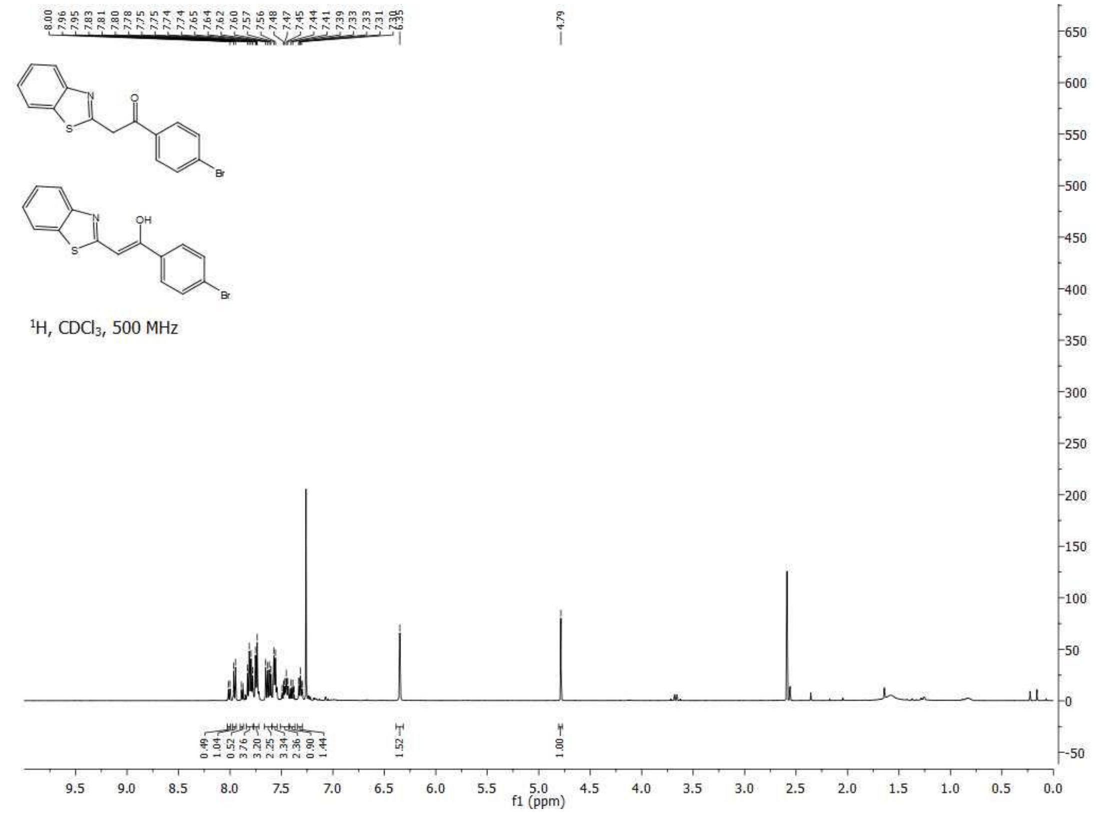

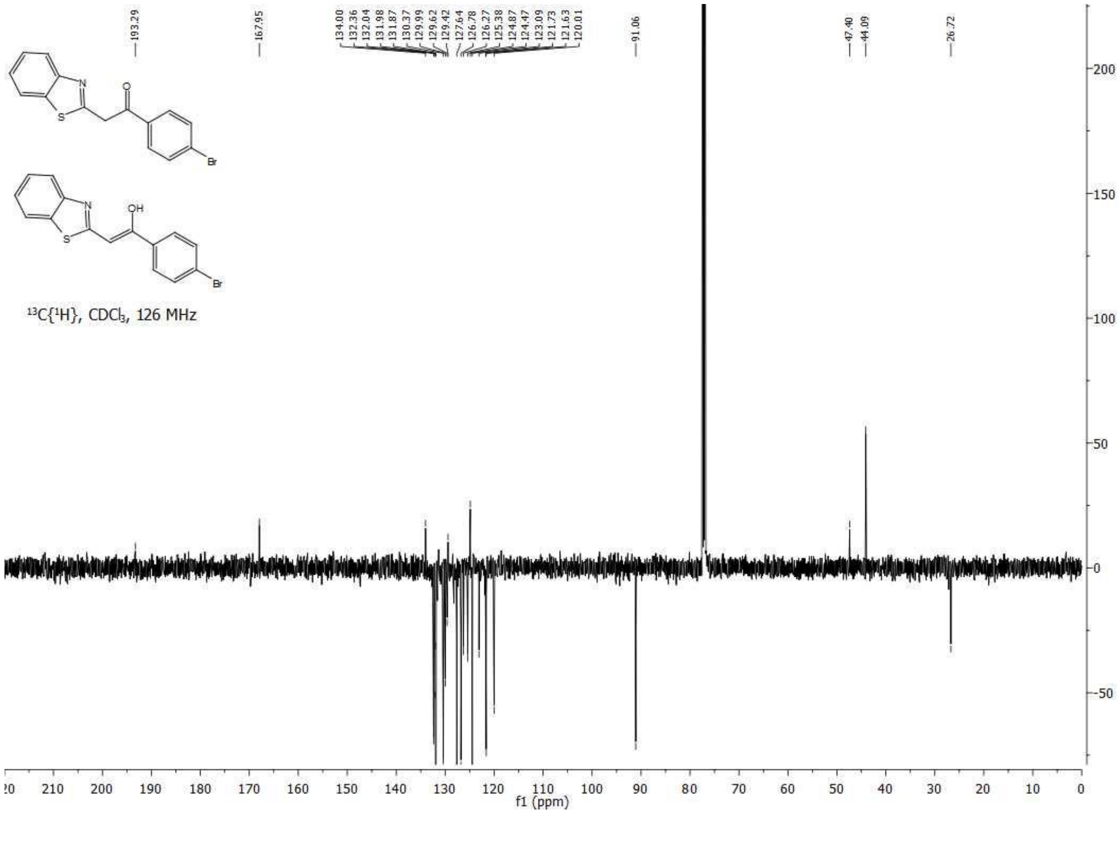

2-(Benzo[d]thiazol-2-yl)-1-(4-methoxyphenyl)ethan-1-one (S40)

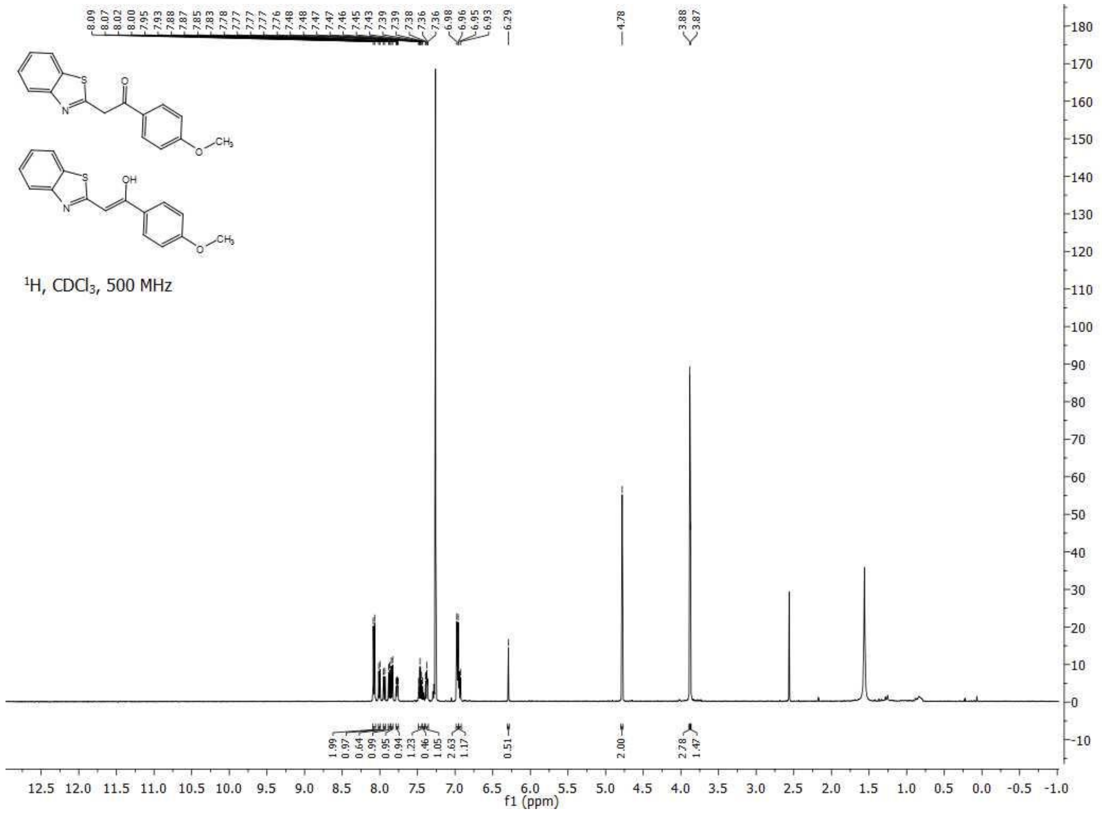

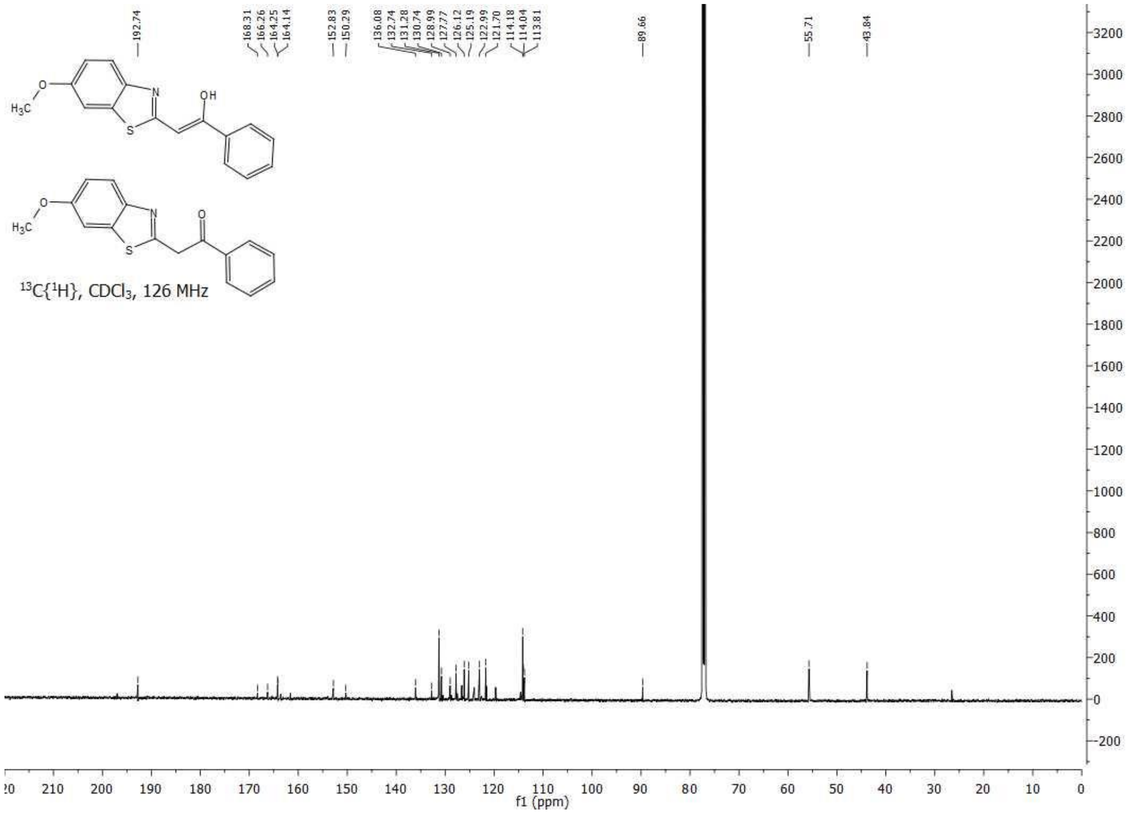

2-(6-Fluorobenzo[d]thiazol-2-yl)-1-phenylethan-1-one (S41)

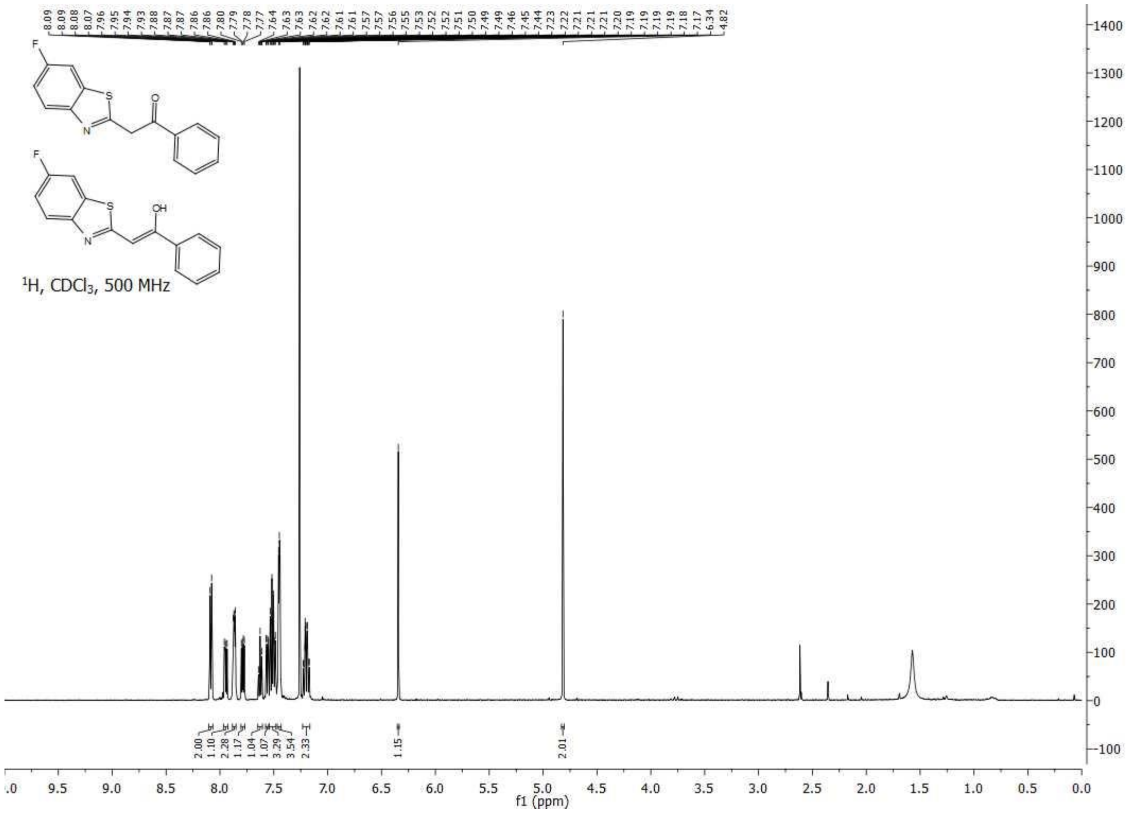

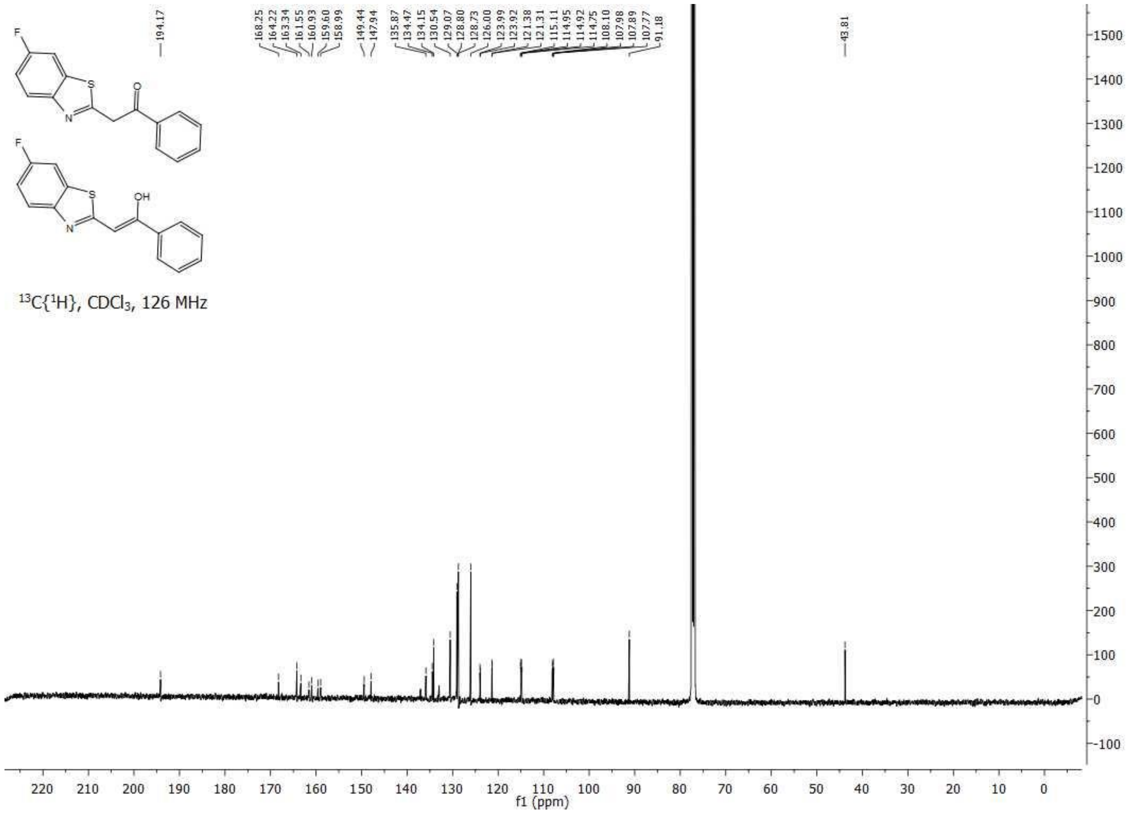

2-(6-Bromobenzo[d]thiazol-2-yl)-1-phenylethan-1-one (S42)

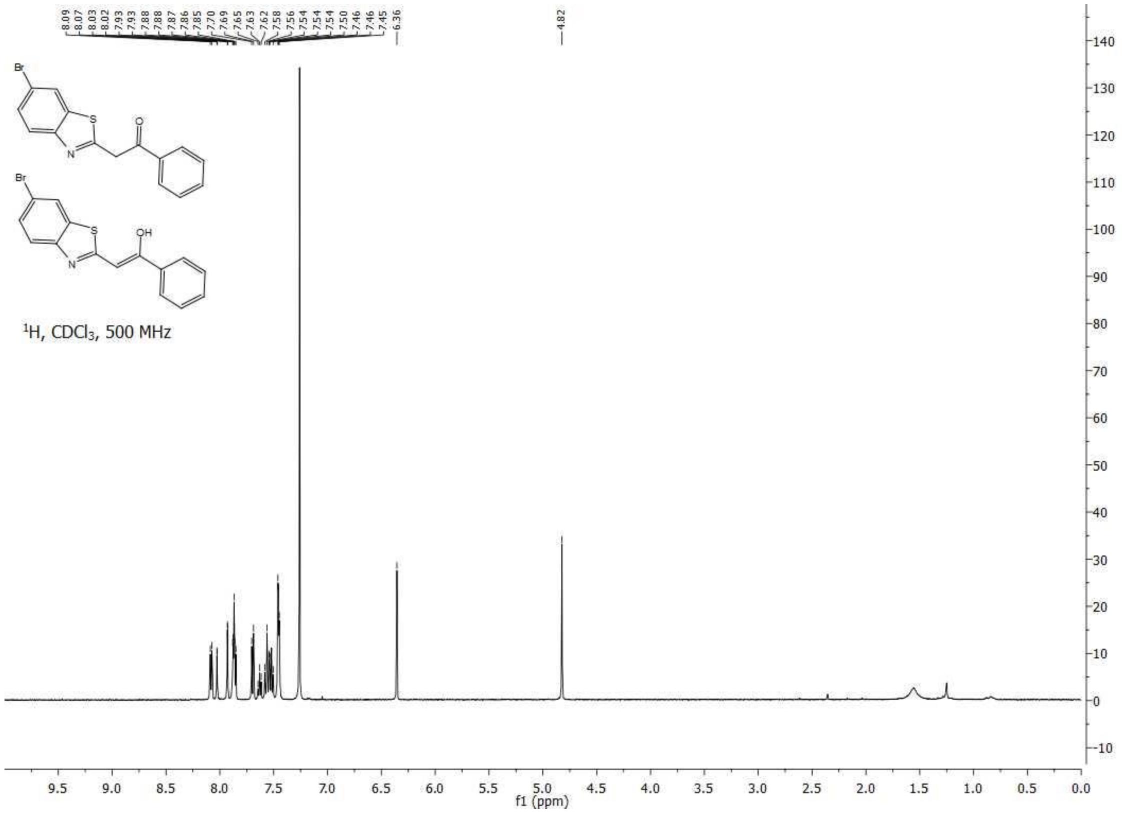

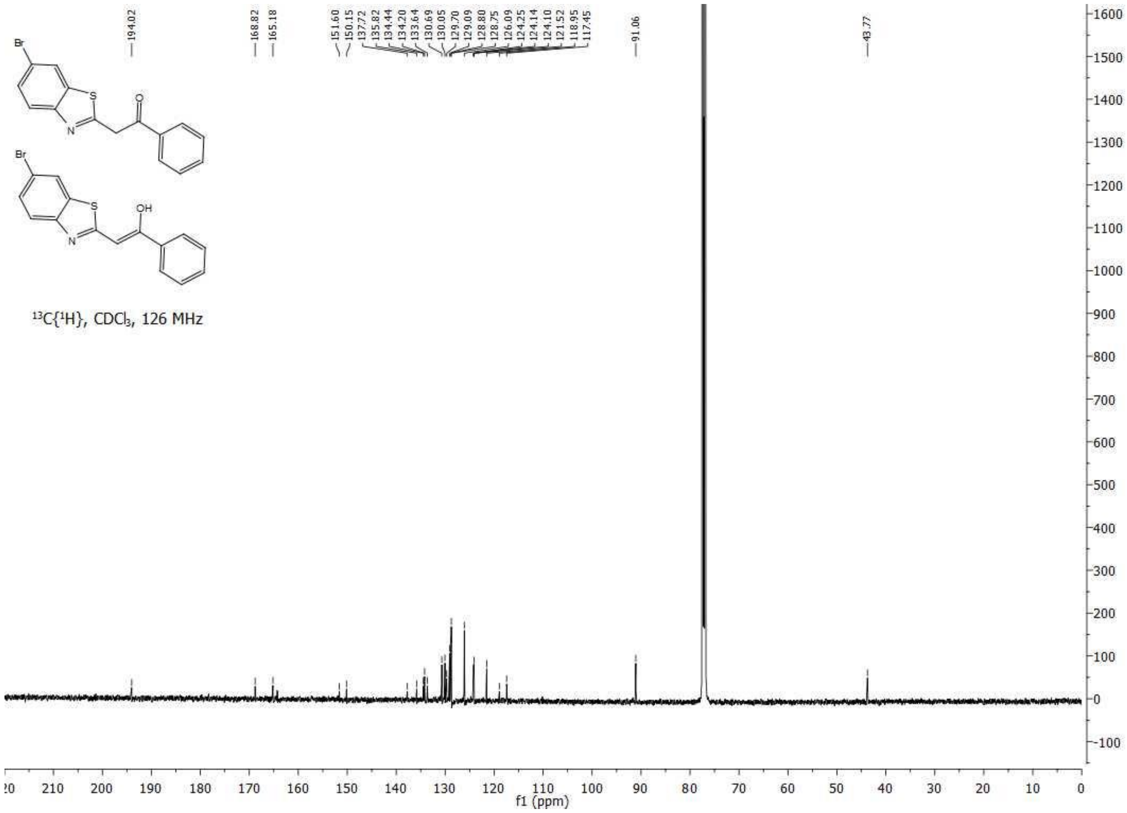

2-(6-Methoxybenzo[d]thiazol-2-yl)-1-phenylethan-1-one (S43)

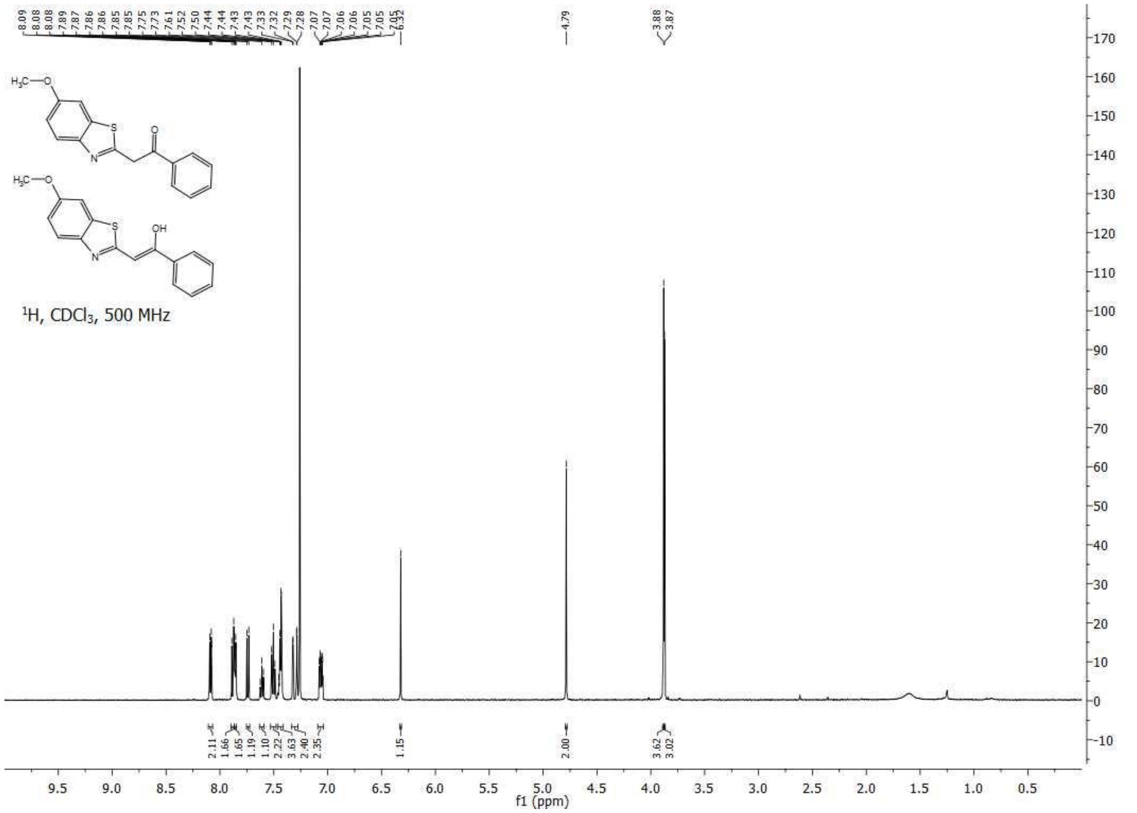

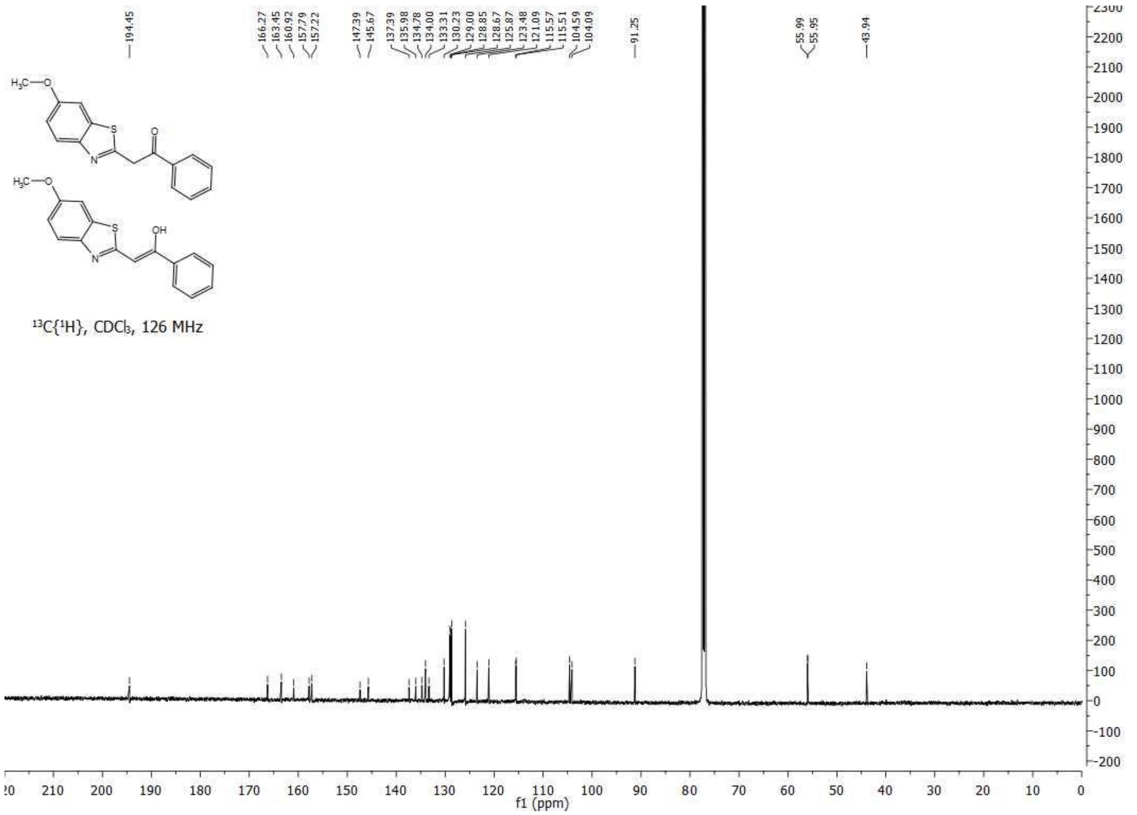

2-(4-Trifluoromethyl)phenacylbenzimidazole (S45)

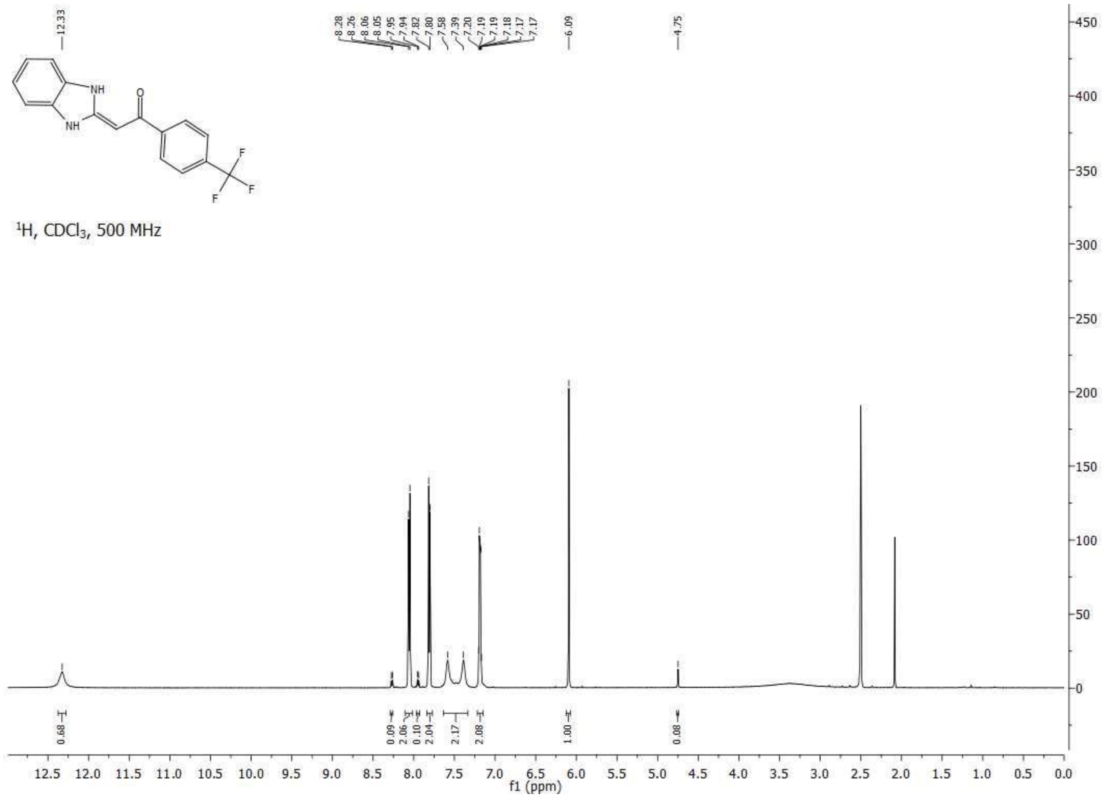

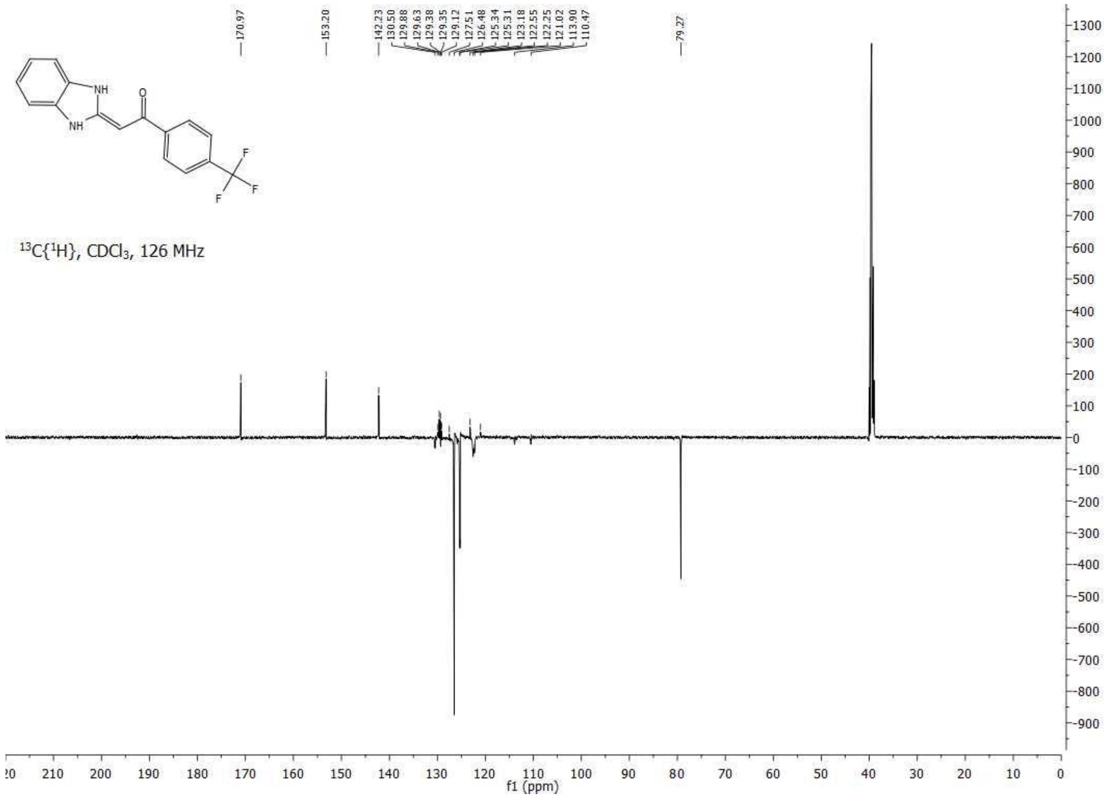

2-(4-Fluoro)phenacylbenzimidazole (S46)

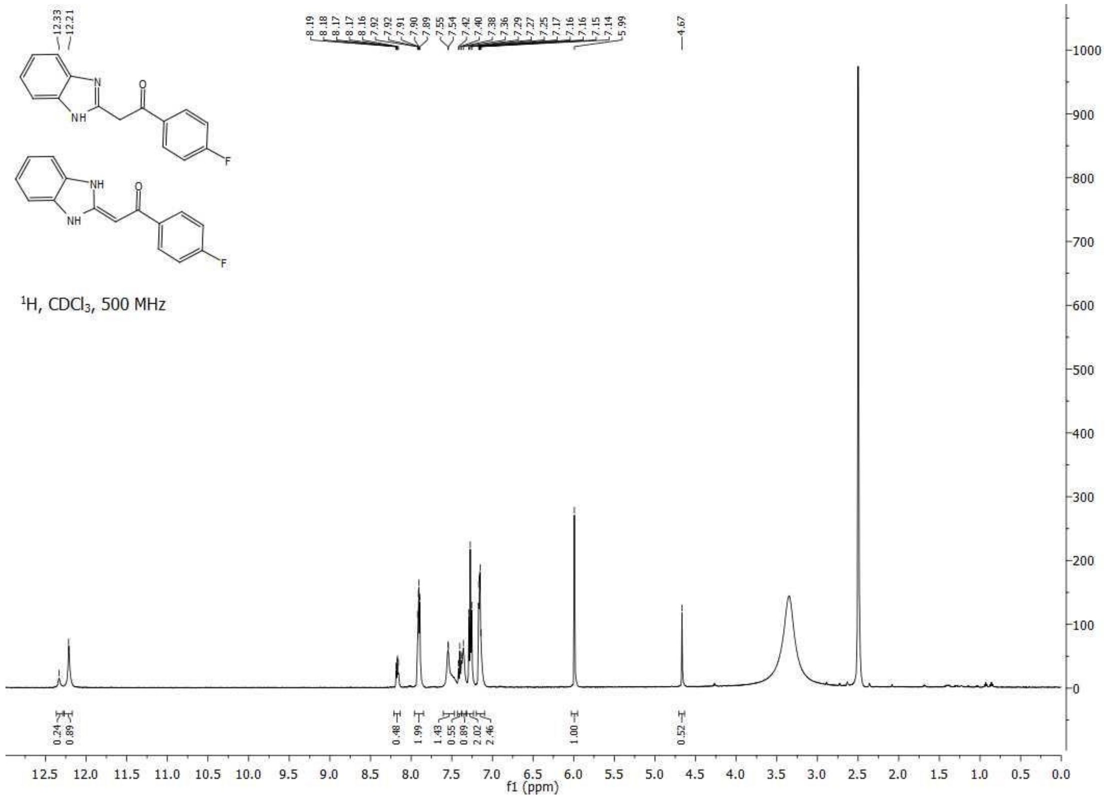

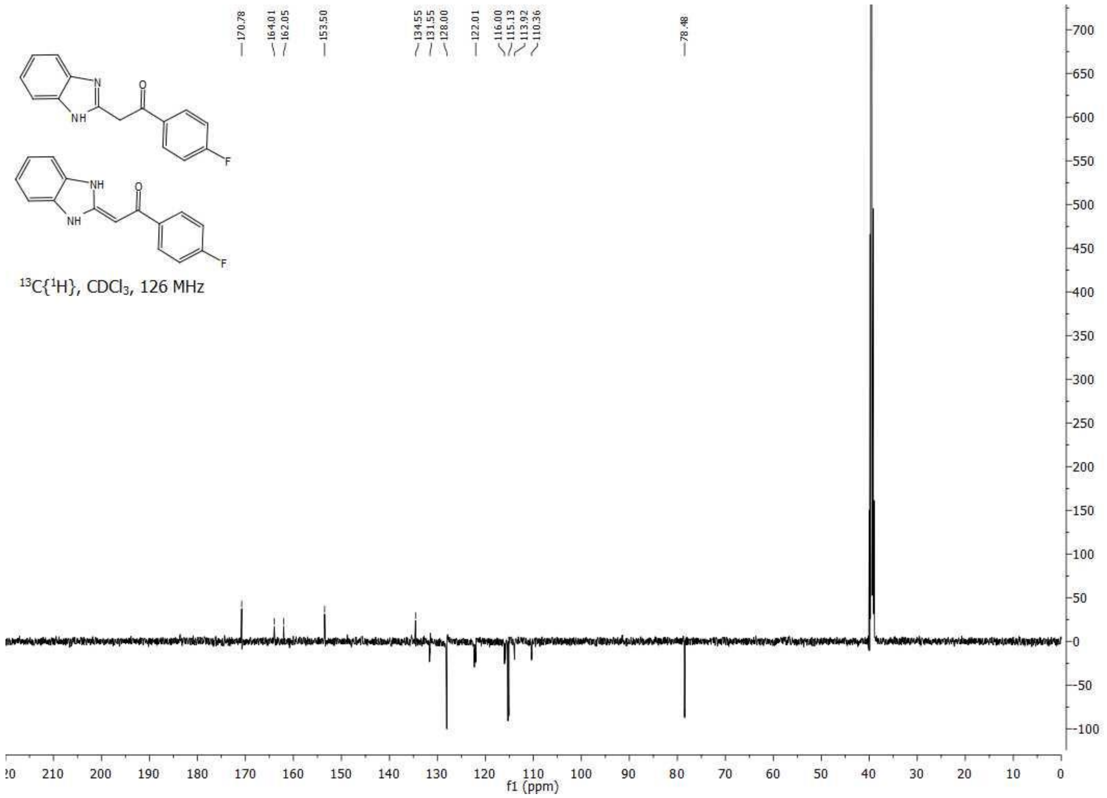

2-(3-Methyl)phenacylbenzimidazole (S48)

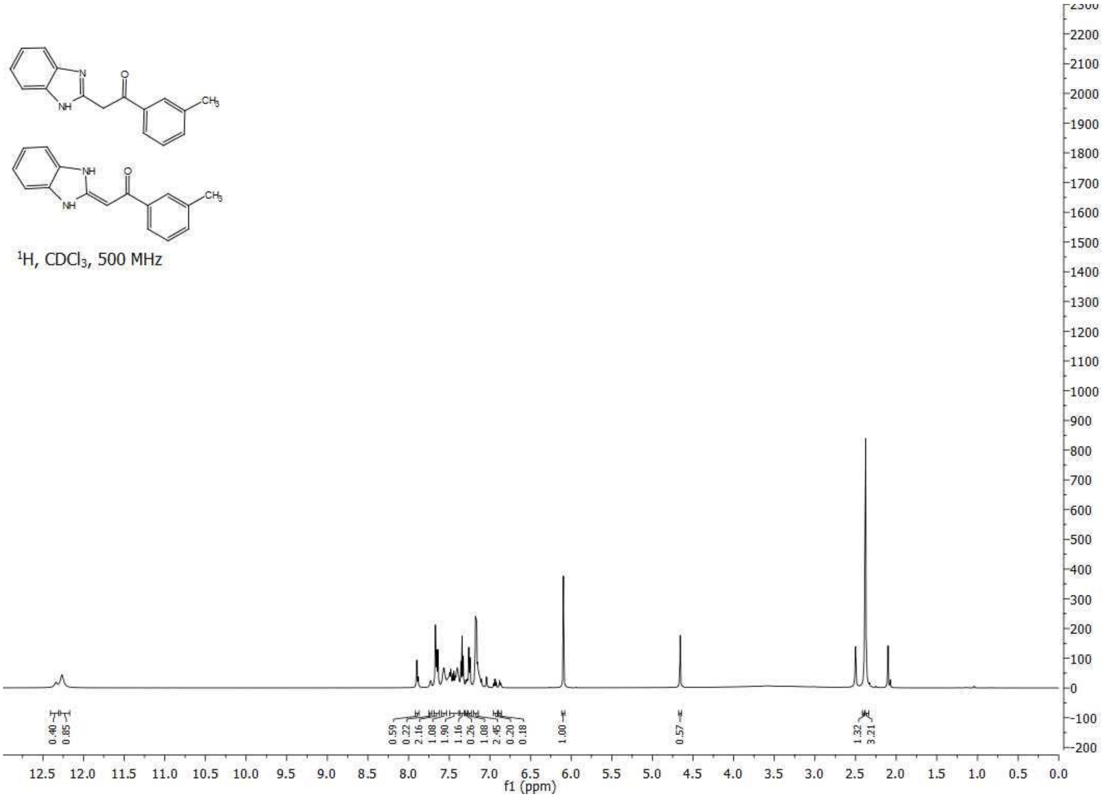

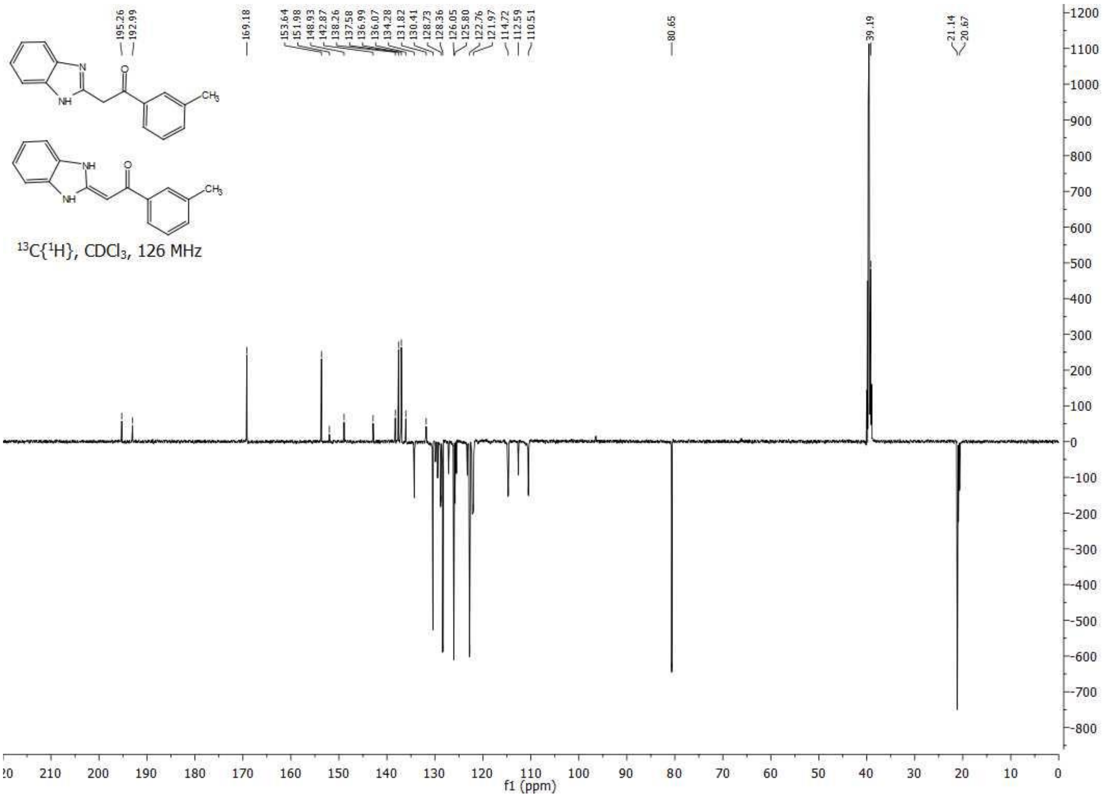

2-(Furan-2-yl)phenacylbenzimidazole (S49)

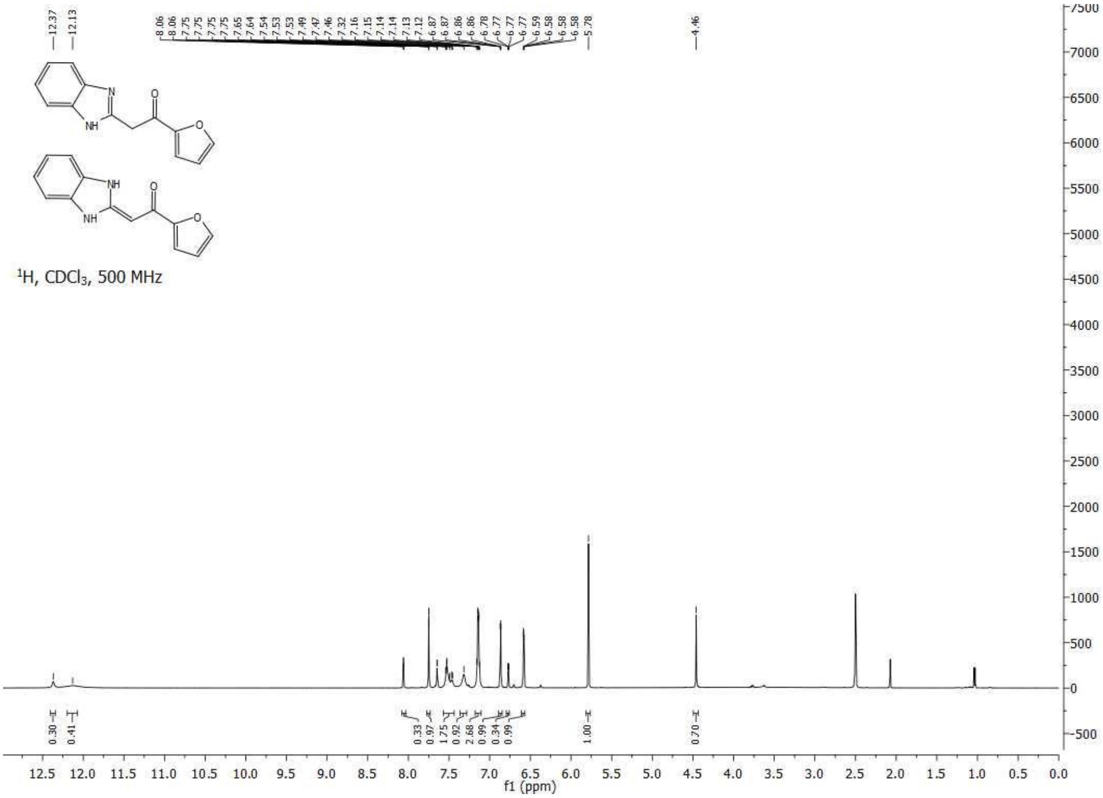

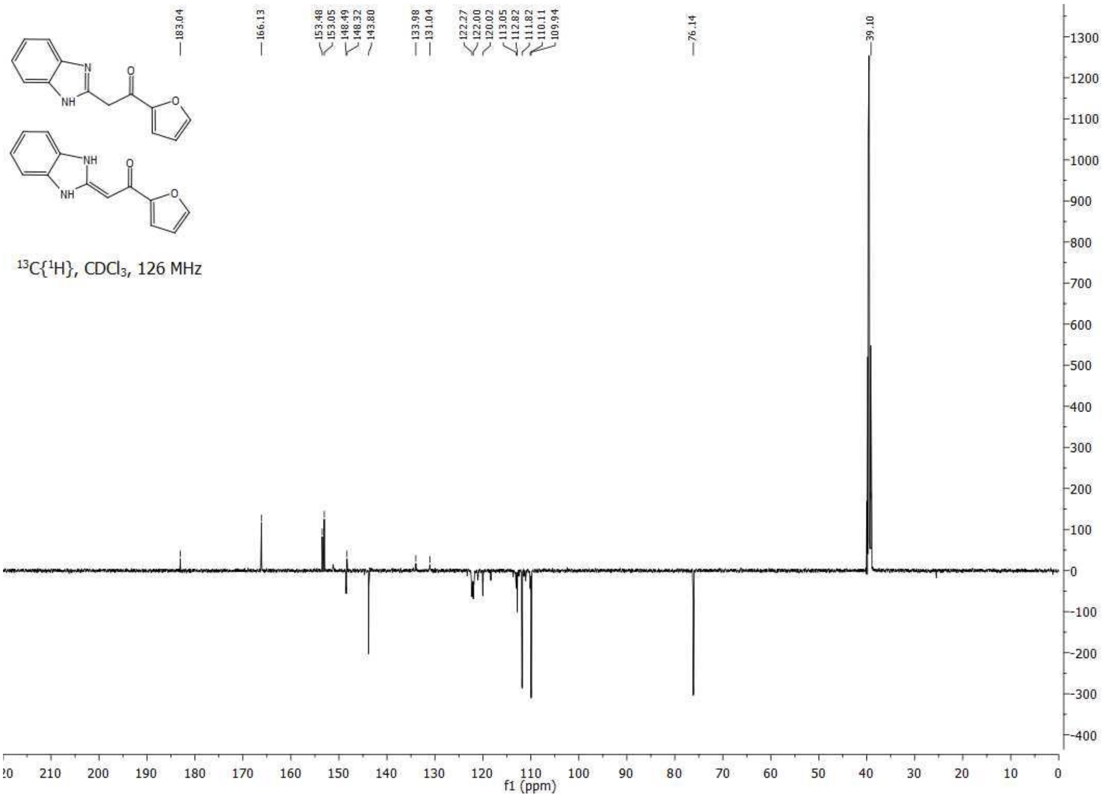

2-Phenacyl-(5-bromo)benzimidazole (S50)

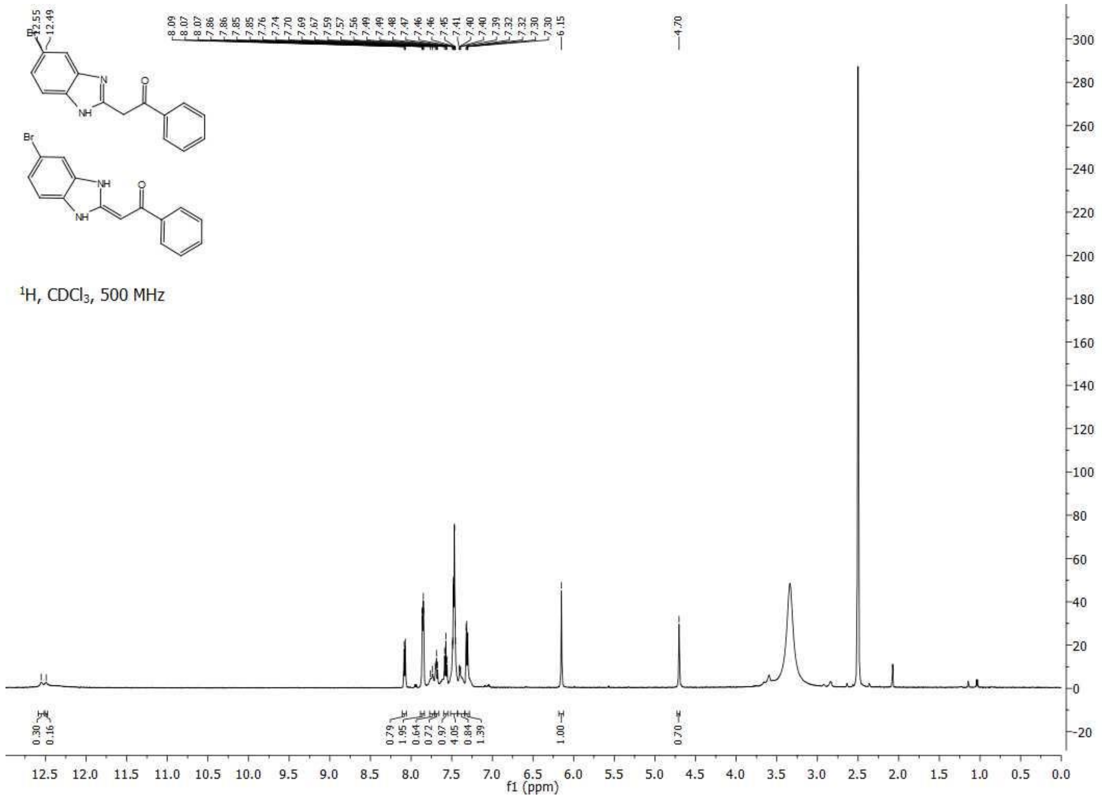

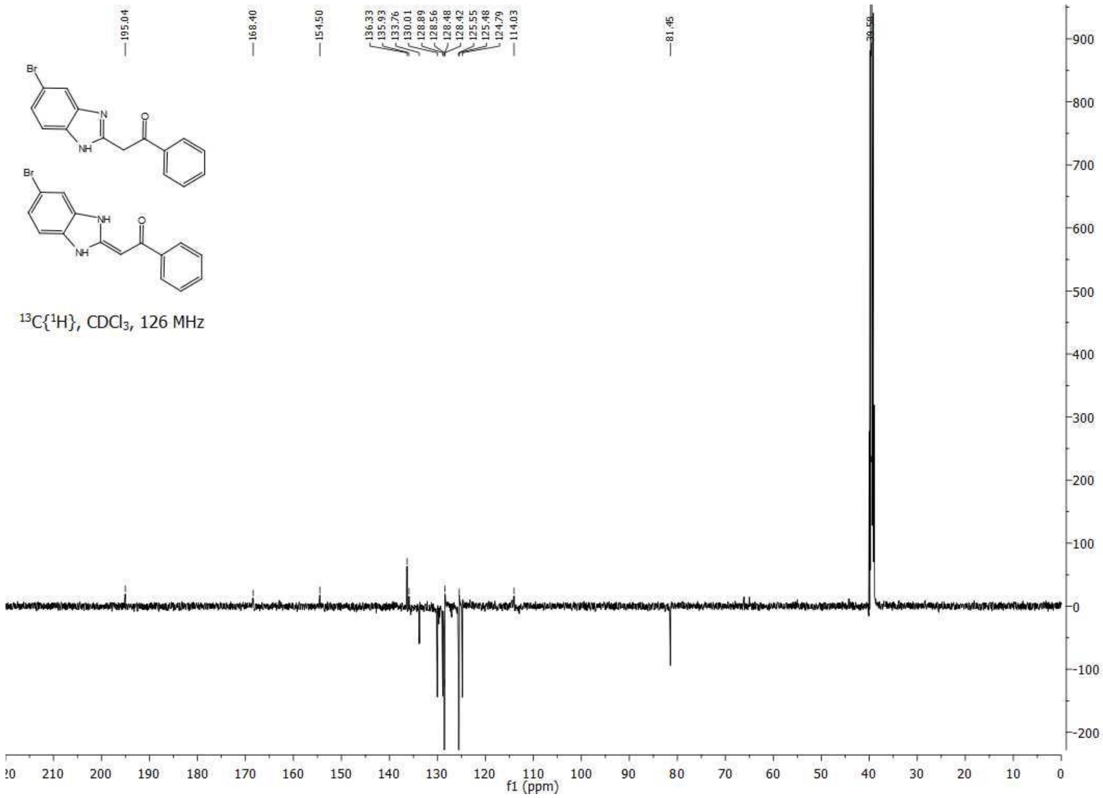

2-(4-Methoxy)phenacyl-(5-chloro)benzimidazole (S51)

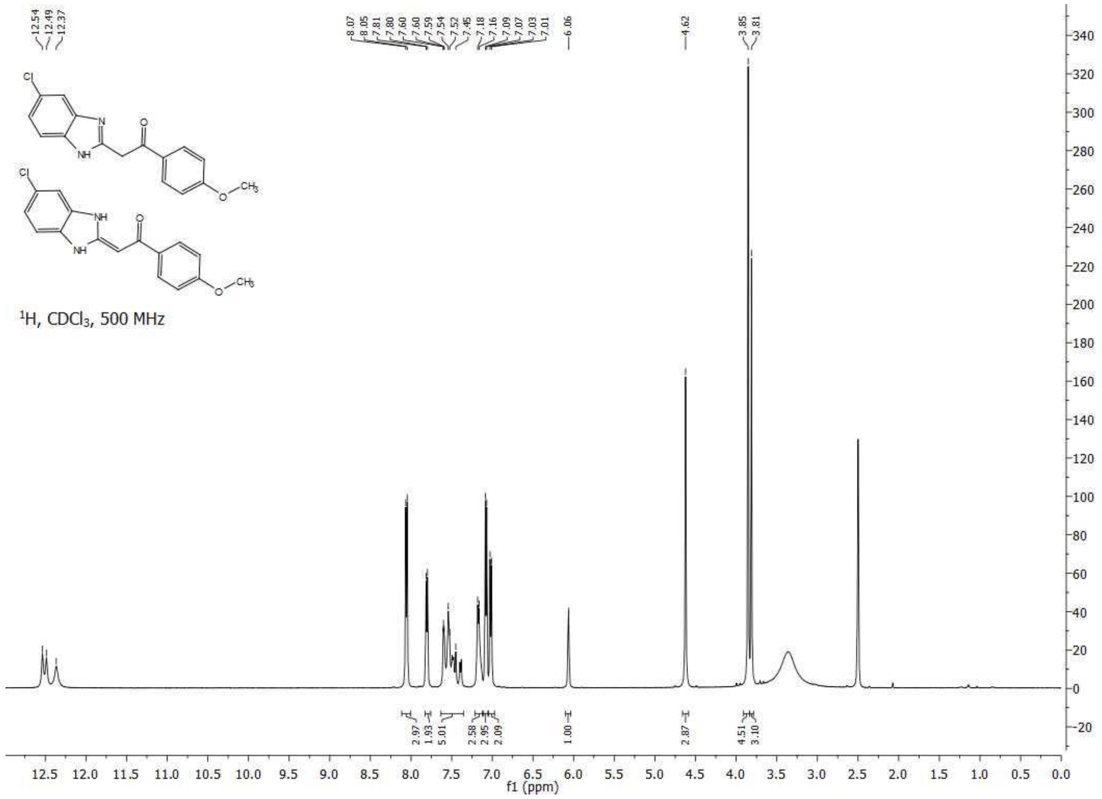

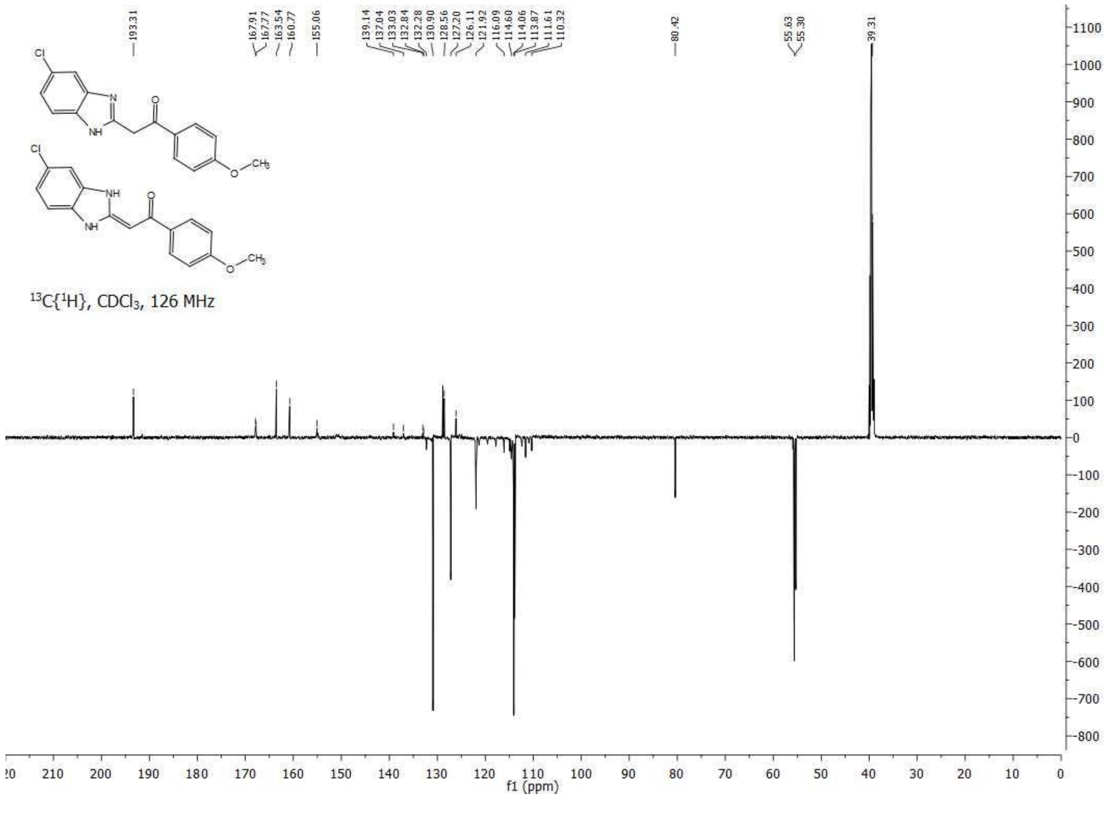

2-Phenacyl-(5,6-dimethyl)benzimidazole (S52)

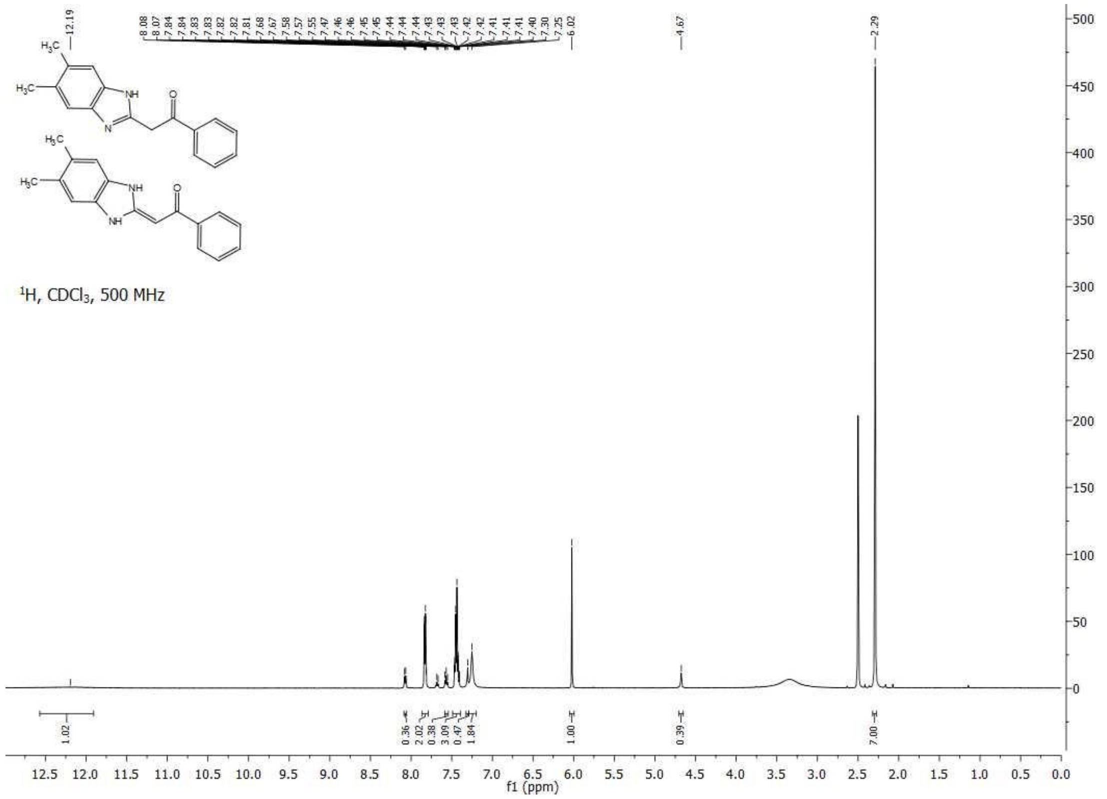

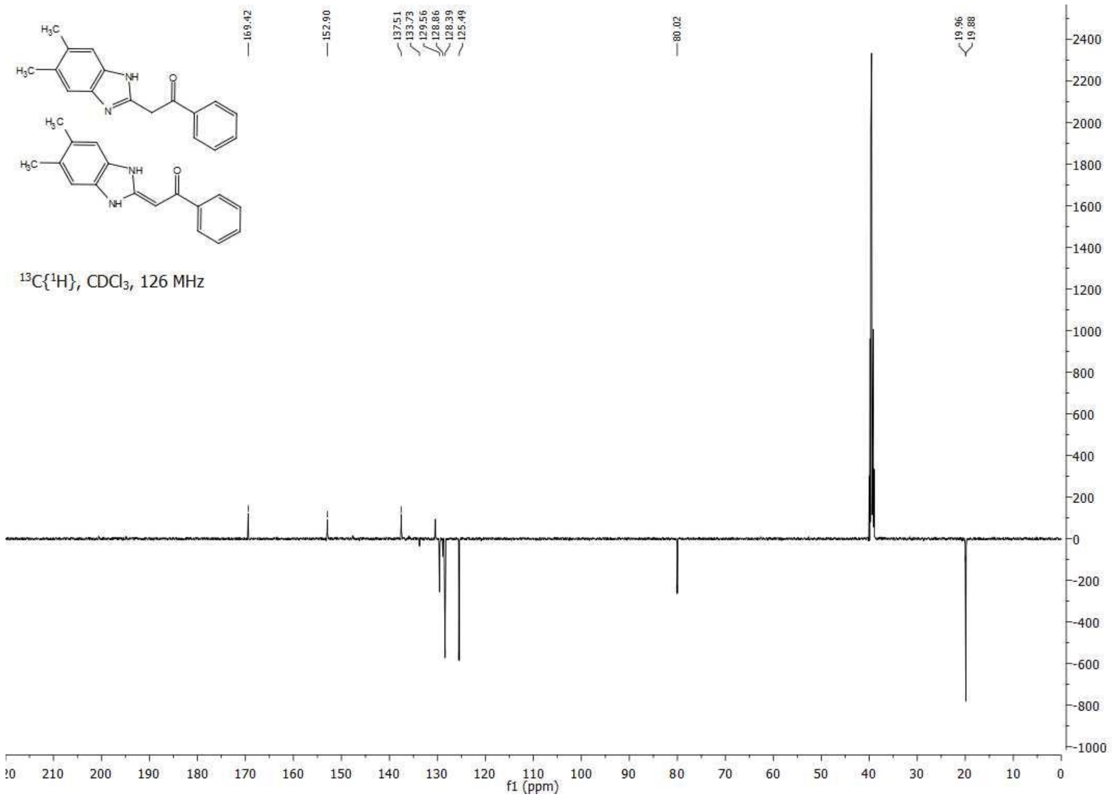

2-((4a*S*,9*S*,9a*R*)-3-Methyl-1-oxo-1,4a,9,9a-tetrahydroindeno[2,1-*c*]pyran-9-yl)-1,3-diphenylpropane-1,3-dione (7a)

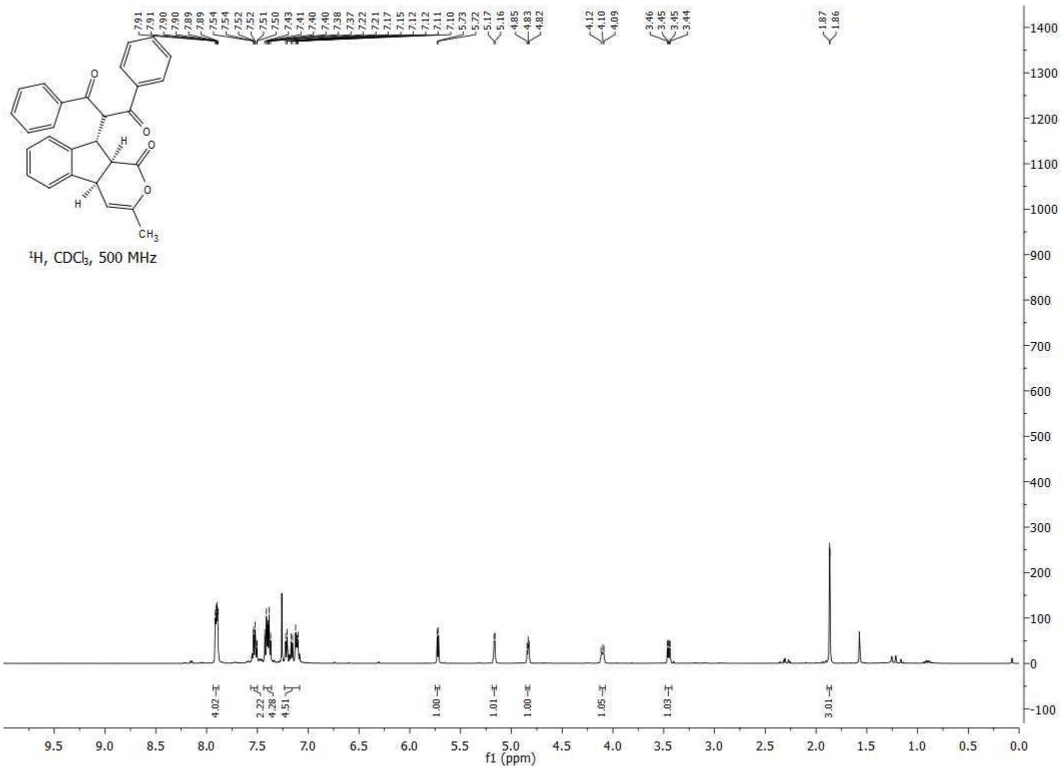

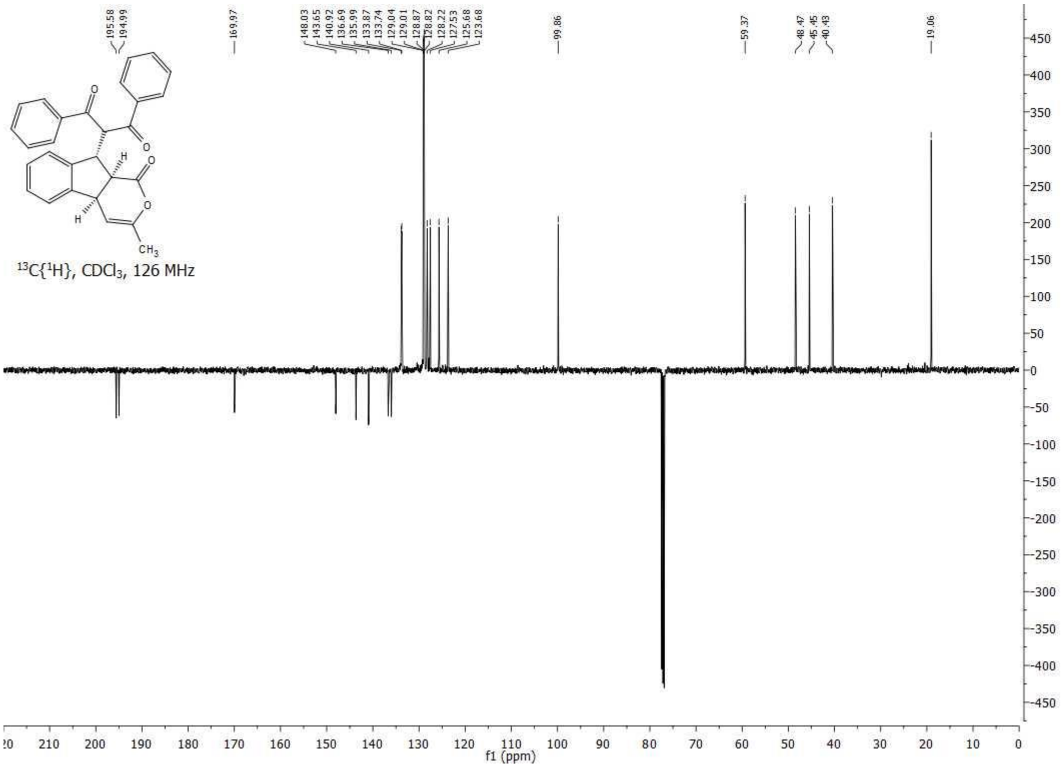

(4aR,9S,9aR)-4-Benzoyl-9-(2-oxopropyl)-3-phenyl-9,9a-dihydroindeno[2,1-*c*]pyran-1(4aH)-one (7a and 7b mixture)

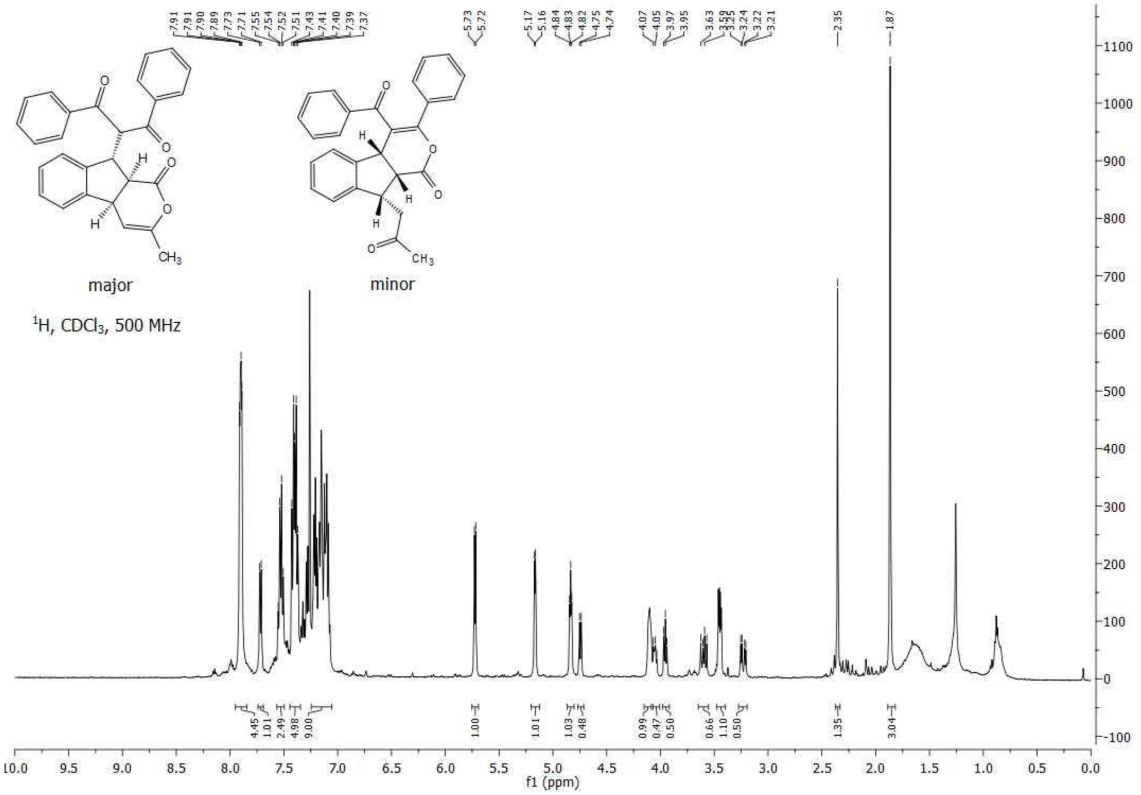

1,3-Bis(4-methoxyphenyl)-2-((4a*R*,9*R*,9a*S*)-3-methyl-1-oxo-1,4a,9,9a-tetrahydroindeno[2,1-*c*]pyran-9-yl)propane-1,3-dione (10a)

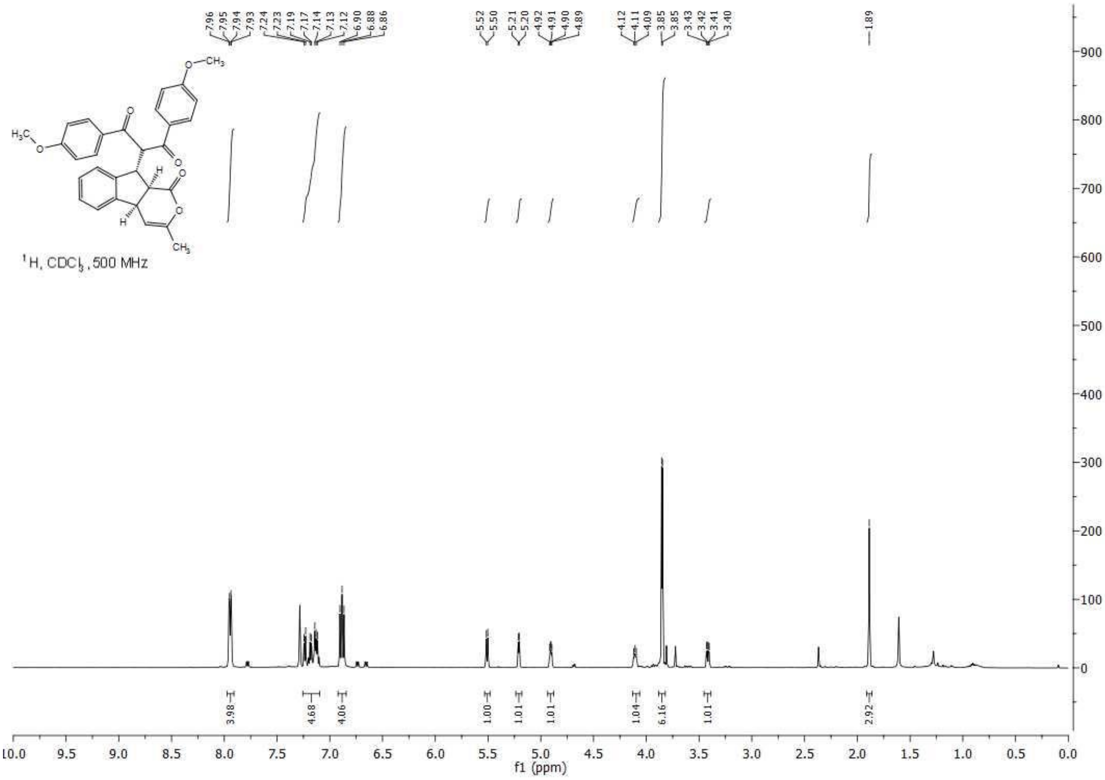

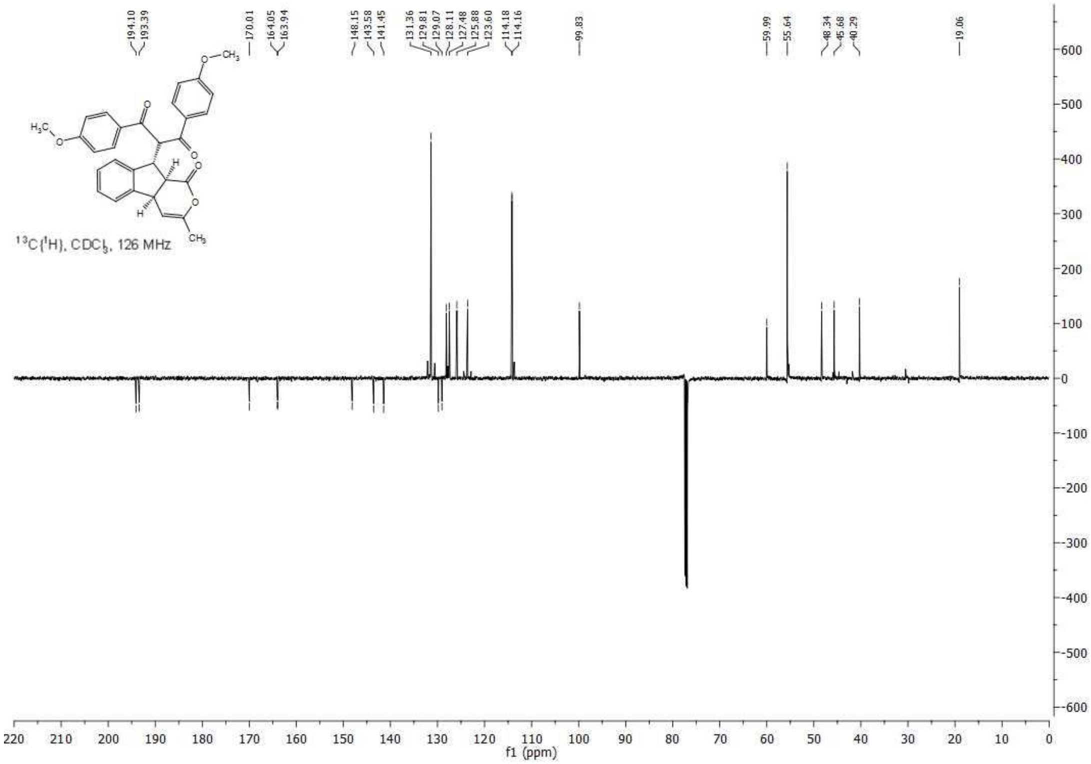

1,3-Bis(4-fluorophenyl)-2-((4a*R*,9*R*,9a*S*)-3-methyl-1-oxo-1,4a,9,9a-tetrahydroindeno[2,1-*c*]pyran-9-yl)propane-1,3-dione (11a)

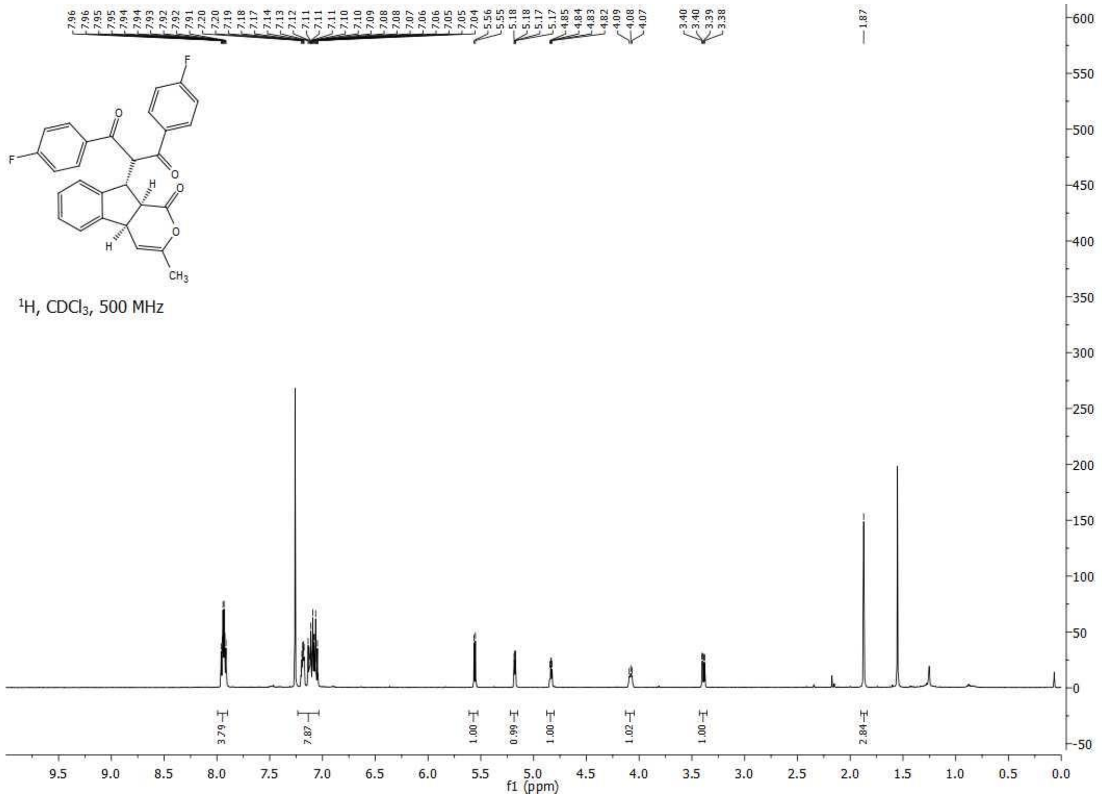

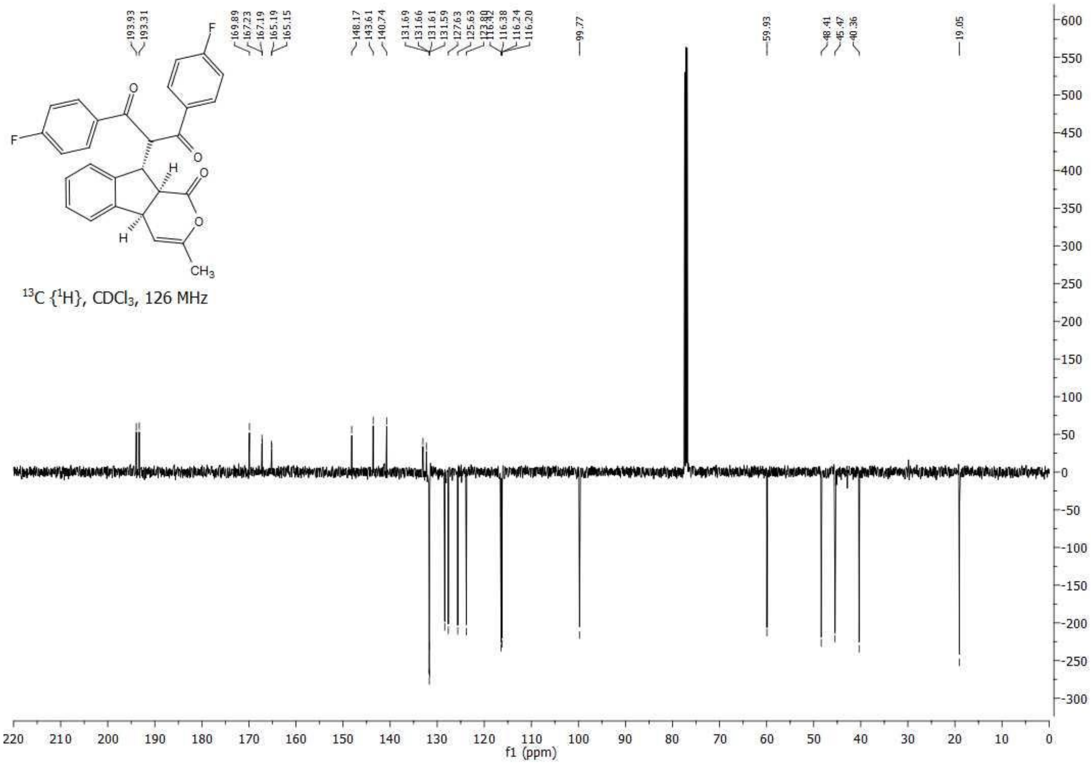

1,3-Di(furan-2-yl)-2-((4a*R*,9*R*,9a*S*)-3-methyl-1-oxo-1,4a,9,9a-tetrahydroindeno[2,1-*c*]pyran-9-yl)propane-1,3-dione (12a)

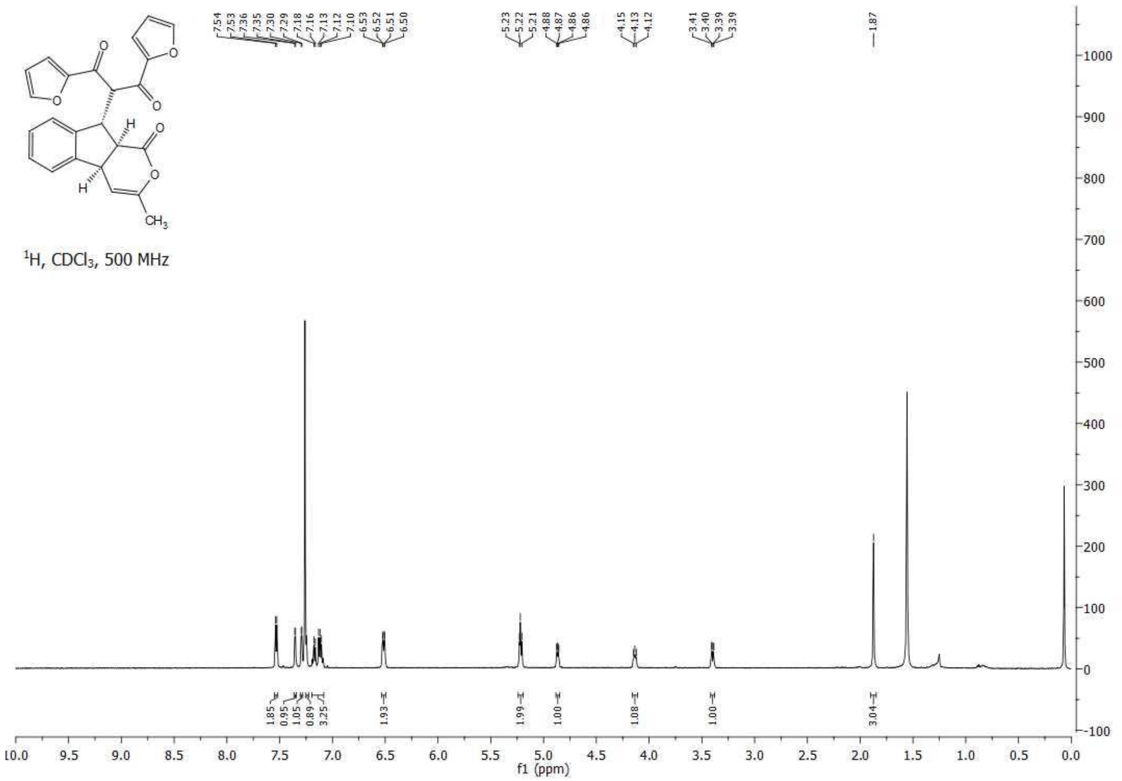

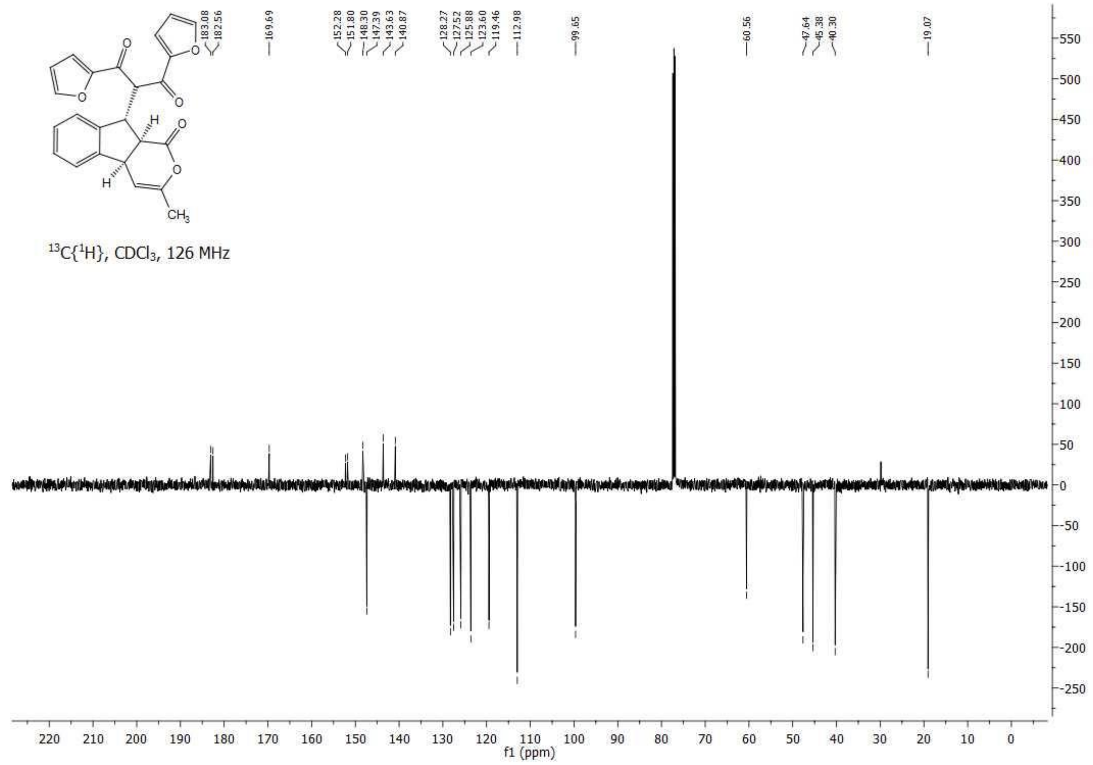

3-((4a*R*,9*R*,9a*S*)-3-Methyl-1-oxo-1,4a,9,9a-tetrahydroindeno[2,1-*c*]pyran-9-yl)pentane-2,4-dione (13a)

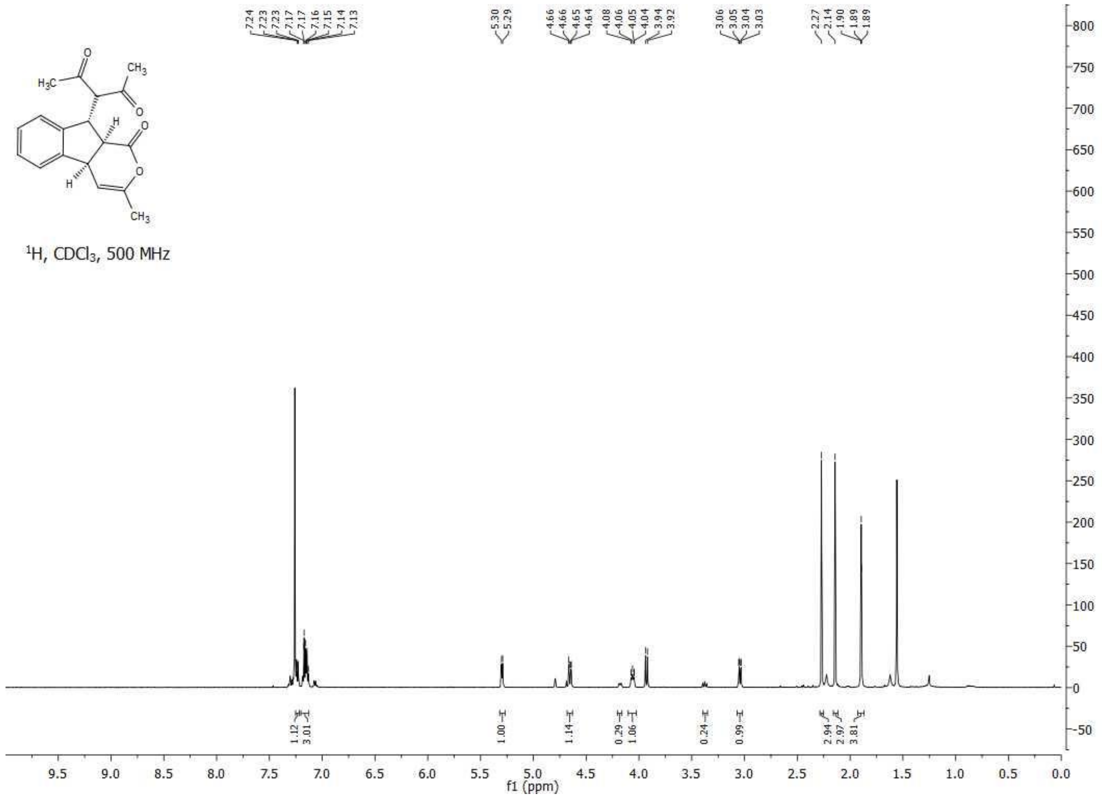

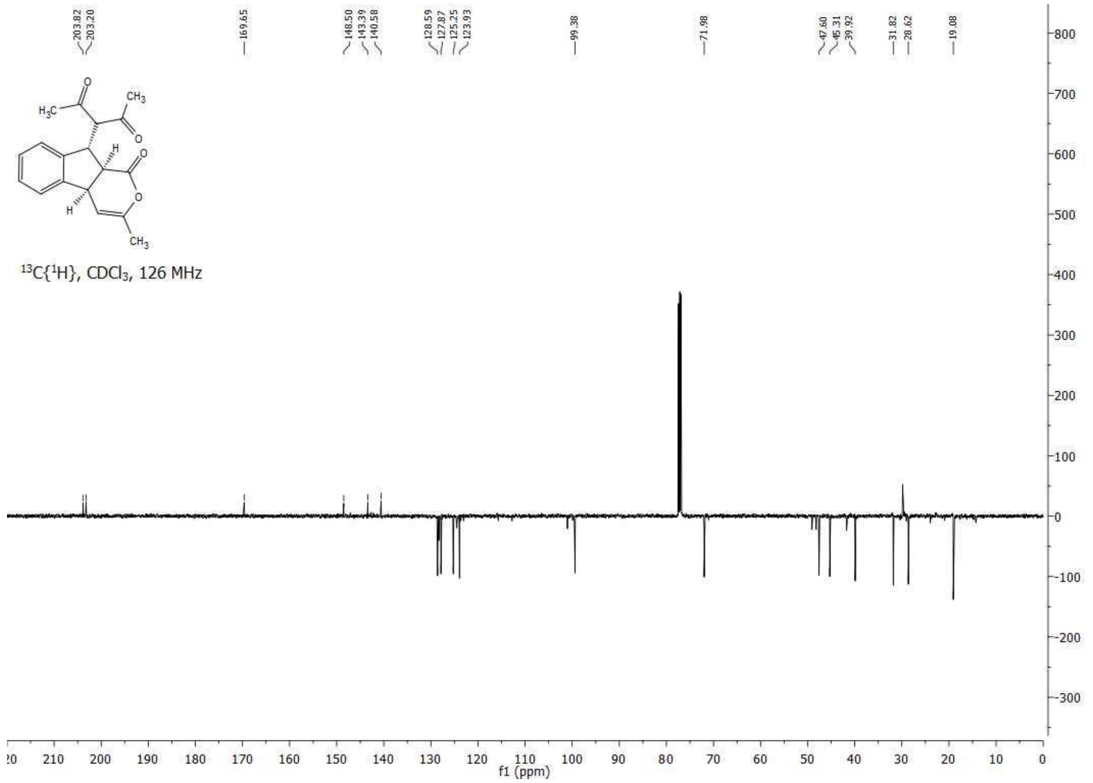

Dimethyl 2-((4aS,9R,9aR)-3-methyl-1-oxo-1,4a,9,9a-tetrahydroindeno[2,1-c]pyran-9-yl)malonate (14a)

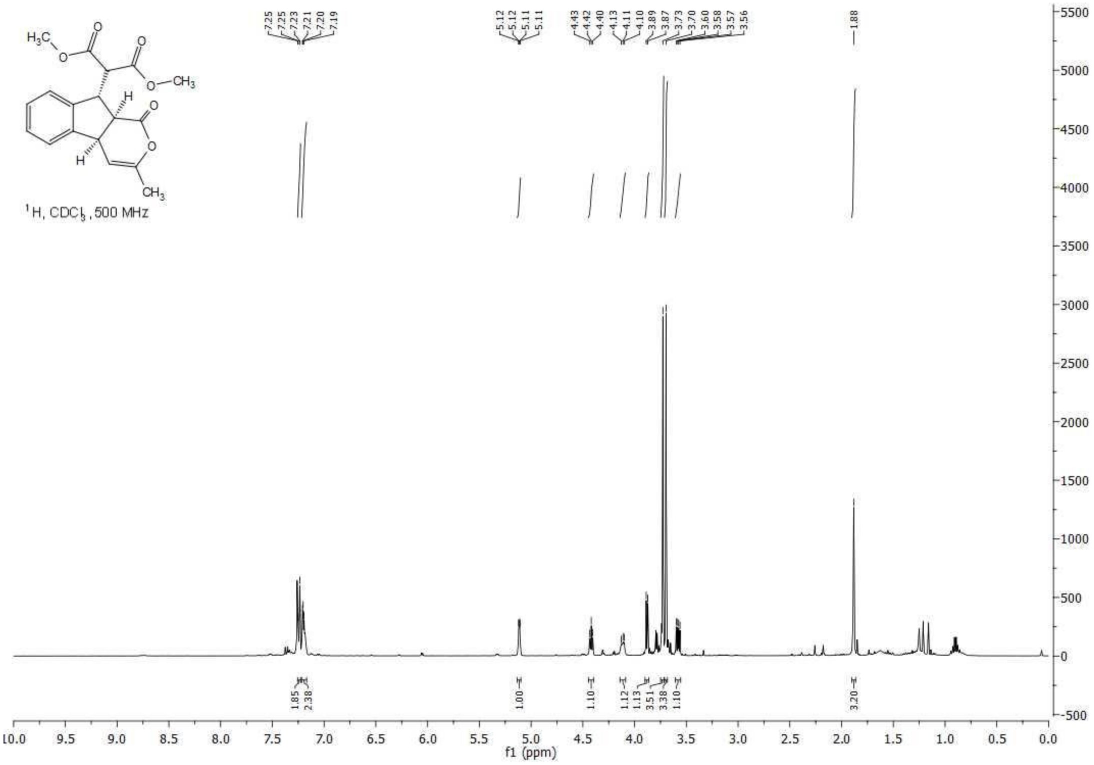

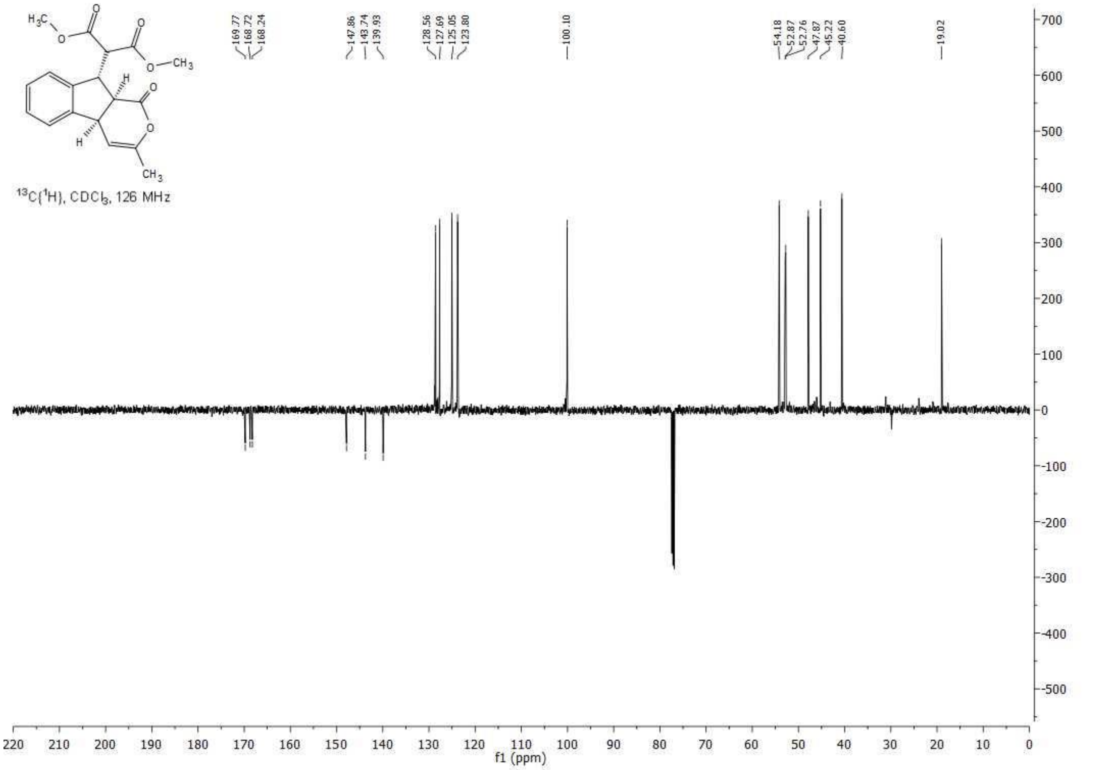

Diisopropyl 2-((4a*R*,9*S*,9a*S*)-3-methyl-1-oxo-1,4a,9,9a-tetrahydroindeno[2,1-*c*]pyran-9-yl)malonate (15a)

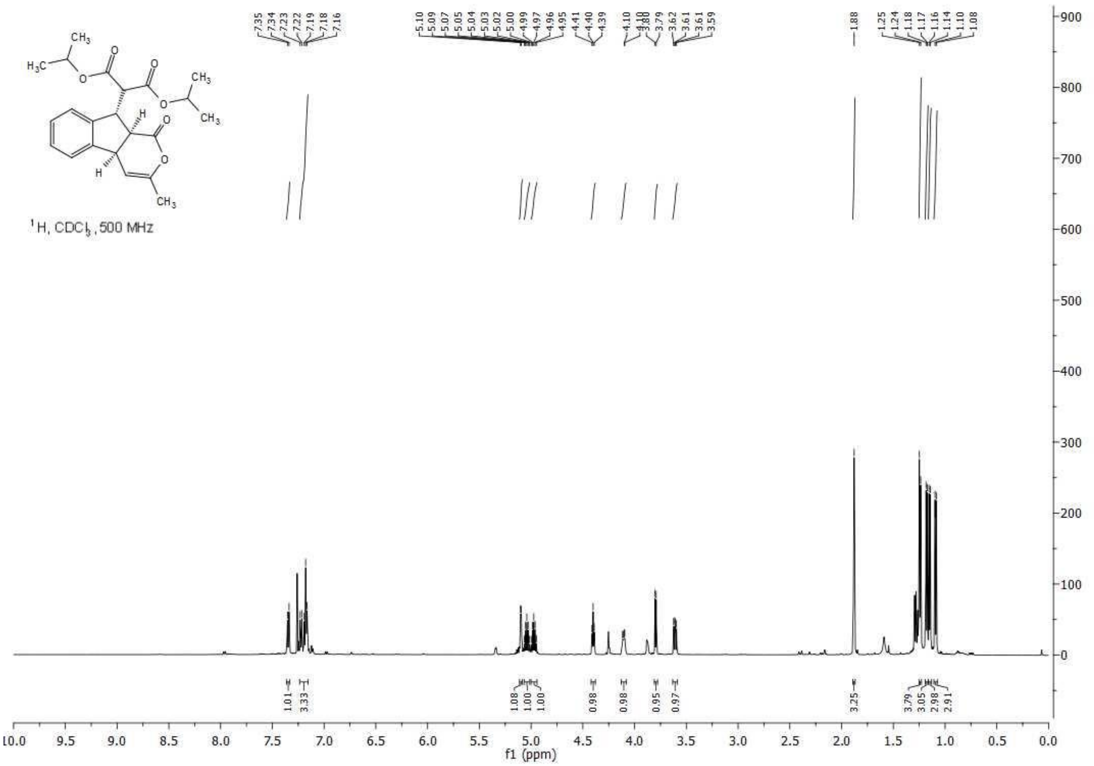

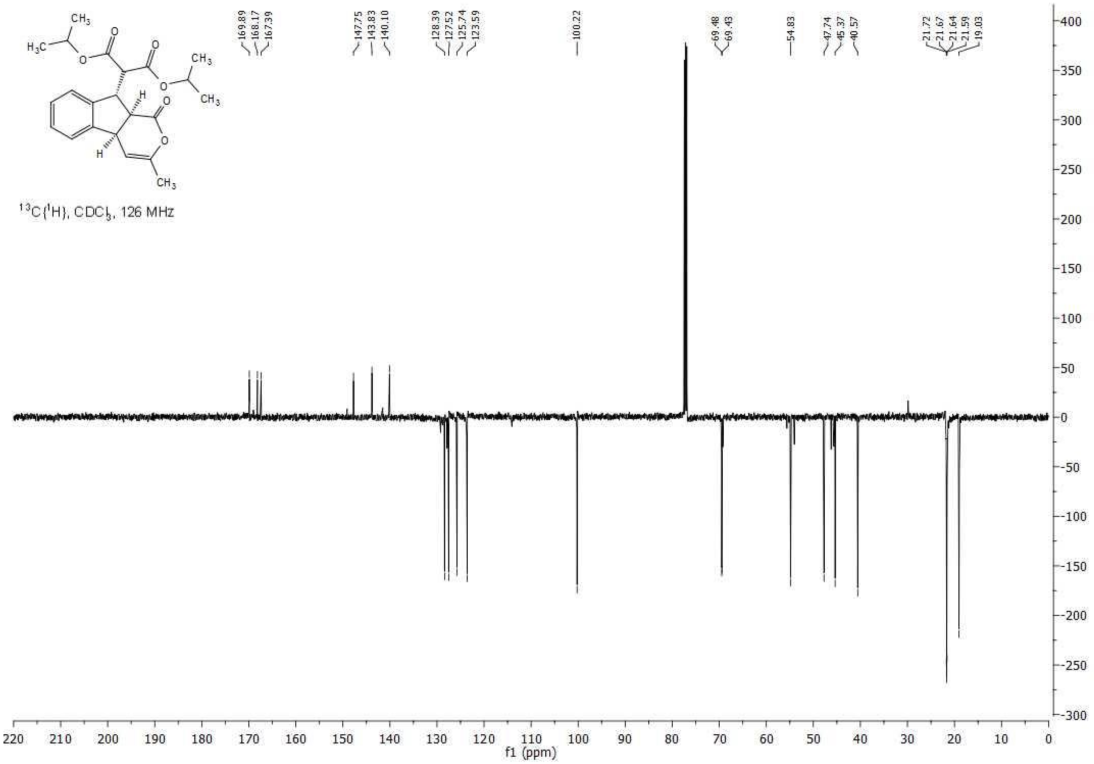

Ethyl 2-((4aR,9S,9aS)-3-methyl-1-oxo-1,4a,9,9a-tetrahydroindeno[2,1-*c*]pyran-9-yl)-3-oxo-3-phenylpropanoate (±16a)

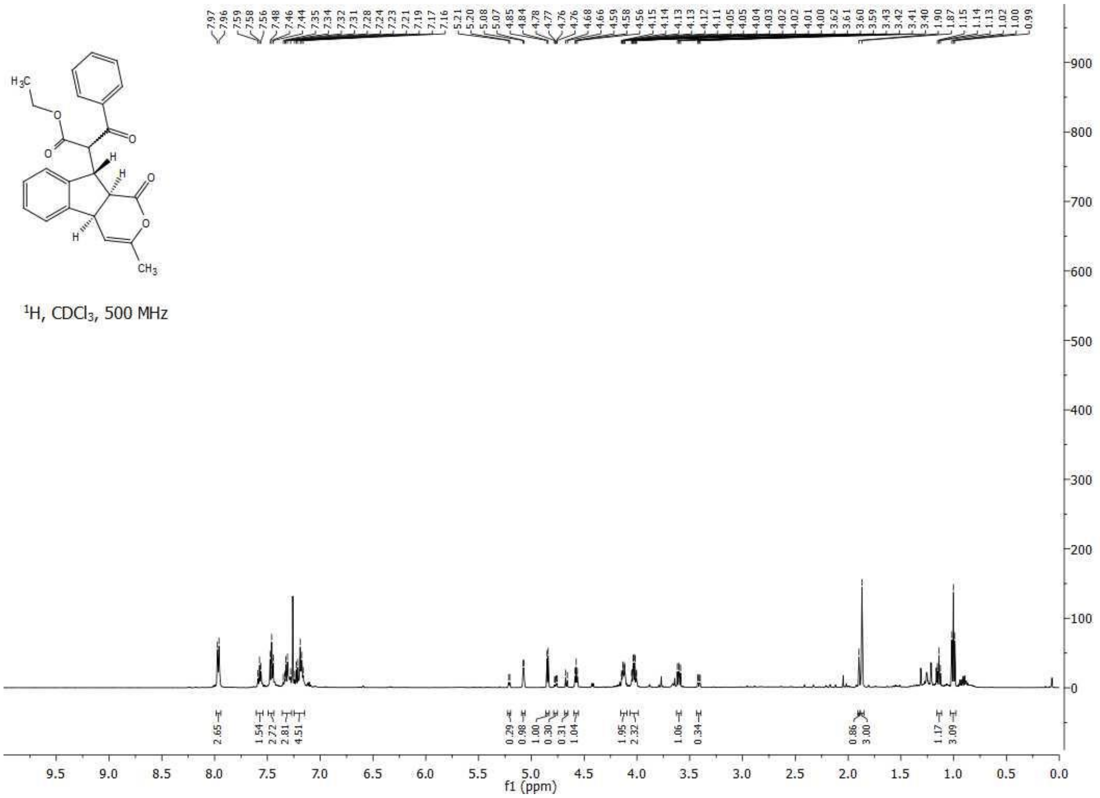

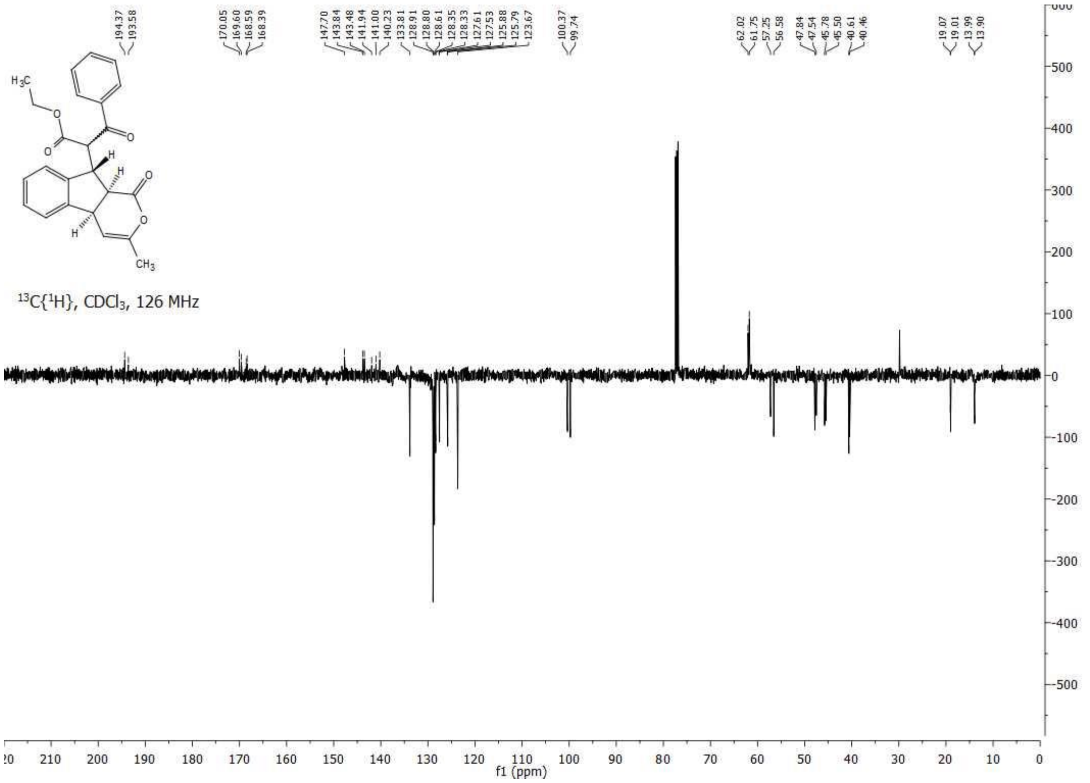

2-((4a*R*,9*R*,9a*S*)-6-Chloro-3-methyl-1-oxo-1,4a,9,9a-tetrahydroindeno[2,1-*c*]pyran-9-yl)-1,3-diphenylpropane-1,3-dione (17a)

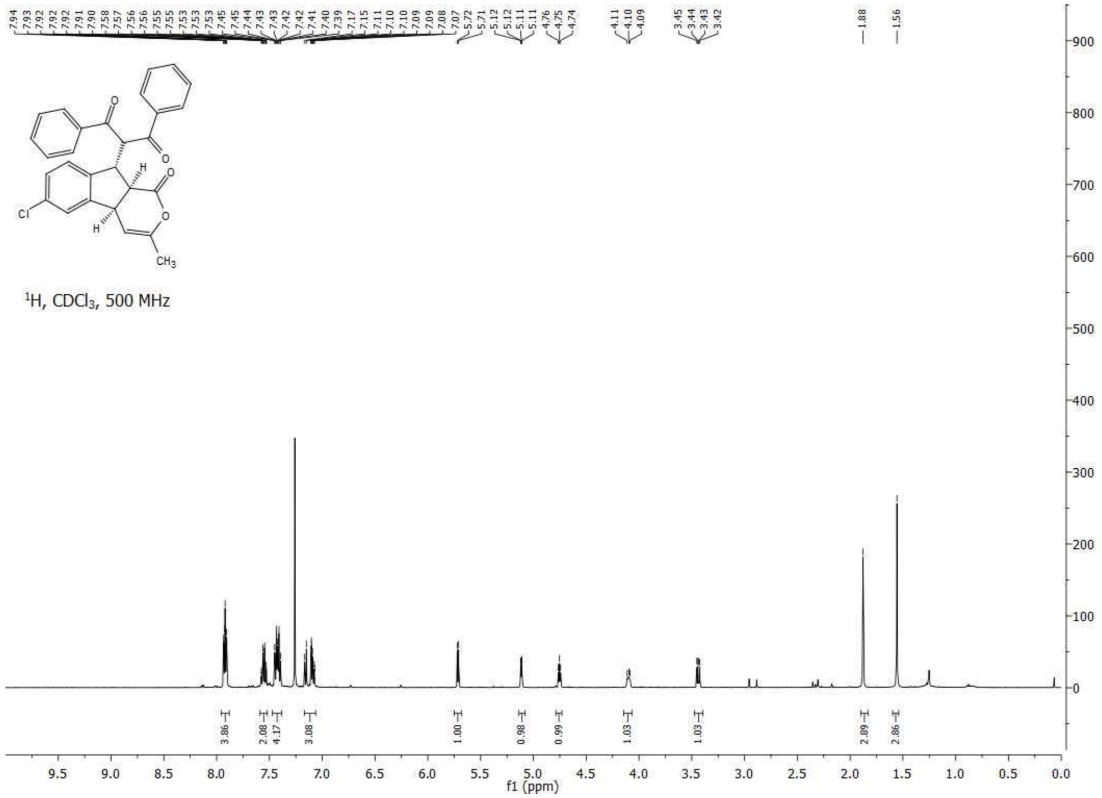

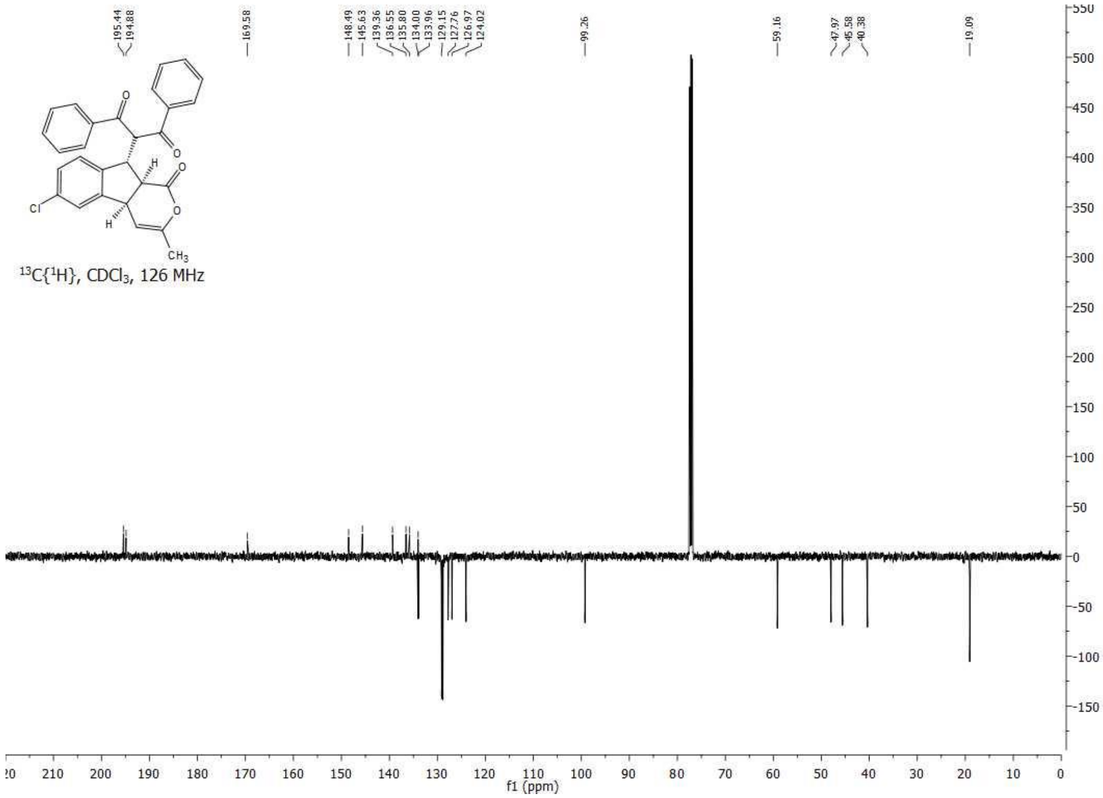

2-((4a*R*,9*R*,9a*S*)-6-Fluoro-3-methyl-1-oxo-1,4a,9,9a-tetrahydroindeno[2,1-*c*]pyran-9-yl)-1,3-diphenylpropane-1,3-dione (18a)

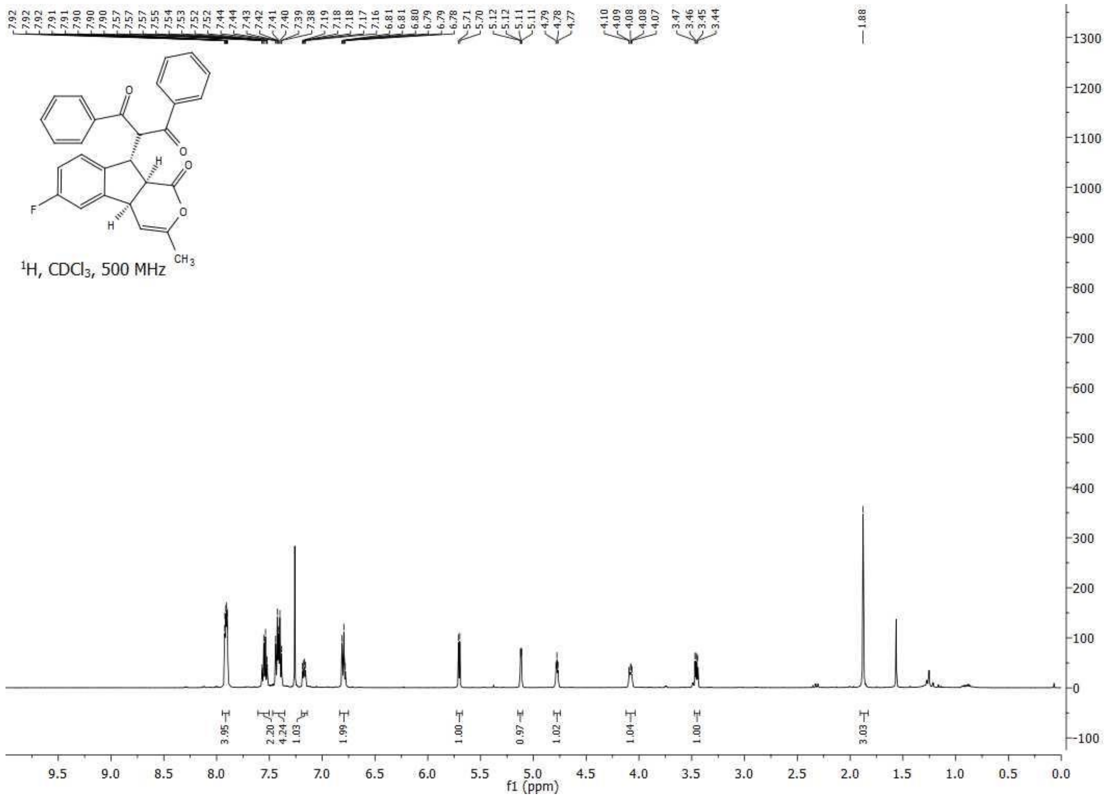

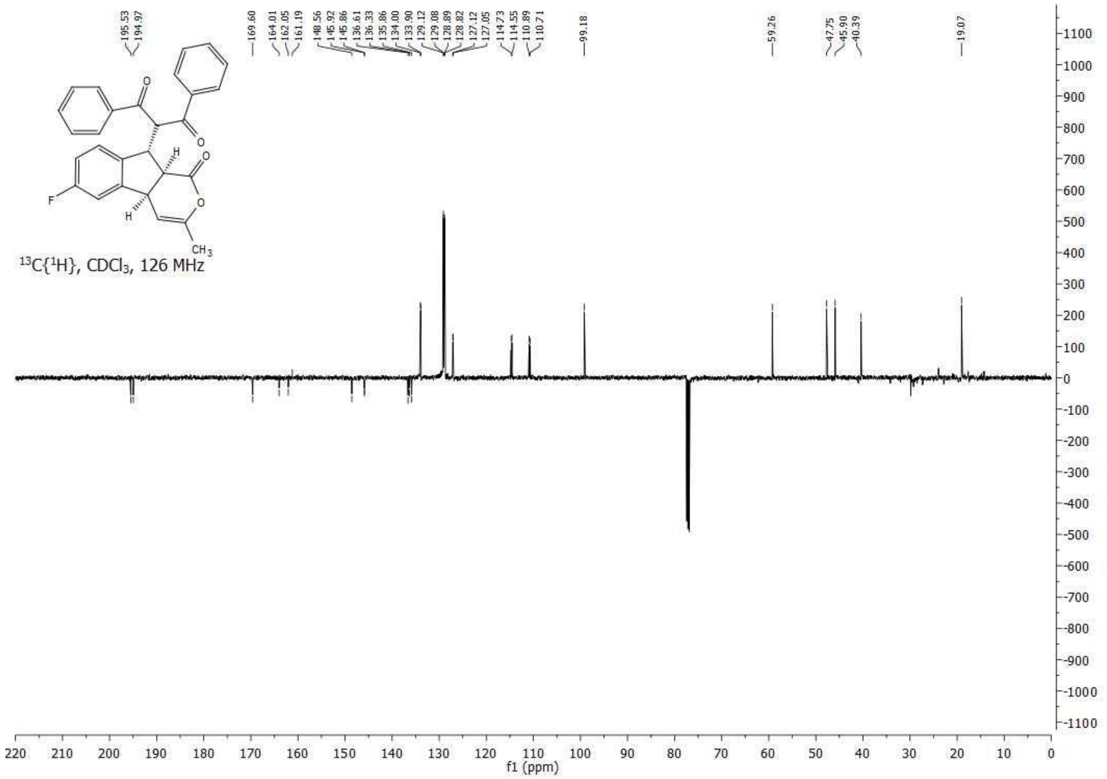

2-((4a*R*,9*R*,9a*S*)-3,7-Dimethyl-1-oxo-1,4a,9,9a-tetrahydroindeno[2,1-*c*]pyran-9-yl)-1,3-diphenylpropane-1,3-dione (19a)

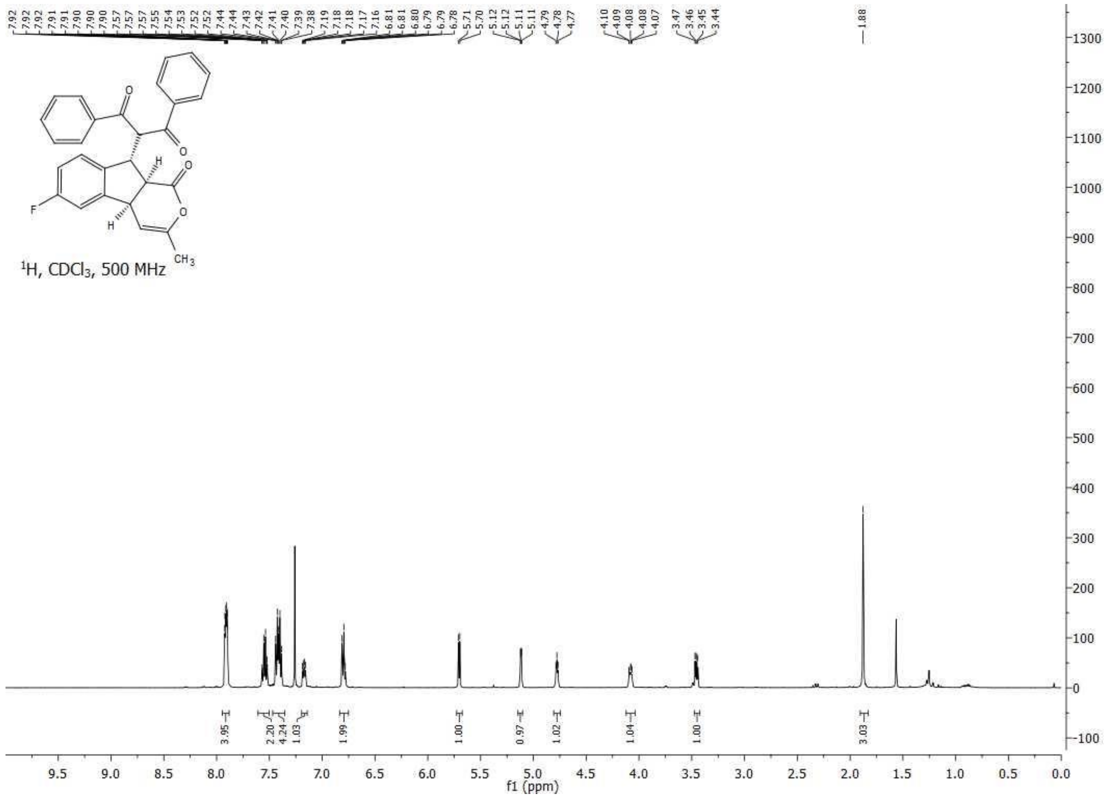

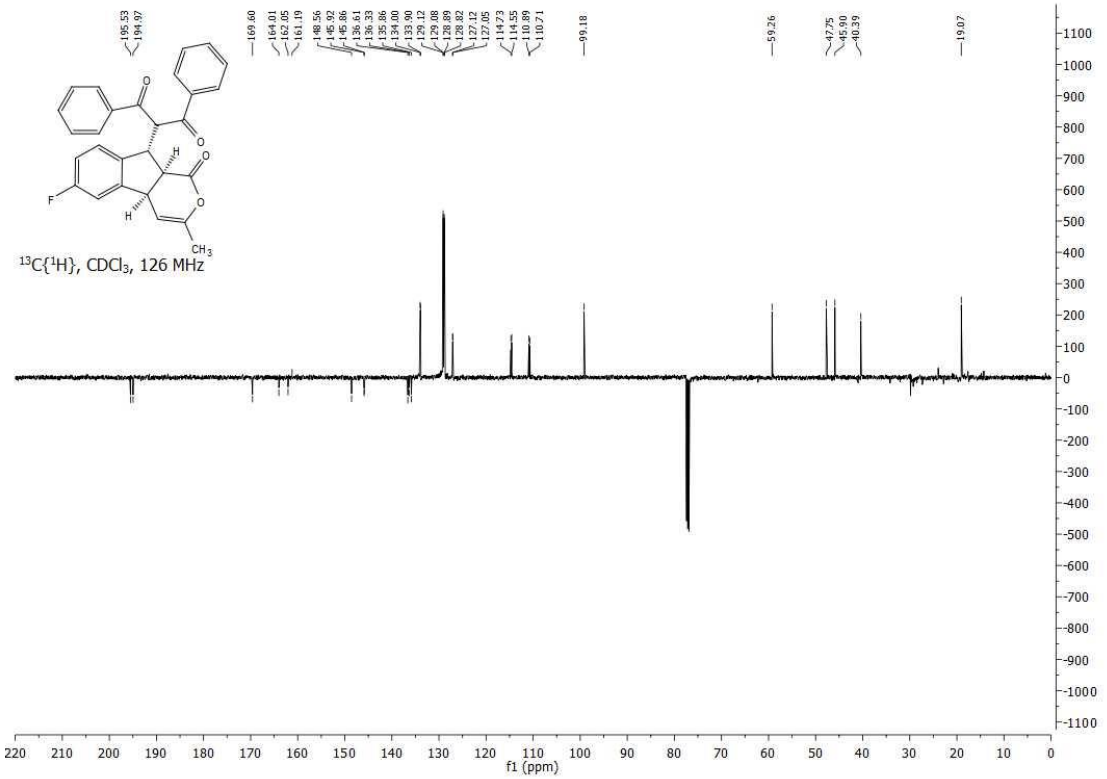

2-((4a*R*,9*R*,9a*S*)-3-(*tert*-Butyl)-1-oxo-1,4a,9,9a-tetrahydroindeno[2,1-*c*]pyran-9-yl)-1,3-diphenylpropane-1,3-dione (20a)

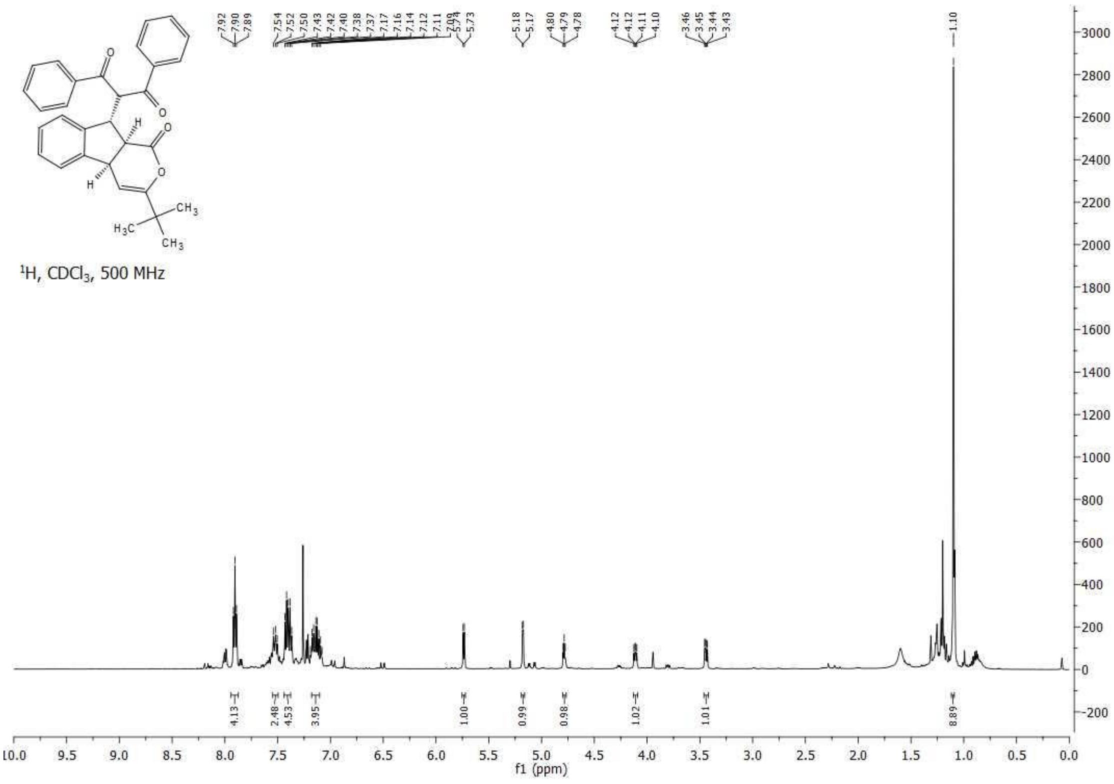

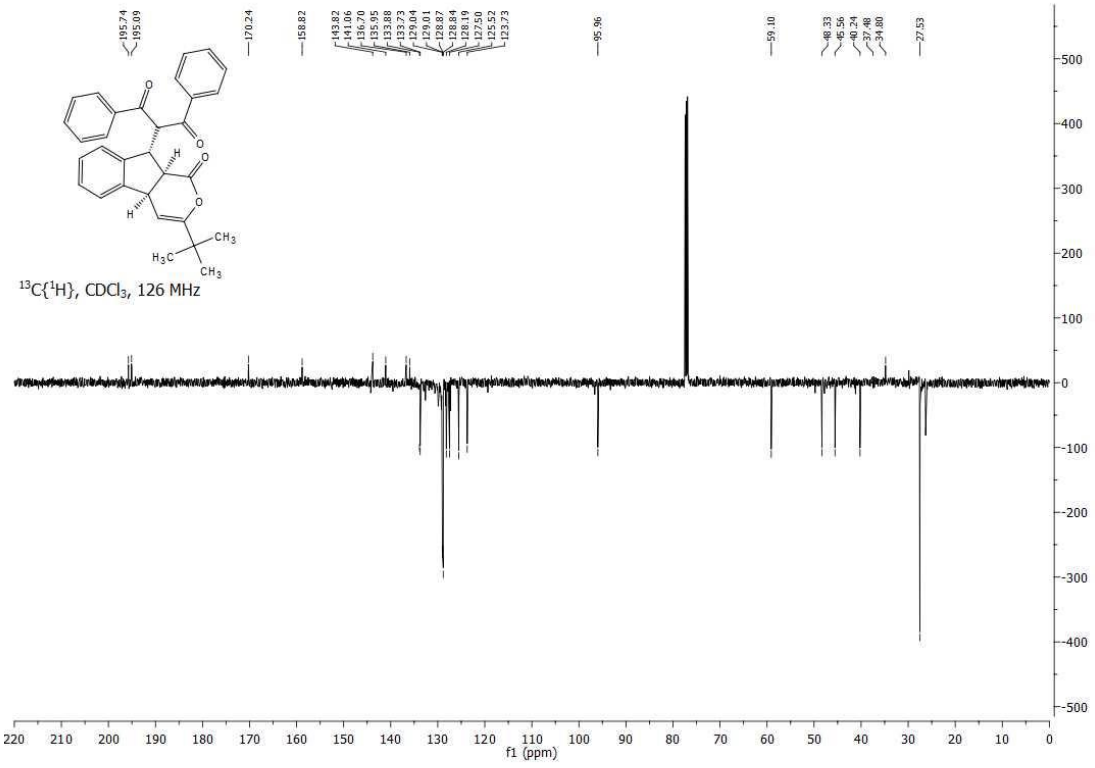

2-((4a*S*,9*S*,9a*R*)-1-Oxo-3-phenyl-1,4a,9,9a-tetrahydroindeno[2,1-*c*]pyran-9-yl)-1,3-diphenylpropane-1,3-dione (21a)

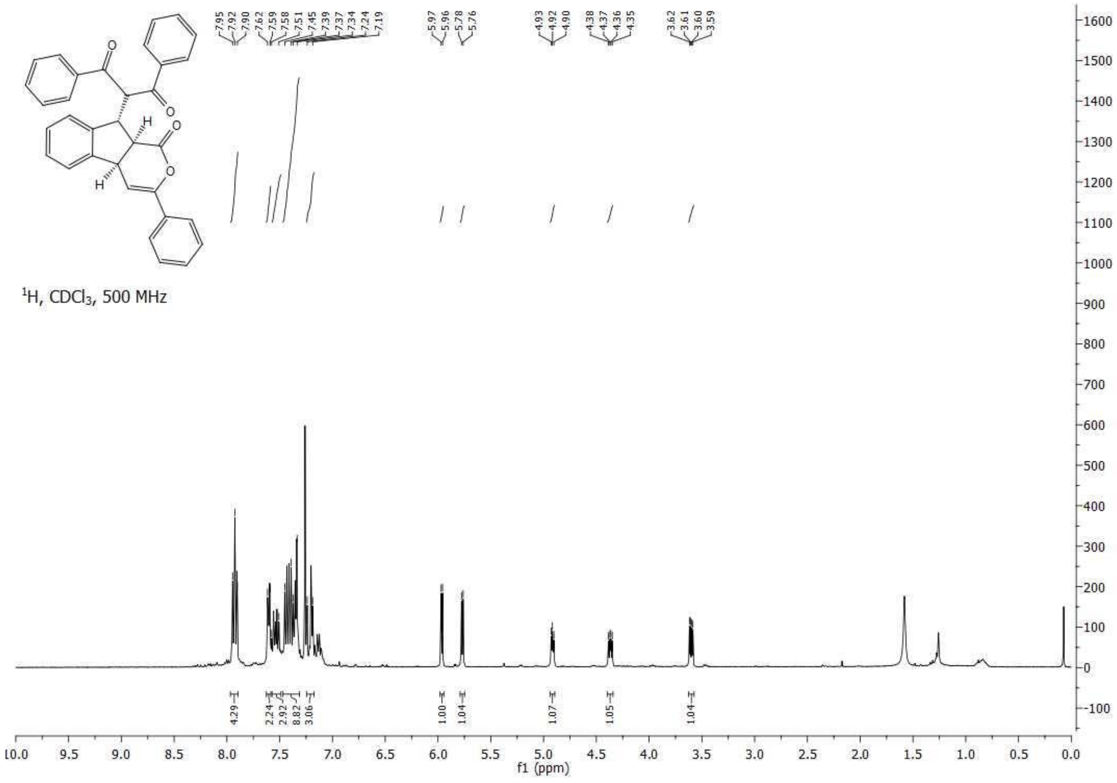

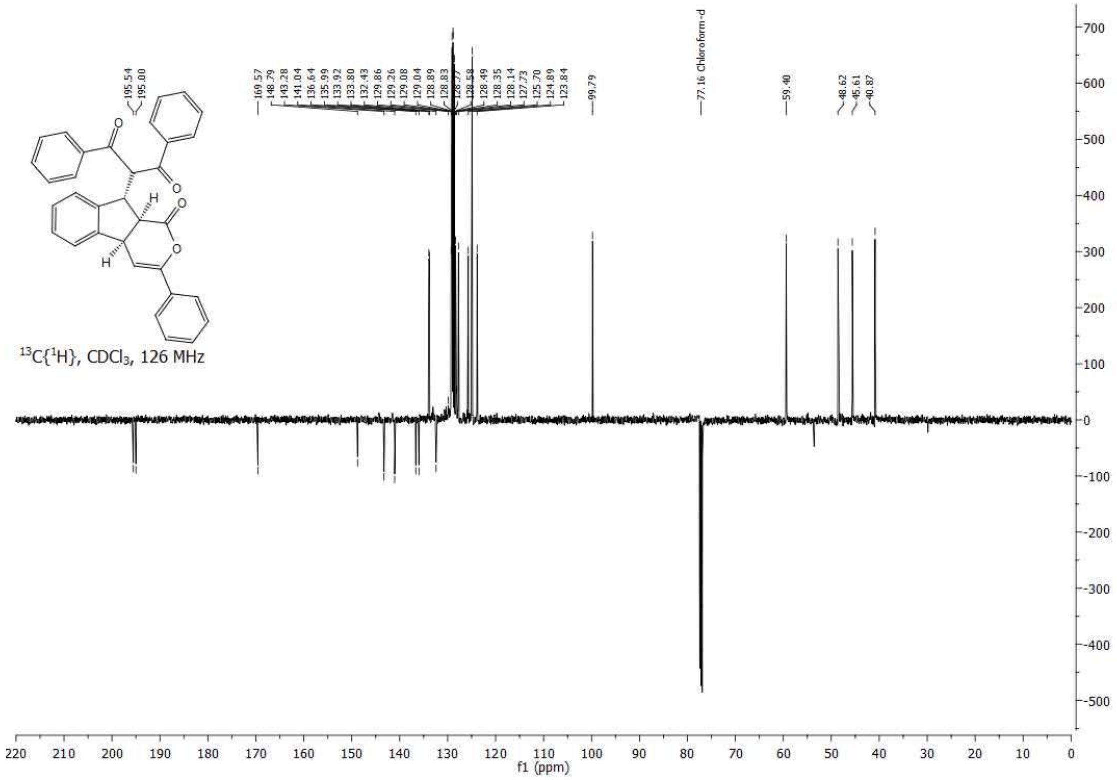

2-((4a*R*,9*R*,9a*S*)-3-(4-Methoxyphenyl)-1-oxo-1,4a,9,9a-tetrahydroindeno[2,1-*c*]pyran-9-yl)-1,3-diphenylpropane-1,3-dione (22a)

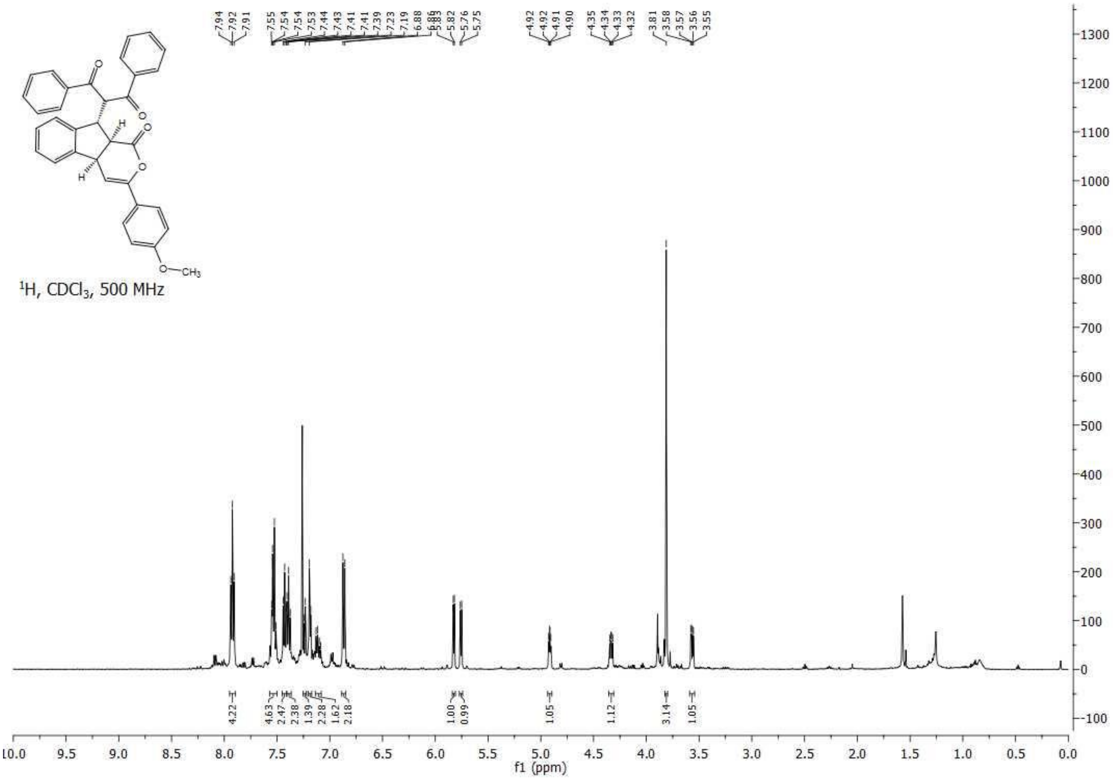

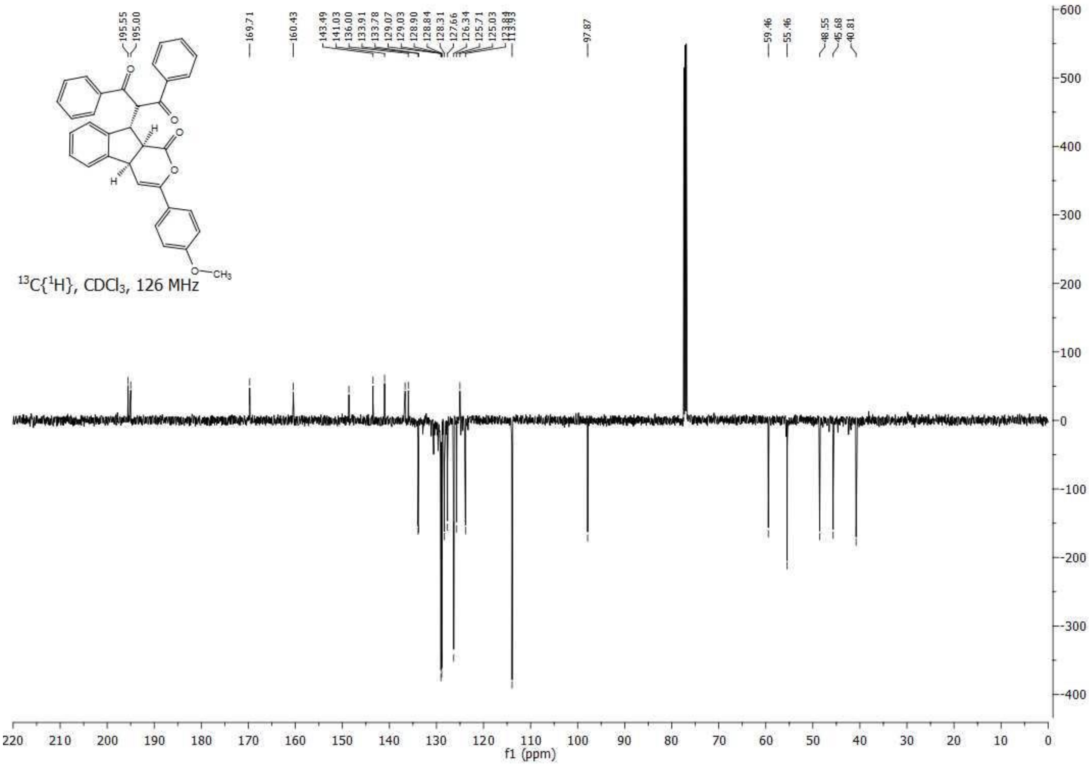

2-((4a*R*,9*R*,9a*S*)-1-Oxo-3-(4-(trifluoromethyl)phenyl)-1,4a,9,9a-tetrahydroindeno[2,1-*c*]pyran-9-yl)-1,3-diphenylpropane-1,3-dione (23a)

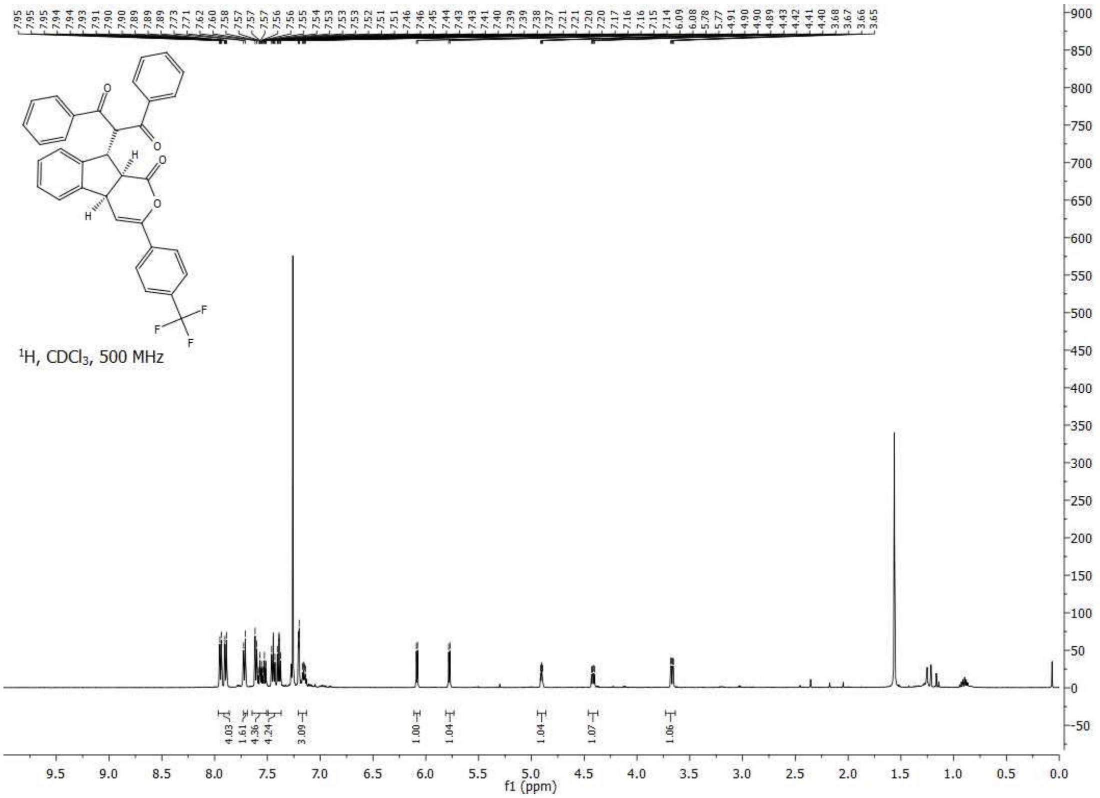

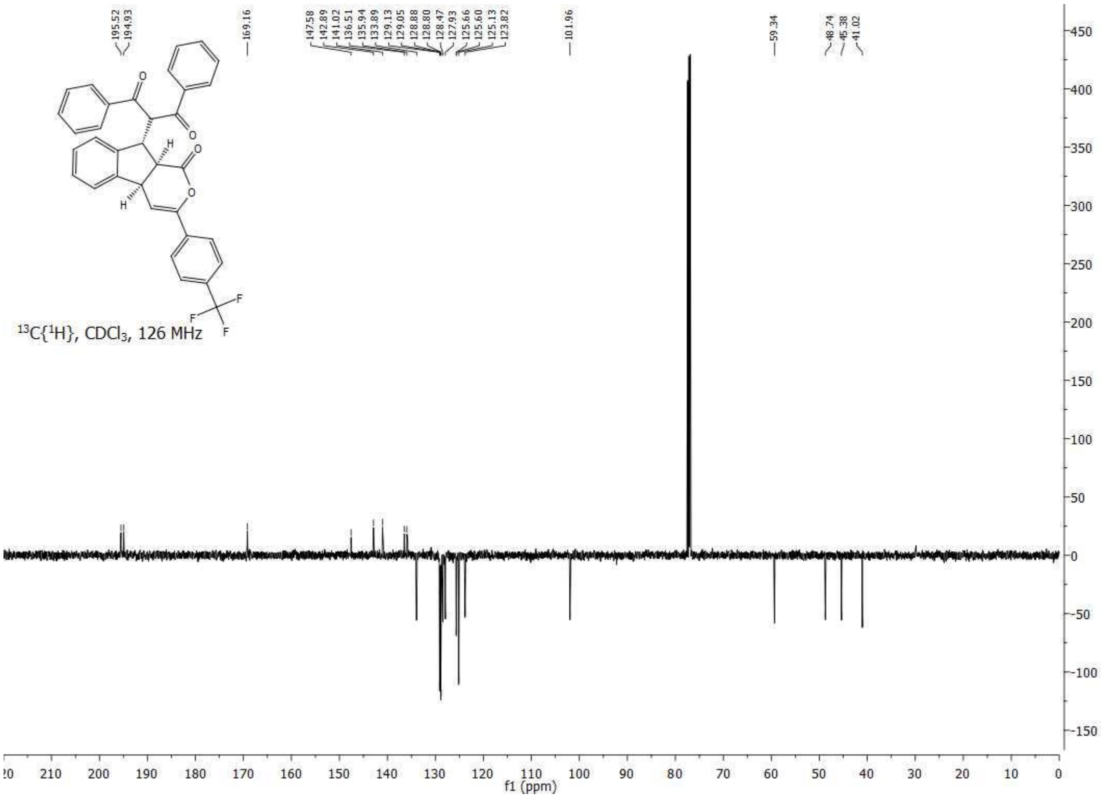

2-((4a*R*,9*R*,9a*S*)-3-(4-Bromophenyl)-1-oxo-1,4a,9,9a-tetrahydroindeno[2,1-*c*]pyran-9-yl)-1,3-diphenylpropane-1,3-dione (24a)

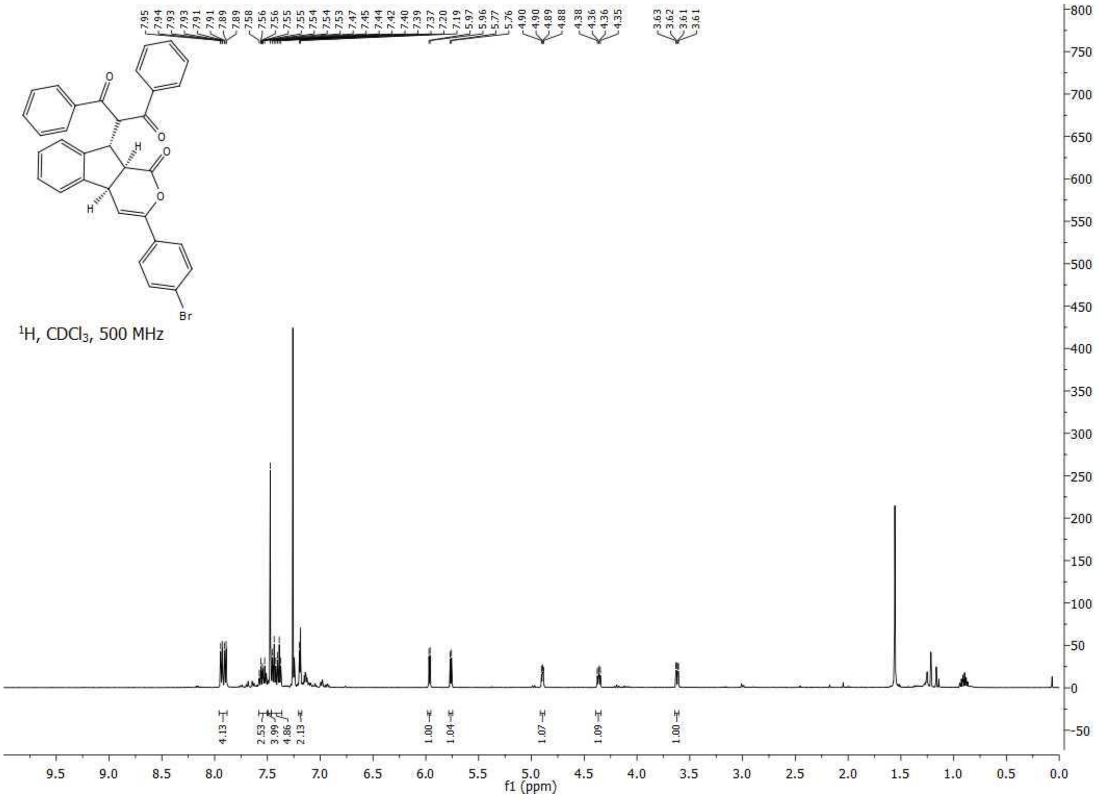

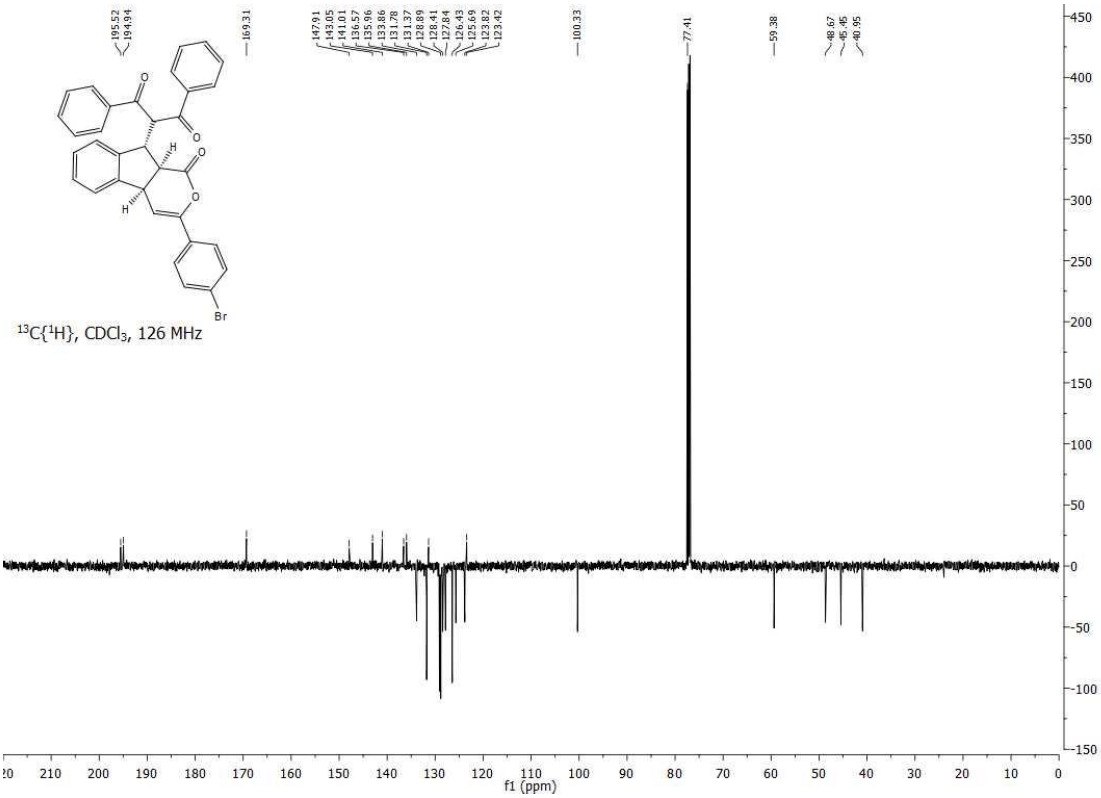

2-((4a*R*,9*R*,9a*S*)-3-(4-Chlorophenyl)-1-oxo-1,4a,9,9a-tetrahydroindeno[2,1-*c*]pyran-9-yl)-1,3-diphenylpropane-1,3-dione (25a)

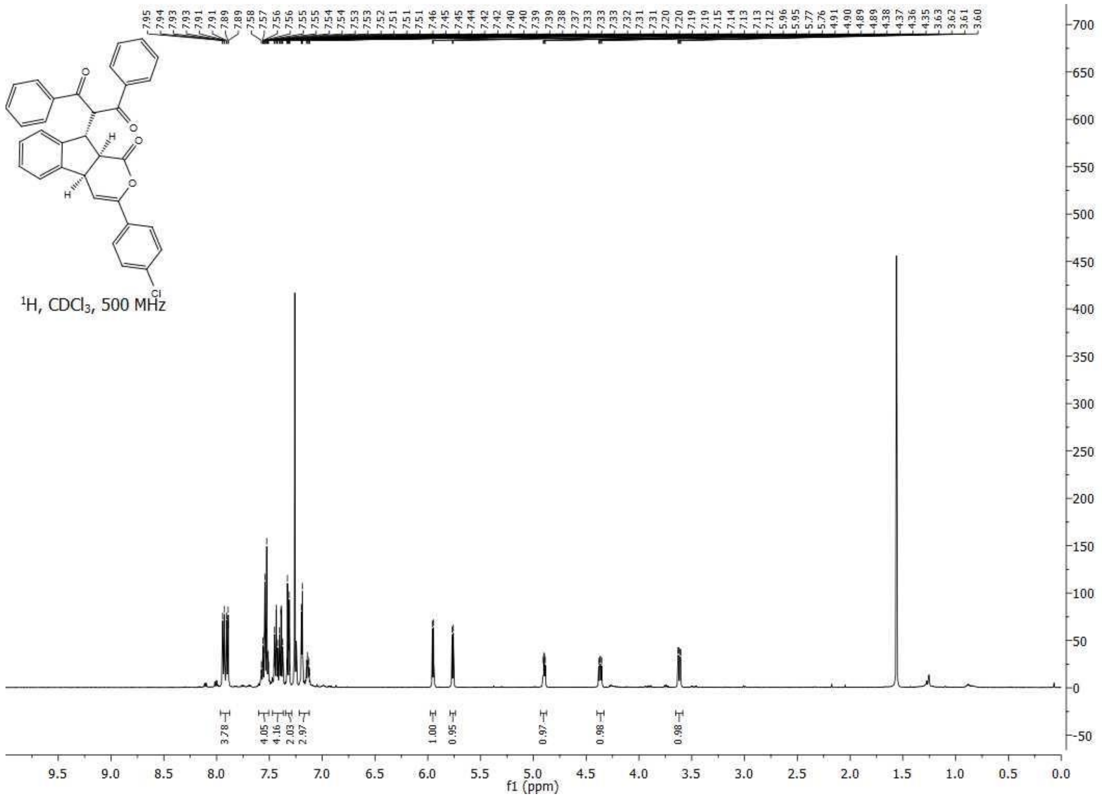

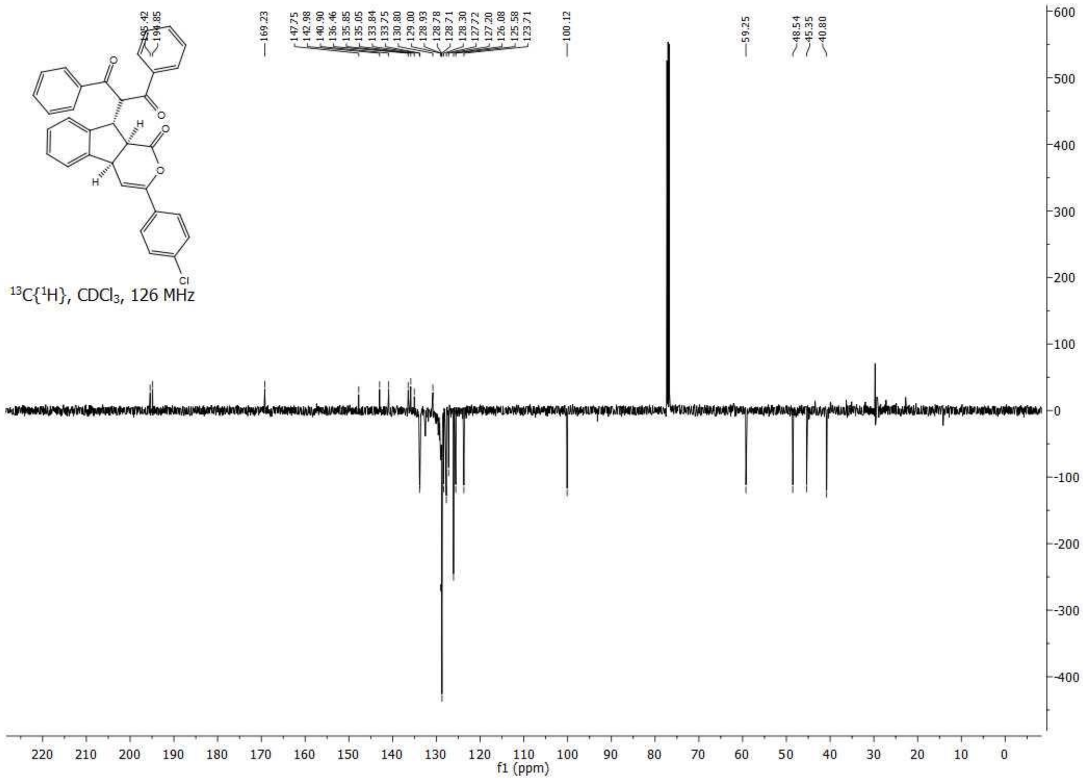

2-((4a*R*,9*R*,9a*S*)-6-Chloro-3-methyl-1-oxo-1,4a,9,9a-tetrahydroindeno[2,1-*c*]pyran-9-yl)-1,3-bis(4-methoxyphenyl)propane-1,3-dione (26a)

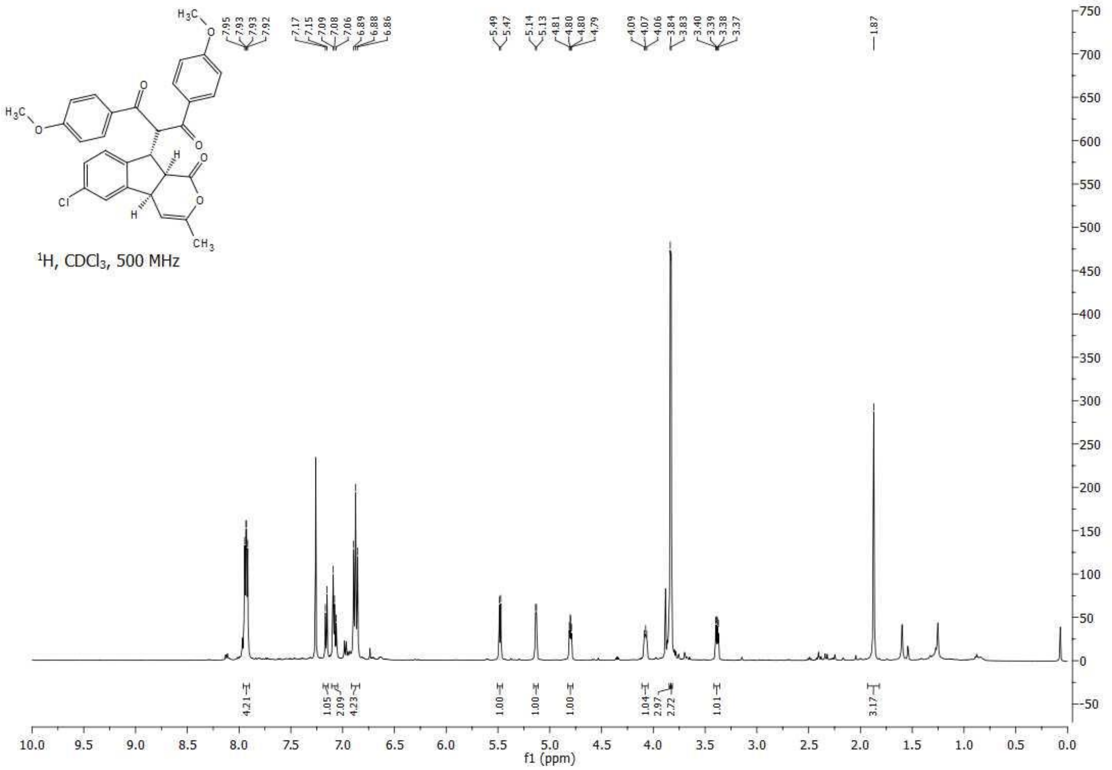

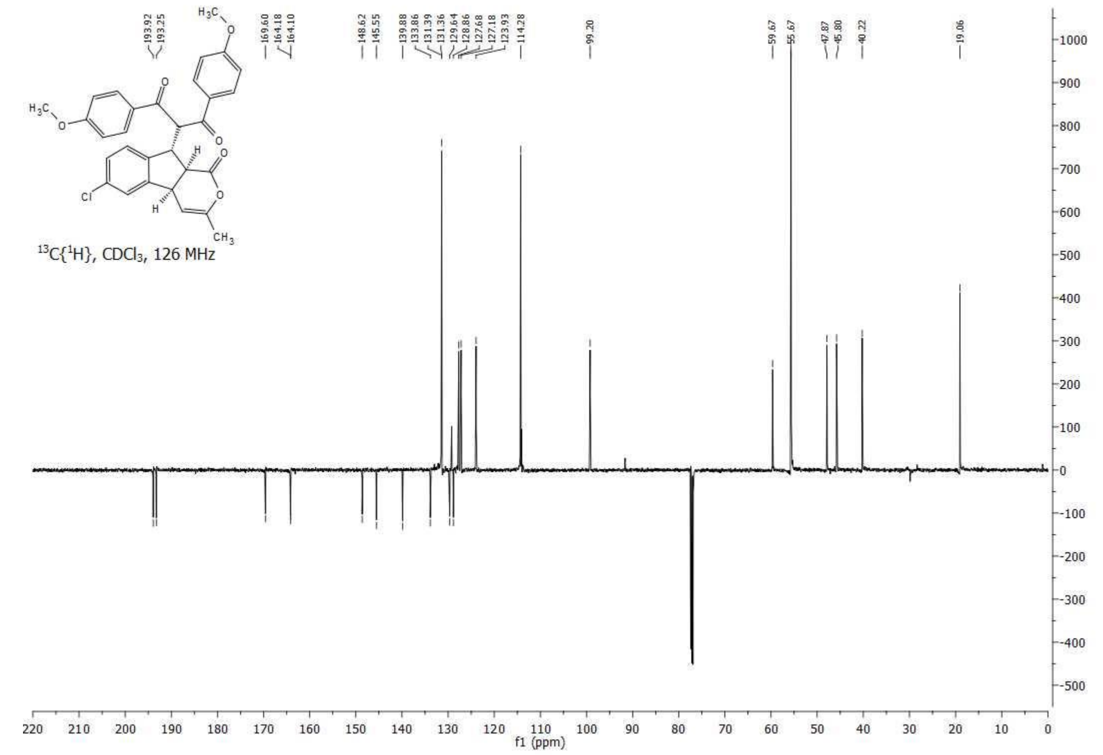

1,3-Bis(4-fluorophenyl)-2-((4a*R*,9*R*,9a*S*)-3-methyl-1-oxo-1,4a,9,9a-tetrahydroindeno[2,1-*c*]pyran-9-yl)propane-1,3-dione (27a)

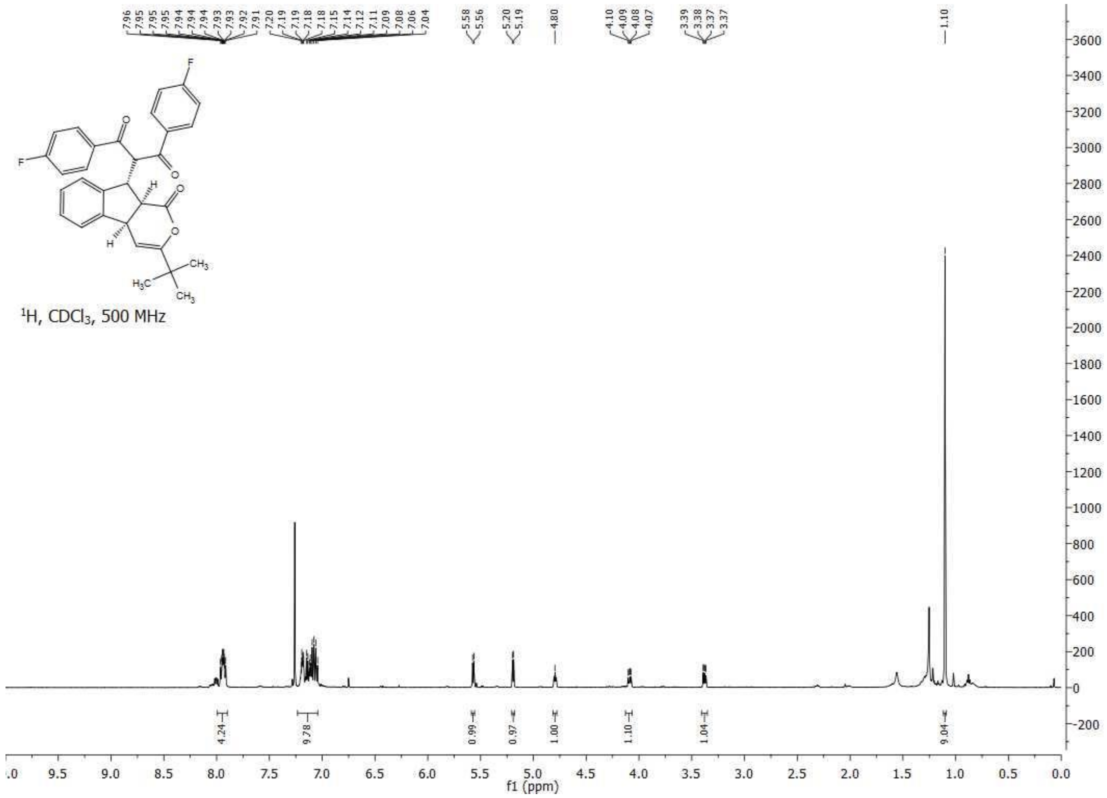

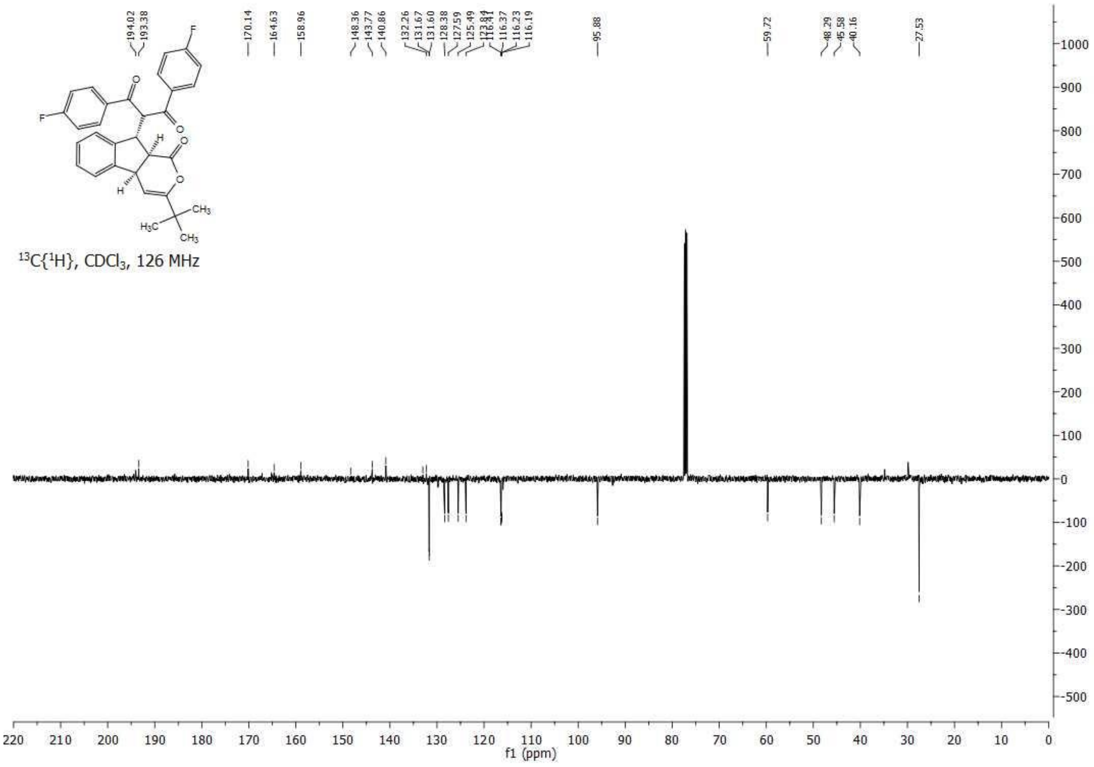

2-((4a*R*,9*R*,9a*S*)-6-Chloro-3-methyl-1-oxo-1,4a,9,9a-tetrahydroindeno[2,1-*c*]pyran-9-yl)-1,3-di(furan-2-yl)propane-1,3-dione (28a)

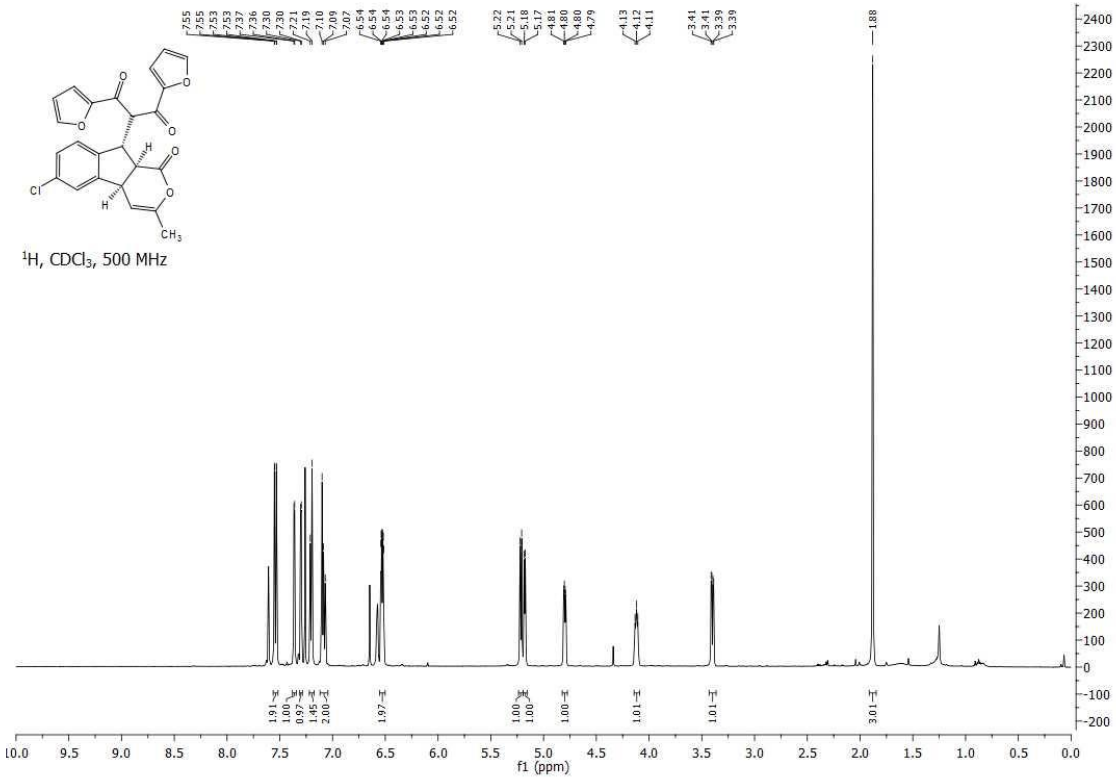

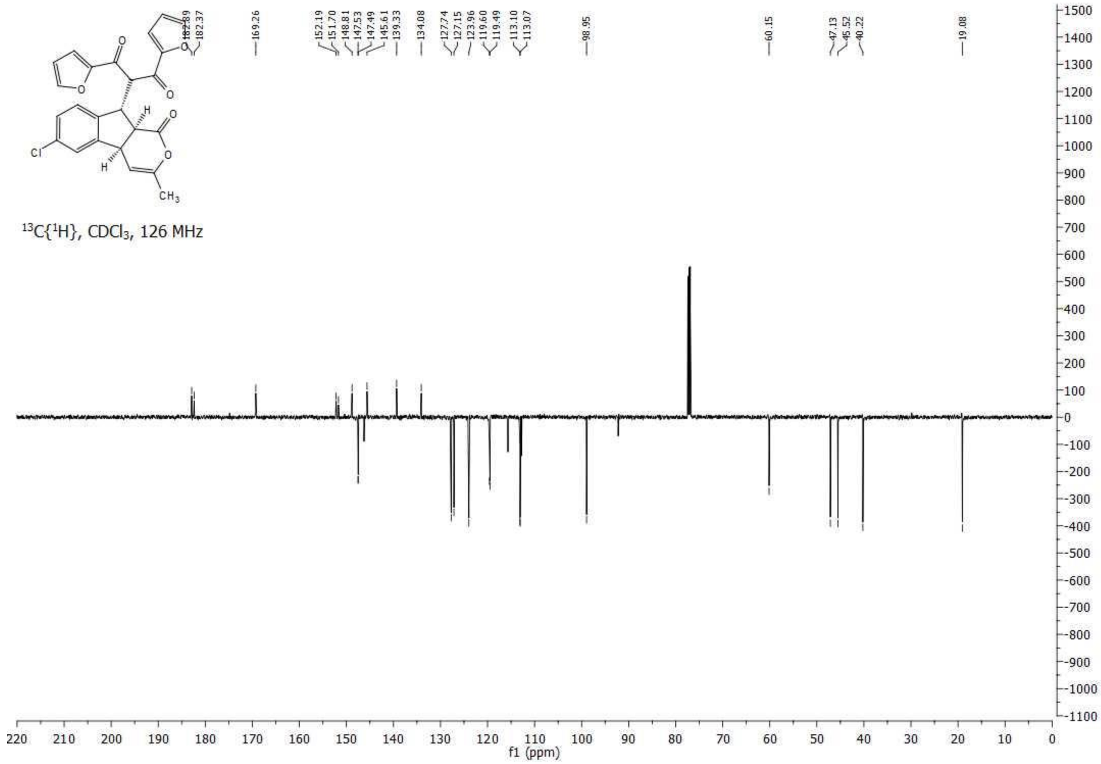

(6a*R*,11*S*,11a*R*)-6-Benzoyl-11-(2-oxopropyl)-11,11a-dihydrobenzo[4,5]thiazolo[3,2-*a*]indeno[1,2-*d*]pyridin-12(6a*H*)-one (35a)

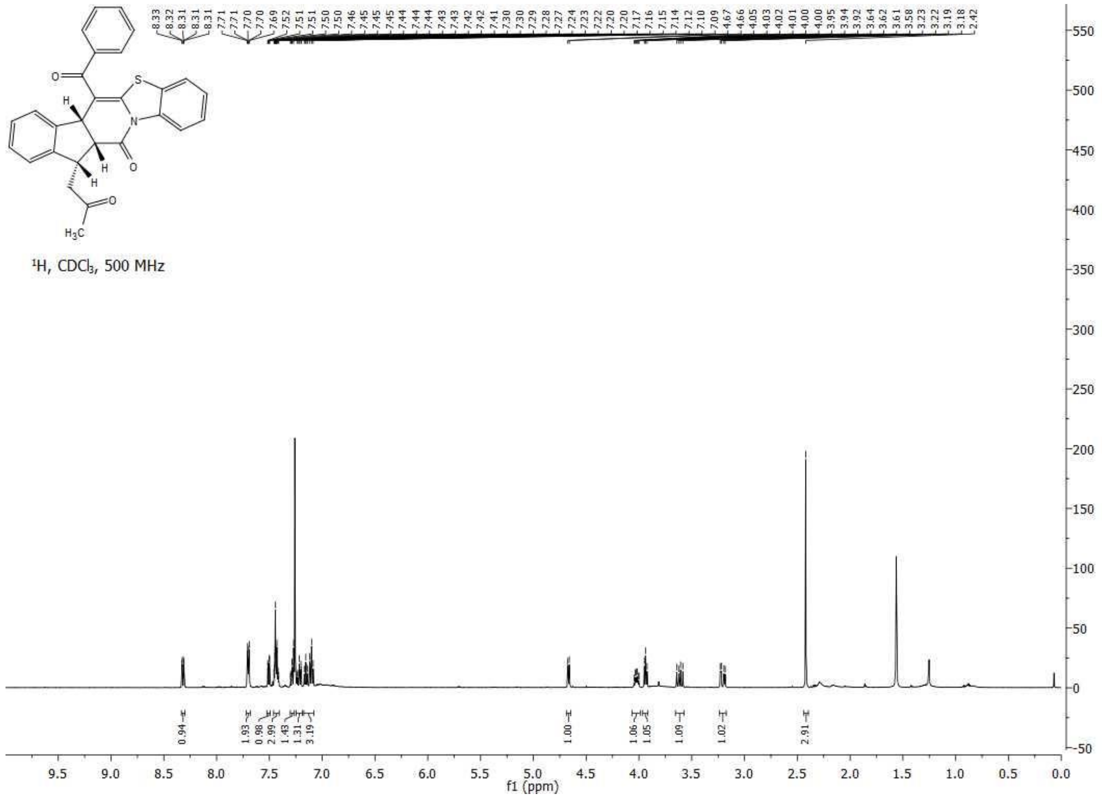

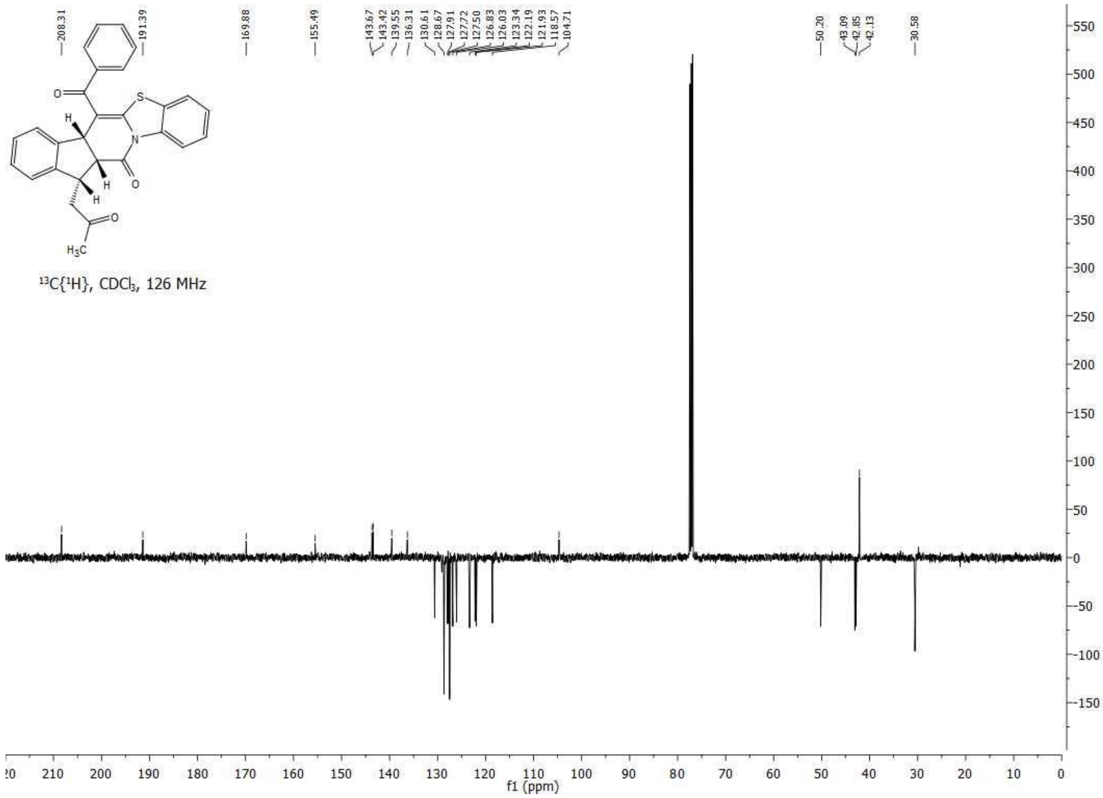

(6a*R*,11*S*,11a*R*)-6-Benzoyl-8-methyl-11-(2-oxopropyl)-11,11a-dihydrobenzo[4,5]thiazolo[3,2-*a*]indeno[1,2-*d*]pyridin-12(6a*H*)-one (36a)

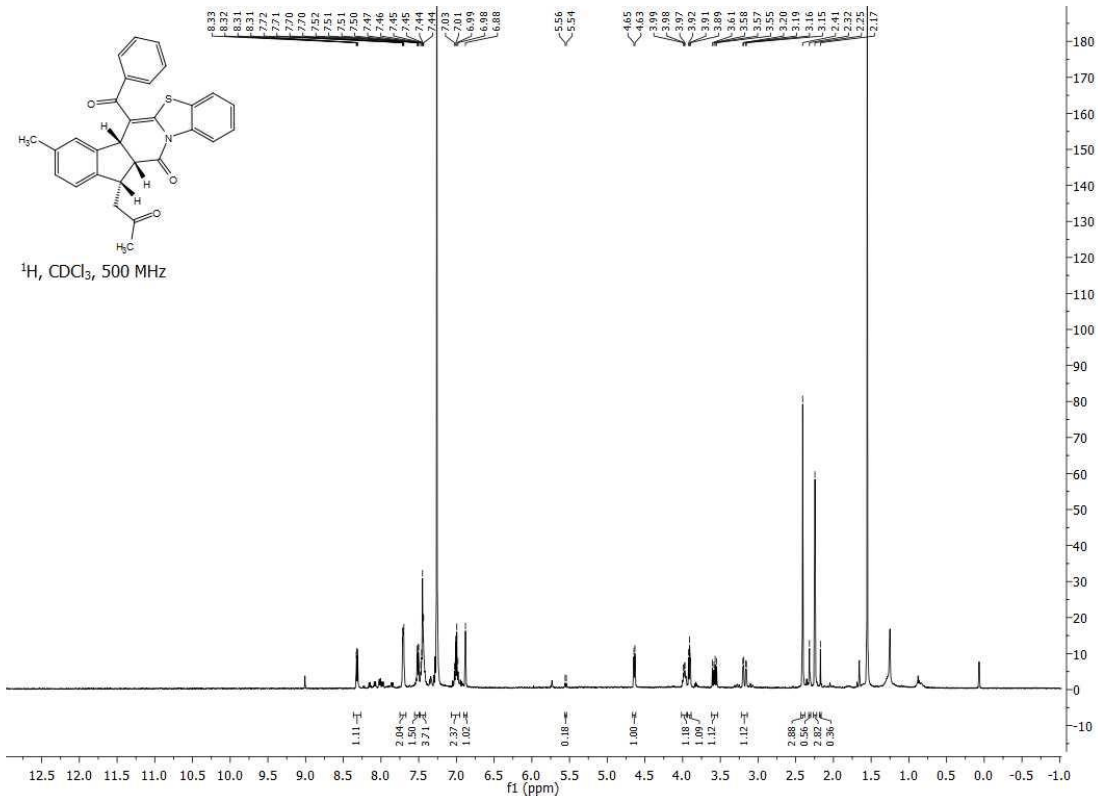

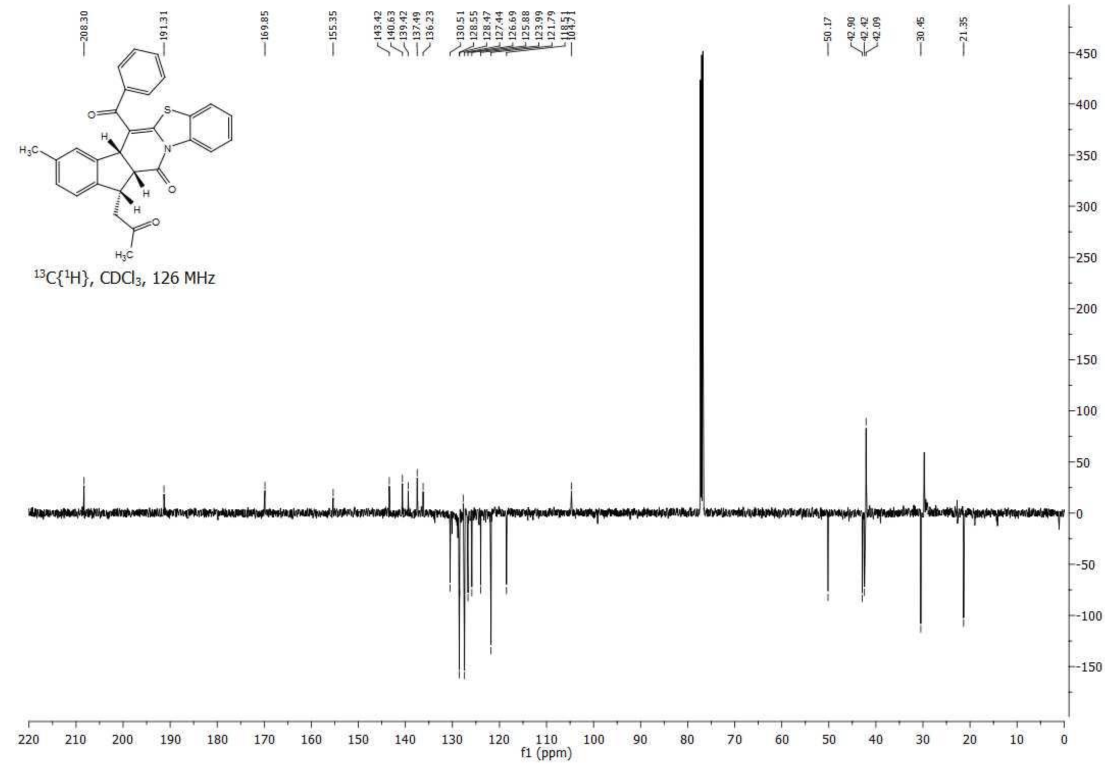

(6a*R*,11*S*,11a*R*)-6-Benzoyl-9-fluoro-11-(2-oxopropyl)-11,11a-dihydrobenzo[4,5]thiazolo[3,2-*a*]indeno[1,2-*d*]pyridin-12(6a*H*)-one (37a)

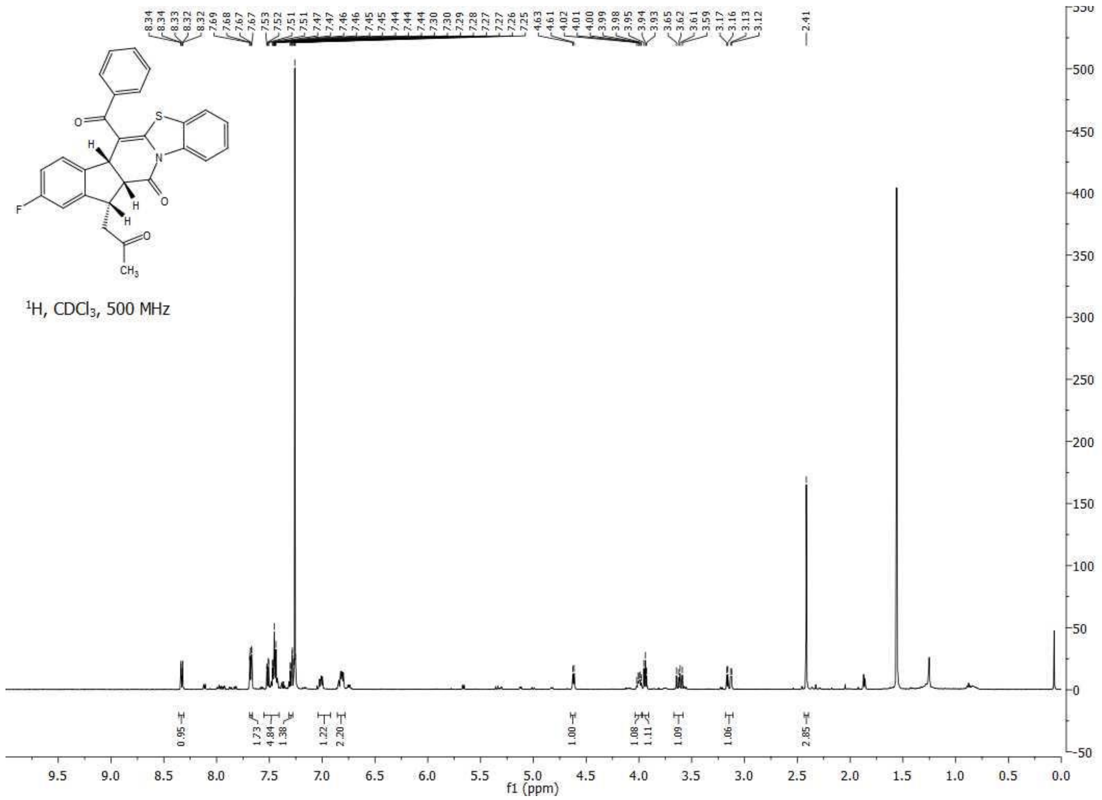

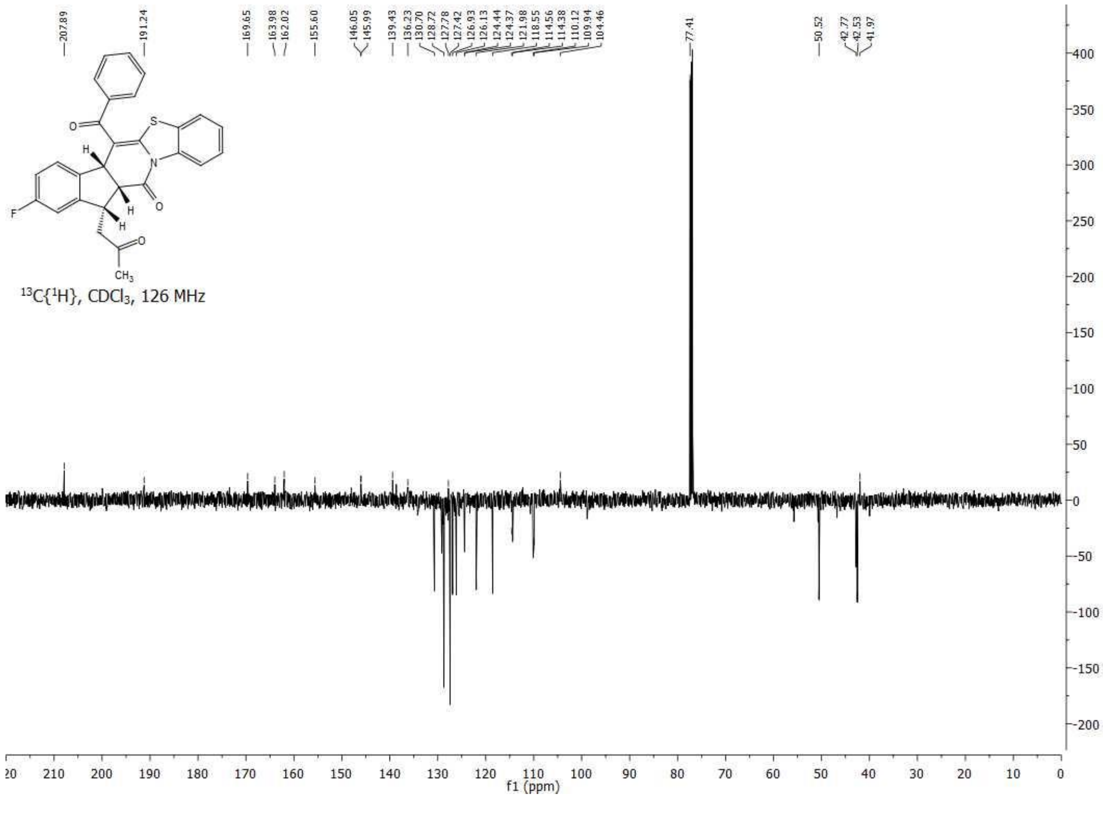

(6a*R*,11*S*,11a*R*)-6-Benzoyl-11-(2-oxo-2-phenylethyl)-11,11a-dihydrobenzo[4,5]thiazolo[3,2-*a*]indeno[1,2-*d*]pyridin-12(6a*H*)-one (38a)

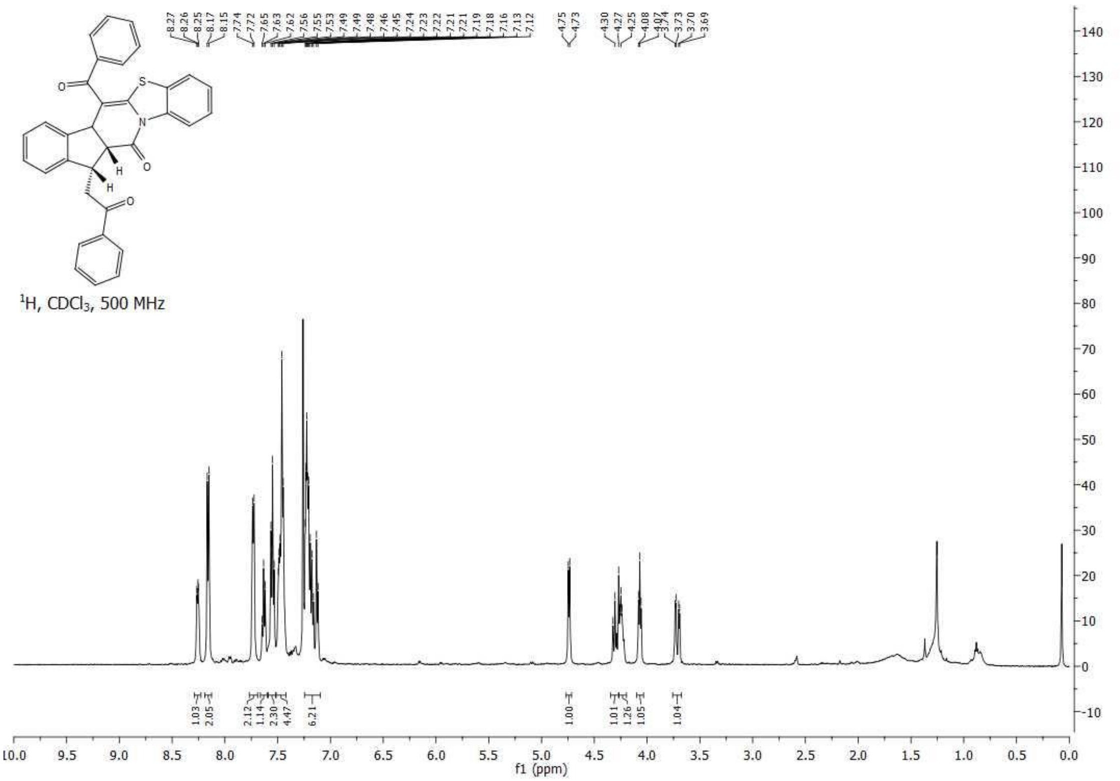

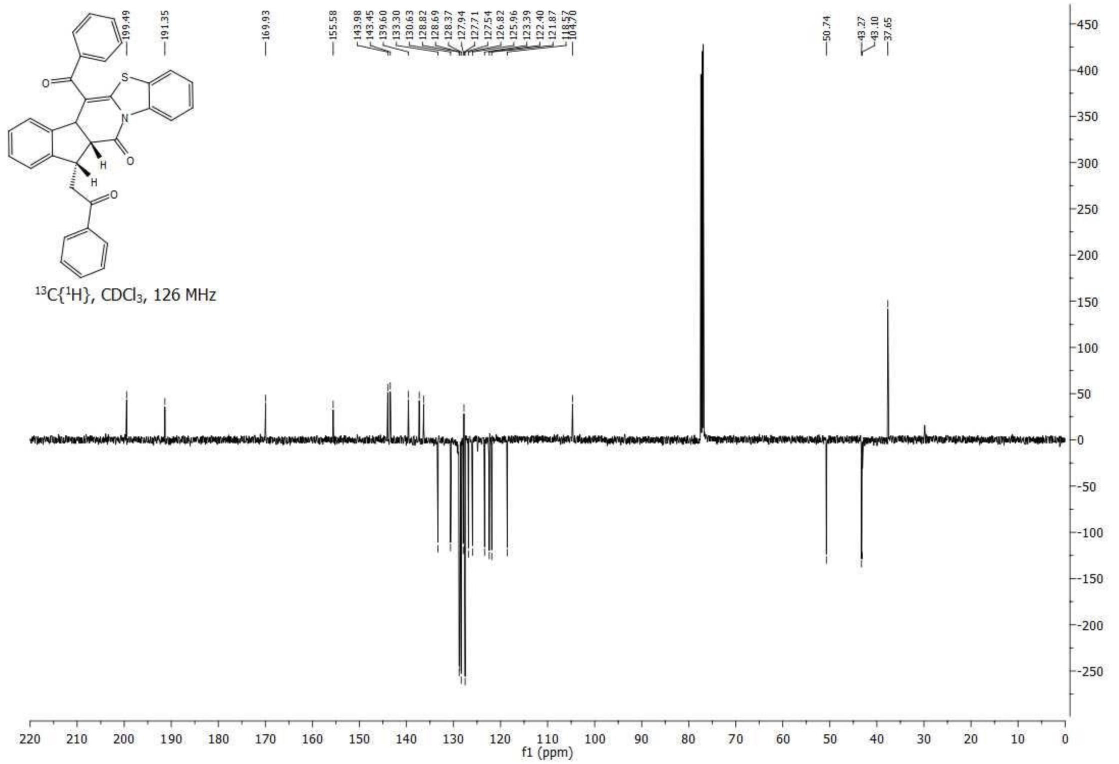

(6a*R*,11*S*,11a*R*)-6-Benzoyl-3-fluoro-11-(2-oxo-2-phenylethyl)-11,11a-dihydrobenzo[4,5]thiazolo-[3,2-*a*]indeno[1,2-*d*]pyridin-12(6a*H*)-one (39a)

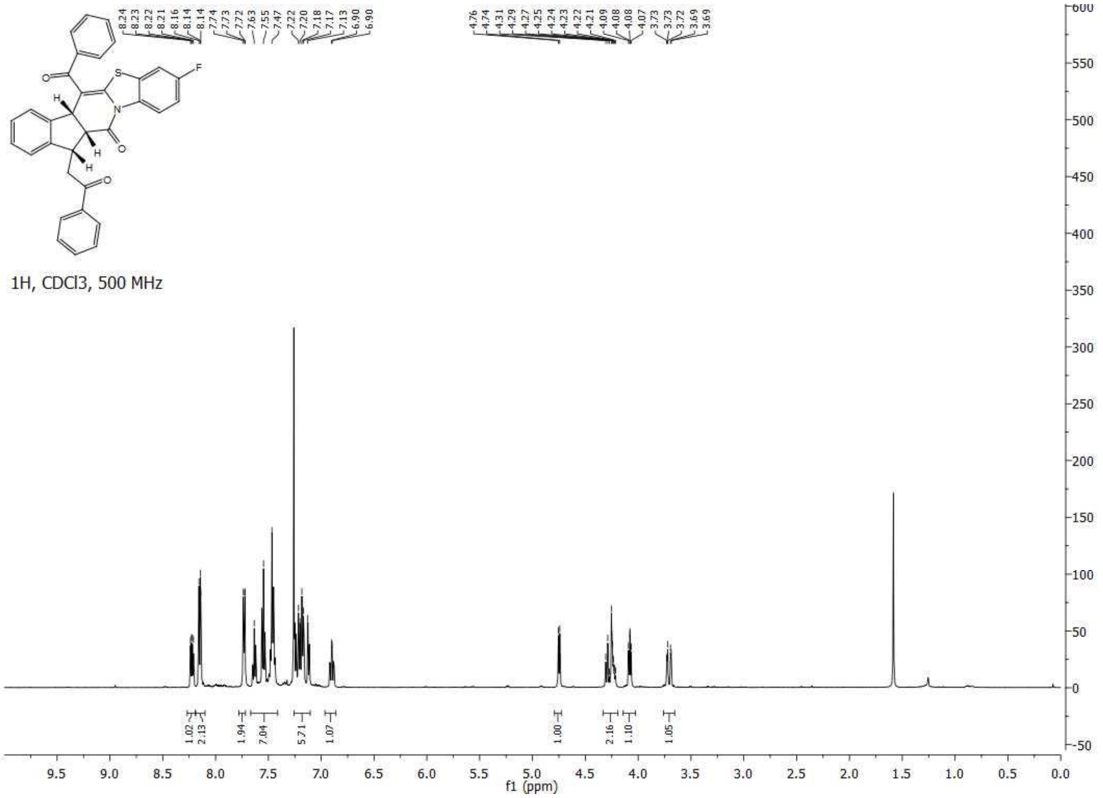

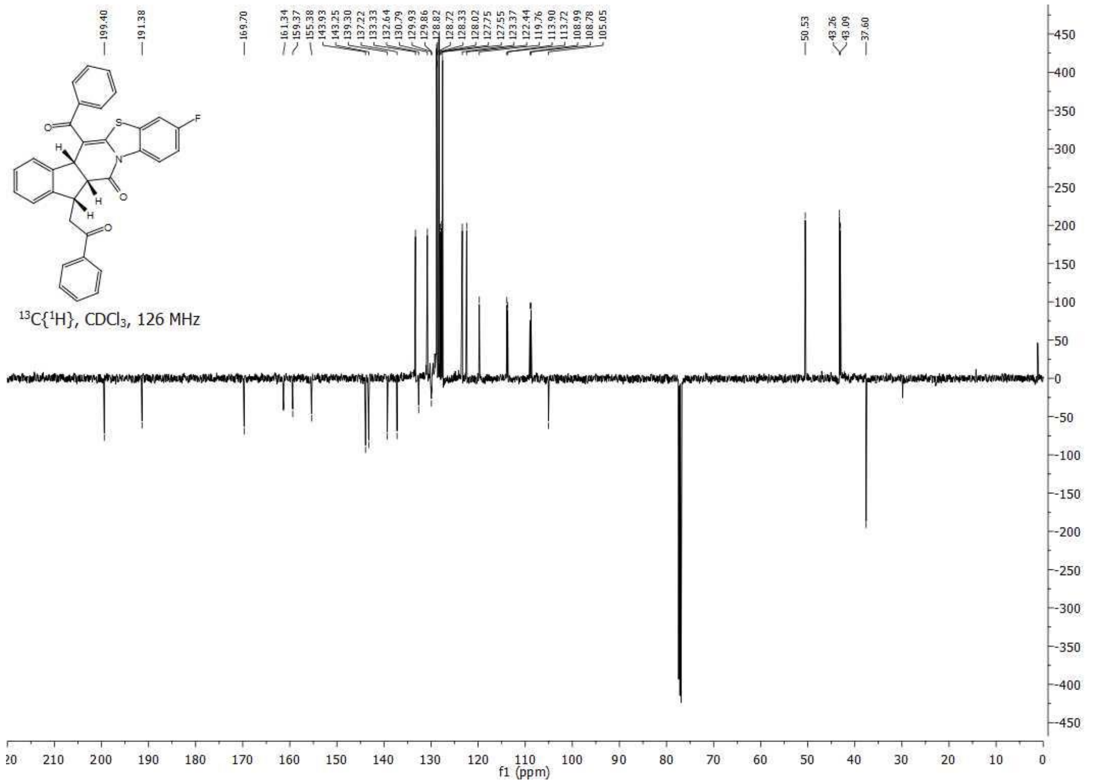

(6a*R*,11*S*,11a*R*)-6-Benzoyl-3-bromo-11-(2-oxo-2-phenylethyl)-11,11a-dihydrobenzo[4,5]thiazolo-[3,2-*a*]indeno[1,2-*d*]pyridin-12(6a*H*)-one (40a)

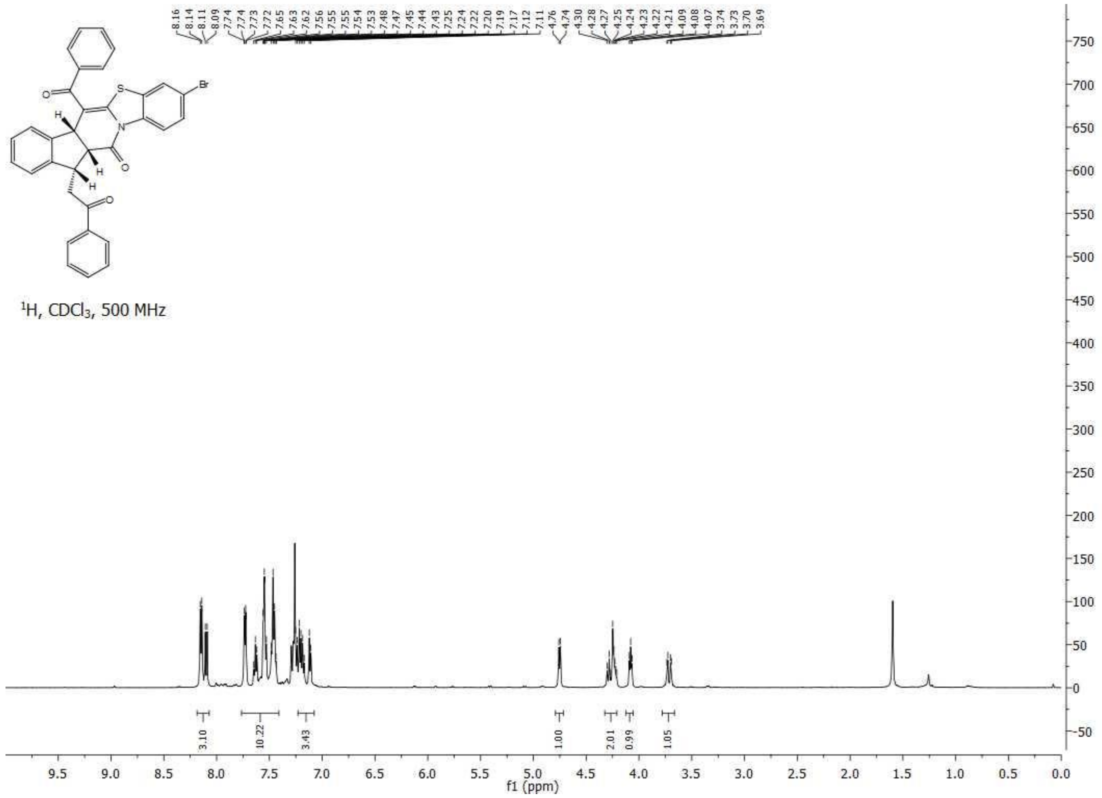

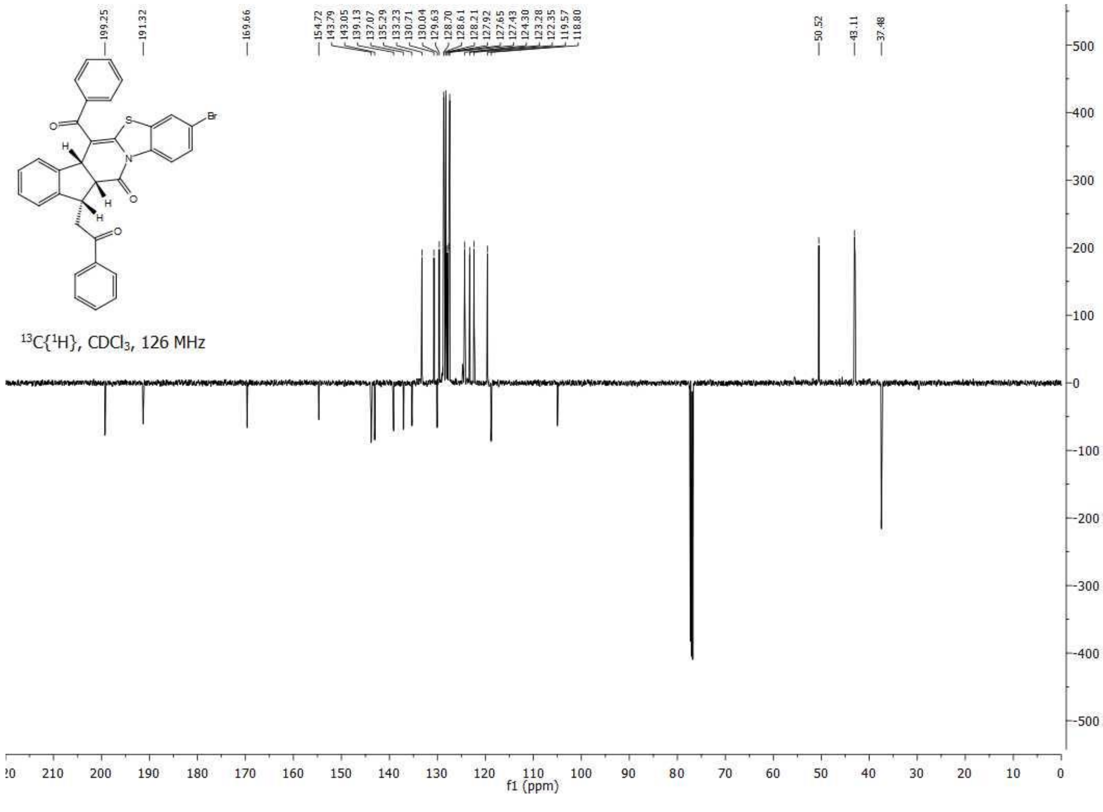

(6a*R*,11*S*,11a*R*)-6-Benzoyl-3-methoxy-11-(2-oxo-2-phenylethyl)-11,11a-dihydrobenzo[4,5]thiazolo-[3,2-*a*]indeno[1,2-*d*]pyridin-12(6a*H*)-one (41a)

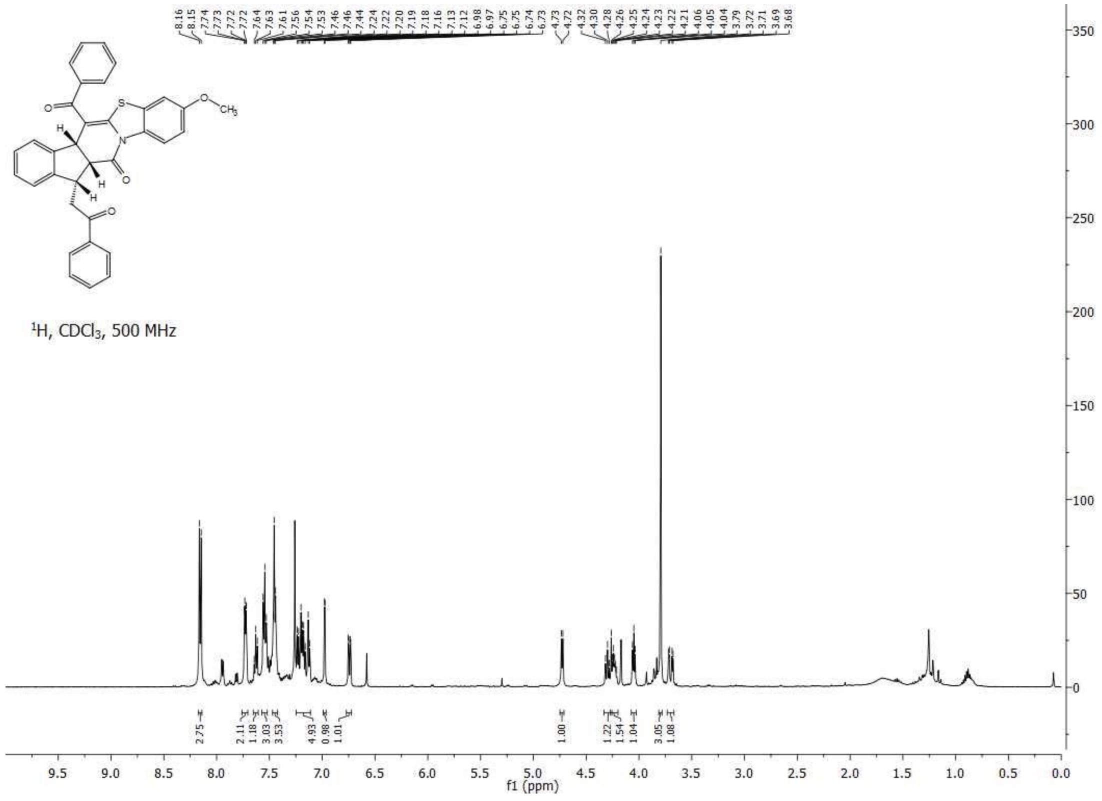

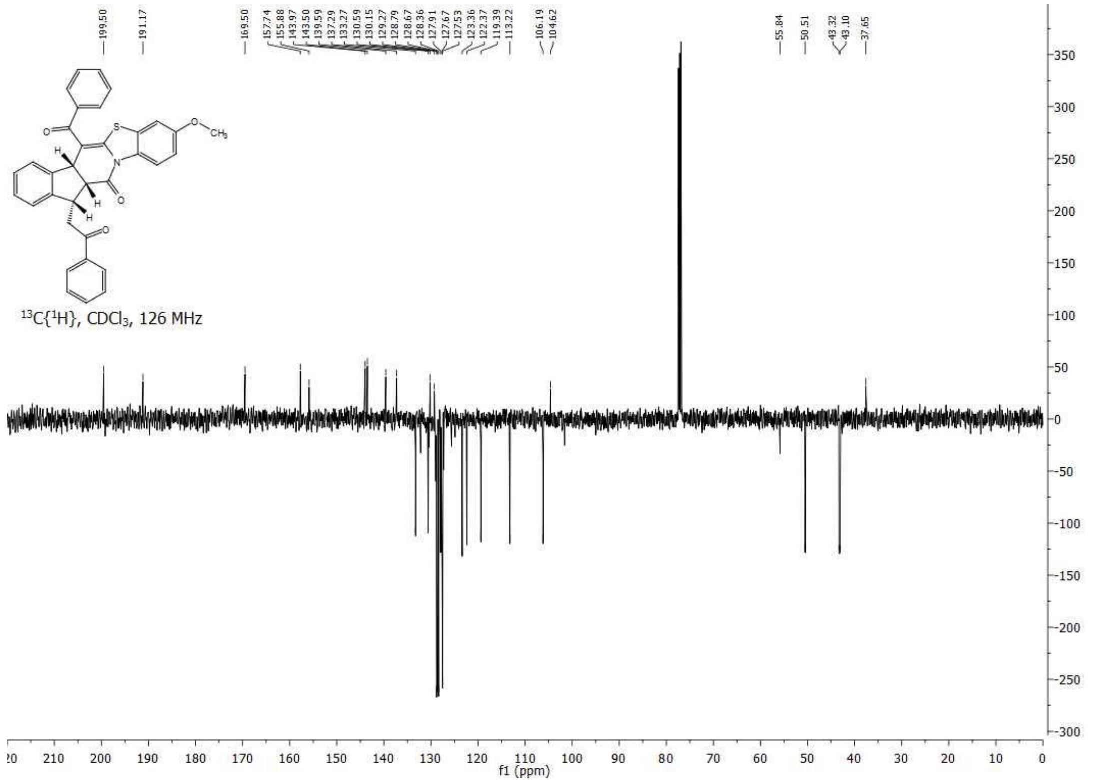

(6a*R*,11*S*,11a*R*)-6-(4-Fluorobenzoyl)-11-(2-oxo-2-phenylethyl)-11,11a-dihydrobenzo[4,5]thiazolo-[3,2-*a*]indeno[1,2-*d*]pyridin-12(6a*H*)-one (42a)

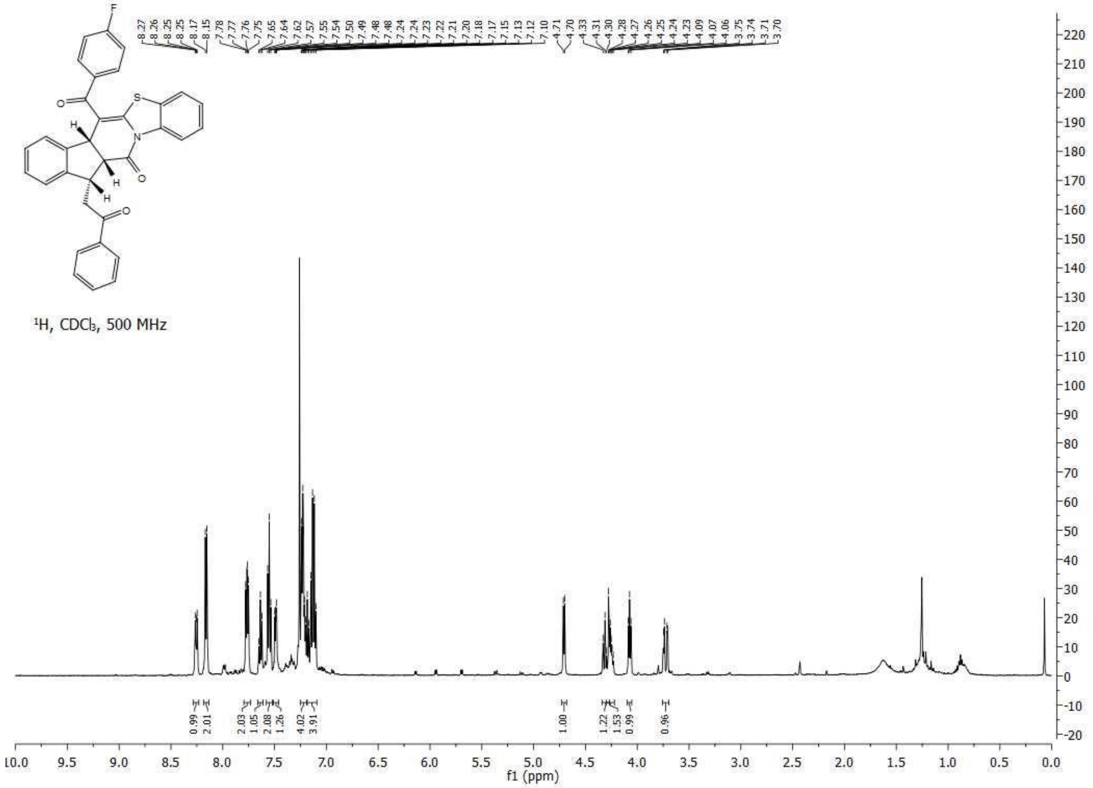

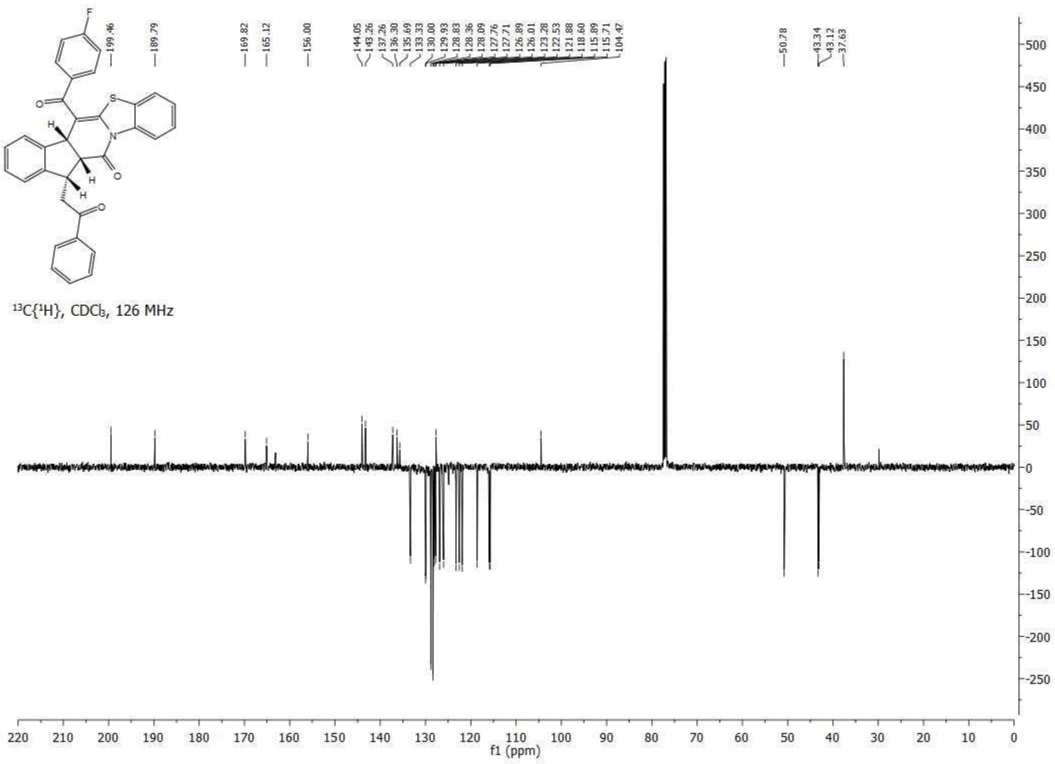

(6a*R*,11*S*,11a*R*)-6-(4-Bromobenzoyl)-11-(2-oxo-2-phenylethyl)-11,11a-dihydrobenzo[4,5]thiazolo-[3,2-*a*]indeno[1,2-*d*]pyridin-12(6a*H*)-one (43a)

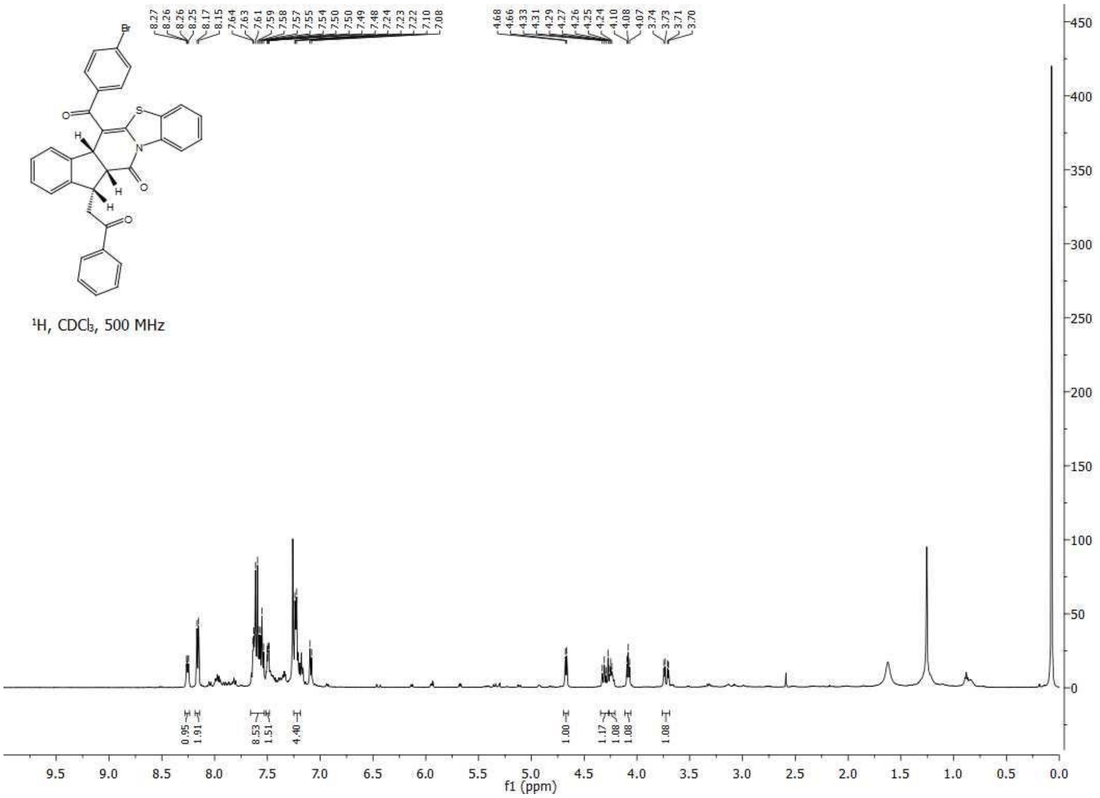

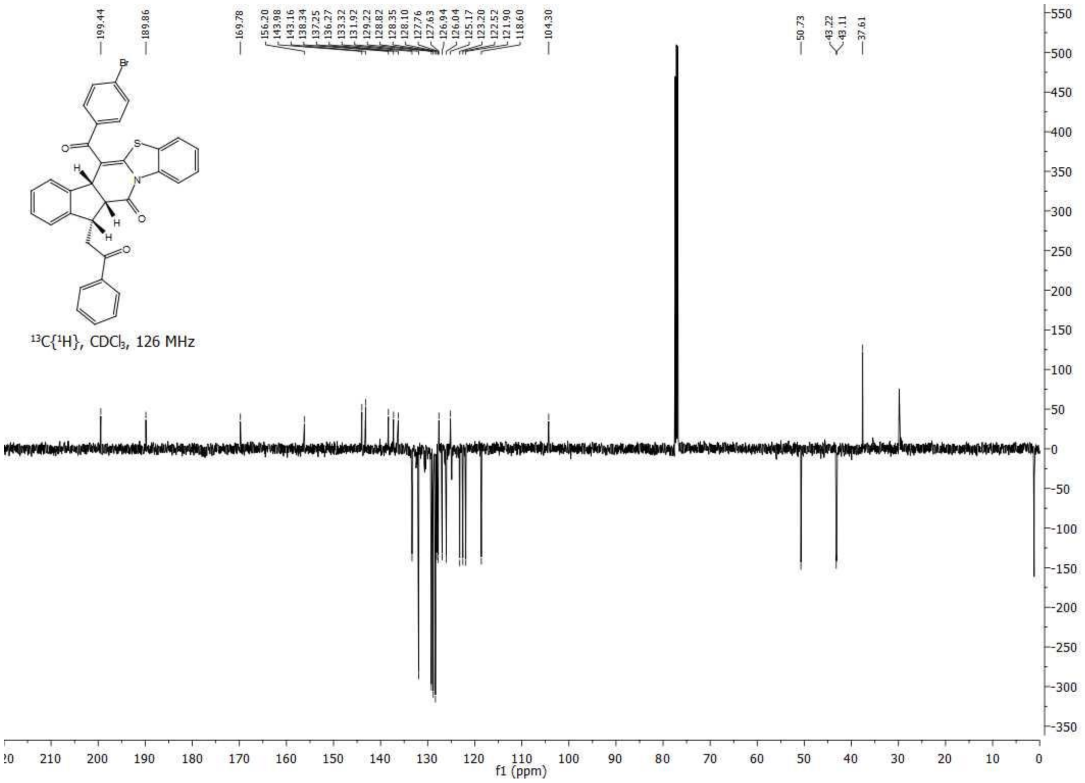

(6a*R*,11*S*,11a*R*)-6-(4-Methoxybenzoyl)-11-(2-oxo-2-phenylethyl)-11,11a-dihydrobenzo[4,5]-thiazolo[3,2-*a*]indeno[1,2-*d*]pyridin-12(6a*H*)-one (44a)

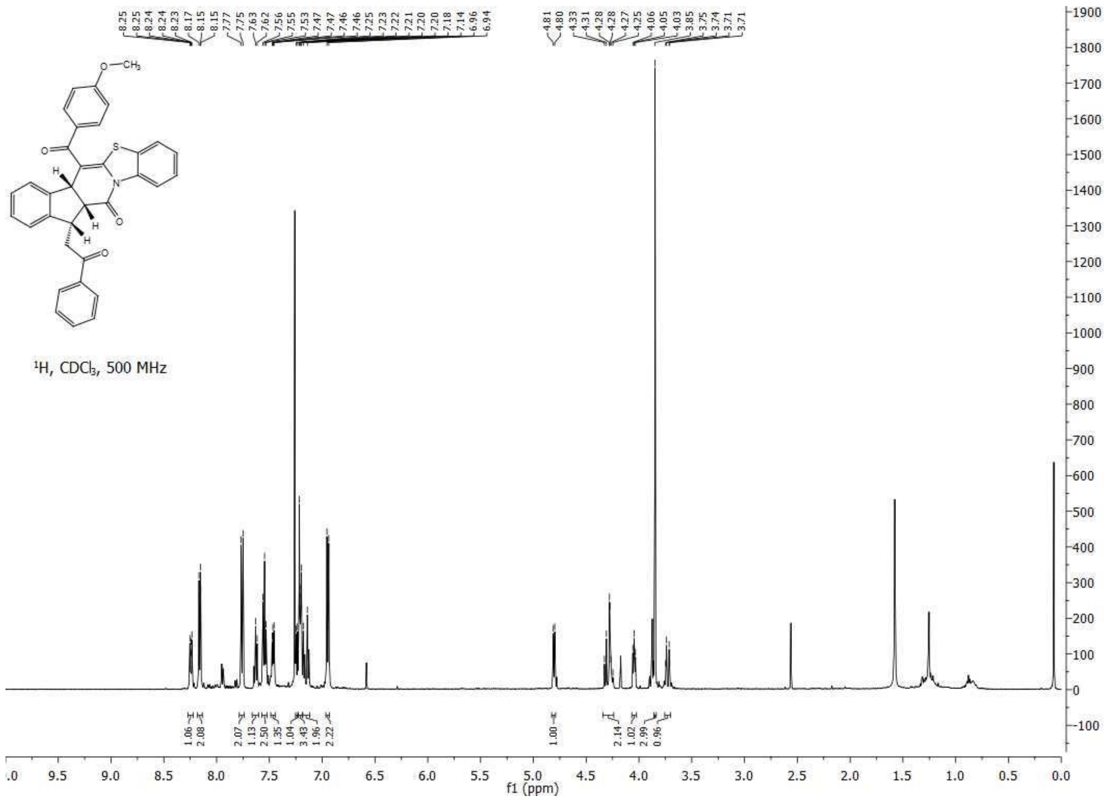

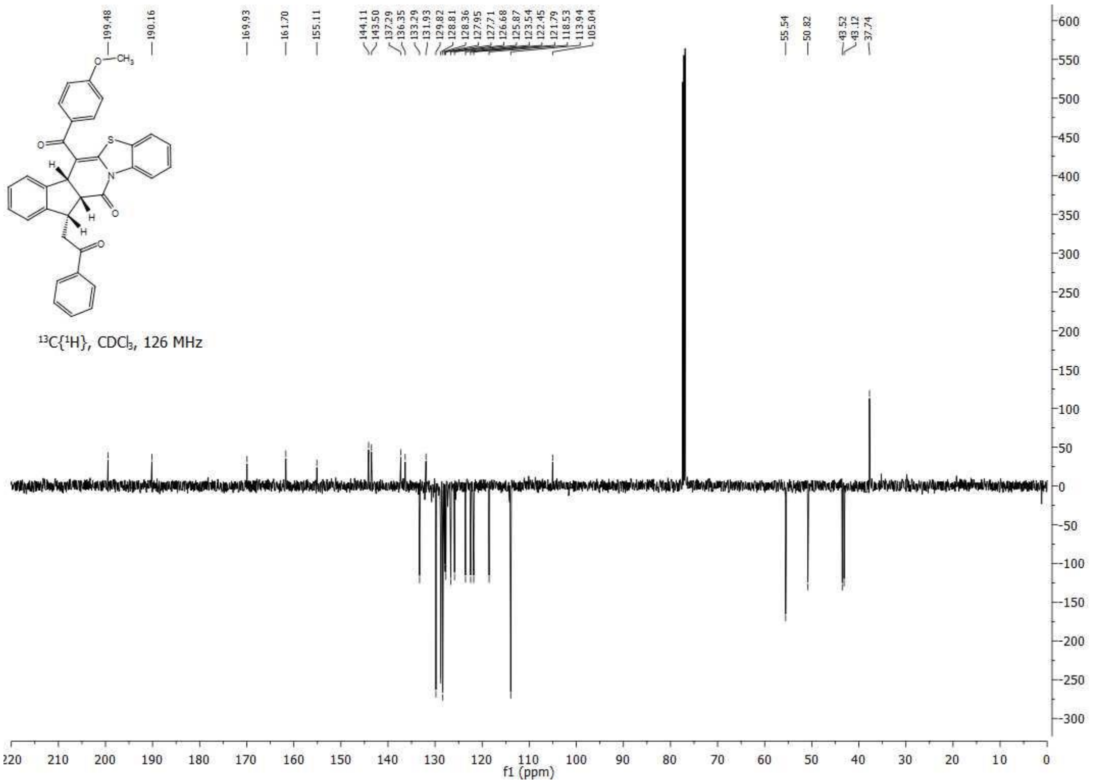

(R,*E*)-4-(4-Fluorobenzoyl)-3-(2-(3-oxobut-1-en-1-yl)phenyl)-2,3-dihydro-1H-benzo[4,5]thiazolo[3,2-*a*]pyridin-1-one (51)

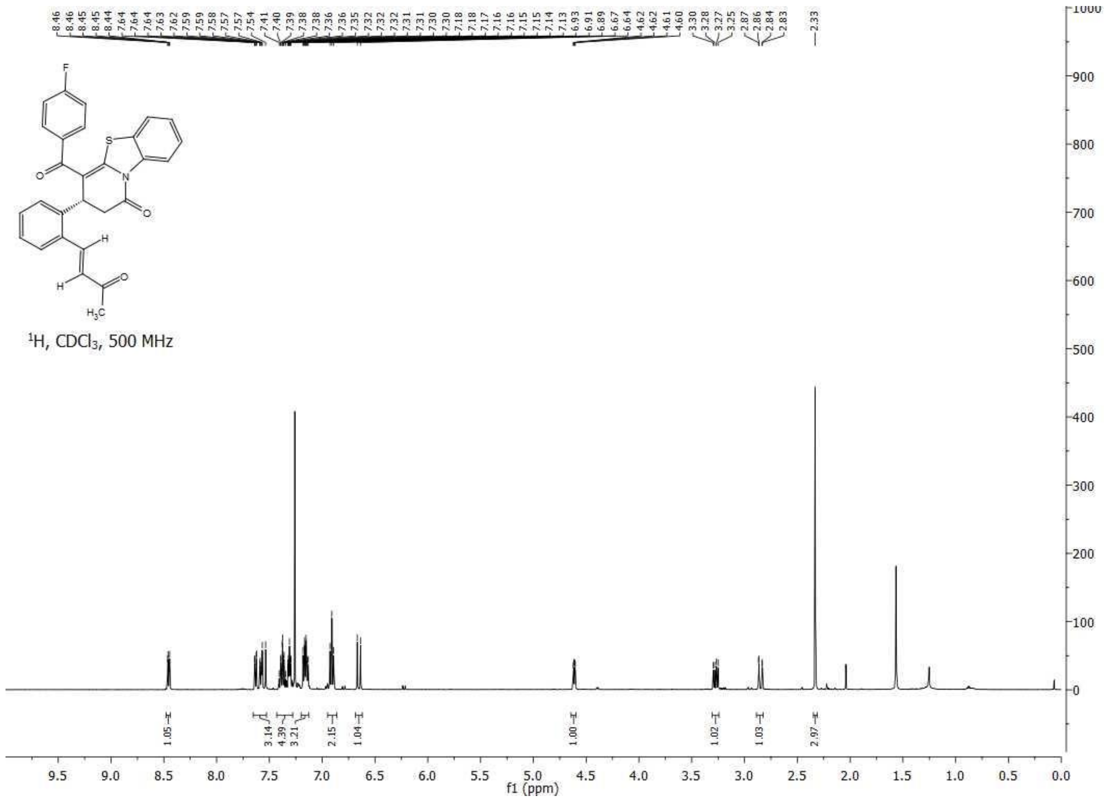

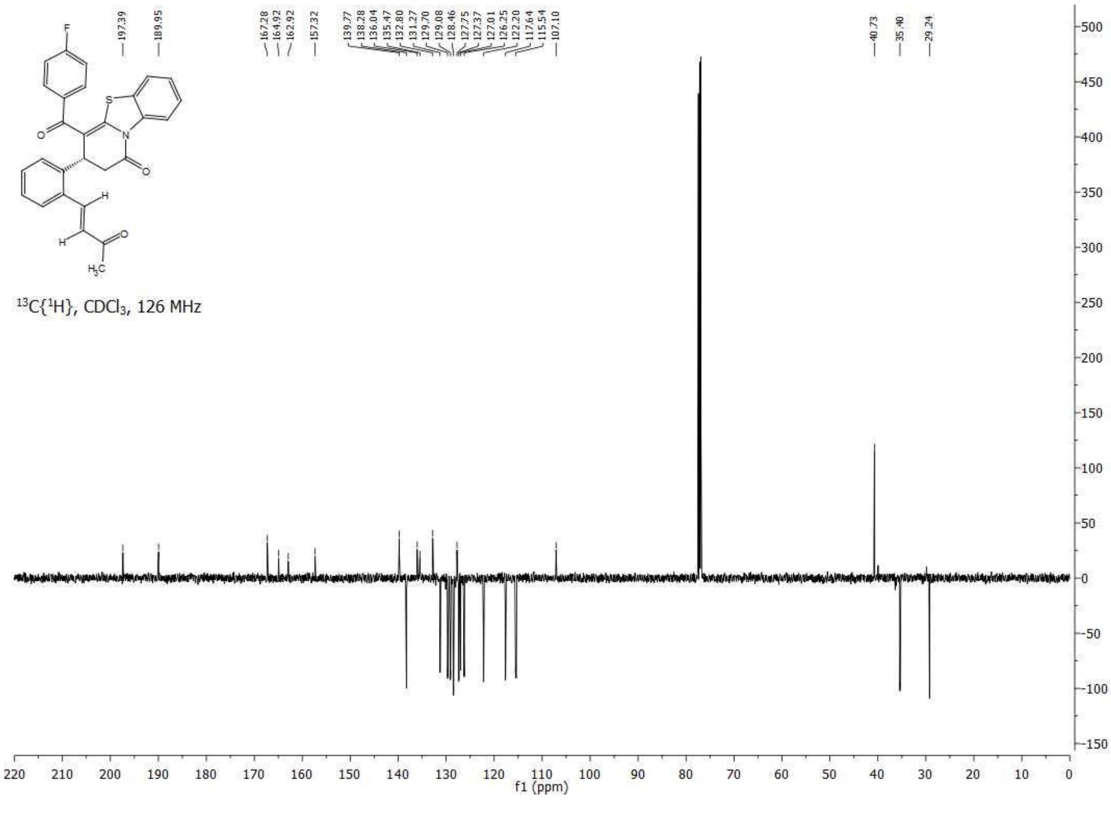

(6a*R*,11*S*,11a*R*)-6-(4-Fluorobenzoyl)-11-(2-oxopropyl)-11,11a-dihydrobenzo[4,5]thiazolo[3,2-*a*]indeno[1,2-*d*]pyridin-12(6a*H*)-one (52)

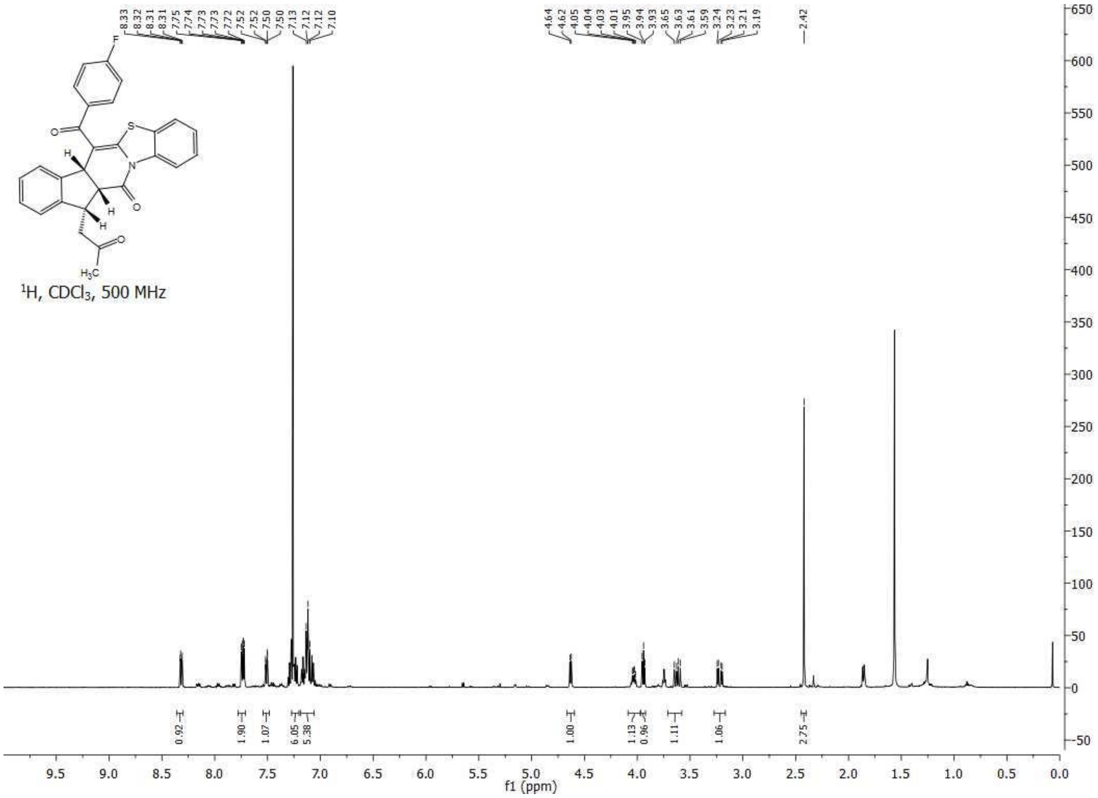

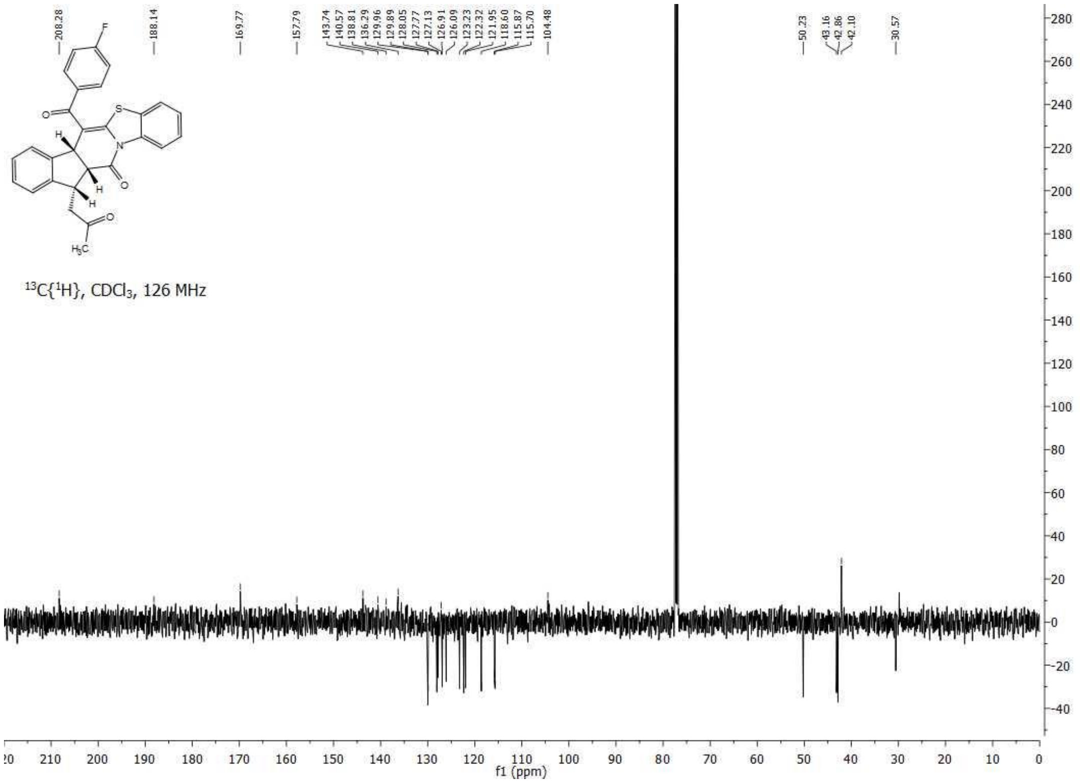

(7a*R*,12*R*,12a*R*)-12a-Benzoyl-12-(2-oxopropyl)-7,7a,12,12a-tetrahydro-6H-benzo[4,5]imidazo[1,2-a]indeno[2,1-*c*]pyridin-6-one (±54)

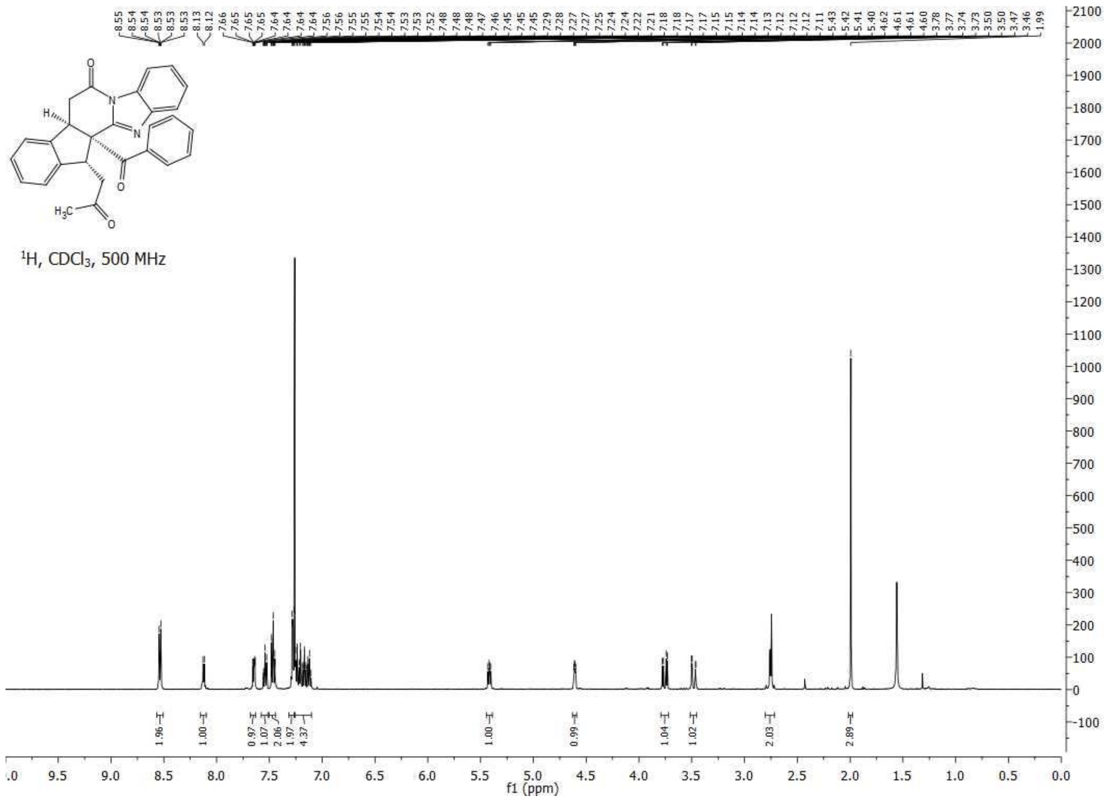

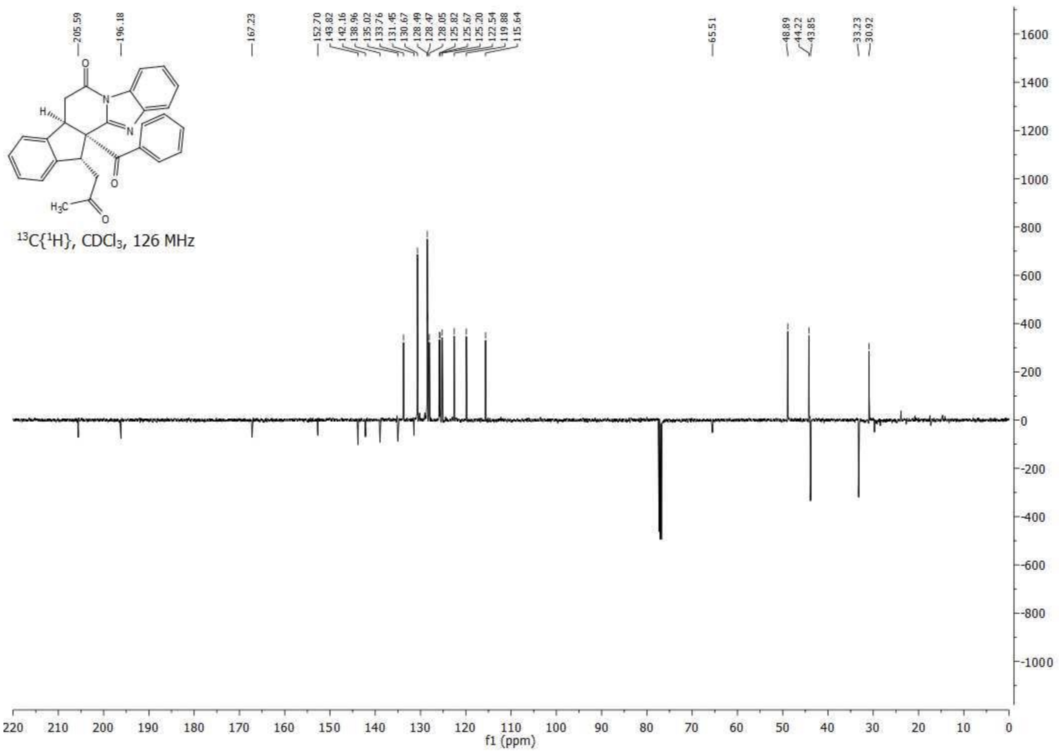

(7a*R*,12*R*,12a*R*)-12a-(4-Methoxybenzoyl)-12-(2-oxopropyl)-7,7a,12,12a-tetrahydro-6Hbenzo[4,5]imidazo[1,2-*a*]indeno[2,1-*c*]pyridin-6-one (±55)

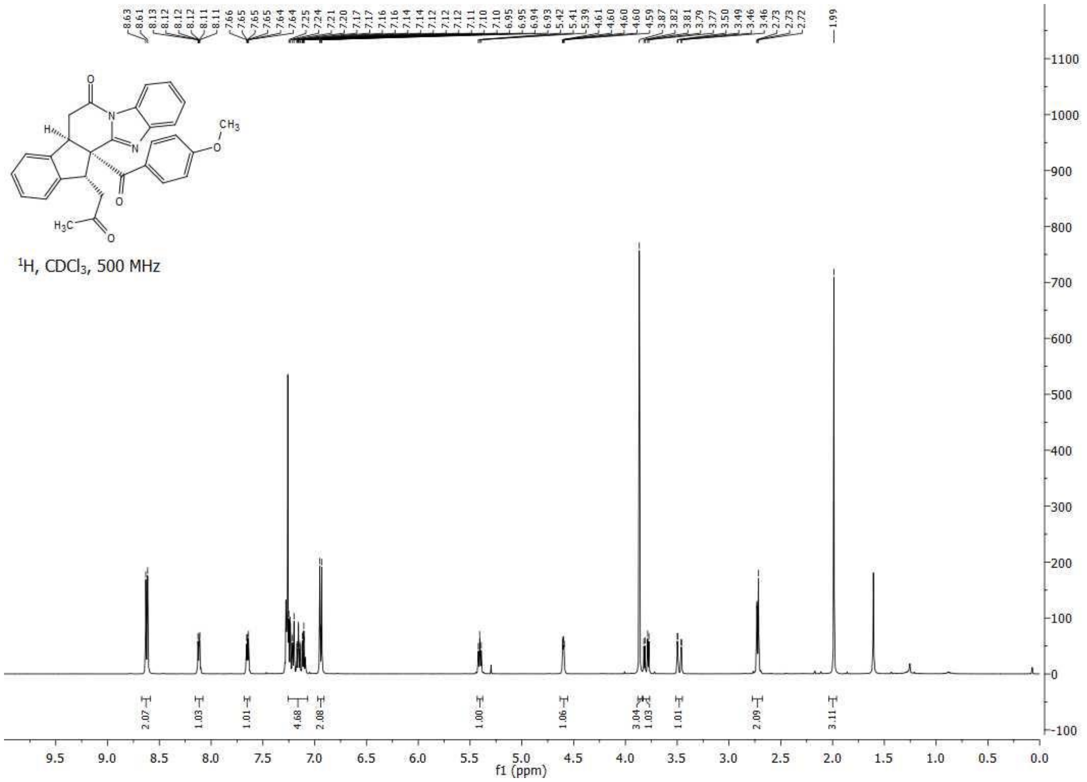

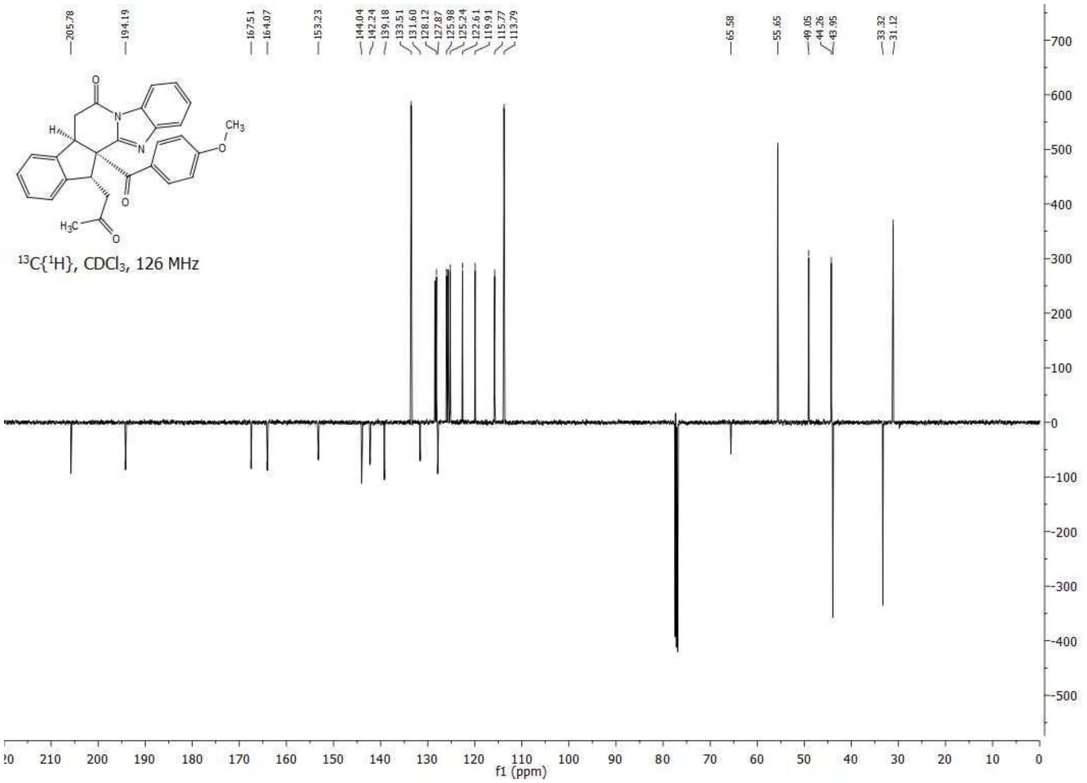

(7a*R*,12*R*,12a*R*)-12a-(4-Nitrobenzoyl)-12-(2-oxopropyl)-7,7a,12,12a-tetrahydro-6Hbenzo[4,5]imidazo[1,2-*a*]indeno[2,1-*c*]pyridin-6-one (±56)

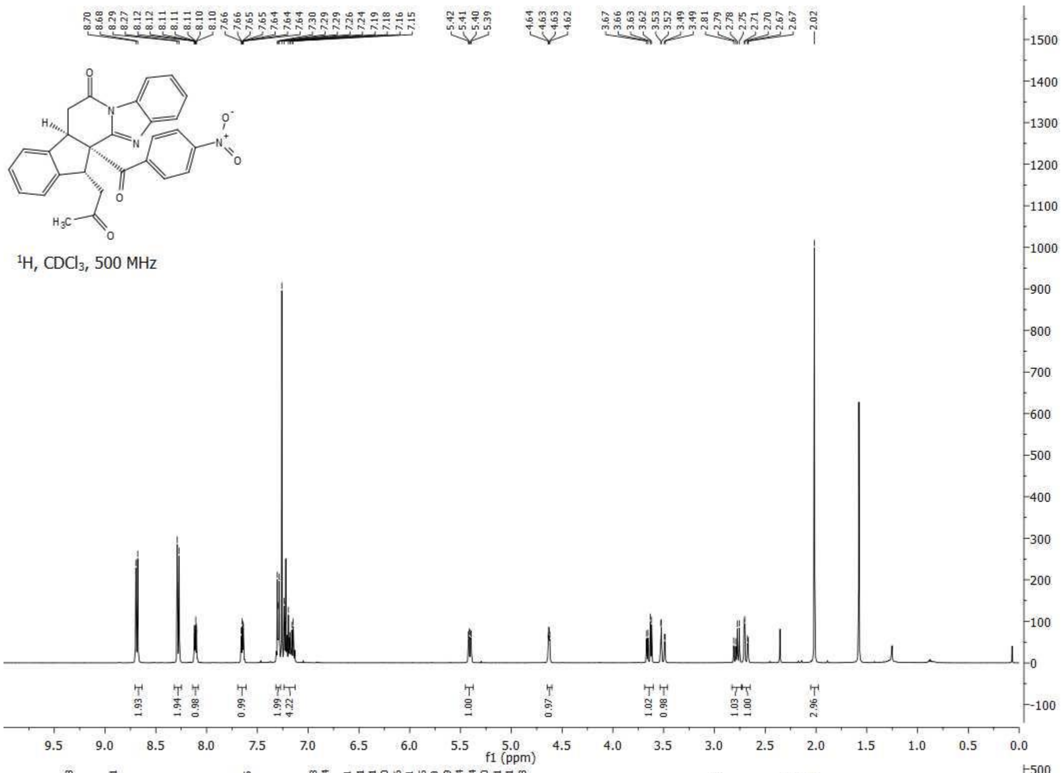

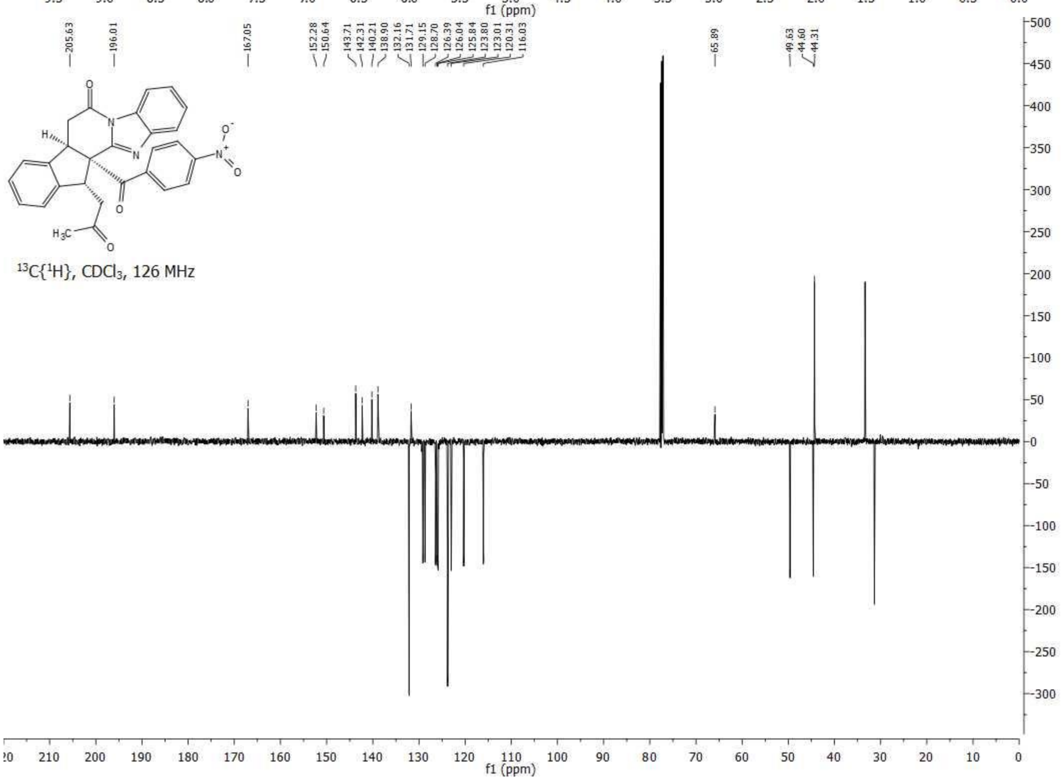

(7a*R*,12*R*,12a*R*)-12a-(4-(Trifluoromethyl)benzoyl)-12-(2-oxopropyl)-7,7a,12,12a-tetrahydro-6Hbenzo[4,5]imidazo[1,2-*a*]indeno[2,1-*c*]pyridin-6-one (±57)

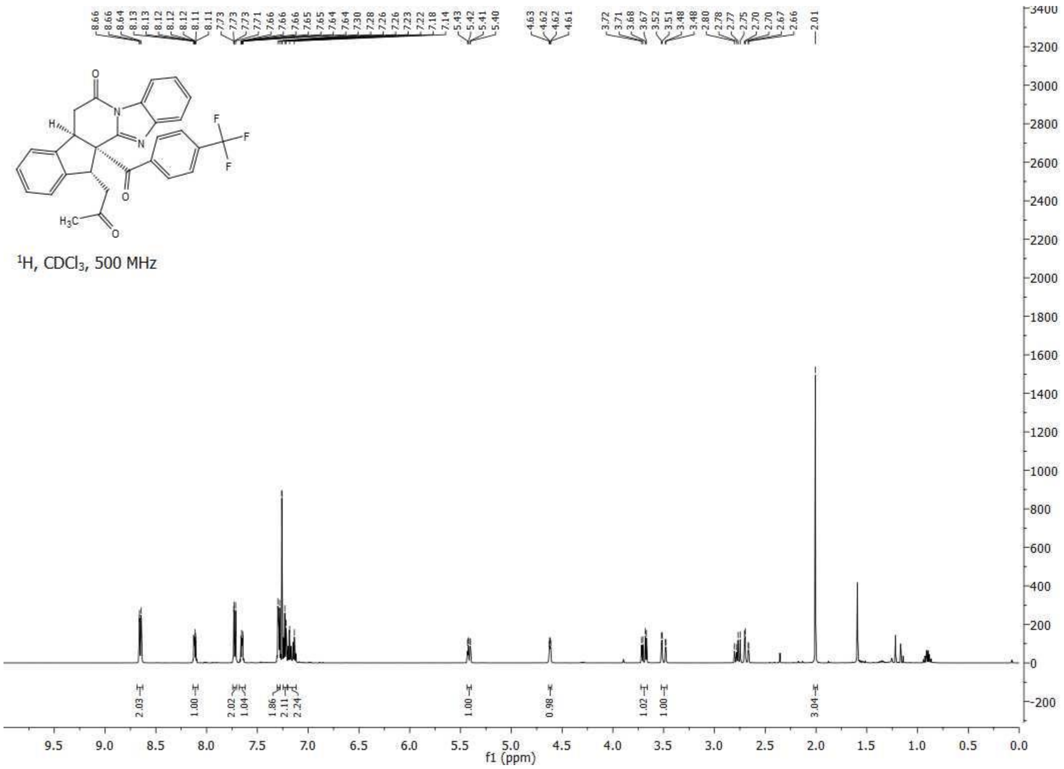

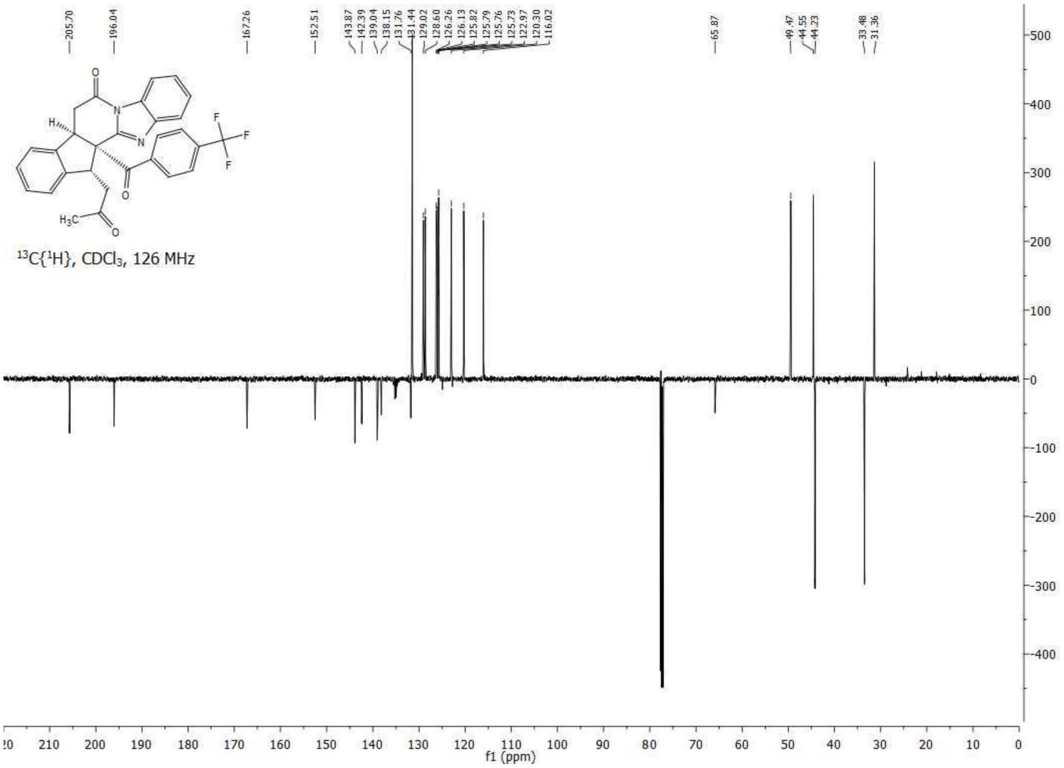

(7a*R*,12*R*,12a*R*)-12a-(4-Fluorobenzoyl)-12-(2-oxopropyl)-7,7a,12,12a-tetrahydro-6Hbenzo[4,5]imidazo[1,2-*a*]indeno[2,1-*c*]pyridin-6-one (±58)

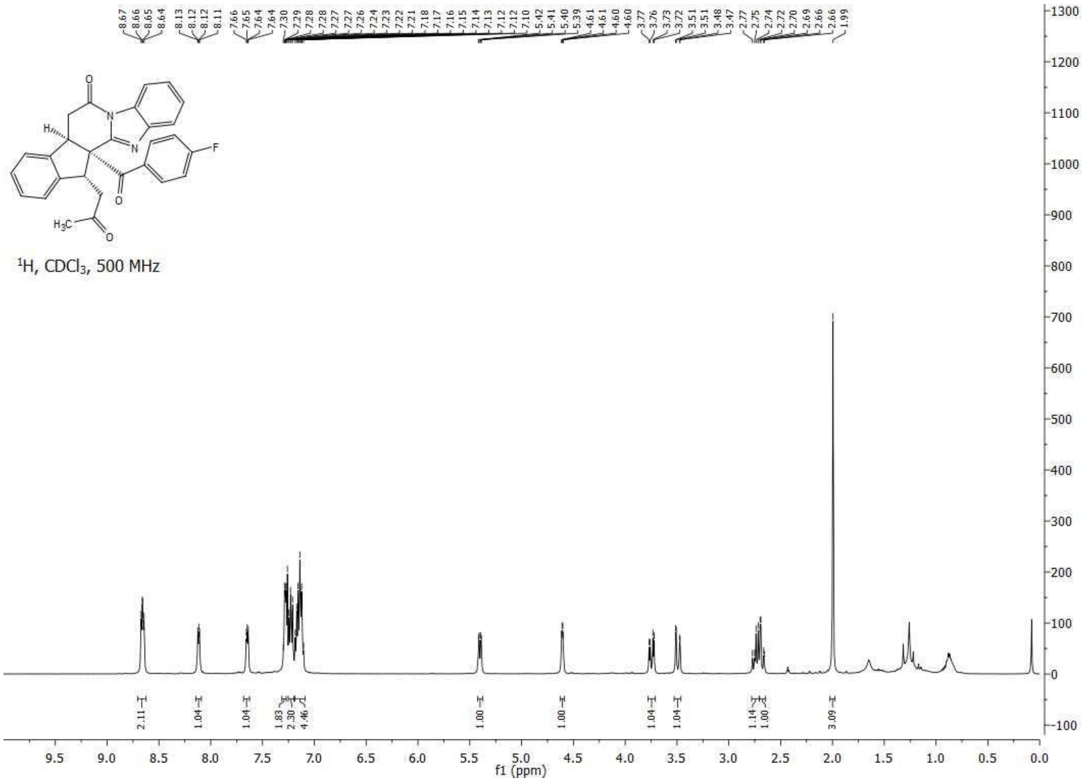

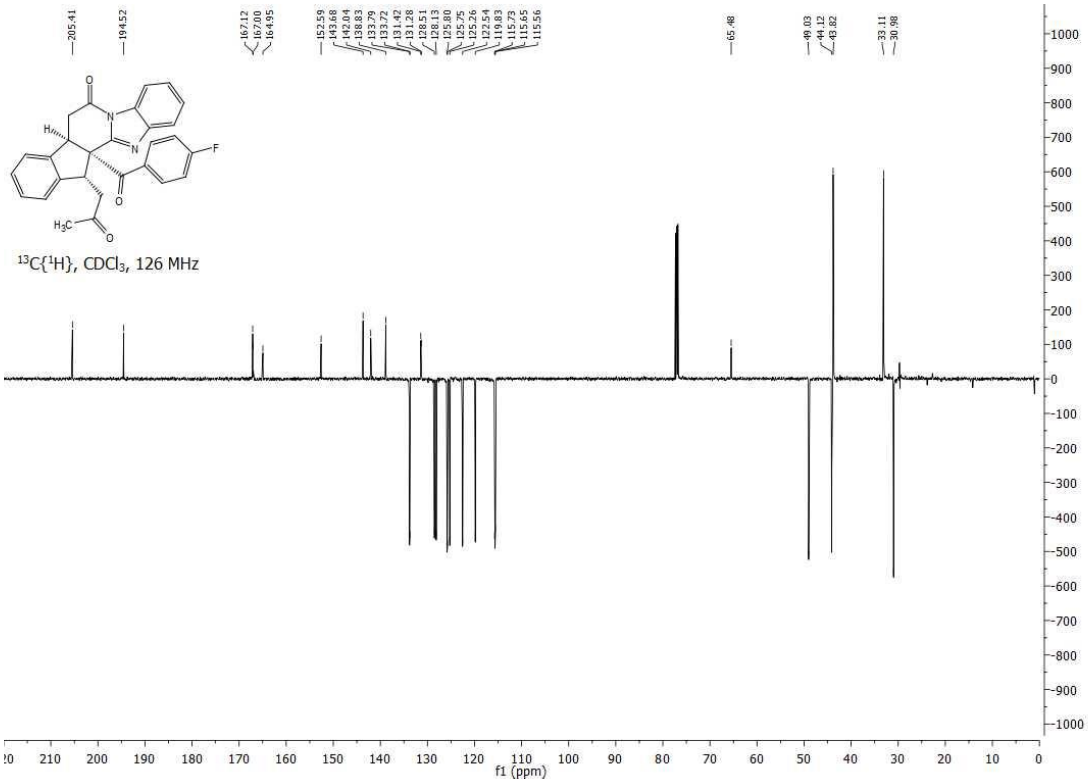

(7a*R*,12*R*,12a*R*)-12a-(4-Bromobenzoyl)-12-(2-oxopropyl)-7,7a,12,12a-tetrahydro-6Hbenzo[4,5]imidazo[1,2-*a*]indeno[2,1-*c*]pyridin-6-one (±59)

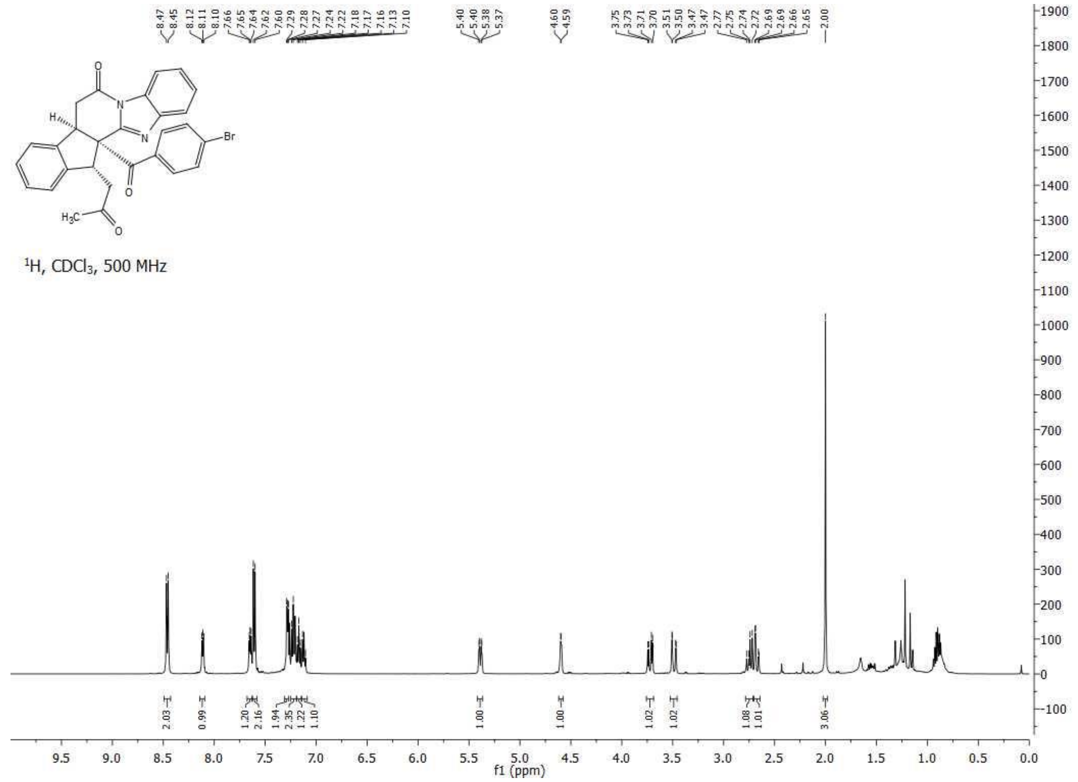

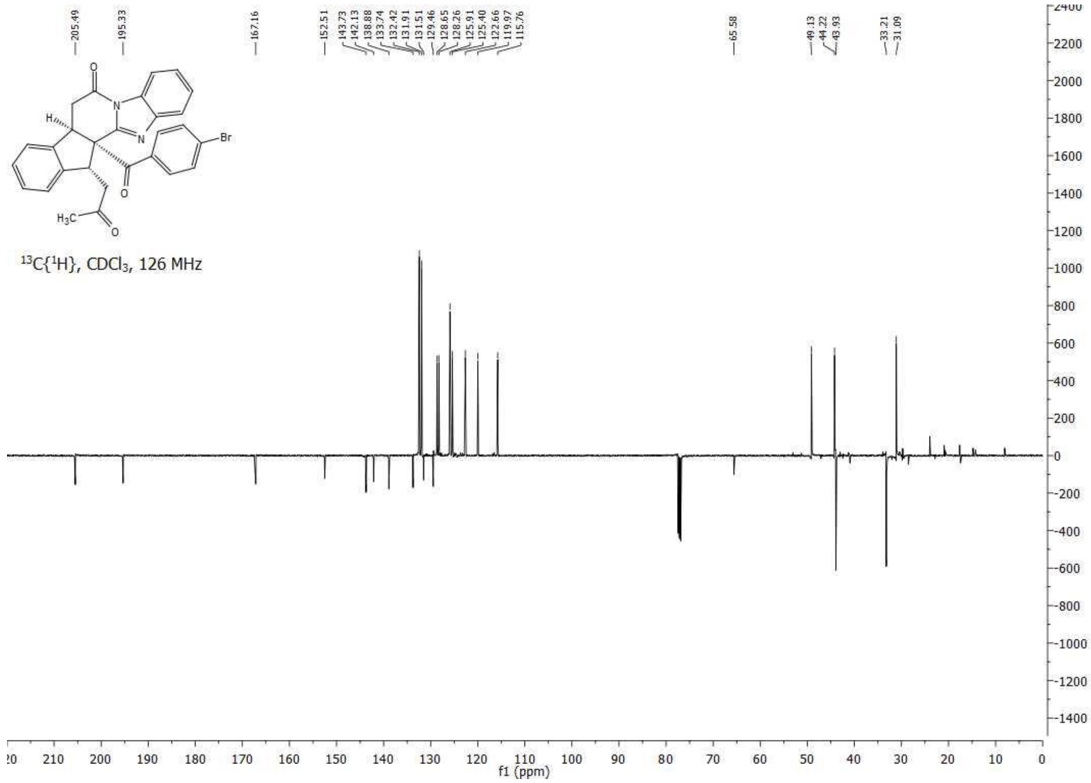

(7a*R*,12*R*,12a*R*)-12a-(3-Methylbenzoyl)-12-(2-oxopropyl)-7,7a,12,12a-tetrahydro-6Hbenzo[4,5]imidazo[1,2-*a*]indeno[2,1-*c*]pyridin-6-one (±60)

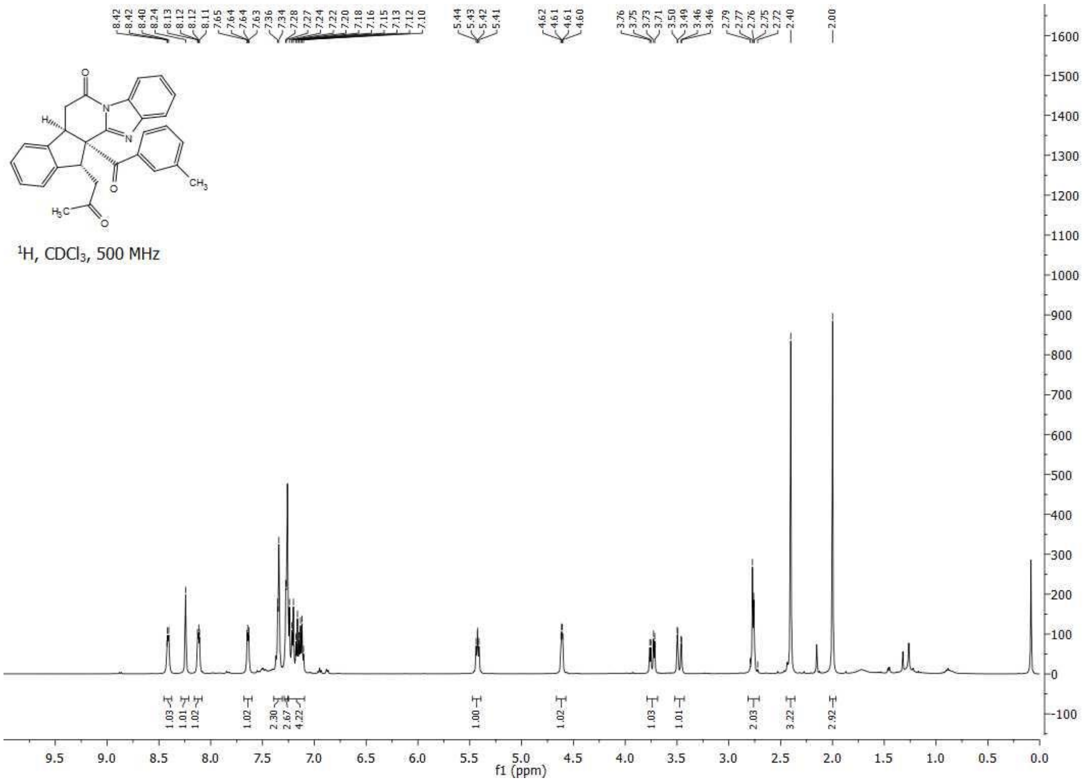

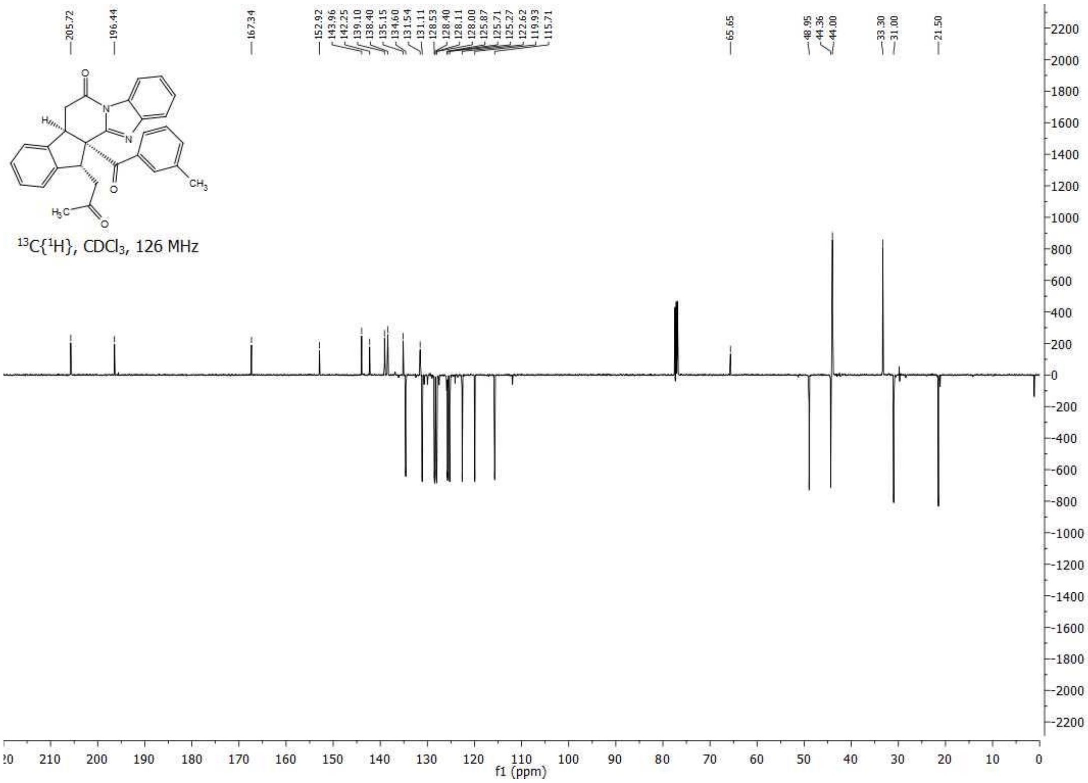

(7a*R*,12*R*,12a*R*)-12a-(Furan-2-carbonyl)-12-(2-oxopropyl)-7,7a,12,12a-tetrahydro-6Hbenzo[4,5]imidazo[1,2-*a*]indeno[2,1-*c*]pyridin-6-one (±61)

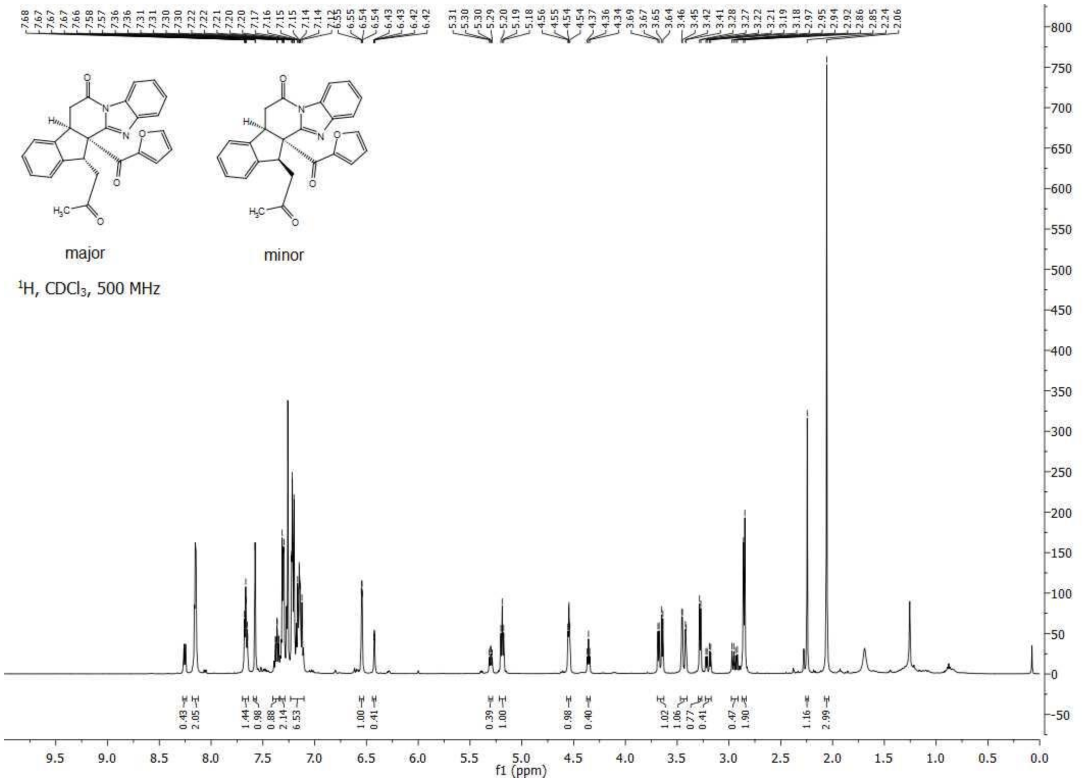

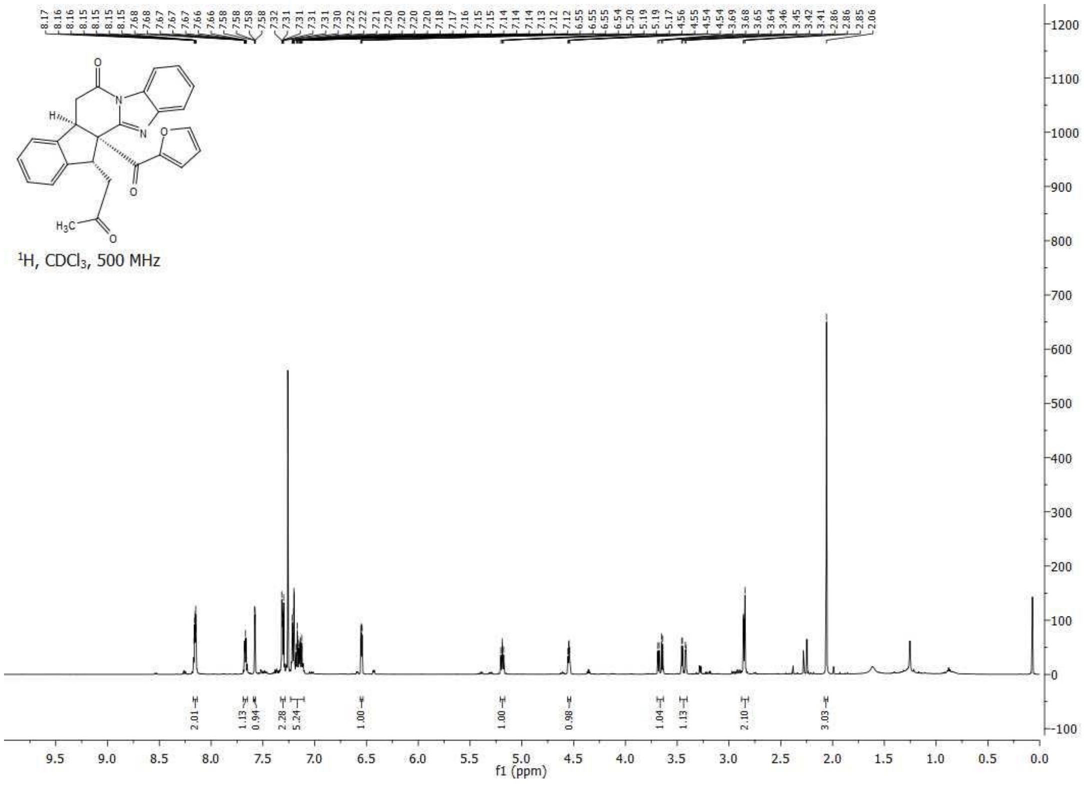

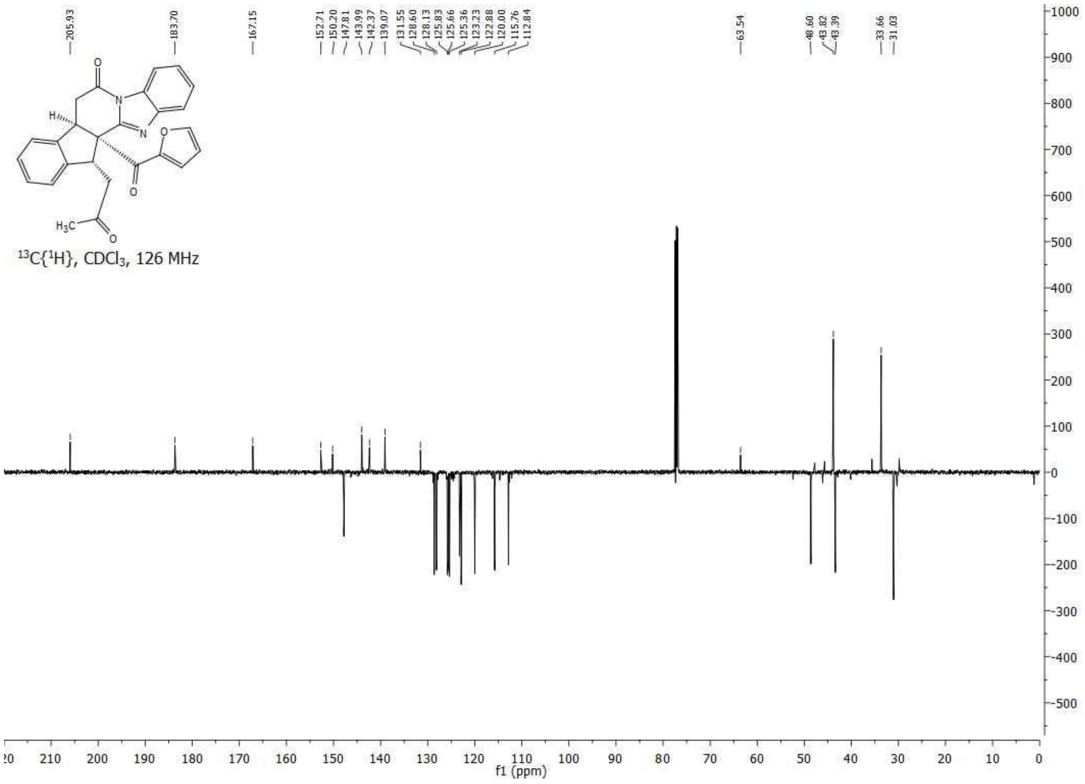
205.93
183.70
167.15
152.71
150.20
147.81
143.99
142.37
139.07
131.55
128.60
128.13
125.83
125.66
125.36
123.23
122.88
120.00
115.76
112.84
63.54
48.60
43.82
43.39
33.66
31.03
$^{13}C\{^1H\}$, $CDCl_3$, 126 MHz
f1 (ppm)

(7a*R*,12*R*,12a*R*)-12a-Benzoyl-3-bromo-12-(2-oxopropyl)-7,7a,12,12a-tetrahydro-6Hbenzo[4,5]imidazo[1,2-*a*]indeno[2,1-*c*]pyridin-6-one (±62)

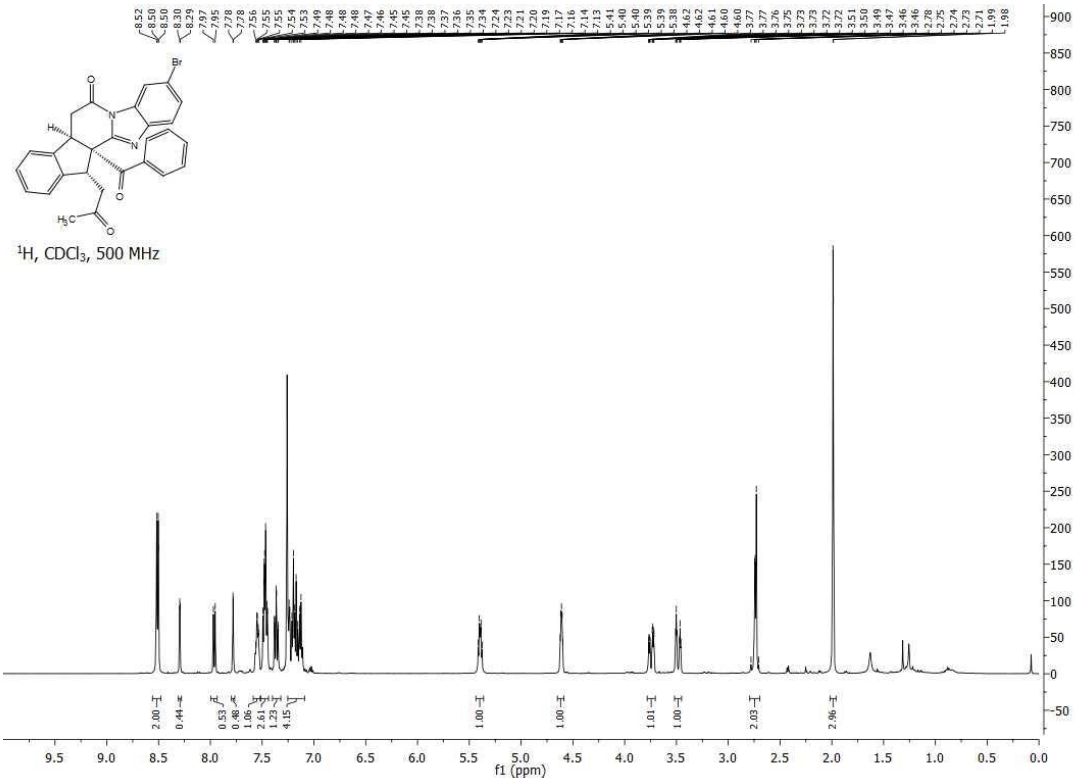

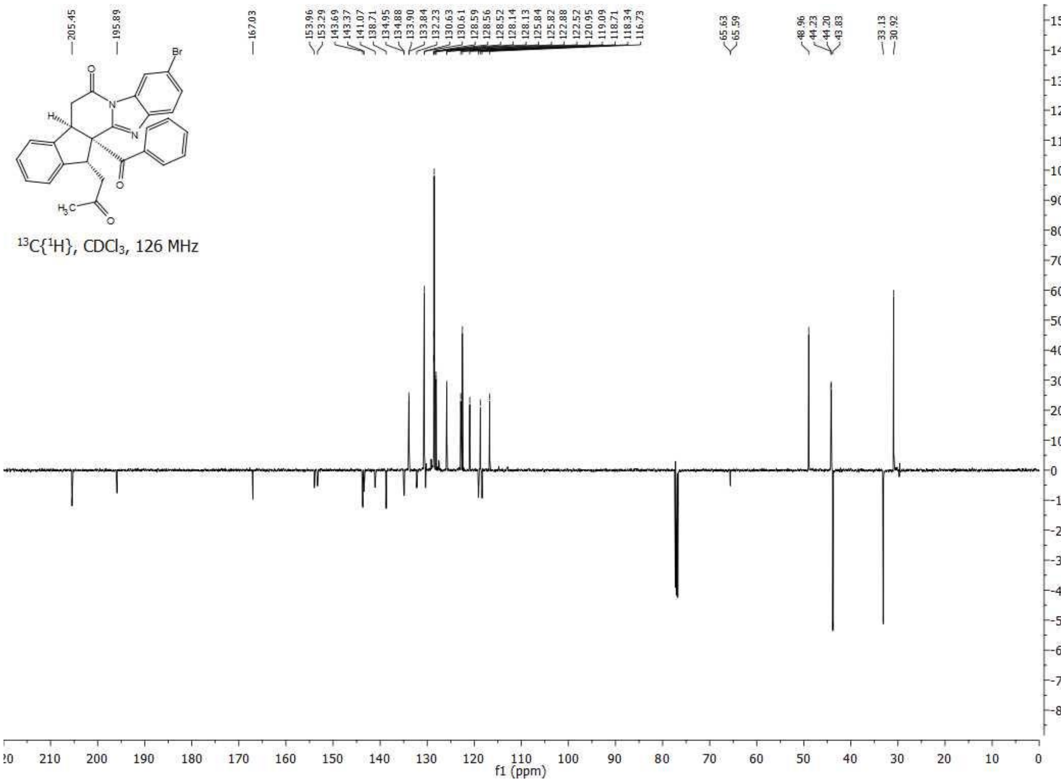

(7a*R*,12*R*,12a*R*)-3-Chloro-12a-(4-methoxybenzoyl)-12-(2-oxopropyl)-7,7a,12,12a-tetrahydro-6Hbenzo[4,5]imidazo[1,2-*a*]indeno[2,1-*c*]pyridin-6-one (±63)

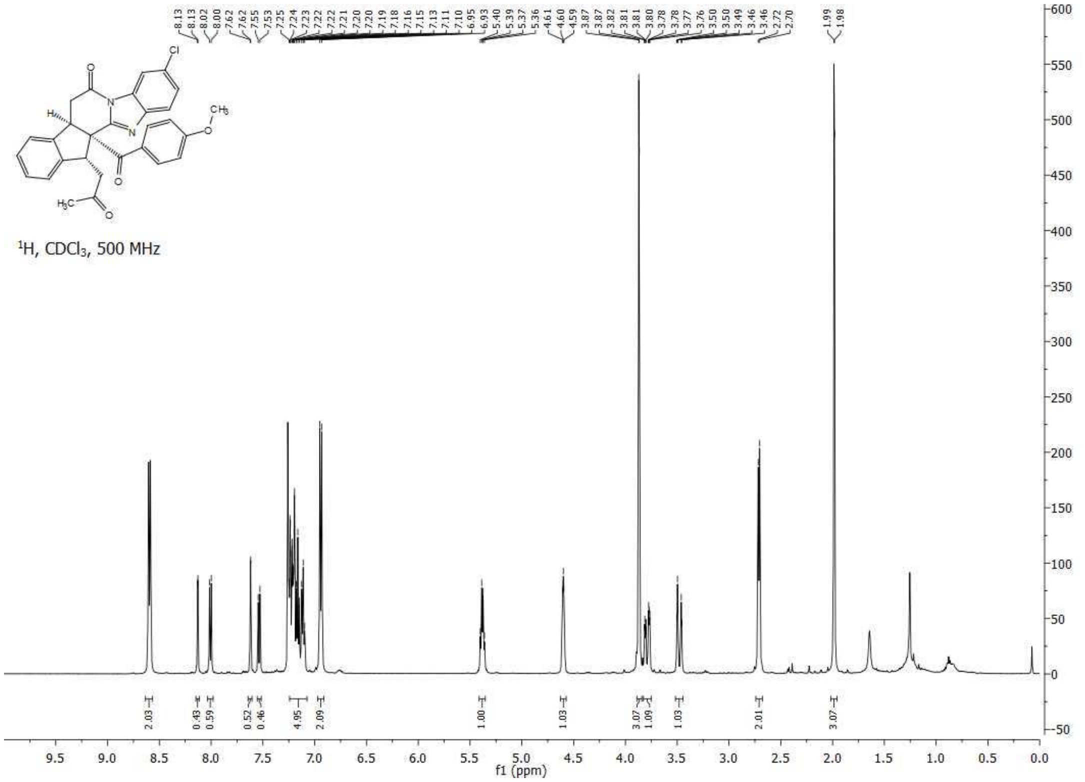

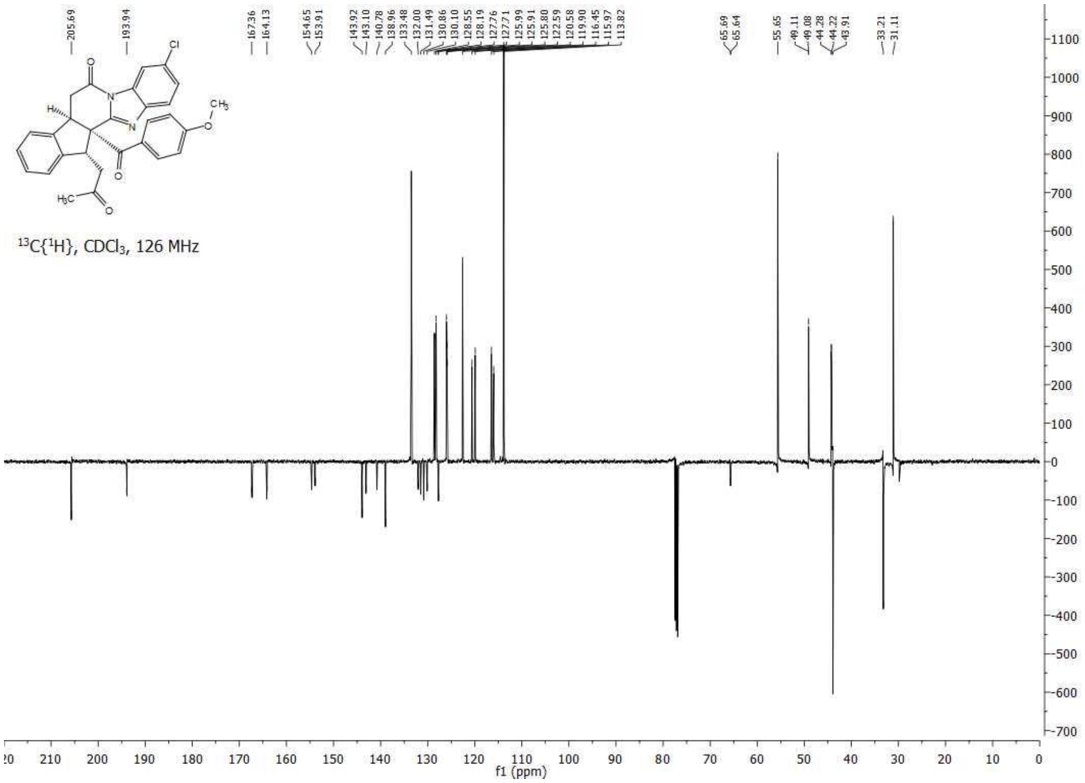

(7aR,12R,12aR)-12a-Benzoyl-2,3-dimethyl-12-(2-oxopropyl)-7,7a,12,12a-tetrahydro-6Hbenzo[4,5]imidazo[1,2-a]indeno[2,1-c]pyridin-6-one (±64)

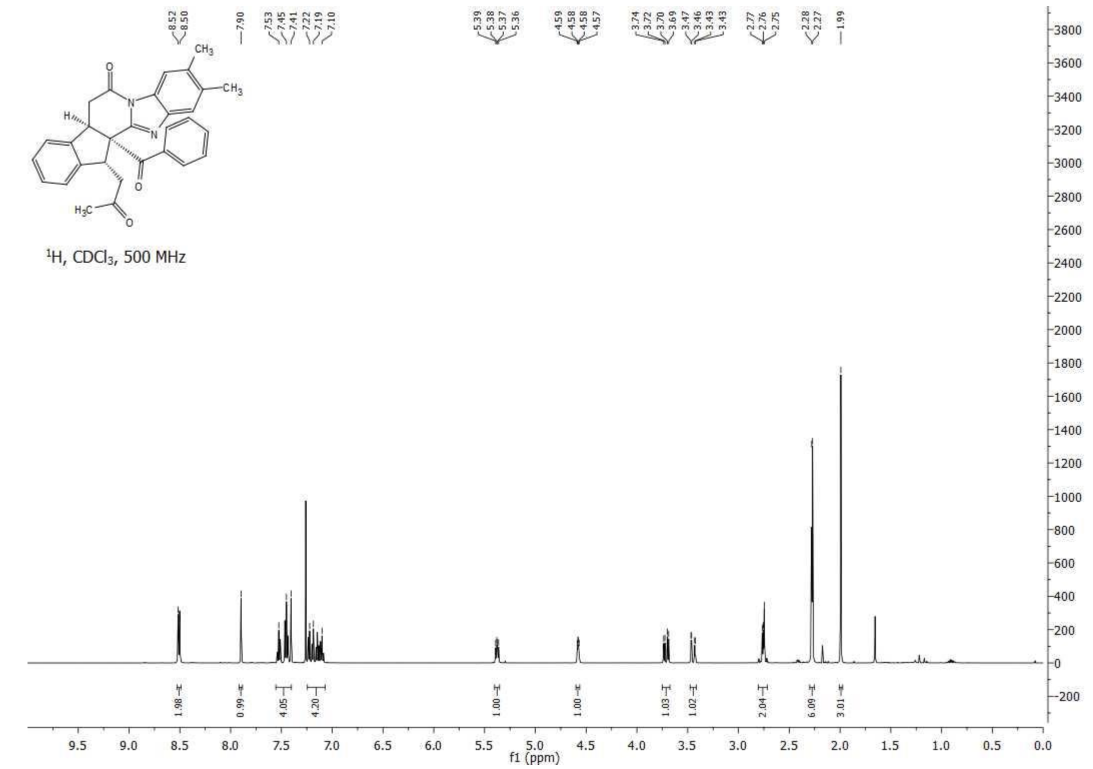

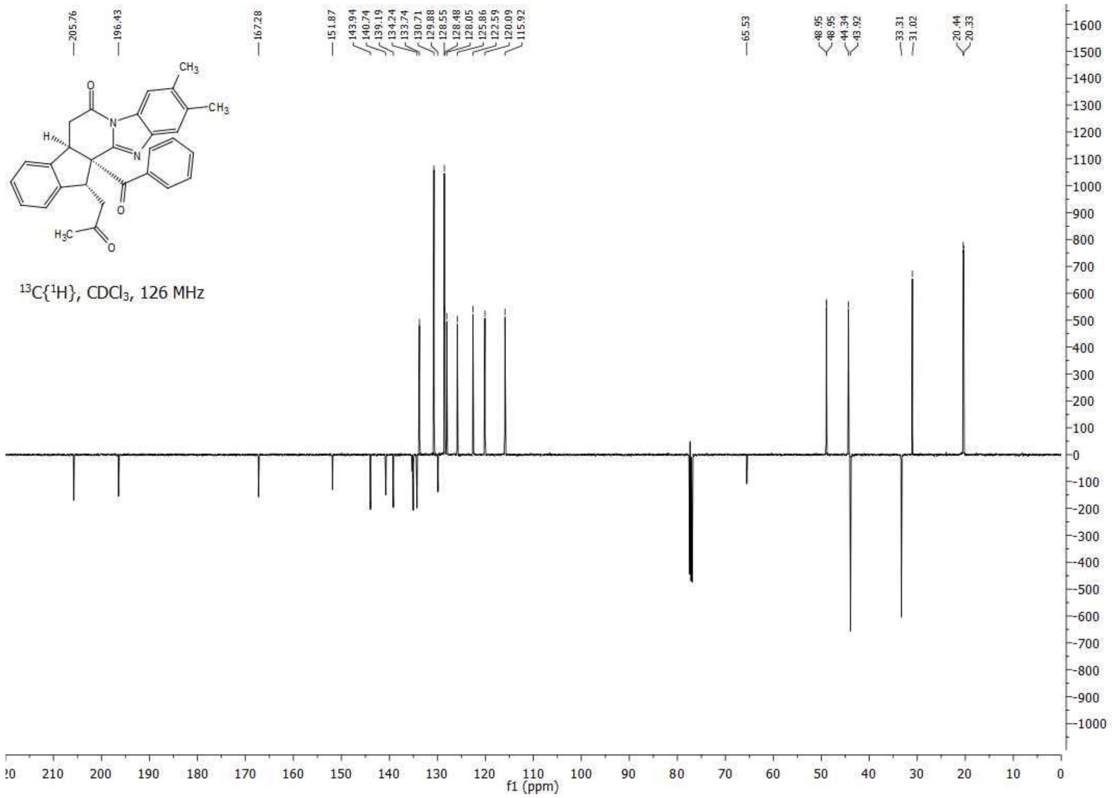

(7a*R*,12*R*,12a*R*)-12a-Benzoyl-9-methyl-12-(2-oxopropyl)-7,7a,12,12a-tetrahydro-6Hbenzo[4,5]imidazo[1,2-*a*]indeno[2,1-*c*]pyridin-6-one (±65)

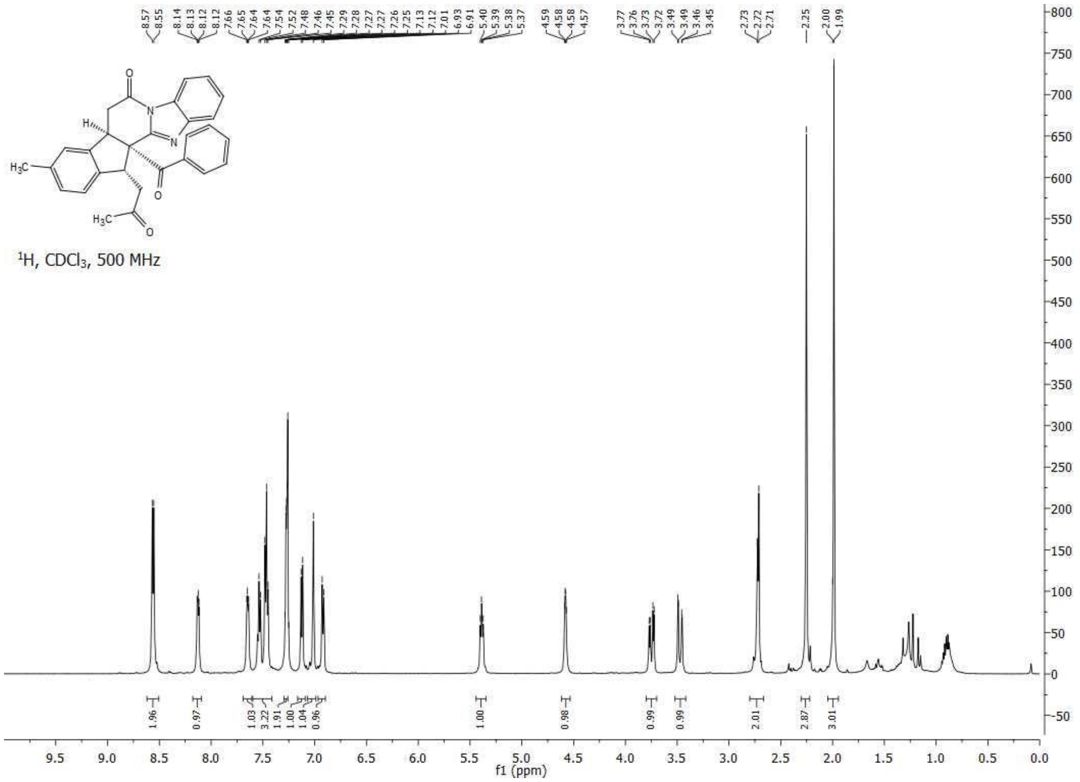

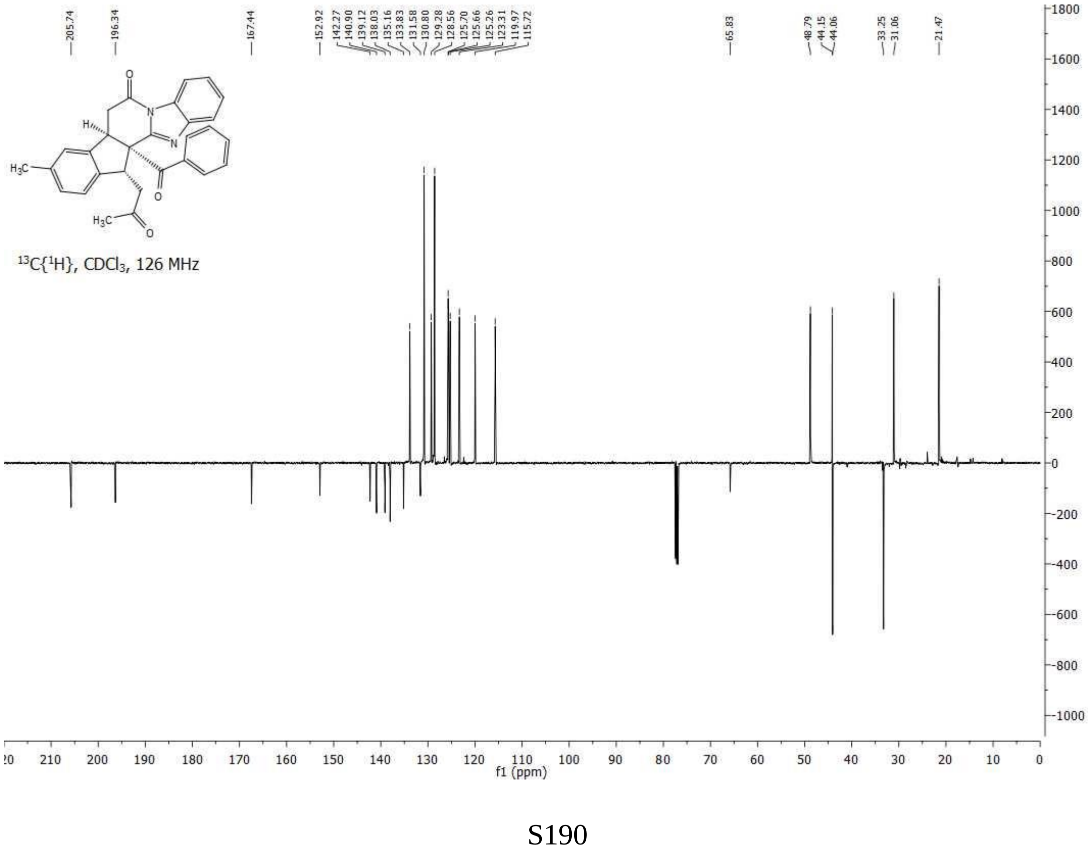

(7a*R*,12*R*,12a*R*)-12a-Benzoyl-10-fluoro-12-(2-oxopropyl)-7,7a,12,12a-tetrahydro-6Hbenzo[4,5]imidazo[1,2-*a*]indeno[2,1-*c*]pyridin-6-one (±66)

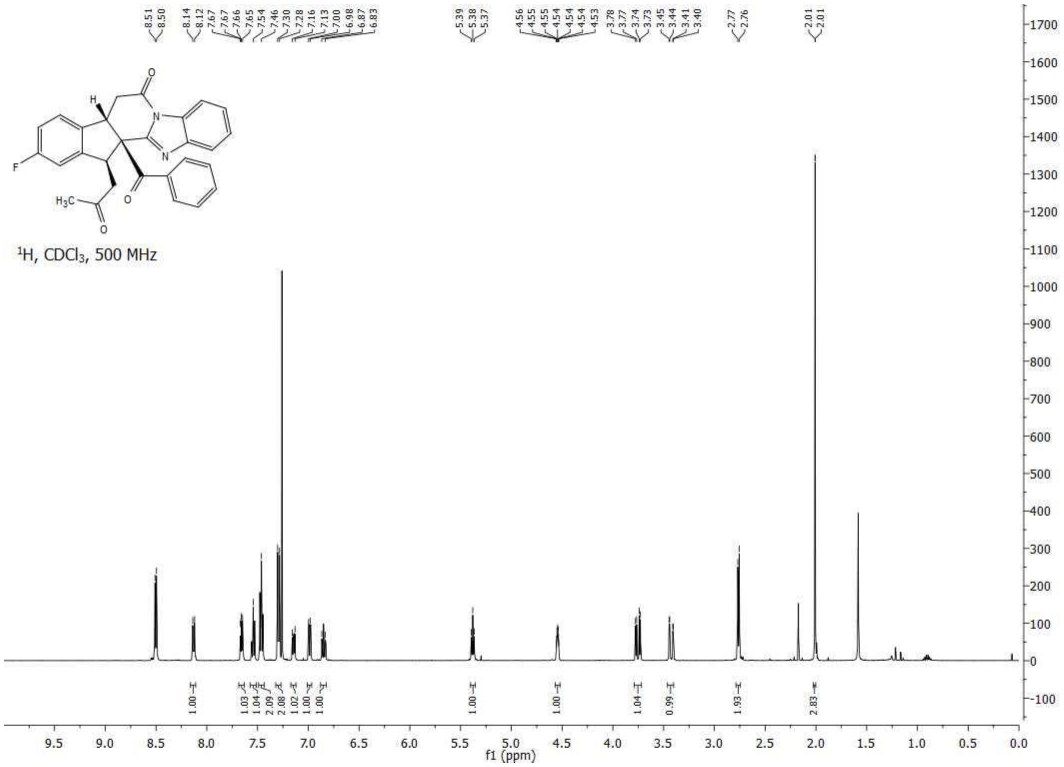

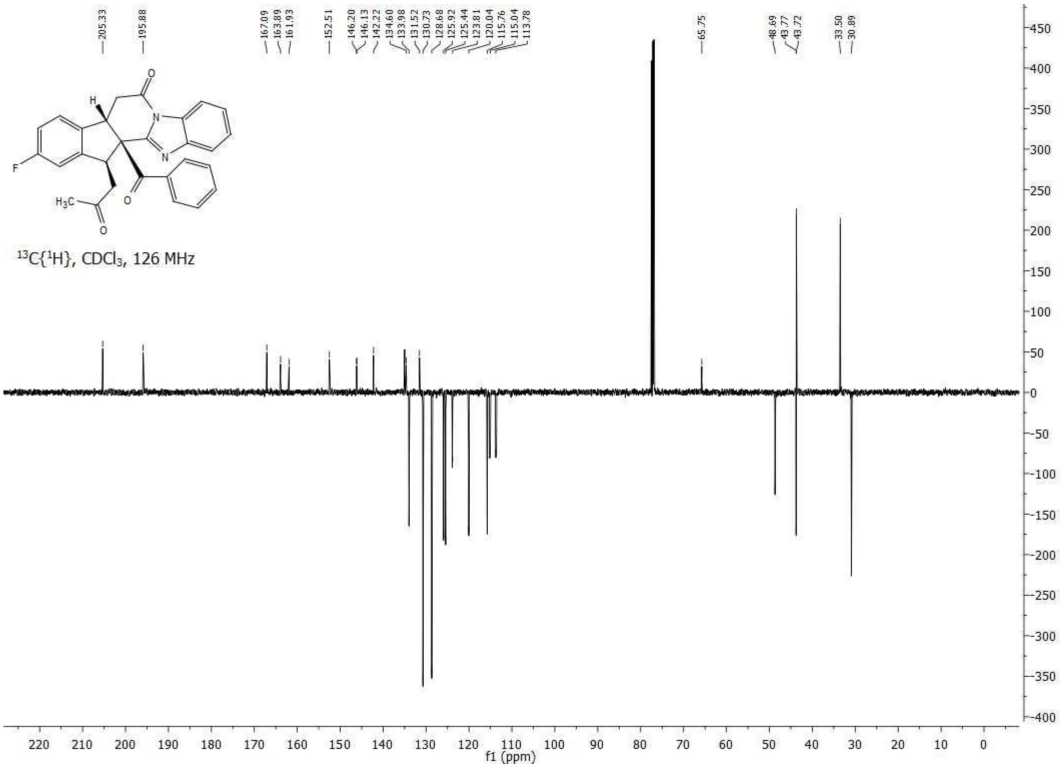

(7a*R*,12*R*,12a*R*)-12a-Benzoyl-12-(3,3-dimethyl-2-oxobutyl)-7,7a,12,12a-tetrahydro-6Hbenzo[4,5]imidazo[1,2-*a*]indeno[2,1-*c*]pyridin-6-one (±67)

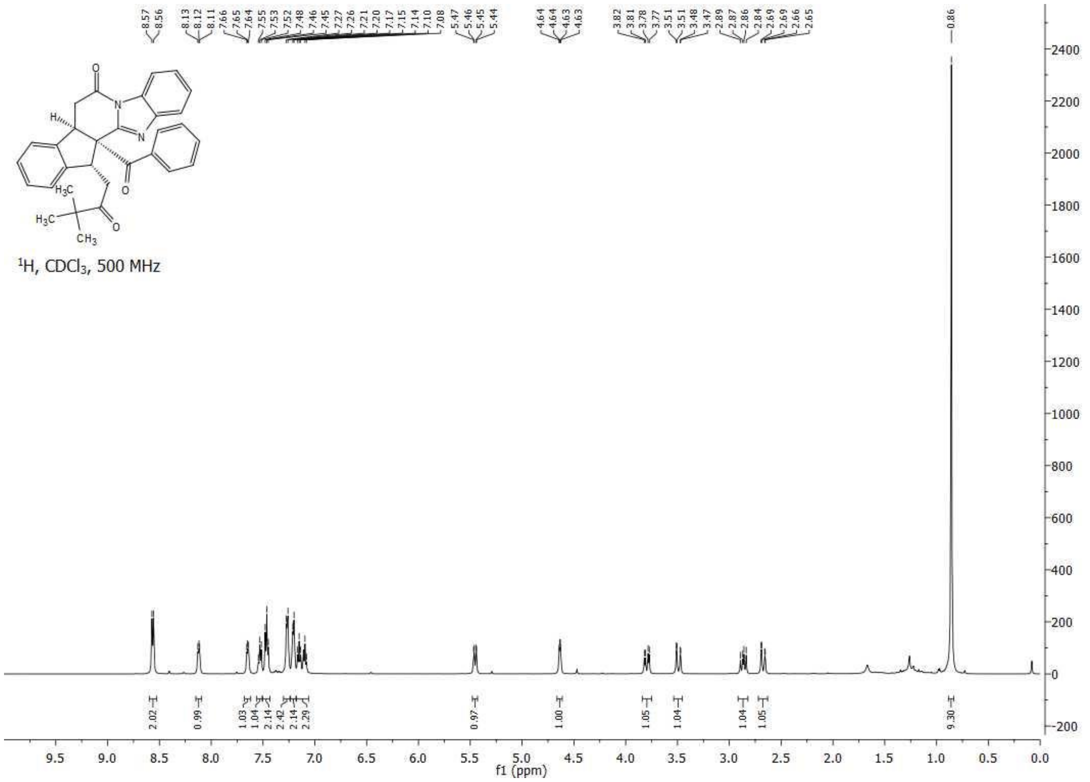

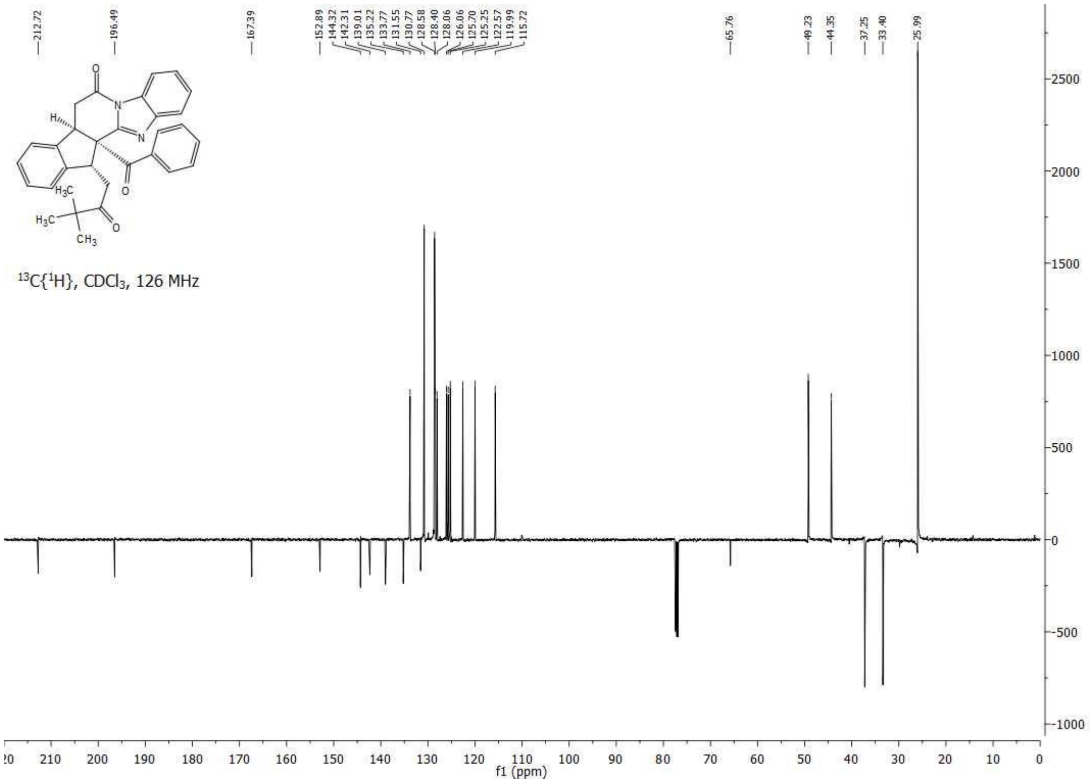

(7a*R*,12*R*,12a*R*)-12a-Benzoyl-12-(2-(4-methoxyphenyl)-2-oxoethyl)-7,7a,12,12a-tetrahydro-6Hbenzo[4,5]imidazo[1,2-*a*]indeno[2,1-*c*]pyridin-6-one (±68)

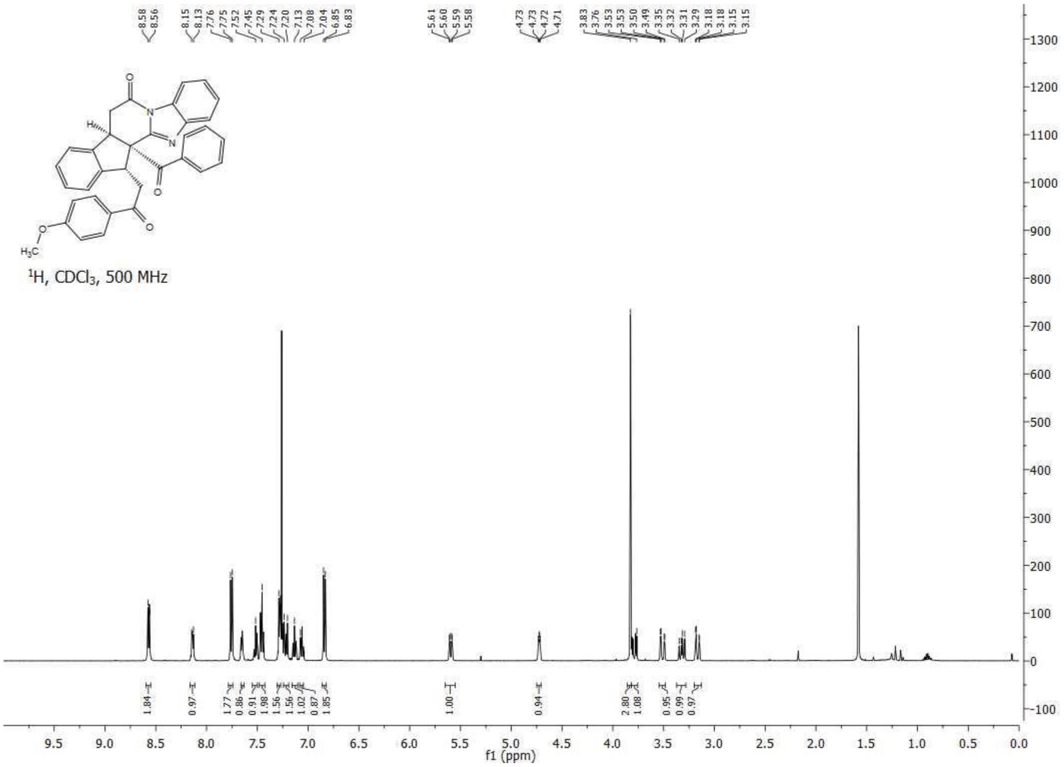

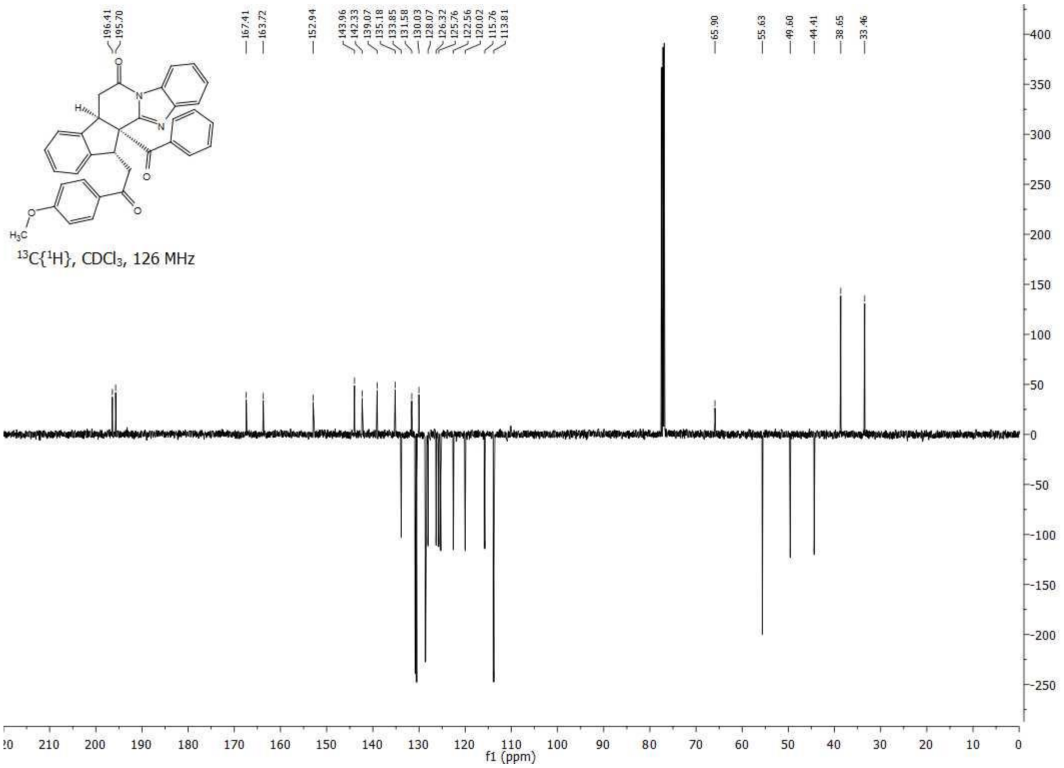

Methyl 2-((7a*R*,12*R*,12a*S*)-12a-benzoyl-6-oxo-7,7a,12,12a-tetrahydro-6H-benzo[4,5]imidazo[1,2-*a*]indeno[2,1-*c*]pyridin-12-yl)acetate (±69)

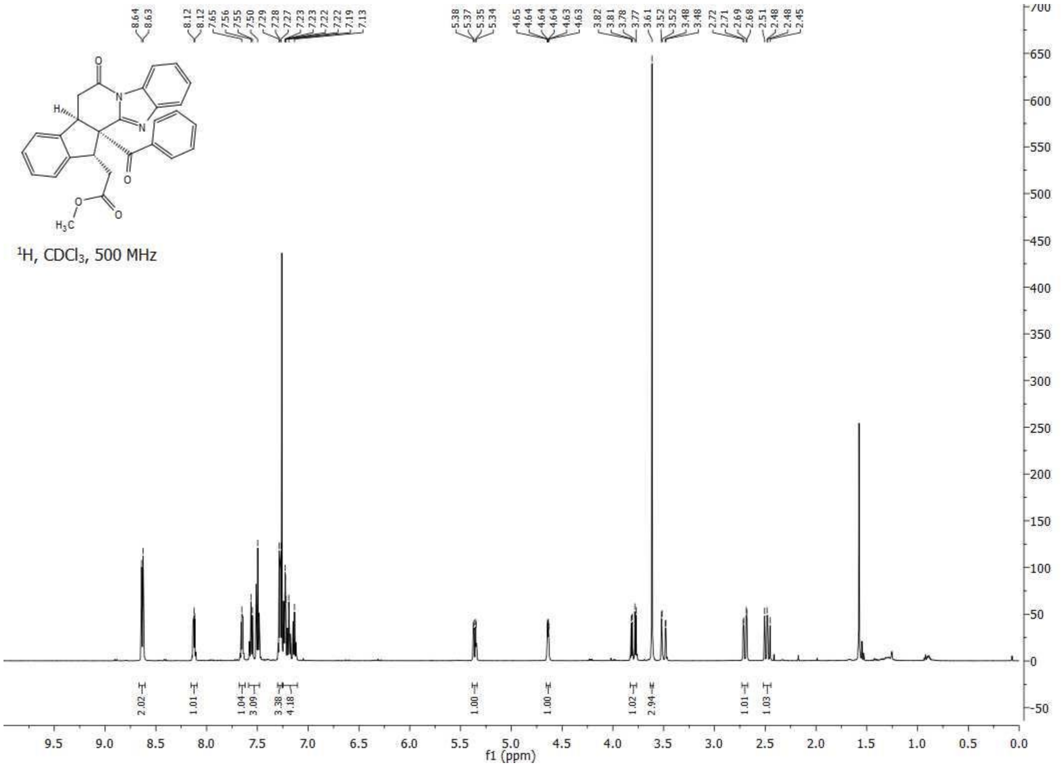

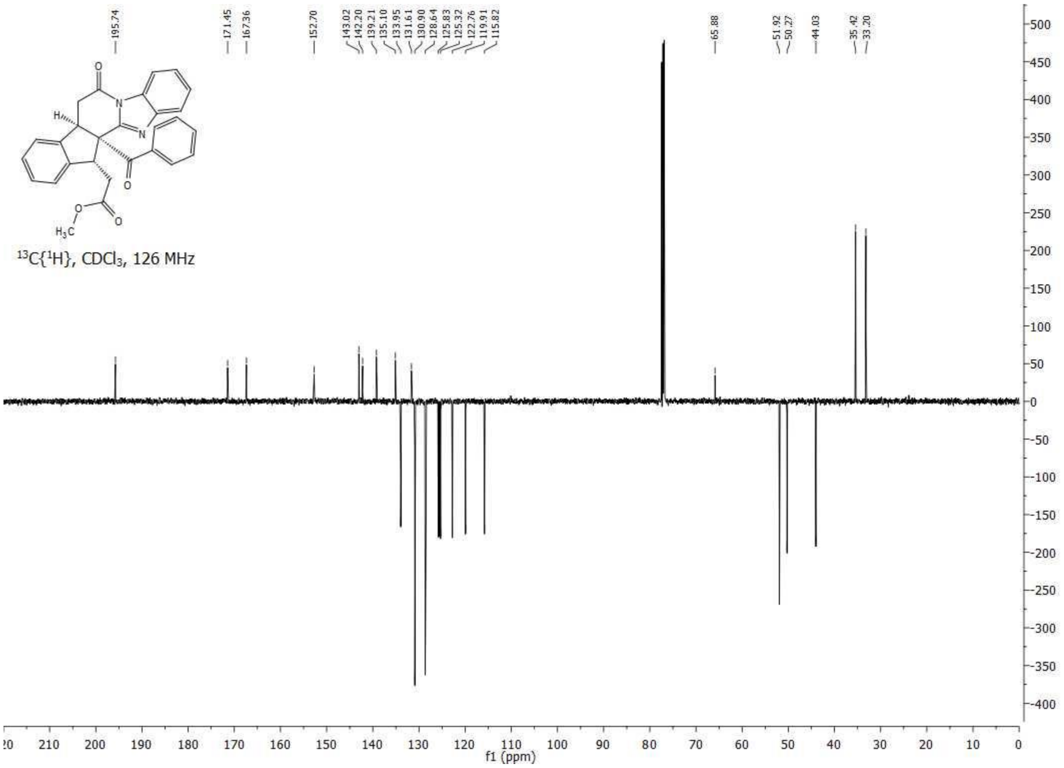

Appendix II: HPLC Traces

HPLC Data for **7a**: Chiralpak IB (97:3 hexane : IPA, flow rate 1.5 mLmin^{-1}, 211 nm, 40 °C) t_R major: 18.2 min, t_R minor: 29.0 min, >99:1 er

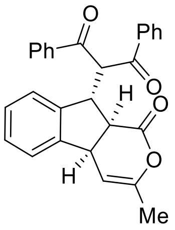

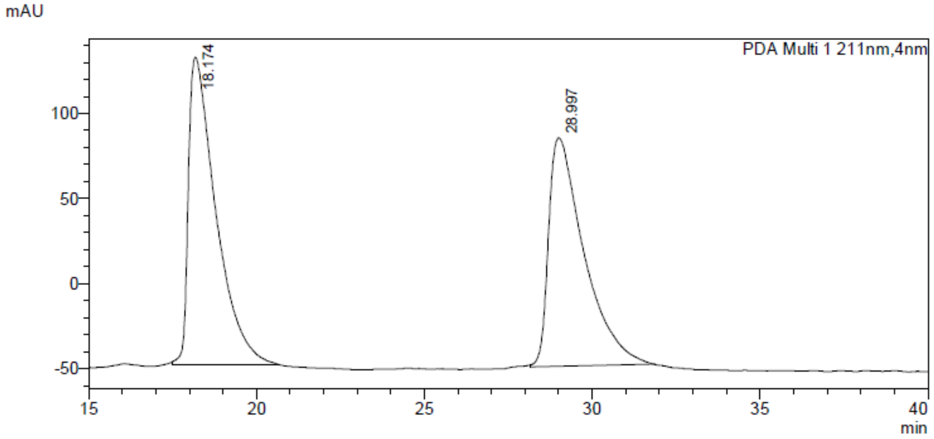

<Peak Table>

PDA Ch1 211nm

Peak#	Ret. Time	Area%
1	18.174	50.950
2	28.997	49.050
Total		100.000

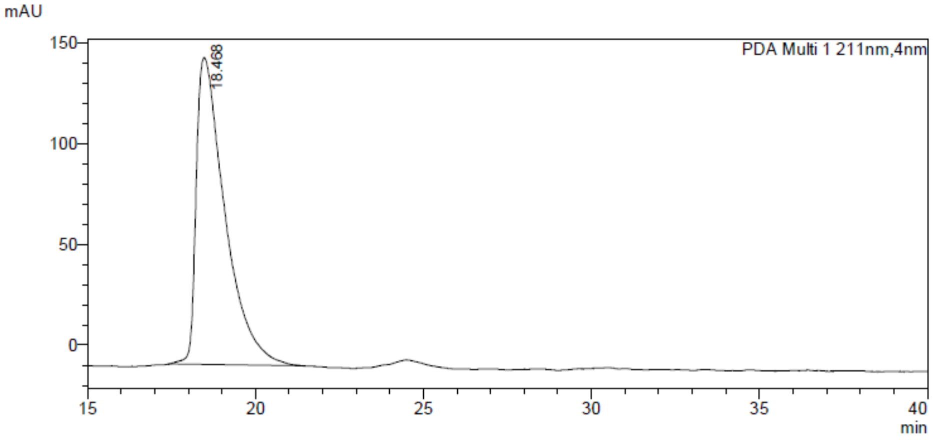

<Peak Table>

PDA Ch1 211nm

Peak#	Ret. Time	Area%
1	18.468	100.000
Total		100.000

HPLC Data for **7b**: Chiralpak IB (97:3 hexane : IPA, flow rate 1.5 mLmin^{-1}, 211 nm, 40 °C) t_R major: 15.2 min, t_R minor: 20.1 min, >99:1 er

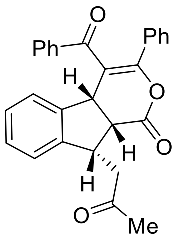

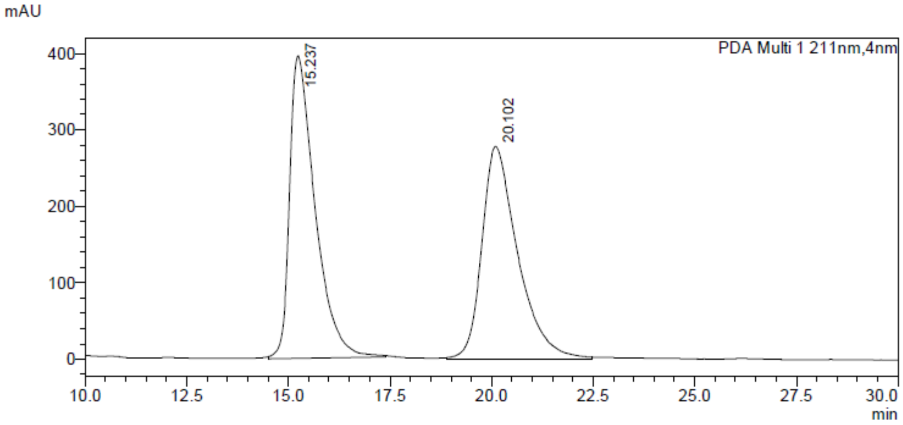

<Peak Table>

PDA Ch1 211nm

Peak#	Ret. Time	Area%
1	15.237	50.431
2	20.102	49.569
Total		100.000

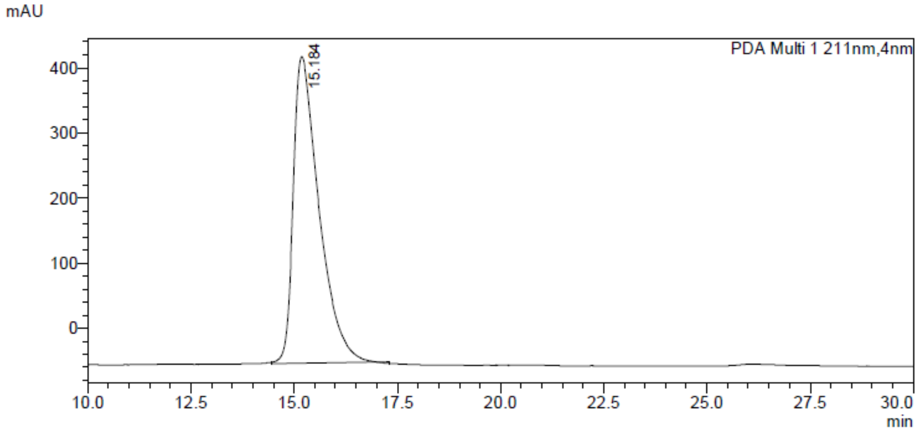

<Peak Table>

PDA Ch1 211nm

Peak#	Ret. Time	Area%
1	15.184	100.000
Total		100.000

HPLC data for **10a**: Chiralpak AS-H (90:10 hexane : IPA, flow rate 1.5 mLmin^{-1}, 220 nm, 40 °C) t_R minor: 18.5 min, t_R major: 28.6 min, 97.5:2.5 er

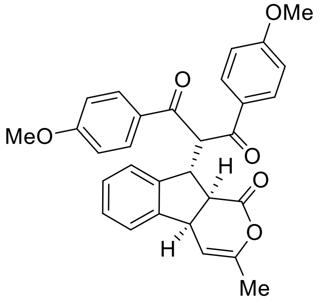

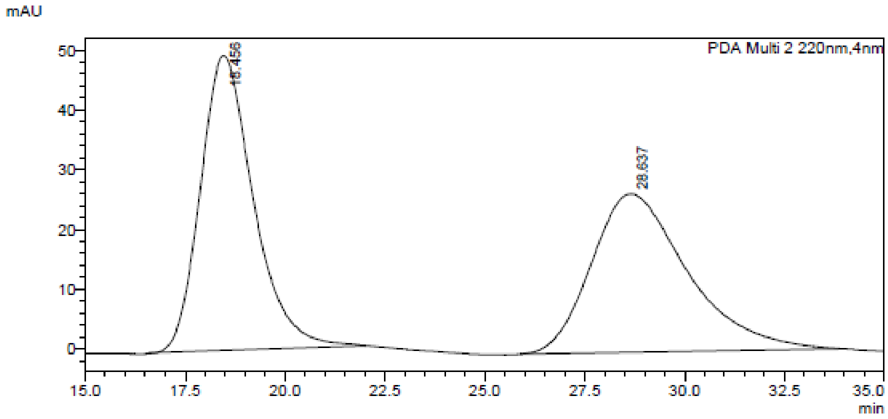

<Peak Table>

PDA Ch2 220nm

Peak#	Ret. Time	Area%
1	18.456	50.664
2	28.637	49.336
Total		100.000

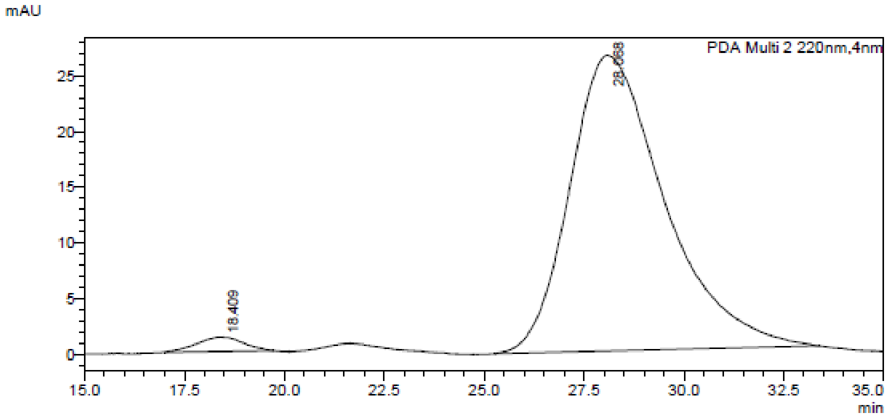

<Peak Table>

PDA Ch2 220nm

Peak#	Ret. Time	Area%
1	18.409	2.357
2	28.068	97.643
Total		100.000

HPLC data for **11a**: Chiralpak IB (97:3 hexane : IPA, flow rate 1.5 mLmin^{-1}, 211 nm, 40 °C) t_R major: 16.5 min, t_R minor: 28.2 min, 98.5:1.5 er

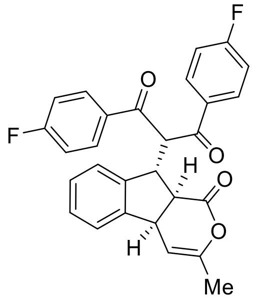

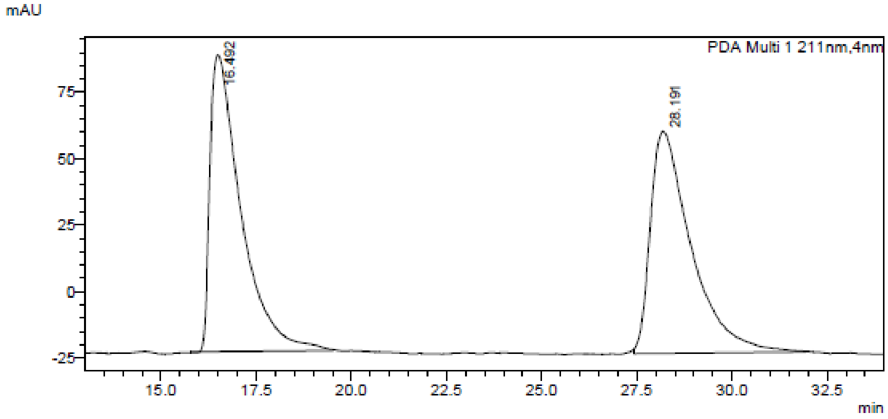

<Peak Table>

PDA Ch1 211nm

Peak#	Ret. Time	Area%
1	16.492	50.307
2	28.191	49.693
Total		100.000

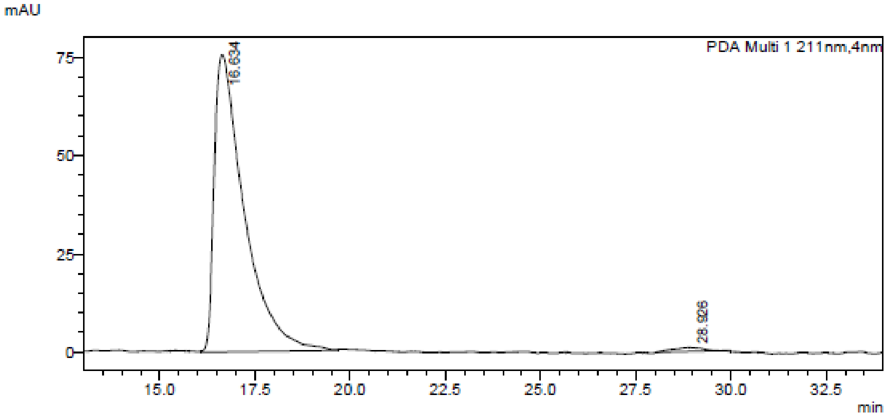

<Peak Table>

PDA Ch1 211nm

Peak#	Ret. Time	Area%
1	16.634	98.642
2	28.926	1.358
Total		100.000

HPLC data for **12a**: Chiralpak AS-H (90:10 hexane : IPA, flow rate 1.5 mLmin^{-1}, 211 nm, 40 °C) t_R minor: 12.1 min, t_R major: 21.8 min, 98:2 er

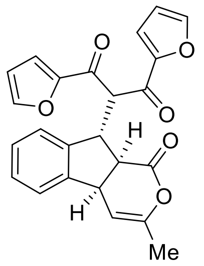

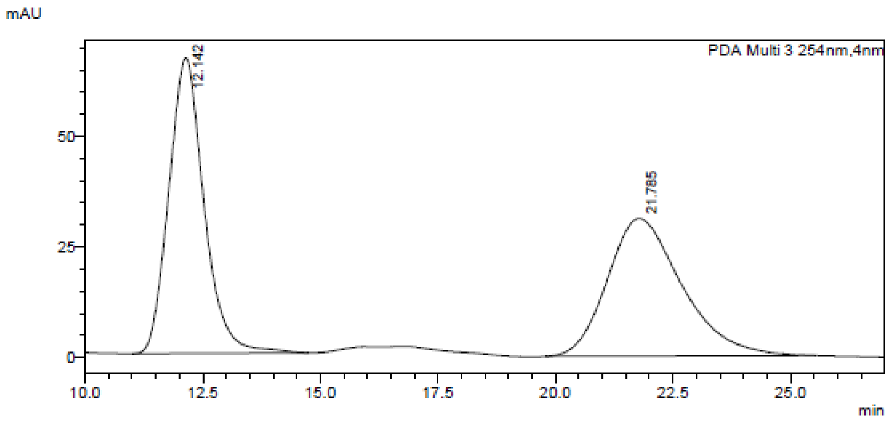

<Peak Table>

PDA Ch3 254nm

Peak#	Ret. Time	Area%
1	12.142	50.362
2	21.785	49.638
Total		100.000

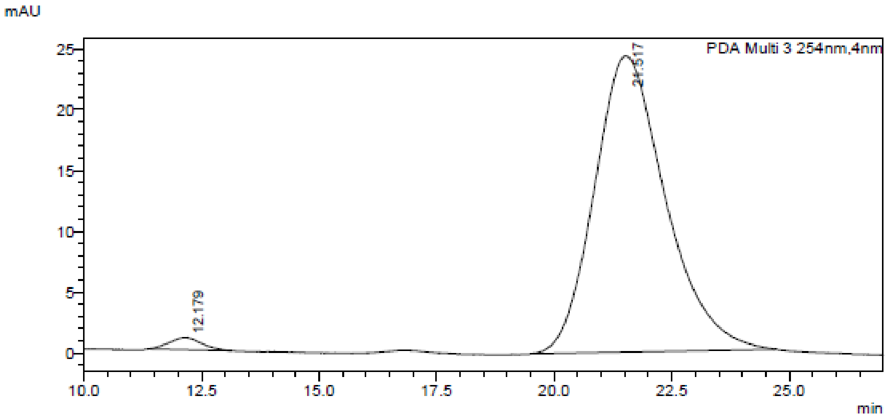

<Peak Table>

PDA Ch3 254nm

Peak#	Ret. Time	Area%
1	12.179	1.833
2	21.517	98.167
Total		100.000

HPLC data for **13a**: Chiralpak AS-H (97:3 hexane : IPA, flowrate 1.0 mLmin^{-1}, 211 nm, 40 °C) t_R major: 11.9 min, t_R minor: 15.9 min, 62.5:37.5 er

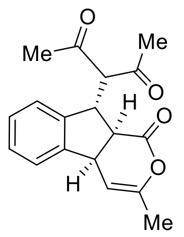

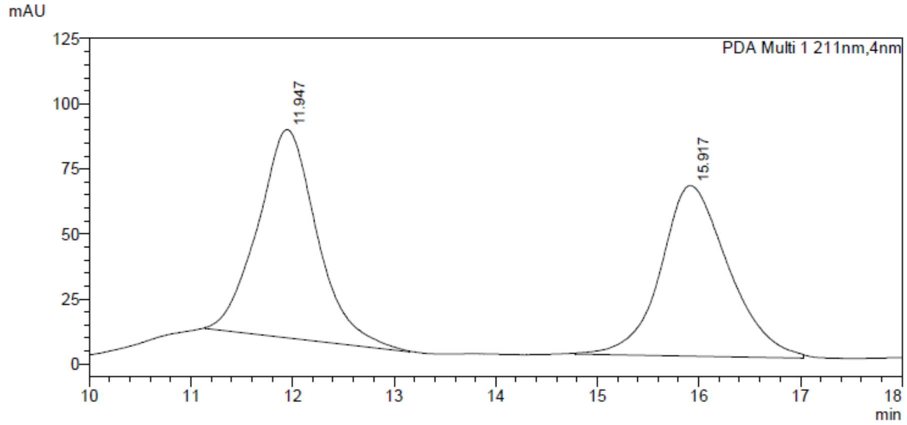

<Peak Table>

PDA Ch1 211nm

Peak#	Ret. Time	Area%
1	11.947	51.562
2	15.917	48.438
Total		100.000

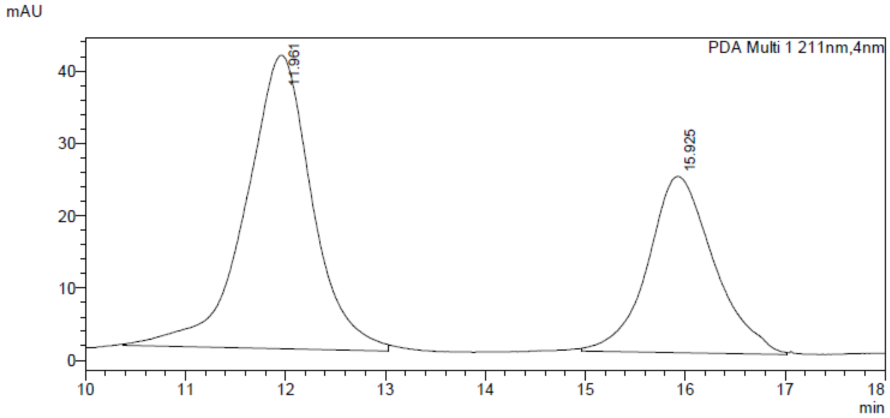

<Peak Table>

PDA Ch1 211nm

Peak#	Ret. Time	Area%
1	11.961	62.656
2	15.925	37.344
Total		100.000

HPLC data for **14a**: Chiralpak OJ-H (97:3 hexane : IPA, flowrate 1.0 mLmin^{-1}, 220 nm, 40 °C) t_R minor: 28.7 min, t_R major: 33.5 min, 73:27 er

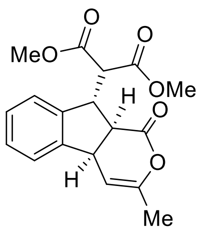

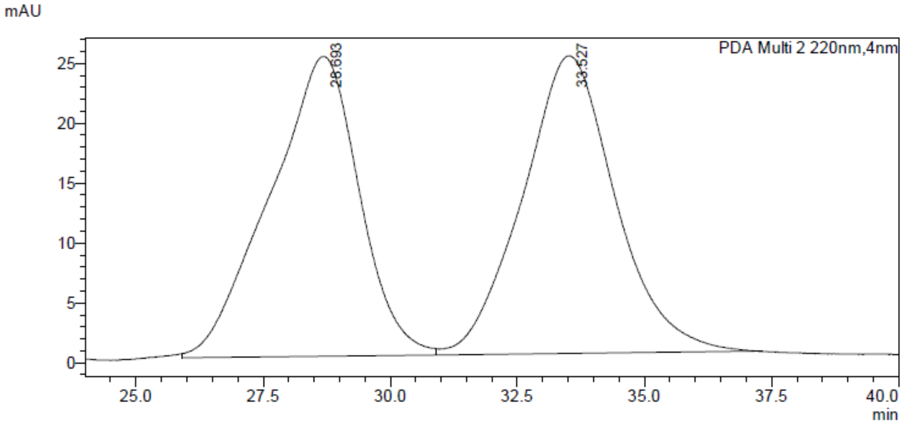

<Peak Table>

PDA Ch2 220nm

Peak#	Ret. Time	Area%
1	28.693	49.240
2	33.527	50.760
Total		100.000

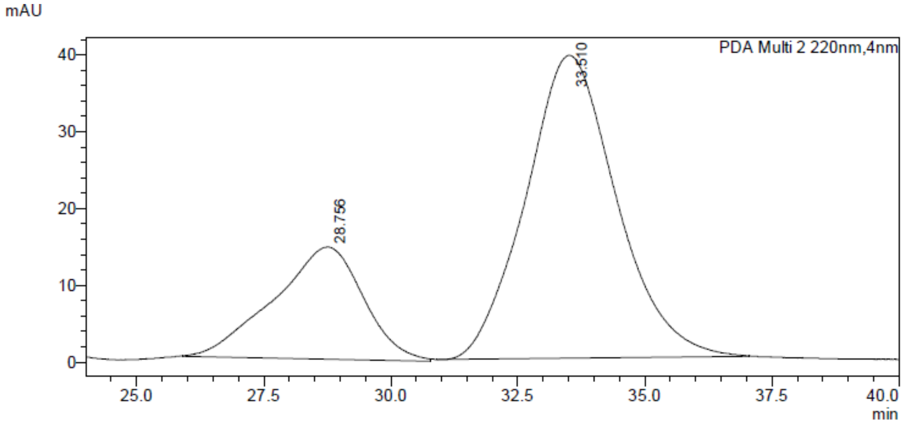

<Peak Table>

PDA Ch2 220nm

Peak#	Ret. Time	Area%
1	28.756	26.769
2	33.510	73.231
Total		100.000

HPLC data for **15a**: Chiralpak OJ-H (98:2 hexane : IPA, flowrate 0.5 mLmin^{-1}, 211 nm, 40 °C) t_R minor: 15.3 min, t_R major: 16.3 min, 82:18 er

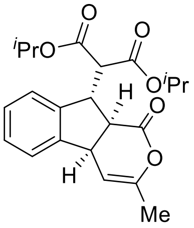

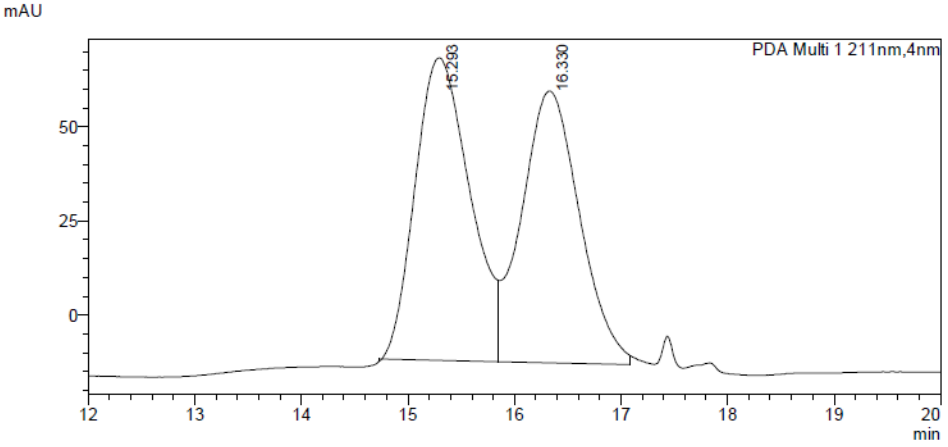

<Peak Table>

PDA Ch1 211nm

Peak#	Ret. Time	Area%
1	15.293	51.399
2	16.330	48.601
Total		100.000

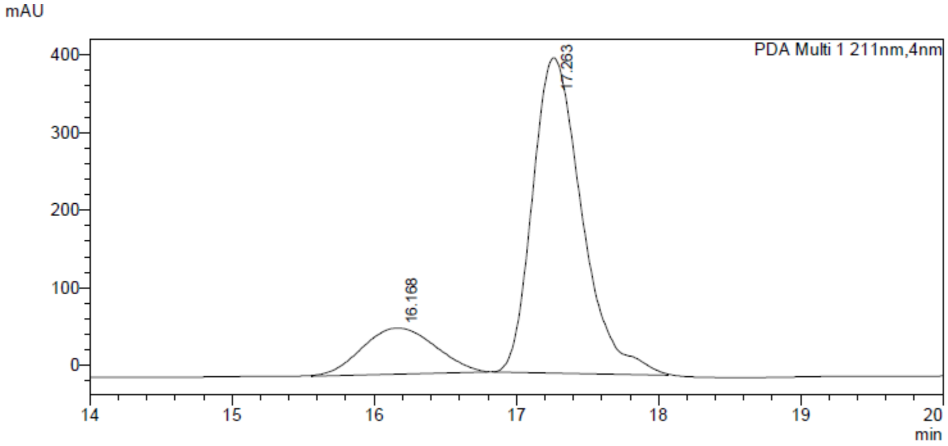

<Peak Table>

PDA Ch1 211nm

Peak#	Ret. Time	Area%
1	16.168	17.854
2	17.263	82.146
Total		100.000

HPLC data for **17a**: Chiralpak IB (97:3 hexane : IPA, flow rate 1.0 mLmin^{-1}, 211 nm, 40 °C) t_R major: 18.7 min, t_R minor: 31.3 min, 99:1 er

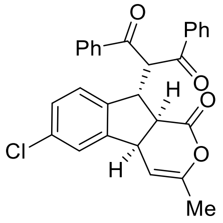

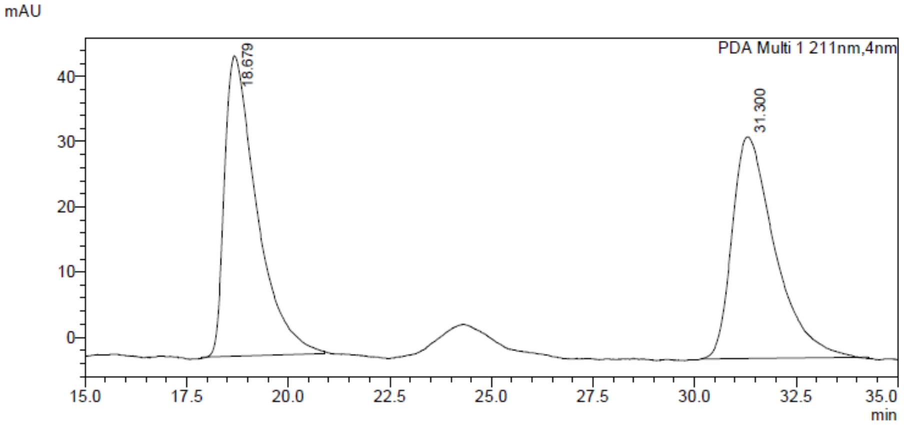

<Peak Table>

PDA Ch1 211nm

Peak#	Ret. Time	Area%
1	18.679	50.599
2	31.300	49.401
Total		100.000

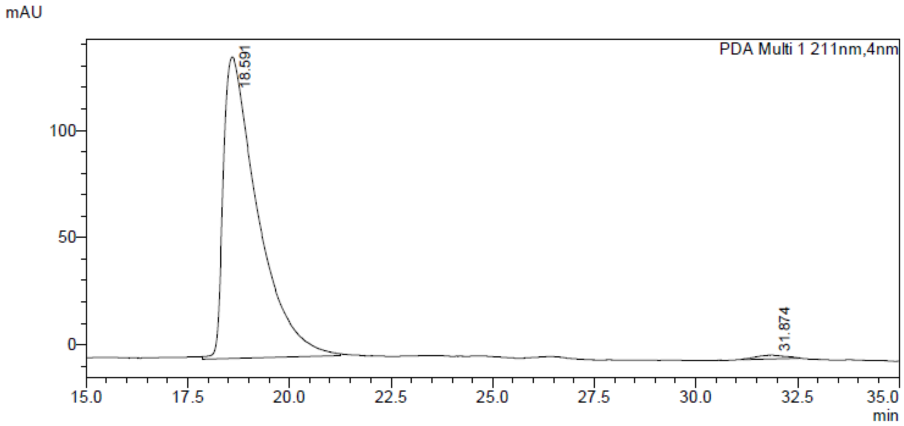

<Peak Table>

PDA Ch1 211nm

Peak#	Ret. Time	Area%
1	18.591	98.890
2	31.874	1.110
Total		100.000

HPLC data for **18a**: Chiralpak IB (97:3 hexane : IPA, flow rate 1.5 mLmin^{-1}, 211 nm, 40 °C) t_R major: 18.6 min, t_R minor: 30.7 min, 98:2 er

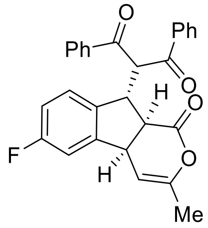

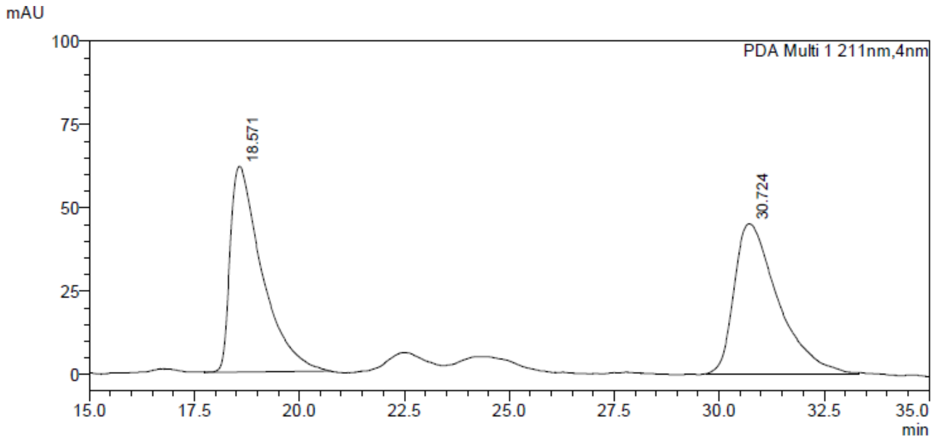

<Peak Table>

PDA Ch1 211nm

Peak#	Ret. Time	Area%
1	18.571	49.910
2	30.724	50.090
Total		100.000

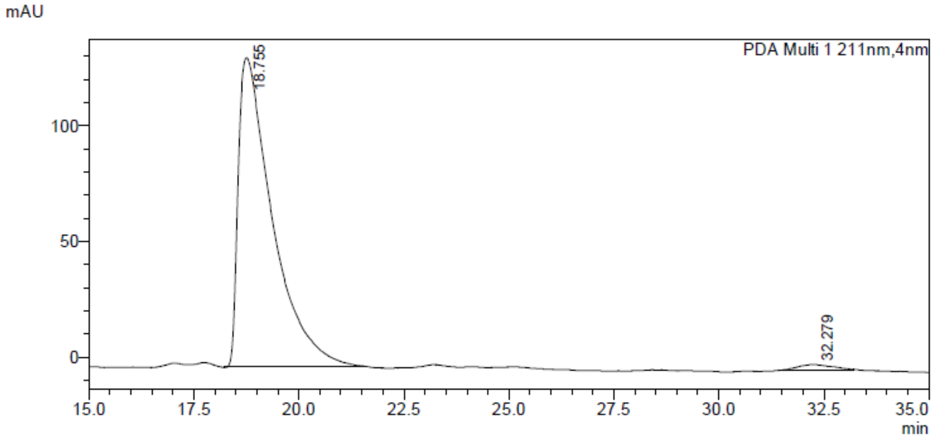

<Peak Table>

PDA Ch1 211nm

Peak#	Ret. Time	Area%
1	18.755	97.876
2	32.279	2.124
Total		100.000

HPLC data for **19a**: Chiralpak IB (98:2 hexane : IPA, flow rate 1.5 mLmin^{-1}, 211 nm, 40 °C) t_R major: 22.8 min, t_R minor: 33.6 min, >99:1 er

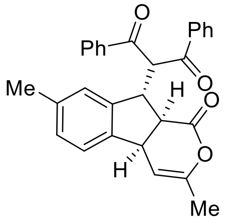

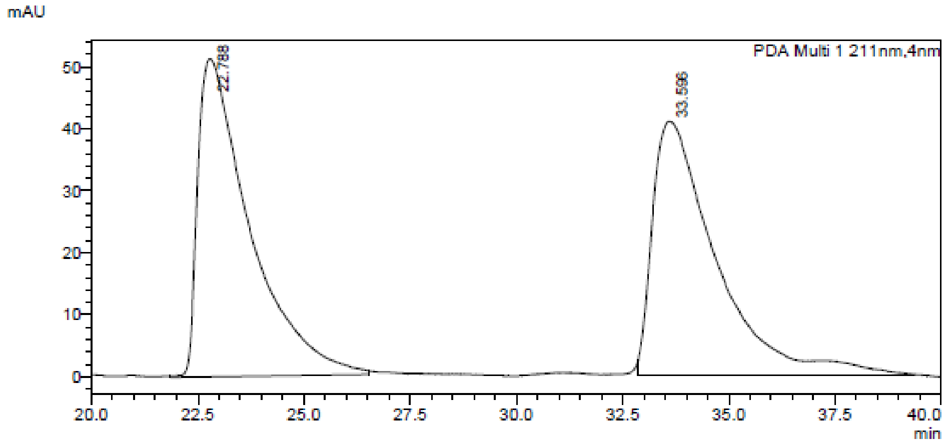

<Peak Table>

PDA Ch1 211nm

Peak#	Ret. Time	Area%
1	22.788	50.745
2	33.596	49.255
Total		100.000

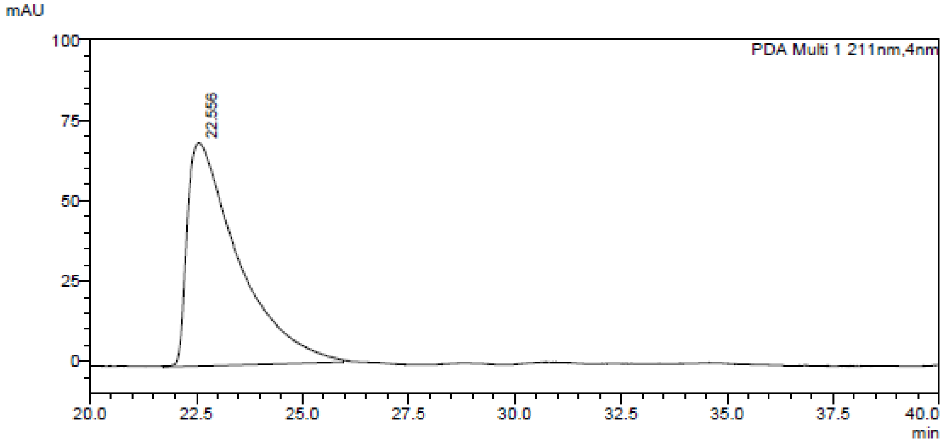

<Peak Table>

PDA Ch1 211nm

Peak#	Ret. Time	Area%
1	22.556	100.000
Total		100.000

HPLC data for **20a**: Chiralpak IB (97:3 hexane : IPA, flow rate 1.5 mLmin^{-1}, 211 nm, 40 °C) t_R major: 10.1 min, t_R minor: 12.1 min, 96.5:3.5 er

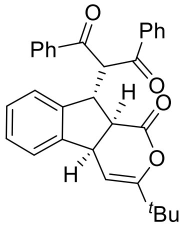

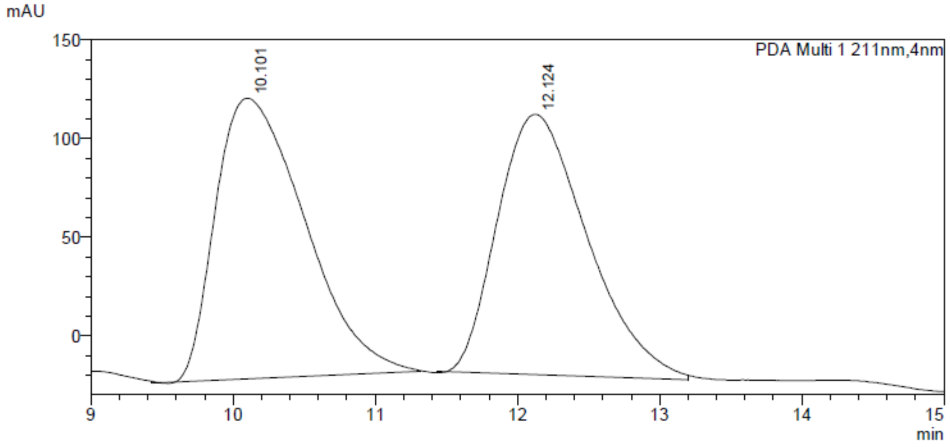

<Peak Table>

PDA Ch1 211nm

Peak#	Ret. Time	Area%
1	10.101	51.727
2	12.124	48.273
Total		100.000

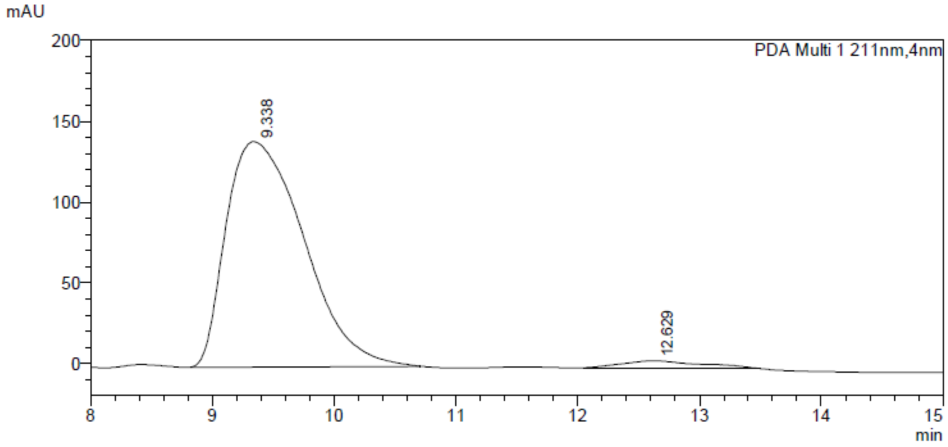

<Peak Table>

PDA Ch1 211nm

Peak#	Ret. Time	Area%
1	9.338	96.641
2	12.629	3.359
Total		100.000

HPLC data for **21a**: Chiralpak IB (97:3 hexane : IPA, flow rate 1.5 mLmin^{-1}, 211 nm, 40 °C) t_R major: 26.9 min, t_R minor: 40.3 min, >99:1 er

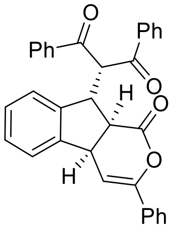

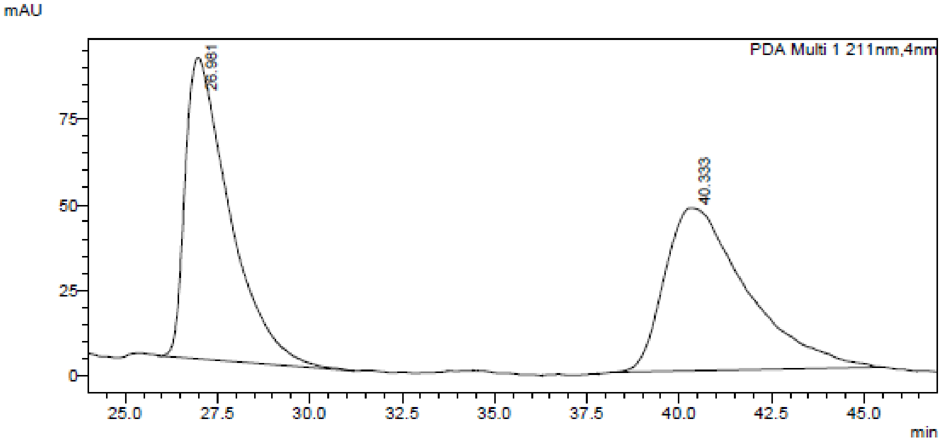

<Peak Table>

PDA Ch1 211nm

Peak#	Ret. Time	Area%
1	26.981	50.064
2	40.333	49.936
Total		100.000

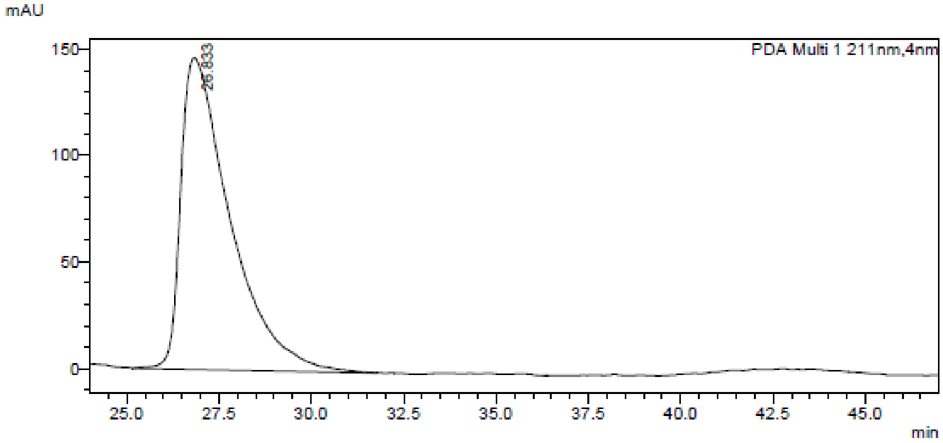

<Peak Table>

PDA Ch1 211nm

Peak#	Ret. Time	Area%
1	26.833	100.000
Total		100.000

HPLC data for **22a**: Chiralpak AS-H (90:10 hexane : IPA, flow rate 1.0 mLmin^{-1}, 211 nm, 40 °C) t_R major: 36.5 min, t_R minor: 43.1 min, >99:1 er

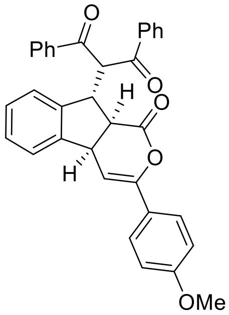

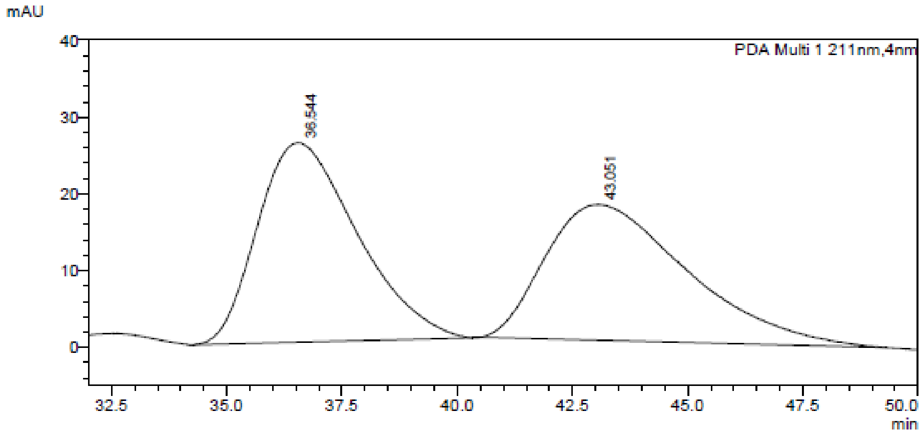

<Peak Table>

PDA Ch1 211nm

Peak#	Ret. Time	Area%
1	36.544	50.751
2	43.051	49.249
Total		100.000

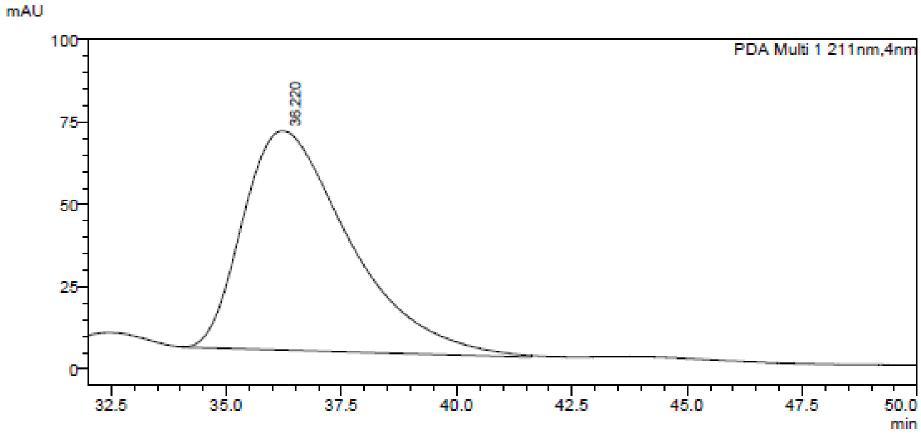

<Peak Table>

PDA Ch1 211nm

Peak#	Ret. Time	Area%
1	36.220	100.000
Total		100.000

HPLC data for **23a**: Chiralpak AS-H (90:10 hexane : IPA, flow rate 1.0 mLmin^{-1}, 211 nm, 40 °C) t_R major: 18.9 min, t_R minor: 23.5 min, 97:3 er

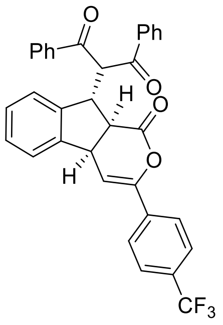

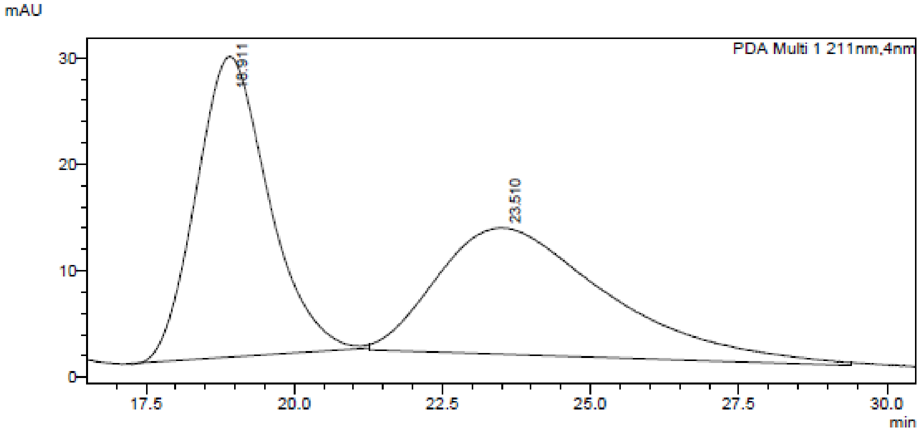

<Peak Table>

PDA Ch1 211nm

Peak#	Ret. Time	Area%
1	18.911	50.160
2	23.510	49.840
Total		100.000

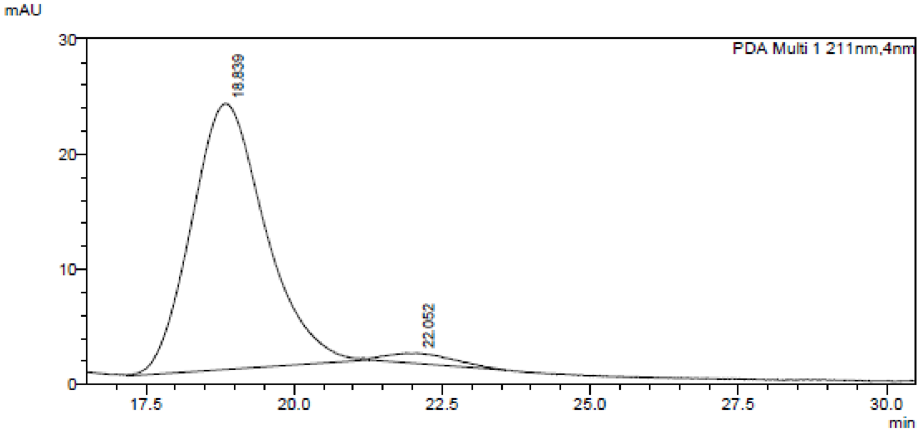

<Peak Table>

PDA Ch1 211nm

Peak#	Ret. Time	Area%
1	18.839	96.688
2	22.052	3.312
Total		100.000

HPLC data for **24a**: Chiralpak AS-H (90:10 hexane : IPA, flow rate 1.0 mLmin^{-1}, 211 nm, 40 °C) t_R major: 27.6 min, t_R minor: 33.3 min, 97:3 er

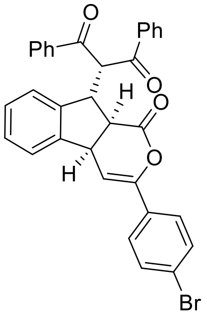

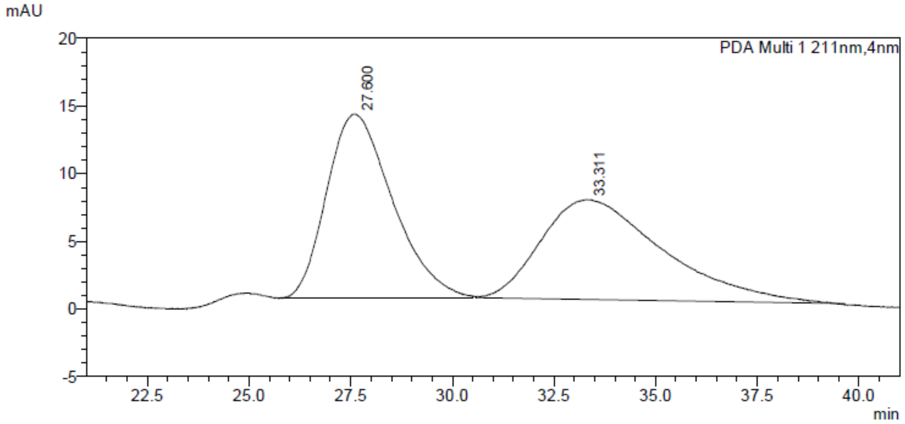

<Peak Table>

PDA Ch1 211nm

Peak#	Ret. Time	Area%
1	27.600	49.806
2	33.311	50.194
Total		100.000

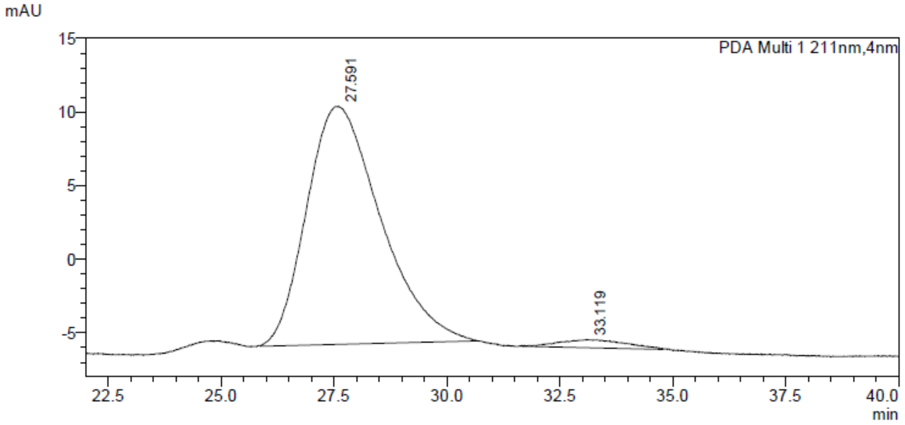

<Peak Table>

PDA Ch1 211nm

Peak#	Ret. Time	Area%
1	27.591	96.935
2	33.119	3.065
Total		100.000

HPLC data for **25a**: Chiralpak AS-H (90:10 hexane : IPA, flow rate 1.0 mLmin^{-1}, 211 nm, 40 °C) t_R major: 25.7 min, t_R minor: 30.7 min, 96:4 er

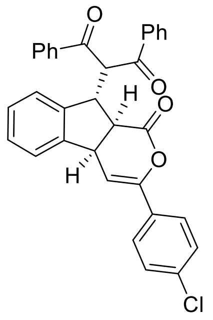

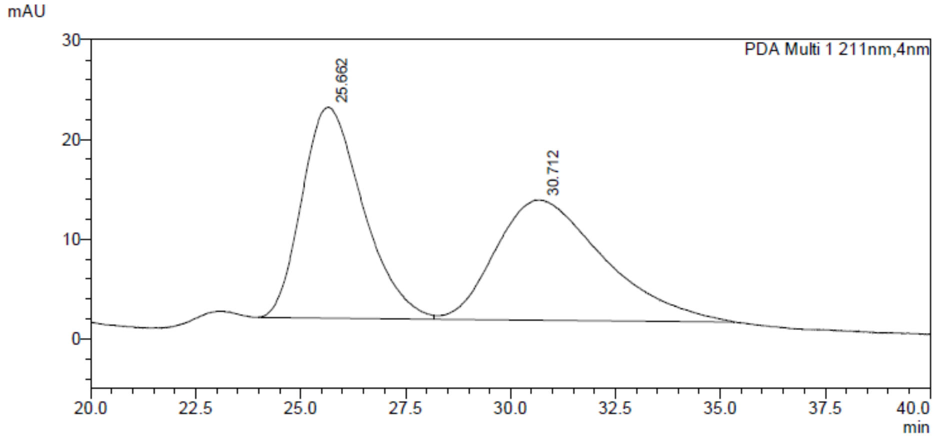

<Peak Table>

PDA Ch1 211nm

Peak#	Ret. Time	Area%
1	25.662	49.732
2	30.712	50.268
Total		100.000

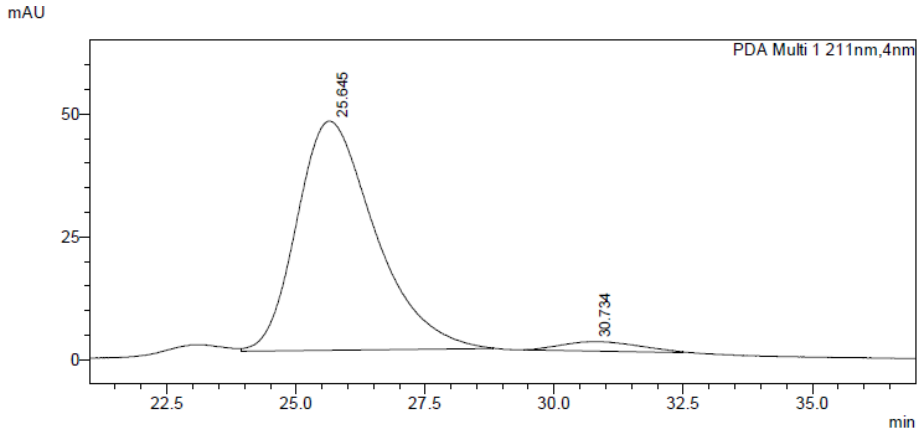

<Peak Table>

PDA Ch1 211nm

Peak#	Ret. Time	Area%
1	25.645	96.051
2	30.734	3.949
Total		100.000

HPLC data for **26a**: Chiralpak AS-H (90:10 hexane : IPA, flow rate 1.5 mLmin^{-1}, 211 nm, 40 °C) t_R minor: 17.5 min, t_R major: 25.3 min, 96.5:3.5 er

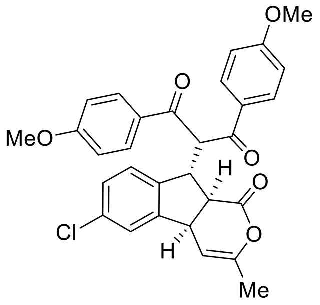

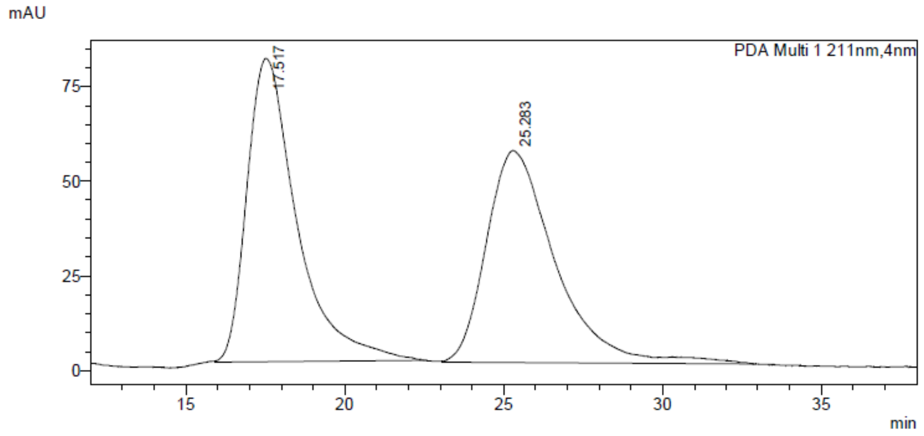

<Peak Table>

PDA Ch1 211nm

Peak#	Ret. Time	Area%
1	17.517	50.811
2	25.283	49.189
Total		100.000

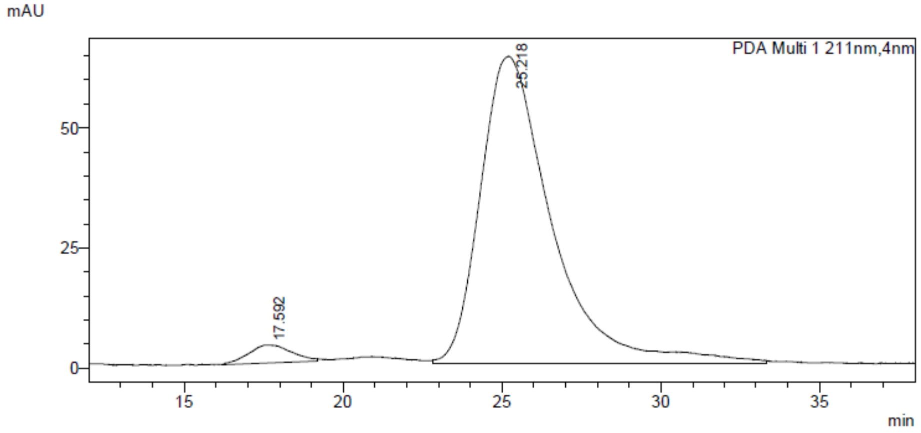

<Peak Table>

PDA Ch1 211nm

Peak#	Ret. Time	Area%
1	17.592	3.432
2	25.218	96.568
Total		100.000

HPLC data for **27a**: Chiralpak IB (97:3 hexane : IPA, flow rate 1.5 mLmin^{-1}, 211 nm, 40 °C) t_R major: 7.5 min, t_R minor: 9.5 min, >99:1 er

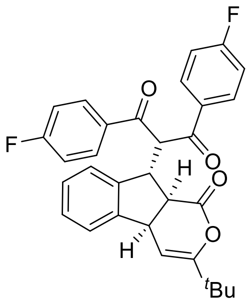

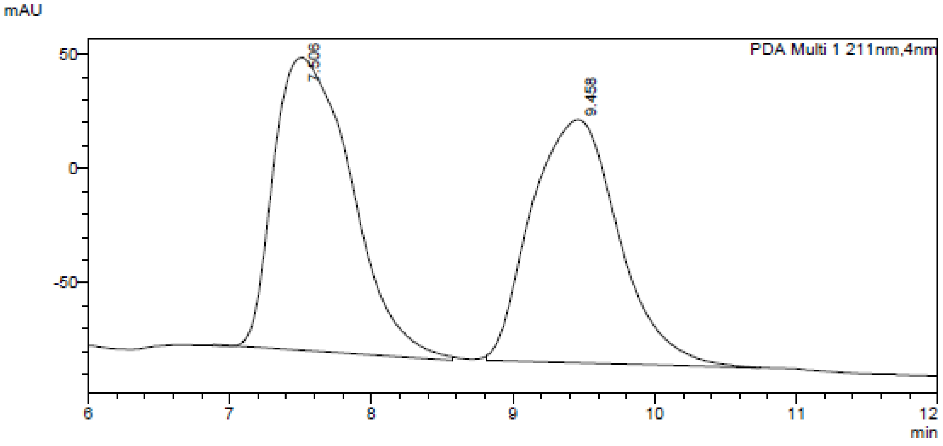

<Peak Table>

PDA Ch1 211nm

Peak#	Ret. Time	Area%
1	7.506	51.710
2	9.458	48.290
Total		100.000

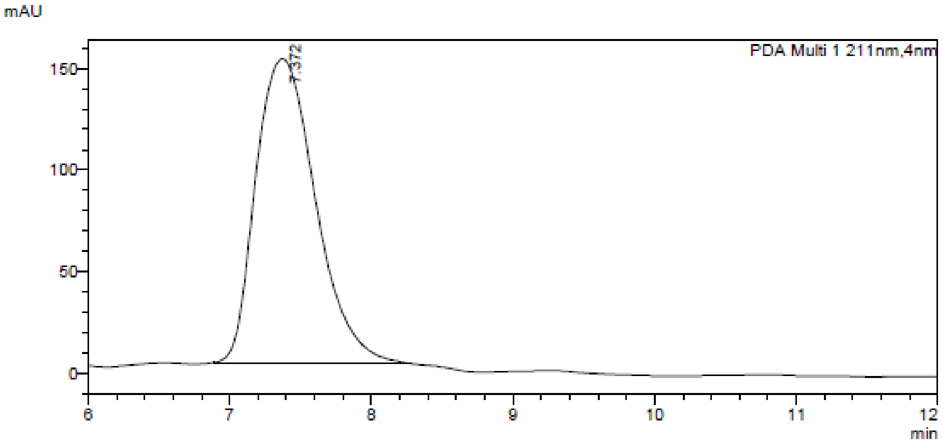

<Peak Table>

PDA Ch1 211nm

Peak#	Ret. Time	Area%
1	7.372	100.000
Total		100.000

HPLC data for **28a**: Chiralpak AS-H (90:10 hexane : IPA, flow rate 1.5 mLmin^{-1}, 211 nm, 40 °C) t_R minor: 11.5 min, t_R major: 18.9 min, 97.5:2.5 er

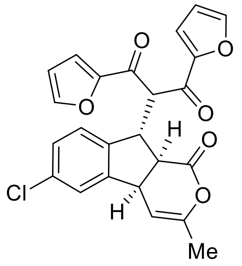

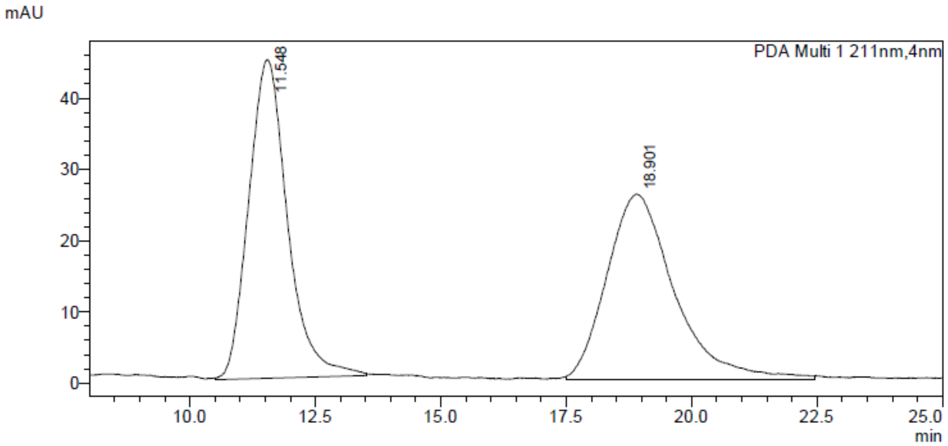

<Peak Table>

PDA Ch1 211nm

Peak#	Ret. Time	Area%
1	11.548	49.688
2	18.901	50.312
Total		100.000

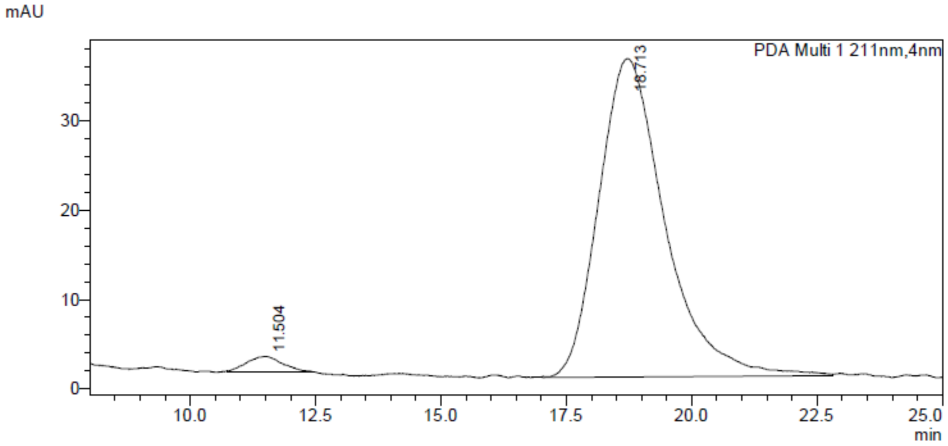

<Peak Table>

PDA Ch1 211nm

Peak#	Ret. Time	Area%
1	11.504	2.539
2	18.713	97.461
Total		100.000

HPLC data for **35a**: Chiralpak IB (95:5 hexane : IPA, flow rate 1.5 mLmin^{-1}, 211 nm, 40 °C) t_R minor: 18.4 min, t_R major: 31.4 min, 94:6 er

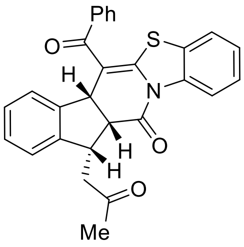

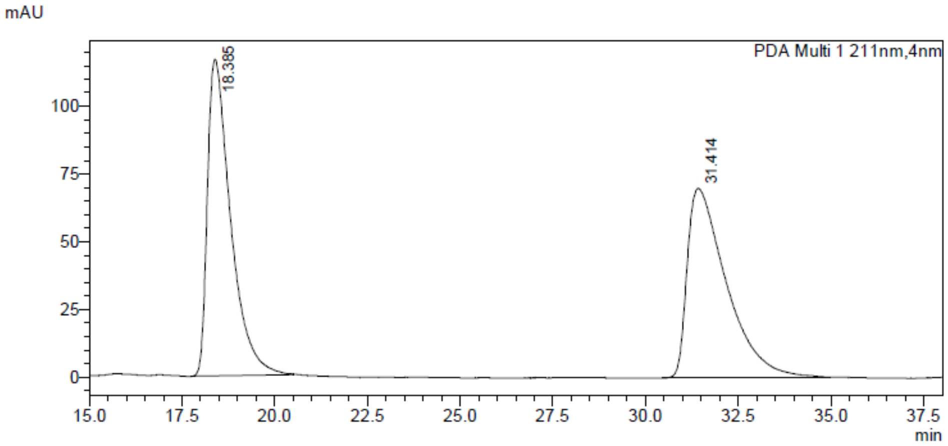

<Peak Table>

PDA Ch1 211nm

Peak#	Ret. Time	Area%
1	18.385	49.587
2	31.414	50.413
Total		100.000

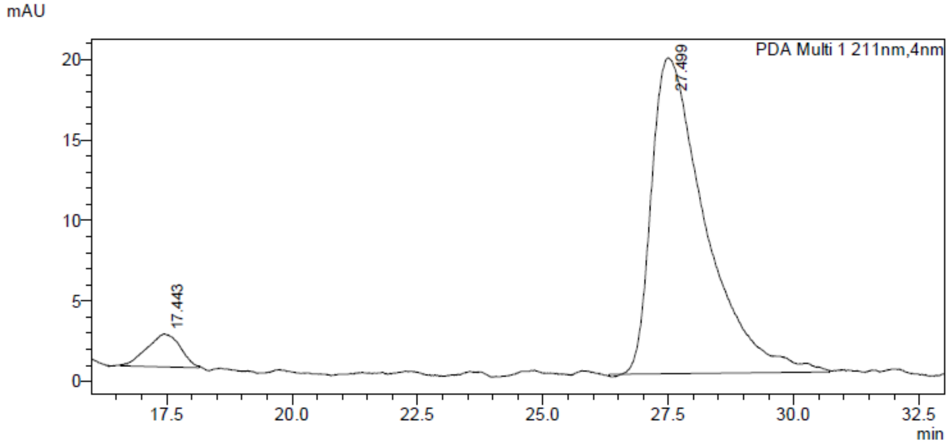

<Peak Table>

PDA Ch1 211nm

Peak#	Ret. Time	Area%
1	17.443	6.097
2	27.499	93.903
Total		100.000

HPLC data for **36a**: Chiralpak IB (97:3 hexane : IPA, flow rate 1.5 mLmin^{-1}, 211 nm, 40 °C) t_R major: 20.6 min, t_R minor: 37.6 min, 82:18 er

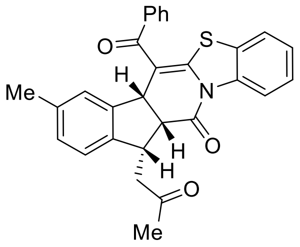

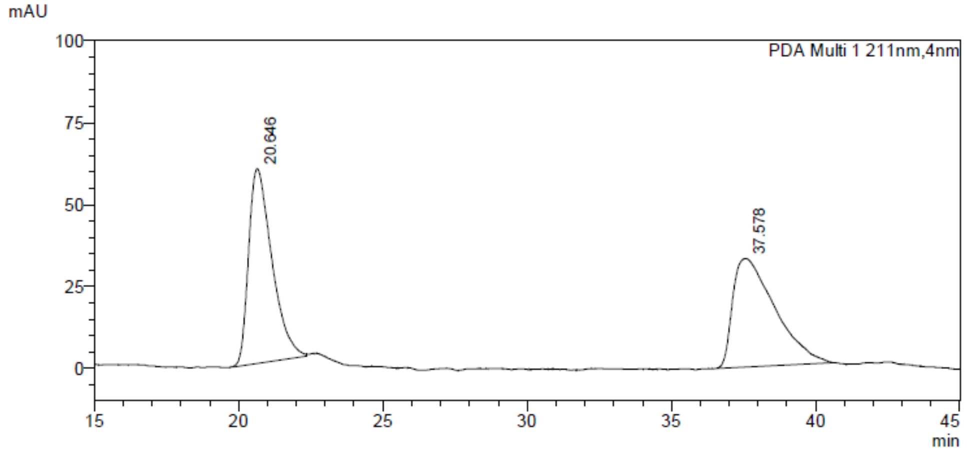

<Peak Table>

PDA Ch1 211nm

Peak#	Ret. Time	Area%
1	20.646	49.849
2	37.578	50.151
Total		100.000

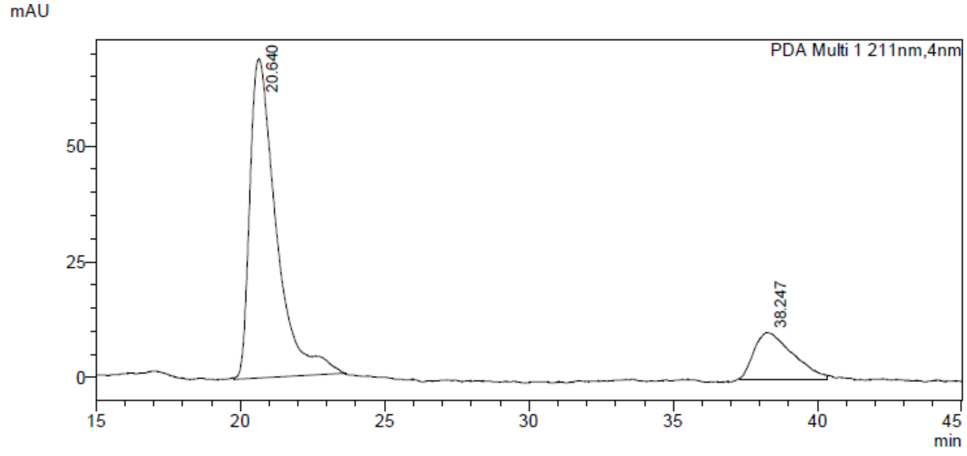

<Peak Table>

PDA Ch1 211nm

Peak#	Ret. Time	Area%
1	20.640	82.352
2	38.247	17.648
Total		100.000

HPLC data for **37a**: Chiralpak AS-H (95:5 hexane : IPA, flow rate 1.0 mLmin^{-1}, 211 nm, 40 °C) t_R minor: 23.4 min, t_R major: 29.4 min, 92:8 er

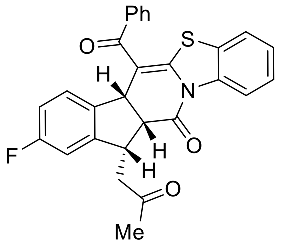

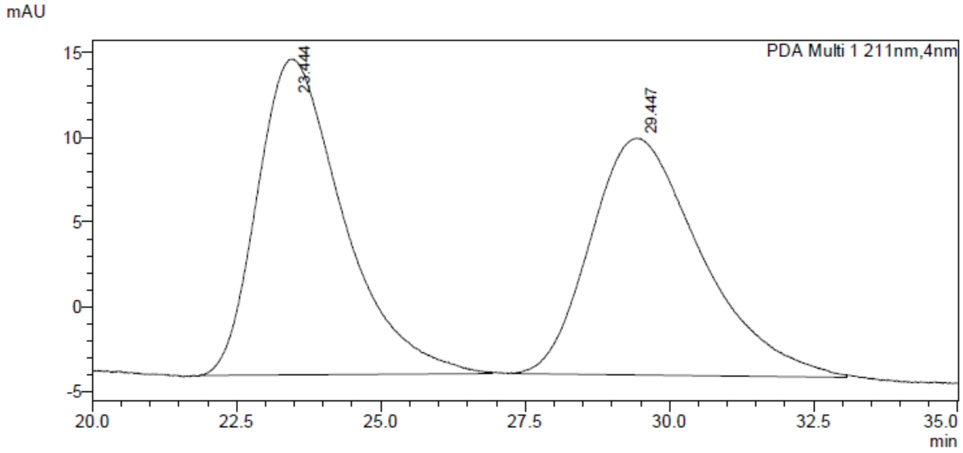

<Peak Table>

PDA Ch1 211nm

Peak#	Ret. Time	Area%
1	23.444	50.843
2	29.447	49.157
Total		100.000

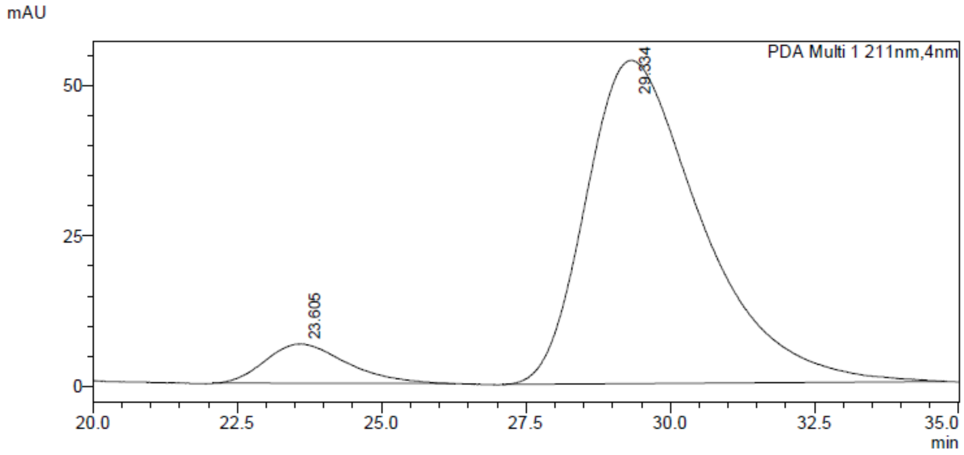

<Peak Table>

PDA Ch1 211nm

Peak#	Ret. Time	Area%
1	23.605	7.926
2	29.334	92.074
Total		100.000

HPLC data for **38a**: Chiralpak AS-H (90:10 hexane : IPA, flow rate 0.5 mLmin^{-1}, 211 nm, 40 °C) t_R major: 38.2 min, t_R minor: 43.3 min, 97:3 er

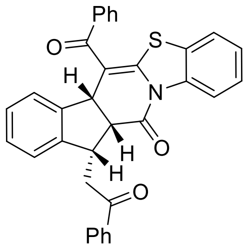

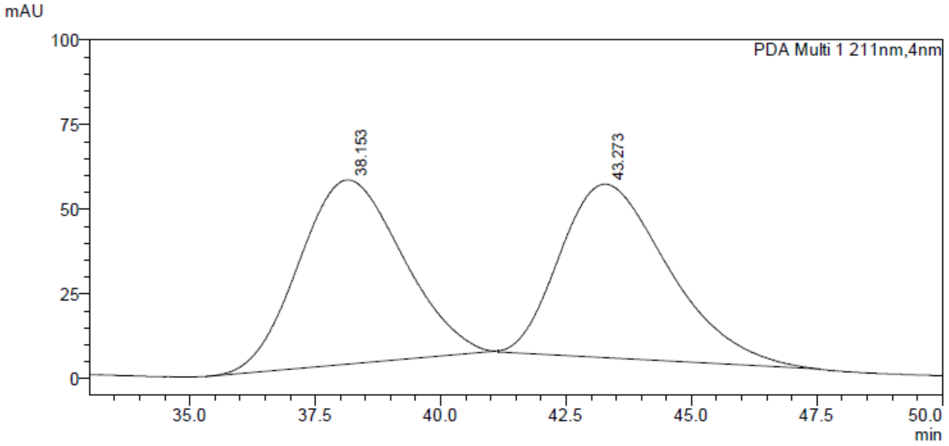

<Peak Table>

PDA Ch1 211nm

Peak#	Ret. Time	Area%
1	38.153	50.320
2	43.273	49.680
Total		100.000

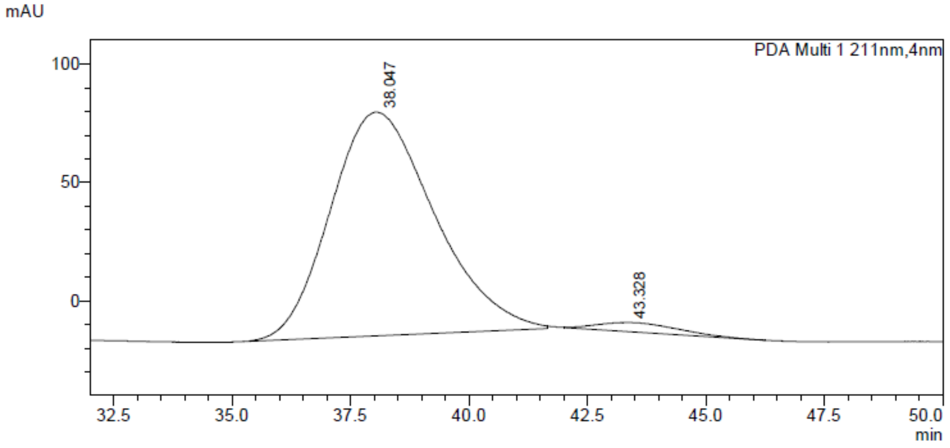

<Peak Table>

PDA Ch1 211nm

Peak#	Ret. Time	Area%
1	38.047	96.841
2	43.328	3.159
Total		100.000

HPLC data for **39a**: Chiralpak ADH (90:10 hexane : IPA, flow rate 1.0 mLmin^{-1}, 211 nm, 40 °C) t_R major: 45.7 min, t_R minor: 50.2 min, 96:4 er

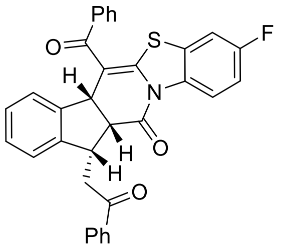

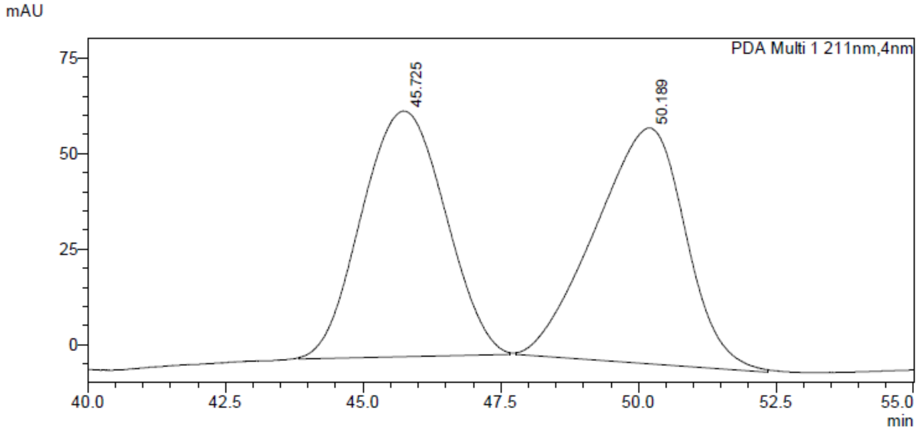

<Peak Table>

PDA Ch1 211nm

Peak#	Ret. Time	Area%
1	45.725	49.019
2	50.189	50.981
Total		100.000

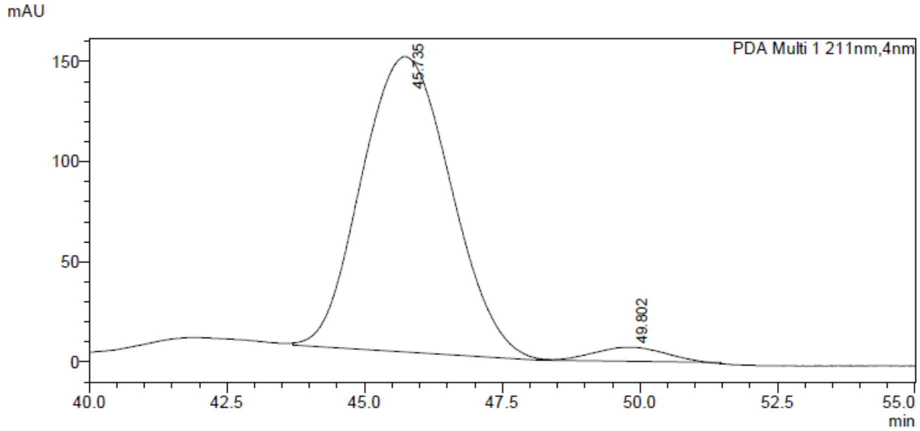

<Peak Table>

PDA Ch1 211nm

Peak#	Ret. Time	Area%
1	45.735	96.378
2	49.802	3.622
Total		100.000

HPLC data for **40a**: Chiralpak ADH (85:15 hexane : IPA, flow rate 1.0 mLmin^{-1}, 211 nm, 40 °C) t_R major: 36.9 min, t_R minor: 43.6 min, 92.5:7.5 er

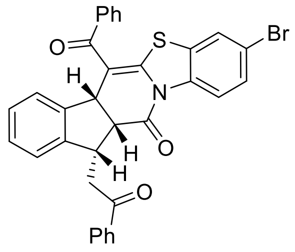

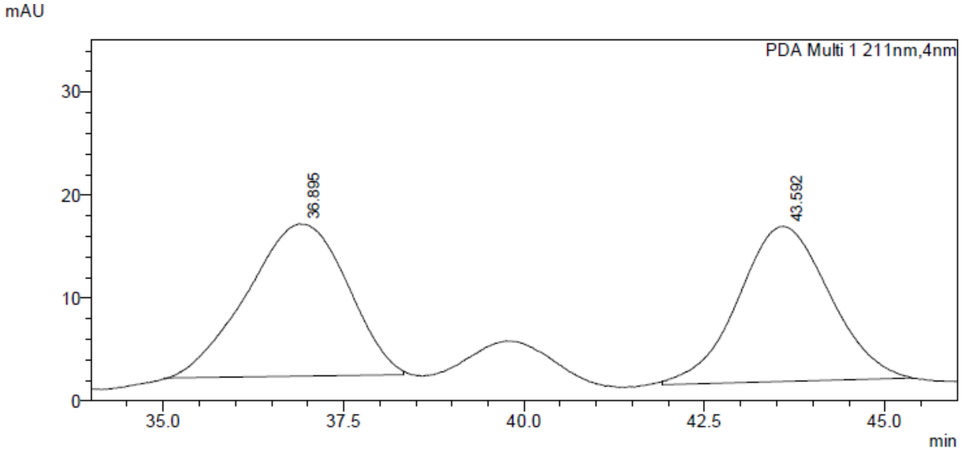

<Peak Table>

PDA Ch1 211nm

Peak#	Ret. Time	Area%
1	36.895	51.823
2	43.592	48.177
Total		100.000

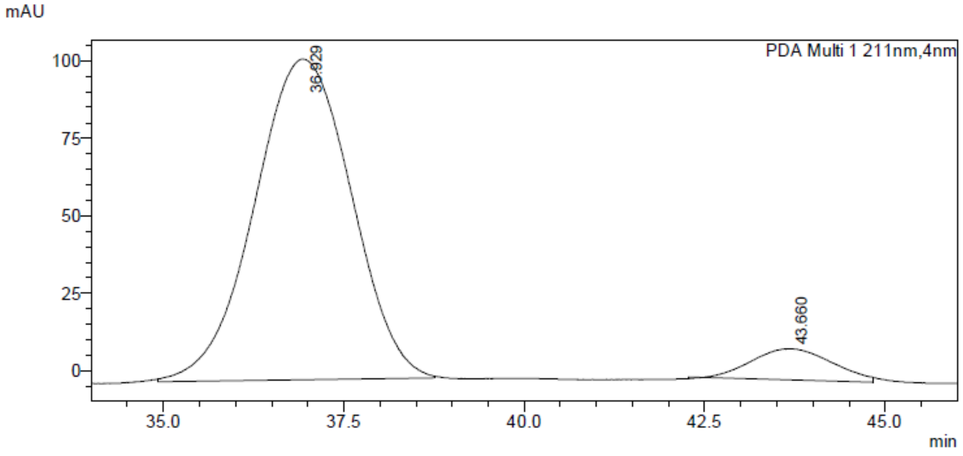

<Peak Table>

PDA Ch1 211nm

Peak#	Ret. Time	Area%
1	36.929	92.565
2	43.660	7.435
Total		100.000

HPLC data for **41a**: Chiralpak IB (93:7 hexane : IPA, flow rate 1.0 mLmin^{-1}, 254 nm, 40 °C) t_R minor: 33.1 min, t_R major: 46.6 min, 91:9 er (recryst. 98:2 er)

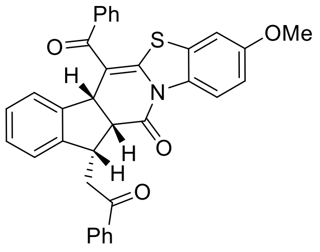

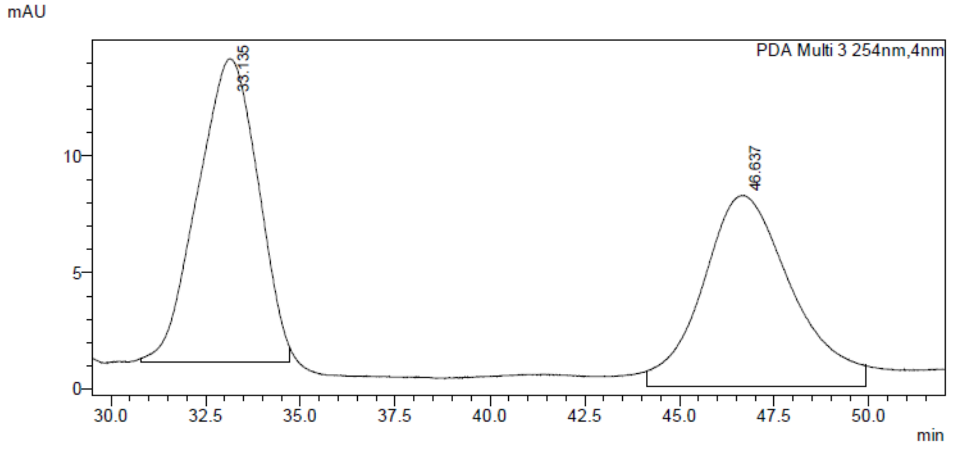

<Peak Table>

PDA Ch3 254nm

Peak#	Ret. Time	Area%
1	33.135	51.772
2	46.637	48.228
Total		100.000

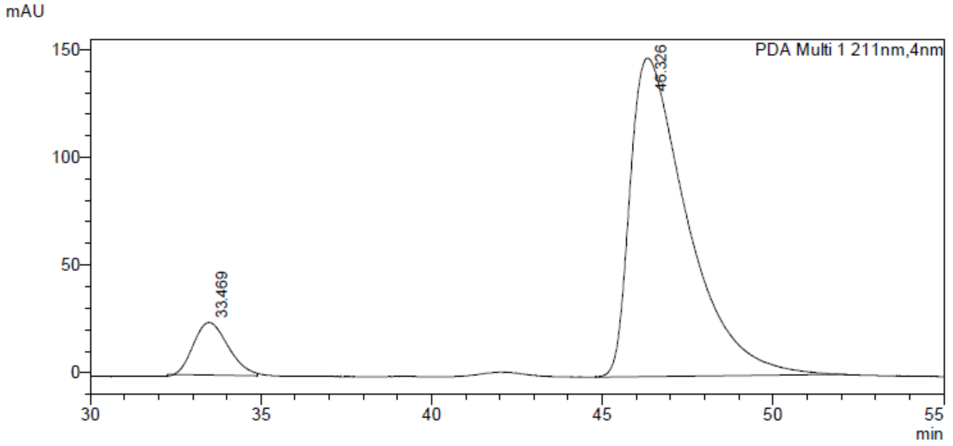

<Peak Table>

PDA Ch1 211nm

Peak#	Ret. Time	Area%
1	33.469	8.995
2	46.326	91.005
Total		100.000

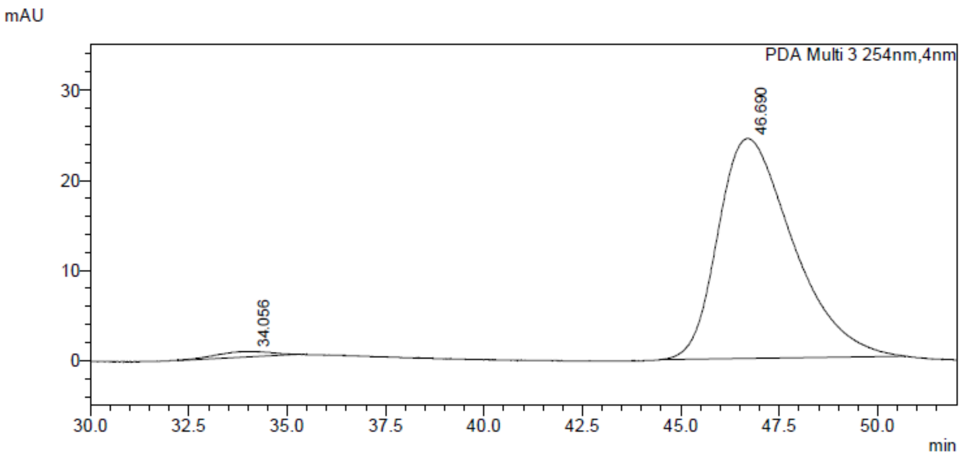

<Peak Table>

PDA Ch3 254nm

Peak#	Ret. Time	Area%
1	34.056	1.836
2	46.690	98.164
Total		100.000

HPLC data for **42a**: Chiralpak IB (95:5 hexane : IPA, flow rate 1.5 mLmin^{-1}, 211 nm, 40 °C) t_R major: 23.8 min, t_R minor: 37.1 min, 94.5:5.5 er

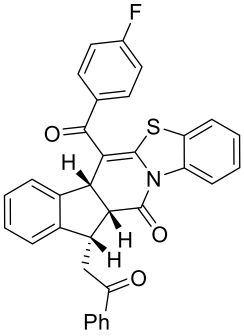

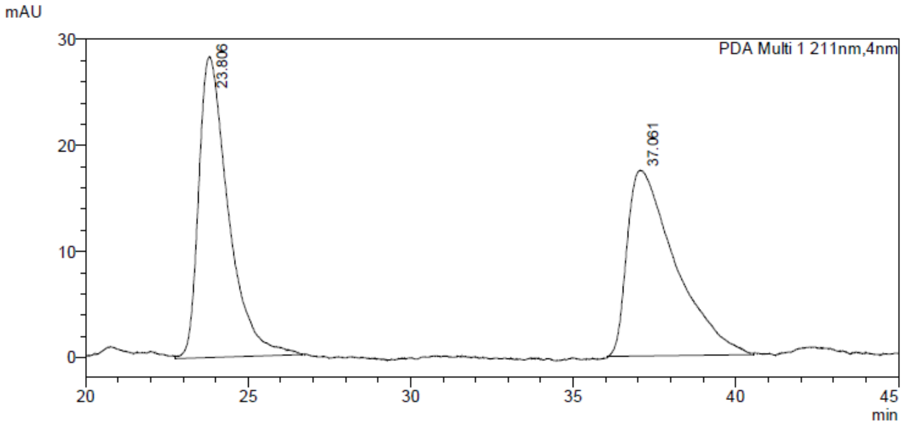

<Peak Table>

PDA Ch1 211nm

Peak#	Ret. Time	Area%
1	23.806	49.818
2	37.061	50.182
Total		100.000

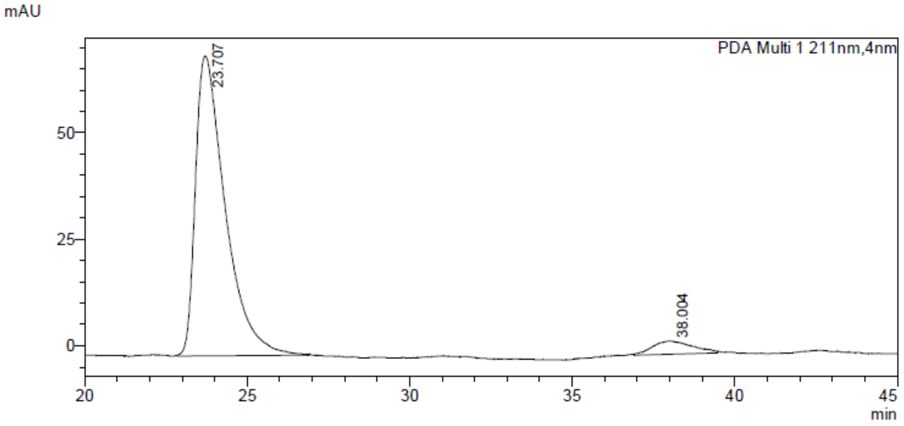

<Peak Table>

PDA Ch1 211nm

Peak#	Ret. Time	Area%
1	23.707	94.532
2	38.004	5.468
Total		100.000

HPLC data for **43a**: Chiralpak IB (95:5 hexane : IPA, flow rate 1.5 $mLmin^{-1}$, 211 nm, 40 °C) t_R major: 26.6 min, t_R minor: 39.8 min, 92:8 er

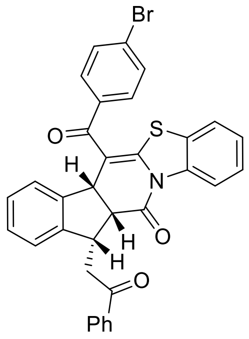

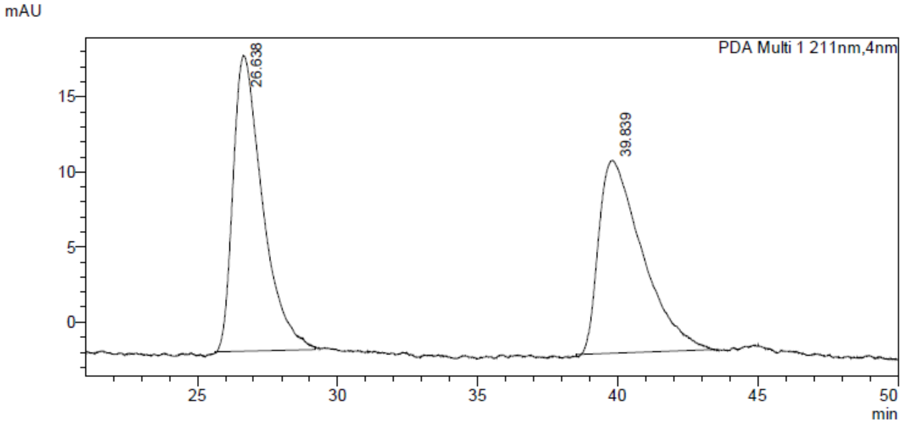

<Peak Table>

PDA Ch1 211nm

Peak#	Ret. Time	Area%
1	26.638	50.936
2	39.839	49.064
Total		100.000

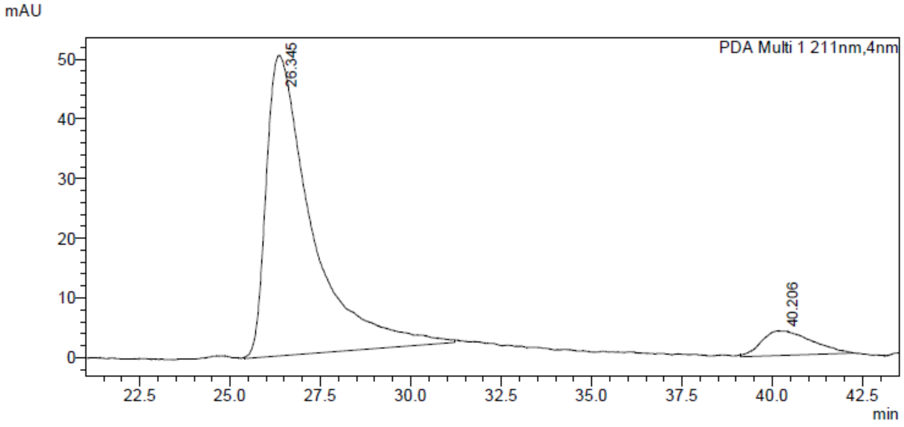

<Peak Table>

PDA Ch1 211nm

Peak#	Ret. Time	Area%
1	26.345	91.835
2	40.206	8.165
Total		100.000

HPLC data for **44a**:Chiralpak AS-H (90:10 hexane : IPA, flow rate 0.5 mLmin^{-1}, 220 nm, 40 °C) t_R major: 53.3 min, t_R minor: 60.7 min, 94.5:5.5 er

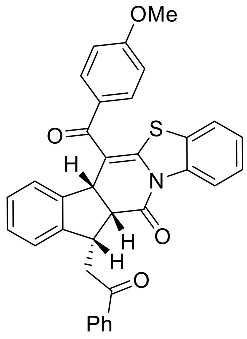

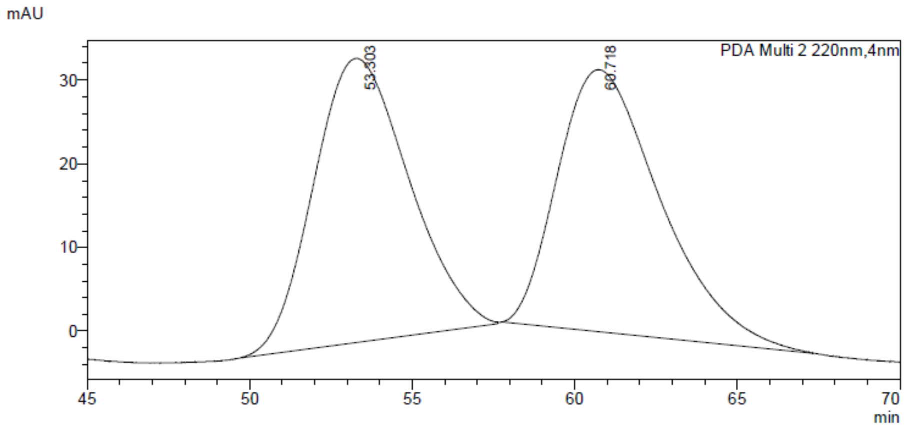

<Peak Table>

PDA Ch2 220nm

Peak#	Ret. Time	Area%
1	53.303	50.078
2	60.718	49.922
Total		100.000

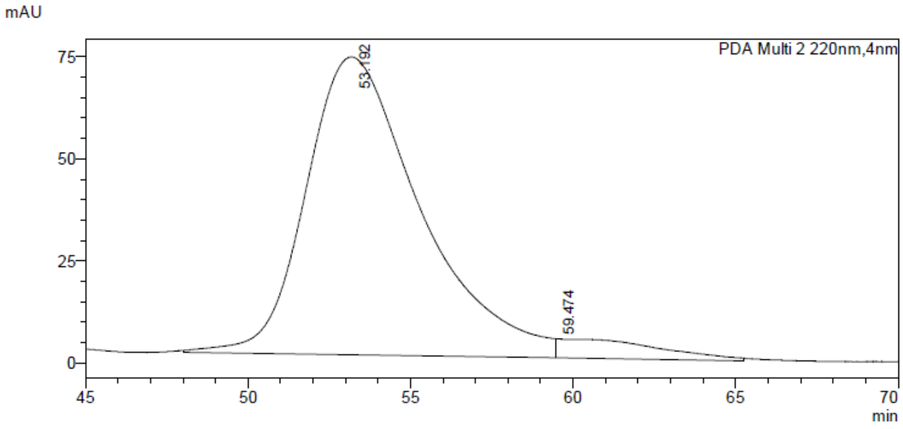

<Peak Table>

PDA Ch2 220nm

Peak#	Ret. Time	Area%
1	53.192	94.553
2	59.474	5.447
Total		100.000

HPLC data for **51**: Chiralpak AS-H (95:5 hexane : IPA, flow rate 1.5 mLmin^{-1}, 220 nm, 40 °C) t_R major: 23.3 min, t_R minor: 31.5 min, 88:12 er

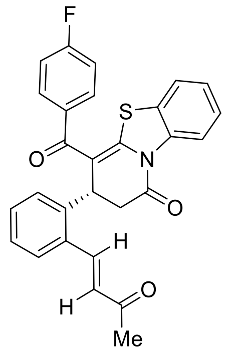

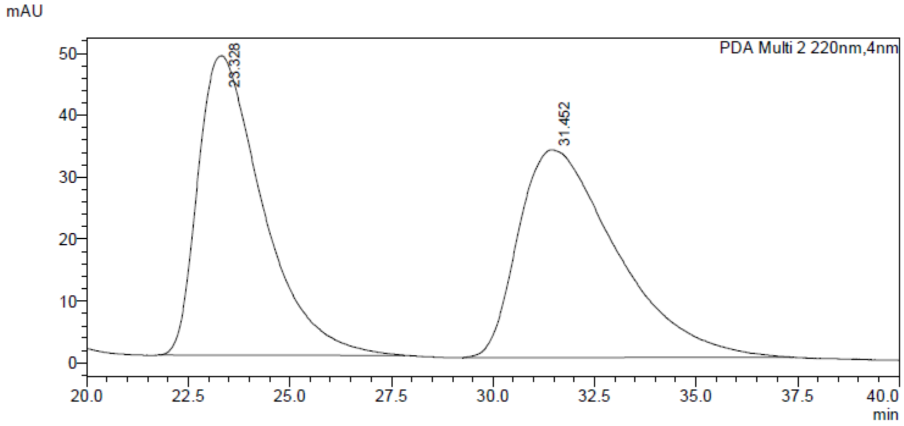

<Peak Table>

PDA Ch2 220nm

Peak#	Ret. Time	Area%
1	23.328	48.698
2	31.452	51.302
Total		100.000

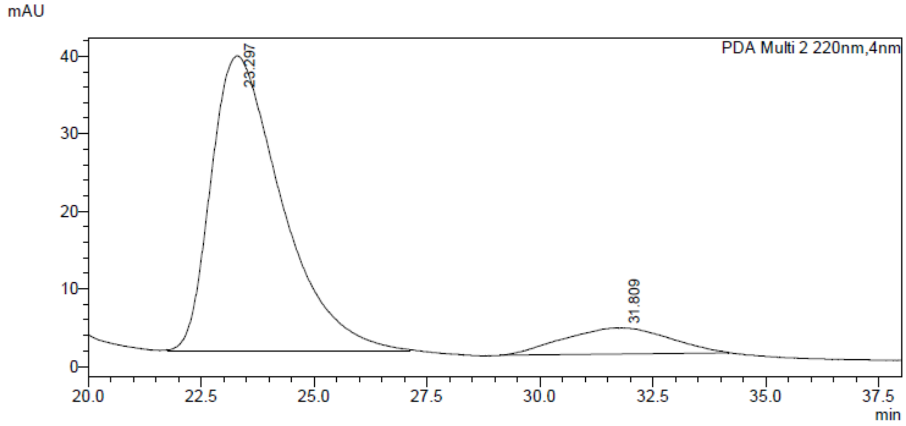

<Peak Table>

PDA Ch2 220nm

Peak#	Ret. Time	Area%
1	23.297	88.346
2	31.809	11.654
Total		100.000

HPLC data for **52**: Chiralpak IB (97:3 hexane : IPA, flow rate 1.5 mLmin^{-1}, 211 nm, 40 °C) t_R major: 25.9 min, t_R minor: 43.1 min, 92:8 er

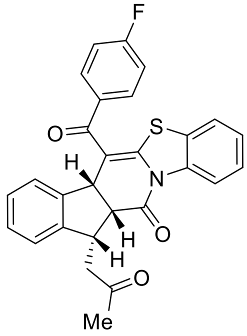

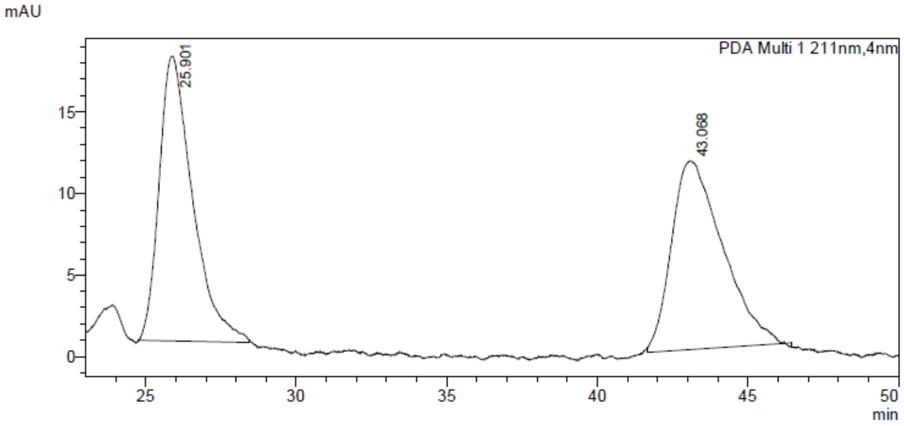

<Peak Table>

PDA Ch1 211nm

Peak#	Ret. Time	Area%
1	25.901	49.780
2	43.068	50.220
Total		100.000

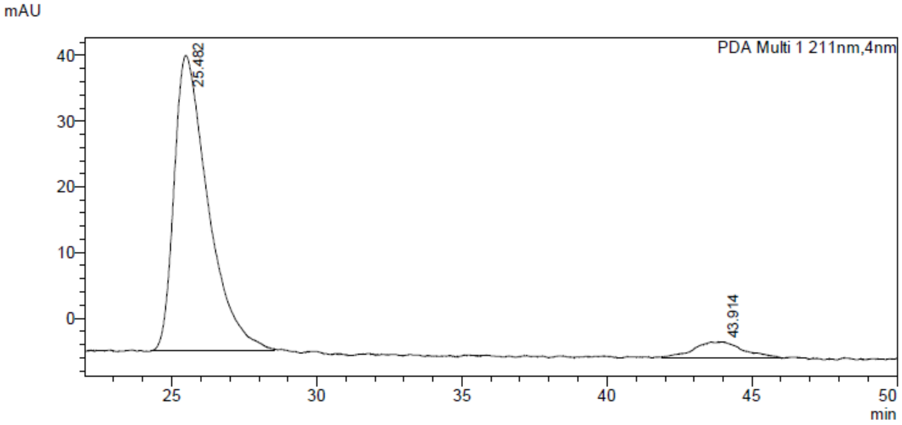

<Peak Table>

PDA Ch1 211nm

Peak#	Ret. Time	Area%
1	25.482	92.374
2	43.914	7.626
Total		100.000

HPLC data for **54**: Chiralpak IA (97:3 hexane : IPA, flow rate 1.5 mLmin^{-1}, 211 nm, 40 °C) t_R major: 14.8 min, t_R minor: 16.6 min, 88:12 er

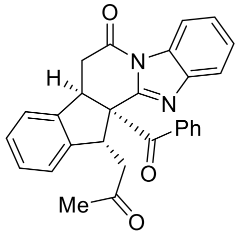

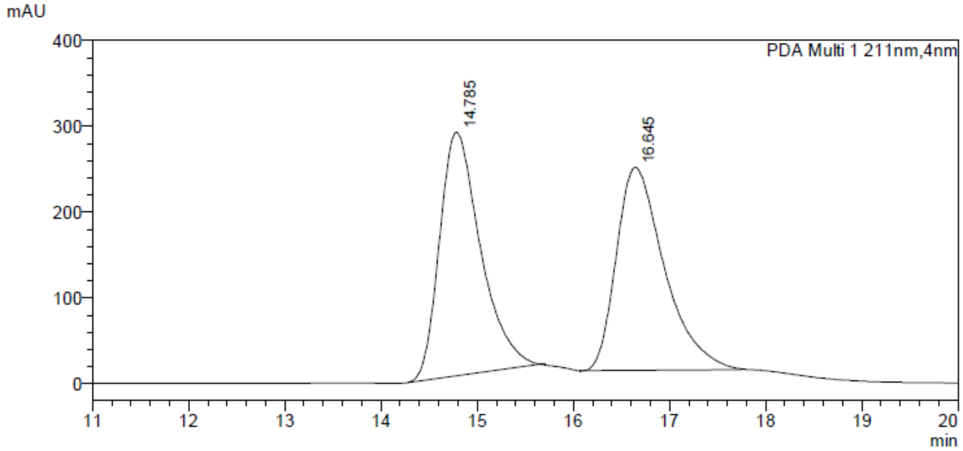

<Peak Table>

PDA Ch1 211nm

Peak#	Ret. Time	Area%
1	14.785	50.572
2	16.645	49.428
Total		100.000

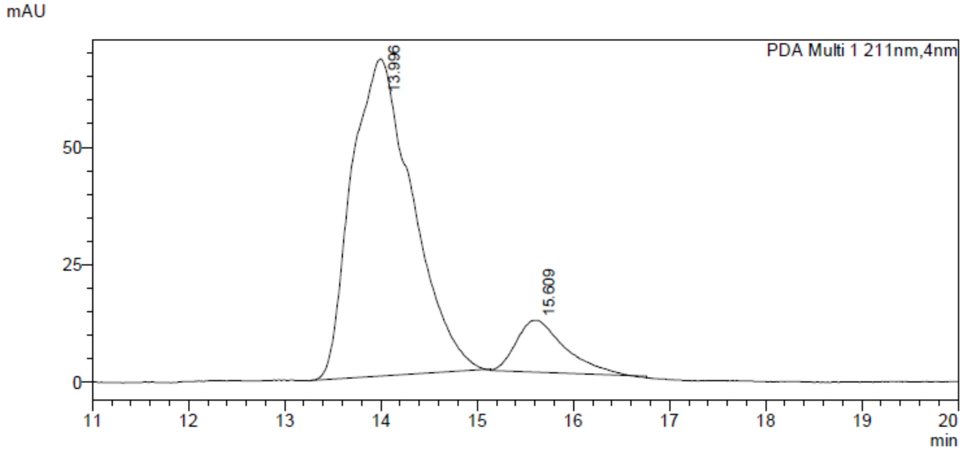

<Peak Table>

PDA Ch1 211nm

Peak#	Ret. Time	Area%
1	13.996	88.413
2	15.609	11.587
Total		100.000

HPLC data for **55**: Chiralpak IA (98:2 hexane : IPA, flow rate 1.5 mLmin^{-1}, 211 nm, 40 °C) t_R minor: 29.8 min, t_R major: 34.0 min, 85:15 er (after single recryst. 98.5:1.5 er)

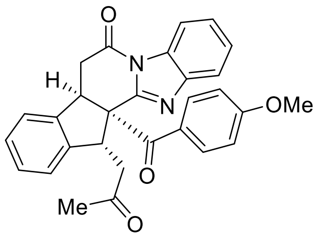

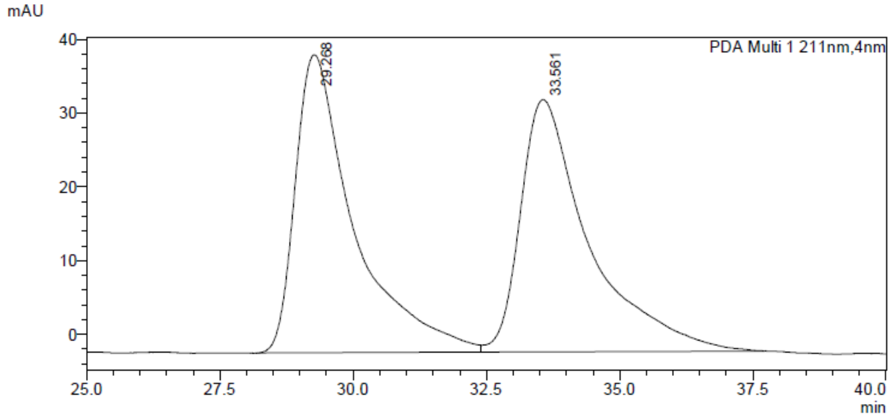

<Peak Table>

PDA Ch1 211nm

Peak#	Ret. Time	Area%
1	29.268	50.293
2	33.561	49.707
Total		100.000

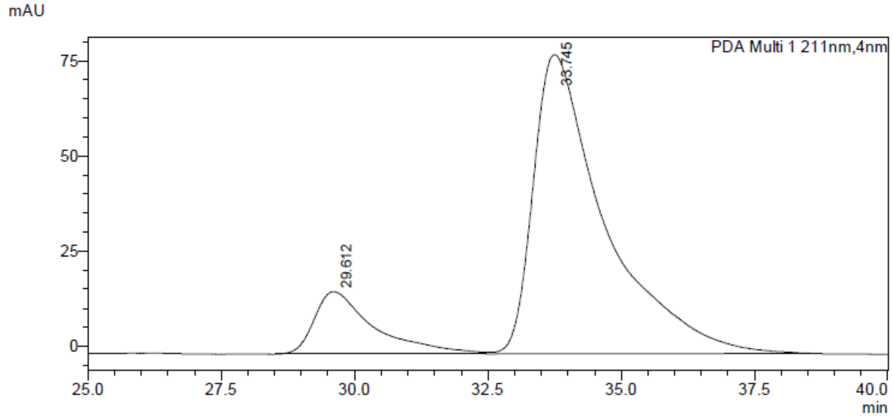

<Peak Table>

PDA Ch1 211nm

Peak#	Ret. Time	Area%
1	29.612	14.828
2	33.745	85.172
Total		100.000

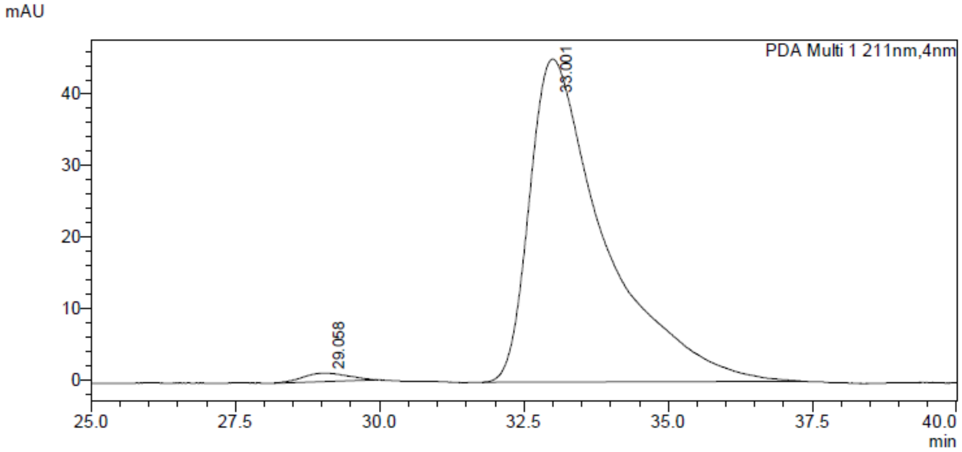

<Peak Table>

PDA Ch1 211nm

Peak#	Ret. Time	Area%
1	29.058	1.521
2	33.001	98.479
Total		100.000

HPLC data for **65**: Chiralpak IA (97.5:2.5 hexane : IPA, flow rate 0.5 mLmin^{-1}, 211 nm, 40 °C) t_R minor: 19.5 min, t_R major: 20.8 min, 81:19 er

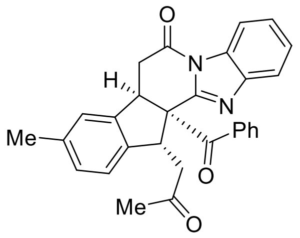

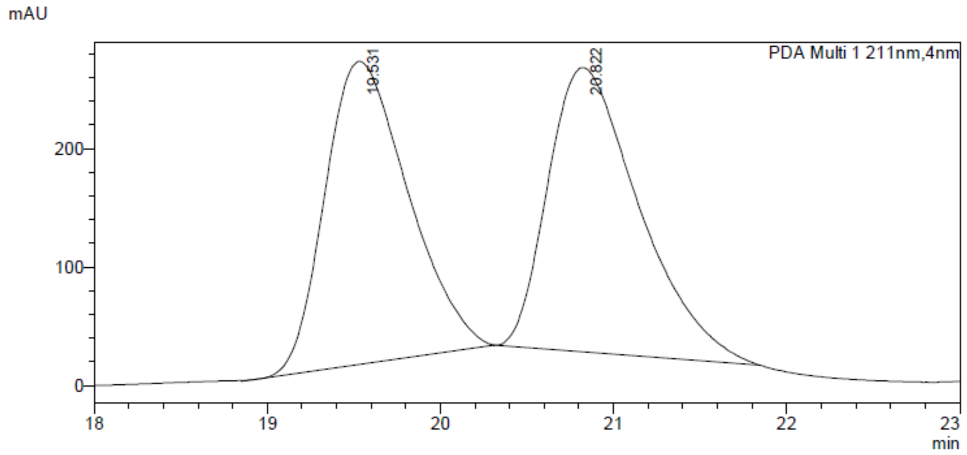

<Peak Table>

PDA Ch1 211nm

Peak#	Ret. Time	Area%
1	19.531	49.320
2	20.822	50.680
Total		100.000

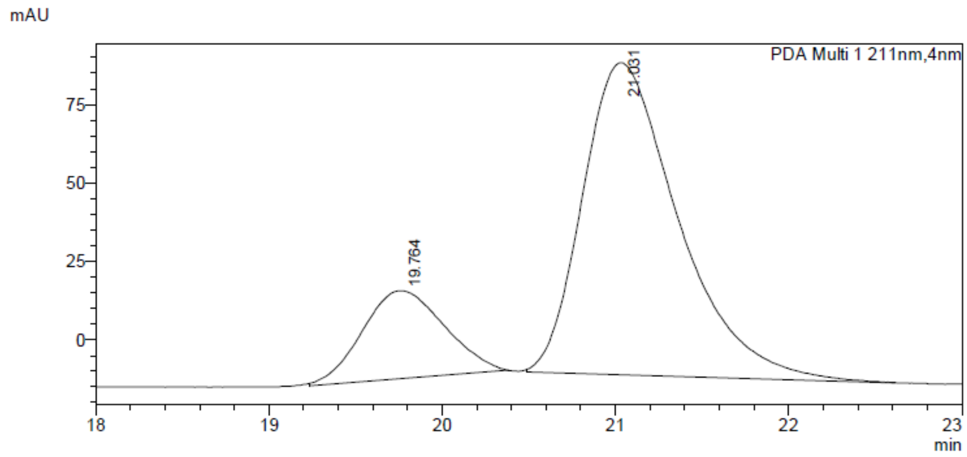

<Peak Table>

PDA Ch1 211nm

Peak#	Ret. Time	Area%
1	19.764	19.394
2	21.031	80.606
Total		100.000

HPLC data for **67**: Chiralpak IA (99:1 hexane : IPA, flow rate 1.0 mLmin^{-1}, 211 nm, 40 °C) t_R major: 16.2 min, t_R minor: 19.8 min, 78:22 er

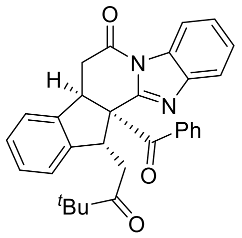

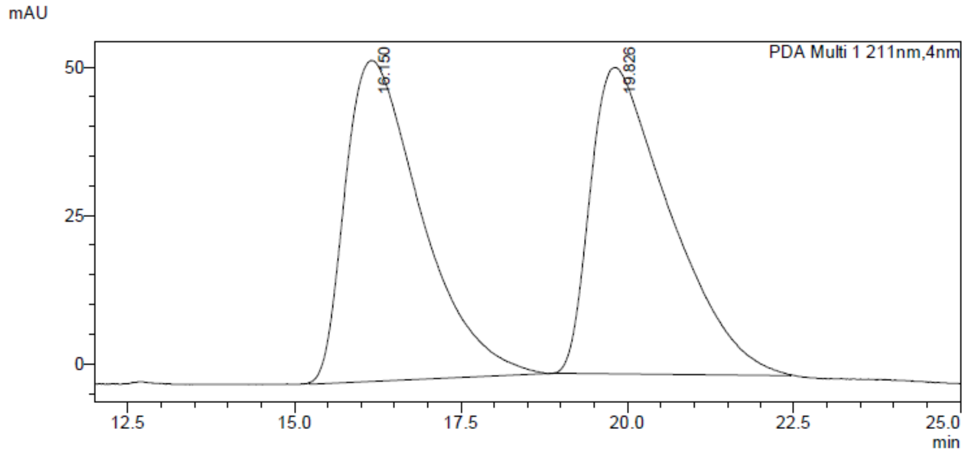

<Peak Table>

PDA Ch1 211nm

Peak#	Ret. Time	Area%
1	16.150	49.935
2	19.826	50.065
Total		100.000

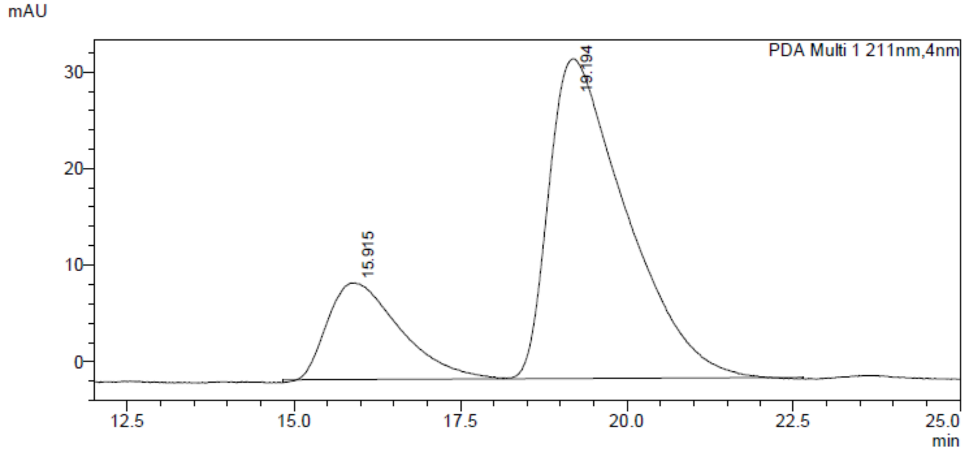

<Peak Table>

PDA Ch1 211nm

Peak#	Ret. Time	Area%
1	15.915	22.057
2	19.194	77.943
Total		100.000

HPLC data for **68**: Chiralpak IB (97:3 hexane : IPA, flow rate 1.5 mLmin^{-1}, 211 nm, 40 °C) t_R minor: 16.1 min, t_R major: 29.7 min, 85:15 er

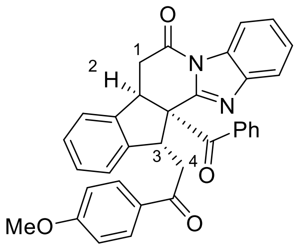

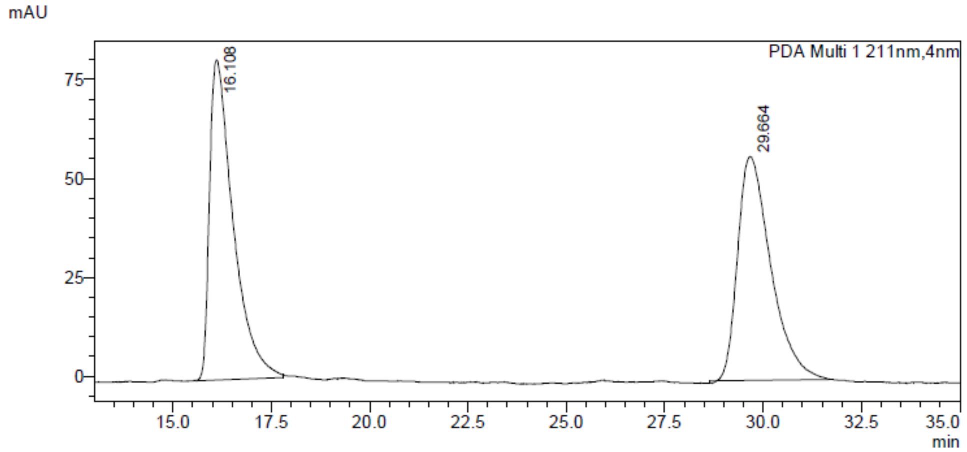

<Peak Table>

PDA Ch1 211nm

Peak#	Ret. Time	Area%
1	16.108	50.529
2	29.664	49.471
Total		100.000

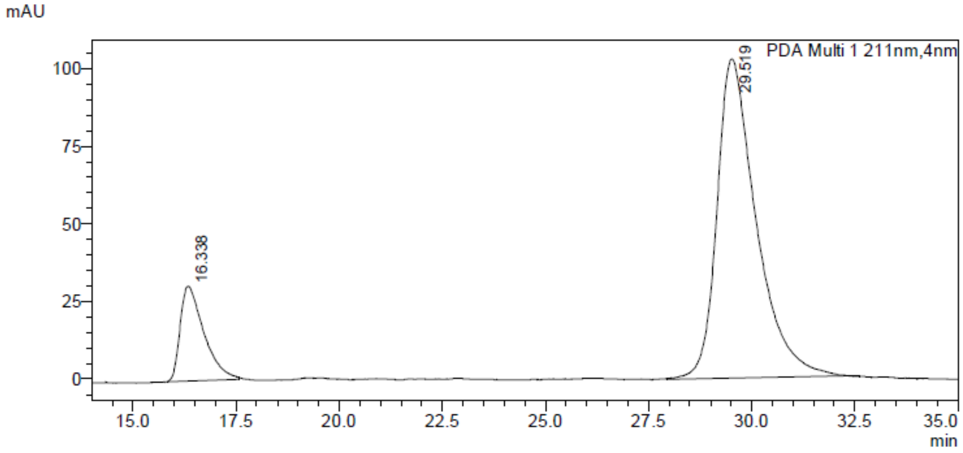

<Peak Table>

PDA Ch1 211nm

Peak#	Ret. Time	Area%
1	16.338	15.232
2	29.519	84.768
Total		100.000
